# Enantioselective Total Synthesis of (−)-Himalensine A via a Palladium and 4-Hydroxyproline Co-catalyzed Desymmetrization of Vinyl-bromide-tethered Cyclohexanones

**DOI:** 10.1021/jacs.2c13710

**Published:** 2023-02-23

**Authors:** Roman Kučera, Sam R. Ellis, Ken Yamazaki, Jack Hayward Cooke, Nikita Chekshin, Kirsten E. Christensen, Trevor A. Hamlin, Darren J. Dixon

**Affiliations:** † Department of Chemistry, Chemical Research Laboratory, University of Oxford, 12 Mansfield Road, Oxford OX1 3TA, U.K.; ‡ Department of Theoretical Chemistry, Amsterdam Institute of Molecular and Life Sciences (AIMMS), and Amsterdam Center for Multiscale Modeling (ACMM), Vrije Universiteit Amsterdam, De Boelelaan 1083, Amsterdam 1081 HV, The Netherlands

## Abstract

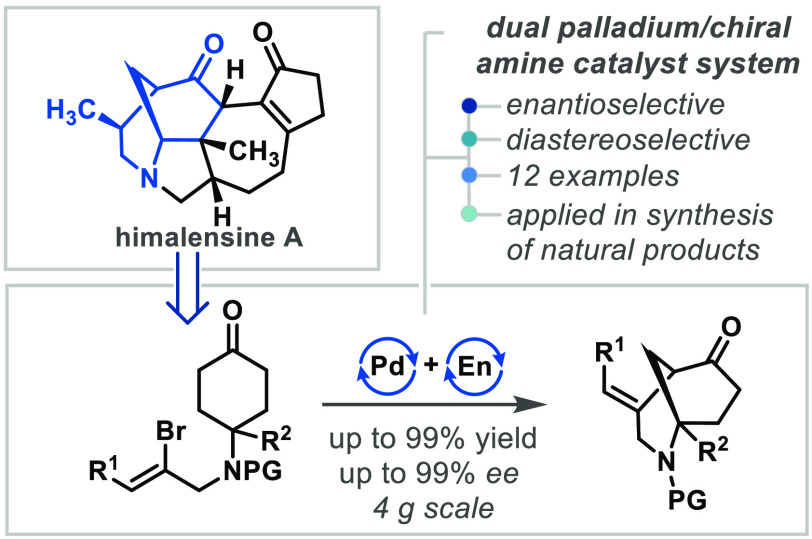

Herein, we describe the convergent enantioselective total synthesis of himalensine A in 18 steps, enabled by a highly enantio- and diastereoselective construction of the morphan core via a palladium/hydroxy proline co-catalyzed desymmetrization of vinyl-bromide-tethered cyclohexanones. The reaction pathway was illuminated by density functional theory calculations, which support an intramolecular Heck reaction of an *in situ*-generated enamine intermediate, where exquisite enantioselectivity arises from intramolecular carboxylate coordination to the vinyl palladium species in the rate- and enantio-determining carbopalladation steps. The reaction tolerates diverse *N*-derivatives, all-carbon quaternary centers, and trisubstituted olefins, providing access to molecular scaffolds found in a range of complex natural products. Following large-scale preparation of a key substrate and installation of a β-substituted enone moiety, the rapid construction of himalensine A was achieved using a highly convergent strategy based on an amide coupling/Michael addition/allylation/ring-closing metathesis sequence which allowed the introduction of three of the five rings in only three synthetic steps (after telescoping). Moreover, our strategy provides a new enantioselective access to a known tetracyclic late-stage intermediate that has been used previously in the synthesis of many *Daphniphyllum* alkaloids.

## Introduction

**Scheme 1:**
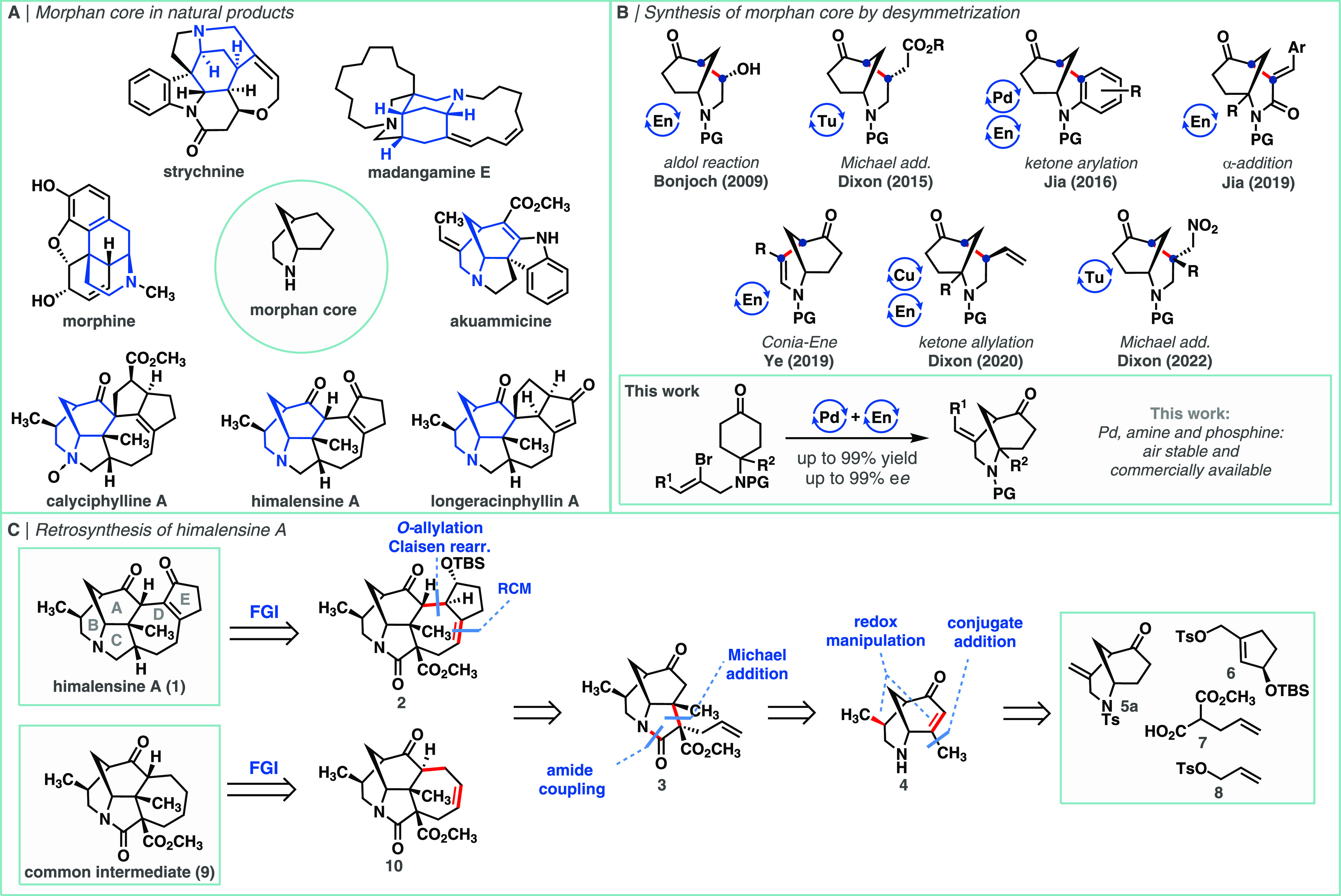
(a) Morphan Core and Selected Relevant Natural Products; (b) Syntheses of the Morphan Core by Enantioselective Desymmetrization; Tu = Thiourea Catalysis, En = Enamine Catalysis; (c) Retrosynthetic Strategy to Himalensine A and Common Intermediate **9**

The 2-azabicyclo[3.3.1]nonane motif, known as the morphan core,^1^ is found in over 300 natural products across a variety of families, including the *Strychnos*,^2^*Morphine*,^3^*Madangamine*,^4^ and *Daphniphyllum* alkaloids^5^ (Scheme 1A). Many of these compounds, such as strychnine^6^ and morphine, exhibit potent biological effects. Thus, such compounds and their derivatives have found widespread pharmaceutical application. Owing to the many potential biological applications of these and related natural products—and often the insufficient quantities available from nature^7^—development of efficient and stereoselective syntheses of these and related complex molecular architectures has been a major research focus in recent years.^5b,8^

In terms of enantioselective approaches, the desymmetrization of structurally simple molecules to rapidly access more architecturally complex and stereochemically defined products is widely considered as one of the most powerful and elegant synthetic strategies.^9^ In this context, the desymmetrization of symmetric carbonyl compounds through intramolecular carbon–carbon bond formation has become a proven asymmetric route to construct complex bridged and fused bicyclic rings. Indeed, impressive contributions from the Bonjoch,^10^ Jia,^11^ and Ye^12^ groups, amongst many others—including our own^13^—have allowed significant progress to be made in this field using enantioselective metal-free or dual amine and metal catalytic approaches (Scheme 1B).

Despite these advances, the application of enantioselective morphan core-generating desymmetrizing technology as a key complexity-building step in natural product synthesis is still in its infancy. To the best of our knowledge, the first such example was our group’s enantioselective total synthesis of madangamine E reported in 2022, wherein bifunctional primary amine catalysis was employed to control a highly enantioselective Michael addition to a pendant β,β-disubstituted nitroalkene (Scheme 1B, bottom right).^13d^ With the intention of broadening this concept to other complex natural product targets, we were drawn toward developing a desymmetrizing vinylation methodology for the enantioselective construction of morphans bearing various synthetically versatile alkylidene groups on the bridging piperidine ring. To date, the highly enantioselective intramolecular α-vinylation of cyclohexanones using vinyl halide-containing substrates has not been disclosed.^14^ Nevertheless, inspired by the work of Jia^11a^ and Bonjoch,^15^ and building on our previous studies, we sought to identify and develop a practical and effective dual amine/palladium catalyst system to enable this transformation and demonstrate its utility in complex natural product synthesis.

The calyciphylline A sub-family of the *Daphniphyllum* alkaloids was chosen as our synthetic target. This class of more than 30 natural products, isolated from the *Daphniphyllum* genus, shares a common [6–6–5–7] tetracyclic core and differs only in the presence and oxidation state of the E/F rings, making it an ideal target for collective synthesis. The structural complexity and biological significance of the members of this family have led to widespread interest from the synthetic community, resulting in multiple total syntheses and even more synthetic studies toward these natural products,^5b^ including our first and enantioselective synthesis of himalensine A.^16^ Among the most notable accomplishments in this field belongs the work of the Li group, achieving a collective total synthesis of 19 natural products from this family, using versatile common tetracyclic intermediate **9**.^17^

In this study, we focused on the synthesis of himalensine A (Scheme 1C). We envisioned that after the retrosynthetic introduction of several synthetic handles, namely, conversion of the amine into an amide, introduction of an ester group and migration of the C=C bond of the enone moiety, the central 7-membered D-ring could be disconnected in a highly convergent fashion employing allylation/ring-closing metathesis (RCM)^18^ to complex allyl tosylate **6**,^19^ representing the E-ring and tricyclic intermediate **3**. Subsequent disconnection of the C-ring by Michael addition and amide coupling took us back to functionalized morphan core **4**, accessible in several steps from the product of enantioselective desymmetrization (**5a**). This new strategy represents a more streamlined approach to himalensine A in comparison to our previous synthesis (22 steps), where we used an enantioselective prototropic shift/furan Diels–Alder cascade for the formation of ACD-tricyclic core followed by stepwise introduction of rings B and E. Moreover, our new strategy could be applied in the synthesis of Li’s common intermediate **9**.^17a,17c,17d^

## Results and Discussion

Our initial investigations focused on the intramolecular desymmetrizing α-vinylation of *N*-Ts-protected amine **11a** using a chiral cyclic secondary amine/Pd(0) dual catalytic system (Table 1, see Supporting Information for full details). The absolute stereochemical configuration of the corresponding cyclized product ***(ent)*-5a** had been previously determined by single-crystal X-ray diffraction studies.^13b^ A preliminary screen (not shown) conducted with *(S)-*proline **(12a)** as the chiral amine catalyst demonstrated that Pd(OAc)_2_ was superior to other Pd(II) or Pd(0) sources, such as PdCl_2_, Pd(PPh_3_)_4_, or Pd_2_(dba)_3_ respectively. In addition, methanol was found to be the optimal solvent. Screening of multiple bases revealed K_2_HPO_4_ to perform best (entries 1–6), and at lower concentrations (entry 7), the cyclized product ***(ent)*-5a** was formed in improved yield with high enantioselectivity (86% ee). Variation of the triarylphosphine ligand led to the observation that *para*-electron-donating groups (such as −OMe, **13b**) resulted in lower reactivity (22% yield, entry 8) compared to PPh_3_. In contrast, the use of triarylphosphines with *para*-electron-withdrawing groups (such as −Cl, **13c**, and −CF_3_, **13d**) led to an improvement in both yield (up to 81%) and enantioselectivity (up to 94% ee, entry 9–10). Using prolinol (**12b**) and prolinamide (**12c**) as the chiral amine catalysts led to poor reactivity, while no reaction was observed when using a proline-tetrazole catalyst (**12d**) (entry 11–13). Employing (2*S*,4*R*)-4-hydroxyproline (**12e**) led to a notable improvement in the outcome of the reaction (entry 14), and ***(ent)*-5a** was formed in excellent yield (95%) and enantioselectivity (92% ee). Interestingly, the use of the C-4 epimeric catalyst **12f** had no significant impact on enantioselectivity when compared with **12e**.

**Table 1:**
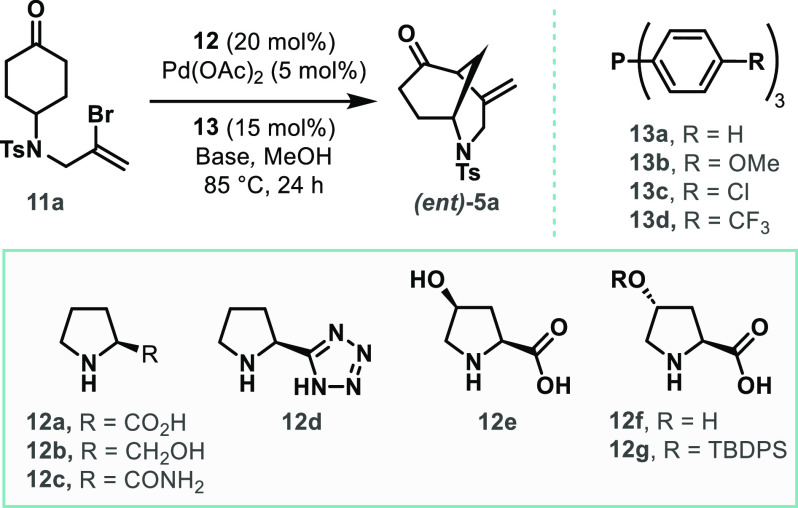
Reaction Optimization^a^

entry	base	amine	ligand	yield (%)^b^	ee (%)^c^
1	K_2_CO_3_	**12a**	**13a**	0	n/a
2	Cs_2_CO_3_	**12a**	**13a**	16	29
3	NaOAc	**12a**	**13a**	15	68
4	K_3_PO_4_	**12a**	**13a**	17	42
5	K_2_HPO_4_	**12a**	**13a**	27	82
6	KH_2_PO_4_	**12a**	**13a**	0	n/a
7^d^	K_2_HPO_4_	**12a**	**13a**	40	86
8^d^	K_2_HPO_4_	**12a**	**13b**	22	66
9^d^	K_2_HPO_4_	**12a**	**13c**	68	94
10^d^	K_2_HPO_4_	**12a**	**13d**	81	93
11	K_2_HPO_4_	**12b**	**13c**	16	n.d.
12	K_2_HPO_4_	**12c**	**13c**	7	n.d.
13	K_2_HPO_4_	**12d**	**13c**	0	n/a
14	K_2_HPO_4_	**12e**	**13c**	95	92
15	K_2_HPO_4_	**12f**	**13c**	95	91
16	K_2_HPO_4_	**12g**	**13c**	66	96
17^d^	K_2_HPO_4_	**12e**	**13d**	95	94
18^d^	K_2_HPO_4_	***(ent)*-12e**	**13d**	95 (*ent*)	94 (*ent*)
19^d^^,^^e^	K_2_HPO_4_	***(ent)*-12e**	**13d**	0	n/a
20^d^		***(ent)*-12e**	**13d**	0	n/a
21^d^	K_2_HPO_4_		**13d**	0	n/a
22^d^^,^^f^	K_2_HPO_4_		**13d**	23	0

a Reagents and conditions: **11a** (0.10 mmol), **12** (20 mol%), Pd(OAc)_2_ (5 mol%), **13** (15 mol%), base (1.5 equiv), MeOH (0.10 M), 85 °C, 24 h.

b Determined by ^1^H NMR analysis of crude reaction mixtures using mesitylene as an internal standard.

c Determined by chiral HPLC analysis.

d Concentration of 0.05 M.

e No Pd(OAc)_2_.

f Pyrrolidine (20 mol%) used.

The silyl-protected 4-hydroxyproline catalyst **12g** also led to the formation of ***(ent)*-5a** with excellent enantioselectivity (96% ee), albeit in lower yields (66%, entry 16). The combination of (2*S*,4*S*)-4-hydroxyproline (**12e**) as the chiral amine catalyst and *para*-trifluoromethyl-substituted triarylphosphine **13d** as the ligand led to further improvement in both yield and enantioselectivity; either enantiomer of the product ***((ent)*-5a** or **5a)** could be prepared in excellent yield (95%) and enantioselectivity (94% ee) by employing **12e** or ***(ent)-*12e**, respectively (entry 17–18). Finally, control studies were carried out to ascertain the importance of each reaction component. In the absence of Pd(OAc)_2_, K_2_HPO_4_, or amine catalyst **12e**, no reaction was observed (entries 19–21). Interestingly, using pyrrolidine as the amine catalyst led to the formation of the racemic product in low yield (23%), suggesting that the carboxylate moiety is necessary to enable both optimal reactivity and enantioselectivity (entry 22).

**Scheme 2:**
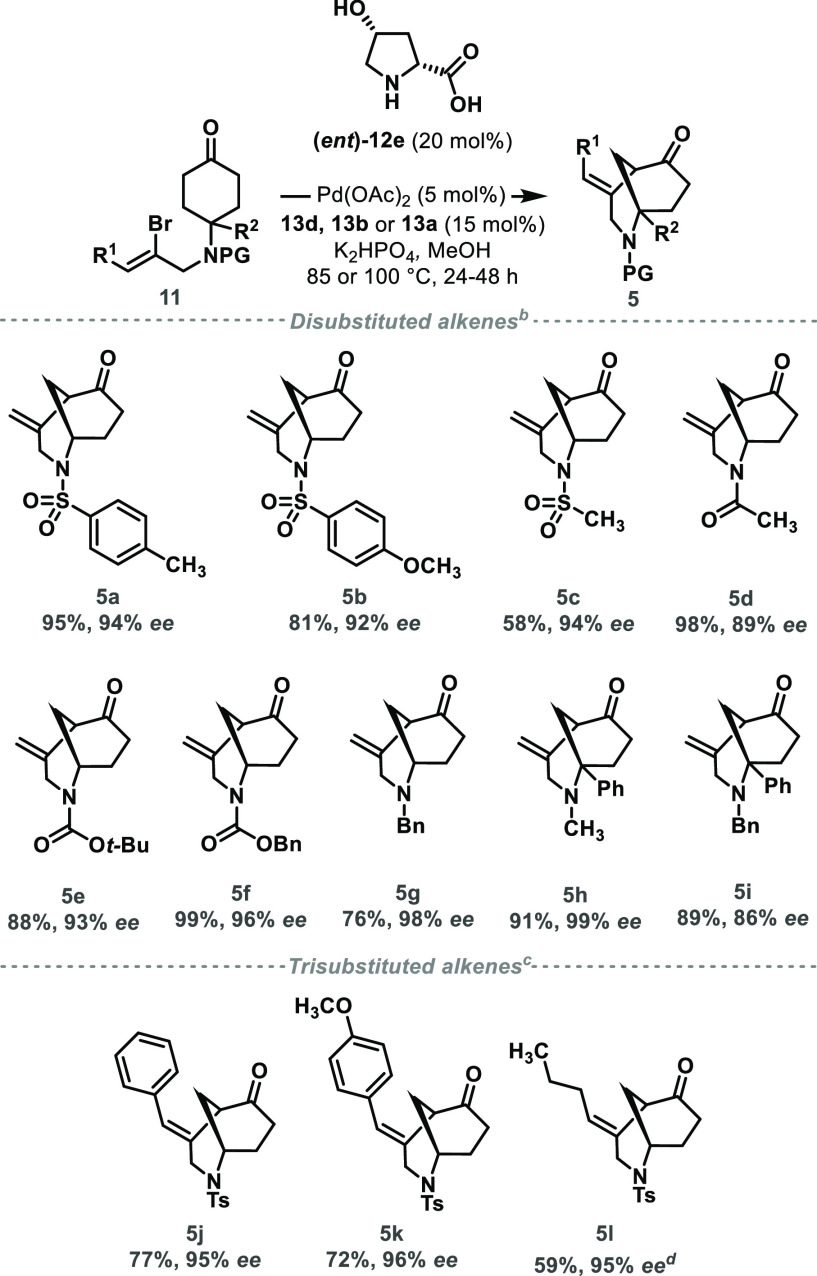
Scope of the Desymmetrizing Vinylation Reaction Reagents and conditions: ^b^**11** (0.10 mmol), ***(ent)*-12e** (20 mol%), Pd(OAc)_2_ (5 mol%), **13d** (15 mol%), K_2_HPO_4_ (1.5 equiv), MeOH (0.05 M), 85 °C, 24–48 h. ^c^**13b** used instead of **13d**, 100 °C. ^d^**13a** used instead of **13d**, 100 °C. Isolated yields; enantioselectivity determined by chiral HPLC analysis.

A range of substrates was subjected to the optimal reaction conditions and found to be well tolerated (Scheme 2). Arylsulfonamides **11a** and **11b** underwent cyclization in good yield with excellent levels of enantioselectivity (up to 94% ee), which further translated to alkylsulfonamide **11c**, albeit forming bicycle **5c** in slightly reduced yield. Amides and carbamates also performed well, affording **5d–5f** in excellent yields (88–99%) and enantioselectivities (89–96% ee). In addition, an *N*-Bn-protected bicycle **5g** was efficiently formed (76% yield) with excellent enantioselectivity (98% ee). Furthermore, the reaction conditions tolerated alkyl-substituted amines featuring fully-substituted α-carbon centers, as exemplified by the synthesis of **5h** and **5i** in good yield (up to 91%) and excellent enantioselectivity (up to 99% ee).

In order to broaden the scope of this reaction, we investigated the suitability of substrates bearing trisubstituted olefins. Interestingly, and in contrast to disubstituted substrates, optimal yields and enantioselectivities required higher reaction temperatures (100 °C) and the use of more electron-rich triarylphosphine ligands. Both phenyl **5j** and *p*-MeOC_6_H_4_-substituted olefin **5k** were obtained in high yield (77% and 72%, respectively) and excellent enantioselectivity (95% ee and 96% ee, respectively), when using phosphine **13b** bearing *para*-electron-donating methoxy group as the ligand. Furthermore, dialkyl-substituted vinyl bromides required a compromise between reactivity (favored by ligands bearing electron-withdrawing groups) and enantioselectivity (favored by ligands bearing electron-donating groups), with the use of triphenylphosphine (**13a**) affording **5l** in moderate yield (59%) while maintaining excellent levels of enantiocontrol (95% ee).

**Scheme 3:**
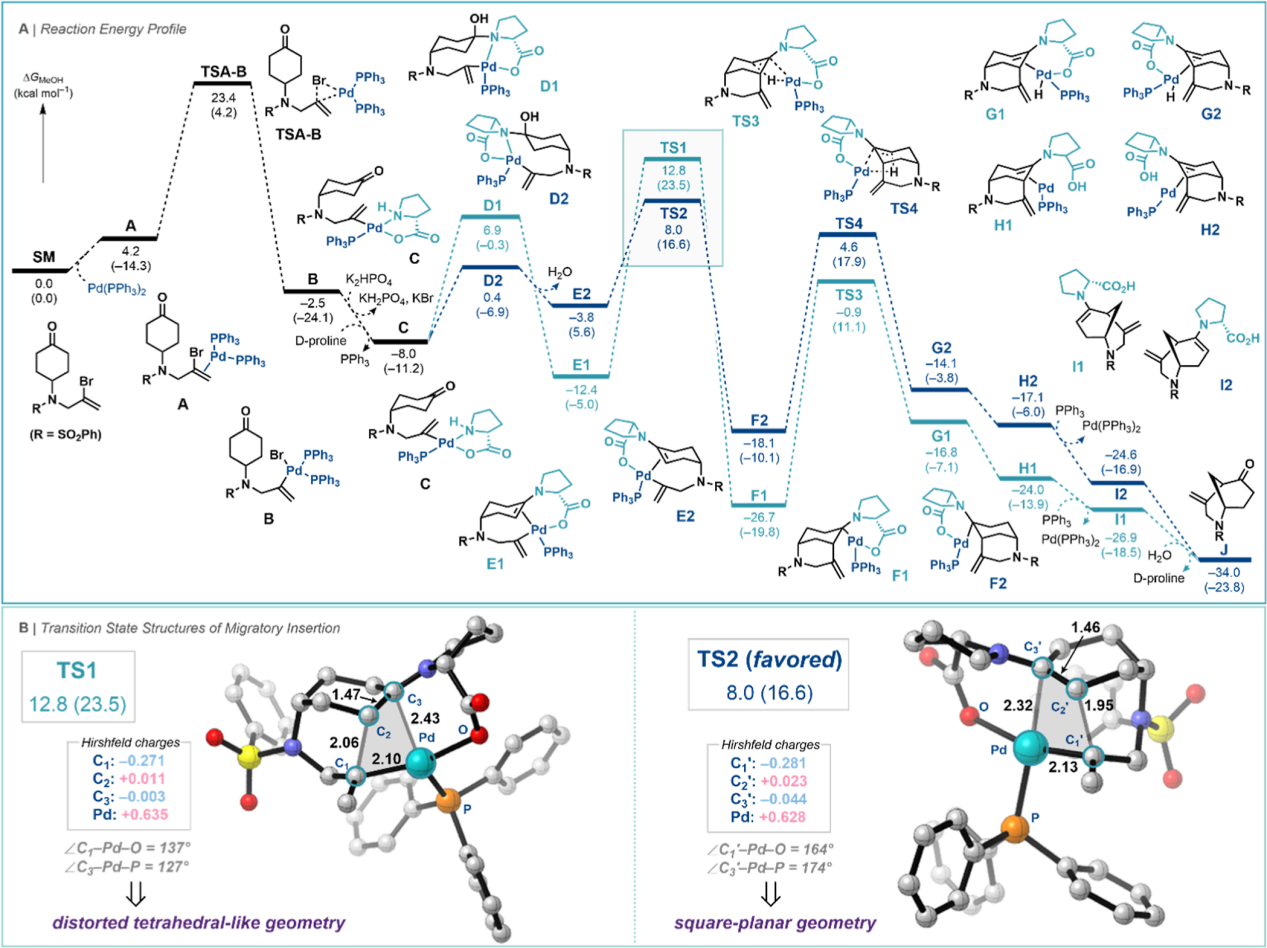
Computed Reaction Energy Profile (Δ*G* [Δ*E*] in kcal mol^–1^) for the Desymmetrization of Vinyl-bromide-tethered Cyclohexanones and the Transition Structures of Migratory Insertion Computed at COSMO(MeOH)-ZORA-M06/TZ2P//COSMO(MeOH)-ZORA-BLYP-D3(BJ)/TZ2P Bond lengths (Å), bond angles (deg) and Hirshfeld charges (a.u.) of key atoms of the distorted reactants in their corresponding TS geometries (hydrogen atoms were removed for clarity) are provided in the inset. R = SO_2_Ph.

To further understand the nature of this reaction, including the roles of both catalysts and the origin of the high enantioselectivity, the intramolecular vinylation reaction was investigated computationally through density functional theory (DFT) calculations [COSMO(MeOH)-ZORA-M06/TZ2P//COSMO(MeOH)-ZORA-BLYP-D3(BJ)/TZ2P using ADF]^20^ using vinyl bromide **SM** as a model substrate and proline as the chiral cyclic secondary amine catalyst (Scheme 3A). The computed reaction pathway begins with the complexation of the vinyl-bromide substrate and a catalytically active palladium(0) species, Pd(PPh_3_)_2_, formed *in situ*, to form metal complex **A**.^21^ Oxidative addition of the vinyl bromide to palladium(0) through **TSA-B**, then proceeds with an energy barrier of 19.2 kcal mol^–1^, forming the Pd–C bond found in palladium(II) complex **B**. In the presence of the proline catalyst and a base, the chelation of proline to the complex **B** is exergonic by 5.5 kcal mol^–1^ and generates intermediate **C**. The ligated nitrogen atom can then react with the ketone to form hemiaminals **D1** or **D2**, generating enamines **E1** or **E2** after the elimination of water. Interestingly, the hemiaminal species **D2** is more stable than **D1**, but the enamine species **E2** is less stable than **E1**. The subsequent enantio determining C–C bond-forming migratory insertion step from **E1** and **E2** has a large energy difference in transition state energies (ΔΔ*G*^‡^ = 4.8 kcal mol^–1^), indicating that this step preferably proceeds through the lower-energy transition structure **TS2**. The origin of the kinetic preference for **TS2** likely originates from the smaller interatomic distances between the positively charged Pd atom and the negatively charged atoms of the substrate to which it is bound, which maximizes the stabilizing electrostatic interactions compared to **TS1**. The shorter catalyst–substrate contact in **TS2** originates from the square-planar geometry of Pd (*trans* L–Pd–L bond angles are nearly 180°), whereas for **TS1**, it is a distorted tetrahedral-like TS.^22^ Therefore, the TS that can adopt the square-planar geometry around the Pd center leads to a lower energy barrier. The intermediate **F2** then undergoes a β-hydride elimination via **TS4**, which has a lower Gibbs free energy than **TS2**, indicating that the C–C bond-forming migratory insertion step is an irreversible and stereoselectivity-determining step. After the β-hydride elimination, the intermediate **G2** is formed, which then furnishes the enamine–Pd(0) complex **H2** after reductive elimination. The final step of the reaction is the dissociation of the cyclized enamine **I2** to regenerate Pd(PPh_3_)_2_ in the catalytic cycle, and **I2** is subsequently hydrolyzed to give the product **J**.

Having probed the scope of the vinylation reaction and elucidated the reaction pathway and origins of stereocontrol computationally using DFT calculations, the stage was set to demonstrate the utility of this chemistry in the context of complex natural product target synthesis. We chose the recently isolated *Daphniphyllum* alkaloid himalensine A^7b^ as our target molecule, but, in the first instance, we were drawn to showcase our new methodology in the streamlined synthesis of tetracycle **9**. Tetracycle **9**, synthetically available in 15 steps,^17a^ holds the privileged position of being the molecular access point to 19 natural products to date.^17^ Furthermore, we recognized that developing chemistry to compound **9** could form the foundation of a new efficient route to himalensine A.

Our synthetic approach to **9** is shown in Scheme 1. We proposed tricycle **3** as a versatile synthetic intermediate that we aimed to access in isomerically pure form, as it could serve as the point of divergence toward the synthesis of both **9** and himalensine A (**1**). Previous work from our group^18^ and Li’s group^23^ indicated that a diastereoselective intramolecular Michael addition of a malonamate group formed from the homologated morphan **4** would indeed provide the requisite reactivity and diastereocontrol. A subsequent α-allylation of the cyclohexanone group, RCM, and hydrogenation should then afford the target tetracycle **9**. Homologated morphan **4** was anticipated to arise in only a few steps from **5**, a desymmetrized product accessible in high yield and enantioselectivity using our new methodology.

**Scheme 4:**
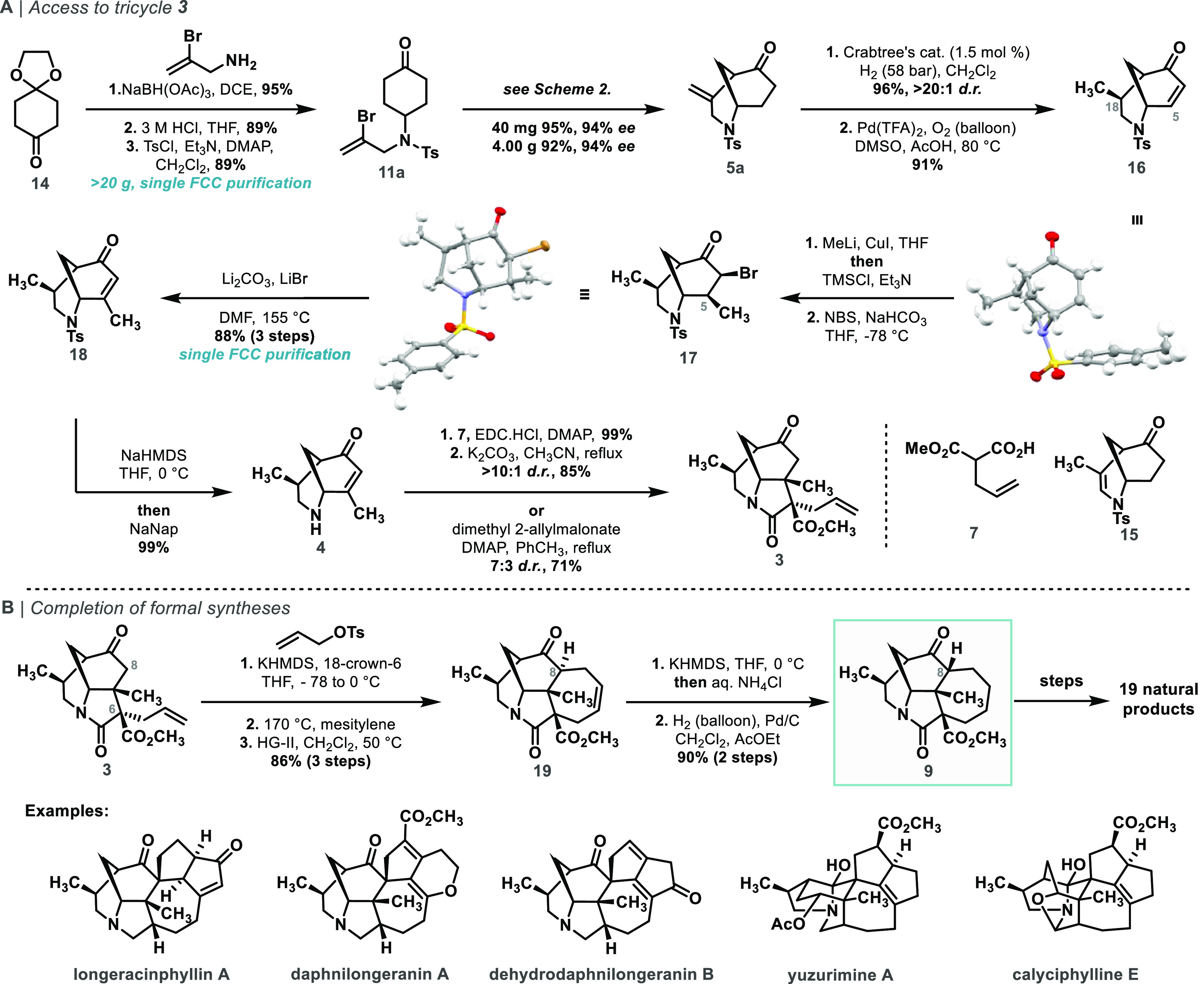
(a) Synthesis of Tricyclic Intermediate **3**; (b) Formal Synthesis of *Daphniphyllum* Alkaloids

The synthesis of the key ketone vinylation substrate **11a** was achieved by a highly efficient three-step sequence from monoprotected diketone **14** (Scheme 4A). Reductive amination of **14** with 2-bromoprop-2-en-1-amine followed by ketal hydrolysis and amine tosylation under standard conditions afforded compound **11a** on decagram scale with purification only required after the final step. Pleasingly, upscaling of the key vinylation reaction from 40 mg to 4.0 g was straightforward, and the desired cyclized product **5a**(24) was obtained in 92% yield and 94% ee.

With access to sufficient quantities of enantiomerically enriched bicyclic morphan core, we focused on its elaboration toward bicycle **4**. This required the stereoselective reduction of the exocyclic double bond, installation of the C-5 methyl group, and subsequent oxidation to afford the enone moiety necessary for the construction of the 5-membered C-ring by Michael addition.^100^

In order to ensure the 18*S* configuration in **16**,^24^ the hydrogenation of the *exo*-methylene group from the more hindered concave face was necessary. Unsurprisingly, hydrogenation in the presence of Pd/C resulted in the formation of a 3:1 mixture of diastereomers, favoring the undesired 18*R* diastereomer. Additionally, partial isomerization to internal alkene containing bicycle **15** was observed. On the other hand, directed hydrogenation with Crabtree’s catalyst, previously used in the synthesis of calyciphylline A-type alkaloids by Dixon,^16^ Li,^17a^ Qui,^25^ and Zhai^26^ led to good diastereoselectivity (>20:1 d.r.), although considerable double bond migration was observed (4:3 in favor of hydrogenation). Intensive optimization of the reaction conditions revealed the positive effect of the hydrogen pressure on the ratio of hydrogenated product (**S28**) to isomer **15**, due to acceleration of the hydrogenation reaction at higher pressure in comparison to double bond migration. An increase of hydrogen pressure to 9 bar significantly suppressed the isomer formation (6:1 **S28**/**15**) while maintaining excellent facial selectivity (>20:1). Further increase of hydrogen pressure to 58 bar almost completely suppressed double bond migration (22:1 **S28**/**15**, d.r. >20:1) and allowed the reduction of catalyst loading to only 1.5 mol%. To install the C-5 methyl group through a conjugate addition, ketone **S25** was converted to enone **16** under Stahl’s conditions,^27^ which was then treated with the Gilman reagent Me_2_CuLi.LiI (generated *in situ* from MeLi and CuI) in the presence of TMSCl. Unfortunately, the increased steric hindrance introduced by the newly installed methyl group made the re-oxidation of the resulting ketone to the conjugated enone very challenging and many commonly used methods failed to facilitate the desired dehydrogenation on our system (for details see Supporting Information). To tackle this issue, the silyl enol ether obtained in the cuprate addition (**S29**) was brominated using NBS and then dehydrobrominated in excellent yield using Li_2_CO_3_ and LiBr. Moreover, the methylation, bromination, and elimination sequence required only a single chromatographic purification and was scalable to over 10 g. Both methyl addition and bromination products **S29** and **17**, respectively, were obtained as a single diastereomers whose relative stereochemical configuration was confirmed by single-crystal X-ray diffraction analysis.^24^ Finally, a highly chemoselective tosyl group cleavage was achieved by treatment of **18** with sodium naphthalenide after prior *in situ* protection of the enone moiety as its extended sodium enolate.

For the C- and D-ring formation, we planned to utilize a highly convergent intramolecular Michael addition/allylation/RCM approach as developed previously in our group during preliminary studies toward calyciphylline A-type alkaloids.^18^ To this end, secondary amine **4** was coupled with malonate **7** using the 1-ethyl-3-(3-dimethylaminopropyl)carbodiimide hydrogen chloride complex (EDC.HCl), and the obtained malonamate was subjected to K_2_CO_3_ in acetonitrile which afforded the desired tricyclic compound **3** in good d.r. (>10:1) and yield (85% of the desired diastereomer, Scheme 4A).^23^ Interestingly, compound **3** could also be obtained efficiently in one step from amine **4** by 4-dimethylaminopyridine catalyzed transamidation-Michael addition with dimethyl 2-allylmalonate, albeit in lower diastereoselectivity (7:3 d.r., 71% yield of the desired isomer).

Next, the C-8 allyl group was installed in a two-step *O*-allylation/Claisen rearrangement sequence (Scheme 4B). To avoid undesired epimerization of C-6 through reversible Michael addition, ketone **3** was deprotonated with potassium hexamethyl-disilazide (KHMDS) at −78 °C, and the resulting enolate was treated with allyl tosylate in the presence of 18-crown-6. This protocol allowed the isolation of enol ether **19** in excellent yield as a single diastereomer. Heating the obtained intermediate to 170 °C in mesitylene yielded the Claisen rearrangement product, which after exposure to Hoveyda–Grubbs 2^nd^ generation catalyst (HG-II) underwent the desired RCM to provide tetracyclic compound **19** in excellent yield (86% over 3 steps). Treatment of **19** with KHMDS followed by a protic work up led to epimerization of C-8. Notably, the presence of the alkene bond in **19** was essential for the epimerization to occur, as the hydrogenated analogue of **19** did not undergo epimerization under the same conditions. Finally, alkene hydrogenation of the C-8 epimerization product concluded the synthesis of compound **9**, a versatile intermediate in *Daphniphyllum* alkaloid synthesis.^17^

**Scheme 5:**
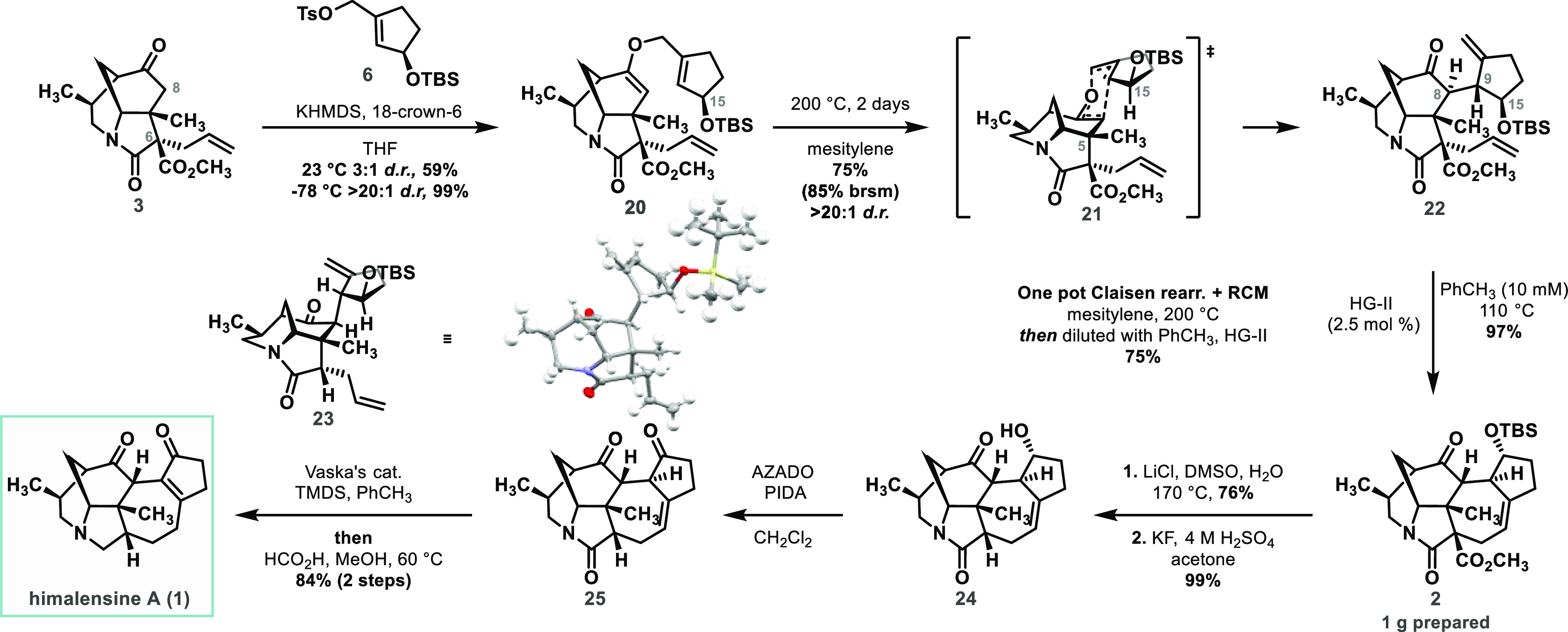
Total Synthesis of Himalensine A

Having successfully synthesized **9** using the robust *O*-allylation/Claisen rearrangement/RCM sequence to install the D-ring, our focus then turned to himalensine A (Scheme 5). Inspired by the work of Carreira,^19c^ our plan was to use more complex allyl tosylate **6** for direct introduction of the E-ring of this natural product. The previously described strategy would form the carbon skeleton of himalensine A, and functional group and redox manipulations would then afford the target natural product.

Deprotonation of tricyclic ketone **3** with KHMDS at low temperature and treatment of the resulting enolate with complex allyl tosylate **6** provided desired enol ether **20** in excellent yield (Scheme 5). Upon heating to 200 °C, compound **20** underwent smooth Claisen rearrangement, presumably through chair-like transition state (**21**), setting up two adjacent tertiary stereogenic centres. The relative stereochemical configuration of **22** was established by single-crystal X-ray diffraction analysis of analogous compound **23**(24) prepared in the same manner (see Supporting Information). It is worth noting that the Claisen reaction performed with a 1:1 mixture of diastereomers obtained by coupling of **3** and racemic allyl tosylate **6** only proceeded with 50% conversion. In this case, only the 15*R* diastereomer, with a bulky OTBS group pointing away from C-5 methyl group, was consumed in the reaction, while the 15*S* diastereomer remained unreacted.

We anticipated that the RCM leading to the formation of the D-ring might be more challenging than it was in the case of the synthesis of compound **9**, not only because it requires the formation of a 7-membered ring bearing a tri-substituted double bond but also because of the potentially restricted rotation around the C-8 and C-9 bonds, which could prevent the adoption of the reactive conformation. Pleasingly, however, treatment of diene **22** with the Hoveyda–Grubbs 2^nd^ generation catalyst resulted in the smooth formation of the D-ring accompanied by C-8 epimerization, leading to thermodynamically more stable bowl-shaped epimer, in excellent yield. Moreover, the sequence of Claisen rearrangement and RCM could be performed in a one-pot process with unchanged yield on gram scale. It is also worth noting that RCM attempted with decarboxylated compound **23** was not productive, and only products of dimerization and double bond migration were observed, indicating the crucial role of the ester group in this cyclization, possibly due to the Thorpe–Ingold effect.

At this point, the methyl ester group in **2** was removed by Krapcho decarboxylation, and the TBS group was cleaved under acidic conditions. A challenging oxidation of sterically hindered alcohol **24** was realized by phenyliodine(III)diacetate (PIDA) in the presence of a 2-azaadamantane *N*-oxyl catalyst (AZADO).^28^ Purification of the oxidized product (**25**) on silica resulted in alkene migration, providing oxy-himalensine A and thus completing the formal synthesis of this natural product. Alternatively, the crude product of oxidation **25** could be subjected to reduction conditions described in our previous synthesis of himalensine A.^16^ Employing this protocol, the lactam in **25** was first reduced to the corresponding silylated hemiaminal using Vaska’s catalyst in the presence of tetramethyldisiloxane (TMDS), which upon treatment with formic acid, was further reduced to the desired pyrrolidine ring. Concomitant migration of the double bond into conjugation with the carbonyl of the cyclopentanone E-ring accomplished the total synthesis of himalensine A in 20 steps and 10% overall yield (18 steps and 9% yield after telescoping).

## Conclusions

In conclusion, we have developed a new enantioselective synthesis of himalensine A. Key to the rapid construction of this natural product was a highly convergent strategy based on an amide coupling/Michael addition/allylation/RCM sequence, which allowed for the introduction of three of the five rings in only five synthetic steps (three steps after telescoping). Moreover, the same strategy provided access to tetracyclic compound **9**, a common intermediate in the synthesis of multiple *Daphniphyllum* alkaloids. Most notably, the synthesis of the functionalized morphan core was enabled by the development of a new highly enantioselective desymmetrizing α-vinylation of cyclohexanones using a dual palladium/4-hydroxyproline catalyst system. The method provides access to a range of morphan core derivatives in high yields and with excellent levels of enantioselectivity, tolerating many commonly utilized functionalities. DFT computations revealed that the reaction proceeds via an intramolecular Heck reaction of an enamine intermediate formed *in situ* by condensation of the hydroxyproline catalyst with the cyclohexanone, where exquisite enantioselectivity arises from carboxylate coordination to the vinyl palladium species in the rate- and enantio-determining carbopalladation step.

## Supporting Information Available

Additional optimization data, full synthetic methods, and characterization data (PDF)
