# Supplementary material for: Enantioselective Total Synthesis of (−)-Himalensine A via a Palladium and 4-Hydroxyproline Co-catalyzed Desymmetrization of Vinyl-bromide-tethered Cyclohexanones
Source: J Am Chem Soc. 2023 Feb 23;145(9):5422–30. doi: 10.1021/jacs.2c13710 (PMC9999414; doi:10.1021/jacs.2c13710)
Supplement: Supplementary file 1 — ja2c13710_si_001.pdf [file ja2c13710_si_001.pdf]

Supporting Information for

Enantioselective Total Synthesis of (–)-Himalensine A via a Palladium and 4-Hydroxyproline Co-catalyzed Desymmetrization of Vinyl-bromide-tethered Cyclohexanones

Roman Kučera,[1,†] Sam R. Ellis,[1,†] Ken Yamazaki,[1,2,‡] Jack Hayward Cooke,[1,‡] Nikita Chekshin,[1] Kirsten E. Christensen,[1] Trevor A. Hamlin[*,2], and Darren J. Dixon[*,1]

[1] Department of Chemistry, University of Oxford, Chemical Research Laboratory, 12 Mansfield Road, Oxford, OX1 3TA, UK. *e-mail: darren.dixon@chem.ox.ac.uk

[2] Department of Theoretical Chemistry, Amsterdam Institute of Molecular and Life Sciences (AIMMS), and Amsterdam Center for Multiscale Modeling (ACMM), Vrije Universiteit Amsterdam, De Boelelaan 1083, 1081 HV Amsterdam (The Netherlands) *e-mail: t.a.hamlin@vu.nl

Table of Contents

1 General Experimental Details 4

1.1 General Information 4

1.2 Characterization 4

2 Computational Studies 6

2.1 Computational Methods 6

3 Synthetic procedures 7

3.1 Optimization Tables 7

3.1.1 Preliminary Studies 7

3.1.2 Optimization of Base and Reaction Concentration 7

3.1.3 Optimization of Phosphine Ligand 9

3.1.4 Optimization of Organocatalyst 10

3.1.5 Optimal Conditions 11

3.2 Synthesis of Starting Materials 12

3.2.1 Synthesis of Substrates 11a – 11g 12

3.2.2 General Procedure A – Sulfonylation of Secondary Amines 15

3.2.3 Synthesis of Substrates 11h and 11i 22

3.2.4 Synthesis of Trisubstituted Alkene Substrates 11j, 11k and 11l 30

3.3 Pd-catalyzed cyclization 44

3.3.1 General Procedure B – Enantioselective Cyclization 44

3.3.2 Cyclized Products 5a–5l 45

3.4 Synthesis of Daphniphyllum alkaloids 58

3.4.1 Synthesis and functionalization of the morphan core 58

3.4.2 Introduction of the C-ring 76

3.4.3 Formal synthesis of Daphniphyllum alkaloids 80

3.4.4 Introduction of the D-ring of himalensine A 88

3.4.5 The endgame of the synthesis of himalensine A 96

3.4.6 Unsuccessful strategy towards himalensine A through intermediate 23: 105

3.5 Synthesis of building block, catalysts, and reagents 110

3.5.1 Preparation of CBS catalyst 111

3.5.2 Preparation of building blocks for *O*-allylation 113

3.5.3 Synthesis of E-ring fragment of himalensine A 114

3.5.4 Preparation of building block for amide coupling... 123

3.5.5 Preparation of ruthenium scavenger 124

4 NMR Spectra..127

5 HPLC Traces 215

6 Crystallographic Data 232

7 Computational Details..236

8 References...303

1 General Experimental Details

1.1 General Information

Reactions were carried out under a nitrogen atmosphere unless stated otherwise. Glassware was oven-dried and cooled under a vacuum, then purged with nitrogen before use. Room temperature refers to 22 ± 2 °C. Reaction temperatures refer to external temperatures, for example, of a heating block or oil bath, not of internal reaction temperatures unless stated otherwise.

Reagents and solvents from commercial sources were used as supplied unless stated otherwise. Where solvent dryness was important, the solvents were obtained from an MBRAUN-SPS solvent purification system in which solvent is passed through an activated alumina column under nitrogen or standing over 3 Å molecular sieves under an atmosphere of argon.

Reactions were monitored using thin layer chromatography (TLC) using Merck aluminium-backed DC60 F254 plates (particle size 0.2 mm). TLC sheets were visualized by UV light and then developed by staining with potassium permanganate. Purification by column chromatography was carried out using Merck silica gel 60 F254 (particle size 43–60 μm).

1.2 Characterization

Proton (^1H) and carbon (^{13}C) spectra were recorded on Bruker AVG400 (400/101 MHz), Bruker AVH400 (400/101 MHz), Bruker AVF400 (400/101 MHz), Bruker AVB500 (500/126 MHz), Bruker AVC500 (500/126 MHz), and Bruker DPX200 (200 MHz) NMR spectrometers. Spectra are referenced to the residual solvent peak ($CHCl_3$: δ_H 7.26, δ_C 77.16 ppm), chemical shifts (δ) are given in parts per million (ppm, ± 0.01), and coupling constants are given in Hertz (Hz) to the nearest 0.5 Hz. Peak multiplicities are described as singlet (s), doublet (d), triplet (t), quartet (q), pentet (p), a combination, e.g. doublet of doublets (dd), or as a multiplet (m) over a peak range. Additionally, peaks may be described as broad (br), or apparent (app).

Melting points were recorded using a Leica Galen III hot-stage microscope apparatus and are reported uncorrected in degrees Celsius (°C). Infrared spectra were recorded using a Bruker Tensor 27 FT-IR spectrometer as a thin film. Selected diagnostic absorption maxima ($\tilde{\nu}_{max}$) are reported in wavenumbers (cm^{-1}). Low-resolution mass spectra were acquired using a Micromass LCT Premier spectrometer (ESI). High-resolution mass spectra were recorded by Chemistry Research Laboratory staff using a Bruker Daltronics MicroTOF spectrometer (ESI). Mass-to-charge ratios (*m*/*z*) are reported in Daltons. HPLC separation of racemic and enantioenriched compounds was carried out using an

Agilent Technologies 1200 series HPLC employing a chiral stationary phase column and a mobile phase as specified in the relevant experiments.

Optical rotations were recorded using a Perkin Elmer 241 optical activity polarimeter at 25 °C. Specific rotations $[\alpha]_D^{25}$ are reported in 10^{-1} deg cm^2 g^{-1}, with concentrations (c) in g/100 mL, and D refers to the D-line of sodium (589 nm).

2 Computational Studies

2.1 Computational Methods

All calculations reported in this paper were performed using the Amsterdam Density Functional (ADF) software.[1] Equilibrium and transition structure geometries were optimized using the BLYP functional[2,3] and the TZ2P basis set.[4] This approach was extensively tested against ab initio reference benchmarks from hierarchical series up till CCSD(T).[5] The solvent effects of methanol were accounted for using the conducter-like screen model (COSMO) of solvation.[6] Dispersion interactions were included using Grimme's DFT-D3 correction with Becke-Johnson damping.[7] The zeroth-order regular approximation (ZORA) was used to account for scalar relativistic effects.[8] This level is referred to as COSMO(MeOH)-ZORA-BLYP-D3(BJ)/TZ2P. All stationary points have been verified, through vibrational analysis, to be minima (zero imaginary frequencies) or transition structures (one imaginary frequency). The character of the normal mode associated with the imaginary frequency has been analyzed to ensure it resembles the reaction under consideration. Optimized structures were illustrated using CYLview.[9] Potential energies were refined by means of single-point calculations using the M06 functional[10] and the TZP2 basis test.[4] This level is denoted COSMO(MeOH)-ZORA-M06/TZ2P//COSMO(MeOH)-ZORA-BLYP-D3(BJ)/TZ2P.

For the thermochemistry calculations, we used a standard approach whereby the geometries were optimized, and the vibrational frequencies were obtained through numerical differentiation of the analytical gradient. Enthalpies at 298.15 K and 1 atm ($\Delta H°$) were calculated from the electronic bond energies and vibrational frequencies by using a standard thermochemistry relation for an ideal gas [Eq. 1].

$$\Delta H° = \Delta E_{trans,298} + \Delta E_{rot,298} + \Delta E_{vib,0} + \Delta(\Delta E_{vib,298}) + \Delta(pV) \qquad (1)$$

$\Delta E_{trans,298}$, $\Delta E_{rot,298}$, and $\Delta E_{vib,0}$ are the differences between the reactants in the translational, rotational, and zero-point vibrational energy, respectively, whereas $\Delta E_{vib,298}$ takes the vibrational energy change upon going from 0 to 298.15 K into account. The vibrational energy corrections and the entropic term $T\Delta S°$ are based on frequency calculations. Thermal corrections for the electronic term are neglected and $\Delta(pV) \approx \Delta(nRT)$ was used. The change of the Gibbs free energy (ΔG) in both the gas and condensed phase was then calculated for 298.15 K and 1 atm ($\Delta G°$) [Eq. 2].

$$\Delta G° = \Delta H - T\,\Delta\,S° \qquad (2)$$

3 Synthetic procedures

3.1 Optimization Tables

3.1.1 Preliminary Studies

Preliminary investigations demonstrated that $Pd(OAc)_2$ was superior to other Pd(II) (such as $PdCl_2$) or Pd(0) catalysts (such as $Pd(PPh_3)_4$ or $Pd_2(dba)_3$). No reaction was observed using Ni(II) catalysts. In addition, MeOH was found to be a superior solvent; EtOH and *i*PrOH behaved similarly, while all others tested (such as THF, MeCN, and DMF) resulted in poor reactivity. A temperature of 85 °C was employed, as no reaction took place at lower temperatures (<80 °C), while higher temperatures (>90 °C) offered no improvement in enantioselectivity and led to decomposition taking place.

3.1.2 Optimization of Base and Reaction Concentration

Using a $Pd(OAc)_2/PPh_3$/L-Pro-OH catalyst system in MeOH at 85 °C, the effect of different bases was investigated. In the absence of base (Entry 1), or in the presence of acid (AcOH, Entry 2), acetal formation of the starting material was observed, and therefore, no reaction took place. A range of bases tested led to the formation of the cyclized product with moderate enantioselectivity and low yields (Entries 4–6). A combination of K_3PO_4 and AcOH (1:1) improved both yield and enantioselectivity, and investigating the corresponding conjugate base and acids identified optimal results using KOAc (Entry 8) and K_2HPO_4 (Entry 9). As the reaction using K_2HPO_4 was cleaner than that using KOAc (a better correlation between conversion and yield), it was selected as the base going forward. The use of KH_2PO_4 led to the acetal formation of the starting material.

Carrying out the reaction at higher concentrations (0.20 M, Entry 11) reduced the yield, while lower concentrations (0.05 M, Entry 12) improved the yield to 40%, with a slight improvement in enantioselectivity (86%).

Table S1. Optimization of base.

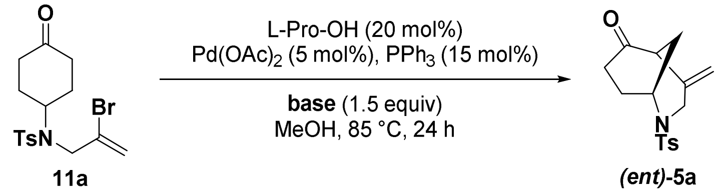

Entry	Base	Yield (%)	*ee* (%)
1	None	0 [a]	n/a
2 [b]	None	0 [a]	n/a
3	K_2CO_3	0	n/a
4	Cs_2CO_3	16	29
5	NaOAc	15	68
6	K_3PO_4	17	42
7 [b]	K_3PO_4	27	75
8	KOAc	28	82
**9**	**K_2HPO_4**	**27**	**82**
10	KH_2PO_4	0 [a]	n/a
11 [c]	K_2HPO_4	16	86
**12 [d]**	**K_2HPO_4**	**40**	**86**

Conditions: 0.10 mmol scale, reaction concentration of 0.10 M, carried out in sealed 12 mL tubes in a heating block at 85 °C for 24 h. [a]Acetal formation of starting material occurred; [b]AcOH (1.5 equiv) was added; [c]0.20 M; [d]0.05 M.

3.1.3 Optimization of Phosphine Ligand

The effect of different phosphine ligands was investigated, which identified that trialkylphosphines (such as PCy_3) were ineffective, while *para*-substituted triarylphosphines enabled cyclization. Triaylphosphines with para-electron-donating groups such as OMe led to reduced yield and enantioselectivity compared to PPh_3, while para-electron-withdrawing groups gave significant improvements; the cyclized product was formed in excellent enantioselectivity (up to 94% ee) using $P(4\text{-}Cl\text{-}C_6H_4)_3$ or $P(4\text{-}(CF_3)\text{-}C_6H_4)_3$.

Scheme S2. Optimization of phosphine ligand.

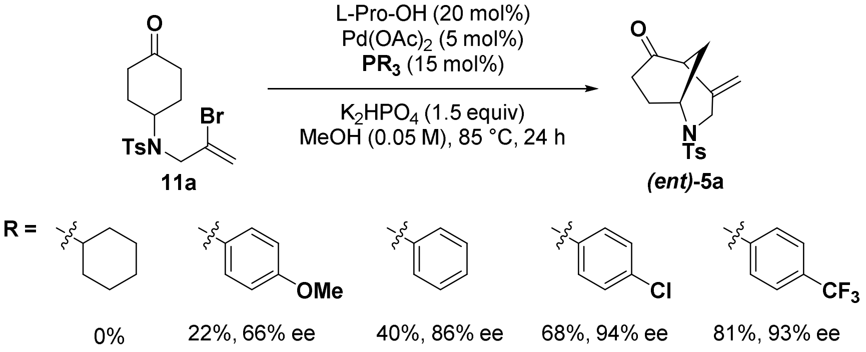

3.1.4 Optimization of Organocatalyst

Using P(4-Cl-C_6H_4)$_3$, the effect of different organocatalysts was investigated. In preliminary work, only cyclic secondary amine (pyrrolidinyl) catalysts afforded the cyclized product, while other catalyst classes, such as acyclic amino acids and cinchona alkaloid derivatives, were not effective. As a result, at this stage, further pyrrolidinyl-type catalysts were investigated. Using prolinol, prolinamide led to poor reactivity, while no reaction was observed using a proline-tetrazole catalyst. Using L-Hyp-OH (2*S*,4*R*)-4-hydroxyproline offered significant improvement compared to L-Pro-OH, with the cyclized product formed in excellent yield (>95%) and enantioselectivity (92%). Using (2*S*,4*S*)-4-hydroxyproline delivered the cyclized product in similar yield and enantioselectivity, indicating that the stereochemical configuration of the 4-hydroxyl group has no significant impact on yield or enantioselectivity. Importantly, the enantiomeric cyclized product could be obtained using the enantiomeric organocatalyst (2*R*,4*R*)-4-hydroxyproline. The silyl-protected 4-hydroxyproline organocatalyst also led to excellent enantioselectivity, albeit with reduced yields.

Scheme S3. Optimization of organocatalyst in the presence of phosphine P(4-Cl-C_6H_4)$_3$.

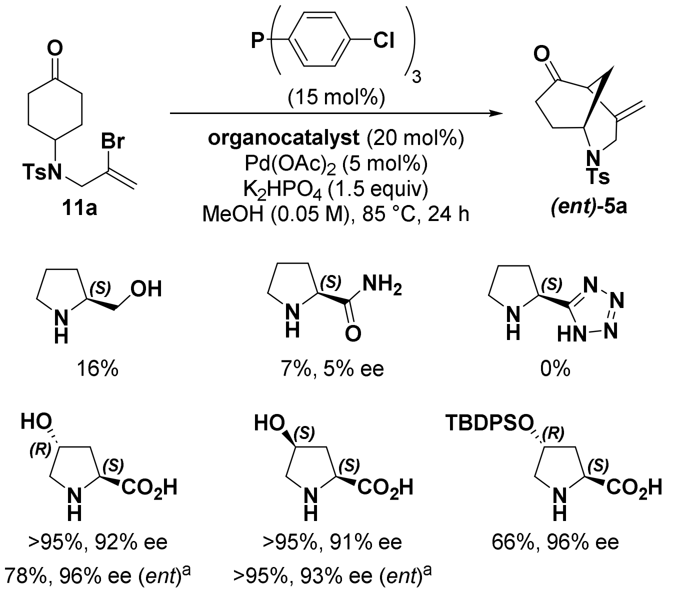

3.1.5 Optimal Conditions

By employing (*2S,4S*)-4-hydroxyproline or (*2R,4R*)-4-hydroxyproline in conjunction with phosphine P(4-(CF_3)-C_6H_4)$_3$, both enantiomers of the cyclized product were formed in excellent yield (95%) and enantioselectivity (94% *ee*).

Scheme S4. Optimal conditions employing 4-hydroxyproline organocatalysts in the presence of phosphine P(4-(CF_3)-C_6H_4)$_3$.

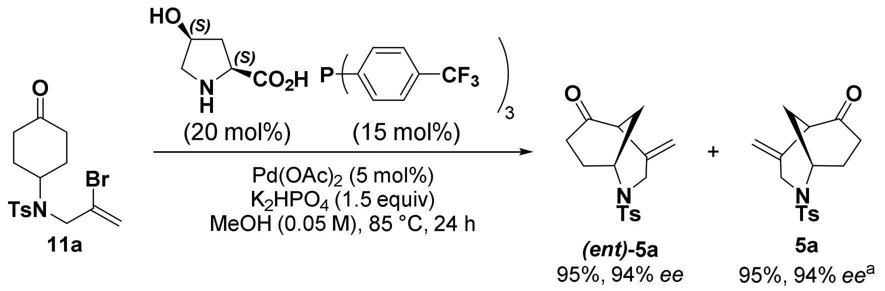

[a](*2R,4R*)-4-hydroxyproline used as an catalyst.

3.2 Synthesis of Starting Materials

3.2.1 Synthesis of Substrates 11a – 11g

2-bromoprop-2-en-1-amine (S1)

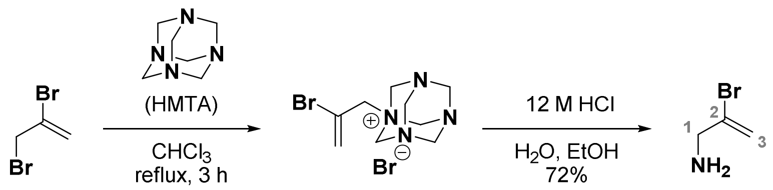

Compound **S1** was prepared according to a literature procedure.[11] 2,3-dibromoprop-1-ene (20.0 g, 90 wt %, 90.1 mmol, 0.91 equiv.) was added dropwise over 1 h using a syringe pump to a refluxed solution of urotropine (HMTA)(13.9 g, 99.0 mmol, 1 equiv.) in chloroform (170 mL). During the addition of bromide, the formation of a white precipitate was observed. The resulting suspension was refluxed for an additional 3 h and then allowed to cool down to room temperature. After standing for two days at room temperature and one day at 0 °C, the white crystalline ammonium salt was filtered off and dried on air (33.9 g). The obtained 2-bromoallylhexaminium bromide was dissolved in a mixture of water (57 mL), ethanol (200 mL) and 12 M HCl (68 mL) and the reaction mixture was left to stand at room temperature for 5 days. Then, the precipitated ammonium chloride was filtered off, washed with a small amount of ethanol and filtrated and concentrated under reduced pressure. The residue was dissolved in water (50 mL), and 3 M NaOH (100 mL) was added. The resulting mixture was extracted with Et_2O (4 x 75 mL), and the combined organic phases dried over anhydrous Na_2SO_4, filtered and concentrated under reduced pressure. The vacuum distillation (120 mbar, 70 °C) of crude product afforded 2-bromoprop-2-en-1-amine as a colorless oil in a mixture with Et_2O (10.4 g, 93 wt %, 72% yield). Data are in agreement with literature values.[12]

^{1}H NMR (400 MHz, $CDCl_3$) δ: 5.76 (q, *J* = 1.4 Hz, 1H, H-3a), 5.46 (dt, *J* = 1.8, 0.8 Hz, 1H, H-3b), 3.45 (d, *J* = 1.1 Hz, 2H, H-1), 1.46 (br s, 2H, NH_2).

^{13}C NMR (101 MHz, $CDCl_3$) δ: 136.9 (C-2), 115.4 (C-3), 51.2 (C-1).

***N*-(2-bromoallyl)-1,4-dioxaspiro[4.5]decan-8-amine (S2)**

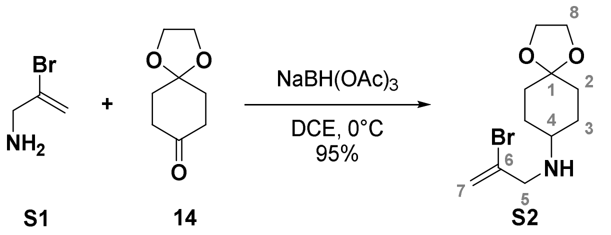

To a stirred solution of 2-bromoprop-2-en-1-amine (10.3 g, 93 wt%, 70.4 mmol, 1 equiv.) in DCE (195 mL) was added 1,4-dioxaspiro[4.5]decan-8-one (11.0 g, 70.4 mmol, 1.00 equiv.) and the resulting solution was cooled to 0 °C. Then, $NaBH(OAc)_3$ (20.9 g, 98.6 mmol, 1.40 equiv.) was added slowly, the ice bath was removed, and the resulting solution was stirred at room temperature for 2 days. The reaction was quenched with 1 M aq. KOH (200 mL) and extracted with CH_2Cl_2 (3 x 150 mL). The combined organic phases were dried over anhydrous Na_2SO_4, filtered and concentrated under reduced pressure. The product **S2** was obtained as a yellow oil (18.5 g, 95%) and used in the next step without further purification.

IR (film) $\tilde{\nu}_{max}$/cm^{-1}: 2935, 2880, 1626, 1446, 1377, 1279, 1240, 1178, 1104, 916, 895, 740, 661.

^{1}H NMR (400 MHz, $CDCl_3$) δ: 5.78 (q, *J* = 1.3 Hz, 1H, H-7a), 5.53 (dd, *J* = 1.7, 0.9 Hz, 1H, H-7b), 3.92 (s, 4H, H-8), 3.46 (t, *J* = 1.0 Hz, 2H, H-5), 2.56 (tt, *J* = 9.4, 3.4 Hz, 1H, H-4), 1.91 – 1.71 (m, 4H, H-3, H-2), 1.70 – 1.37 (m, 4H, H-3, H-2).

^{13}C NMR (101 MHz, $CDCl_3$) δ: 134.2 (C-6), 117.3 (C-7), 108.8 (C-1), 64.41 (C-8), 64.37 (C-8), 55.0 (C-5), 52.9 (C-4), 32.8 (2xC-3 or 2xC-2), 30.1 (2xC-3 or 2xC-2).

HRMS (ES+): Exact mass calculated for $[M+H]^+$ $(C_{11}H_{19}O_2N^{79}Br)^+$ requires 276.0594, found 232.0595.

4-((2-bromoallyl)amino)cyclohexan-1-one (S3)

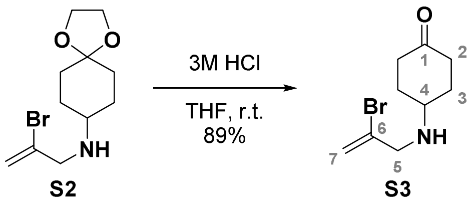

To a solution of acetal **S2** (18.5 g, 67.0 mmol, 1 equiv.) in THF (185 mL) was added 3 M aq. HCl (185 mL) and the resulting solution was stirred for 2 days. Then, the reaction mixture was basified by adding 3 M aq. KOH (300 mL) and extracted with ethyl acetate (3 x 200 mL). The combined organic phases were washed with brine (2 x 100 mL), dried over anhydrous Na_2SO_4, filtered and concentrated under reduced pressure. Purification by column chromatography (ethyl acetate:pentane = 2:1) afforded ketone **S3** as a yellowish oil (13.9 g, 89%).

Note: The crude product can be used in the next step without further purification. However, when used in a tosylation reaction leading to product **11a**, it is necessary to remove all ethylene glycol formed during the acetal hydrolysis. The ethylene glycol ditosylate side product is not separable by FCC and acts as a catalyst poison in the cyclization step.

IR (film) $\tilde{\nu}_{max}$/cm^{-1}: 2933, 2864, 1712, 1629, 1446, 1424, 1329, 1233, 1172, 1118, 896, 842, 745, 678.

^{1}H NMR (400 MHz, CDCl$_3$) δ: 5.81 (dd, *J* = 2.0, 1.1 Hz, 1H, H-7a), 5.59 (d, *J* = 1.8 Hz, 1H, H-7b), 3.53 – 3.49 (m, 2H, H-5), 3.04 – 2.93 (m, 1H, H-4), 2.51 (dtd, *J* = 15.0, 5.8, 1.3 Hz, 2H, H-2a), 2.37 – 2.23 (m, 2H, H-2b), 2.10 – 1.97 (m, 2H, H-3a), 1.72 (dddd, *J* = 13.3, 9.6, 8.0, 5.2 Hz, 2H, H-3b).

^{13}C NMR (101 MHz, CDCl$_3$) δ: 211.2 (C-1), 133.8 (C-6), 117.9 (C-7), 54.8 (C-5), 50.8 (C-4), 38.2 (2xC-2), 31.7 (2xC-3).

HRMS (ES+): Exact mass calculated for $[M+H]^+$ $(C_9H_{15}ON^{79}Br)^+$ requires 232.0332, found 232.0334.

3.2.2 General Procedure A – Sulfonylation of Secondary Amines

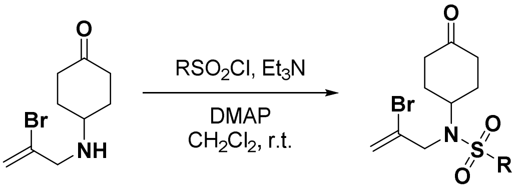

To a solution of amine (1.00 equiv) in CH_2Cl_2 (0.20 m) at 0 °C was dropwise added Et_3N (1.75 equiv). After the addition of Et_3N was complete, DMAP (0.20 equiv) was added, followed by sulfonyl chloride (1.75 equiv). The reaction mixture was stirred at RT until complete, then carefully acidified with aqueous 1 M HCl. The phases were separated then the aqueous layer was extracted with CH_2Cl_2. The combined organic extracts were dried over Na_2SO_4, filtered, and concentrated under a vacuum. The crude residue was purified by flash column chromatography.

***N*-(2-bromoallyl)-4-methyl-*N*-(4-oxocyclohexyl)benzenesulfonamide (11a)**

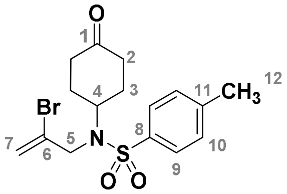

11a

Prepared according to General Procedure A using amine **S3** (13.8 g, 59.5 mmol, 1 equiv.) in CH_2Cl_2 (158 mL), Et_3N (14.5 mL, 104.0 mmol, 1.75 equiv.), DMAP (1.45 g, 11.9 mmol, 0.20 equiv.) and a solution of *p*-toluenesulfonyl chloride (19.8 g, 104.0 mmol, 1.75 equiv.) in CH_2Cl_2 (30 mL). Purification by gradient column chromatography (ethyl acetate:pentane = 1:4 to 1:1) to ketone **11a** as a white solid (20.5 g, 89%).

Note: If needed, the purity of the obtained material can be increased by recrystallization from boiling cyclohexane/ethyl acetate.

MP: 113–116 °C.

IR (film) $\tilde{\nu}_{max}/cm^{-1}$: 2980, 1715, 1642, 1341, 1156, 1042.

^{1}H NMR (400 MHz, $CDCl_3$) δ: 7.79 – 7.71 (m, 2H, H-9), 7.32 (d, *J* = 8.2 Hz, 2H, H-10), 6.00 (q, *J* = 1.8 Hz, 1H, H-7a), 5.64 (dt, *J* = 2.5, 1.3 Hz, 1H, H-7b), 4.20 (tt, *J* = 11.8, 4.0 Hz, 1H, H-4), 4.01 (t, *J* = 1.5 Hz, 2H, H-5), 2.44 (s, 3H, H-12), 2.41 – 2.33 (m, 4H, H-2), 1.97 – 1.75 (m, 4H, H-3).

^{13}C NMR (101 MHz, $CDCl_3$) δ: 208.4 (C-1), 144.0 (C-11), 137.7 (C-8), 130.1 (2xC-10), 130.0 (C-6), 127.2 (2xC-9), 118.9 (C-7), 56.3 (C-4), 51.5 (C-5), 40.1 (2xC-2), 30.0 (2xC-3), 21.7 (C-12).

HRMS (ES+): Exact mass calculated for $[M+Na]^+$ $(C_{16}H_{20}O_3N^{79}Br^{23}Na^{32}S)^+$ requires 408.0240, found 408.0239.

***N*-(2-bromoallyl)-4-methoxy-*N*-(4-oxocyclohexyl)benzenesulfonamide (11b)**

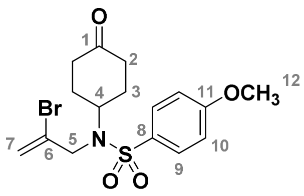

11b

Prepared according to General Procedure A using amine **S3** (500 mg, 2.15 mmol, 1 equiv.) in CH_2Cl_2 (10.8 mL), Et_3N (1.13 mL, 8.08 mmol, 3.75 equiv.), DMAP (52.6 mg, 0.431 mmol, 0.20 equiv.), and a solution of 4-methoxybenzenesulfonyl chloride (1.22 g, 5.92 mmol, 2.75 equiv.) in CH_2Cl_2 (1 mL). Purification by gradient column chromatography (ethyl acetate:pentane = 1:4 to 3:7) afforded **11b** (690 mg, 80%) as a white solid.

MP: 106–108 °C.

IR (film) $\tilde{\nu}_{max}/cm^{-1}$: 2945, 1709, 1595, 1497, 1341, 1304, 1259, 1151, 1094, 1032.

^{1}H NMR (400 MHz, $CDCl_3$) δ: 7.79 (d, *J* = 9.0 Hz, 2H, H-9), 6.98 (d, *J* = 9.0 Hz, 2H, H-10), 5.99 (s, 1H, H-7a), 5.63 (s, 1H, H-7b), 4.18 (tt, *J* = 11.5, 4.0 Hz, 1H, H-4), 4.00 (s, 2H, H-5), 3.88 (s, 3H, H-12), 2.45 – 2.30 (m, 4H, H-2), 1.97 – 1.75 (m, 4H, H-3).

^{13}C NMR (101 MHz, $CDCl_3$) δ: 208.5 (C-1), 163.2 (C-11), 132.2 (C-8), 130.0 (C-6), 129.4 (2xC-9), 118.8 (C-7), 114.6 (2xC-10), 56.2 (C-4), 55.8 (C-12), 51.4 (C-5), 40.1 (2xC-2), 30.0 (2xC-3).

HRMS (ES+): Exact mass calculated for $[M+H]^+$ $(C_{16}H_{21}O_4N^{79}Br^{32}S)^+$ requires 402.0370, found 402.0370.

***N*-(2-bromoallyl)-*N*-(4-oxocyclohexyl)methanesulfonamide (11c)**

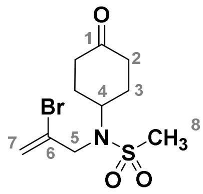

11c

Prepared according to General Procedure A using amine **S3** (500 mg, 2.15 mmol, 1 equiv.) in CH_2Cl_2 (10.8 mL), Et_3N (0.53 mL, 3.77 mmol, 1.75 equiv.), DMAP (52.6 mg, 0.43 mmol, 0.2 equiv.), and methanesulfonyl chloride (0.29 mL, 3.77 mmol, 1.75 equiv.). Purification by gradient column chromatography (ethyl acetate:pentane = 3:7 to 2:3) afforded **11c** (446 mg, 67% yield) as a white solid.

MP: 68–70 °C.

IR (film) $\tilde{\nu}_{max}$/cm^{-1}: 2981, 1710, 1328, 1142, 1105, 1037.

^{1}H NMR (400 MHz, $CDCl_3$) δ: 6.01 (dt, *J* = 2.0, 1.5 Hz, 1H, H-7a), 5.70 (dt, *J* = 2.0, 1.5 Hz, 1H, H-7b), 4.20 (tt, *J* = 12.0, 4.0 Hz, 1H, H-4), 4.07 (t, *J* = 1.5 Hz, 2H, H-5), 3.01 (s, 3H, H-10), 2.51 – 2.43 (m, 4H, H-2), 2.26 – 2.17 (m, 2H, H-3), 2.12 – 1.97 (m, 2H, H-3).

^{13}C NMR (101 MHz, $CDCl_3$) δ: 208.3 (C-1), 129.7 (C-6), 120.3 (C-7), 56.6 (C-4), 52.0 (C-5), 41.9 (C-10), 40.0 (2xC-2), 30.6 (2xC-3).

HRMS (ES+): Exact mass calculated for $[M+Na]^+$ $(C_{10}H_{16}O_3N^{79}Br^{23}Na^{32}S)^+$ requires 331.9926, found 331.9927.

***N*-(2-bromoallyl)-*N*-(4-oxocyclohexyl)acetamide (11d)**

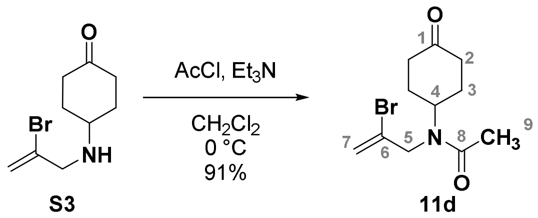

To a solution of amine **S3** (500 mg, 2.15 mmol, 1 equiv.) in CH_2Cl_2 (10.8 mL) at 0 °C was added Et_3N (0.38 mL, 2.69 mmol, 1.25 equiv.) dropwise, followed by acetyl chloride (0.19 mL, 2.69 mmol, 1.25 equiv.). The resulting mixture was stirred at 0 °C for 2 h until complete and then acidified with aqueous 1 M HCl. The layers were separated then the aqueous layer was extracted with CH_2Cl_2 (3 x 20 mL). The combined organic layers were dried over Na_2SO_4, then filtered and concentrated. Purification by gradient column chromatography (ethyl acetate:pentane = 1:1 to 7:3) afforded 11d (538 mg, 91% yield) as a pale orange solid.

Note: Compound **11d** is a 6:1 mixture of rotamers in $CDCl_3$ at room temperature (* minor rotamer).

MP: 72–74 °C.

IR (film) $\tilde{\nu}_{max}$/cm^{-1}: 2967, 2873, 1740, 1631, 1412, 1225, 1059.

^{1}H NMR (400 MHz, $CDCl_3$) δ: 5.78 (q, J = 1.9 Hz, 1H, H-7a), 5.64 (q, J = 1.8 Hz, 1H, H-7b), 5.61 (q, J = 1.9 Hz, 1H, H-7a*), 5.52 – 5.46 (m, 1H, H-7b*), 4.82 (tt, J = 12.1, 3.8 Hz, 1H, H-4), 4.11 (br s, 2H, H-5*), 3.99 (t, J = 1.8 Hz, 2H, H-5), 2.52 – 2.32 (m, 4H, H-2), 2.24 (s, 3H, H-9*), 2.09 (s, 3H, H-9), 2.01 (dtd, J = 11.6, 6.4, 2.8 Hz, 2H, H-3), 1.81 (qd, J = 12.8, 5.1 Hz, 2H, H-3).

^{13}C NMR (101 MHz, $CDCl_3$) δ: 209.3 (C-1), 208.0 (C-1*), 171.4 (C-8), 170.2 (C-8*), 129.7 (C-6), 129.4 (C-6*), 117.2 (C-7), 116.4 (C-7*), 56.2 (C-5*), 52.4 (C-5), 51.7 (C-4), 48.7 (C-4*), 39.9 (2xC-2), 39.8 (2xC-2*), 30.3(2xC-3*), 29.2 (2xC-3), 22.1 (C-9), 22.0 (C-9*).

HRMS (ES+): Exact mass calculated for $[M+H]^+$ $(C_{11}H_{17}O_2N^{79}Br)^+$ requires 274.0437, found 274.0439.

***tert*-Butyl (2-bromoallyl)(4-oxocyclohexyl)carbamate (11e)**

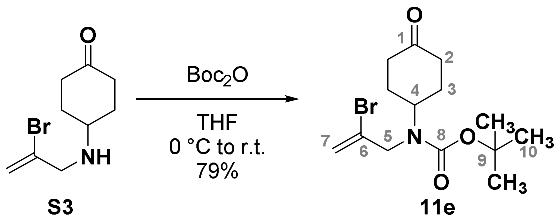

To a solution of amine **S3** (2.00 g, 8.62 mmol, 1 equiv.) in dry THF (8.6 mL) at 0 °C was added di-*tert*-butyldicarbonate (2.03 g, 9.31 mmol, 1.08 equiv.) portionwise. The mixture was allowed to warm to r.t. overnight and, upon completion, was quenched with H_2O and diluted with CH_2Cl_2. The phases were separated, and the aqueous layer was extracted with CH_2Cl_2 (2 x 50 mL). The combined organic layers were dried over Na_2SO_4, filtered and concentrated. Purification by gradient column chromatography (ethyl acetate:pentane = 1:9 to 1:3) afforded **11e** (2.73 g, 96% yield) as a white solid.

MP: 76–78 °C

IR (film) $\tilde{\nu}_{max}$/cm^{-1}: 2975, 2938, 1716, 1680, 1365, 1236.

^{1}H NMR (400 MHz, $CDCl_3$) δ: 5.70 (br s, 1H, H-7a), 5.53 (br s, 1H, H-7b), 4.43 (br s, 1H, H-4), 3.93 (br s, 2H, H-5), 2.50 – 2.35 (m, 4H, H-2), 2.12 – 2.02 (m, 2H, H-3), 1.87 (br s, 2H, H-3), 1.44 (s, 9H, H-10).

^{13}C NMR (101 MHz, $CDCl_3$) δ: 209.5 (C-1), 155.0 (C-8), 131.0 (C-6), 116.0 (C-7), 80.8 (C-9), 53.4 (C-4), 51.1 (C-5), 40.1 (2xC-2), 29.8 (2xC-3), 28.4 (C-10).

HRMS (ES+): Exact mass calculated for $[M+Na]^+$ $(C_{14}H_{22}O_3N^{79}Br^{23}Na)^+$ requires 354.0675, found 354.0676.

benzyl (2-bromoallyl)(4-oxocyclohexyl)carbamate (11f)

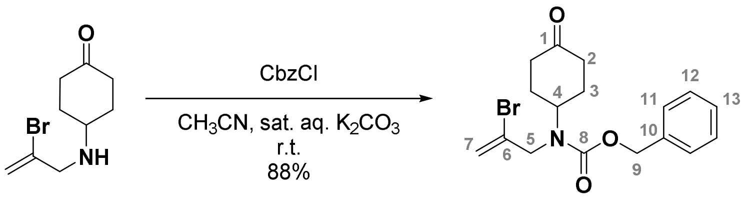

S3 11f

To a solution of amine S**3** (500 mg, 2.15 mmol, 1 equiv.) in acetonitrile (21.5 mL) was added a saturated aqueous solution of K_2CO_3 (21.5 mL) followed by benzyl chloroformate (0.62 mL, 4.31 mmol, 2.00 equiv.) dropwise. The resulting solution was stirred at room temperature overnight and, upon completion, diluted with EtOAc. The aqueous phase was extracted with EtOAc (3 x 25 mL) then the combined organic layers were dried over Na_2SO_4, filtered and concentrated. Purification by column chromatography (ethyl acetate:pentane = 1:4) afforded **11f** (695 mg, 88% yield) as a pale yellow oil.

IR (film) $\tilde{\nu}_{max}$/cm^{-1}: 2955, 1695, 1410, 1220, 1125.

^{1}H NMR (400 MHz, $CDCl_3$) δ: 7.42 – 7.29 (m, 5H, Ar), 5.69 (br s, 1H, H-7a), 5.55 (br s, 1H, H-7b), 5.17 (br s, 2H, H-9), 4.43 (br s, 1H, H-4), 4.06 (br s, 2H, H-5), 2.54 – 2.36 (m, 4H, H-2), 2.16 – 2.07 (m, 2H, H-3), 2.03 – 1.88 (m, 2H, H-3).

^{13}C NMR (101 MHz, $CDCl_3$) δ: 209.3 (C-1), 155.7 (C-8), 136.3 (C-6), 130.2 (C-10), 128.6 (2xC-Ar), 128.3 (C-13), 128.1 (2xC-Ar), 116.7 (C-7), 67.7 (C-9), 54.5 (C-4), 51.2 (C-5), 39.9 (2xC-2), 29.5 (2xC-3).

HRMS (ES+): Exact mass calculated for $[M+H]^+$ $(C_{17}H_{21}O_3N^{79}Br)^+$ requires 366.0699, found 366.0701.

4-(benzyl(2-bromoallyl)amino)cyclohexan-1-one (11g)

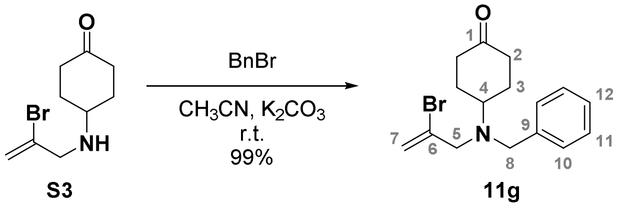

To a solution of amine **S3** (928 mg, 4.00 mmol, 1 equiv.) in dry CH_3CN (4.0 mL) was added K_2CO_3 (829 mg, 6.00 mmol, 1.5 equiv.) followed by benzyl bromide (0.71 mL, 6.00 mmol, 1.5 equiv.). The heterogeneous mixture was stirred at room temperature for 4 d. Upon completion, the reaction mixture was filtered through Celite, eluting with EtOAc, and then the filtrate was concentrated under a vacuum. Purification by column chromatography (ethyl acetate:pentane = 1:19) afforded **11g** (1.28 g, 99%) as a white solid.

MP: 40–42 °C.

IR (film) $\tilde{\nu}_{max}$/cm^{-1}: 2933, 2797, 1715, 1626, 1493, 1447, 1325, 1262, 905, 730.

^{1}H NMR (400 MHz, $CDCl_3$) δ: 7.43 – 7.37 (m, 2H, H-10), 7.36 – 7.30 (m, 2H, H-11), 7.29 – 7.23 (m, 1H, H-12), 5.96 (q, J = 1.5 Hz, 1H, H-7a), 5.57 (q, J = 1.5 Hz, 1H, H-7b), 3.71 (s, 2H, H-5), 3.34 (t, J = 1.0 Hz, 2H, H-8), 3.06 (tt, J = 11.5, 3.5 Hz, 1H, H-4), 2.46 – 2.38 (m, 2H, H-2), 2.36 – 2.25 (m, 2H, H-2), 2.19 – 2.10 (m, 2H, H-3), 1.82 – 1.69 (m, 2H, H-3).

^{13}C NMR (101 MHz, $CDCl_3$) δ: 210.0 (C-1), 139.6 (C-9), 133.2 (C-6), 128.5 (2xC-10, 2xC-11), 127.2 (C-12), 118.0 (C-7), 58.4 (C-8), 56.2 (C-4), 54.2 (C-5), 40.1 (2xC-2), 27.7 (2xC-3).

HRMS (ES+) Exact mass calculated for $[M+H]^+$ $(C_{16}H_{21}ON^{79}Br)^+$ requires 322.0801, found 322.0802.

3.2.3 Synthesis of Substrates 11h and 11i

Scheme S5. Preparation of substrates **11h** and **11i.**

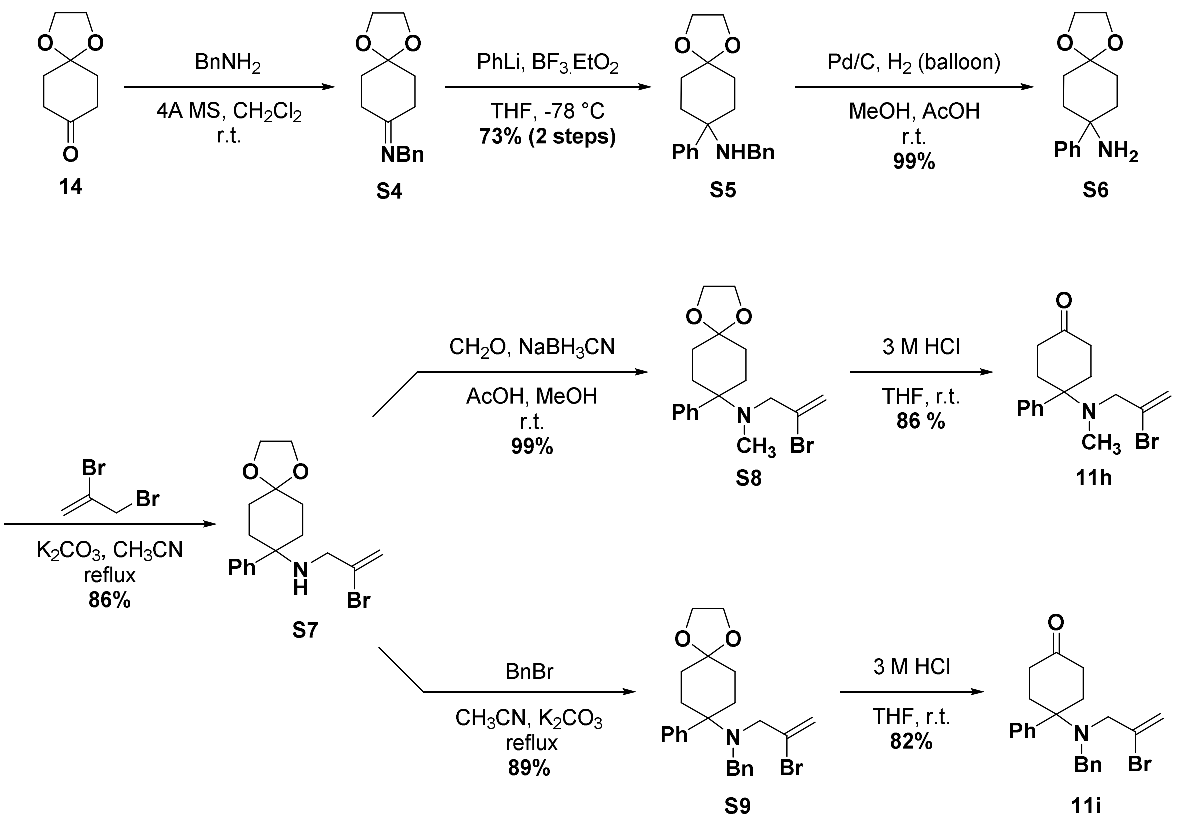

N-benzyl-8-phenyl-1,4-dioxaspiro[4.5]decan-8-amine (S5)

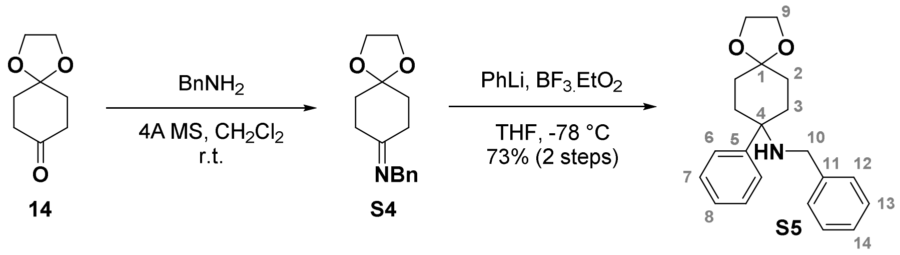

Prepared according to a literature procedure.[13] To a solution of 1,4-cyclohexanedione monoethylene acetal (4.00 g, 25.6 mmol, 1 equiv.) in CH_2Cl_2 (80.0 mL) was added benzylamine (3.36 mL, 30.7 mmol, 1.20 equiv.) and 4 A molecular sieves (12.8 g). The mixture was stirred at room temperature overnight until complete, then filtered through Celite and concentrated under a vacuum. The crude imine **S4** was sufficiently pure to be used without further purification.

Prepared according to a literature procedure.[14] Boron trifluoride diethyl ether complex (5.0 mL, 40.8 mmol, 2.00 equiv.) was added dropwise to a solution of imine **S4** (5.00 g, 20.4 mmol, 1 equiv.), in dry THF (100 mL) at –78 °C then the mixture was stirred at that temperature for 45 mins. A solution of phenyl lithium (21.5 mL, 1.9 M in Bu_2O, 40.8 mmol, 2.00 equiv.) was then added dropwise to the imine solution, then the reaction mixture was stirred at –78 °C for a further 45 mins. The reaction was then warmed to RT and stirred for 2 – 3 h until complete. The reaction was quenched with a 20% aqueous NaOH solution and diluted with Et_2O, the phases were separated, and the aqueous layer was extracted with Et_2O (3 x 100 mL). The combined organic layers were dried over Na_2SO_4, filtered and concentrated. Purification by gradient column chromatography (ethyl acetate:pentane = 1:19 to 1:4) afforded amine **S5** (4.83 g, 73% yield) as a white solid. The spectroscopic data match those reported in the literature.[14]

^{1}H NMR (400 MHz, $CDCl_3$) δ: 7.53 – 7.46 (m, 2H, H-6), 7.41 – 7.33 (m, 1H, H-7), 7.33 – 7.17 (m, 6H, H-8, H-12, H-13, H-14), 4.02 – 3.91 (m, J = 3.6 Hz, 4H, H-9), 3.35 (s, 2H, H-10), 2.19 – 2.05 (m, 4H, H-2, H-3), 2.05 – 1.94 (m, 2H, H-2), 1.70 – 1.61 (m, 2H, H-3), 1.49 (s, 1H, NH).

^{13}C NMR (101 MHz, $CDCl_3$) δ: 146.7 (C-5), 141.3 (C-11), 128.4 (2xC-12, 2xC-13), 128.4 (2xC-7), 126.9 (C-14), 126.5 (C-8), 126.1 (2xC-6), 108.9 (C-1), 64.4 (C-9), 64.3 (C-9), 57.0 (C-4), 46.9 (C-10), 33.7 (2xC-3), 30.9 (2xC-2).

8-phenyl-1,4-dioxaspiro[4.5]decan-8-amine (S6)

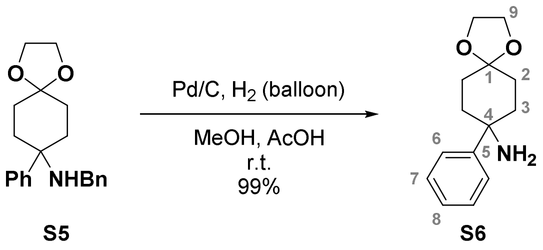

Prepared according to a literature procedure.[14] To a solution of amine **S5** (2.36 g, 7.30 mmol, 1 equiv.) in a mixture of MeOH (63 mL) and AcOH (3.2 mL) under N_2 was added palladium on carbon (777 mg, 0.73 mmol, 10 wt. % loading, 0.10 equiv.) portionwise. H_2 was bubbled through the solution for 2 mins, then the mixture was stirred under a H_2 atmosphere (balloon, two skins) for 24 h. Upon completion, the reaction mixture was filtered through Celite and washed with EtOAc. The filtrate was then washed with aqueous 3 M NaOH, then the phases were separated, and the aqueous phase was extracted with EtOAc (3 x 100 mL). The combined organic layers were dried over Na_2SO_4, filtered and concentrated to afford amine **S6** (1.69 g, 99% yield) as a pale-yellow oil which was sufficiently pure to be used without further purification. The spectroscopic data match those reported in the literature.[14]

^{1}H NMR (400 MHz, CDCl$_3$) δ: 7.56 – 7.49 (m, 2H, H-6), 7.34 (dd, *J* = 8.4, 7.0 Hz, 2H, H-7), 7.25 – 7.19 (m, 1H, H-8), 4.02 – 3.91 (m, 4H, H-9), 2.22 (ddd, *J* = 14.3, 11.4, 3.9 Hz, 2H, H-3), 2.02 – 1.91 (m, 2H, H-2), 1.80 – 1.71 (m, 2H, H-3), 1.70 – 1.61 (m, 2H, H-2), 1.48 (br s, 2H, NH_2).

^{13}C NMR (101 MHz, CDCl$_3$) δ: 149.3 (C-5), 128.5 (2xC-7), 126.6 (C-8), 125.1 (2xC-6), 108.7 (C-1), 64.4 (C-9), 64.3 (C-9), 53.4 (C-4), 36.9 (2xC-3), 31.4 (2xC-2).

N-(2-bromoallyl)-8-phenyl-1,4-dioxaspiro[4.5]decan-8-amine (S7)

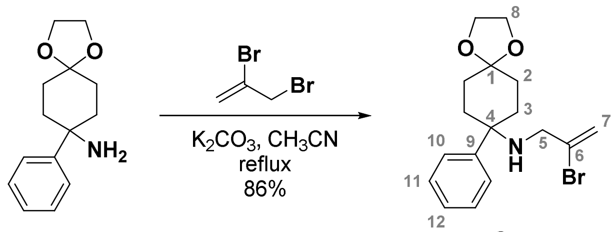

To a solution of amine **S6** (1.60 g, 6.86 mmol, 1 equiv.) in MeCN (34.3 mL) was added K_2CO_3 (1.90 g, 13.7 mmol, 2 equiv.) followed by 2,3-dibromopropene (3.05 g, 13.7 mmol, 90%, 2.00 equiv.) dropwise. The mixture was heated to reflux and stirred overnight. On completion, the reaction was cooled to room temperature, filtered through Celite, washed with EtOAc, and the filtrate was concentrated. Purification by column chromatography (ethyl acetate:pentane = 1:9) afforded **S7** (2.07 g, 86% yield) as a pale orange solid.

MP: 40–42 °C.

IR (film) $\tilde{\nu}_{max}$/cm^{-1}: 2934, 1606, 1510, 1335, 1249, 1154, 1091, 1032.

^{1}H NMR (400 MHz, $CDCl_3$) δ: 7.46 – 7.40 (m, 2H, H-10), 7.37 – 7.30 (m, 2H, H-11), 7.25 – 7.20 (m, 1H, H-12), 5.85 (q, J = 1.5 Hz, 1H, H-7a), 5.45 (d, J = 1.5 Hz, 1H, H-7b), 4.00 – 3.91 (m, 4H, H-8), 3.04 (s, 2H, H-5), 2.16 – 1.89 (m, 6H, H-2, H-3), 1.68 – 1.60 (m, 2H, H-3).

^{13}C NMR (101 MHz, $CDCl_3$) δ: 145.9 (C-9), 133.5 (C-6), 128.4 (2xC-11), 126.7 (C-12), 126.0 (2xC-10), 116.6 (C-7), 108.7 (C-1), 66.4 (C-8), 64.3 (C-8), 56.7 (C-4), 51.3 (C-5), 33.7 (2xC-2), 30.9 (2xC-3).

HRMS (ES+): Exact mass calculated for $[M+H]^+$ $(C_{17}H_{23}O_2N^{79}Br)^+$ requires 352.0907, found 352.0907.

N-(2-bromoallyl)-N-methyl-8-phenyl-1,4-dioxaspiro[4.5]decan-8-amine (S8)

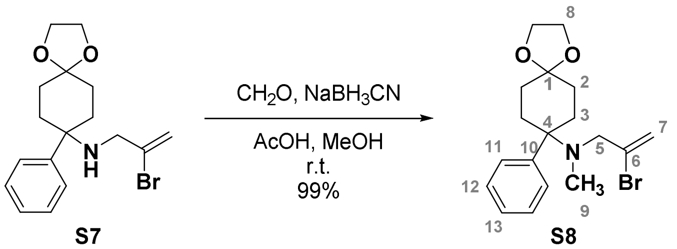

Prepared following a literature procedure.[15] To a solution of amine **S7** (1.00 mg, 2.84 mmol, 1 equiv.) in MeOH (18.9 mL) was added formaldehyde (1.7 mL, 22.7 mmol, 37% aq. solution, 8.00 equiv.) dropwise, AcOH (1.3 mL, 22.7 mmol, 8.00 equiv.) dropwise, and finally $NaBH_3CN$ (357 mg, 5.68 mmol, 2.00 equiv.) portionwise. The mixture was stirred at r.t. for 2 hours until complete before quenching with saturated aqueous K_2CO_3 solution. The layers were separated, and the aqueous layer was extracted with CH_2Cl_2 (3 x 50 mL), then the combined organic layers were dried over Na_2SO_4, filtered and concentrated. Purification by column chromatography (ethyl acetate:pentane = 1:9) afforded ketal of **S8** (1.04 g, 99% yield) as a white solid.

MP: 92-94 °C.

IR (film) $\tilde{\nu}_{max}$/cm^{-1}: 2958, 2927, 2878, 2361, 2241, 1627, 1445, 1377, 1101, 1039, 1011, 944, 892, 765, 701.

^{1}H NMR (400 MHz, $CDCl_3$) δ: 7.40 – 7.31 (m, 4H, H-9, H11, H12), 7.31 – 7.20 (m, 1H, H-13), 5.89 (q, J = 1.5 Hz, 1H, H-7a), 5.50 (q, J = 1.5 Hz, 1H, H-7b), 4.01 – 3.88 (m, 4H, H-8), 3.04 (s, 2H, H-5), 2.40 – 2.30 (m, 2H, H-2), 2.10 (s, 3H, H-9), 2.08 – 1.95 (m, 4H, H-2, H-3), 1.62 – 1.52 (m, 2H, H-3).

^{13}C NMR (101 MHz, $CDCl_3$) δ: 140.5 (C-10), 133.9 (C-6), 128.0 (2xC-Ar), 126.82 (2xC-Ar), 126.78 (C-13), 117.1 (C-7), 109.0 (C-1), 64.5 (C-8), 64.3 (C-8), 60.1 (C-4), 59.4 (C-5), 34.6 (C-9), 31.3 (2xC-2), 38 31.2 (2xC-3).

HRMS (ES+): Exact mass calculated for $[M+H]^+$ $(C_{18}H_{25}O_2N^{79}Br)^+$ requires 366.1063, found 366.1062.

4-((2-bromoallyl)(methyl)amino)-4-phenylcyclohexan-1-one (11h)

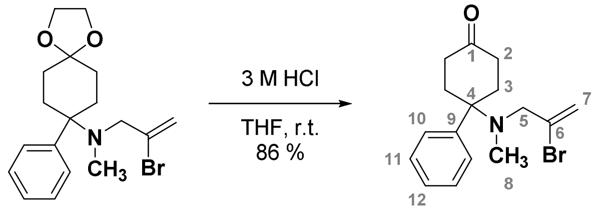

S8 11h

The ketal **S8** (800 mg, 2.18 mmol, 1 equiv.) was dissolved in a 1:1 mixture of THF (10.9 mL) and aqueous 3 M HCl (10.9 mL) and stirred at r.t. until complete (48 h, monitored by TLC). The solution was then basified with aqueous 3 M NaOH to pH 12 and extracted with EtOAc (3 x 30 mL). The combined organic layers were washed with brine (30 mL), then dried over Na_2SO_4 and concentrated under a vacuum. Purification by column chromatography (ethyl acetate:pentane = 1:19) afforded **11h** (608 mg, 86% yield) as a white solid.

MP: 85–87 °C.

IR (film) $\tilde{\nu}_{max}$/cm^{-1}: 2959, 2904, 2879, 2846, 2816, 2788, 1713.

^{1}H NMR (400 MHz, $CDCl_3$) δ: 7.43 – 7.35 (m, 4H, H-10, H11), 7.34 (m, 1H, H-12), 5.81 (m, 1H, H-7a), 5.53 (m, 1H, H-7b), 3.12 (s, 2H, H-5), 2.85 (ddd, *J* = 16.0, 11.5, 5.5 Hz, 2H, H-2), 2.77 – 2.68 (m, 2H, H-3), 2.35 – 2.26 (m, 2H, H-2), 2.18 – 2.08 (m, 2H, H-3), 2.14 (s, 3H, H-8).

^{13}C NMR (101 MHz, $CDCl_3$) δ: 211.7 (C-1), 139.3 (C-9), 133.4 (C-6), 128.4 (2xC-Ar), 127.4 (C-12), 126.6 (2xC-Ar), 118.0 (C-7), 59.6 (C-4), 59.4 (C-5), 37.3 (C-2), 34.3 (C-8), 33.5 (C-3).

HRMS (ES+): Exact mass calculated for $[M+H]^+$ $(C_{16}H_{21}ON^{79}Br)^+$ requires 322.0801, found 322.0803.

N-benzyl-N-(2-bromoallyl)-8-phenyl-1,4-dioxaspiro[4.5]decan-8-amine (S9)

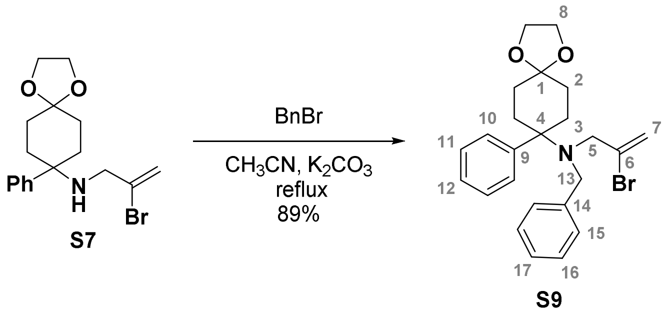

To a stirring solution of amine **S7** (300 mg, 0.852 mmol, 1 equiv.) in MeCN (4.3 mL) was added K_2CO_3 (1.18 g, 8.52 mmol, 10.00 equiv.) and dropwise benzyl bromide (2.0 mL, 17.0 mmol, 20 equiv.). The mixture was heated to reflux and stirred overnight, then allowed to cool to room temperature, filtered through celite, washed with EtOAc, and evaporated. The residue was purified by gradient column chromatography (ethyl acetate:pentane = 1:19 to 1:9) to afford the acetal of **S9** (334 mg, 89% yield) as a colorless oil.

IR (film) ṽmax/cm^{-1}: 2957, 2880, 1601, 1494, 1447, 1375, 1272, 1096, 1034, 940, 765, 699.

^{1}H NMR (400 MHz, $CDCl_3$) δ: 7.54 – 7.45 (m, 2H, H-10), 7.41 (t, *J* = 7.7 Hz, 2H, H-11), 7.35 – 7.13 (m, 6H, H-Ar), 5.79 – 5.74 (m, 1H, H-7a), 5.29 – 5.24 (m, 1H, H-7b), 4.02 – 3.92 (m, 2H, H-8a), 3.92 – 3.85 (m, 2H, H-8b), 3.55 (s, 2H, H-13), 3.29 (s, 2H, H-5), 2.41 – 2.30 (m, 2H, H-3), 2.15 (td, *J* = 13.1, 3.8 Hz, 2H, H-3), 1.82 – 1.72 (m, 2H. H-2), 1.50 (td, *J* = 13.0, 3.7 Hz, 2H, H-2).

^{13}C NMR (101 MHz, $CDCl_3$) δ: 141.2 (C-9), 140.4 (C-14), 134.6 (C-6), 128.9 (2x-C-Ar), 128.3 (2xC-Ar), 128.0 (2xC-Ar), 127.7 (2xC-Ar), 127.0 (C-Ar), 126.7 (C-Ar), 117.3 (C-7), 108.7 (C-1), 64.4 (C-8), 64.3 (C-8), 63.1 (C-4), 58.8 (C-5), 54.8 (C-13), 31.8 (C-2), 31.3 (C-3).

HRMS (ES+): Exact mass calculated for $[M+H]^+$ $(C_{24}H_{29}O_2N^{79}Br)^+$ requires 442.1376, found 442.1369.

4-(benzyl(2-bromoallyl)amino)-4-phenylcyclohexan-1-one (11i)

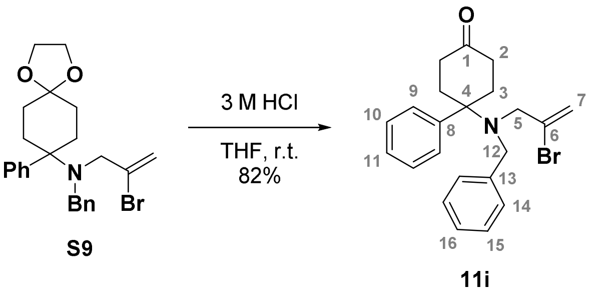

The ketal **S9** (900 mg, 2.03 mmol, 1 equiv.) was dissolved in a 1:1 mixture of THF (10.2 mL) and aqueous 3 M HCl (10.2 mL) and stirred at r.t. until complete (48 h, monitored by TLC). The solution was then basified with aqueous 3 M NaOH to pH 12 and extracted with EtOAc (3 x 30 mL). The combined organic layers were washed with brine (30 mL), then dried over Na_2SO_4 and concentrated under a vacuum. Purification by gradient column chromatography (ethyl acetate:pentane = 1:19 to 3:17) afforded **11i** (665 mg, 82% yield) as a white solid.

MP: 116–118 °C.

IR (film) $\tilde{\nu}_{max}$/cm^{-1}: 2957, 2361, 2341, 1716, 1452, 1097, 893, 701.

^{1}H NMR (400 MHz, $CDCl_3$) δ: 7.58 – 7.53 (m, 2H, H-Ar), 7.50 – 7.44 (m, 2H, H-Ar), 7.39 – 7.34 (m, 1H, H-Ar), 7.31 – 7.16 (m, 5H, H-Ar), 5.73 (q, J = 1.0 Hz, 1H, H-7a), 5.33 (q, J = 1.0 Hz, 1H, H-7b), 3.61 (s, 2H, H-13), 3.33 (s, 2H, H-5), 2.56 – 2.46 (m, 4H, H-2, H-3), 2.44 – 2.32 (m, 2H, H-3), 2.26 – 2.16 (m, 2H, H-2).

^{13}C NMR (101 MHz, $CDCl_3$) δ: 211.2 (C-1), 140.9 (C-Ar), 140.0 (C-Ar), 134.0 (C-6), 128.8 (4xC-Ar), 128.3 (2xC-Ar), 127.7 (C-Ar), 127.4 (2xC-Ar), 127.0 (C-Ar), 118.2 (C-7), 62.8 (C-4), 59.2 (C-5), 54.7 (C-12), 38.1 (2xC-2), 33.5 (2xC-3).

HRMS (ES+): Exact mass calculated for $[M+H]^+$ $(C_{22}H_{25}ON^{79}Br)^+$ requires 398.1114, found 398.1115.

3.2.4 Synthesis of Trisubstituted Alkene Substrates 11j, 11k and 11l

Scheme S6. Preparation of substituted alkenes 11j – l.

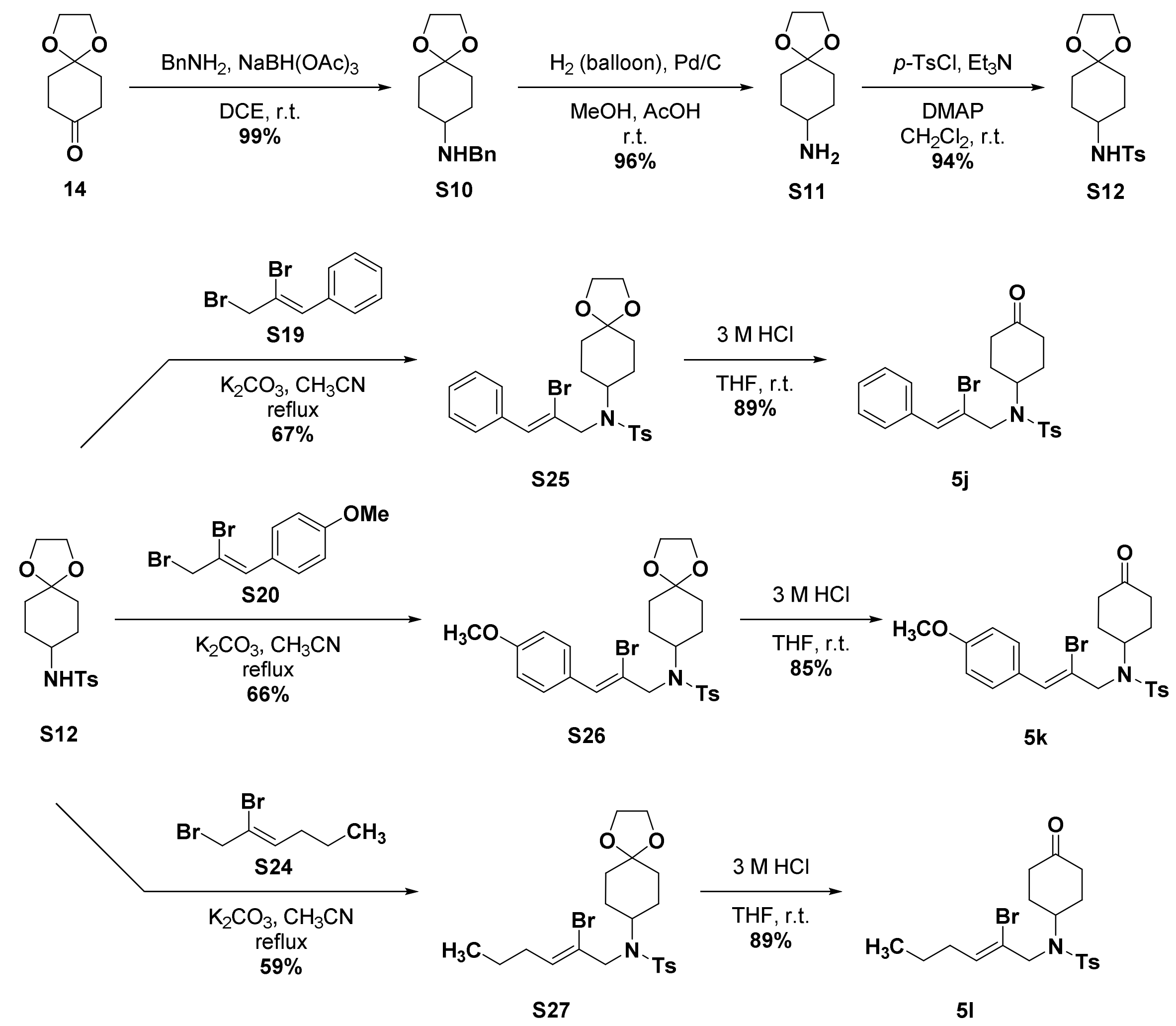

***N*-benzyl-1,4-dioxaspiro[4.5]decan-8-amine (S10)**

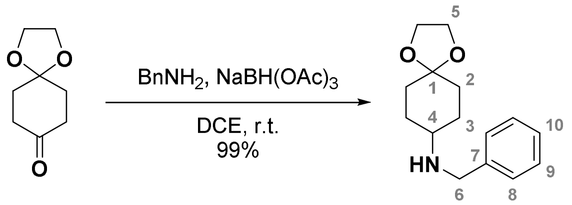

14 **S10**

To a solution of 1,4-cyclohexanedione monoethylene acetal (10.0 g, 64.0 mmol, 1 equiv.) in DCE (150 mL) was added benzylamine (7.0 mL, 64.0 mmol, 1.00 equiv.) dropwise at 0 °C fallowed by $NaBH(OAc)_3$ (19.0 g, 89.6 mmol, 1.40 equiv.) portionwise. The mixture was stirred at r.t. overnight until complete, before quenching with 2 M solution of NaOH. The layers were separated, and the aqueous layer was extracted with CH_2Cl_2 (3 x 100 mL), then the combined organic layers were washed with brine (100 mL), dried over Na_2SO_4, filtered and concentrated. Amine **S10** was obtained as a pale-yellow oil (15.8 g, 99% yield) and was used without further purification. The spectroscopic data match those reported in the literature.[16]

^{1}H NMR (400 MHz, $CDCl_3$) δ: 7.37 – 7.27 (m, 4H, H-8,H-9), 7.28 – 7.20 (m, 1H, H-10), 3.93 (s, 4H, H-5), 3.81 (s, 2H, H-6), 2.61 (tt, *J* = 9.7, 3.7 Hz, 1H, H-4), 1.95 – 1.85 (m, 2H, H-3), 1.84 – 1.75 (m, 2H, H-2), 1.62 – 1.41 (m, 4H, H-2, H-3), 1.25 (br s, 1H, NH).

^{13}C NMR (101 MHz, $CDCl_3$) δ: 141.0 (C-7), 128.5 (C-8), 128.2 (C-9), 126.9 (C-10), 108.9 (C-1), 64.4 (C-5), 64.4 (C-5), 54.5 (C-4), 51.4 (C-6), 33.0 (C-2), 30.3 (C-3).

1,4-dioxaspiro[4.5]decan-8-amine (S11)

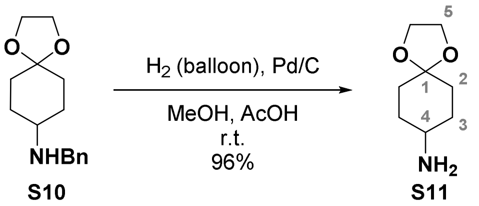

To a solution of amine **S10** (15.5 g, 62.7 mmol, 1 equiv.) in a mixture of MeOH (251 mL) and AcOH (34 mL) under N_2 was added palladium on carbon (3.33 g, 3.13 mmol, 10 wt. % loading, 0.05 equiv.) portionwise. H_2 was bubbled through the solution for 2 mins, then the mixture was stirred under a H_2 atmosphere (balloon, two skins) for 24 h. Upon completion, the reaction mixture was filtered through Celite and washed with EtOAc. The filtrate was then washed with aqueous 3 M KOH, then the phases were separated, and the aqueous phase was extracted with EtOAc (3 x 150 mL). The combined organic layers were dried over Na_2SO_4, filtered and concentrated to afford amine **S11** (9.41 g, 96% yield) as a colorless oil which was sufficiently pure to be used without further purification.

The spectroscopic data match those reported in the literature.[17]

^{1}H NMR (400 MHz, CDCl$_3$) δ: 3.91 (s, 4H, H-5), 2.74 (tt, *J* = 10.2, 3.9 Hz, 1H, H-4), 1.87 – 1.67 (m, 4H, H-2, H-3), 1.55 (td, *J* = 12.9, 4.0 Hz, 2H, H-2), 1.48 – 1.31 (m, 2H, H-3), 1.19 (s, 2H, NH_2).

^{13}C NMR (101 MHz, CDCl$_3$) δ: 108.4 (C-1), 64.3 (C-5), 64.3 (C-5), 49.2 (C-4), 33.7 (C-3), 33.2 (C-2).

4-methyl-*N*-(1,4-dioxaspiro[4.5]decan-8-yl)benzenesulfonamide (S12)

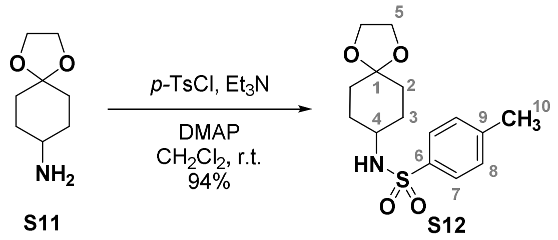

Prepared according to General Procedure A using 1,4-dioxaspiro[4.5]decan-8-amine **(S11)** (5.00 g, 31.8 mmol, 1 equiv.), Et_3N (17.7 mL, 127 mmol, 4.00 equiv.), DMAP (389 mg, 3.18 mmol, 0.10 equiv.), and 4-toluenesulfonyl chloride (12.1 g, 63.6 mmol, 2.00 equiv.) in CH_2Cl_2 (160 mL) were stirred at r.t. overnight. Purification by gradient column chromatography (ethyl acetate:pentane = 1:4 to 3:2) afforded **S12** (9.31 g, 94% yield) as a white solid.

MP: 190–192 °C

IR (film) $\tilde{\nu}_{max}$/cm^{-1}: 3246, 2934, 1443, 1317, 1160, 1090, 1028, 928, 817, 669.

^{1}H NMR (400 MHz, $CDCl_3$) δ: 7.78 – 7.73 (m, 2H, H-7), 7.31 – 7.26 (m, 2H, H-8), 3.87 (s, 4H, H-5), 3.26 – 3.14 (m, 1H, H-4), 2.41 (s, 3H, H-10), 1.80 – 1.71 (m, 2H, H-3), 1.71 – 1.58 (m, 2H, H-2), 1.56 – 1.43 (m, 4H, H-2, H-3).

^{13}C NMR (101 MHz, $CDCl_3$) δ: 143.4 (C-9), 138.3 (C-6), 129.8 (2xC-8), 127.0 (2xC-7), 107.6 (C-1), 64.4 (2xC-5), 51.1 (C-4), 32.8 (2xC-2), 30.7 (2xC-3), 21.6 (C-10).

HRMS (ES+): Exact mass calculated for $[M+Na]^+$ $(C_{15}H_{21}O_4N^{23}Na^{32}S)^+$ requires 334.1084, found 334.1083.

(Z)-(2,3-dibromoprop-1-en-1-yl)benzene (S19) and (Z)-1-(2,3-dibromoprop-1-en-1-yl)-4-methoxybenzene (S20)

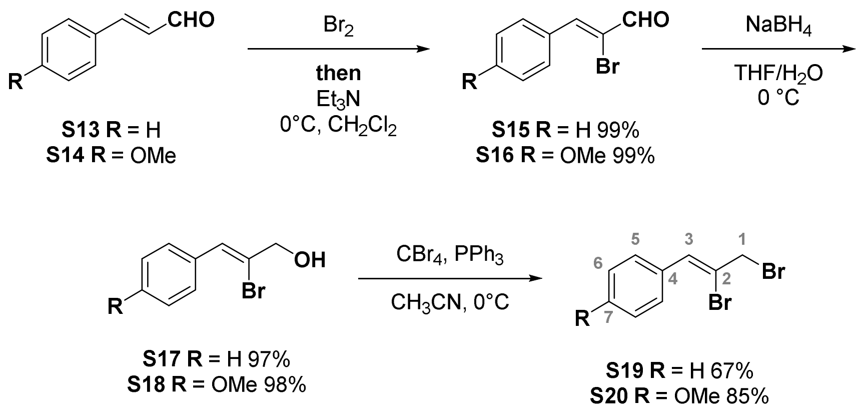

Compound **S15** was prepared according to a literature procedure.[18] To a solution of aldehyde **S13** (3.96 g, 30.0 mmol, 1 equiv) in CH_2Cl_2 (40 mL) was added dropwise Br_2 (1.84 mL, 36.0 mmol, 1.20 equiv.) at 0 °C. The reaction mixture was stirred for 30 min, followed by the addition of Et_3N (7.11 mL, 50.9 mmol, 1.7 equiv.). After stirring for an additional 30 min, the reaction mixture was diluted with CH_2Cl_2 and washed sequentially with a saturated aqueous solution of $Na_2S_2O_3$, H_2O, and brine. The organic layer was dried over anhydrous Na_2SO_4, filtered and concentrated under reduced pressure. The crude product **S15** was obtained as a brown oil (6.30 g, 99%), which after standing at room temperature for 3 days, crystalized as *Z*-isomer. Product **S15** was sufficiently pure and was used in the next step without further purification.

Prepared according to a literature procedure.[19] To a solution of aldehyde **S15** (5.07 g, 24.0 mmol, 1 equiv.) in 9:1 THF (98 mL) to H_2O (11 mL) was added portionwise $NaBH_4$ (1.03 g, 27.1 mmol, 1.13 equiv.) at 0 °C. The reaction mixture was stirred for 1 hour and then carefully quenched with a saturated aqueous solution of NH_4Cl (60 mL), extracted with Et_2O (3 x 40 mL). The combined organic phases were washed with H_2O (50 mL) and brine (50 mL), dried over anhydrous Na_2SO_4, filtered, and evaporated under reduced pressure. Product **S17** was obtained as a brown oil (4.96 g, 97%) in sufficient purity to be used in the next step without further purification.

Prepared according to a literature procedure.[20] To a solution of alcohol **S17** (716 mg, 3.36 mmol, 1 equiv.), in MeCN (6.7 mL) was added portionwise CBr_4 (1.67 g, 5.04 mmol, 1.5 equiv.) and PPh_3 (1.32 g, 5.04 mmol, 1.5 equiv.) at 0 °C. The mixture was stirred for 40 minutes and then filtered through Celite, washed with Et_2O, and concentrated under reduced pressure. The obtained residue was purified by column chromatography (pentane) to afford the title compound (661 mg, 67%) as a colorless oil. The spectroscopic data match those reported in the literature.[20]

^{1}H NMR (400 MHz, CDCl$_3$) δ: 7.68 – 7.59 (m, 2H, H-6), 7.48 – 7.31 (m, 3H, H-7, H-5), 7.14 (s, 1H, H-3), 4.44 (d, *J* = 0.7 Hz, 2H, H-1).

^{13}C NMR (101 MHz, CDCl$_3$) δ: 134.7 (C-4), 132.4 (C-3), 129.2 (2xC-Ar), 128.9 (C-7), 128.4 (2xC-Ar), 120.9 (C-2), 40.8 (C-1).

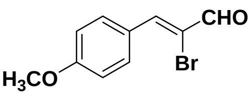

S16

Aldehyde **S16** was prepared according to the procedure used for **S13**, using aldehyde **S14** (4.87 g, 30.0 mmol, 1 equiv.), Br$_2$ (1.84 mL, 36.0 mmol, 1.20 equiv.) and Et$_3$N (7.11 mL, 50.9 mmol, 1.7 equiv.). Compound **S16** was obtained as a brown oil (7.20 g, 99%), single isomer and was used in the next step without further purification.

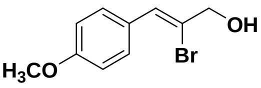

S18

Compound **S18** was prepared according to the procedure used for **S17**, using aldehyde **S16** (5.79 g, 2.00 mmol, 1 equiv.) and NaBH$_4$ (1.03 g, 27.1 mmol, 1.13 equiv.). Compound **S18** was obtained as a light brown solid (7.20 g, 98%), 5:1 mixture of inseparable Z/E-isomers, and was used in the next step without further purification.

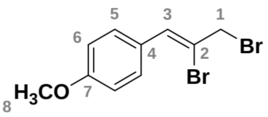

S20

Bromide **S20** was prepared according to the procedure used for **S19**, using alcohol **S18** (1.70 g, 7.00 mmol, 1 equiv.), CBr$_4$ (3.49 g, 10.5 mmol, 1.5 equiv.) and PPh$_3$ (2.75 g, 10.5 mmol, 1.5 equiv.). Compound **S18** was obtained as a pale-yellow oil (1.85 g, 85%), 5.8:1 mixture of inseparable Z/E-isomers.

^{1}H NMR (400 MHz, CDCl$_3$) δ: (mixture of Z/E isomers, minor *E*-isomer marked with "*") 7.70 – 7.61 (m, 2H, H-5), 7.35 – 7.24 (m, 2H, H-5*), 7.06 (s, 1H, H-3), 6.98 – 6.87 (m, 2H, H-6, H-6*), 4.45 (s, 2H, H-1), 4.43 (s, 2H, H-1*), 3.83 (s, 3H, H-8, H-8*).

^{13}C NMR (101 MHz, CDCl$_3$) δ: (mixture of Z/E isomers, minor *E*-isomer marked with "*") 160.2 (C-7), 159.7 (C-7*), 136.7 (C-3*), 131.9 (C-3), 130.8 (2xC-5), 129.7 (2xC-5*), 128.0 (C-4*), 127.1 (C-4), 120.9 (C-2*), 118.7 (C-2), 114.5 (2xC-2*), 113.8 (2xC-6), 55.5 (C-8*), 55.4 (C-8), 41.6 (C-1), 35.8 (C-1*).

IR (film) $\tilde{\nu}_{max}$/cm^{-1}: 2956, 2835, 1604, 1509, 1251, 1177, 1030, 823, 667.

HRMS (ES+): Exact mass calculated for $[M+H]^+$ $(C_{10}H_{11}O^{79}Br_2)^+$ requires 306.9151, found 306.9152.

ethyl (Z)-2-bromohex-2-enoate (22)

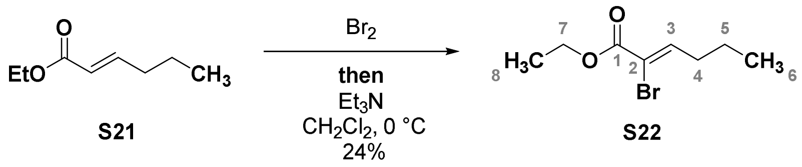

Aldehyde **S22** was prepared according to the procedure used for **S13**, using ester **S21** (1.5 mL, 10.0 mmol, 1 equiv.), Br_2 (0.62 mL, 12.0 mmol, 1.20 equiv.) and Et_3N (2.36 mL, 17.0 mmol, 1.7 equiv.). Purification by column chromatography (pentane then diethyl ether:pentane = 1:499) afforded compound **S22** as a colorless oil (540 g, 24%), 9:1 mixture of inseparable Z/E-isomers.

^{1}H NMR (400 MHz, $CDCl_3$) δ: 7.28 (t, J = 7.0 Hz, 1H, H-3), 4.27 (q, J = 7.0 Hz, 2H, H-7), 2.32 (q, J = 7.5 Hz, 2H, H-4), 1.54 (h, J = 7.5 Hz, 2H, H-5), 1.33 (t, J = 7.0 Hz, 3H, H-8), 0.98 (t, J = 7.5 Hz, 3H, H-6).

^{13}C NMR (101 MHz, $CDCl_3$) δ: 163.7 (C-1), 146.1 (C-3), 117.6 (C-2), 62.5 (C-7), 34.2 (C-4), 21.1 (C-5), 14.3 (C-8), 14.0 (C-6).

IR (film) $\tilde{\nu}_{max}$/cm^{-1}: 2961, 1727, 1715, 1625, 1462, 1252, 1221, 1034, 744.

HRMS (ES+): Exact mass calculated for $[M+H]^+$ $(C_8H_{14}O_2{}^{79}Br)^+$ requires 221.0172, found 221.0174.

(Z)-1,2-dibromohex-2-ene (S24)

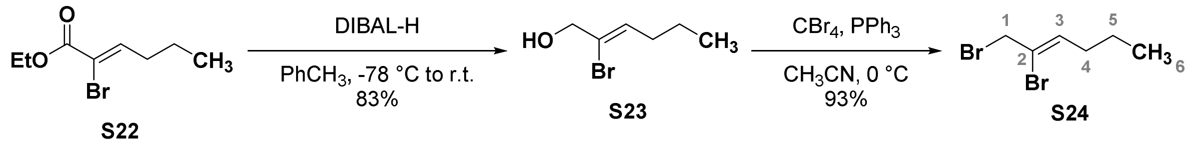

To a stirring solution of compound **S22** (442 mg, 2.00 mmol, 1 equiv.) in PhMe (20 mL) was added dropwise diisobutylaluminium hydride solution (5.0 mL, 1 M in THF, 5.00 mmol, 2.50 equiv) at -78 °C. The reaction mixture was allowed to warm to r.t., and was stirred for 1 h, then carefully quenched with MeOH (5.0 mL) and sat. Rochelle's salt (aq, 5.0 mL), extracted with Et_2O (3 x 20 mL), and washed with H_2O (20 mL) and brine (20 mL). The combined organic layers were dried with Na_2SO_4, filtered, and evaporated under reduced pressure. Compound **S23** was obtained as a clear oil (296 mg, 83% yield), which was sufficiently pure to be used without further purification.

Bromide **S24** was prepared according to the procedure used for **S19**, using alcohol **S23** (286 mg, 1.60 mmol, 1 equiv.), CBr_4 (794 mg, 2.40 mmol, 1.50 equiv.) and PPh_3 (628 mg, 2.40 mmol, 1.50 equiv.). Purification by column chromatography (pentane) afforded compound **S24** as a colorless oil (359 mg, 93%). The spectroscopic data match those reported in the literature.[21]

^{1}H NMR (400 MHz, $CDCl_3$) δ: 6.12 (tt, J = 7.0, 1.0 Hz, 1H, H-3), 4.25 (d, J = 1.0, 2H, H-1), 2.17 (qt, J = 7.0, 1.0 Hz, 2H, H-4), 1.46 (h, J = 7.5 Hz, 2H, H-5), 0.94 (t, J = 7.5 Hz, 3H, H-6).

^{13}C NMR (101 MHz, $CDCl_3$) δ: 135.0 (C-3), 122.6 (C-2), 39.1 (C-1), 33.7 (C-4), 21.4 (C-5), 13.8 (C-6).

(*Z*)-*N*-(2-bromo-3-phenylallyl)-4-methyl-*N*-(1,4-dioxaspiro[4.5]decan-8-yl)benzenesulfonamide (S25)

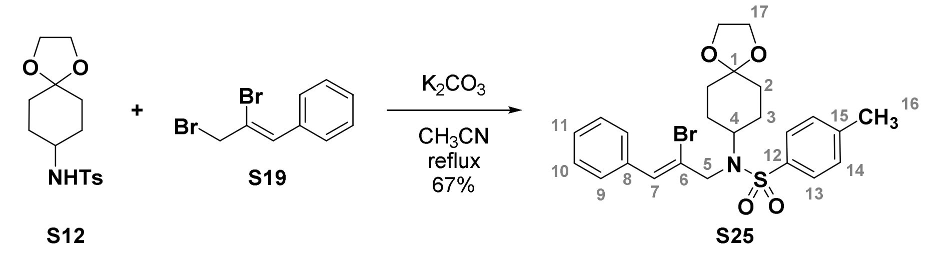

Amine **S12** (1.02 g, 3.26 mmol, 1 equiv.) was reacted according to the procedure for **11g**, using K_2CO_3 (1.80 g, 13.0 mmol, 4.00 equiv.) and bromide **S19** (900 mg, 3.26 mmol, 1.00 equiv.) instead of 2,3-dibromopropene. After refluxing the reaction overnight, the purification by column chromatography (ethyl acetate:pentane = 1:4) afforded **S25** (1.10 g, 67% yield) as a white solid.

MP: 121–123 °C.

IR (film) $\tilde{\nu}_{max}$/cm^{-1}: 2944, 1331, 1152.

^{1}H NMR (400 MHz, $CDCl_3$) δ: 7.77 – 7.72 (m, 2H, H-13), 7.75 – 7.49 (m, 2H, H-10), 7.37 – 7.31 (m, 2H, H-9), 7.31 – 7.26 (m, 3H, H-14, H-11), 7.08 (s, 1H, H-7), 4.22 (d, *J* = 1.5 Hz, 2H, H-5), 3.93 – 3.84 (m, 5H, H-17, H-4), 2.41 (s, 3H, H-16), 1.82 – 1.68 (m, 4H, H-2, H-3), 1.68 – 1.53 (m, 4H, H-2, H-3).

^{13}C NMR (101 MHz, $CDCl_3$) δ: 143.6 (C-15), 138.2 (C-12), 135.4 (C-8), 129.9 (2xC-14), 129.3 (C-7), 129.1 (2xC-10), 128.2 (2xC-9), 128.1 (C-11), 127.3 (2xC-13), 123.1 (C-6), 107.3 (C-1), 64.5 (C-17), 64.4 (C-17), 57.2 (C-4), 52.6 (C-5), 34.3 (2xC-2), 28.1 (2xC-3), 21.7 (C-16). //by RK

HRMS (ES+): Exact mass calculated for $[M+Na]^+$ ($C_{24}H_{28}O_4N^{79}Br^{23}Na^{32}S)^+$ requires 528.0815, found 528.0815.

(*Z*)-*N*-(2-bromo-3-phenylallyl)-4-methyl-*N*-(4-oxocyclohexyl)benzenesulfonamide (11j)

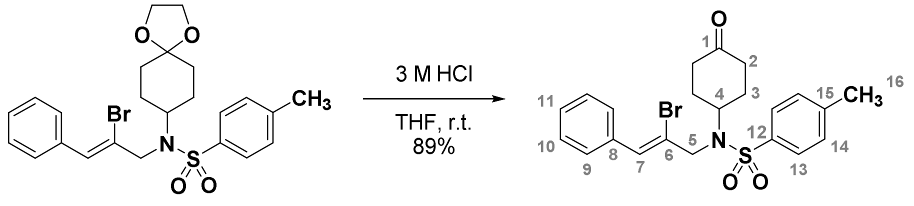

Acetal **S25** (800 mg, 1.58 mmol, 1 equiv.) was reacted in a 1:1 mixture of THF (7.9 mL) and 3 M HCl (7.9 mL) according to the procedure given for compound **S3.** Purification by column chromatography (diethyl ether:pentane = 2:3) afforded **5j** (653 mg, 89% yield) as a white solid.

MP: 114–116 °C.

IR (film) $\tilde{\nu}_{max}$/cm^{-1}: 2980, 1708, 1324, 1161, 1089, 1048.

^{1}H NMR (400 MHz, $CDCl_3$) δ: 7.80 – 7.75 (m, 2H, H-13), 7.54 – 7.50 (m, 2H, H-10), 7.38 – 7.28 (m, 5H, H-14, H-9, H-11), 7.07 (s, 1H, H-7), 4.30 (tt, *J* = 11.8, 3.8 Hz, 1H, H-4), 4.23 (d, *J* = 1.4 Hz, 2H, H-5), 2.43 (s, 3H, H-16), 2.48 – 2.35 (m, 4H, H-2), 2.12 – 1.86 (m, 4H, H-3).

^{13}C NMR (101 MHz, $CDCl_3$) δ: 208.5 (C-1), 143.9 (C-15), 137.8 (C-12), 134.9 (C-8), 130.0 (2xC-14), 129.9 (C-7), 129.1 (2xC-10), 128.4 (C-11), 128.3 (2xC-9), 127.2 (2xC-13), 122.4 (C-6), 56.4 (C-4), 53.2 (C-5), 40.1 (2xC-2), 30.2 (2xC-3) , 21.7 (C-16).

HRMS (ES+): Exact mass calculated for $[M+H]^+$ $(C_{22}H_{25}O_3N^{79}Br^{32}S)^+$ requires 462.0733, found 462.0734.

(*Z*)-*N*-(2-bromo-3-(4-methoxyphenyl)allyl)-4-methyl-*N*-(1,4-dioxaspiro[4.5]decan-8-yl)benzenesulfonamide (S26)

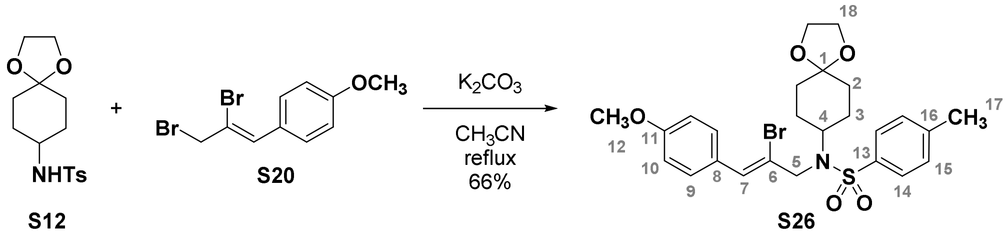

Amine **S12** (0.509 g, 1.63 mmol, 1 equiv.) was reacted according to the procedure for **11g**, using K_2CO_3 (904 mg, 6.54 mmol, 4.00 equiv.) and bromide **S20** (500 mg, 1.63 mmol, 1.00 equiv.) instead of 2,3-dibromopropene. After refluxing the reaction mixture overnight, the purification by column chromatography (ethyl acetate:pentane = 1:4) afforded **S26** (811 mg, 92% yield, 4.8:1 Z/E mixture) as a colorless oil.

Recrystallization of the product from the mixture of ethyl acetate (5 mL) and hexane (15 mL) provided pure Z-isomer (508 mg, 58%) as a white solid. Further recrystallization of the mother liquor provided another 70 mg (8.0%) of Z-isomer.

MP: 146-148 °C.

IR (film) $\tilde{v}_{max}$/cm^{-1}: 2947, 1606, 1510, 1334, 1250, 1154, 1090, 1032, 817, 667.

^{1}H NMR (400 MHz, $CDCl_3$) δ: 7.76 – 7.71 (m, 2H, H-14), 7.53 – 7.48 (m, 2H, H-9), 7.29 – 7.25 (m, 2H, H-15), 7.00 (s, 1H, H-7), 6.90 -6.85 (m, 2H, H-10), 4.20 (d, J = 1.5 Hz, 2H, H-5), 3.93 – 3.83 (m, 5H, H-18, H-4), 3.82 (s, 3H, H-12), 2.41 (s, 3H, H-17), 1.83 – 1.68 (m, 4H, H-2, H-3), 1.68 – 1.52 (m, 4H, H-2, H-3).

^{13}C NMR (101 MHz, $CDCl_3$) δ: 159.5 (C-11), 143.5 (C-16), 138.3 (C-13), 130.5 (2xC-9), 129.8 (2xC-15), 128.9 (C-7), 127.8 (C-8) 127.3 (2xC-14), 121.1 (C-6), 113.6 (2xC-10), 107.4 (C-1), 64.5 (C-18), 64.5 (C-18), 57.2 (C-4), 55.4 (C-12), 52.7 (C-5), 34.3 (2xC-2), 28.2 (2xC-3), 21.7 (C-17).

HRMS (ES+): Exact mass calculated for $[M+Na]^+$ ($C_{25}H_{30}O_5N^{79}Br^{23}Na^{32}S)^+$ requires 558.0920, found 558.0918.

(*Z*)-*N*-(2-bromo-3-(4-methoxyphenyl)allyl)-4-methyl-*N*-(4-oxocyclohexyl)benzenesulfonamide (11k)

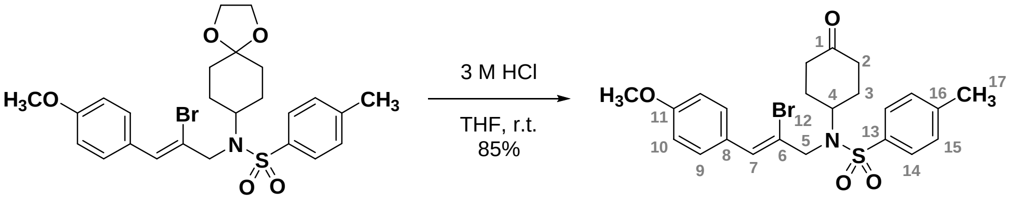

Acetal **S26** (900 mg, 1.68 mmol, 1 equiv.) was reacted in a 1:1 mixture of THF (8.4 mL) and 3 M HCl (8.4 mL) according to the procedure given for compound **S3.** Purification by gradient column chromatography (diethyl ether:pentane = 2:3 to 7:3) afforded **5k** (700 mg, 85% yield) as a white solid.

MP: 135–137 °C.

IR (film) $\tilde{\nu}_{max}$/cm^{-1}: 2947, 1712, 1510, 1253, 1240, 1161, 1152, 1021.

^{1}H NMR (400 MHz, CDCl$_3$) δ: 7.80 – 7.72 (m, 2H, H-14), 7.56 – 7.47 (m, 2H, H-9), 7.34 – 7.27 (m, 2H, H-15), 6.98 (s, 1H, H-7), 6.92 – 6.84 (m, 2H, H-10), 4.28 (tt, *J* = 11.7, 4.0 Hz, 1H, H-4), 4.22 (d, *J* = 1.0 Hz, 2H, H-5), 3.82 (s, 3H, H-12), 2.43 (s, 3H, H-17), 2.45 – 2.35 (m, 4H, H-2), 2.05 – 1.87 (m, 4H, H-3).

^{13}C NMR (101 MHz, CDCl$_3$) δ: 208.5 (C-1), 159.7 (C-11), 143.9 (C-16), 138.0 (C-13), 130.6 (2xC-9), 130.0 (2xC-15), 129.5 (C-7), 127.4 (C-8), 127.3 (2xC-14), 120.2 (C-6), 113.7 (2xC-10), 56.5 (C-4), 55.4 (C-12), 53.5 (C-5), 40.2 (2xC-2), 30.3 (2xC-3), 21.7 (C-17).

HRMS (ES+): Exact mass calculated for [M+Na]$^+$ ($C_{23}H_{26}O_4N^{79}Br^{23}Na^{32}S$)$^+$ requires 514.0658, found 514.0660.

(*Z*)-*N*-(2-bromohex-2-en-1-yl)-4-methyl-*N*-(1,4-dioxaspiro[4.5]decan-8-yl)benzenesulfonamide (S27)

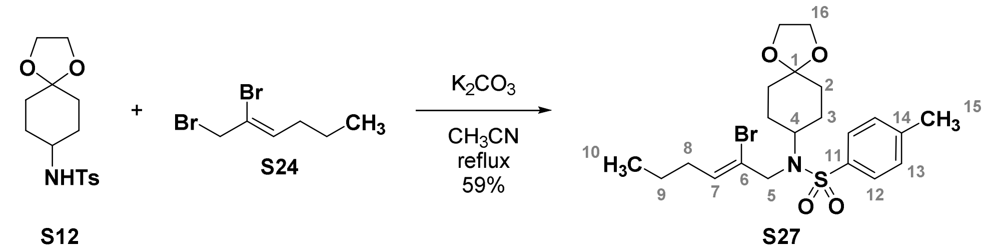

Amine **S12** (351 mg, 1.13 mmol, 1 equiv.) was reacted according to the procedure for **11g**, using K_2CO_3 (623 mg, 4.51 mmol, 4.00 equiv.) and bromide **S24**[21] (273 mg, 1.13 mmol, 1.00 equiv.) instead of 2,3-dibromopropene. Purification by column chromatography (ethyl acetate:pentane = 1:4) afforded **S27** (314 mg, 59% yield) as a white solid.

MP: 68–70 °C.

IR (film) $\tilde{\nu}_{max}$/cm^{-1}: 2957, 2873, 1336, 1155, 1091, 1035, 908, 730.

^{1}H NMR (400 MHz, $CDCl_3$) δ: 7.70 (d, J = 8.0 Hz, 2H, H-12), 7.27 (d, J = 8.0 Hz, 2H, H-13), 6.06 – 5.99 (m, 1H, H-7), 4.04 (s, 2H, H-5), 3.91 – 3.83 (m, 4H, H-16), 3.77 (tt, J = 12.5, 4.0 Hz, 1H, H-4), 2.41 (s, 3H, H-15), 2.13 (q, J = 7.0 Hz, 2H, H-8), 1.77 – 1.62 (m, 4H, H-2, H-3), 1.60 – 1.48 (m, 4H, H-2, H-3), 1.40 (h, J = 7.5 Hz, 2H, H-9), 0.91 (t, J = 7.5 Hz, 3H, H-10).

^{13}C NMR (101 MHz, $CDCl_3$) δ: 143.4 (C-14), 138.3 (C-11), 131.2 (C-7), 129.7 (2xC-13), 127.2 (2xC-12), 123.9 (C-6), 107.4 (C-1), 64.5 (C-16), 64.3 (C-16), 57.1 (C-4), 51.5 (C-5), 34.3 (2xC-2), 33.2 (C-8), 28.0 (2xC-3), 21.6 (C-9), 21.5 (C-15), 13.9 (C-10).

HRMS (ES+): Exact mass calculated for $[M+H]^+$ $(C_{21}H_{31}O_4N^{79}Br^{32}S)^+$ requires 472.1152, found 472.1149.

(*Z*)-*N*-(2-bromohex-2-en-1-yl)-4-methyl-*N*-(4-oxocyclohexyl)benzenesulfonamide (11l)

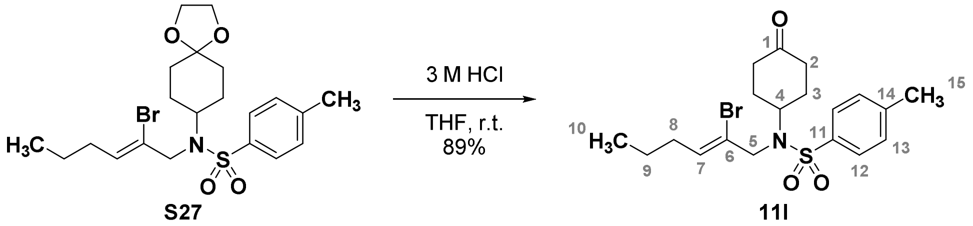

Acetal **S27** (260 mg, 0.55 mmol) was reacted in a 1:1 mixture of THF (2.75 mL) and 3 M HCl (2.75 mL) according to the procedure given for compound **S3** and purified by column chromatography (ethyl acetate:pentane = 1:4) to afford **5l** (210 mg, 89% yield) as a white solid.

MP: 79–81 °C

IR (film) $\tilde{\nu}_{max}$/cm^{-1}: 2958 , 1717, 1340, 1158, 1090, 1045, 916, 814, 656

^{1}H NMR (400 MHz, $CDCl_3$) δ: 7.76 – 7.71 (m, 2H, H-12), 7.33 – 7.29 (m, 2H, H-13), 6.03 (tt, J = 7.0, 1.5 Hz, 1H, H-7), 4.19 (tt, J = 11.5, 4.5 Hz, 1H, H-4), 4.08 – 4.05 (m, 2H, H-5), 2.43 (s, 3H, H-15), 2.40 – 2.34 (m, 4H, H-2), 2.18 – 2.10 (m, 2H, H-8), 1.98 – 1.81 (m, 4H, H-3), 1.40 (h, J = 7.5 Hz, 2H, H-9), 0.91 (t, J = 7.5 Hz, 3H, H-10).

^{13}C NMR (101 MHz, $CDCl_3$) δ: 208.6 (C-1), 143.8 (C-14), 138.0 (C-11), 132.1 (C-7), 130.0 (2xC-13), 127.3 (2xC-12), 123.6 (C-6), 56.3 (C-4), 52.1 (C-5), 40.2 (2xC-2), 33.2 (C-8), 30.2 (2xC-3), 21.7 (C-15), 21.6 (C-9), 13.9 (C-10).

HRMS (ES+): Exact mass calculated for $[M+H]^+$ $(C_{19}H_{27}O_3N^{79}Br^{32}S)^+$ requires 428.0890, found 428.0888.

3.3 Pd-catalyzed cyclization

3.3.1 General Procedure B – Enantioselective Cyclization

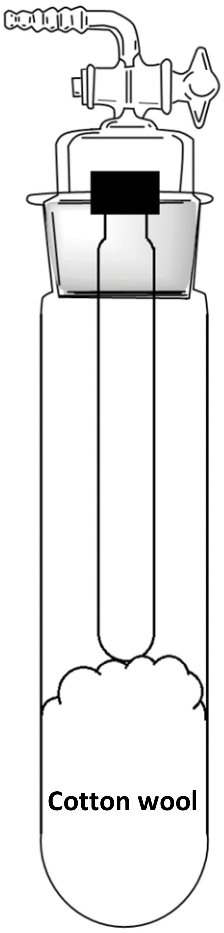

In the following procedure, degassing refers to three cycles of high vacuum (6.10^{-2} mbar) followed by backfilling with N_2. An oven-dried 15-mL screw-capped tube, with a cap and PTFE septum loosely attached, was cooled under a vacuum and then degassed (placed in a bigger tube, which was attached to a Schlenk line). The vessel was charged with the substrate (0.10 mmol), $Pd(OAc)_2$ (1.1 mg, 0.005 mmol, 5 mol%), tris(4-trifluoromethylphenyl)phosphine (7.0 mg, 0.015 mmol, 15 mol%), (*2R,4R*)-4-hydroxypyrrolidine-2-carboxylic acid (2.6 mg, 0.02 mmol, 20 mol%), and K_2HPO_4 (26.1 mg, 0.15 mmol, 1.5 equiv.). During weighing, the vessel was capped. The vessel was degassed before dry, degassed MeOH (2 mL) was added, and then the vessel was again degassed while stirring (carefully to avoid bumping or excessive solvent evaporation).

After sealing under a stream of N_2 with the screw cap, the reaction mixture was stirred at room temperature for 5 minutes before transferring to a heating block pre-heated to 85 °C and stirring at that temperature for the specified time (24–48 h).

The reaction tube was then removed from the heating block and allowed to cool to room temperature before diluting with CH_2Cl_2 (2 mL) and filtering through a plug of Celite (1 cm) and silica (1 cm). After eluting with additional CH_2Cl_2 (5 mL), the filtrate was concentrated under a vacuum.

NMR yields and NMR conversions were obtained by dissolving the crude reaction mixture in $CDCl_3$ before adding the appropriate volume of internal standard (mesitylene, 14 µL, 0.10 mmol) and transferring a small volume to an NMR tube, diluting with additional $CDCl_3$. Purification by column chromatography yielded the cyclized product. Enantiomeric excess was measured using HPLC with a chiral stationary phase and a hexane/isopropanol mobile phase as specified for each compound. The racemic samples were prepared by performing the reaction with 1:1 mixture of (2*R*,4*R*) and (*2S,4S*)-4-hydroxypyrrolidine-2-carboxylic acid.

3.3.2 Cyclized Products 5a–5l

Scheme S7. Scope of the desymmetrizing vinylation reaction.[a]

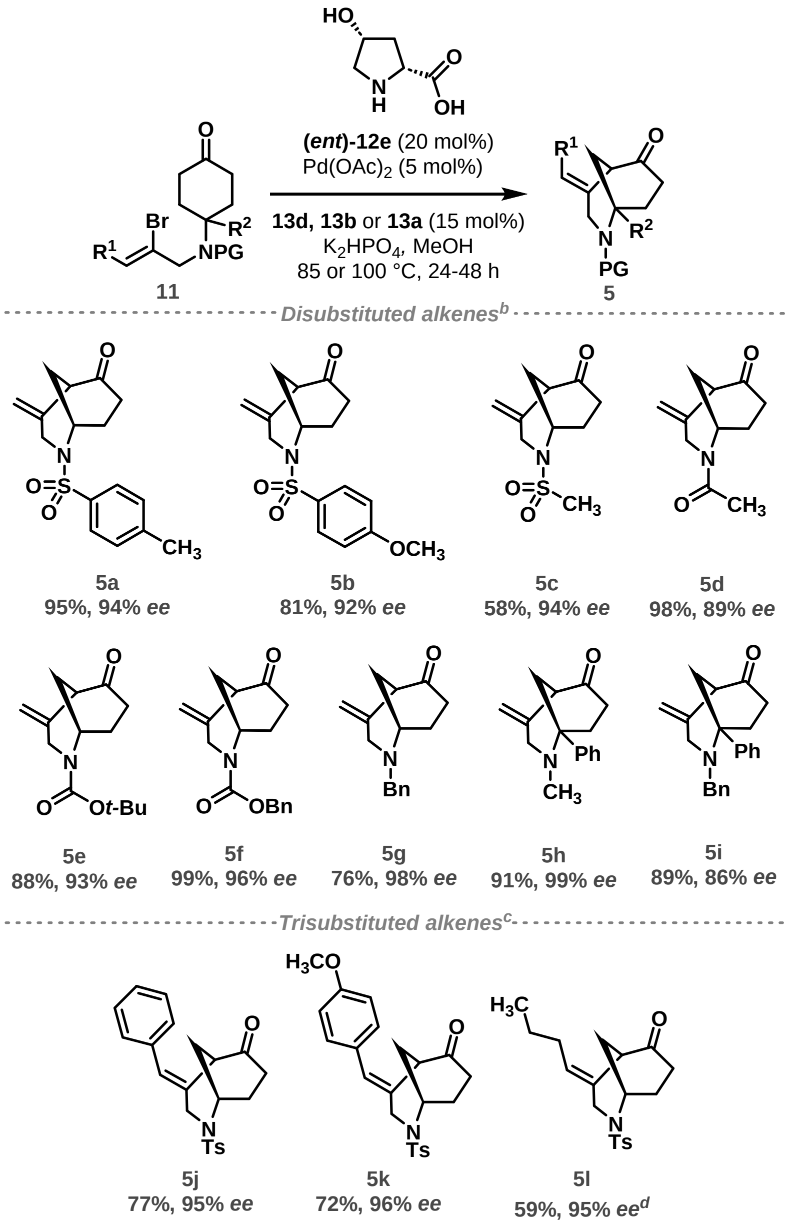

[a] Reagents and conditions: [b]**11** (0.10 mmol), ***(ent)*-12e** (20 mol%), $Pd(OAc)_2$ (5 mol%), **13d** (15 mol%), K_2HPO_4 (1.5 equiv), MeOH (0.05 M), 85 °C, 24–48 h. [c] **13b** used instead of **13d**, 100 °C. [d]**13a** used instead of **13d**, 100 °C Isolated yields; enantioselectivity determined by chiral HPLC analysis.

(*1S,5R*)-4-methylene-2-tosyl-2-azabicyclo[3.3.1]nonan-6-one ((+)-5a)

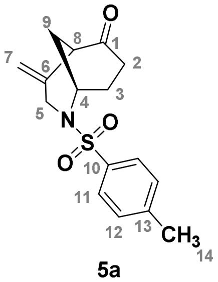

5a

Prepared according to General Procedure B using **11a** (38.6 mg, 0.10 mmol). Purification by column chromatography (ethyl acetate:pentane = 2:3) afforded **(+)-5a** (29 mg, 95% yield, 94% *ee*) as a white solid. The enantiomeric form **(–)-5a** was prepared in the same manner, using (2*R*,4*R*)-4-hydroxyproline to afford **(–)-5a** (29 mg, 95% yield, 94% *ee*) as a white solid. Data is in agreement with literature values.[14]

MP: 92–94 (>99% ee), 140–142 °C (racemic, lit. 136–138 °C).[14]

IR (film) $\tilde{\nu}_{max}$/cm^{-1}: 2925, 1714, 1344, 1161, 1096.

^{1}H NMR (500 MHz, $CDCl_3$) δ: 7.75 (d, *J* = 8.0 Hz, 2H, H-11), 7.36 (d, *J* = 8.0 Hz, 2H, H-12), 5.13 (t, *J* = 1.5 Hz, 1H, H-9a), 5.04 (t, *J* = 1.5 Hz, 1H, H-9b), 4.19 (d, *J* = 14.0 Hz, 1H, H-5a), 4.12 (p, *J* = 3.0 Hz, 1H, H-4), 3.77 (dq, *J* = 14.0, 1.5 Hz, 1H, H-5b), 3.25 (s, 1H, H-8), 2.83 (ddd, *J* = 15.5, 13.0, 7.5 Hz, 1H, H-2a), 2.46 (s, 3H, H-14), 2.43–2.34 (m, 1H, H-3a), 2.34–2.25 (m, 1H, H-2b), 1.99 (dq, *J* = 14.0, 3.0 Hz, 1H, H-9a), 1.94–1.82 (m, 2H, H-9b, H-3b).

^{13}C NMR (101 MHz, $CDCl_3$) δ: 208.3 (C-1), 143.9 (C-6), 138.2 (C-10), 134.8 (C-13), 130.0 (C-12), 127.4 (C-11), 115.2 (C-7), 50.2 (C-8), 48.0 (C-4), 47.2 (C-5), 34.8 (C-2), 32.5 (C-3), 30.5 (C-9), 21.7 (C-14).

HRMS (ES+): Exact mass calculated for $[M+H]^+$ $(C_{16}H_{20}O_3N^{32}S)^+$ requires 306.1158, found 306.1159. $[\alpha]_D^{25}$=+94.6 (c = 1.00, $CHCl_3$, >99% *ee*).

HPLC (Chiralpak AD-H, hexane/isopropanol 90:10, 1.0 mL min^{-1}, λ =210 nm) t_R = 23.0 min (major), 27.2 min (minor).

(1*S*,5*R*)-2-((4-methoxyphenyl)sulfonyl)-4-methylene-2-azabicyclo[3.3.1]nonan-6-one ((+)-5b)

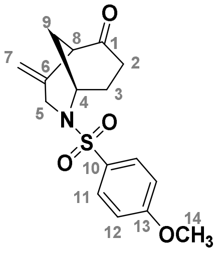

5b

Prepared according to General Procedure B using **11b** (40.2 mg, 0.10 mmol), except for 48 h reaction time instead of 24 h. Purification by column chromatography (ethyl acetate:pentane = 3:7) afforded **(+)-5b** (25.8 mg, 81% yield, 92% *ee*) as a white solid. The spectroscopic data match those reported in the literature.[14]

^{1}H NMR (400 MHz, $CDCl_3$) δ: 7.81 – 7.75 (m, 2H, H-11), 7.03 – 6.97 (m, 2H, H-12), 5.10 (d, *J* = 1.5 Hz, 1H, H7-a), 5.02 (dt, *J* = 1.5, 1.0 Hz, 1H, H-7b), 4.15 (d, *J* = 14.5 Hz, 1H, H-5a), 4.09 (t, *J* = 3.5 Hz, 1H, H-4), 3.88 (s, 3H, H-14), 3.75 (dtd, *J* = 14.0, 1.8, 0.8 Hz, 1H, H-5b), 3.23 (s, 1H, H-8), 2.80 (ddd, *J* = 16.0, 13.0, 7.5 Hz, 1H, H-2a), 2.42 – 2.32 (m, 1H, H-3a), 2.27 (ddt, *J* = 16.1, 6.1, 2.0 Hz, 1H, H-2b), 1.98 (dq, *J* = 13.9, 3.4 Hz, 1H, H-9a), 1.92 – 1.79 (m, 2H, H-3b, H-9b).

^{13}C NMR (101 MHz, $CDCl_3$) δ: 208.4 (C-1), 163.2 (C-13), 138.3 (C-6), 129.6 (C-11), 129.6 (C-10), 115.2 (C-7), 114.6 (C-12), 55.8 (C-14), 50.3 (C-8), 47.9 (C-4), 47.3 (C-3), 34.9 (C-2), 32.5 (C-3), 30.6 (C-9).

$[\boldsymbol{\alpha}]_{\boldsymbol{D}}^{\mathbf{25}}$= +108.4 (c = 1.00, $CHCl_3$, 92% *ee*).

HPLC (Chiralpak AD-H, hexane/isopropanol 95:5, 1.0 mL min^{-1}, λ = 210 nm) t_R = 65.3 (major), 70.7 (minor).

(*1S,5R*)-4-methylene-2-(methylsulfonyl)-2-azabicyclo[3.3.1]nonan-6-one ((+)-5c)

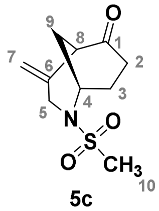

5c

Prepared according to General procedure B using **11c** (31.0 mg, 0.1 mmol). Purification by column chromatography (ethyl acetate:pentane = 1:1) afforded **(+)-5c** (13.3 mg, 58% yield, 94% *ee*) as an orange oil. The spectroscopic data match those reported in the literature.[14]

^{1}H NMR (400 MHz, $CDCl_3$) δ: 5.14 (d, *J* = 1.5 Hz, 1H, H-7a), 5.09 (q, *J* = 1.5 Hz, 1H, H-7b), 4.21 – 4.12 (m, 2H, H-5a, H-4), 4.03 – 3.96 (m, 1H, H-7b), 3.36 (s, 1H, H-8), 2.90 (s, 3H, H-10), 2.82 (ddd, *J* = 16.0, 13.5, 7.5 Hz, 1H, H-2a), 2.43 – 2.26 (m, 3H, H-2b, H-3a, H-9a), 2.07 (dt, *J* = 13.5, 3.0 Hz, 1H, H-9b), 1.94 (dddd, *J* = 14.5, 13.0, 6.5, 3.0 Hz, 1H, H-3b).

^{13}C NMR (101 MHz, $CDCl_3$) δ: 207.9 (C-1), 138.0 (C-6), 115.4 (C-7), 50.6 (C-8), 48.0 (C-4), 47.1 (C-5), 36.7 (C-10), 35.0 (C-2), 32.4 (C-3), 31.2 (C-9).

$[\alpha]_D^{25}$= +108.3 (c = 1.00, $CHCl_3$, 94% *ee*).

HPLC (Chiralpak AD-H, hexane/isopropanol 90:10, 1.0 mL min^{-1}, λ = 210 nm) t_R = 18.8 (minor), 21.2 (major).

(*1S,5R*)-2-acetyl-4-methylene-2-azabicyclo[3.3.1]nonan-6-one ((+)-5d)

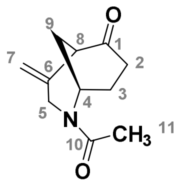

5d

Prepared according to General procedure B using **11d** (27.4 mg, 0.1 mmol). Purification by column chromatography (ethyl acetate) afforded **(+)-5d** (18.9 mg, 98% yield, 89% *ee*) as a yellow oil. The spectroscopic data match those reported in the literature.[14]

Note: Compound **5d** is a 3:2 mixture of rotamers in $CDCl_3$ at room temperature (* minor).

^{1}H NMR (400 MHz, $CDCl_3$) δ: 5.11 – 5.06 (m, 2H, H-7a, H-7a*), 5.04 (q, J = 1.5 Hz, 1H, H-7b), 5.00 (br s, 1H, H-7b*), 4.61 (p, J = 3.2 Hz, 1H, H-4), 4.44 – 4.29 (m, 2H, H-5a, H-5b), 4.20 (s, 2H, H-5a*, H-5b*), 4.17 (p, *J* = 3.0 Hz, 1H, H-4*), 3.35 (s, 2H, H-8, H-8*), 2.70 – 2.49 (m, 2H, H-2a, H-2a*), 2.46 – 2.32 (m, 2H, H-2b*, H-CH_2-Ba), 2.32 – 2.19 (m, 3H, H-CH_2-Aa*, H-CH_2-Aa, H-2b), 2.19 – 2.08 (m, H2, H-CH_2-Ba*, H-CH_2-Bb*) 2.17 (s, 3H, H-11*), 2.14 (s, 3H, H-11), 2.08 – 1.95 (m, 2H, H-CH_2-Ab, H-CH_2-Ab*), 1.79 (dddd, J = 14.0, 13.2, 5.9, 3.2 Hz, 1H, H-CH_2-Bb).

^{13}C NMR (101 MHz, $CDCl_3$) δ: 208.8 (C-1), 207.8 (C-1*), 170.4 (C-10*), 170.3 (C-10), 139.3 (C-6), 138.1 (C-6*), 114.8 (C-7*), 114.6 (C-7), 51.7 (C-8*), 50.9 (C-8), 48.4 (C-5), 47.5 (C-4*), 45.0 (C-5*, C-4), 35.5 (C-2*), 35.1 (C-2), 32.3 (CH_2-A*), 32.0 (CH_2-B*), 30.7 (CH_2-A), 30.0 (CH_2-B), 22.7 (C-11), 21.6 (C-11*).

$[\alpha]_D^{25}$= +264.0 (c = 1.00, $CHCl_3$, 89% *ee*).

HPLC (Chiralpak AS-H, hexane/isopropanol 70:30, 1.0 mL min^{-1}, λ = 210 nm) t_R = 28.6 (minor), 45.0 (major).

***tert*-butyl (*1S,5R*)-4-methylene-6-oxo-2-azabicyclo[3.3.1]nonane-2-carboxylate ((+)-5e)**

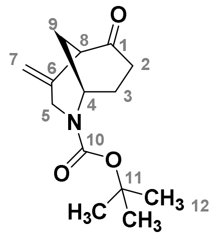

5e

Prepared according to General procedure B using **11e** (33.2 mg, 0.1 mmol). Purification by column chromatography (ethyl acetate:pentane = 1:3) afforded **(+)-5e** (22.1 mg, 88% yield, 93% *ee*) as a white solid. The spectroscopic data match those reported in the literature.[14]

MP: 68–72 °C.

Note: Compound **5e** is a mixture of rotamers in $CDCl_3$ at room temperature (* minor rotamer).

^{1}H NMR (400 MHz, $CDCl_3$) δ: 5.08 – 4.94 (m, 2H, H-7), 4.40 – 4.12 (m, 2H, H-4, H-5a), 4.10 – 3.95 (m, 1H, H-5b), 3.31 (s, 1H, H-8), 2.72 – 2.54 (m, 1H, H-2a), 2.41 – 2.17 (m, 3H, H2-b, H-9), 1.99 (ddt, J = 16.2, 13.3, 2.8 Hz, 1H, H-3a), 1.83 – 1.70 (m, 1H, H-3b), 1.47 (s, 9H, H-12).

^{13}C NMR (101 MHz, $CDCl_3$) δ: (rotameric); 209.1 (C-1*), 208.9 (C-1), 155.1 (C-10), 154.9 (C-10*), 139.7 (C-6*), 139.6 (C-6), 114.1 (C-7), 113.8 (C-7*), 80.2 (C-11, C-11*), 51.3 (C-8*), 50.8 (C-8), 46.3 (C-4), 46.3 (C-5*), 45.5 (C-4*), 45.3 (C-5), 35.3 (C-2*), 34.9 (C-2), 31.4 (C-3), 31.1 (C-9), 30.6 (C-3*), 28.5 (C-12).

$[\alpha]_D^{25}$= +128.8 (c = 1.00, $CHCl_3$, 93% *ee*).

HPLC (Chiralpak AD-H, hexane/isopropanol 98:2, 1.0 mL min^{-1}, λ = 210 nm) t_R = 10.9 (major), 14.2 (minor).

benzyl (*1S,5R*)-4-methylene-6-oxo-2-azabicyclo[3.3.1]nonane-2-carboxylate ((+)-5f)

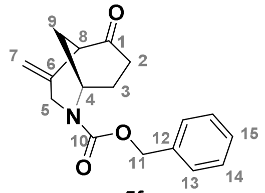

5f

Prepared according to General procedure B using **11f** (36.6 mg, 0.1 mmol). Purification by column chromatography (ethyl acetate:pentane = 3:7) afforded **(+)-5f** (28.4 mg, 99% yield, 96% *ee*) as a yellow oil. The spectroscopic data match those reported in the literature.[14]

Note: Compound **5f** is a 1:1 mixture of rotamers in $CDCl_3$ at room temperature.

^{1}H NMR (400 MHz, $CDCl_3$) δ: 7.44 – 7.29 (m, 5H, H-13, H-14, H-15), 5.26 – 5.11 (m, 2H, H-11), 5.11 – 4.98 (m, 2H, H-7), 4.52 – 4.27 (m, 2H, H-4, H-5a), 4.21 – 4.07 (m, 1H, H-5b), 3.35 (br s, 1H, H-8), 2.65 (dddd, J = 15.7, 13.2, 10.4, 7.3 Hz, 1H, H-2a), 2.50 – 2.19 (m, 3H, H-2b, H-3a, H-9a), 2.12 – 1.94 (m, 1H, H-9b), 1.81 (tddd, J = 13.5, 10.6, 5.7, 2.9 Hz, 1H, H-3b).

^{13}C NMR (101 MHz, $CDCl_3$) δ: 208.9 (C-1), 208.7 (C-1), 155.8 (C-10), 155.5 (C-10), 139.3 (C-6), 139.2 (C-6), 136.6 (C-12), 136.6 (C-12), 128.7 (C_{Ar}H), 128.3 (C_{Ar}H), 128.2 (C_{Ar}H), 128.1 (C_{Ar}H), 114.5 (C-7), 114.3 (C-7), 67.5 (C-11), 67.4 (C-11), 51.2 (C8), 50.9 (C-8), 46.4 (C-4), 46.3 (C-4), 46.1 (C-5), 46.0 (C-5), 35.2 (C-2), 34.9 (C-2), 31.5 (C-3), 31.2 (C-9), 31.0 (C-9), 30.5 (C-3).

$[\alpha]_D^{25}$= +253.5 (c = 1.00, $CHCl_3$, 96% *ee*).

HPLC (Chiralpak AD-H, hexane/isopropanol 98:2, 1.0 mL min^{-1}, λ = 210 nm) t_R = 26.6 (major), 43.8 (minor).

(*1S,5R*)-2-benzyl-4-methylene-2-azabicyclo[3.3.1]nonan-6-one ((–)-5g)

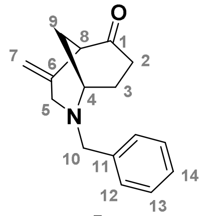

5g

Prepared according to General procedure B using **11g** (32.2 mg, 0.1 mmol). Purification by column chromatography (ethyl acetate:pentane = 1:3) afforded **(–)-5g** (18.3 mg, 76% yield, 98% *ee*) as a white solid.

MP: 48–50 °C.

IR (film) $\tilde{\nu}_{max}$/cm^{-1}: 2926, 1719, 1669, 1323, 1176, 1132, 703.

^{1}H NMR (400 MHz, $CDCl_3$) δ: 7.38 – 7.30 (m, 4H, H-12, H-13), 7.29 – 7.23 (m, 1H, H-14), 4.95 – 4.91 (m, 1H, H-7a), 4.88 – 4.83 (m, 1H, H-7b), 3.76 (q, *J* = 13.4 Hz, 2H, H-10), 3.36 (s, 2H, H-5), 3.26 (s, 1H, H-8), 3.10 (s, 1H, H-4), 2.61 (ddd, *J* = 17.1, 10.7, 8.5 Hz, 1H, H-2a), 2.52 -2.42 (m, 1H, H-2b), 2.36 – 2.22 (m, 2H, H-3a, H-9a), 1.99 (dt, J = 13.0, 3.0 Hz, 1H, H-9b), 1.81 – 1.68 (m, 1H, H-3b).

^{13}C NMR (101 MHz, $CDCl_3$) δ: 210.7 (C-1), 141.0 (C-11), 138.9 (C-6), 128.8 (2xC-Ar), 128.5 (2xC-Ar), 127.3 (C-14), 112.6 (C-7), 60.0 (C-10), 53.9 (C-5), 53.7 (C-8), 50.2 (C-4), 38.1 (C-2), 32.9 (C-9), 25.7 (C-3).

HRMS (ES+): Exact mass calculated for $[M+H]^+$ $(C_{16}H_{20}ON)^+$ requires 242.1539, found 242.1541.

$[\alpha]_D^{25}$= –16.3 (c = 0.37, $CHCl_3$, 98% *ee*).

HPLC (Chiralpak AD-H, hexane-isopropanol 98:2, 1.0 mL min^{-1}, λ = 210 nm) 9.0 (major), 10.8 (minor).

(*1S,5R*)-2-methyl-4-methylene-1-phenyl-2-azabicyclo[3.3.1]nonan-6-one ((+)-5h)

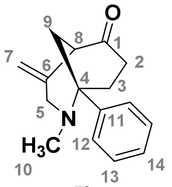

5h

Prepared according to General Procedure B using **11h** (32.2 mg, 0.1 mmol). Purification by column chromatography (ethyl acetate:pentane = 1:4) afforded **(+)-5h** (21.9 mg, 91% yield, 99% *ee*) as a pink oil.

IR (film) $\tilde{\nu}_{max}$/cm^{-1}: 2938, 2782, 1708, 1065, 1024.

^{1}H NMR (400 MHz, $CDCl_3$) δ: 7.52 – 7.47 (m, 2H, H-13), 7.36 – 7.30 (m, 2H, H-12), 7.27 – 7.21 (m, 1H, H-14), 4.99 (br s, 1H, H-7a), 4.97 (br s, 1H, H-7b), 3.76 – 3.63 (m, 2H, H-5), 3.31 (t, *J* = 3.0 Hz, 1H, H-8), 2.71 – 2.61 (m, 3H, H-2, H-3a), 2.48 – 2.37 (m, 1H, H-3b), 2.35 (dt, *J* = 13.3, 3.1 Hz, 1H, H-9a), 2.17 – 2.10 (m, 1H, H-9b), 2.11 (s, 3H, H-10).

^{13}C NMR (101 MHz, $CDCl_3$) δ: 210.1 (C-1), 146.5 (C-11), 141.1 (C-6), 128.4 (2xC-12), 127.0 (C-14), 126.5 (2xC-13), 111.9 (C-7), 57.1 (C-5), 57.0 (C-4), 55.0 (C-8), 42.2 (C-9), 38.8 (C-10), 38.4 (C-2), 29.0 (C-3).

HRMS (ES+): Exact mass calculated for $[M+H]^+$ $(C_{16}H_{20}ON)^+$ requires 242.1539, found 242.1540.

$[\alpha]_D^{25}$=+54.6 (c = 1.00, $CHCl_3$, 99% *ee*).

HPLC (Chiralpak OD-H, hexane/isopropanol 99:1, 1.0 mL min^{-1}, λ = 220 nm) t_R = 26.2 (major), 33.2 (minor).

(*1S,5R*)-2-benzyl-4-methylene-1-phenyl-2-azabicyclo[3.3.1]nonan-6-one ((+)-5i)

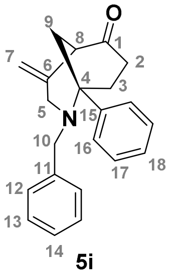

5i

Prepared according to General Procedure B using **11i** (39.8 mg, 0.1 mmol). Purification by column chromatography (ethyl acetate:pentane = 1:9) afforded **(+)-5i** (28.1 mg, 89% yield, 86% *ee*) as a colorless oil.

IR (film) $\tilde{\nu}_{max}$/cm^{-1}: 2938, 2361, 2341, 1711, 1494, 1446, 1137, 910, 762, 699.

^{1}H NMR (400 MHz, $CDCl_3$) δ: 7.69 – 7.61 (m, 2H, H-17), 7.38 (dd, J = 8.5, 7.0 Hz, 2H, H-14, H-18), 7.33 – 7.17 (m, 6H, H-12, H-13, H-16), 4.98 (br s, 1H, H-7a), 4.84 (s, 1H, H-7b), 3.63 (d, J = 15.1 Hz, 1H, H-10a), 3.62 (d, J = 13.6 Hz, 1H, H-5a), 3.55 (dt, J = 15.1, 2.0 Hz, 1H, H-10b), 3.35 (q, J = 2.8 Hz, 1H, H-8), 3.30 (d, J = 13.6 Hz, 1H, H-5b), 2.85 (ddd, J = 16.7, 11.0, 8.3 Hz, 1H, H-2a), 2.78 – 2.65 (m, 2H, H-2b, H-3a), 2.57 – 2.45 (m, 2H, h-3b, H-9a), 2.21 (dd, J = 13.4, 3.2 Hz, 1H, H-9b).

^{13}C NMR (101 MHz, $CDCl_3$) δ: 210.0 (C-1), 146.7 (C-15), 140.8 (C-6), 139.5 (C-11), 128.5 (2xC-Ar), 128.41 (2xC-Ar), 128.35 (2xC-Ar), 127.2 (C-14), 127.0 (C-18), 126.6 (2xC-Ar), 112.3 (C-7), 57.7 (C-4), 55.7 (C-5), 54.8 (C-8), 53.1 (C-10), 41.4 (C-9), 38.3 (C-2), 31.4 (C-3).

HRMS (ES+): Exact mass calculated for $[M+H]^+$ $(C_{22}H_{24}ON)^+$ requires 318.1852, found 318.1852.

$[\alpha]_D^{25}$=+70.3 (c = 0.040, $CHCl_3$, 86% *ee*).

HPLC (Chiralpak AD-H, hexane/isopropanol 98:2, 1.0 mL min^{-1}, λ = 254 nm) t_R = 9.7 (minor), 11.6 (major).

(*1S,5R*)-4-((*E*)-benzylidene)-2-tosyl-2-azabicyclo[3.3.1]nonan-6-one ((–)-5j)

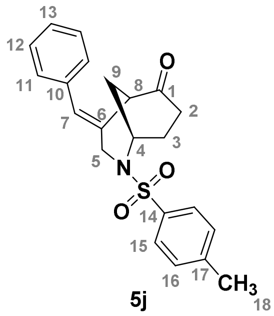

5j

Compound **11j** (46.2 mg, 0.1 mmol) was reacted according to general procedure B for desymmetrization except for tris(4-methoxyphenyl)phosphane (5.3 mg, 0.015 mmol, 15 mol%) was used instead of tris(4-trifluoromethylphenyl)phosphine and 48 h reaction time instead of 24 h. Purification by column chromatography (ethyl acetate:pentane = 1:4) afforded **5j** (29.2 mg, 77% yield, 95% *ee*) as a white solid.

MP: 100–102 °C.

IR (film) $\tilde{\nu}_{max}$/cm^{-1}: 2951, 1710, 1333, 1156, 1093, 1033.

^{1}H NMR (400 MHz, $CDCl_3$) δ: 7.79 – 7.75 (m, 2H, H-15), 7.40 – 7.32 (m, 6H, Ar-H), 7.29 – 7.23 (m, 1H, H-25), 6.59 (s, 1H, H-7), 4.28 – 4.07 (m, 3H, H-5, H-4), 3.74 (s, 1H, H-8), 2.72 (ddd, *J* = 16.0, 11.9, 7.8 Hz, 1H, H-2a), 2.45 (s, 3H, H-18), 2.44 – 2.36 (m, 1H, H-2b), 2.35 – 2.26 (m, 1H, H-3a), 2.03 – 1.85 (m, 3H, H-3b, H-9).

^{13}C NMR (101 MHz, $CDCl_3$) δ: 208.5 (C-1), 143.9 (C-14), 135.9 (C-17), 135.0 (C-6), 130.2 (C-7), 130.0 (2xC-Ar), 129.8 (C-9), 129.2 (2xC-Ar), 128.6 (2xC-Ar), 127.9 (C-13), 127.5 (2xC-15), 49.3 (C-5), 47.7 (C-4), 47.1 (C-8), 37.0 (C-2), 32.4 (C-9), 31.7 (C-3), 21.7 (C-18).

HRMS (ES+): Exact mass calculated for $[M+Na]^+$ $(C_{22}H_{23}O_3N^{23}Na^{32}S)^+$ requires 404.1291, found 404.1289.

$[\boldsymbol{\alpha}]_{\boldsymbol{D}}^{\mathbf{25}}$= -218.6 (c = 1.00, $CHCl_3$, 95% *ee*).

HPLC (Chiralpak IA, hexane/isopropanol 80:20, 1.0 mL min^{-1}, λ = 220 nm) t_R = 14.2 (minor), 17.9 (major).

(*1S,5R*)-4-((*E*)-4-methoxybenzylidene)-2-tosyl-2-azabicyclo[3.3.1]nonan-6-one ((–)-5k)

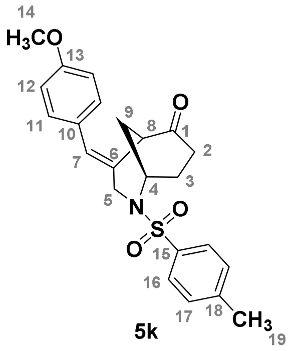

5k

Compound **11k** (49.2 mg, 0.1 mmol) was reacted according to general procedure B for desymmetrization except for tris(4-methoxyphenyl)phosphane (5.3 mg, 0.015 mmol, 15 mol%) was used instead of tris(4-trifluoromethylphenyl)phosphine and 48 h reaction time instead of 24 h. Purification by column chromatography (ethyl acetate:pentane = 1:4) to afforded **5k** (29.6 mg, 72% yield, 96% *ee*) as a white solid.

MP: 161–163 °C.

IR (film) $\tilde{\nu}_{max}$/cm^{-1}: 2921, 1704, 1608, 1515, 1459, 1316, 1303, 1252, 1147, 1097, 1033.

^{1}H NMR (400 MHz, CDCl$_3$) δ: 7.81 – 7.74 (m, 2H, H-16), 7.38 – 7.32 (m, 4H, H-11,H-17), 6.91 – 6.85 (m, 2H, H-12), 6.52 (s, 1H, H-10), 4.26 – 4.17 (m, 2H, H-4, H-5a), 4.16 – 4.09 (m, 1H, H-5b), 3.80 (s, 3H, H-14), 3.73 (s, 1H, H-8), 2.80 – 2.69 (m, 1H, H-2), 2.44 (s, 3H, H-19), 2.42 – 2.27 (m, 2H, H-2b, H-3a), 2.02 – 1.92 (m, 2H, H-9a, H-3b), 1.88 (dt, *J* = 13.8, 2.9 Hz, 1H, H-9b).

^{13}C NMR (101 MHz, CDCl$_3$) δ: 208.8 (C-1), 159.3 (C-13), 143.9 (C-15), 135.8 (C-18), 130.7 (C-17), 130.0 (C-11), 129.9 (C-7), 127.9 (C-Ar), 127.54 (C-Ar), 127.48 (C-16), 114.0 (C-12), 55.4 (C-14), 49.4 (C-5), 47.8 (C-4), 47.3 (C-8), 36.9 (C-2), 32.4 (C-9), 32.1 (C-3), 21.7 (C-19).

HRMS (ES+): Exact mass calculated for $[M+Na]^+$ $(C_{23}H_{25}O_4N^{23}Na^{32}S)^+$ requires 434.1397, found 434.1395.

$[\alpha]_D^{25}$= -223.1 (c = 1.00, CHCl$_3$, 96% *ee*).

HPLC (Chiralpak IA, hexane/isopropanol 80:20, 1.0 mL min^{-1}, λ = 220 nm) t_R = 20.2 (minor), 21.3 (major).

(*1S,5R,E*)-4-butylidene-2-tosyl-2-azabicyclo[3.3.1]nonan-6-one ((–)-5l)

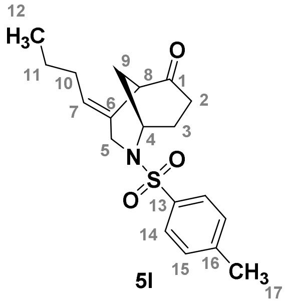

5l

Compound **11l** (42.8 mg, 0.1 mmol) was reacted according to general procedure B for desymmetrization except with triphenylphosphine (3.9 mg, 0.015 mmol, 15 mol%) instead of tris(4-trifluoromethylphenyl)phosphine, and purified by column chromatography (diethyl ether:pentane = 3:7 to 3:2) to afford compound **5l** (20.3 mg, 59% yield, 95% *ee*) as a white solid.

MP: 102–104 °C.

IR (film) $\tilde{\nu}_{max}$/cm^{-1}: 2959, 2927, 1712, 1455, 1339, 1161, 1095, 932, 662.

^{1}H NMR (400 MHz, $CDCl_3$) δ: 7.72 (d, J = 8.5 Hz, 2H, H-14), 7.33 (d, J = 8.5 Hz, 2H, H-15), 5.55 (tt, J = 8.0, 2.0 Hz, 1H, H-7), 4.10 (d, J = 13.5, 1H, H-5a), 4.03 – 3.98 (m, 1H, H-4), 3.65 – 3.58 (m, 1H, H-5b), 3.35 (s, 1H, H-8), 2.94 (ddd, J = 15.0, 13.5, 7.0 Hz, 1H, H-2a), 2.43 (s, 3H, H-17), 2.43 – 2.36 (m, 1H, H-3a), 2.23 – 2.15 (m, 1H, H-2b), 2.02 – 1.90 (m, 3H, H-10, H-9a), 1.87 – 1.77 (m, 2H, H-3b, H-9b), 1.35 – 1.23 (m, 2H, H-11), 0.84 (t, J = 7.5 Hz, 3H, H-12).

^{13}C NMR (101 MHz, $CDCl_3$) δ: 208.9 (C-1), 143.8 (C-16), 134.5 (C-13), 132.4 (C-7), 130.0 (2xC-15), 128.6 (C-6), 127.6 (2xC-14), 48.8 (C-4), 48.2 (C-5), 47.0 (C-8), 34.6 (C-2), 33.9 (C-3), 30.7 (C-9), 29.4 (C-10), 22.2 (C-11), 21.7 (C-17), 13.8 (C-12).

HRMS (ES+): Exact mass calculated for[M+H]$^+$ ($C_{19}H_{26}O_3N^{32}S$)$^+$ requires 348.1628, found 348.1627.

$[\alpha]_D^{25}$= -15.6 (c = 1.00, $CHCl_3$).

HPLC (Chiralcel OD, hexane/isopropanol 90:10, 1.0 mL min^{-1}, λ = 210 nm) t_R = 10.2 (minor), 12.0 (major).

3.4 Synthesis of Daphniphyllum alkaloids

3.4.1 Synthesis and functionalization of the morphan core

Scheme S8. Synthesis of enantiomerically enriched and racemic bicyclic amine **4**.

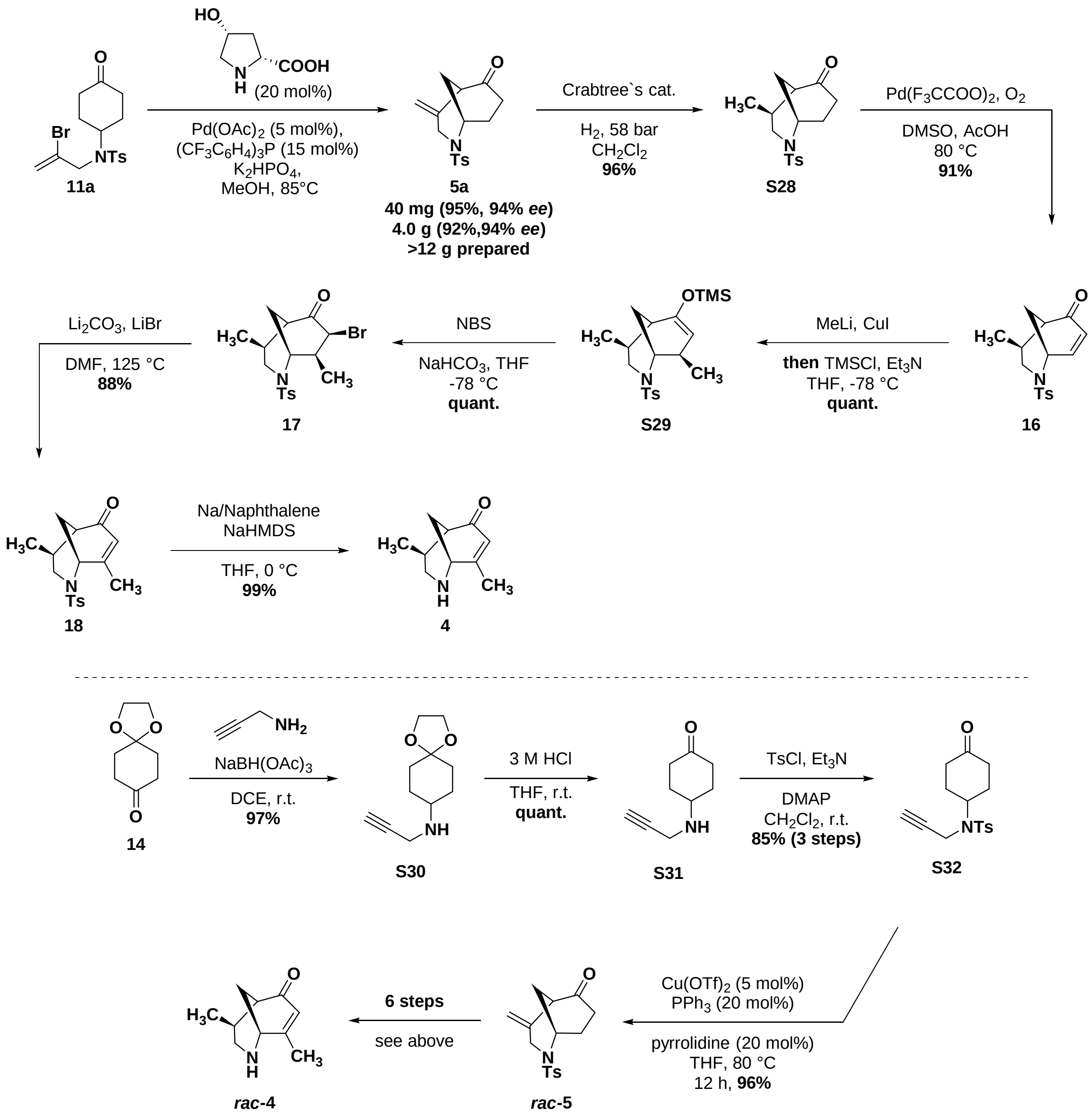

(*1S,5R*)-4-methylene-2-tosyl-2-azabicyclo[3.3.1]nonan-6-one (5a)

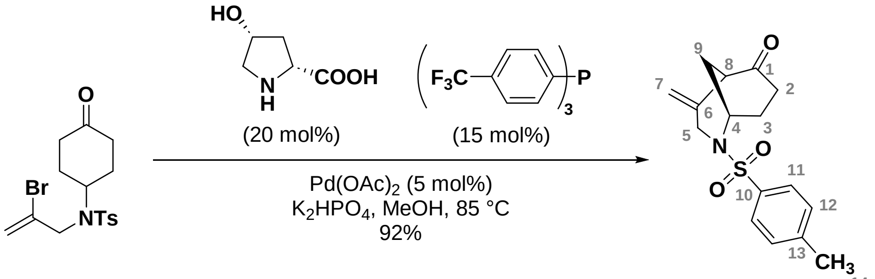

11a 5a

Oven-dried thick-wall Schlenk tube under the argon atmosphere was charged with ketone **11a** (4.02 g, 10.4 mmol, 1 equiv.), palladium acetate (116.8 mg, 0.520 mmol, 5.00 mol %), (*2R,4R*)-4-hydroxypyrrolidine-2-carboxylic acid (273 mg, 2.08 mmol, 0.20 equiv.), tris(4-trifluoromethylphenyl)phosphine (728 mg, 1.56 mmol, 0.15 equiv.) and K_2HPO_4 (2.72 g, 15.61 mmol, 1.50 equiv.). The reaction vessel was then degassed by 5 cycles of vacuum (oil pump) – argon. Methanol (200 mL), degassed by bubbling with nitrogen (1 h) and argon (1 h) prior to use, was added, and another 5 cycles of vacuum – argon were repeated. Then, the reaction vessel was sealed and submerged into a pre-heated oil bath (85 °C) and stirred at this temperature for 24 h. The reaction mixture was then allowed to cool down to room temperature, diluted with CH_2Cl_2 (200 mL), filtered through Celite® in a column by the pressure of nitrogen and washed out with additional CH_2Cl_2 (100 mL). Collected filtrate was concentrated under reduced pressure, combined with material from several other runs (total 16.9 g of starting material was used) and purified by gradient column chromatography (ethyl acetate/pentane = 0:1 to 6:4). The desired product of cyclization was obtained as a white solid (12.36 g, 92%, 94% *ee*).

Note: The product of the cyclization contained a trace amount of phosphine oxide that has a very similar R_f to the product. In order to obtain phosphine residues-free material, the product obtained after column chromatography was triturated from Et_2O (25 + 5 + 5 mL). The combined ether fraction concentrated and trituration repeated. This method provided 11.5 g of phosphine-free material (93% yield of trituration, 86% overall yield).

N-(prop-2-yn-1-yl)-1,4-dioxaspiro[4.5]decan-8-amine (S30)

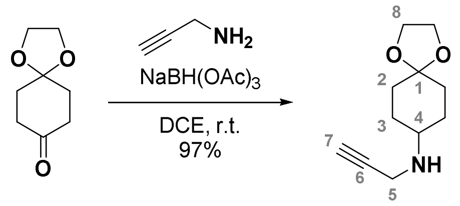

14 S30

Compound **S30** was prepared according to a literature procedure.[14] To a solution of ketone **14** (20.0 g, 128 mmol, 1 equiv.) in DCE (400 mL) was added propargyl amine (8.26 mL, 132 mmol, 1.03 equiv.) and after stirring for 5 min at room temperature, the reaction mixture was cooled to 0 °C. $NaBH(OAc)_3$ (38.0 g, 179 mmol, 1.40 equiv.) was then added in a small portion over 30 min and the resulting suspension was stirred at room temperature for 22 h. The reaction mixture was then quenched with 1 M KOH (400 mL) and extracted with CH_2Cl_2 (3 x 200 mL). The combined organic phases were dried over anhydrous Na_2SO_4, filtered and concentrated under reduced pressure. The amine **S30** was obtained as a yellowish solid (24.2 g, 97%) and was used in the next step without further purification. Experimental data are in agreement with literature values.[14]

^{1}H NMR (400 MHz, $CDCl_3$) δ: 3.91 (s, 4H, H-8), 3.43 (d, J = 2.5 Hz, 2H, H-5), 2.75 (tt, J = 9.6, 3.7 Hz, 1H, H-4), 2.18 (t, J = 2.4 Hz, 1H, H-7), 1.89 – 1.70 (m, 4H, H-2a, H-3a), 1.55 (td, J = 12.5, 3.8 Hz, 2H, H-2b), 1.42 (tdd, J = 12.1, 9.5, 3.2 Hz, 2H, H-3b), 1.24 (br s, 1H, NH).

^{13}C NMR (101 MHz, $CDCl_3$) δ: 108.7 (C-1), 82.6 (C-6), 71.2 (C-7), 64.38 (C-8), 64.36 (C-8), 53.4 (C-4), 35.6 (C-5), 32.8 (C-2), 29.9 (C-3).

4-(prop-2-yn-1-ylamino)cyclohexan-1-one (S31)

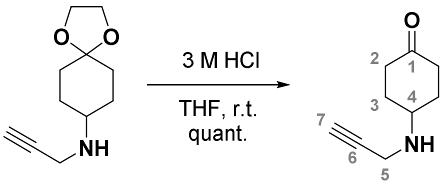

Compound **S31** was prepared according to a literature procedure.[14] To a solution of acetal **S30** (24.2 g, 124 mmol, 1 equiv.) in THF (320 mL) was added 3 M HCl (320 mL), and the resulting solution was stirred for 2 days at room temperature. The reaction mixture was then quenched with 3 M KOH (350 mL), phases separated, and the aqueous phase extracted with *i*-PrOH/$CHCl_3$ (1:5) (3 x 500 mL). The combined organic phases were dried over anhydrous Na_2SO_4, filtered and concentrated under reduced pressure. Ketone **S31** was obtained as a brown oil (18.7 g, quant.) and was used in the next step without further purification. Experimental data are in agreement with literature values.[14]

^{1}H NMR (400 MHz, $CDCl_3$) δ: 3.49 (d, J = 2.4 Hz, 2H, H-5), 3.21 (tt, J = 8.2, 3.5 Hz, 1H, H-4), 2.56 – 2.41 (m, 2H, H-2a), 2.39 – 2.26 (m, 2H, H-2b), 2.24 (t, J = 2.4 Hz, 1H, H-7), 2.12 – 1.99 (m, 2H, H-3a), 1.77 – 1.63 (m, 2H, H-3b).

^{13}C NMR (101 MHz, $CDCl_3$) δ: 211.3 (C-1), 82.0 (6), 71.7 (C-7), 51.6 (C-4), 38.3 (C-2), 35.7 (C-5), 31.6 (C-3).

4-methyl-*N*-(4-oxocyclohexyl)-*N*-(prop-2-yn-1-yl)benzenesulfonamide (S32)

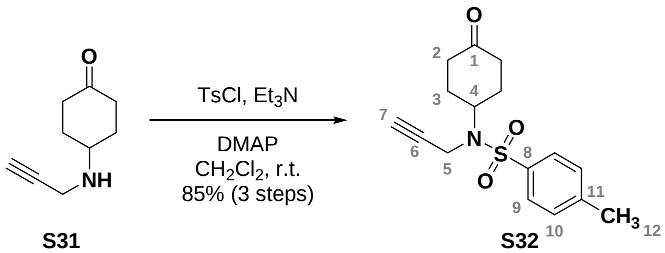

Compound **S32** was prepared according to a literature procedure.[14] To a solution of amine **S31** (18.7 g, 124 mmol, 1 equiv.) in CH_2Cl_2 (400 mL) was added Et_3N (20.3 mL, 146 mmol, 1.18 equiv.) followed by TsCl (27.8 g, 146 mmol, 1.18 equiv.) and DMAP (1.48 g, 12.1 mmol, 0.10 equiv.). After stirring for 24 h, the reaction mixture was diluted with CH_2Cl_2 (300 mL) and washed with 1 M HCl (100 mL), water (400 mL) and brine (300 mL). The organic phase was dried over anhydrous Na_2SO_4, filtered and concentrated under reduced pressure. Purification by column chromatography (ethyl acetate:pentane = 30:70) afforded product **S32** as a white solid (33.3 g, 88%, 85% over 3 steps). Experimental data are in agreement with literature values.[14]

^{1}H NMR (400 MHz, $CDCl_3$) δ: 7.84 – 7.75 (m, 2H, H-9), 7.33 – 7.23 (m, 2H, H-10), 4.19 (tt, J = 11.4, 4.6 Hz, 1H, H-4), 4.10 (d, J = 2.5 Hz, 2H, H-5), 2.42 (s, 3H, H-12), 2.45 – 2.34 (m, 4H, H-2), 2.17 (t, J = 2.5 Hz, 1H, H-7), 2.11 – 1.92 (m, 4H, H-3).

^{13}C NMR (101 MHz, $CDCl_3$) δ: 208.6 (C-1), 143.8 (C-8), 137.6 (C-11), 129.8 (C-10), 127.4 (C-9), 79.8 (C-6), 73.1 (C-7), 55.7 (C-C), 40.0 (C-2), 32.6 (C-5), 30.1 (C-3), 21.7 (C-12).

***rac*-(*1S,5R*)-4-methylene-2-tosyl-2-azabicyclo[3.3.1]nonan-6-one (*rac*-5a)**

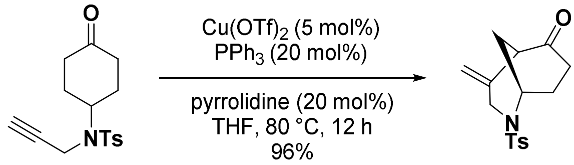

S32 *(rac)*-**5a**

Compound **5a** was prepared according to a literature procedure.[14] An oven-dried round-bottom flask was changed with alkyne **S32** (11.7 g, 38.3 mmol, 1 equiv.), $Cu(OTf)_2$ (693 mg, 1.92 mmol, 5.00 mol %) and PPh_3 (2.01 g, 7.66 mmol, 0.20 equiv.) and degassed using three vacuum – N_2 cycles. Degassed THF (250 mL) was then added, followed by pyrrolidine (630 µL, 7.66 mmol, 0.20 equiv.). The reaction vessel was sealed and heated to 90 °C for 18 h. The reaction mixture was then concentrated under reduced pressure and purified by column chromatography (ethyl acetate:pentane = 20:80). The product of cyclization ***(rac)*-5a** was obtained as a white solid (11.2g, 96%). Experimental data are in agreement with literature values.[14]

Optimization of hydrogenation

Table S2. Catalyst screening.

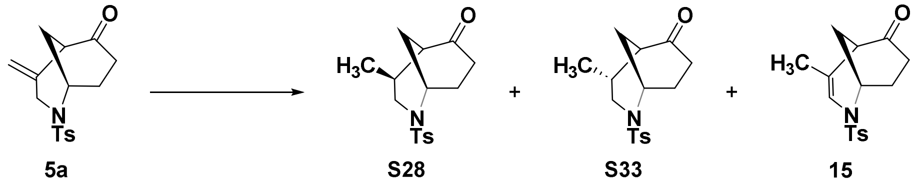

Entry	Catalyst	Pressure (bar)	Hydrog. (%)	d.r. (S28:S33)	15 (%)
1[a]	Pd/C (10 mol %)	1	15	1:3.0	40
2	$Rh(PPh_3)_3Cl$ (5.0 mol %)	1	38	1:2.3	14
3	$[Rh(dppb)(nbd)]ClO_4$ (5.0 mol%)	1	20	1:2.5	2
4	$[Rh(dppb)(nbd)]ClO_4$ (10 mol%)	1	14	1:5.2	<1
5	$[Ir(cod)(PPh_3)py]PF_6$ (20 mol%)	1	18	>20:1	82
**6**	**$[Ir(cod)(PPh_3)py]PF_6$ (5.0 mol%)**	**9**	**86**	**>20:1**	**14**
7	$Mn(dpm)_3$ (10 mol%), $PhSiH_3$ (1.0 equiv), TBHP (1.5 equiv), *i*-PrOH[22]	-	68	<1:20	<1
8	$Rh(cod)Cl_2$ (5.0 mol %), PPh_3(10 mol%), $AgBF_4$ (15 mol %)	1	74	>20:1	24
9	$Rh(cod)Cl_2$ (2.5 mol %), PPh_3(5.0 mol%), $AgBF_4$ (7.5 mol %)	1	69	>20:1	20
10	$Rh(cod)Cl_2$ (5.0 mol %), PPh_3(10 mol%), $AgBF_4$ (15 mol%)	9	53	5:1	2

Reagents and conditions: Catalyst, H_2 (1 bar), CH_2Cl_2, r.t., 5–16 h. [a]EtOAc used as a solvent. Yields were determined by analysis of ^1H NMR spectra of crude reaction mixtures.

Table S3. Effect of catalyst loading and pressure on the directed hydrogenation using Crabtree`s catalyst.

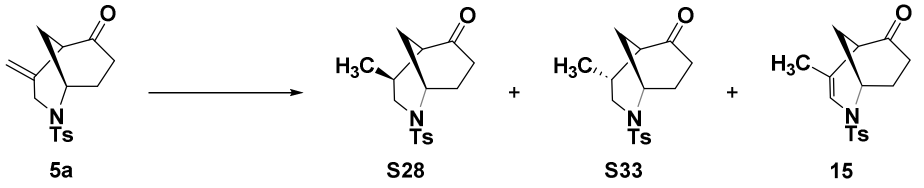

Entry	Cat. loading (mol %)	Pressure (bar)	Conc. ($mg.mL^{-1}$)	Temp. (°C)	Hydrog. (%)	d.r. (S28:S33)	15 (%)
1	20.0	1	20	r.t.	18	>20:1	82
2	5.0	1	20	r.t.	56	>20:1	44
3	5.0	5	20	r.t.	60	>20:1	40
4	5.0	9	20	r.t.	86	>20:1	14
5	5.0	5	20	40	20	n.d.	80
6	5.0	5	20	0	0	n/a	0
7	5.0	9	30	r.t.	86	>20:1	14
8	5.0	9	10	r.t.	79	>20:1	21
9[a]	5.0	9	20	r.t.	83	>20:1	17
**10[a]**	**1.5**	**58**	**20**	**r.t.**	**96**	**>20:1**	**4**

Reagents and conditions: $[Ir(cod)(PCy_3)py]PF_6$, H_2, CH_2Cl_2, 4 h; [a] Reaction time 30 min.

(*1S,4S,5R*)-4-methyl-2-tosyl-2-azabicyclo[3.3.1]nonan-6-one (S28)

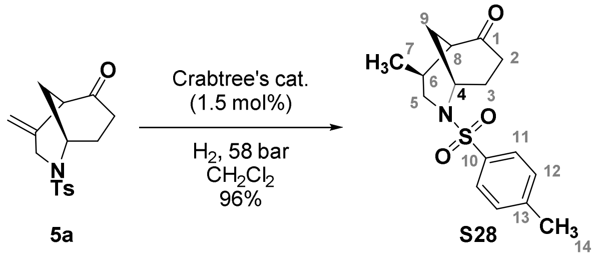

A high-pressure steel reactor was charged with alkene **5a** (3.00 g, 9.82 mmol, 1 equiv.), Crabtree's catalyst (119 mg, 0.147 mmol, 1.5 mol%) and CH_2Cl_2 (150 mL). While the reaction mixture was stirred vigorously, the reactor was pressurized with hydrogen (58 bar) and stirred at room temperature for 30 min. Then, the pressure was carefully released, and the reaction mixture was concentrated under reduced pressure. Purification of material obtained from several runs (total 11.0 g of starting material was used) by gradient column chromatography (ethyl acetate:pentane = 1:9 to 4:6) afforded desired product of reduction **S28** as a white solid (10.58 g, 96%).

Note: The purity of the starting material is crucial for achieving good reactivity and diastereoselectivity. Traces of phosphine ligand (used in the synthesis of the starting material) leads to poisoning of the hydrogenation catalyst and lower *d.r.* and yield.

MP: 86–88 °C (>99% ee), 98–100 °C (rac).

IR (thin film) $\tilde{\nu}_{max}$/cm^{-1}: 2961, 2928, 1707, 1331, 1162, 1093.

^{1}H NMR (400 MHz, $CDCl_3$) δ: 7.73 – 7.64 (m, 2H, H-11), 7.35 – 7.27 (m, 2H, H-12), 4.20 – 4.12 (m, 1H, H-4), 3.26 (dd, J = 12.5, 5.0 Hz, 1H, H-5a), 2.99 (dd, J = 12.5, 5.8 Hz, 1H, H-5b), 2.59 (dt, J = 16.7, 8.2 Hz, 1H, H-2a), 2.43 (s, 3H, H-14), 2.34 (dddd, J = 17.0, 7.9, 5.2, 1.0 Hz, 1H, H-2b), 2.23 (q, J = 3.0 Hz, 1H, H-8), 2.19 – 2.05 (m, 2H, H-6, H-9a), 2.00 – 1.83 (m, 2H, H-3), 1.74 (dt, J = 13.9, 3.0 Hz, 1H, H-9b), 1.07 (d, J = 6.9 Hz, 3H, H-7).

^{13}C NMR (101 MHz, $CDCl_3$) δ: 213.4 (C-1), 143.6 (C-10), 136.0 (C-13), 129.9 (C-12), 127.3 (C-11), 48.3 (C-8), 47.3 (C-4), 45.5 (C-5), 35.7 (C-2), 32.0 (C-6), 28.2 (C-3), 26.1 (C-9), 21.6 (C-14), 18.6 (C-7).

HRMS (ES+): exact mass calculated for $[M+H]^+$ $(C_{16}H_{22}O_3N^{32}S)^+$ requires 308.1315, found 308.1316.

$[\alpha]_D^{25}$= +78.9 (c = 1.00, $CHCl_3$, >99% ee).

***rac*-(*1S,5R*)-4-methyl-2-tosyl-2-azabicyclo[3.3.1]non-3-en-6-one (15)**

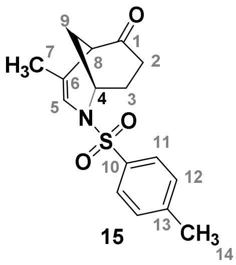

15

Enamide **15** was isolated as a side-product in the hydrogenation of **5a**, when the reaction was performed at lower pressure of hydrogen and/or the starting material was contaminated with traces of phosphine.

MP: 120–122 °C (racemic).

IR (thin film) ṽ$_{max}$/cm^{-1}: 2959, 2933, 1709, 1341, 1162, 1094.

^{1}H NMR (500 MHz, $CDCl_3$) δ: 7.69 (d, J = 8.0 Hz, 2H, H-11), 7.31 (d, J = 8.0 Hz, 2H, H-12), 6.82 (d, J = 1.5 Hz, 1H, H-5), 4.11–4.15 (m, 1H, H-4), 2.70–2.75 (m, 1H, H-8), 2.57 (ddd, J = 15.5, 13.5, 7.5 Hz, 1H, H-2a), 2.41 (s, 3H, H-14), 2.37 (dddd, J = 14.0, 7.5, 3.5, 1.5 Hz, 1H, H-3a), 2.15 (dd, J = 15.7, 6.0 Hz, 1H, H-2b), 1.85—1.92 (m, 1H, H-3b), 1.83 (dt, J = 13.0, 2.5, 2.5 Hz, 1H, H-9a), 1.65 (d, 3H, J = 1.5 Hz, H-7), 1.27–1.34 (m, 1H, H-9b).

^{13}C NMR (126 MHz, $CDCl_3$) δ: 208.3 (C-1), 144.0 (C-10), 135.9 (C-13), 130.0 (C-12), 126.9 (C-11), 122.9 (C-5), 113.7 (C-6), 49.8 (C-8), 47.7 (C-4), 34.6 (C-3), 33.7 (C-2), 27.8 (C-9), 21.7 (C-14), 19.5 (C-7).

HRMS (ES+): exact mass calculated for $[M+H]^+$ $(C_{16}H_{20}O_2N^{32}S)^+$ requires 306.1158, found 306.1159.

***rac*-(*1S,4R,5R*)-4-methyl-2-tosyl-2-azabicyclo[3.3.1]nonan-6-one (S33)**

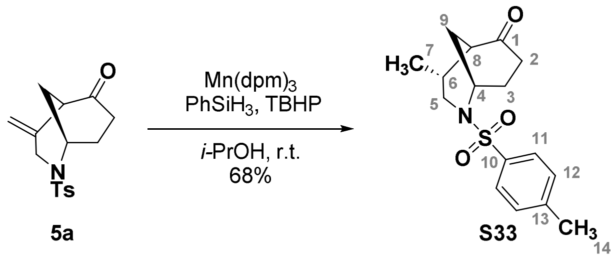

Compound **S33** was prepared adopting the procedure published by Shevni[22] To a solution of alkene **5a** (30.5 mg, 0.10 mmol, 1 equiv.) in *i*-PrOH (0.20 mL) at room temperature under Ar was added $PhSiH_3$ (12 µL, 0.10 mmol, 1.00 equiv.) and *tert*-butyl hydroperoxide (27.7 µL, 0.15 mmol, 1.50 equiv.) followed by $Mn(dpm)_3$ (6.1 mg, 0.01 mmol, 0.10 equiv.). The reaction was stirred at room temperature for 16 h until complete before concentrating under a vacuum. Purification by gradient column chromatography (ethyl acetate:pentane = 20% to 40%) afforded **S33** (21.0 mg, 68%, >20:1 dr) as a white solid.

MP: 88 – 91 °C (*rac*).

IR (thin film) $\tilde{\nu}_{max}$/cm^{-1}: 2930, 1703, 1339, 1190, 1177, 1096.

^{1}H NMR (400 MHz, $CDCl_3$) δ: 7.72 (dq, J = 8.5, 2.0 Hz, 2H, 11), 7.35 – 7.29 (m, 2H, 12), 4.34 – 4.28 (m, 1H, 4), 3.84 (dd, J = 13.5, 6.0 Hz, 1H, 5a), 2.87 – 2.78 (m, 1H, 5b), 2.49 – 2.43 (m, 5H, 2a, 8, 14), 2.12 (ddd, J = 18.5, 11.0, 8.5 Hz, 1H, 2b), 2.05 – 1.86 (m, 5H, 3, 6, 9), 0.86 (d, J = 7.0 Hz, 2H, 7);

^{13}C NMR (101 MHz, $CDCl_3$) C: 211.1 (C-1), 143.6 (C-10), 137.4 (C-13), 130.0 (C-12), 127.2 (C-11), 49.1 (C-8), 47.4 (C-5), 46.1 (C-4), 39.3 (C-2), 32.9 (C-6), 32.8 (C-3), 28.7 (C-9), 21.7 (C-14), 16.9 (C-7).

HRMS (ES+): exact mass calculated for $[M+Na]^+$ $(C_{16}H_{21}O_3N^{32}SNa)^+$ requires 330.1134, found 330.1140.

(*1R,4S,5R*)-4-methyl-2-tosyl-2-azabicyclo[3.3.1]non-7-en-6-one (16)

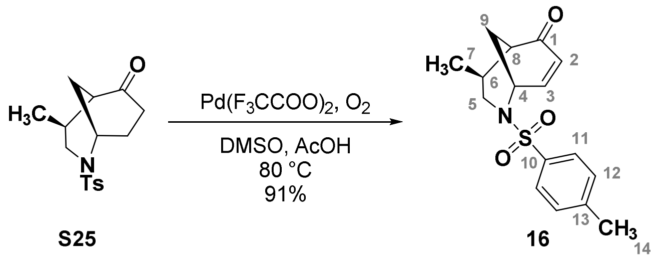

Compound **16** was prepared adopting the procedure published by Stahl[23] To a stirred solution of ketone **S28** (16.7 g, 54.7 mmol, 1 equiv.) and Pd(TFA)$_2$ (727 mg, 2.19 mmol, 4.0 mol %) in acetic acid (164 mL) was added DMSO (311 µL, 2.75 mmol, 8.0 mol %), reflux condenser was attached, and the system was flushed with oxygen. The reaction mixture was heated to 80 °C for 2-3 days under the atmosphere of oxygen introduced from a double-coated balloon attached to the top of the condenser. After completion (monitored by NMR), the reaction mixture was allowed to cool to room temperature and concentrated under reduced pressure. The brown residue was purified by gradient column chromatography (ethyl acetate:pentane = 1:9 to 3:7). Enone **16** was isolated as a white solid (15.1 g, 91%).

MP: 124–126 °C (*rac*), 115–118 °C (>99% *ee*).

IR (thin film) $\tilde{\nu}_{max}$/cm^{-1}: 2967, 2927, 2873, 1678, 1342, 1163, 1091.

^{1}H NMR (400 MHz, CDCl$_3$) δ: 7.74 – 7.64 (m, 2H, H-11), 7.35 – 7.26 (m, 2H, H-12), 6.31 (ddd, J = 9.9, 6.1, 1.7 Hz, 1H, H-3), 6.07 (dd, J = 9.9, 1.1 Hz, 1H, H-2), 4.64 (dt, J = 6.4, 3.2 Hz, 1H, H-4), 3.42 (ddd, J = 12.7, 1.5, 0.8 Hz, 1H, H-5a), 3.02 (dd, J = 12.8, 4.0 Hz, 1H, H-5b), 2.43 (s, 3H, H-14), 2.37 (d, J = 3.0 Hz, 1H, H-8), 2.28 (dtd, J = 13.4, 3.0, 1.7 Hz, 1H, H-9a), 2.07 – 1.96 (m, 2H, H-6, H-9b), 1.18 (d, J = 7.0 Hz, 3H, H-7).

^{13}C NMR (101 MHz, CDCl$_3$) δ: 201.3 (C-1), 143.9 (C-10), 140.8 (C-3), 136.7 (C-13), 133.0 (C-2), 130.0 (C-12), 127.3 (C-11), 47.1 (C4), 46.7 (C-8), 43.9 (C-5), 31.4 (C-6), 27.7 (C-9), 21.7 (C-14), 17.5 (C-7).

HRMS (ES+): exact mass calculated for $[M+H]^+$ $(C_{16}H_{20}O_2N^{32}S)^+$ requires 306.1158, found 306.1159.

$[\alpha]_D^{25}$= +382.04 (c = 1.00, CHCl$_3$).

(*1R,4S,5R,8R*)-4,8-dimethyl-2-tosyl-6-((trimethylsilyl)oxy)-2-azabicyclo[3.3.1]non-6-ene (S29)

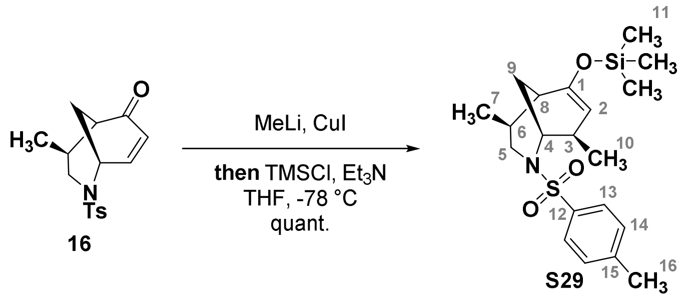

To a suspension of CuI (6.18 g, 32.5 mmol, 1.10 equiv.) in dry THF (150 mL) was dropwise added MeLi (1.6 M in Et_2O, 38.7 mL, 62.0 mmol, 2.10 equiv.) at 0 °C. During the addition of MeLi the suspension gradually turned from white to dark yellow and then brown and, after the complete addition of MeLi became a colorless solution. The resulting solution was stirred for 15 min at 0 °C before cooling to -78 °C. Then, a solution of ketone **16** (9.02 g, 29.5 mmol, 1 equiv.) in dry THF (70 mL) was added dropwise, followed by the addition of Et_3N (16.5 mL, 118.1 mmol, 4.00 equiv.) and TMSCl (11.2 mL, 88.6 mmol, 3.00 equiv.). (**Note:** *The solution becomes yellow on the addition of enone* ***16****, and darkens on the addition of TMSC*l). After 30 minutes, the reaction mixture was diluted with Et_2O (500 mL) and transferred to a separation funnel. The reaction mixture, while still cold, was washed with sat. aq. solution of NH_4Cl (5 x 100 mL) until the aq. phase was no longer blue. The combined aq. phases were back-extracted with Et_2O (200 mL), and combined organic phases were dried over Na_2SO_4, filtered and concentrated under reduced pressure. Silyl enol ether **S29** was obtained as a colorless oil (11.6 g, quant.) and was used in the next step without further purification.

IR (thin film) $\tilde{\nu}_{max}$/cm^{-1}: 3016, 2957, 1661, 1332, 1252, 1194, 1166, 1099, 1011.

^{1}H NMR (400 MHz, C_6D_6) δ: 7.76 (d, J = 8.0 Hz, 2H, H-13), 6.83 (d, J = 8.0 Hz, 2H, H-14), 4.54 (d, J = 4.0 Hz, 1H, H-2), 4.01 (d, J = 3.5 Hz, 1H, H-4), 3.46 (d, J = 12.0, 1H, H-5), 3.28 (dd, J = 12.5, 3.5 Hz, 1H, H-5), 1.95 (qd, J = 7.5, 4.0 Hz, 1H, H-3), 1.90 (s, 3H, H-16), 1.77–1.84 (m, 3H, H-6, H-8, H-9a), 1.36 (ddd, J = 13.5, 5.0, 3.5 Hz, 3H, H-9b), 0.97 (d, J = 7.0 Hz, 3H, H-7), 0.78 (d, J = 7.0 Hz, 3H, H-10), 0.06 (s, 9H, H-11).

^{13}C NMR (101 MHz, C_6D_6) δ: 152.9 (C-1), 142.5 (C-12), 139.5 (C-15), 129.7 (C-14), 127.4 (C-13), 108.2 (C-2), 53.3 (C-4), 44.5 (C-5), 39.4 (C-8), 33.7 (C-3), 29.9 (C-6), 22.4 (C-9), 21.3 (C-10), 21.2 (C-16), 17.1 (C-7), 0.3 (C-11).

^{1}H NMR (400 MHz, $CDCl_3$) δ: 7.72 – 7.62 (m, 2H, H-13), 7.31 – 7.24 (m, 2H, H-14), 4.65 (d, J = 3.9 Hz, 1H, H-2), 3.81 (t, J = 3.5 Hz, 1H, H-4), 3.32 (dd, J = 12.2, 1.4 Hz, 1H, H-5a), 3.20 (dd, J = 12.2, 3.7 Hz, 1H,

H-5b), 2.42 (s, 3H, H-16), 1.97 – 1.89 (m, 1H, H-9a), 1.89 – 1.77 (m, 3H, H-3, H-8, H-6), 1.53 (dt, J = 13.3, 3.0 Hz, 1H, H-9b), 1.09 (d, J = 6.8 Hz, 3H, H-7), 0.88 (d, *J* = 7.2 Hz, 3H, H-10), 0.15 (s, 9H, H-11).

^{13}C NMR (101 MHz, $CDCl_3$) δ: 152.5 (C-1), 143.0 (C-12), 138.2 (C-15), 129.7 (C-14), 127.0 (C-13), 108.8 (C-2), 53.2 (C-4), 44.3 (C-5), 39.0 (C-8), 33.3 (C-3), 29.5 (C-6), 22.3 (C-9), 21.7 (C-10), 21.2 (C-16), 17.1 (C-7), 0.05 (C-11).

HRMS (ES+): exact mass calculated for $[M+H]^+$ $(C_{20}H_{32}O_3N^{32}S^{28}Si)^+$ requires 394.1867, found 394.1864.

$[\alpha]_D^{25}$= +10.4 (c = 1.00, C_6H_6).

***rac*-(*1R,4S,5R,8R*)-4,8-dimethyl-2-tosyl-2-azabicyclo[3.3.1]nonan-6-one (S34)**

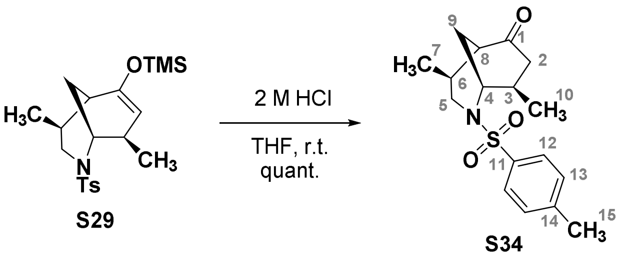

Ketone **S34** was prepared from enone **16** (5.11 g, 16.7 mmol) by *in situ* hydrolysis of silyl enol ether **S29**. After the formation of **S29** was complete (by TLC), the reaction was warmed to room temperature, and aqueous HCl (2 M) was added. The solution was stirred until the formation of methyl ketone **S34** was complete (as judged by TLC). The reaction was then diluted with Et_2O and washed with sat. aq. NH_4Cl soln. until the aq. phase was no longer blue. The organic layer was dried of $MgSO_4$ and concentrated in vacuo to afford ketone **S34** as a white solid (5.37 g, quant.).

MP: 113 – 116 °C (*rac*).

IR (thin film) $\tilde{\nu}_{max}$/cm^{-1}: 2960, 2925, 1708, 1330, 1155, 1092.

^{1}H NMR (400 MHz, C_6D_6) δ: 7.66 (d, J = 8.0 Hz, 2H, H-12), 6.83–6.87 (m, 2H, H-13), 3.84 (tt, J = 3.0, 1.5 Hz, 1H, H-4), 3.06 (dd, J = 13.0, 4.0 Hz, 1H, 5), 2.84 (dd, J = 13.0, 4.0 Hz, 1H, H-5), 2.18 (dd, J = 16.0, 7.0 Hz, 1H, H-2), 1.89–2.01 (m, 1H, H-6), 1.94 (s, 3H, H-15), 1.80 (q, J = 3.0 Hz, H-8), 1.64–1.79 (m, 1H, H-3), 1.59 (ddt, J = 13.5, 3.0, 1.5 Hz, 1H, H-9), 1.53 (dd, J = 16.0, 8.0 Hz, 1H, H-2), 1.26 (dt, J = 14.0, 3.0 Hz, 1H, H-9), 0.80 (d, J = 7.0 Hz, 3H, H-7), 0.65 (d, J = 7.0 Hz, 3H, H-10).

^{13}C NMR (101 MHz, C_6D_6) δ: 212.2 (C-1), 143.0 (C-11), 138.1 (C-14), 129.8 (C-13), 127.5 (C-12), 54.2 (C-4), 46.7 (C-8), 44.9 (C-5), 44.7 (C-2), 32.4 (C-6), 31.9 (C-3), 21.8 (C-9), 21.4 (C-10), 21.2 (C-15), 17.4 (C-7).

HRMS (ES+): exact mass calculated for $[M+H]^+$ $(C_{17}H_{24}O_3N^{32}S)^+$ requires 322.1471, found 322.1474.

Screening of conditions for oxidation to β-methyl enone (18)

Table S4. Conditions screening for oxidation to enone.

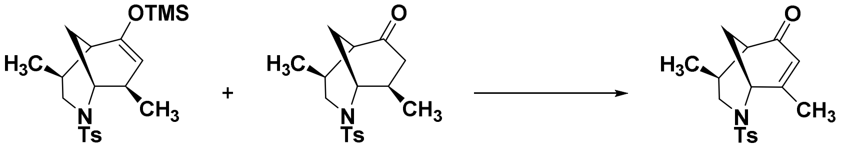

S29 S34 18

Entry	Starting material	Reagents and conditions	Observation
**1**	S34	Pd(TFA)$_2$(DMSO)$_2$, O_2, AcOH, 80 °C	No reaction
**2**	S29	Pd(TFA)$_2$(DMSO)$_2$, O_2, AcOH, 80 °C	Hydrolysis of enol ether
**3**	S34	IBX, EtOAc, 100 °C	Traces of product
**4**	S29	IBX, EtOAc, 100 °C	Traces of product
**5**	S29	IBX, MPO, EtOAc, 100 °C	Traces of product
**6**	S29	Pd(OAc)$_2$ (20 mol%), *p*-benzoquinone	Traces of product
**7**	S29	Pd(OAc)$_2$ (20 mol%), O_2 DMSO, Na_2HPO_4, 90 °C	Traces of product
**8**	S29	Pd(OAc)$_2$ (20 mol%), Oxone, DMSO, Na_2HPO_4, 90 °C	25%
**9**	S29	Pd(OAc)$_2$ (50 mol%), O_2, DMSO, Na_2HPO_4, 90 °C	56%

The oxidation of β-methyl enol silane **S29** or β-methyl ketone **S34** proved much more challenging than the oxidation of ketone **S28**. Under the Stahl's conditions,[23] ketone **S34** did not undergo oxidation to enone **18** (Entry 1), while enol silane **S29** was hydrolyzed to ketone **S34** under the acidic reaction conditions (Entry 2). Treatment of ketone **S34** with IBX, or enol silane **S29** with IBX, with or without the additive *p*-methyoxypyridine-N-oxide (MPO),[24] led to the formation of enone **18** in very low yields (Entries 3–5). Low yields were also obtained using Pd(OAc)$_2$ under Saegusa–Ito conditions with *p*-benzoquinone,[25] Larock conditions with O_2,[26] or Lebel conditions with Oxone®,[27] and meaningful yields could only be obtained with stoichiometric quantities of Pd catalyst (Entries 6–9). Attempts at carrying out a one-pot conjugate addition-oxidation from enone **16,** as described by Matsuo[28] and Kerr[29] were also unsuccessful.

(*1R,4S,5R,7S,8S*)-7-bromo-4,8-dimethyl-2-tosyl-2-azabicyclo[3.3.1]nonan-6-one (17)

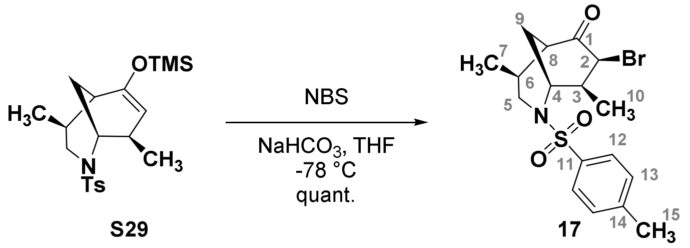

To a solution of silyl enol ether **S29** (11.6 g, 29.5 mmol, 1 equiv.) in dry THF (270 mL) at -78 °C was added $NaHCO_3$ (2.98 g, 35.4 mmol, 1.20 equiv.) followed by NBS (6.31 g, 35.4 mmol, 1.20 equiv.). After stirring for 30 min at this temperature, the reaction mixture was diluted with Et_2O (250 mL) and washed with sat. aq. solution of $NaHCO_3$ (300 mL). The aqueous layer was back-extracted with Et_2O (3 x 200 mL), and the combined organic phases were dried over Na_2SO_4, filtered and concentrated under reduced pressure. The bromide **17** was obtained as a yellowish solid contaminated with traces of succinimide (13.8 g obtained, theoretical yield 11.8 g, supp. quant.). The obtained product was used in the next step without further purification.

NOTE: Decomposition occurs quickly on silica.

MP: 160–162 °C (*rac*), 150–152 °C (>99% *ee*).

IR (thin film) $\tilde{\nu}_{max}$/cm^{-1}: 3018, 2925, 1721, 1345, 1165, 1099.

^{1}H NMR (400 MHz, $CDCl_3$) δ: 7.74 – 7.64 (m, 2H, H-12), 7.39 – 7.27 (m, 2H, H-13), 5.29 (d, *J* = 6.2 Hz, 1H, H-2), 3.82 – 3.74 (m, 1H, H-4), 3.49 (dd, *J* = 12.1, 6.0 Hz, 1H, H-5a), 2.94 – 2.81 (m, 1H, H-3), 2.63 (dd, *J* = 12.1, 9.3 Hz, 1H, H-5b), 2.49 – 2.40 (m, 1H, H-8), 2.44 (s, 3H, H-15), 2.22 (dtd, *J* = 9.0, 6.4, 2.2 Hz, 1H, H-6), 2.09 (dt, *J* = 14.7, 3.0 Hz, 1H, H-9a), 1.82 (dddd, *J* = 14.6, 3.8, 2.8, 2.0 Hz, 1H, H-9b), 1.06 (d, *J* = 7.1 Hz, 3H, H-10), 1.01 (d, *J* = 6.8 Hz, 3H, H-7).

^{13}C NMR (101 MHz, $CDCl_3$) δ: 203.2 (C-1), 144.1 (C-11), 134.6 (C-14), 130.1 (C-13), 127.6 (C-12), 56.1 (C-2), 55.2 (C-4), 48.4 (C-8), 45.9 (C-5), 43.4 (C-3), 31.8 (C-6), 22.4 (C-9), 21.7 (C-15), 19.4 (C-7), 16.3 (C-10).

HRMS (ES+): exact mass calculated for $[M+Na]^+$ $(C_{17}H_{22}O_2NBrNaS)^+$ requires 422.0396, found 422.0395.

$[\alpha]_D^{25}$= +35.93 (c = 1.00, $CHCl_3$).

(*1R,4S,5R*)-4,8-dimethyl-2-tosyl-2-azabicyclo[3.3.1]non-7-en-6-one (18)

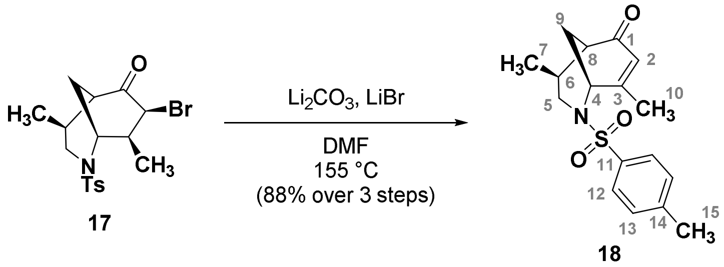

A solution of crude bromide **17** (11.8 g, 29.5 mmol, 1 equiv.), Li_2CO_3 (5.45 g, 73.8 mmol, 2.50 equiv.) and LiBr (3.84 g, 44.3 mmol, 1.50 equiv.) in DMF (122 mL) was heated to 155 °C for 3 h. Then, the reaction mixture was cooled to room temperature, diluted with ethyl acetate (500 mL) and washed with 1 M aq. HCl (2 x 200 mL) and brine (6 x 150 mL). The combined organic layers were dried over Na_2SO_4, filtered and concentrated under reduced pressure. Purification by gradient column chromatography (ethyl acetate:pentane = 15:85 to 25:75) afforded enone **18** as a white solid (8.30 g, 88% over 3 steps).

Note: The *ee* of product **18** can be increased at this stage by recrystallization from a boiling mixture of hexane and ethyl acetate (23 mL of hexane per g of **18** and 4.8 mL of ethyl acetate per g of **18**). The recrystallization was repeated with material obtained from mother liquor (74% yield of recrystallyzation, >99% *ee*).

MP: 108–110 °C (racemic), 91–93 °C (>99% ee).

IR (thin film) $\tilde{\nu}_{max}$/cm^{-1}: 2923, 1669, 1329, 1160, 1094.

^{1}H NMR (400 MHz, $CDCl_3$) δ: 7.78 – 7.69 (m, 2H, H-12), 7.35 – 7.27 (m, 2H, H-13), 6.02 (p, J = 1.4 Hz, 1H, H-2), 4.52 (t, J = 3.2 Hz, 1H, H-4), 3.32 (d, J = 13.8 Hz, 1H, H-5a), 3.22 (dd, J = 13.9, 4.0 Hz, 1H, H-5b), 2.43 (s, 3H, H-15), 2.31 – 2.25 (m, 1H, H-8), 2.14 (dt, J = 13.2, 3.0 Hz, 1H, H-9a), 1.97 (dt, J = 13.3, 3.4 Hz, 1H, H-9b), 1.94 – 1.87 (m, 1H, H-6), 1.89 (d, J = 1.5 Hz, 3H, H-10), 0.90 (d, J = 7.0 Hz, 3H, H-7).

^{13}C NMR (101 MHz, $CDCl_3$) δ: 201.4 (C-1), 154.8 (C-3), 143.7 (C-11), 137.8 (C-14), 129.8 (C-13), 129.5 (C-2), 127.3 (C-12), 51.6 (C-4), 45.7 (C-8), 43.9 (C-5), 31.4 (C-6), 27.2 (C-9), 22.1 (C-10), 21.7 (C-15), 17.6 (C-7).

HRMS (ES+): exact mass calculated for $[M+H]^+$ $(C_{17}H_{22}O_3N^{32}S)^+$ requires 320.1315 , found 320.1316.

$[\alpha]_D^{25}$= +385.86 (c = 1.00, $CHCl_3$).

SFC: Chiralpak® IA; 1500 psi, 30 °C; flow: 1.5 mL.min^{-1}; from 1% to 30% MeOH in 5 min; >99.8% *ee* (major enantiomer t_R = 3.96 min; minor enantiomer t_R = 3.81 min).

(*1R,4S,5R*)-4,8-dimethyl-2-azabicyclo[3.3.1]non-7-en-6-one (4)

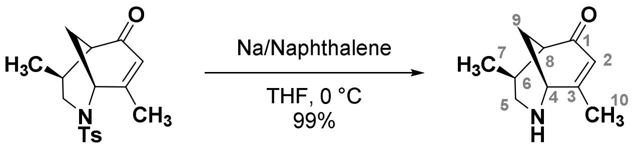

Preparation of sodium naphthalene: To 1 M solution of naphthalene in THF was added sodium (1.5 equiv. related to naphthalene) washed with pentane and cut to small pieces. The resulting mixture was placed into a sonicator for 60 min, which resulted in the slow dissolution of sodium and the formation of a dark blue-colored solution.

To a solution of enone **18** (5.00 g, 15.7 mmol, 1 equiv.) in dry THF (157 mL) was added NaHMDS (1 M in THF, 32.9 mL, 32.9 mmol, 2.10 equiv.) at 0 °C and after stirring for 30 min at this temperature, freshly prepared solution of sodium naphthalene (1M in THF, 62.6 mL, 62.6 mmol, 4.00 equiv.) was added dropwise, with the blue-black color dissipating during the addition until approximately 3 equiv. had been added, after which the solution remained black. After stirring for 30 min, the reaction was quenched with distilled water (150 mL) and diluted with Et_2O (150 mL). The layers were separated, and aq. phase washed with CH_2Cl_2/IPA (9:1) (6 x 100 mL). The combined organic layers were dried over Na_2SO_4, filtered and concentrated under reduced pressure. Purification by gradient column chromatography (ethyl acetate:methanol = 100:0 to 70:30) afforded amine **4** as a yellow oil (2.58 g, 99%).

IR (thin film) $\tilde{\nu}_{max}$/cm^{-1}: 3316, 2959, 2919, 1661.

^{1}H NMR (400 MHz, $CDCl_3$) δ: 6.07 (s, 1H, H-2), 3.42 (t, *J* = 3.4 Hz, 1H, H-4), 3.01 (dd, *J* = 12.7, 4.4 Hz, 1H, H-5a), 2.55 (d, *J* = 12.7 Hz, 1H, H-5b), 2.31 – 2.22 (m, 2H, H-8, H-9a), 1.97 (s, 3H, H-10), 1.93 (dt, *J* = 14.0, 3.7 Hz, 1H, H-9b), 1.89 – 1.79 (m, 2H, H-6, NH), 1.26 (d, *J* = 7.1 Hz, 3H).

^{13}C NMR (101 MHz, $CDCl_3$) δ: 202.9 (C-1), 156.1 (C-3), 128.3 (C-2), 52.4 (C-1), 46.6 (C-8), 43.4 (C-5), 30.9 (C-6), 28.2 (C-9), 22.2 (C-10), 17.5 (C-7).

HRMS (ES+): exact mass calculated for $[M+H]^+$ $(C_{10}H_{16}ON)^+$ requires 166.1226, found 166.1227.

$[\alpha]_D^{25}$= +250.3 (c = 1.00, $CHCl_3$)

3.4.2 Introduction of the C-ring

Scheme S9. Introduction of the C-ring.

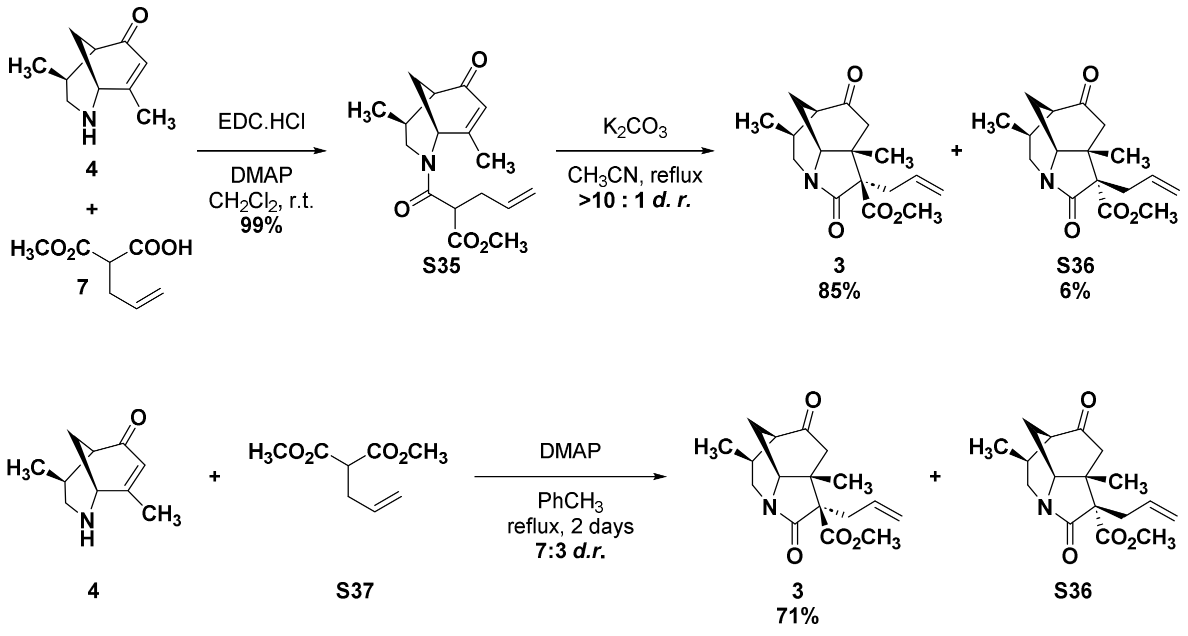

methyl 2-((*1R,4S,5R*)-4,8-dimethyl-6-oxo-2-azabicyclo[3.3.1]non-7-ene-2-carbonyl)pent-4-enoate (S35)

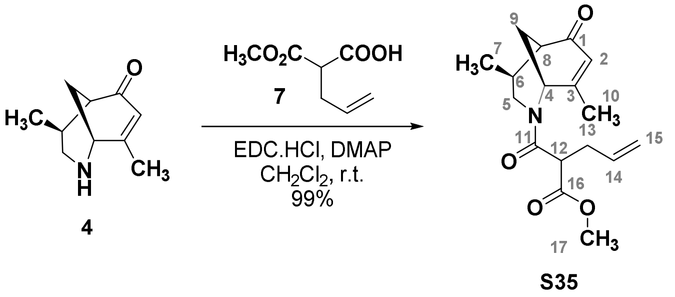

To a solution of amine **4** (2.58 g, 15.6 mmol, 1 equiv.) in CH_2Cl_2 (78.0 mL) at room temperature was added 2-(methoxycarbonyl)pent-4-enoic acid (3.46 g, 21.9 mmol, 1.40 equiv.), followed by DMAP (477 mg, 3.90 mmol, 0.25 equiv.) and EDC.HCl (4.49 g, 23.4 mmol, 1.50 equiv.). After stirring for 3 h, the reaction mixture was diluted with water (80 mL) and extracted with CH_2Cl_2 (4 x 80 mL). The combined organic phases were dried over anhydrous Na_2SO_4, filtered and concentrated under reduced pressure. Purification by column chromatography (ethyl acetate:pentane = 40:60) afforded amide **S35** as a colorless oil (4.73 g, 99%). Product **S35** is a complex mixture of isomers – presumably diastereomers at C12 and rotamers.

IR (thin film) $\tilde{\nu}_{max}$/cm^{-1}: 2922, 1710, 1645, 1199.

^{1}H NMR (500 MHz, $CDCl_3$) δ: 6.08 – 6.05 (m, 1H, H-2), 5.86 – 5.65 (m, 1H, H-14), 5.38 (s, 1H, H-4), 5.23 – 4.94 (m, 2H, H-15), 3.73 (s, 3H, H-17), 3.67 (s, 3H, H-17), 3.66 – 3.59 (m, 1H, H-12), 3.54 (d, *J* = 13.7 Hz, 1H, H-5), 3.43 – 3.30 (m, 1H, H-5), 2.81 – 2.61 (m, 2H, H-13), 2.42 – 2.35 (m, 1H, H-8), 2.24 – 2.09 (m, 1H, H-9), 2.06 – 2.00 (m, 1H-H9), 2.01 – 1.90 (m, 1H, H-6), 1.94 (d, *J* = 1.5 Hz, 3H, H-10), 1.90 (d, *J* = 1.5 Hz, 3H, H-10), 1.17 (d, *J* = 7.4 Hz, 3H, H-7), 1.15 (d, *J* = 7.4 Hz, 3H, H-7).

^{13}C NMR (126 MHz, $CDCl_3$) δ: 201.62 (C-1), 201.55 (C-1), 169.9 (C-16), 169.8 (C-16), 168.5 (C-11), 156.3 (C-3), 156.0 (C-3), 134.8 (C-14), 134.5 (C-14), 129.5 (C-2), 129.4 (C-2), 117.7 (C-15), 117.6 (C-15), 52.60 (C-17), 52.57 (C-17), 49.2 (C-12), 49.0 (C-12), 47.8 (C-4), 47.7 (C-4), 46.14 (C-8), 46.10 (C-8), 45.0 (C-5), 44.8 (C-5), 33.7 (C-13), 33.3 (C-13), 31.6 (C-6), 31.3 (C-6), 26.41 (C-9), 26.39 (C-9), 22.11 (C-10), 22.08 (C-10), 17.6 (C-7), 17.4 (C-7).

HRMS (ES+): exact mass calculated for $[M+H]^+$ $(C_{17}H_{24}O_4N)^+$ requires 306.1700, found 306.1699.

$[\alpha]_D^{25}$= +62.2° (c = 1.00, in $CHCl_3$, >99% ee).

methyl (*3R,3aR,6R,7aS,8S*)-3-allyl-3a,8-dimethyl-2,5-dioxooctahydro-6,1-ethanoindole-3-carboxylate (3) and methyl (*3S,3aR,6R,7aR,8S*)-3-allyl-3a,8-dimethyl-2,5-dioxooctahydro-6,1-ethanoindole-3-carboxylate (S36)

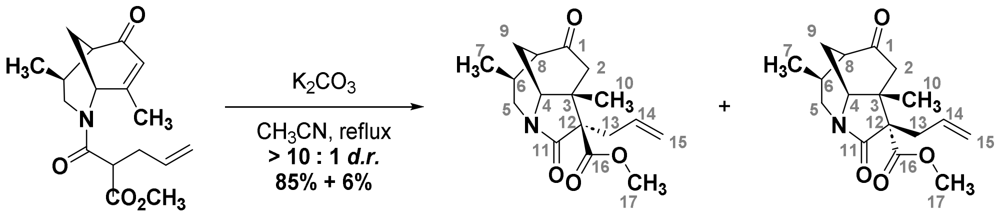

S35 **3** **S36**

Compound **3** was prepared adopting the procedure published by Li[30] To a solution of enone **S35** (4.63 g, 15.2 mmol, 1 equiv.) in CH_3CN (62.0 mL) was added K_2CO_3 (10.5 g, 75.8 mmol, 5.00 equiv.) and the resulting mixture was purged with argon for 10 minutes and then heated to 100 °C. After stirring for 15 h, the reaction mixture was cooled down to room temperature, concentrated under reduced pressure and loaded onto silica. Purification by gradient column chromatography (ethyl acetate:pentane = 30:70 to 50:50) afforded compound **3** as a white solid (3.93 g, 85%) and compound **S36** as a white amorphous solid (285 mg, 6.2%).

MP: 116 – 118 °C (>99% *ee*), 130 – 132 °C (*rac*).

IR (neat) $\tilde{\nu}_{max}$/cm^{-1}: 2972, 2934, 2878, 1712, 1686, 1430, 1226, 1152, 914.

^{1}H NMR (400 MHz, $CDCl_3$) δ: 6.13 (dddd, J = 17.2, 10.2, 9.2, 4.5 Hz, 1H, H-14), 5.20 (dtd, J = 17.3, 2.1, 1.0 Hz, 1H, H-15a), 5.11 (dtd, J = 10.2, 2.0, 0.8 Hz, 1H, H-15b), 4.01 (dd, J = 13.5, 8.8 Hz, 1H, H-5a), 4.00 – 3.96 (m, 1H, H-4), 3.69 (s, 3H, H-7), 2.96 – 2.85 (m, 1H, H-6), 2.84 (ddt, J = 15.2, 4.3, 2.1 Hz, 1H, H-H-13a), 2.75 (d, J = 14.7 Hz, 1H, H-2a), 2.33 (dd, J = 15.2, 9.3 Hz, 1H, H-13b), 2.27 (dd, J = 13.5, 9.6 Hz, 1H, H-5b), 2.18 (d, J = 14.7 Hz, 1H, H2-b), 2.13 – 2.06 (m, 2H, H-9), 1.96 – 1.90 (m, 1H, H-8), 1.18 (s, 3H, H-10), 1.00 (d, J = 7.0 Hz, 3H, H-7).

^{13}C NMR (101 MHz, $CDCl_3$) δ: 212.5 (C-1), 171.3 (C-16), 170.6 (C-11), 134.3 (C-14), 118.6 (C-15), 60.7 (C-12), 59.5 (C-4), 52.3 (C-17), 50.5 (C-3), 46.2 (C-8), 46.0 (C-2), 41.2 (C-5), 36.2 (C-6), 34.9 (C-13), 21.5 (C-10), 19.8 (C-9), 17.8 (C-7).

HRMS (ES+): exact mass calculated for $[M+H]^+$ $(C_{17}H_{24}NO_4)^+$ requires 306.1700, found 306.1964.

$[\alpha]^{25}_D$= -97.3 (c = 1.00, $CHCl_3$).

Minor diastereomer S36

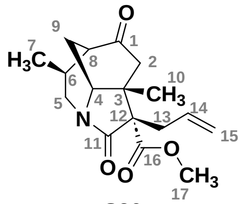

S36

IR (thin film) ṽ$_{max}$/cm^{-1}: 2952, 2925, 2872, 1754, 1689, 1426, 1292, 1156.

^{1}H NMR (400 MHz, CDCl$_3$) δ: 5.77 (ddt, J = 17.2, 10.1, 7.2 Hz, 1H, H-14), 5.16 – 5.03 (m, 1H, H-15a, H-15b), 4.07 (dd, J = 13.6, 8.8 Hz, 1H, H-5a), 3.81 – 3.76 (m, 1H, H-4), 3.78 (s, 3H, H-17), 2.98 – 2.84 (m, 1H, H-6), 2.77 (ddt, J = 13.8, 7.2, 1.4 Hz, 1H, H-13a), 2.38 (ddt, J = 13.9, 7.2, 1.1 Hz, 1H, H-13b), 2.28 – 2.18 (m, 2H, H-2a, H-5b), 2.15 – 2.00 (m, 3H, H-2b, H-9a, H-9b), 1.98 – 1.92 (m, 1H, H-8), 1.30 (s, 3H, H-10), 1.00 (d, J = 7.0 Hz, 3H, H-7).

^{13}C NMR (101 MHz, CDCl$_3$) δ: 210.9 (C-1), 170.5 (C-11 or C-16), 170.3 (C-16 or C-11), 132.6 (C-14), 119.1 (C-15), 61.5 (C-12), 58.3 (C-4), 52.0 (C-17), 49.17 (C-3 or C-2), 49.15 (C-2 or C-3), 45.8 (C-8), 40.9 (C-5), 37.1 (C-6), 36.3 (C-13), 19.40 (C-10 or C-9), 19.37 (C-9 or C-10), 18.1 (C-7).

HRMS (ES+): exact mass calculated for $[M+H]^+$ $(C_{17}H_{24}NO_4)^+$ requires 306.1700, found 306.1996.

$[\alpha]_D^{25}$= -44.6 (c = 1.00, CHCl$_3$).

One-step procedure for the synthesis of 3 from amine 4

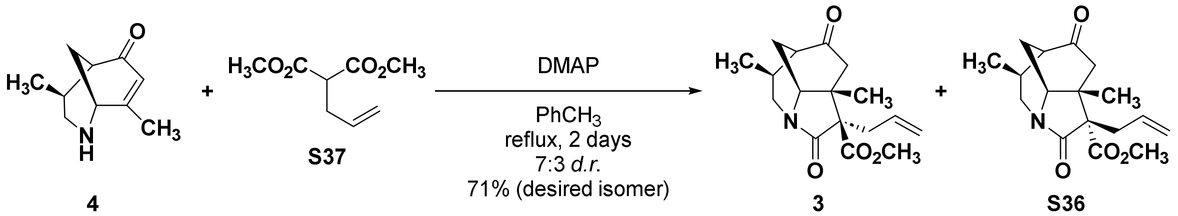

A solution of amine **4** (20.0 mg, 121 µmol, 1 equiv.), dimethyl allylmalonate (83.4 mg, 484 µmol, 3.00 equiv.) and DMAP (7.4 mg, 60.5 µmol, 0.50 equiv.) in dry toluene (3.6 mL) was refluxed for 2 days with Dean-Stark trap. The reaction mixture was then quenched with sat. aq. solution of NH_4Cl (10 mL) and extracted with ethyl acetate (3 x 10 mL). The combined organic phases were dried over anhydrous Na_2SO_4, filtered and concentrated. The crude reaction mixture was analyzed by NMR. Purification by gradient column chromatography (ethyl acetate:pentane = 30:70 to 60:40) provided isomer **3** as a white solid (26.1 mg, 71%).

3.4.3 Formal synthesis of Daphniphyllum alkaloids

Scheme S10. Synthesis of common intermediate in Daphniphyllum alkaloids.

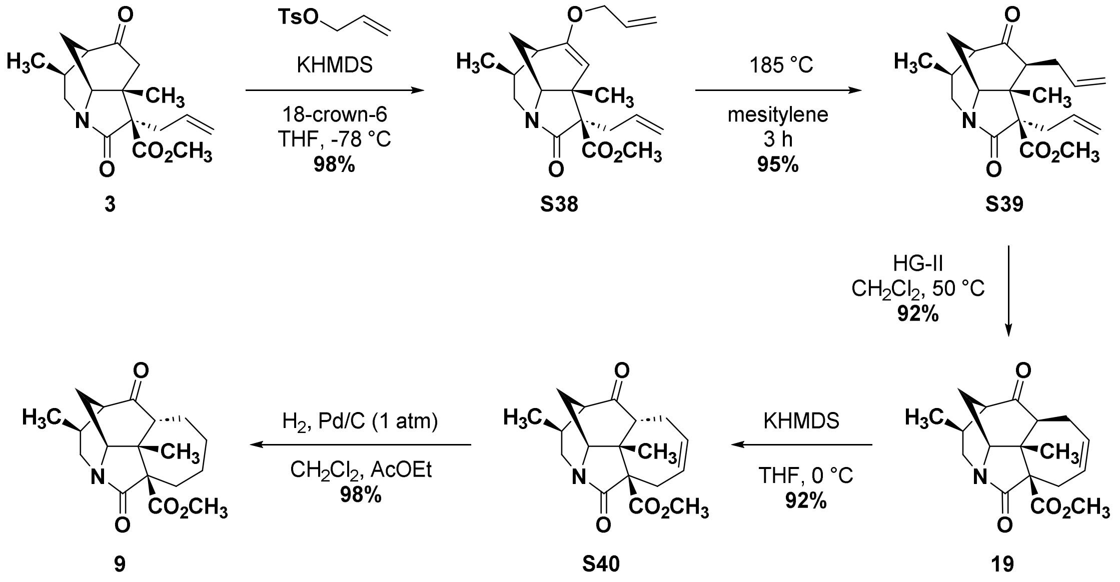

Scheme S11. Application of intermediate **9** in previous syntheses of Daphniphyllum alkaloids.

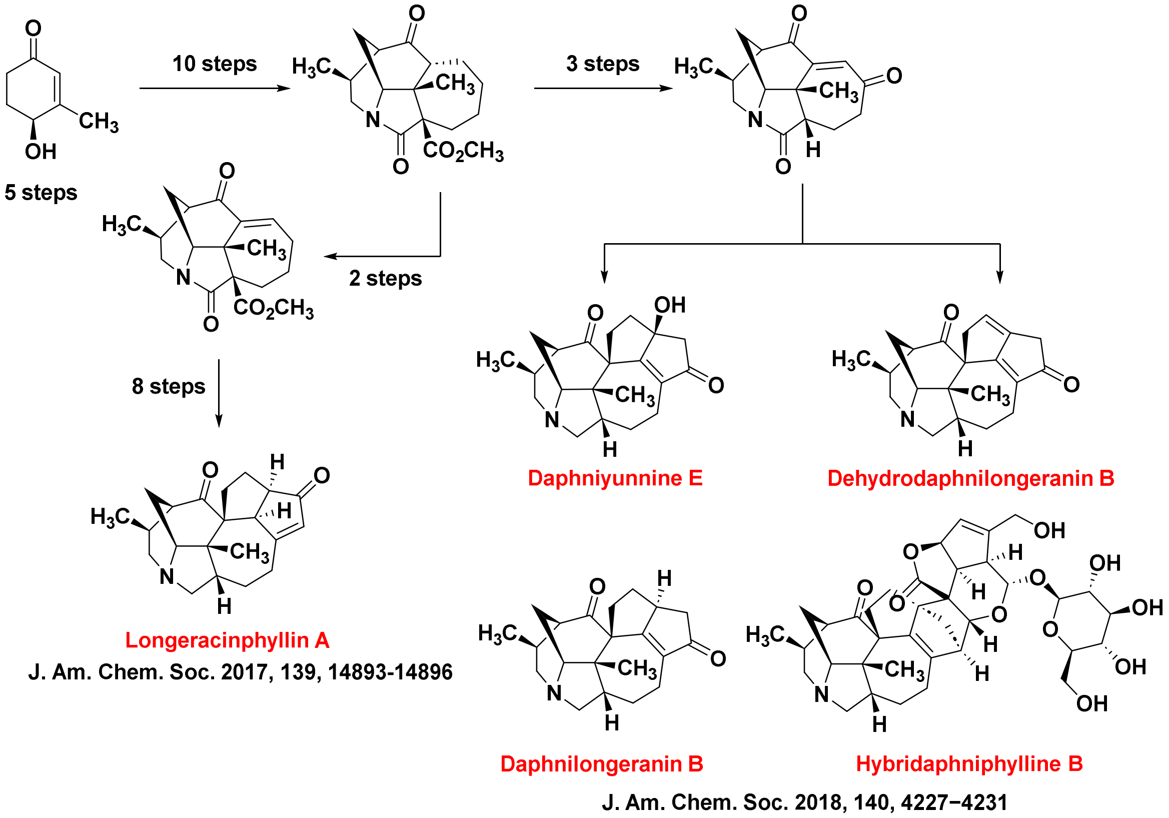

methyl (*3R,3aR,6R,7aR,8S*)-3-allyl-5-(allyloxy)-3a,8-dimethyl-2-oxo-2,3,3a,6,7,7a-hexahydro-6,1-ethanoindole-3-carboxylate (S38)

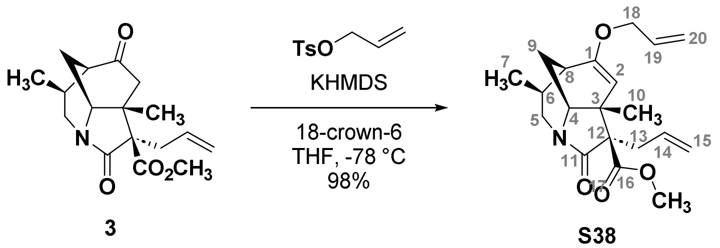

3 **S38**

To a solution of ketone **3** (200.0 mg, 0.655 mmol, 1 equiv.) in dry, degassed THF (4.0 ml) at -78 °C was dropwise added solution of KHMDS (0.5 M in toluene, 1.96 mL, 0.982 mmol, 1.50 equiv.) and the resulting solution was stirred at this temperature for 20 minutes. A solution of 18-crown-6 (173.0 mg, 655 mmol, 1.00 equiv.) in degassed THF (1.0 mL) and allyl *p*-toluenetosylate (208.5 mg, 0.982 mmol, 1.50 equiv.) were added dropwise at -78 °C. After stirring for 1.5 h at -78 °C the cooling bath was removed, and the reaction mixture was allowed to slowly warm up to room temperature (formation of white precipitate in the whole volume of the reaction mixture was observed). The reaction was then quenched with brine (20 mL), extracted with ethyl acetate (4 x 20 mL), dried over anhydrous Na_2SO_4, filtered and concentrated under reduced pressure. Purification by gradient column chromatography (ethyl acetate:pentane = 20:80 to 30:70) afforded allyl enol ether **S38** as a colorless oil (221.1 mg, 98%).

IR (thin film) $\tilde{\nu}_{max}$/cm^{-1}: 2951, 2920, 2872, 1729, 1694, 1644, 1419, 1217, 1196, 1169, 916.

^{1}H NMR (400 MHz, $CDCl_3$) δ: 6.24 (dddd, J = 17.2, 10.2, 9.2, 4.7 Hz, 1H, H-14), 5.91 (ddt, J = 17.3, 10.6, 5.4 Hz, 1H, H-19), 5.28 (dq, J = 17.3, 1.6 Hz, 1H, H-20a), 5.21 (dq, J = 10.4, 1.4 Hz, 1H, H-20b), 5.16 – 5.04 (m, 2H, H-15), 4.28 (d, J = 1.3 Hz, 1H, H-2), 4.22 (ddt, J = 12.6, 5.5, 1.5 Hz, 1H, H-18a), 4.13 (ddt, J = 12.6, 5.4, 1.6 Hz, 1H, H-18b), 4.06 (dd, J = 13.6, 8.0 Hz, 1H, H-5a), 3.85 – 3.79 (m, 1H, H-4), 3.70 (s, 3H, H-17), 2.73 (ddt, J = 14.6, 4.8, 2.0 Hz, 1H, H-13a), 2.47 – 2.35 (m, 2H, H-13b, H-5b), 2.12 – 1.95 (m, 1H, H-6), 1.95 (ddd, J = 13.7, 5.2, 2.3 Hz, 1H, H-9a), 1.93 – 1.87 (m, 1H, H-8), 1.62 (ddt, J = 13.8, 3.9, 1.0 Hz, 1H, H-9b), 1.14 (s, 3H, H-10), 0.96 (d, J = 6.9 Hz, 3H, H-7).

^{13}C NMR (101 MHz, $CDCl_3$) δ: 174.0 (C-16), 170.9 (C-11), 159.6 (C-1), 135.4 (C-14), 133.3 (C-19), 117.6 (C-15), 117.5 (C-20), 95.1 (C-2), 67.9 (C-18), 61.9 (C-12), 56.9 (C-4), 52.1 (C-17), 50.0 (C-3), 41.6 (C-5), 37.4 (C-8), 35.7 (C-13), 31.8 (C-6), 20.1 (C-9), 19.6 (C-10), 19.5 (C-7).

HRMS (ES+): exact mass calculated for $[M+H]^+$ $(C_{20}H_{28}O_4N)^+$ requires 346.2013, found 346.2012.

$[\alpha]_D^{25}$= -151.0 (c = 1.00, $CHCl_3$).

methyl (*3R,3aR,4S,6R,7aR,8S*)-3,4-diallyl-3a,8-dimethyl-2,5-dioxooctahydro-6,1-ethanoindole-3-carboxylate (S39)

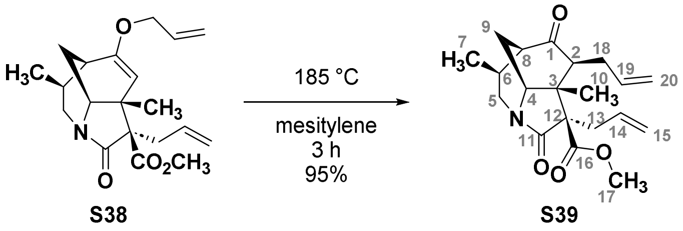

Compound **S39** was prepared adopting the procedure published by Carreira.[31]

Glass passivation: A pressure tube with attached argon balloon was passivated by heating a solution of N,O-bis(trimethylsilyl) acetamide (01. mL) in hexane (10 mL) to 50 °C. The pressure tube was swirled for 5 minutes so that all surface of the reaction vessel was in contact with the reagent. The solution was then discarded and tube washed with hexane (2 x 15 mL) and MTBE (2 x 15 mL) and dried under a high vacuum.

The solution of enol **S38** (190 mg, 0.550 mmol, 1 equiv.) was transferred to passivated pressure tube in benzene (3.0 mL), and the solvent was removed in a vacuum. The solvent-free enol was dissolved in mesitylene (18.3 mL, 0.030 M) (previously distilled and stored over molecular sieves). A PTFE syringe filter was used to ensure that no solid particles were transferred with the solvent into the reaction mixture. The resulting solution was purged with argon for 30 min, and the tube was sealed and heated to 185 °C for 3 h. After cooling to room temperature, the reaction mixture was directly poured onto a silica column and flushed with pentane until mesitylene was no longer eluted. Elution with ethyl acetate:pentane (60:40) afforded product **S39** as a white solid (180 mg, 95%).

MP: 109-112 °C (>99% ee), 112-113 °C (rac).

IR (neat) $\tilde{\nu}_{max}$/cm^{-1}: 2973, 2951, 2930, 1735, 1705, 1639, 1427, 1210, 915, 1425, 1208, 912.

^{1}H NMR (400 MHz, $CDCl_3$) δ: 6.19 (ddt, J = 17.2, 14.7, 4.5 Hz, 1H, H-14), 5.61 (ddt, J = 16.7, 10.3, 6.9 Hz, 1H, H-19), 5.22 – 5.09 (m, 2H, H-15a, H-15b), 5.03 – 4.93 (m, 2H, H-20a, H20b), 4.08 (dd, J = 13.6, 8.4 Hz, 1H, H-5a), 3.81 (d, J = 4.5 Hz, 1H, H-4), 3.70 (s, 3H, H-17), 2.84 – 2.70 (m, 3H, H-13a, H-2, H-6), 2.49 – 2.24 (m, 4H, H-13b, H-5b, H-18a, H18b), 2.21 – 2.05 (m, 2H, H-9a, H-9b), 2.05 – 1.99 (m, 1H, H-8), 1.12 (s, 3H, H-10), 1.01 (d, J = 6.9 Hz, 3H, H-7).

^{13}C NMR (101 MHz, $CDCl_3$) δ: 212.4 (C-1), 172.3 (C-11), 170.7 (C-16), 135.8 (C-19), 134.3 (C-14), 118.8 (C-15), 116.9 (C-20), 64.1 (C-12), 59.7 (C-4), 53.1 (C-3), 52.3 (C-17), 51.2 (C-2), 46.2 (C-8), 41.1 (C-5), 35.4 (C-6), 35.0 (C-13), 33.0 (C-18), 20.2 (C-9), 18.2 (C-7), 16.7 (C-10).

HRMS (ES+): exact mass calculated for $[M+H]^+$ $(C_{20}H_{28}NO_4)^+$ requires 346.2013, found 346.2010.

$[\alpha]_D^{25}$= 10.2 (c = 1.00, $CHCl_3$).

methyl (*2R,3S,6aR,11S,11aR,11bR*)-3,11a-dimethyl-6,12-dioxo-1,3,4,7,10,11,11a,11b-octahydro-2H-2,11-methanocyclohepta[a]indolizine-6a(6H)-carboxylate (19)

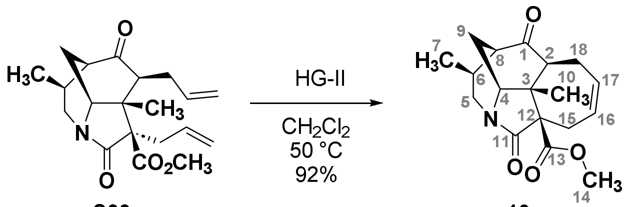

S39 19

To a solution of diene **S39** (87.0 mg, 0.252 mmol, 1 equiv.) in degassed CH_2Cl_2 (50.5 mL) was added Grubbs-Hoveyda 2nd generation catalyst (10.7 mg, 12.6 µmol, 5.0 mol %), reflux condenser was attached, and the reaction mixture was heated to reflux for 19 h while gently bubbled with argon. The reaction was then quenched by the addition of a solution of potassium isocyanoacetate (25 mg) in methanol (3.0 mL). After stirring for 30 min at room temperature, the reaction mixture was concentrated under reduced pressure and loaded onto silica. Purification by gradient column chromatography (ethyl acetate:pentane = 40:60 to 60:40) afforded compound **19** as a white solid (73.6 mg, 92%).

MP: 196-199 °C (>99% ee), 186-188 °C (rac).

IR (thin film) $\tilde{\nu}_{max}/cm^{-1}$: 3024, 2969, 2951, 2922, 1725, 1695, 1414, 1292, 1209.

^{1}H NMR (400 MHz, $CDCl_3$) δ: 5.75 – 5.63 (m, 1H, H-16), 5.56 (ddt, J = 10.8, 5.3, 2.5 Hz, 1H, H-17), 4.10 (dd, J = 13.7, 6.8 Hz, 1H, H-5a), 3.87 (m, 1H, H-4), 3.73 (s, 3H, H-14), 3.17 (dd, J = 11.8, 4.8 Hz, 1H, H-2), 2.80 – 2.71 (m, 1H, H-15a), 2.74 (dd, J = 13.7, 11.3 Hz, 1H, H-5b), 2.66 (ddt, J = 19.3, 5.1, 2.8 Hz, 1H, H-18a), 2.58 (dd, J = 16.0, 8.6 Hz, 1H, H-15b), 2.37 – 2.22 (m, 3H, H-18b, H-8, H-9a), 2.15 – 2.01 (m, 1H, H-6), 1.85 – 1.75 (m, 1H, H-9b), 1.14 (s, 3H, H-10), 1.01 (d, J = 6.9 Hz, 3H, H-7).

^{13}C NMR (101 MHz, $CDCl_3$) δ: 211.1 (C-1), 175.9 (C-11), 170.9 (C-13), 129.0 (C-17), 124.2 (C-16), 65.4 (C-12), 57.3 (C-4), 55.1 (C-3), 52.5 (C-14), 50.0 (C-8), 44.1 (C-2), 42.3 (C-5), 32.7 (C-6), 29.2 (C-15), 27.5 (C-18), 22.3 (C-9), 20.0 (C-7), 16.0 (C-10).

HRMS (ES+): exact mass calculated for $[M+H]^+$ $(C_{18}H_{24}NO_4)^+$ requires 318.1700, found 318.1703.

$[\alpha]_D^{25}$= 175.3 (c = 1.00, $CHCl_3$).

methyl (*2R,3S,6aR,11R,11aR,11bR*)-3,11a-dimethyl-6,12-dioxo-1,3,4,7,10,11,11a,11b-octahydro-2H-2,11-methanocyclohepta[a]indolizine-6a(6H)-carboxylate (S40)

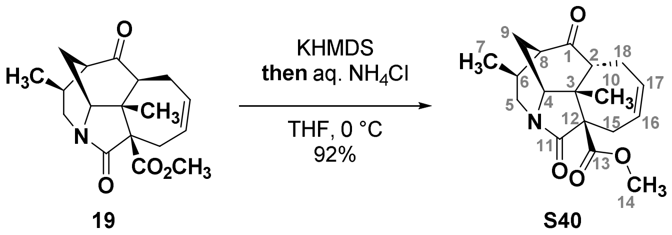

To a solution of ketone **19** (60.0 mg, 0.189 mmol, 1 equiv.) in dry, degassed THF (4.0 ml) at 0 °C was dropwise added solution of KHMDS (0.5 M in toluene, 560 µL, 0.284 mmol, 1.50 equiv.) and the reaction mixture was stirred at this temperature for 50 min. The reaction was then quenched with sat. aq. sol. of NH_4Cl (15 mL), extracted with CH_2Cl_2 (4 x 15 mL), dried over anhydrous Na_2SO_4, filtered and concentrated under reduced pressure. Purification by gradient column chromatography (ethyl acetate:pentane = 40:60 to 80:20) afforded compound **S40** as a white solid (55.2 mg, 92%).

NOTE: Compound **19** can be epimerized under thermodynamic conditions by heating its solution in MeOH in the presence of K_2CO_3 (50 °C, 30 min).

MP: 195-198 °C (>99% ee), 176-179 °C (rac).

IR (thin film) $\tilde{\nu}_{max}$/cm^{-1}: 2953, 2872, 1705, 1431, 1229.

^{1}H NMR (500 MHz, CDCl$_3$) δ: 6.11 (dddd, J = 9.3, 7.2, 5.6, 1.9 Hz, 1H, H-17), 5.73 (dddd, J = 9.9, 8.1, 5.3, 1.8 Hz, 1H, H-16), 4.07 (dd, J = 13.6, 9.1 Hz, 1H, H-5a), 3.90 – 3.85 (m, 1H, H-4), 3.70 (s, 3H, H-14), 2.88 – 2.79 (m, 1H, H-6), 2.76 (ddd, J = 15.2, 5.3, 1.9 Hz, 1H, H-15a), 2.52 – 2.46 (m, 2H, H-18a, H-2), 2.46 (dd, J = 8.1, 7.1 Hz, 1H, H-15b), 2.32 (dd, J = 13.7, 9.5 Hz, 1H, H-5b), 2.33 – 2.25 (m, 1H, H-18b), 2.12 – 2.00 (m, 2H, H-9a, H-9b), 1.94 (ddd, J = 3.7, 2.1, 0.9 Hz, 1H, H-8), 1.32 (s, 3H, H-10), 1.03 (d, J = 7.0 Hz, 3H, H-7).

^{13}C NMR (126 MHz, CDCl$_3$) δ: 212.1 (C-1), 171.5 (C-13), 170.5 (C-11), 132.4 (C-17), 126.5 (C-16), 60.9 (C-12), 60.0 (C-4), 53.7 (C-2), 53.4 (C-3), 52.6 (C-14), 46.3 (C-8), 40.6 (C-5), 36.7 (C-6), 26.7 (C-15), 22.0 (C-10), 21.9 (C-18), 18.4 (C-9), 18.2 (C-7).

HRMS (ES+): exact mass calculated for $[M+H]^+$ $(C_{18}H_{24}NO_4)^+$ requires 318.1705, found 318.1701.

$[\alpha]_D^{25}$= -89.9 (c = 1.00, $CHCl_3$).

methyl (*2R,3S,6aR,11R,11aR,11bR*)-3,11a-dimethyl-6,12-dioxodecahydro-2H-2,11-methanocyclohepta[a]indolizine-6a(6H)-carboxylate (9)

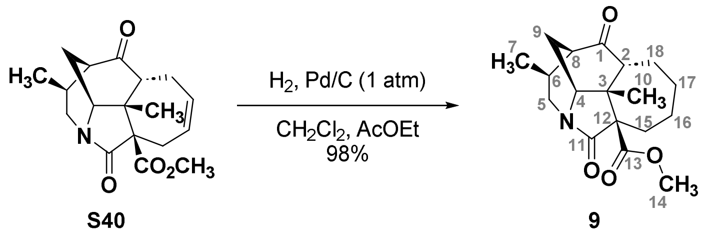

To a solution of alkene **S40** (29.0 mg, 91.4 µmol, 1 equiv.) in a mixture of ethyl acetate (6.0 mL) and CH_2Cl_2 (3.0 mL) was added palladium on charcoal (10 wt %, 4.9 mg, 4.57µmol, 5.0 mol %). After the introduction of hydrogen from the double-coated balloon and repeating 4 cycles of vacuum/hydrogen, the reaction mixture was stirred for 2 h. Then, the reaction mixture was filtered through a pad of Celite and concentrated under reduced pressure. Purification by gradient column chromatography (ethyl acetate:pentane = 40:60 to 80:20) afforded compound **9** as a white solid (28.6 mg, 98%). The spectroscopic data are in agreement with the literature values.[32]

MP: 189-193 °C (>99% ee), 174-177 (rac).

IR (thin film) $\tilde{\nu}_{max}$/cm^{-1}: 2951, 2928, 2878, 1730, 1703, 1693, 1454, 1435, 1248, 1213,1196, 752.

^{1}H NMR (500 MHz, $CDCl_3$) δ: 4.11 (dd, J = 13.6, 8.7 Hz, 1H, H-5a), 3.96 (d, J = 5.2 Hz, 1H, H-4), 3.68 (s, 3H, H-14), 2.84 – 2.73 (m, 1H, H-6), 2.53 (dd, J = 6.0, 2.2 Hz, 1H, H-2), 2.37 (dd, J = 13.6, 9.8 Hz, 1H, H-5b), 2.30 – 2.20 (m, 1H, H-17a), 2.16 (dd, J = 15.2, 9.0 Hz, 1H, H-15a), 2.13 – 2.03 (m, 2H, H-9a, H-9b), 1.99 – 1.86 (m, 3H, H-15b, H-18a, H-8), 1.79 – 1.72 (m, 1H, H-18b), 1.72 – 1.63 (m, 1H, H-16a), 1.51 – 1.39 (m, 1H, H-17b), 1.23 (s, 3H, H-10), 1.18 – 1.06 (m, 1H, H-16b), 1.03 (d, J = 7.0 Hz, 3H, H-7).

^{13}C NMR (126 MHz, $CDCl_3$) δ: 214.5 (C-1), 171.8 (C-13), 171.4 (C-11), 64.8 (C-12), 60.2 (C-4), 54.6 (C-2), 52.9 (C-3), 52.4 (C-14), 46.8 (C-8), 41.1 (C-5), 36.9 (C-6), 27.3 (C-15), 23.7 (C-17), 22.3 (C-10), 21.1 (C-16), 20.4 (C-18), 19.1 (C-9), 18.4 (C-7).

HRMS (ESI) m/z: $[M + H]^+$ Calcd 320.1856 for $C_{18}H_{25}NO_4$; Found 320.1857.

HRMS (ES+): exact mass calculated for $[M+H]^+$ $(C_{18}H_{26}NO_4)^+$ requires 320.1856, found 320.1857.

$[\alpha]_D^{25}$= -118.0 (c = 1.00, $CHCl_3$, >99% *ee*), literature value $[\alpha]_D^{25}$= -77.4 (c = 1.0, $CHCl_3$, 99.8% *ee*).[32]

HPLC (Chiralpak AD-H, hexane/isopropanol 85:15, 1.0 mL min^{-1}, λ =210 nm).

Table S5. Comparison of ^{1}H (500 MHz, $CDCl_3$) and ^{13}C NMR (126 MHz, $CDCl_3$) spectra of compound **9** prepared by Li's[32] and our group.

	^{1}H NMR data				^{13}C NMR data		
**Atom**	**Synthesized**	**Reported**	**Diff.**	**Atom**	**Synth.**	**Rep.**	**Diff.**
**5a**	4.11 (dd, J = 13.6, 8.7 Hz, 1H)	4.10 (dd, J = 13.5, 8.8 Hz, 1H)	0.01	**1**	214.5	214.4	0.1
**4**	3.96 (d, J = 5.2 Hz, 1H)	3.96 (d, J = 4.9 Hz, 1H)	0.00	**13**	171.8	171.6	0.2
**14**	3.68 (s, 3H)	3.67 (s, 3H)	0.01	**11**	171.4	171.3	0.1
**6**	2.84 – 2.73 (m, 1H)	2.85 – 2.70 (m, 1H)	0.01*	**12**	64.8	64.7	0.1
**2**	2.53 (dd, J = 6.0, 2.2 Hz, 1H)	2.52 (dd, J = 5.8, 1.9 Hz, 1H)	0.01	**4**	60.2	60.1	0.1
**5b**	2.37 (dd, J = 13.6, 9.8 Hz, 1H)	2.36 (dd, J = 13.5, 9.8 Hz, 1H)	0.01	**2**	54.6	54.3	0.3
**17a**	2.30 – 2.20 (m, 1H)	2.29 – 2.20 (m, 1H)	0.01*	**3**	52.9	52.8	0.1
**15a**	2.16 (dd, J = 15.2, 9.0 Hz, 1H)	2.15 (dd, J = 15.2, 9.0 Hz, 1H)	0.01	**14**	52.4	52.3	0.1
**9a,9b**	2.13 – 2.03 (m, 2H)	2.10 – 2.02 (m, 2H)	0.04*	**8**	46.8	46.7	0.1
**18a, 15b, 8**	1.99 – 1.86 (m, 3H)	1.97 – 1.84 (m, 3H)	0.02*	**5**	41.1	40.9	0.2
**18b**	1.79 – 1.72 (m, 1H)	1.79 – 1.62 (m, 2H)	0.01*	**6**	36.9	36.7	0.2
**16a**	1.72 – 1.63 (m, 1H)			**15**	27.3	27.2	0.1
**17b**	1.51 – 1.39 (m, 1H)	1.52 – 1.35 (m, 1H)	0.02*	**17**	23.7	23.6	0.1
**10**	1.23 (s, 3H)	1.22 (s, 3H)	0.01	**10**	22.3	22.1	0.2
**16b**	1.18 – 1.06 (m, 1H)	1.16 – 1.05 (m, 1H)	0.02*	**16**	21.1	21.0	0.1
**7**	1.03 (d, J = 7.0 Hz, 3H)	1.02 (d, J = 6.9 Hz, 3H)	0.01	**18**	20.4	20.2	0.2
				**9**	19.1	18.9	0.2
				**7**	18.4	18.3	0.1

*The differences in ^{1}H NMR shifts for multiples were calculated by using their centres, obtained by equation: centre = (left boundary + right boundary)/2.

^{1}H NMR (500 MHz, $CDCl_3$) of compound **9** – reported.

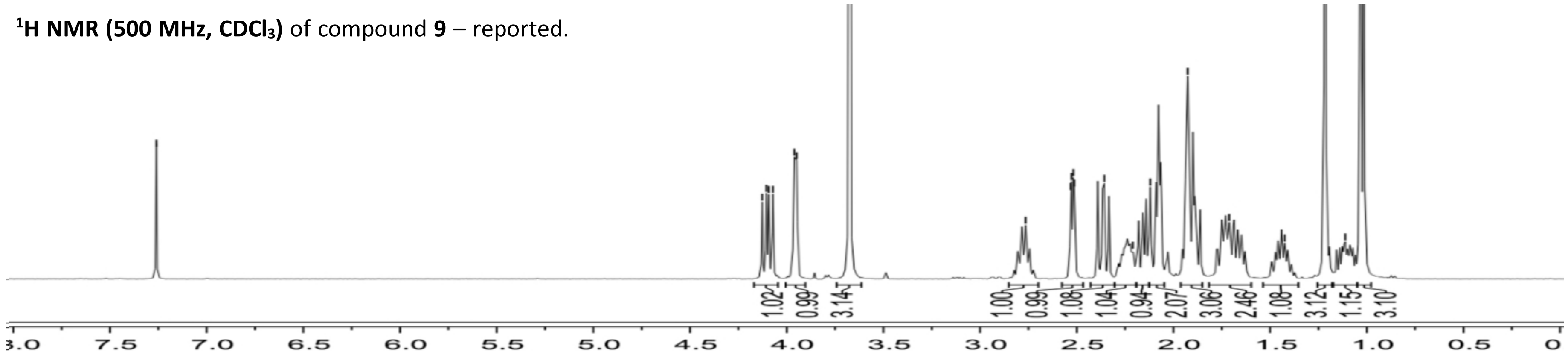

^{1}H NMR (500 MHz, $CDCl_3$) of compound **9.**

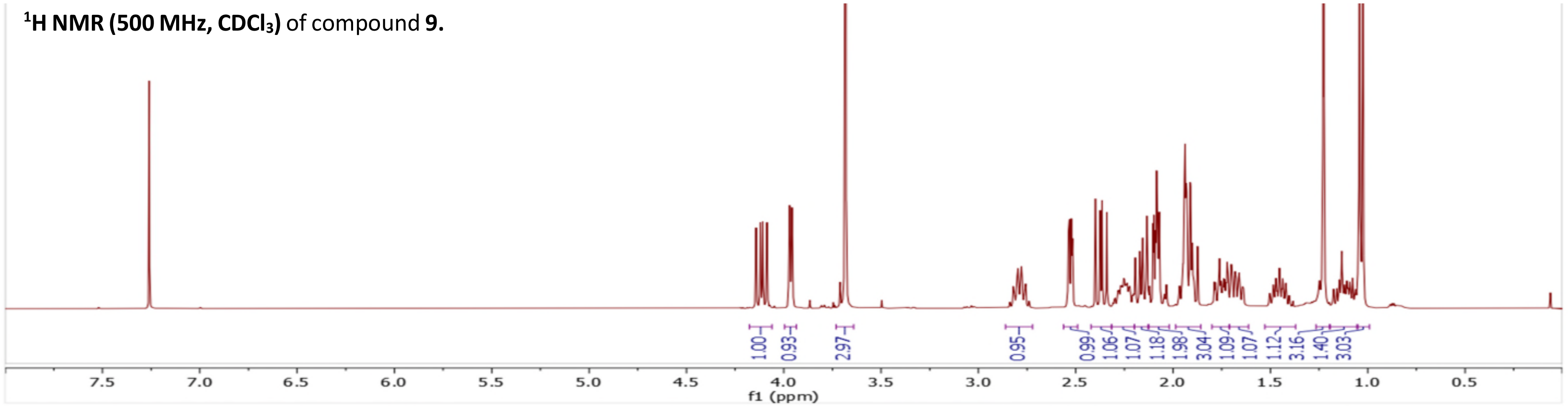

Figure S1. Comparison of ^{1}H NMR spectra of compound **9** prepared by Li's[32](top) and our group (bottom).

3.4.4 Introduction of the D-ring of himalensine A

Scheme S12. Installation of the D-ring.

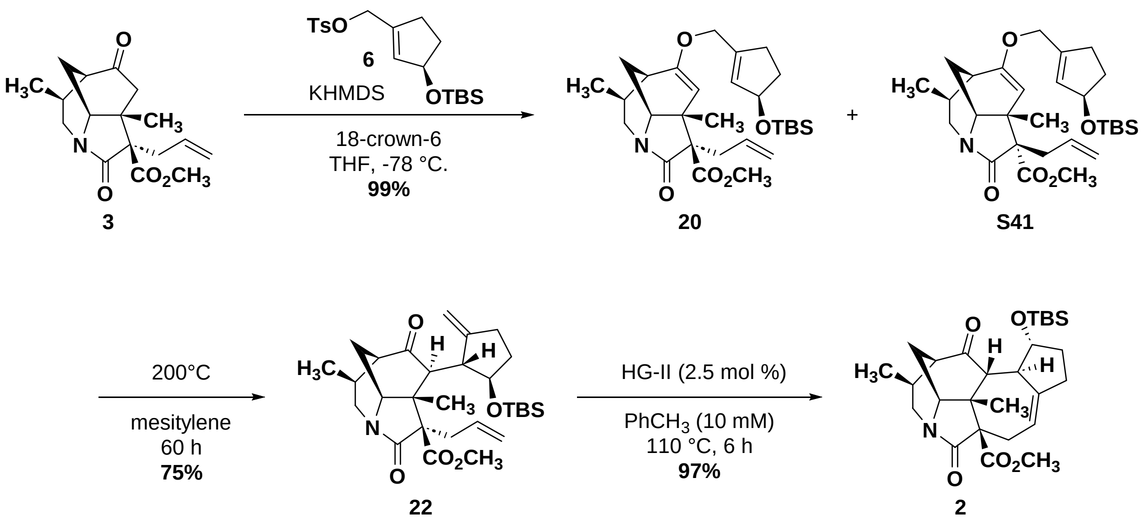

One pot synthesis of **2** from **20**

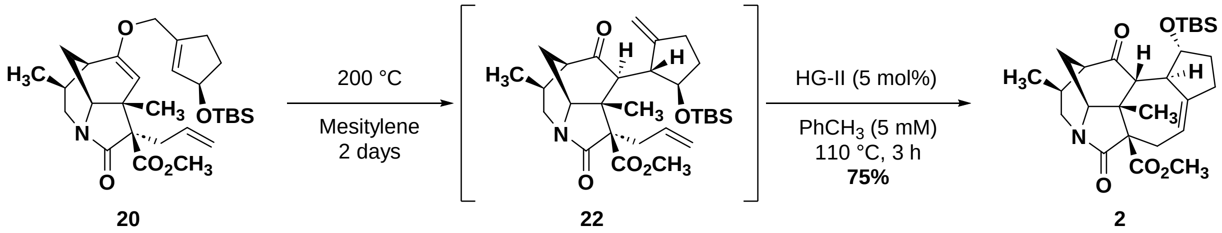

methyl (*3R,3aR,6R,7aS,8S*)-3-allyl-5-(((*R*)-3-((*tert*-butyldimethylsilyl)oxy)cyclopent-1-en-1-yl)methoxy)-3a,8-dimethyl-2-oxo-2,3,3a,6,7,7a-hexahydro-6,1-ethanoindole-3-carboxylate (20)

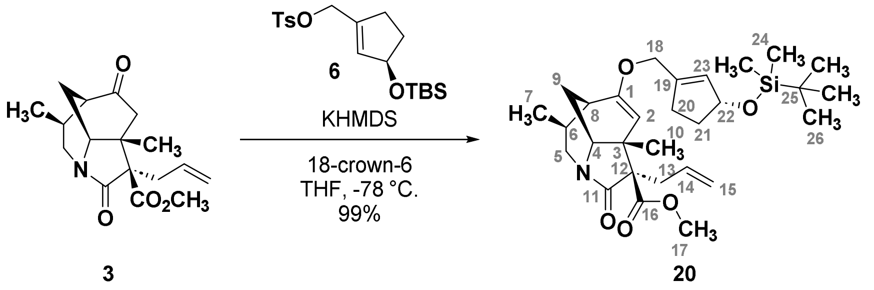

To a solution of ketone **3** (205.0 mg, 0.671 mmol, 1 equiv.) in degassed THF (4.0 mL) at -78 °C was added KHMDS (0.5 M solution in toluene, 2.0 mL, 1.01 mmol, 1.50 equiv.) and the resulting solution was stirred at this temperature for 20 min. Then, a solution of 18-crown-6 (177.4 mg, 0.671 mmol, 1.00 equiv.) in degassed THF (2.0 mL) followed by tosylate **6** (11.8 wt % solution in benzene, 2.61 g, 0.806 mmol, 1.20 equiv.) in THF (0.8 mL) were added. The solution was stirred for 1 h at this temperature and then warmed up slowly to 0 °C, which resulted in the formation of a white precipitate. The reaction mixture was then poured onto brine (20 mL) and extracted with ethyl acetate (4 x 20 mL). The combined organic phases were dried over Na_2SO_4, filtered and concentrated under reduced pressure. Purification by gradient column chromatography (ethyl acetate:pentane = 5:95 to 30:70) afforded compound **20** as a colorless oil (344 mg, 99%).

IR (thin film) $\tilde{\nu}_{max}$/cm^{-1}: 2951, 2929, 2856, 1731, 1697, 1649, 1417, 1216, 1195, 1167, 1061, 834, 775.

^{1}H NMR (400 MHz, $CDCl_3$) δ: 6.24 (dddd, J = 17.2, 10.2, 9.2, 4.8 Hz, 1H, H-14), 5.65 – 5.58 (m, 1H, H-23), 5.18 – 5.05 (m, 2H, H-15), 4.95 – 4.87 (m, 1H, H-22), 4.30 – 4.25 (m, 1H, H-2), 4.33 – 4.18 (m, 2H, H-18), 4.06 (dd, J = 13.7, 8.0 Hz, 1H, H-5a), 3.85 – 3.79 (m, 1H, H-4), 3.71 (s, 3H, H-17), 2.69 (ddt, J = 14.6, 4.8, 2.0 Hz, 1H, H-13a), 2.49 – 2.34 (m, 3H, H-13b, H-5b, H-20a), 2.37 – 2.24 (m, 1H, H-21a), 2.24 – 2.12 (m, 1H, H-20b), 2.10 – 1.97 (m, 1H, H-6), 1.95 (ddd, J = 13.7, 5.3, 2.3 Hz, 1H, H-9a), 1.93 – 1.86 (m, 1H, H-8), 1.79 – 1.66 (m, 1H, H-21b), 1.62 (ddt, J = 13.7, 3.8, 1.0 Hz, 1H, H-9b), 1.14 (s, 3H, H-10), 0.95 (d, J = 6.9 Hz, 3H, H-7), 0.89 (s, 9H, H-26), 0.07 (s, 6H, H-24).

^{13}C NMR (101 MHz, $CDCl_3$) δ: 173.9 (C-11), 170.9 (C-16), 159.6 (C-1), 143.1 (19), 135.3 (C-14), 130.6 (C-23), 117.6 (C-15), 95.0 (C-2), 78.0 (C-22), 65.9 (C-18), 62.0 (C-3), 56.9 (C-4), 52.1 (C-17), 50.0 (C-3), 41.6 (C-5), 37.5 (C-8), 35.7 (C-13), 34.2 (C-21), 31.9 (C-6), 31.2 (C-20), 26.1 (C-26), 20.1 (C-9), 19.6 (C-10), 19.5 (C-7), 18.5 (C-25), -4.42 (C-24), -4.44 (C-24).

HRMS (ES+): exact mass calculated for $[M+H]^+$ $(C_{29}H_{45}NO_5SiNa)^+$ requires 538.2961, found 538.2957.

$[\alpha]_D^{25}$= -59.1 (c= 1.00, $CHCl_3$).

methyl (3S,3aR,6R,7aS,8S)-3-allyl-5-(((*R*)-3-((*tert*-butyldimethylsilyl)oxy)cyclopent-1-en-1-yl)methoxy)-3a,8-dimethyl-2-oxo-2,3,3a,6,7,7a-hexahydro-6,1-ethanoindole-3-carboxylate (S39)

C12 Diastereomer was formed as a side product when the reaction was performed at room temperature.

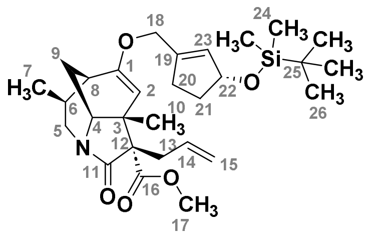

S41

^{1}H NMR (400 MHz, $CDCl_3$) δ: 5.83 (dddd, *J* = 16.8, 10.0, 7.6, 6.7 Hz, 1H, H-14), 5.62 – 5.54 (m, 1H, H-23), 5.15 – 5.03 (m, 2H, H-15), 4.95 – 4.87 (m, 1H, H-22), 4.17 (d, *J* = 12.4 Hz, 1H, H-18), 4.14 – 4.00 (m, 1H, H-5a), 4.07 (d, *J* = 13.3 Hz, 1H, H-18), 3.86 (d, *J* = 1.3 Hz, 1H, H-2), 3.73 (s, 3H, H-18), 3.69 (d, *J* = 4.1 Hz, 1H, H-4), 2.78 (ddt, *J* = 13.7, 7.6, 1.2 Hz, 1H, H-13a), 2.49 – 2.36 (m, 2H, H-13b, H-20a), 2.34 (dd, *J* = 13.6, 10.5 Hz, 1H, H-5b), 2.31 – 2.23 (m, 1H, H-21a), 2.23 – 2.13 (m, 1H, H-20b), 2.14 – 2.03 (m, 1H, H-6), 1.95 (ddd, *J* = 13.7, 5.2, 2.3 Hz, 1H, H-9a), 1.93 – 1.87 (m, 1H, H-8), 1.76 – 1.65 (m, 1H, H-21b), 1.62 (dd, *J* = 13.8, 3.7 Hz, 1H, H-9b), 1.20 (s, 3H, H-10), 0.93 (d, *J* = 6.9 Hz, 3H, H-7), 0.89 (s, 9H, H-26), 0.06 (s, 6H, H-24).

^{13}C NMR (101 MHz, $CDCl_3$) δ: 173.0 (C-11), 170.7 (C-16), 159.7 (C-1), 142.9 (C-19), 133.2 (C-14), 130.3 (C-23), 118.5 (C-15), 96.7 (C-2), 78.0 (C-22), 65.9 (C-18), 62.0 (C-12), 55.9 (C-4), 51.6 (C-17), 48.7 (C-3), 41.5 (C-5), 37.4 (C-8), 35.0 (C-13), 34.2 (C-21), 32.0 (C-6), 31.2 (C-20), 26.1 (-26), 20.2 (C-9), 19.5 (C-7), 18.4 (C-25), 17.4 (C-10), -4.42 (C-24), -4.44 (C-24).

HRMS (ES+): exact mass calculated for $[M+Na]^+$ $(C_{29}H_{45}NO_3SiNa)^+$ requires 538.2959, found 538.2975.

$[\alpha]_D^{25}$= -50.0 (c = 1.00, benzene).

methyl (3R,3aR,4S,6R,7aS,8S)-3-allyl-4-((*1R,2R*)-2-((*tert*-butyldimethylsilyl)oxy)-5-methylenecyclopentyl)-3a,8-dimethyl-2,5-dioxooctahydro-6,1-ethanoindole-3-carboxylate (22)

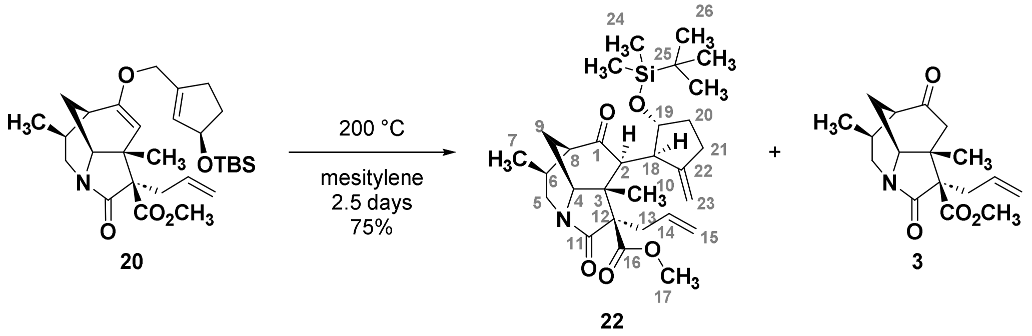

Compound **22** was prepared adopting procedure published by Carreira.[31]

Passivation of the glassware: A pressure tube or Schlenk tube with attached argon balloon was passivated by heating a solution of *N,O*-bis(trimethylsilyl) acetamide (0.1 mL) in hexane (10 mL) to 50 °C. The reaction vessel was swirled for 5 minutes so that all surface of the reaction vessel was in contact with the reagent. The solution was then discarded and tube washed with hexane (2 x 15 mL) and MTBE (2 x 15 mL) and dried under a high vacuum.

The solution of enol **20** (1.27 g, 2.46 mmol, 1 equiv.) was transferred to passivated pressure tube in benzene (5.0 mL), and the solvent was removed in a vacuum. The solvent-free enol was dissolved in mesitylene (78.0 mL, 0.032 M) (previously distilled and stored over molecular sieves). A PTFE syringe filter was used to ensure that no solid particles were transferred with the solvent into the reaction mixture. The resulting solution was degassed using freeze-pump-thaw (5 cycles), and the tube was backfilled with argon, sealed and heated to 200 °C for 60 h. After cooling to room temperature, the reaction mixture was directly poured onto a silica column and flushed with pentane until mesitylene was no longer eluted. Elution with ethyl acetate:pentane (10:90 to 25:75) afforded product **22** as a white amorphous solid (948 mg, 75%), unreacted starting material **20** (130 mg, 10%) and traces product of enol hydrolysis **3** (78 mg, 10%).

IR (thin film) $\tilde{\nu}_{max}$/cm^{-1}: 2954, 2929, 2856, 1737, 1704, 1430, 1289, 1255, 1208, 1088, 838.

^{1}H NMR (500 MHz, $CDCl_3$) δ: 6.24 (dtd, *J* = 17.3, 9.9, 3.7 Hz, 1H, H-14), 5.25 (dt, *J* = 17.4, 1.9 Hz, 1H, H-15a), 5.16 (dt, *J* = 10.2, 2.1 Hz, 1H, H-15b), 4.92 (s, 1H, H-23a), 4.87 (s, 1H, H-23b), 4.04 (d, *J* = 4.1 Hz, 1H, H-19), 4.02 (dd, J = 13.6, 9.0 Hz, 1H, H-5a), 3.92 (d, *J* = 6.1 Hz, 1H, H-4), 3.73 (s, 3H, H-17), 3.00 – 2.87 (m, 2H, H-13a, H-6), 2.65 (d, *J* = 8.8 Hz, 1H, H-18), 2.51 (d, *J* = 8.9 Hz, 1H, H-2), 2.41 – 2.30 (m, 2H, H-13b, H-9a), 2.29 – 2.20 (m, 3H, H-5b, H-21a, H-21b), 2.09 – 1.98 (m, 3H, H-8, H-9b, H-20), 1.60 – 1.50 (m, 1H, H-20b), 1.31 (s, 3H, H-10), 0.99 (d, *J* = 7.0 Hz, 3H, H-7), 0.83 (s, 9H, H-36), 0.01 (s, 3H, H-24), 0.01 (s, 3H, H-24).

^{13}C NMR (126 MHz, CDCl$_3$) δ: 212.8 (C-1), 171.3 (C-16), 171.2 (C-11), 152.6 (C-22), 134.4 (C-14), 118.7 (C-15), 110.5 (C-23), 78.4 (C-19), 62.8 (C-12), 60.8 (C-4), 54.8 (C-18), 54.1 (C-2), 52.5 (C-17), 51.6 (C-3), 45.6 (C-8), 41.2 (C-5), 36.8 (C-6), 34.8 (C-13), 32.0 (C-20), 28.8 (C-21), 25.8 (C-26), 18.6 (C-9), 18.2 (C-10), 18.0 (C-25), 17.8 (C-7), -4.2 (C-24), -4.3 (C-24).

HRMS (ES+): exact mass calculated for $[M+H]^+$ ($C_{29}H_{45}NO_5Si^{23}Na)^+$ requires 538.2975, found 538.2957.

$[\boldsymbol{\alpha}]_{\boldsymbol{D}}^{\mathbf{25}}$= -46.9 (c = 1.00, CHCl$_3$).

methyl (*2R,3S,6aR,11R,11aR,12S,12aR,12bS*)-11-((tert-butyldimethylsilyl)oxy)-3,12a-dimethyl-6,13-dioxo-1,2,3,4,7,9,10,11,11a,12,12a,12b-dodecahydro-2,12-methanoazuleno[5,6-a]indolizine-6a(*6H*)-carboxylate (2)

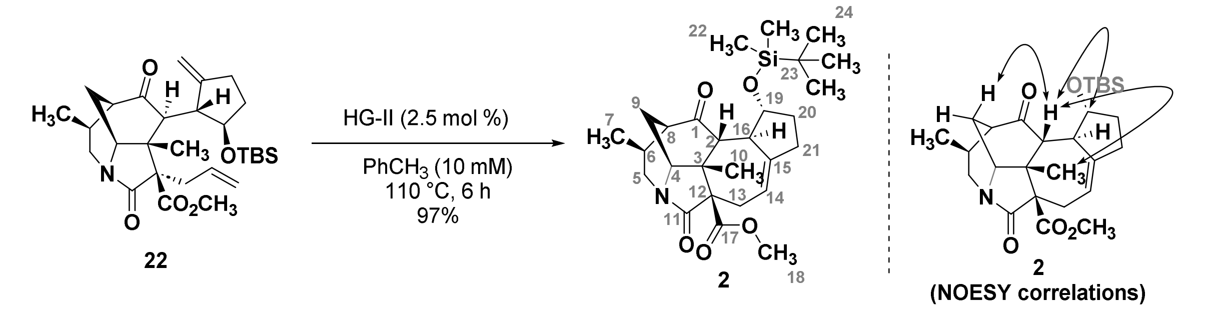

To a solution of diene **22** (10 mM, 950 mg, 1.84 mmol, 1 equiv.) in dry toluene (185 mL) degassed by a stream of argon (30 min) was added Grubbs-Hoveyda 2nd generation catalyst (28.9 mg, 46.0 µmol, 2.5 mol%) and the reaction mixture was heated to reflux with attached condenser while gently bubbled with argon. After stirring for 6 h, the reaction mixture was cooled down, the catalyst was quenched by the addition of potassium isocyanoacetate (110 mg, 0.92 mmol, 0.50 equiv.) in methanol (10 mL) and the reaction mixture was stirred for another 30 min. Volatiles were removed under reduced pressure, and the crude product was purified by column chromatography (dry loading, ethyl acetate:pentane = 35:65). The product of cyclization **2** was obtained as a white solid (871 mg, 97%).

NOTE: The configuration of C2 was determined using NOESY, observed corelations and indicated by the arrows.

MP: 182 – 188 °C (>99% ee).

^{1}H NMR (500 MHz, $CDCl_3$) δ: 5.75 – 5.65 (m, 1H, H-14), 4.05 (dd, *J* = 13.5, 8.8 Hz, 1H, H-5a), 3.91 (d, *J* = 5.3 Hz, 1H, H-4), 3.68 (s, 3H, H-18), 3.60 (td, *J* = 8.3, 5.0 Hz, 1H, H-19), 3.55 (dd, *J* = 7.7, 3.6 Hz, 1H, H-16), 3.06 – 2.95 (m, 1H, H-6), 2.57 (dd, *J* = 14.6, 7.2 Hz, 1H, H-13a), 2.51 (d, *J* = 3.8 Hz, 1H, H-2), 2.44 (dd, *J* = 16.3, 7.4 Hz, 1H, H-21a), 2.31 (dd, *J* = 13.5, 9.7 Hz, 1H, H-5b), 2.26 – 2.12 (m, 2H, H-9a, H-21b), 2.14 – 2.07 (m, 1H, H9b), 2.11 – 2.02 (m, 1H, H-13b), 1.90 – 1.85 (m, 1H, H-8), 1.89 – 1.77 (m, 1H, H-20a), 1.61 – 1.49 (m, 1H, H-20b), 1.18 (s, 3H, H-10), 1.03 (d, *J* = 7.0 Hz, 3H, H-7), 0.81 (s, 9H, H-24), 0.01 (s, 6H, H-22).

^{13}C NMR (126 MHz, $CDCl_3$) δ: 211.2 (C-10), 172.1 (C-11), 171.5 (C-17), 142.3 (C-15), 117.5 (C-14), 83.7 (C-19), 61.8 (C-4), 59.1 (C-12), 55.1 (C-2), 53.4 (C-3), 52.3 (C-18), 47.1 (C-8), 44.4 (C-16), 41.3 (C-5), 35.4 (C-6), 33.9 (C-20), 29.3 (C-13), 29.1 (C-21), 25.8 (C-24), 23.1 (C-10), 19.4 (C-9), 17.9 (C-23), 17.8 (C-7), -4.2 (C-22), -4.7 (C-22).

IR (thin film) $\tilde{\nu}_{max}$/cm^{-1}: 2954, 2929, 2857, 1739, 1713, 1693, 1429, 1288, 1133, 837, 775, 732.

HRMS (ES+): exact mass calculated for $[M+H]^+$ $(C_{27}H_{42}NO_5Si)^+$ requires 488.2827, found 488.2825.

$[\alpha]^{25}_{D}$= -60.4 (c = 1.00, $CHCl_3$).

One-pot procedure for synthesis of compound 2 from compound 20

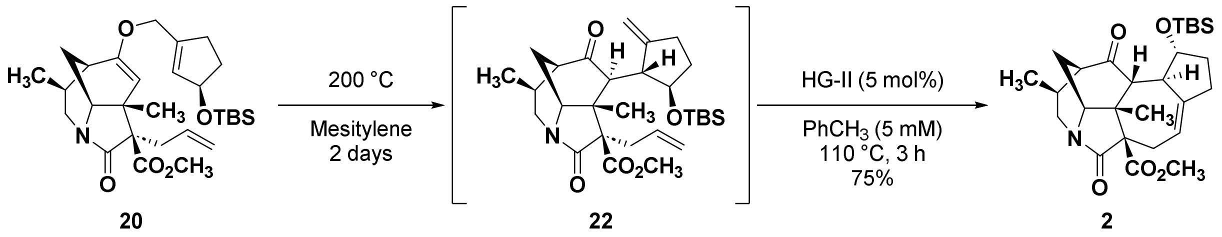

Claisen rearrangement and ring-closing metathesis can be telescoped using the reaction conditions described above.

A solution of enol ether **20** (100 mg, 0.194 mmol, 32 mM, 1 equiv.) in mesitylene (6.1 mL) was heated for 2 days at 200 °C. The reaction mixture was then diluted with dry, degassed toluene (32.7 mL) to 5 mM concentration and, after the addition of Grubbs-Hoveyda 2nd generation catalyst (6.1 mg, 9.69 µmol, 5 mol%), heated to 125 °C for 3 h while gently bubbled with argon. The catalyst was then quenched with potassium isocyanoacetate (23.0 mg, 0.194 mmol, 1.00 equiv.) in methanol (5 mL), and the reaction mixture worked up as described above. Product **2** was obtained in unchanged yield as white solid (70.5 mg, 75%).

3.4.5 The endgame of the synthesis of himalensine A

Scheme S13. The endgame of the synthesis of himalensine A.

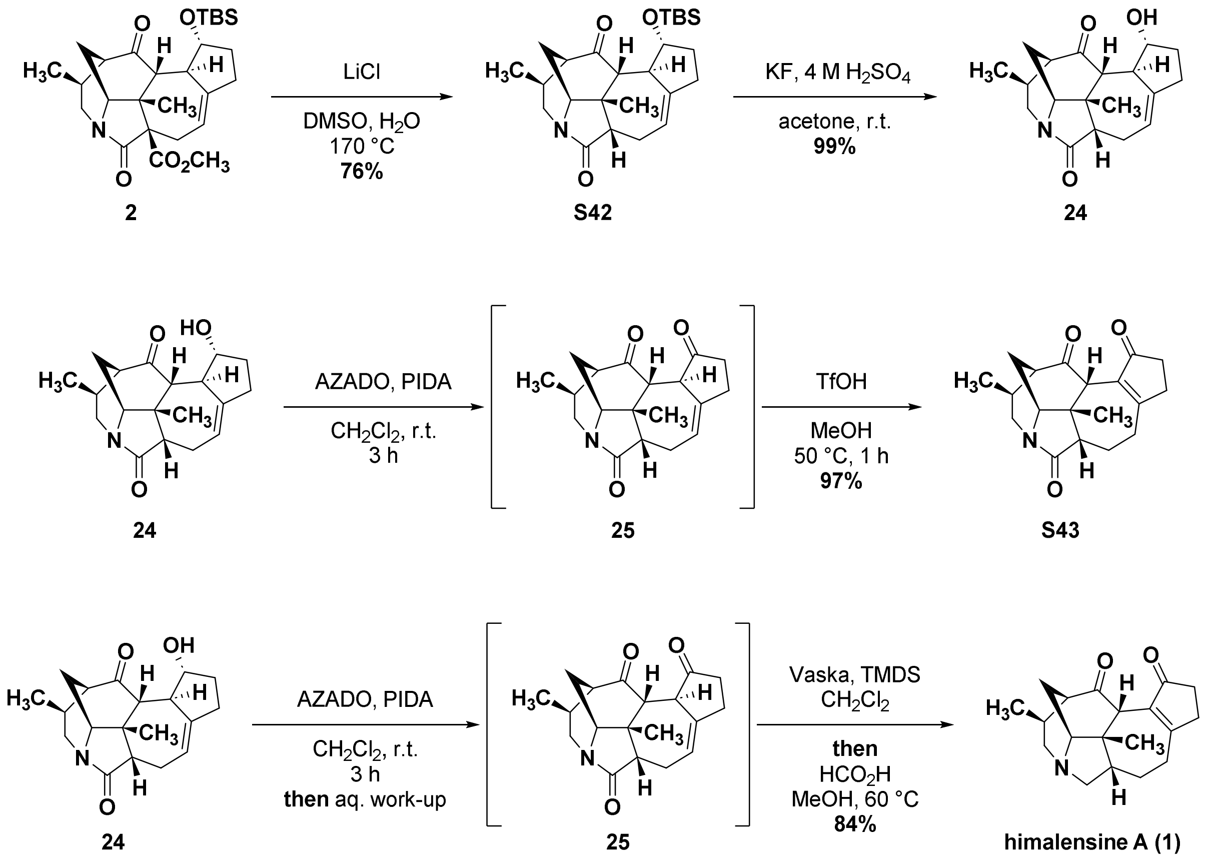

(*2R,3S,6aS,11R,11aR,12R,12aR,12bS*)-11-((*tert*-butyldimethylsilyl)oxy)-3,12a-dimethyl-1,3,4,6a,7,9,10,11,11a,12,12a,12b-dodecahydro-2,12-methanoazuleno[5,6-a]indolizine-6,13(*2H*)-dione (S42)

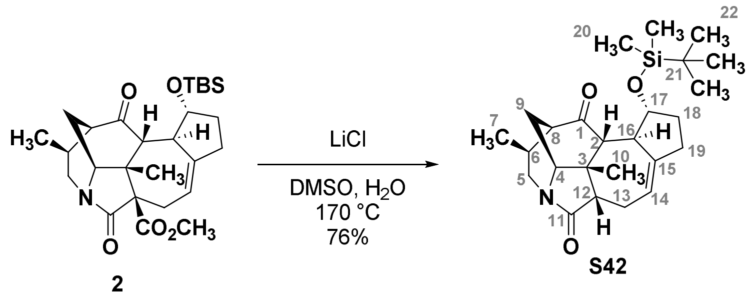

Compound **S42** was prepared adopting the procedure published by Dixon.[33] To a solution of compound **2** (162 mg, 332 µmol, 1 equiv.) in DMF (4.6 mL) was added LiCl (140.8 mg, 3.32 mmol, 10.0 equiv.) and water (18 uL, 996 µmol, 3.00 equiv.), the reaction mixture was purged with nitrogen and heated to 170 °C with an attached reflux condenser. After stirring for 2 h, the reaction mixture was allowed to cool down, poured onto half-saturated brine and extracted with ether (5 x 20 mL). The combined organic layers were dried over anhydrous Na_2SO_4, filtered and concentrated under reduced pressure. Purification by column chromatography (dry loading, ethyl acetate:pentane = 40:60 to 50:50) afforded the product of decarboxylation **S42** as a white solid (108.5 mg, 76%).

MP: 112 – 114 °C (>99% ee).

IR (neat) $\tilde{\nu}_{max}$/cm^{-1}: 2959, 2946, 2926, 2891, 2877, 2854, 1711, 1689, 1421, 1095, 867, 835, 773.

^{1}H NMR (500 MHz, $CDCl_3$) δ: 5.54 (tq, *J* = 7.2, 2.3 Hz, 1H, H-14), 4.01 (dd, J = 13.5, 8.8 Hz, 1H, H-5a), 3.60 (td, J = 6.3, 4.8 Hz, 1H, H-17), 3.54 (d, J = 5.4 Hz, 1H, H-4), 3.51 – 3.45 (m, 1H, H-16), 2.96 (dt, J = 16.4, 8.1 Hz, 1H, H-6), 2.54 – 2.44 (m, 1H, H-19a), 2.36 (dt, J = 14.1, 7.3 Hz, 1H, H-13a), 2.28 (ddd, J = 11.8, 7.2, 1.0 Hz, 1H, H-12), 2.25 – 2.10 (m, 5H, H-13b, H-5b, H-9a, H-19b, H-2), 2.04 (ddd, J = 14.6, 5.4, 1.3 Hz, 1H, H-9b), 1.87 (d, *J* = 3.7 Hz, 1H, H-8), 1.82 – 1.71 (m, 1H, H-18a), 1.58 (dq, *J* = 11.8, 7.4 Hz, 1H, H-18b), 1.27 (s, 3H, H-10), 1.01 (d, J = 6.9 Hz, 3H, H-7), 0.82 (s, 9H, H-22), 0.03 (s, 3H, H-20), 0.00 (s, 3H, H-20).

^{13}C NMR (126 MHz, $CDCl_3$) δ: 211.7 (C-1), 175.6 (C-11), 145.5 (C-15), 117.0 (C-14), 82.6 (C-17), 62.5 (C-4), 54.5 (C-2), 50.4 (C-3), 46.8 (C-8), 46.6 (C-12), 45.2 (C-16), 40.9 (C-5), 35.9 (C-6), 33.9 (C-18), 29.4 (C-19), 27.2 (C-10), 25.8 (C-22), 25.5 (C-13), 18.9 (C-9), 18.0 (C-21), 17.9 (C-7), -4.4 (C-20), -4.6 (C-20).

HRMS (ES+): exact mass calculated for $[M+H]^+$ $(C_{25}H_{40}NO_3Si)^+$ requires 430.2772, found 430.2771.

$[\alpha]_D^{25}$= -96.9 (c = 1.00, $CHCl_3$).

(*2R,3S,6aS,11R,11aR,12R,12aR,12bS*)-11-hydroxy-3,12a-dimethyl-1,3,4,6a,7,9,10,11,11a,12,12a,12b-dodecahydro-2,12-methanoazuleno[5,6-a]indolizine-6,13(*2H*)-dione (24)

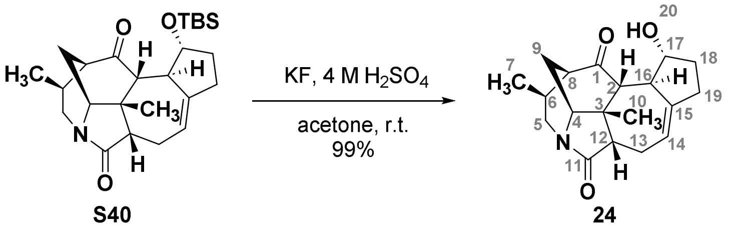

To a solution of protected alcohol **S42** (50.0 mg, 116 µmol, 1 equiv.) in acetone (1.5 mL) was added KF (33.8 mg, 582 µmol. 5.00 equiv.) and 4 M aq. solution of H_2SO_4 (204 µL, 815 µmol. 7.00 equiv.) and the resulting mixture was stirred for 7.5 h. The reaction mixture was then quenched with sat. aq. solution of Na_2CO_3 (10 mL) and extracted with CH_2Cl_2 (4 x 10 mL). The combined organic phases were dried over anhydrous Na_2SO_4, filtered and concentrated under reduced pressure. Purification by column chromatography (CH_3CN:CH_2Cl_2 = 60:40) afforded alcohol **24** as a white solid (36.5 mg, 99%).

MP: 220 – 224 °C (decomp., >99% ee).

^{1}H NMR (500 MHz, CDCl$_3$) δ: 5.63 – 5.56 (m, 1H, H-14), 4.01 (dd, *J* = 13.6, 8.9 Hz, 1H, H-5a), 3.67 (q, *J* = 6.3 Hz, 1H, H-17), 3.52 (d, *J* = 5.6 Hz, 1H, H-4), 3.26 (ddt, *J* = 7.9, 5.3, 2.6 Hz, 1H, H-16), 2.96 – 2.85 (m, 1H, H-6), 2.53 – 2.43 (m, 2H, H-20, H-19a), 2.39 (dt, *J* = 13.7, 7.8 Hz, 1H, H-13a), 2.34 – 2.27 (m, 1H, H-12), 2.27 – 2.14 (m, 4H, H-13b, H-5b, H-19b, H-2), 2.15 (dd, *J* = 14.6, 4.5 Hz, 1H, H-9a), 2.05 (ddd, *J* = 14.7, 5.7, 1.4 Hz, 1H, h-9b), 2.02 – 1.99 (m, 1H, H-8), 1.96 (tt, *J* = 10.1, 3.7 Hz, 1H), 2.00 – 1.91 (m, 1H, H-18a), 1.62 (ddt, *J* = 12.3, 9.5, 7.2 Hz, 1H, H-18b), 1.28 (s, 3H, H-10), 1.04 (d, *J* = 6.9 Hz, 3H, H-7).

^{13}C NMR (101 MHz, CDCl$_3$) δ: 214.5 (C-1), 175.2 (C-11), 146.0 (C-15), 117.5 (C-14), 83.1 (C-17), 62.1 (C-4), 55.7 (C-2), 50.7 (C-3), 46.6 (C-8), 46.5 (C-12), 46.4 (C-16), 40.6 (C-5), 36.9 (C-6), 34.1 (C-18), 30.2 (C-19), 27.1 (C-10), 25.1 (C-13), 18.2 (C-9), 18.0 (C-7).

IR (thin film) $\tilde{\nu}_{max}$/cm^{-1}: 3398, 2953, 2926, 2870, 1703, 1668, 3432, 1290, 909.

HRMS (ES+): exact mass calculated for $[M+H]^+$ $(C_{19}H_{26}NO_3)^+$ requires 316.1907, found 316.1906.

$[\alpha]_D^{25}$= -126.3 (c = 1.00, CHCl$_3$)

(2R,3S,6aS,12S,12aR,12bR)-3,12a-dimethyl-1,2,3,4,6a,7,8,9,10,12,12a,12b-dodecahydro-2,12-methanoazuleno[5,6-a]indolizine-6,11,13-trione (S43)

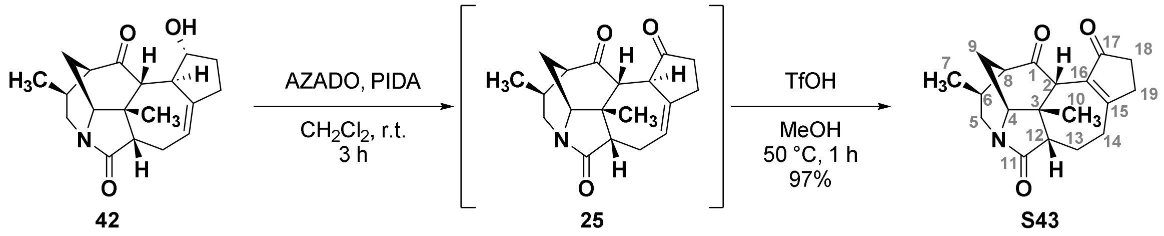

To a solution of alcohol **24** (20.0 mg, 63.4 µmol, 1 equiv.) in CH_2Cl_2 (1.2 mL) was added AZADO (1.0 mg, 6.34 µmol, 10 mol%) fallowed by PIDA (73.6 mg, 253 µmol, 4.00 equiv.) and the resulting solution was stirred for 3 h. *i*-PrOH (200 µL) was then added to quench the excess of PIDA. After stirring for 30 min, the reaction mixture was diluted with MeOH (0.5 mL), acidified with TfOH (50 µL) and stirred for another 1 h at 50 °C. The reaction mixture was then poured onto sat. aq. solution of $NaHCO_3$ (10 mL), extracted with CH_2Cl_2 (3 x 10 mL). The combined organic phases were dried over anhydrous Na_2SO_4, filtered and concentrated under reduced pressure. Purification by column chromatography (CH_3CN:CH_2Cl_2 = 50:50) afforded oxy-himalensine A as a white solid (19.5 mg, 97%). The spectroscopic data are in agreement with the literature values.[34]

NOTE: Double bond tends to partially migrate on silica gel, however, for full migration, heating with acid in the protic solvent is required.

MP: >240 °C (>99% ee).

^{1}H NMR (500 MHz, CDCl$_3$) δ: 4.08 (dd, J = 13.6, 9.0 Hz, 1H, H-5a), 3.73 – 3.68 (m, 2H, H-4, H-2), 3.03 (td, J = 8.6, 6.3 Hz, 1H, H-6), 2.81 – 2.72 (m, 1H, H-14a), 2.72 – 2.59 (m, 2H, H-21a, H-21b), 2.55 (t, J = 4.4 Hz, 1H, H-12), 2.51 – 2.39 (m, 2H, H-22a, H-22b), 2.42 – 2.35 (m, 1H, H-9a), 2.29 – 2.20 (m, 2H, H-14a, H-5b), 2.20 – 2.11 (m, 2H, H-13a, H-9b), 2.04 (d, J = 4.6 Hz, 1H, H-8), 1.65 (dddd, J = 14.7, 12.9, 4.1, 1.9 Hz, 1H, H-13b), 1.25 (s, 3H, H-10), 1.03 (d, J = 7.0 Hz, 3H, H-7).

^{13}C NMR (126 MHz, CDCl$_3$) δ: 208.7 (C-1), 208.5 (C-17), 182.0 (C-15), 173.4 (C-11), 134.2 (C-16), 61.9 (C-4), 51.7 (C-12), 50.3 (C-2), 49.1 (C-3), 46.3 (C-8), 40.6 (C-5), 36.2 (C-6), 34.6 (C-18), 31.9 (C-19), 28.2 (C-14), 23.7 (C-10), 20.9 (C-13), 19.6 (C-9), 17.7 (C-7).

HRMS (ES+): exact mass calculated for $[M+H]^+$ $(C_{19}H_{24}NO_3)^+$ requires 314.1751, found 314.1750.

IR (thin film) $\tilde{\nu}_{max}$/cm^{-1}: 2956, 2925, 2871, 1689, 1654, 1433, 1289, 1258, 918, 734.

$[\alpha]_D^{25}$= -70.1 (c = 0.10, $CHCl_3$), literature values: $[\alpha]_D^{25}$= -69.8 (c = 0.1, $CHCl_3$),[34] $[\alpha]_D^{25}$= -67.4 (c = 0.2, MeOH),[35] $[\alpha]_D^{25}$= -65 (c = 0.12, $CHCl_3$).[36]

(2R,3S,6aS,12S,12aS,12bR)-3,12a-dimethyl-1,3,4,6,6a,7,8,9,10,12,12a,12b-dodecahydro-2,12-methanoazuleno[5,6-a]indolizine-11,13(2H)-dione (himalensine A)

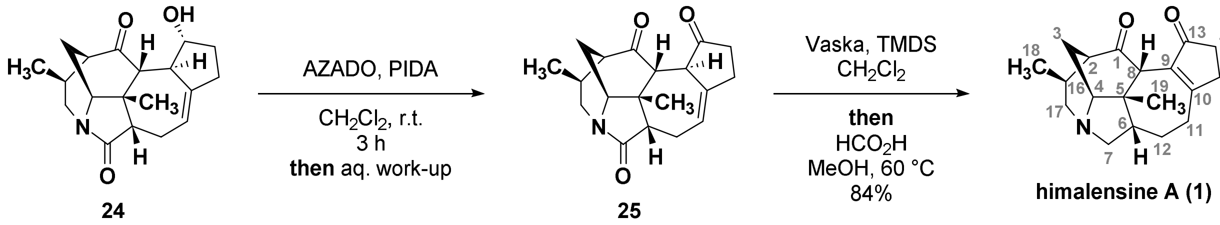

Reduction of intermediate **25** was accomplished by adopting the procedure previously reported by our group.[34]

To a solution of alcohol **24** (6.0 mg, 19.0 µmol, 1 equiv.) in CH_2Cl_2 (0.3 mL) was added AZADO (0.2 mg, 1.52 µmol, 8 mol%) fallowed by PIDA (22.1 mg, 4.00 equiv.) and the resulting solution was stirred for 3 h. The reaction mixture was then poured onto sat. aq. solution of $Na_2S_2O_3$ (7 mL) and extracted with CH_2Cl_2 (4 x 7 mL). The combined organic phases were dried over anhydrous Na_2SO_4, filtered and concentrated under reduced pressure.

The obtained residue was dissolved in toluene (0.5 mL), and Vaska's catalyst (1.5 mg. 1.90 µmol, 0.10 equiv.) was added. TMDS (33.6 µL, 190 µmol, 10.0 equiv.) was added dropwise and the reaction was monitored by TLC after the addition of every drop (evolution of gas was observed). After full consumption of intermediate **25**, formic acid (2 mL) and MeOH (0.5 mL) were added, and the resulting mixture was heated to 60 °C for 22 h. The reaction mixture was then diluted with distilled water (10 mL) and basified by the careful addition of solid K_2CO_3. The resulting mixture was extracted with CH_2Cl_2 (4 x 10 mL), and the combined organic phases were dried over anhydrous Na_2SO_4, filtered and concentrated under reduced pressure. Purification by column chromatography (MeOH:$CHCl_3$:Et_3N 0:98:2 to 0.2:97.8:2) afforded himalensine A **(1)** as a yellowish foam (4.8 mg, 84%).

NOTE: It was reported,[34] that NMR shifts of himalensine A depend on the purification method, solvent and time after purification. Residual acid from silica/chloroform is causing partial protonation of himalensine A and shift of the peaks in ^1H NMR downfield. ^1H NMR spectrum of our synthetic sample is identical to ^1H NMR spectrum of a free-base form of himalensine A reported by Shi.[34]

^1H NMR (600 MHz, $CDCl_3$) δ: 3.63 (s, 1H, H8), 3.45 (d, *J* = 4.7 Hz, 1H, H-4), 2.86 (dd, *J* = 9.0, 6.7 Hz, 1H, H-7), 2.83 – 2.71 (m, 3H, H-7a, H-16a, H-17a), 2.71 – 2.67 (m, 1H, H-15a), 2.66 – 2.57 (m, 2H, H-15b, H-11a), 2.52 (dd, *J* = 13.4, 9.2 Hz, 1H, H-17b), 2.50 – 2.45 (m, 1H, H-14a), 2.42 (ddd, *J* = 18.9, 6.5, 2.9 Hz, 1H, H-14b), 2.30 – 2.24 (m, 1H, H-6), 2.24 – 2.18 (m, 1H, H-11b), 2.11 (dd, *J* = 14.5, 4.1 Hz, 1H, H-3a), 2.06 (ddd, *J* = 14.6, 4.7, 1.8 Hz, 1H, H-3b), 2.03 – 1.99 (m, 1H, H-2), 1.83 – 1.75 (m, 2H, H-12a, H-12b), 1.11 (s, 3H, H-19), 0.97 (d, *J* = 6.6 Hz, 3H, H-18).

^{13}C NMR (151 MHz, $CDCl_3$) δ: 214.5 (C-1), 208.7 (C-13), 179.9 (C-10), 135.9 (C-9), 65.0 (C-4), 54.9 (C-7), 51.6 (C-8), 49.6 (C-17), 48.9 (C-6), 48.3 (C-5), 46.1 (C-2), 34.6 (C-14), 32.0 (C-15), 31.9 (C-16), 28.1 (11), 26.2 (C-19), 22.9 (C-12), 20.4 (3), 19.1 (C-1).

HRMS (ES+): exact mass calculated for $[M+H]^+$ $(C_{19}H_{26}NO_2)^+$ requires 300.1958, found 300.1956.

IR (thin film) $\tilde{\nu}_{max}$/cm^{-1}: 2953, 2918, 2867, 2849, 1699, 1657, 1440, 1280, 1255.

$[\alpha]_D^{25}$= -91.9 (c = 0.10, MeOH), literature values: $[\alpha]_D^{25}$= -44.0 (c = 0.10, MeOH),[37] $[\alpha]_D^{25}$= -74.0 (c = 0.12, MeOH),[34] $[\alpha]_D^{25}$= -71.2 (c = 0.1, MeOH),[35] $[\alpha]_D^{25}$= -55 (c = 0.03, $CHCl_3$).[36]

Table S6. ^{1}H NMR ($CDCl_3$) data comparison of isolated and synthetic himalensine A.

Atom		Synthesized (600 MHz)	Isolated[37]	Diff.	Synthesized (Shi)[34] (700 MHz)	Diff.
**1**		-	-	-	-	-
**2**		2.01, m	2.04, brs	-0.03	2.02, app dp (4.2, 1.2 Hz)	-0.01
**3**	α	2.11, dd (14.5, 4.1 Hz)	2.14, dd (14.8, 3.5)	-0.03	2.11, dd (14.7, 4.4 Hz)	0.00
	β	2.06, ddd (14.6, 4.7, 1.8 Hz)	2.08 (br dd (14.8, 4.9)	-0.02	2.06, ddd (14.7, 5.0, 1.8 Hz)	0.00
**4**		3.45, d (4.7 Hz)	3.52, br s	-0.07	3.46, d (5.2 Hz)	-0.01
**5**		-	-	-	-	-
**6**		2.27, m	2.30, m	-0.03	2.29 – 2.20, m	0.02*
**7**	α	2.81, m	2.80, m	0.01	2.84 – 2.73, m	0.02*
	β	2.86 (dd, *J* = 9.0, 6.7 Hz, 1H)	2.92, m	-0.06	2.87, dd (9.0, 6.7 Hz)	-0.01
**8**		3.63, s	3.65, s	-0.02	3.63, s	0.00
**9**		-	-	-	-	-
**10**		-	-	-	-	-
**11**	α	2.62, m	3.65, s	-0.03	2.68 – 2.55, m	0.00*
	β	2.21, m	2.23, m	-0.02	2.29 – 2.20, m	-0.04*
**12**		1.80, m	1.80, m	0.00	1.81 – 1.78, m	0.00*
**13**		-	-	-	-	-
**14**	α	2.48, m	2.49, ddd (18.9, 6.5, 2.5)	-0.01	2.48, m	0.00
	β	2.42, ddd (18.9, 6.5, 2.9 Hz)	2.43, ddd (18.9, 6.5, 2.5)	-0.01	2.42, ddd (18.8, 6.7, 2.6 Hz)	0.00
**15**	α	2.69, m	2.71, m	-0.02	2.84 – 2.73, m	-0.01*
	β	2.65, m	2.64, m	0.01	2.68-2.55, m	0.03*
**16**		2.76, m	2.74, m	0.02	2.84 – 2.73, m	-0.03*
**17**	α	2.71, m	2.82, dd (14.1, 7.1)	-0.09	2.70, dddd (18.8, 6.9, 2.6, 1.2 Hz)	0.01
	β	2.52, dd (13.4, 9.2 Hz)	2.56, dd (14.1, 10.3)	-0.04	2.52, dd (14.1, 9.1 Hz)	0.00
**18**		0.97, d (6.6 Hz)	0.99, d (6.8)	-0.02	0.97, d (6.8 Hz)	0.00
**19**		1.11, s	1.12, s	-0.01	1.11, s	0.00

NOTE: It was reported,[34] that NMR shifts of himalensine A depend on the purification method, solvent and time after purification. Residual acid from silica/chloroform is causing partial protonation of himalensine A and shift of the peaks in ^{1}H NMR downfield. ^{1}H NMR spectrum of our synthetic sample is identical to ^{1}H NMR spectrum of a free-base form of himalensine A reported by Shi.[34] The centres of multiplets in ^{1}H NMR spectrum were determined using HSQC. *The differences in ^{1}H NMR shifts for multiples were calculated by using their centres, obtained by equation: centre = (left boundary + right boundary)/2.

Table S7. ^{13}C NMR ($CDCl_3$) data comparison of isolated and synthetic himalensine A.

Atom	Synthesized (151 MHz)	Isolated[37]	Diff.	Synthesized (Shi)[34] (175 MHz)	Diff
**1**	214.5	214.4	0.1	214.5	0.0
**2**	46.1	46.1	0.0	46.1	0.0
**3**	20.4	20.3	0.1	20.4	0.0
**4**	65.0	65.1	-0.1	65.0	0.0
**5**	48.3	48.3	0.0	48.3	0.0
**6**	48.9	48.8	0.1	48.9	0.0
**7**	54.9	54.9	0.0	54.9	0.0
**8**	51.6	51.7	-0.1	51.7	-0.1
**9**	135.9	135.8	0.1	135.9	0.0
**10**	179.9	179.9	0.0	179.9	0.0
**11**	28.1	28.1	0.0	28.1	0.0
**12**	22.9	22.9	0.0	23.0	-0.1
**13**	208.7	208.7	0.0	208.7	0.0
**14**	34.6	34.6	0.0	34.6	0.0
**15**	32.0	32.0	0.0	32.0	0.0
**16**	31.9	31.8	0.1	31.9	0.0
**17**	49.6	49.6	0.0	49.6	0.0
**18**	19.1	19.2	-0.1	19.1	0.0
**19**	26.2	26.3	-0.1	26.3	-0.1

NOTE: It was reported,[34] that NMR shifts of himalensine A depend on the purification method, solvent and time after purification. Residual acid from silica/chloroform is causing partial protonation of himalensine A and shift of the peaks in ^{1}H NMR downfield. ^{1}H NMR spectrum of our synthetic sample is identical to ^{1}H NMR spectrum of a free-base form of himalensine A reported by Shi.[34]

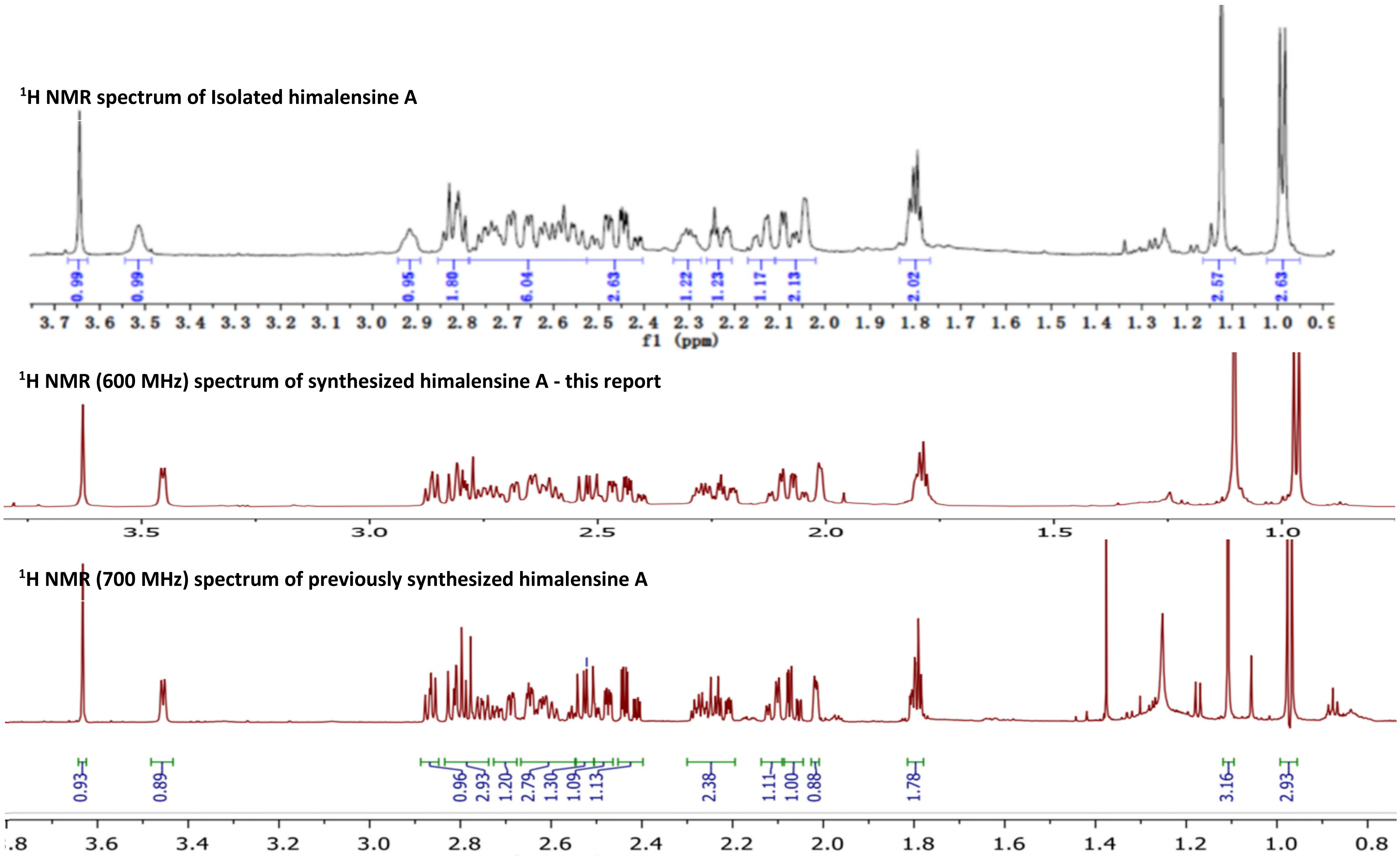

Figure S2. Comparison of ^{1}H NMR spectra of isolated[37] (top), synthesized – this work (middle) and previously synthesized[34] (bottom) himalensine A.

3.4.6 Unsuccessful strategy towards himalensine A through intermediate 23:

Scheme S14. Unsuccessful strategy towards himalensine A through intermediate **23**.

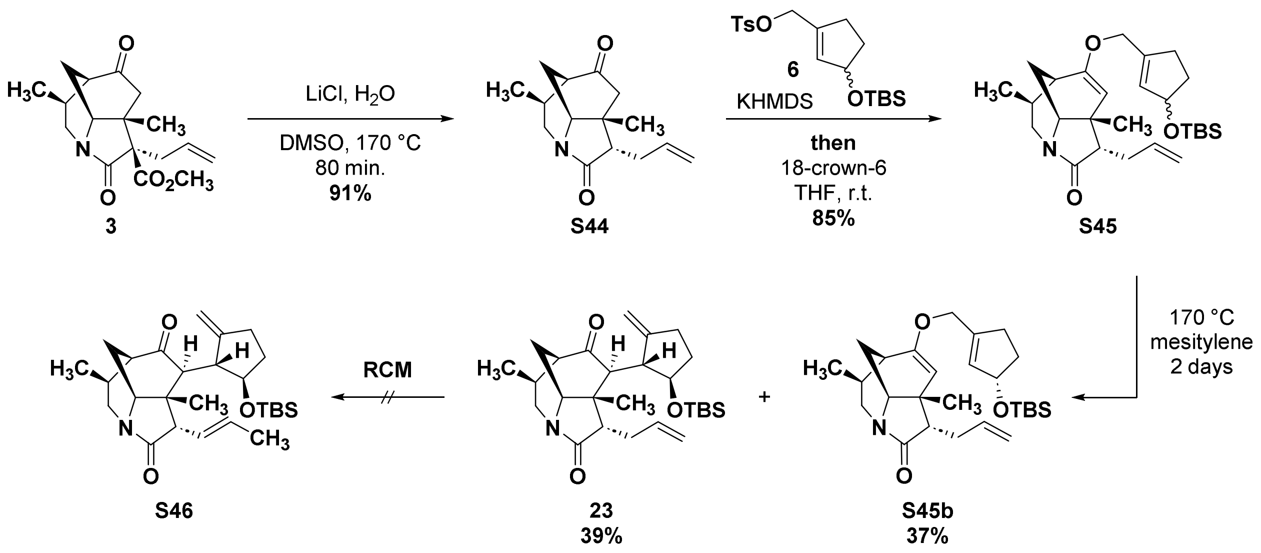

(*3S,3aR,6R,7aR,8S*)-3-allyl-3a,8-dimethyltetrahydro-6,1-ethanoindole-2,5(*3H,4H*)-dione (S44)

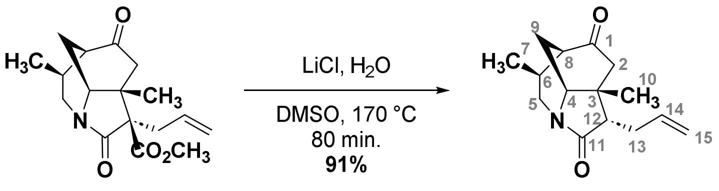

Compound **S44** was prepared adopting the procedure published by Dixon.[33] A stirred solution of compound **3** (120 mg, 0.393 mmol, 1 equiv.), LiCl (166 mg, 3.93 mmol, 10 equiv.) in a mixture of DMSO (5.4 mL) and water (21.2 µL, 1.18 mmol, 3.00 equiv.) was heated to 170 °C for 80 minutes. After cooling down, the reaction mixture was loaded onto silica and purified by column chromatography (ethyl acetate:pentane = 40:60). The compound **S44** was obtained as a white solid (88.0 mg, 91%) after lyophilization overnight.

MP: 92-94 °C (rac).

^{1}H NMR (400 MHz, $CDCl_3$) δ: 5.89 (dddd, J = 17.1, 10.1, 8.9, 5.2 Hz, 1H, H-14), 5.14 (dtd, J = 17.1, 1.9, 1.1 Hz, 1H, H-15a), 5.03 (dtd, J = 10.1, 1.8, 0.7 Hz, 1H, H-15b), 4.02 (dd, J = 13.6, 8.8 Hz, 1H, H-5a), 3.69 – 3.62 (m, 1H, H-4), 2.97 – 2.80 (m, 1H, H-6), 2.68 – 2.56 (m, 1H, H-13a), 2.47 (d, J = 14.7 Hz, 1H, H-2a), 2.31 (ddd, J = 8.6, 6.2, 1.0 Hz, 1H, H-12), 2.20 (ddd, J = 13.6, 9.6, 1.0 Hz, 1H, H-5b), 2.19 – 2.08 (m, 2H, H-13b, H-2b), 2.10 – 2.07 (m, 2H, H-9), 1.96 – 1.89 (m, 1H, H-8), 1.27 (s, 3H, H-10), 1.00 (d, J = 7.0 Hz, 3H, H-7).

^{13}C NMR (101 MHz, $CDCl_3$) δ: 213.1 (C-8), 174.4 (C-11), 136.9 (C-14), 116.4 (C-15), 60.4 (C-4), 50.7 (C-12), 47.5 (C-3), 46.2 (C-8), 44.6 (C-2), 40.8 (C-5), 36.7 (C-6), 30.1 (C-13), 24.6 (C-10), 19.8 (C-9), 18.0 (C-7).

IR (neat) $\tilde{\nu}_{max}$/cm^{-1}: 2959, 2924, 2872, 2698, 1673, 1421, 1288, 929.

HRMS (ES+): exact mass calculated for $[M+H]^+$ $(C_{15}H_{22}NO_2)^+$ requires 248.1645, found 248.1646.

***rac*-(*3S,3aR,6R,7aR,8S*)-3-allyl-5-(((*R*)-3-((*tert*-butyldimethylsilyl)oxy)cyclopent-1-en-1-yl)methoxy)-3a,8-dimethyl-3a,6,7,7a-tetrahydro-6,1-ethanoindol-2(*3H*)-one (S45a) and *rac*-(*3S,3aR,6R,7aR,8S*)-3-allyl-5-(((*S*)-3-((*tert*-butyldimethylsilyl)oxy)cyclopent-1-en-1-yl)methoxy)-3a,8-dimethyl-3a,6,7,7a-tetrahydro-6,1-ethanoindol-2(3H)-one (S45b)**

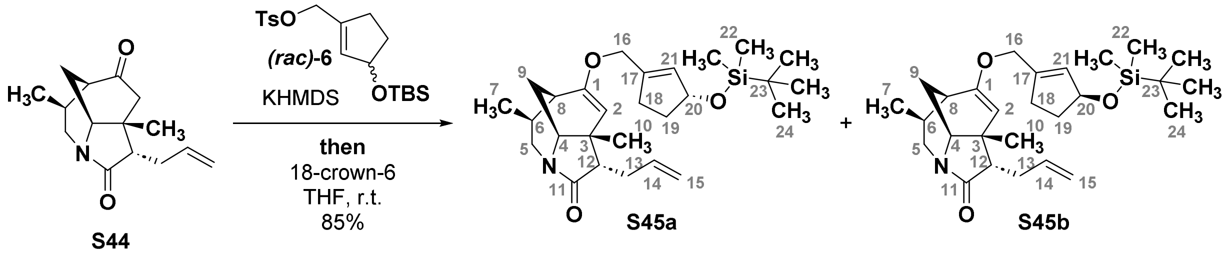

To a solution of ketone **S44** (60 mg, 243 µmol, 1 equiv.) in degassed THF (3.0 mL) was added KHMDS (0.5 M solution in toluene, 730 µL, 364 µmol, 1.50 equiv.) at 0 °C and the resulting solution was stirred for 20 min at room temperature. Then, a solution of 18-crown-6 (64.1 mg, 242.6 µmol, 1.00 equiv.) in degassed THF (1.5 mL) followed by tosylate **6** (14.7 wt % solution in benzene, 948 mg, 364 µmol, 1.50 equiv.) in THF (1.2 mL) were added. The solution was stirred for 1 h, and the formation of white precipitate and darkening of the reaction mixture was observed. The reaction mixture was then poured onto brine (20 mL) and extracted with ethyl acetate (4 x 15 mL). The combined organic phases were dried over Na_2SO_4, filtered and concentrated under reduced pressure. Purification by column chromatography (ethyl acetate:pentane = 20:80) afforded compound **S45** as an inseparable mixture of diastereomer, colorless oil (94.2 mg, 85%).

Note: Carbon peaks of compound **S45b** are labelled by "*". The assignment was possible because this isomer is not undergoing Claisen rearrangement (see next page), which allowed its isolation. The proton spectra of both compounds are not distinguishable.

^{1}H NMR (400 MHz, CDCl$_3$) δ: 6.01 (dddd, *J* = 17.1, 10.1, 8.7, 5.3 Hz, 1H, H-14), 5.61 (m, 1H, H-21), 5.11 (dtd, *J* = 17.1, 2.0, 1.1 Hz, 1H, H-15a), 5.01 (dtd, *J* = 10.0, 1.8, 0.8 Hz, 1H, H-15b), 4.91 (m, 1H, H-20), 4.28 (d, *J* = 12.7 Hz, 1H, H-16a), 4.16 – 4.08 (m, 2H, H-16b, H-2), 4.01 (dd, *J* = 13.6, 8.0 Hz, 1H, H-5a), 3.51 (d, *J* = 3.7 Hz, 1H, H-4), 2.56 – 2.47 (m, 1H, H-13a), 2.46 – 2.37 (m, 1H, H-18a), 2.37 – 2.24 (m, 3H, H-12, H-5b, H-19a), 2.23 – 1.99 (m, 3H, H-13b, H-6, H-18b), 1.98 – 1.86 (m, 2H, H-8, H-9a), 1.74 (ddt, *J* = 13.3, 7.5, 4.3 Hz, 1H, H-19b), 1.66 – 1.57 (m, 1H, H-9b), 1.17 (s, 3H, H-10), 0.94 (d, *J* = 6.9 Hz, 3H, H-7), 0.89 (s, 9H, H-24), 0.07 (s, 6H, H-22).

^{13}C NMR (101 MHz, CDCl$_3$) δ: 177.08 (C-11)*, 177.05 (C-11), 159.65 (C-1)*, 159.41 (C-1), 143.31 (C-17), 142.94 (C-17)*, 138.00 (2xC-14), 130.38 (C-21), 130.08 (C-21)*, 115.60 (C-15)*, 115.54 (C-15), 93.94 (C-2), 93.80 (C-2)*, 78.02 (C-20)*, 78.00 (C-20), 65.89 (C-16)*, 65.85 (C-16), 58.56 (2xC-4), 52.24 (2xC-

12), 47.20 (2xC-3), 41.19 (2xC-5), 37.74 (2xC-8), 34.17 (C-19)*, 34.15 (C-19), 32.78 (C-6)*, 32.73 (C-6), 31.25 (C-18)*, 31.16 (C-18), 30.12 (C-13)*, 30.08 (C-13), 26.12 (6xC-24), 22.42 (C-10)*, 22.39 (C-10), 19.90 (2xC-9), 19.53 (2xC-7), 18.43 (3xC-23), -4.43 (2xC-22), -4.45 (2xC-22).

IR (thin film) $\tilde{\nu}_{max}$/cm^{-1}: 2952, 2928, 2856, 1697, 1650, 1411, 1159, 1063, 835.

HRMS (ES+): exact mass calculated for $[M+Na]^+$ $(C_{27}H_{43}NO_3SiNa)^+$ requires 480.2904, found 480.2899.

***rac*-(3S,3aR,4S,6R,7aR,8S)-3-allyl-4-((1R,2R)-2-((tert-butyldimethylsilyl)oxy)-5-methylenecyclopentyl)-3a,8-dimethyltetrahydro-6,1-ethanoindole-2,5(3H,4H)-dione (23)**

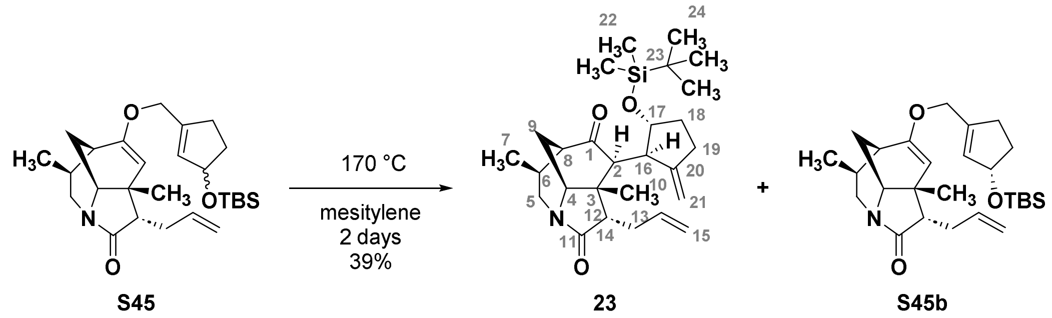

Compound **23** was prepared adopting the procedure published by Carreira.[31] A solution of compound **S45** (1:1 mixture of diastereomers, 90.0 mg, 197 µmol, 1 equiv.) in dry degassed mesitylene (5.5 mL) was heated for 2 days in passivated pressure tube at 170 °C. Purification by gradient column chromatography (ethyl acetate:pentane = 0:100 to 30:70) afforded compound **23** as a crystalline solid (34.7 mg, 39%) and unreactive diastereomer **S45b** as oil (35.5 mg, 37%).

MP: 158 – 162 °C (*rac*).

^{1}H NMR (500 MHz, CDCl$_3$) δ: 5.86 (dtd, *J* = 17.1, 9.8, 4.5 Hz, 1H, H-14), 5.15 (dtd, *J* = 17.1, 2.1, 0.9 Hz, 1H, H-15a), 5.05 (dt, *J* = 10.1, 1.9 Hz, 1H, H-15b), 4.89 (s, 1H, H-21a), 4.86 – 4.80 (m, 1H, H-21b), 4.04 – 3.99 (m, 1H, H-17), 3.98 (dd, *J* = 13.6, 8.9 Hz, 1H, H-5a), 3.60 (d, *J* = 5.7 Hz, 1H, H-), 2.86 (td, *J* = 9.2, 6.9 Hz, 1H, H-6), 2.69 (dddd, *J* = 15.2, 7.6, 4.4, 2.1 Hz, 1H, H-13a), 2.61 (d, *J* = 8.5 Hz, 1H, H-16), 2.40 (dd, *J* = 9.5, 5.5 Hz, 1H, H-12), 2.35 – 2.23 (m, 4H, H-9a, H-21a, H-21b, H-2), 2.22 – 2.13 (m, 2H, H-13b, H-5b), 2.14 – 2.05 (m, 1H, H-18a), 2.07 – 1.99 (m, 1H, H-9b), 1.98 (d, *J* = 4.6 Hz, 1H, H-8), 1.64 – 1.55 (m, 1H, H-18b), 1.40 (s, 3H, H-10), 0.97 (d, *J* = 7.0 Hz, 3H, H-7), 0.84 (s, 9H, H-24), 0.02 (s, 6H, H-22).

^{13}C NMR (126 MHz, CDCl$_3$) δ: 213.2 (C-1), 173.8 (C-11), 152.7 (C-20), 137.1 (C-14), 116.3 (C-15), 109.7 (C-21), 78.9 (C-17), 61.7 (C-4), 54.3 (C-16), 53.4 (C-12), 53.1 (C-2), 48.8 (C-3), 45.8 (C-8), 40.7 (C-5), 37.0 (C-6), 32.0 (C-18), 29.9 (C-13), 28.6 (C-19), 25.8 (C-24), 21.9 (C-10), 18.6 (C-9), 18.0 (C-7, C-29), -4.3 (C-22), -4.4 (C-22).

IR (thin film) $\tilde{\nu}_{max}$/cm^{-1}: 2955, 2928, 2892, 1693, 1680, 1427, 1252, 1092, 999, 891, 837, 771.

HRMS (ES+): exact mass calculated for $[M+H]^+$ $(C_{27}H_{44}NO_3Si)^+$ requires 458.3085, found 458.3083.

3.5 Synthesis of building block, catalysts, and reagents

tris(4-(trifluoromethyl)phenyl)phosphane (13d)

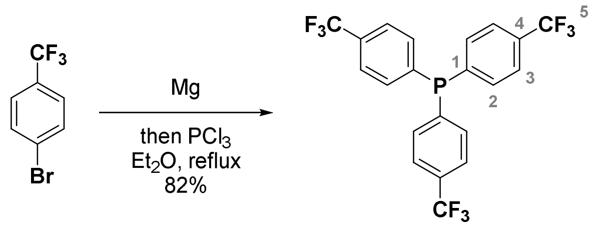

13d

Prepared according to a literature procedure.[38] To a suspension of Mg (2.16 g, 88.9 mmol, 3.00 equiv.) in Et_2O (16 mL) was dropwise added solution of bromide 1-bromo-4-(trifluoromethyl)benzene (20 g, 88.9 mmol, 3.00 equiv.) in Et_2O (32 mL) over 1 h and the resulting mixture was stirred for additional 1 h. Then, a solution of PCl_3 (2.6 mL) in Et_2O (60 ml) was added dropwise over 45 min, and the reaction mixture was stirred for 2 h. The reaction was quenched with water (100 mL) and extracted with Et_2O (3 x 100 mL). The combined organic phases were dried over Na_2SO_4, filtered and concentrated under reduced pressure. Purification by column chromatography (pentane) afforded phosphine **13d** as a white solid (11.35 g, 82%). The spectroscopic data are in agreement with the literature values.[38]

^{1}H NMR (400 MHz, $CDCl_3$) δ: 7.70 – 7.60 (m, 6H, H-2), 7.46 – 7.37 (m, 6H, H-3).

^{13}C NMR (101 MHz, $CDCl_3$) δ: 140.4 (d, *J* = 14.2 Hz, C-1), 134.1 (d, *J* = 20.1 Hz, C-2), 131.7 (q, *J* = 32.7 Hz, C-4), 125.8 (dq, *J* = 7.5, 3.7 Hz, C-3), 124.0 (q, *J* = 272.6 Hz, C-6).

^{19}F NMR (376 MHz, $CDCl_3$) δ: -62.96.

^{31}P NMR (162 MHz, $CDCl_3$): δ -6.05.

3.5.1 Preparation of CBS catalyst

(*S*)-diphenyl(pyrrolidin-2-yl)methanol (S48)

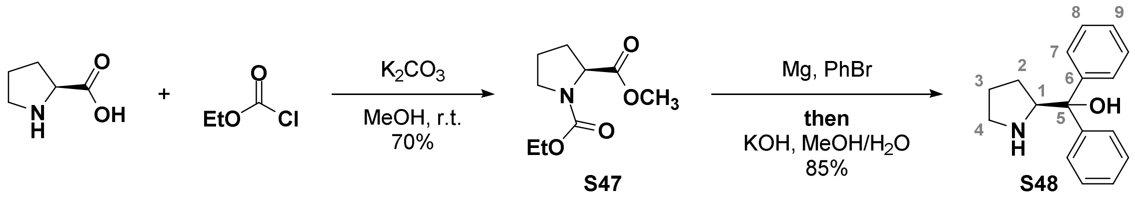

Prepared according to a literature procedure.[39] To a stirred suspension of L-proline (6.00 g, 52.1 mmol, 1 equiv.) and K_2CO_3 (9.36 g, 67.8 mmol, 1.30 equiv.) in dry MeOH (90 mL) at 0 °C was dropwise added ethyl chloroformate (11.0 mL, 115 mmol, 2.20 equiv.) and the resulting mixture was stirred at room temperature for 16 h. The reaction mixture was then concentrated under reduced pressure, and the residues dissolved in water (50 mL) and extracted with $CHCl_3$ (4 x 35 mL). The combined organic phases were dried over anhydrous Na_2SO_4, filtered and concentrated under reduced pressure. The product **S47** was obtained as light-yellow oil (7.36 g, 70%) and was used in the next step without further purification.

An oven-dried two-necked flask equipped with a reflux condenser was charged with Mg turnings (6.62 g, 272 mmol, 8.00 equiv.), a catalytic amount of I_2 and THF (103 mL). A solution of bromobenzene (17.9 mL, 170 mmol, 5.00 equiv.) in THF (51 mL) was added dropwise, resulting in an exothermic reaction that was compensated with a room-temperature water bath. After stirring for 1 h at room temperature, a solution of ester **S47** (6.85 g, 34.0 mmol, 1 equiv.) in THF (34 mL) was added at 0 °C, and the resulting solution was stirred at room temperature for 4 h. The reaction mixture was then cautiously quenched with sat. aq. solution of NH_4Cl (60 mL) and extracted with Et_2O (4 x 90 mL). The combined organic layers were washed with brine (90 mL), dried over anhydrous Na_2SO_4, filtered and concentrated under reduced pressure.

To a stirred solution of crude product obtained in the previous step in MeOH (11.0 mL) and toluene (38.4 mL) was added KOH (19.1 g, 340 mmol, 10.0 equiv.), and the resulting mixture was heated to 60 °C. After 1 h, water (65.8 mL) was added, and the resulting mixture was stirred under reflux for another 1 h. After cooling to room temperature, the phases were separated, and the aqueous phase was washed with toluene (3 x 50 mL). The combined organic phases were washed with brine (90 mL), dried over anhydrous Na_2SO_4, filtered and concentrated under reduced pressure. Purification by gradient column chromatography (ethyl acetate:pentane = 25:75 to 100:0) afforded alcohol **S48** as a colorless solid (7.36 g, 85%). The spectroscopic data are in agreement with the literature values.[39]

^{1}H NMR (400 MHz, $CDCl_3$) δ: 7.62 – 7.55 (m, 2H, H-7), 7.55 – 7.48 (m, 2H, H-7), 7.35 – 7.24 (m, 4H, H-8), 7.22 – 7.13 (m, 2H, H-9), 4.27 (t, J = 7.6 Hz, 1H, H-1), 3.05 (ddd, J = 9.3, 6.6, 5.1 Hz, 1H, H-4a), 2.95 (dt, J = 9.3, 7.4 Hz, 1H, H-4b), 1.82 – 1.67 (m, 2H, H-3), 1.67 – 1.51 (m, 2H, H-2).

^{13}C NMR (101 MHz, $CDCl_3$) δ: 148.3 (C-9), 145.6 (C-9), 128.4 (C-8), 128.1 (C-8), 126.6 (C-9), 126.5 (C-9), 126.0 (C-7), 125.7 (C-7), 77.2 (C-5), 64.6 (C-1), 46.9 (C-4), 26.4 (C-2), 25.6 (C-3).

$[\boldsymbol{\alpha}]_{\boldsymbol{D}}^{\mathbf{25}}$= -53.0 (c = 1.0, MeOH), literature value $[\boldsymbol{\alpha}]_{\boldsymbol{D}}^{\mathbf{25}}$= -56.1 (c = 2.5, MeOH).[39]

3.5.2 Preparation of building blocks for *O*-allylation

allyl 4-methylbenzenesulfonate (S49)

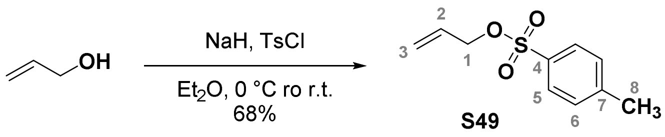

Compound **S49** was prepared according to the literature.[40] To a suspension of NaH (60% in mineral oil, 1.26 g, 31.5 mmol, 1.5 equiv.) washed with pentane in Et_2O (32 mL) was added allyl alcohol (1.57 mL, 23.1 mmol, 1.10 equiv.). After gas evolution ceased, the reaction mixture was cooled to 0 °C, and a solution of TsCl (4.00 g, 21.0 mmol, 1 equiv.) in Et_2O (50 mL) was added dropwise. After stirring for 2 h at room temperature, the reaction mixture was quenched with sat. aq. solution of NH_4Cl (50 mL) and extracted with Et_2O (3 x 25 mL). The combined organic phases were dried over anhydrous Na_2SO_4, filtered and concentrated under reduced pressure. The crude product was washed with pentane (2 x 10 mL), and the volatiles were removed *in a vacuo*. Product **S49** was obtained as a colorless oil (3.037 g, 68%). The spectroscopic data are in agreement with the literature values.[40]

^{1}H NMR (400 MHz, $CDCl_3$) δ: 7.83 – 7.76 (m, 2H, H-5), 7.38 – 7.30 (m, 2H, H-6), 5.82 (ddt, *J* = 17.1, 10.3, 5.9 Hz, 1H, H-2), 5.32 (dq, *J* = 17.1, 1.4 Hz, 1H,H-3a), 5.25 (dq, *J* = 10.3, 1.1 Hz, 1H, H-3b), 4.53 (dt, *J* = 5.9, 1.3 Hz, 2H, H-1), 2.45 (s, 3H, H-8).

^{13}C NMR (101 MHz, $CDCl_3$) δ: 144.9 (C-4), 133.4 (C-7), 130.4 (C-2), 130.0 (C-6), 128.1 (C-5), 120.4 (C-3), 70.9 (C-1), 21.8 (C-8).

3.5.3 Synthesis of E-ring fragment of himalensine A

Scheme S15. Synthesis of E-ring building blocks.

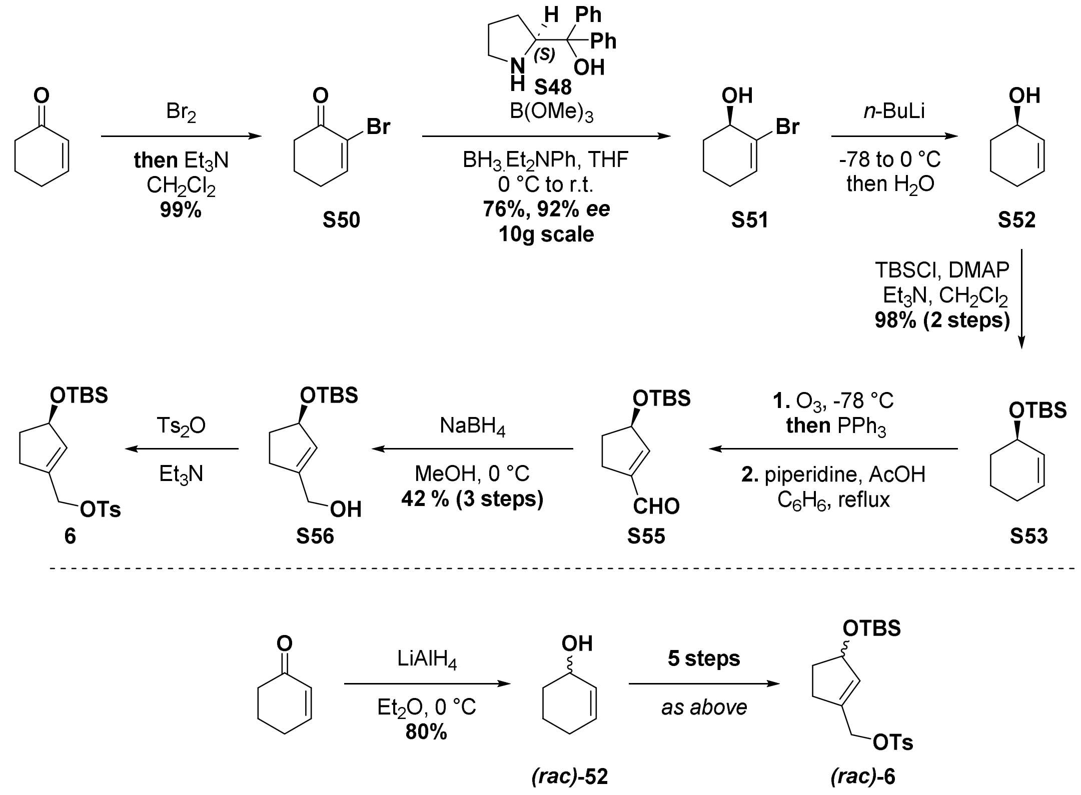

***rac*-cyclohex-2-en-1-ol (S52)**

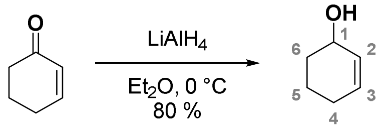

S52

To a suspension of $LiAlH_4$ (987 mg, 26.0 mmol, 0.50 equiv.) in dry Et_2O (25 mL) was dropwise added solution of cyclohexenone (5.00 g, 52.0 mmol, 1 equiv.) in dry Et_2O (13 mL) at 0 °C. After 30 min, the ice bath was removed, and the reaction mixture was stirred at room temperature for 5 h. The reaction was then carefully quenched with distilled water (20 mL), diluted with Et_2O (120 mL) and washed with water (3x30 mL). The organic phase was dried over Na_2SO_4, filtered and concentrated under reduced pressure (300 mbar, 40 °C). The crude product **S52** was obtained as yellowish oil and used in the next step without further purification (4.82 g, 80%). The spectroscopic data are in agreement with the literature values.[41]

^{1}H NMR (400 MHz, CDCl$_3$) δ: 5.88 – 5.79 (m, 1H, H-3), 5.79 – 5.70 (m, 1H, H-2), 4.19 (s, 1H, H-1), 2.11 – 1.95 (m, 2H, H-4), 1.94 – 1.81 (m, 1H, H-6a), 1.80 – 1.67 (m, 1H, H-5a), 1.66 – 1.53 (m, 2H, H-6b, H-5b).

^{13}C NMR (101 MHz, CDCl$_3$) δ: 130.7 (C-3), 130.0 (C-2), 65.7 (C-1), 32.2 (C-6), 25.2 (C-4), 19.1 (C-5).

2-bromocyclohex-2-en-1-one (S50)

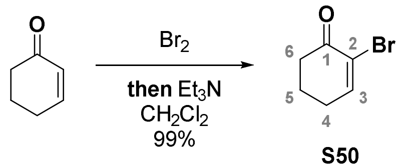

S50

Prepared according to a literature procedure.[42] To a solution of cyclohexenone (3.00 g, 31.2 mmol, 1 equiv.) in CH_2Cl_2 (80 mL) was dropwise added solution of bromine (1.63 mL, 31.8 mmol, 1.02 equiv.) in CH_2Cl_2 (80 mL) at 0 °C. The immediate consumption of bromine was observed. After stirring for 1.5 h, Et_3N (7.26 mL, 52.1 mmol, 1.67 equiv.) was added dropwise at 0 °C, and the resulting mixture was stirred for another 1.5 h at room temperature. The reaction mixture was then washed with 1 M HCl (50 mL) and brine (50 mL). The organic phase was dried over anhydrous Na_2SO_4, filtered and concentrated under reduced pressure. The product **S50** was obtained as a brownish solid (5.43 g, 99%). The spectroscopic data are in agreement with the literature values.[43]

^{1}H NMR (400 MHz, CDCl$_3$) δ: 7.42 (t, *J* = 4.5 Hz, 1H, H-3), 2.66 – 2.58 (m, 2H, H-6), 2.44 (td, *J* = 6.0, 4.4 Hz, 2H, H-4), 2.12 – 1.97 (m, 2H, H-5).

^{13}C NMR (101 MHz, CDCl$_3$) δ: 191.4 (C-1), 151.3 (C-3), 124.0 (C-2), 38.5 (C-6), 28.5 (C-4), 22.8 (C-5).

***(R)*-2-bromocyclohex-2-en-1-ol (S51)**

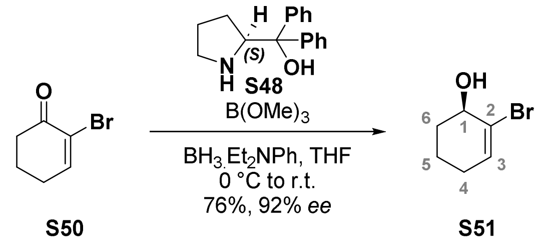

Prepared according to a literature procedure.[44] To a solution of *(S)-α,α*-diphenylprolinol (1.45 g, 5.71 mmol, 0.10 equiv.) in dry THF (170 mL) was added $B(OMe)_3$ (764 µL, 6.86 mmol, 0.12 equiv.) and the resulting solution was stirred at room temperature for 1 h. Then, BH_3.*N,N*-diethylaniline (10.2 mL, 57.1 mmol, 1.00 equiv.) was added, and the resulting solution was cooled to – 10°C before a solution of enone **S50** (10.0 g, 57.1 mmol, 1 equiv.) in dry THF (20 mL) was added dropwise over 45 min using a syringe pump. The reaction was stirred for 2 days at 0 °C and the progress of the reaction was monitored by ^1H NMR. After the complete conversion of the enone, the reaction was quenched with 1 M HCl (100 mL), phases were separated, and the organic phase was extracted with Et_2O (3 x 150 mL). The combined organic phases were dried over anhydrous Na_2SO_4, filtered and concentrated under reduced pressure. Purification by gradient column chromatography (Et_2O:pentane = 15:85 to 20:80) afforded allylic alcohol **S51** as a colorless oil (7.71 g, 76%, 92.3% *ee*). The spectroscopic data are in agreement with the literature values.[44]

^{1}H NMR (400 MHz, $CDCl_3$) δ: 6.20 (t, *J* = 4.1 Hz, 1H, H-3), 4.21 (dtd, *J* = 6.4, 4.8, 1.6 Hz, 1H, H-1), 2.18 (d, *J* = 4.2 Hz, 1H, OH), 2.17 – 1.99 (m, 2H, H-4), 1.99 – 1.84 (m, 2H, H-6), 1.74 (m, 1H, H-5a), 1.68 – 1.58 (m, 1H, H-5b).

^{13}C NMR (101 MHz, $CDCl_3$) δ: 132.7 (C-3), 126.0 (C-2), 70.0 (C-1), 32.1 (C-6), 27.9 (C-4), 17.8 (C-5).

HPLC: (Chiralpak AD-H, hexane/isopropanol 95:5, 1.0 mL min^{-1}, λ=220 nm) t_R = 10.4 min (major), 9.4 min (minor).

$[\alpha]_D^{25}$= 81.3 (c = 1.0, $CHCl_3$, >92.3% ee), literature value for enantiomer $[\alpha]_D^{25}$= -85.2 (c = 1.89, $CHCl_3$, 99% ee).[44]

(R)-tert-butyl(cyclohexe-2-en-1-yloxy)dimethylsilane (S53)

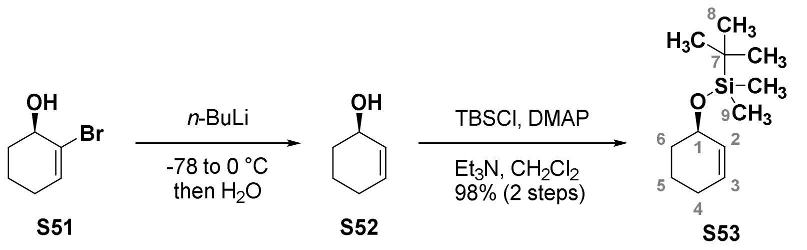

Prepared according to a literature procedure.[42] To a stirred solution of bromide **S51** (7.70 g, 43.5 mmol, 1 equiv.) in dry Et_2O (220 mL) at -78 °C was dropwise added *n*-BuLi (2.5 M in hexanes, 60.9 mL, 3.50 equiv.). The resulting mixture was stirred at this temperature for 1 h and then 3 h at 0°C. The reaction was then quenched with distilled water (100 mL), phases were separated, and the aqueous phase was extracted with Et_2O (3 x 100 mL). The combined organic phases were dried over Na_2SO_4, filtered and concentrated under reduced pressure. The crude product **S52** was obtained as a colorless oil and used in the next step without further purification.

To a stirred solution of *(R)*-cyclohex-2-en-1-ol (43.5 mmol, 1 equiv.) in dry CH_2Cl_2 (80 mL) at room temperature was added Et_3N (9.10 mL, 65.3 mmol, 1.50 equiv.), TBSCl (8.52 g, 56.6 mmol, 1.30 equiv.) and DMAP (532 mg, 4.35 mmol, 0.10 equiv.). After stirring for 17 h, the reaction mixture was diluted with CH_2Cl_2 and washed with 1 M HCl (100 mL), sat. aq. solution of $NaHCO_3$ (100 mL) and brine (100 mL). The organic phase was dried over Na_2SO_4, filtered and concentrated under reduced pressure. Purification by column chromatography (Et_2O:pentane = 0:100 to 5:95) afforded protected alcohol **S53** as a colorless oil (9.04 g, 98%). The spectroscopic data are in agreement with the literature values.[45]

^{1}H NMR (400 MHz, $CDCl_3$) δ: 5.79 – 5.70 (m, 1H, H-3), 5.67 – 5.58 (m, 1H, H-2), 4.23 (m, 1H, H-1), 2.11 – 1.97 (m, 1H, H-4a), 1.97 – 1.87 (m, 1H, H-4b), 1.87 – 1.70 (m, 2H, H-6a, H-5a), 1.63 – 1.46 (m, 2H, H-6b, H-5b), 0.90 (s, 9H), 0.08 (s, 3H, H-9), 0.07 (s, 3H, H-9).

^{13}C NMR (101 MHz, $CDCl_3$) δ: 131.4 (C-2), 129.2 (C-3), 66.9 (C-1), 32.7 (C-6), 26.1 (C-8), 25.1 (C-4), 19.9 (C-5), 18.4 (C-7), -4.37 (C-9), -4.43 (C-9).

***(R)*-(3-((*tert*-butyldimethylsilyl)oxy)cyclopent-1-en-1-yl)methanol (S56)**

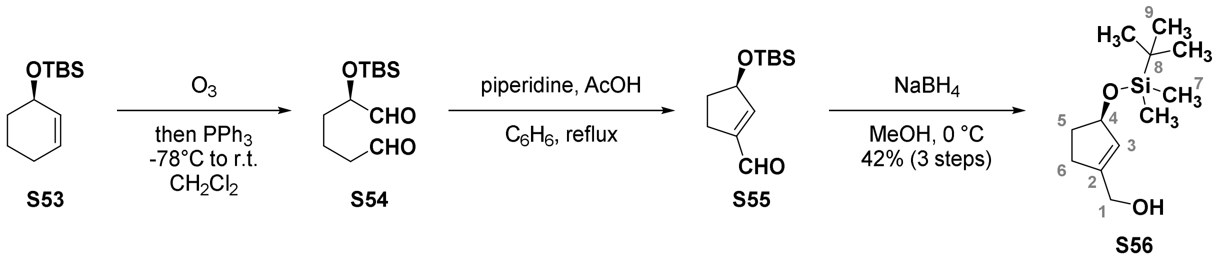

Prepared according to a literature procedure.[42] A stirred solution of alkene **S53** (9.04 g, 42.6 mmol, 1 equiv.) in CH_2Cl_2 (100 mL) at -78 °C was bubbled with ozone until a persistent blue color was observed. After removing the excess of ozone by bubbling the reaction mixture with oxygen for 10 min, the reaction mixture was quenched with a solution of PPh_3 (11.2 g, 42.6 mmol, 1.00 equiv.) in CH_2Cl_2, allowed to warm to room temperature overnight and then concentrated under reduced pressure (30 °C). The obtained residue was dissolved in Et_2O (100 mL), and the formed white precipitate was filtered off. If the formation of more precipitate was observed during the concentration of the filtered solution, the filtration was repeated. The crude dialdehyde **S54** was obtained as a yellowish oil and used in the next step without further purification.

To a solution of dialdehyde **S54** in benzene (50 mL) at room temperature were added acetic acid (97.4 µL, 1.70 mmol, 4.00 mol %) and piperidine (160 µL, 1.62 mmol, 3.80 mol %) and the resulting mixture was placed into pre-heated oil bath (90 °C) and stirred for 1 h. After cooling to room temperature, the reaction mixture was diluted with ethyl acetate (100 mL) and washed with 1 M HCl (2 x 40 mL), sat. aq. solution of $NaHCO_3$ (40 mL) and brine (40 mL). The organic phase was dried over Na_2SO_4, filtered and concentrated under reduced pressure. The enal **S55** was obtained as a yellowish oil and used in the next step without further purification.

To a stirred solution of enal **S55** in MeOH (125 mL) at 0 °C was added $NaBH_4$ (1.61 g, 42.6 mmol, 1.00 equiv.) over a period of 20 min. After stirring for 30 min, the reaction mixture was quenched with water (60 mL) and extracted with CH_2Cl_2 (4 x 80 mL). The combined organic phases were dried over anhydrous Na_2SO_4, filtered and concentrated under reduced pressure. Purification by gradient column chromatography (ethyl acetate:pentane = 1:9 to 2:8) afforded alcohol **S56** as a yellowish oil (4.10 g, 42% over 3 steps). The spectroscopic data are in agreement with the literature values.[42]

^{1}H NMR (400 MHz, $CDCl_3$) δ: 5.61 (h, *J* = 1.8 Hz, 1H, H-3), 4.91 (m, 1H, H-4), 4.21 (d, *J* = 14.5 Hz, 1H, H-1), 4.15 (d, *J* = 14.6 Hz, 1H, H-1), 2.50 – 2.38 (m, 1H, H-6), 2.29 (dddd, *J* = 16.2, 8.7, 7.2, 3.3 Hz, 1H, H-5), 2.24 – 2.13 (m, 1H, H-6), 1.80 – 1.64 (m, 2H, H-5), 0.89 (s, 9H, H-9), 0.07 (s, 6H, H-7).

^{13}C NMR (101 MHz, $CDCl_3$) δ: 147.4 (C-2), 127.9 (C-3), 78.0 (C-4), 62.2 (C-1), 34.3 (C-5), 30.9 (C-6), 26.1 (C-9), 18.5 (C-8), -4.45 (C-7), -4.48 (C-7).

$[\boldsymbol{\alpha}]_{\boldsymbol{D}}^{\mathbf{25}}$= +57.7° (c = 1.00, $CHCl_3$, 92% *ee*), literature value for enantiomer $[\boldsymbol{\alpha}]_{\boldsymbol{D}}^{\mathbf{25}}$= −50° (c = 1.00, $CHCl_3$, >95% *ee*).[42]

(R)-(3-((*tert*-butyldimethylsilyl)oxy)cyclopent-1-en-1-yl)methyl 4-methylbenzenesulfonate (6)

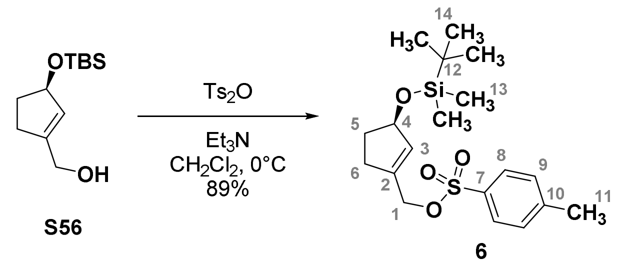

To a solution of alcohol **S56** (400 mg, 1.75 mmol, 1 equiv.) in dry CH_2Cl_2 (6.0 mL) at 0 °C was added Et_3N (366 µL, 2.63 mmol, 1.50 equiv.) followed by a solution of Ts_2O (572 mg, 1.75 mmol, 1.00 equiv.) in CH_2Cl_2 (4.0 mL). After stirring for 30 min, the reaction mixture was quenched with MeOH (0.5 mL) and stirred for an additional 20 min. The reaction mixture was poured onto sat. aq. solution of $NaHCO_3$ (15 mL), extracted with CH_2Cl_2 (3 x 15 mL), dried over anhydrous Na_2SO_4, filtered and concentrated under reduced pressure at 30 °C. The crude tosylated allylic alcohol was purified by very fast gradient column chromatography (Et_2O:pentane = 0:100 to 10:90). The fractions containing the product were concentrated under reduced pressure at 30 °C and then transferred into a vial using Et_2O. Et_2O was blown down by a stream of nitrogen which afforded tosyl alcohol **6** as a colorless oil (596 mg, 89%). Because of its relatively low stability, the pure product, while still under the atmosphere of nitrogen, was diluted with benzene (1.78 g) and stored in the freezer.

NOTE: The rapid decomposition of the purified material after column chromatography was observed several times, mostly after the evaporation of solvent and exposure of the residue to air. Allyl tosylate **6** should be used immediately after its preparation. Short-term storage (for a few days) as a frozen solution in benzene is possible, but even this solution is undergoing slow decomposition/polymerization, indicated by the color change of the solution and the formation of black particles.

^{1}H NMR (400 MHz, C_6D_6) δ: 7.76 – 7.69 (m, 2H, H-8), 6.74 – 6.66 (m, 2H, H-9), 5.51 (m, 1H, H-3), 4.60 (m, 1H, H-4), 4.45 – 4.29 (m, 2H, H-1), 2.11 – 1.99 (m, 1H, H-6), 1.96 – 1.77 (m, 2H, H-6, H-5), 1.84 (s, 3H, H-11), 1.62 – 1.53 (m, 1H, H-5), 0.93 (s, 9H, H-14), 0.01 (s, 6H, H-13).

^{13}C NMR (101 MHz, C_6D_6) δ: 144.8 (C-7), 140.3 (C-10), 135.1 (C-2), 133.7 (C-3), 130.4 (C-9), 128.8 (C-8), 78.4 (C-4), 69.0 (C-1), 34.9 (C-5), 31.4 (C-6), 26.7 (C-14), 21.7 (C-11), 18.9 (C-12), -3.91 (C-13), -3.96 (C-13).

^{1}H NMR (400 MHz, $CDCl_3$) δ: 7.82 – 7.75 (m, 2H, H-8), 7.37 – 7.30 (m, 2H, H-9), 5.65 (hept, *J* = 1.4 Hz, 1H, H-3), 4.82 (dddt, *J* = 6.9, 5.5, 3.2, 1.4 Hz, 1H, H-4), 4.65 – 4.52 (m, 2H, H-1), 2.45 (s, 3H, H-11), 2.39

– 2.27 (m, 1H, H-6), 2.27 – 2.08 (m, 2H, H-5, H-6), 1.72 – 1.58 (m, 1H, H-5), 0.87 (s, 9H, H-14), 0.05 (s, 6H, H-13).

3.5.4 Preparation of building block for amide coupling

2-(methoxycarbonyl)pent-4-enoic acid (7)

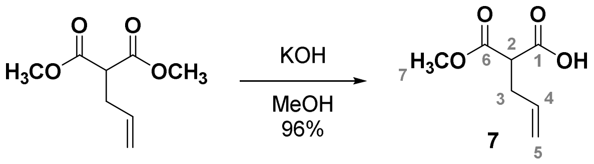

Prepared according to a literature procedure.[33] To a solution of dimethyl allylmalonate (10.0 g, 58.1 mmol, 1 equiv.) in MeOH (116 mL) was added KOH (3.26 g, 58.1 mmol, 1.00 equiv.), and after stirring for 26 h, the reaction mixture was diluted with water (120 mL) and extracted with Et_2O (3 x 100 mL). The aqueous phase was then acidified with 6 M HCl (40 mL) and extracted with CH_2Cl_2 (5 x 80 mL). The combined CH_2Cl_2 phases were dried over anhydrous Na_2SO_4, filtered and concentrated under reduced pressure (50 mbar, 45 °C). The product of hydrolysis **7** was obtained as a yellowish oil (8.81 g, 96%). The spectroscopic data are in agreement with the literature values.[46]

^{1}H NMR (400 MHz, $CDCl_3$) δ: 10.74 (s, 1H, OH), 5.77 (ddt, *J* = 17.1, 10.2, 6.8 Hz, 1H, H-4), 5.13 (dq, *J* = 17.1, 1.5 Hz, 1H, H-5a), 5.08 (dq, *J* = 10.2, 1.3 Hz, 1H, H-5b), 3.75 (s, 3H, H-7), 3.49 (t, *J* = 7.5 Hz, 1H, H-2), 2.66 (dddd, *J* = 8.9, 7.4, 2.5, 1.3 Hz, 2H, H-3).

^{13}C NMR (101 MHz, $CDCl_3$) δ: 174.6 (C-1), 169.2 (C-6), 133.6 (C-4), 118.1 (C-5), 52.8 (C-7), 51.4 (C-2), 32.9 (C-3).

3.5.5 Preparation of ruthenium scavenger

methyl formylglycinate (S58)

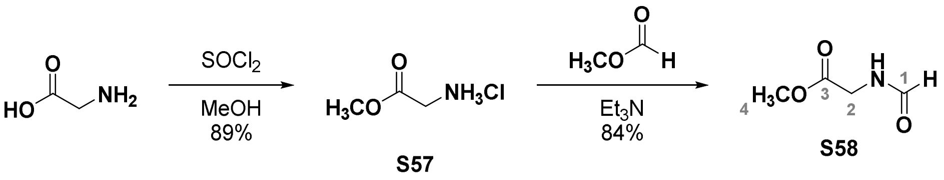

$SOCl_2$ (11.2 mL, 154 mmol, 1.1 equiv.) was dropwise added to MeOH (40 mL) at 0 °C, and after stirring for 30 min at this temperature, glycine (10.5 g, 140 mmol, 1 equiv.) was added portionwise, and the resulting mixture was stirred at 40 °C overnight. After cooling, the formed white precipitate was filtered off and dried under a high vacuum (15.6 g, 89%).

Prepared according to a literature procedure.[47] A solution of the above-obtained glycine salt **S57** (15.5 g, 123 mmol, 1 equiv.) and Et_3N (18.9 mL, 136 mmol, 1.1 equiv.) in methyl formate (93 mL) was stirred for 4 days at room temperature. The formed solids were filtered off, and the reaction mixture concentrated to half of its original volume. After cooling to 0 °C, the reaction mixture was filtered again, and the residual solvent was removed under reduced pressure. Purification by vacuum distillation (≈10 mbar, 150 °C) afforded product **S58** as a colorless oil that partially crystallized at room temperature (12.1 g, 84%). The spectroscopic data are in agreement with the literature values.[47]

^{1}H NMR (400 MHz, $CDCl_3$) δ: 8.23 (s,1H, H-1), 6.49 (br s, 1H, NH), 4.07 (dd, *J* = 5.4, 1.3 Hz, 2H, H-2), 3.78 – 3.72 (m, 3H, H-4).

^{13}C NMR (101 MHz, $CDCl_3$) δ: 170.1 (C-3/1), 161.4 (C-1/3), 52.6 (C-4), 39.9 (C-2).

methyl 2-isocyanoacetate (S59)

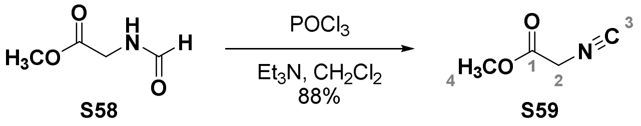

Prepared according to a literature procedure.[48] To a solution of compound **S58** (12.0 g, 102 mmol, 1 equiv.) and Et_3N (35.7 mL, 256 mmol, 2.50 equiv.) in CH_2Cl_2 (240 mL) was dropwise added $POCl_3$ (9.55 mL, 102 mmol, 1.00 equiv.) at 0 °C and the resulting solution was stirred at room temperature for 90 min. The reaction mixture was then carefully quenched with a solution of Na_2CO_3 (12.0 g in 200 mL of water), and after stirring for 30 min, the phases were separated, and the aqueous phase was extracted with CH_2Cl_2 (100 mL). The combined organic phases were dried over anhydrous Na_2SO_4, filtered and concentrated under reduced pressure. The isonitrile **S59** was obtained as a brown oil containing traces of Et_2O (11.0 g, 81 wt%, 88% yield) and was used in the next step without further purification. The spectroscopic data are in agreement with the literature values.[48]

^{1}H NMR (400 MHz, $CDCl_3$) δ: 4.24 (s, 2H, H-2), 3.83 (s, 3H, H-4).

^{13}C NMR (101 MHz, $CDCl_3$) δ: 164.5 (C-1), 161.6 (C-2), 53.5 (C-4), 43.5 (C-3).

potassium 2-isocyanoacetate (S60)

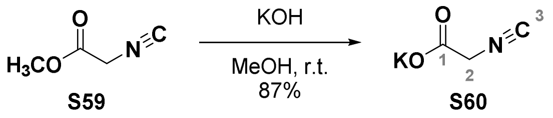

Prepared according to a literature procedure.[49] To a solution of ester **S59** (10.8 g, 81 wt%, 88.8 mmol, 1 equiv.) in MeOH (50 mL) was added KOH (4.98 g, 88.8 mmol, 1.00 equiv.) at 0 °C and after stirring for 30 min at 0 °C, the ice bath was removed, and the reaction mixture was stirred at room temperature for another 3 h. The reaction mixture was then concentrated under reduced pressure, the solid residue was suspended in cold Et_2O, filtered off, washed several times with cold Et_2O and dried *in vacuo*. Potassium isocyanoacetate was obtained as a white solid (9.57 g, 87%). The spectroscopic data are in agreement with the literature values.[50]

^{1}H NMR (400 MHz, DMSO) δ: 3.74 (t, *J* = 1.7 Hz, 2H, H-2).

^{13}C NMR (101 MHz, DMSO) δ: 164.9 (C-1), 154.5 (t, *J* = 6.2 Hz, C-3), 46.2 (t, *J* = 6.3 Hz, C-2).

4 NMR Spectra

^{1}H NMR (400 MHz, $CDCl_3$) of compound **S1.**

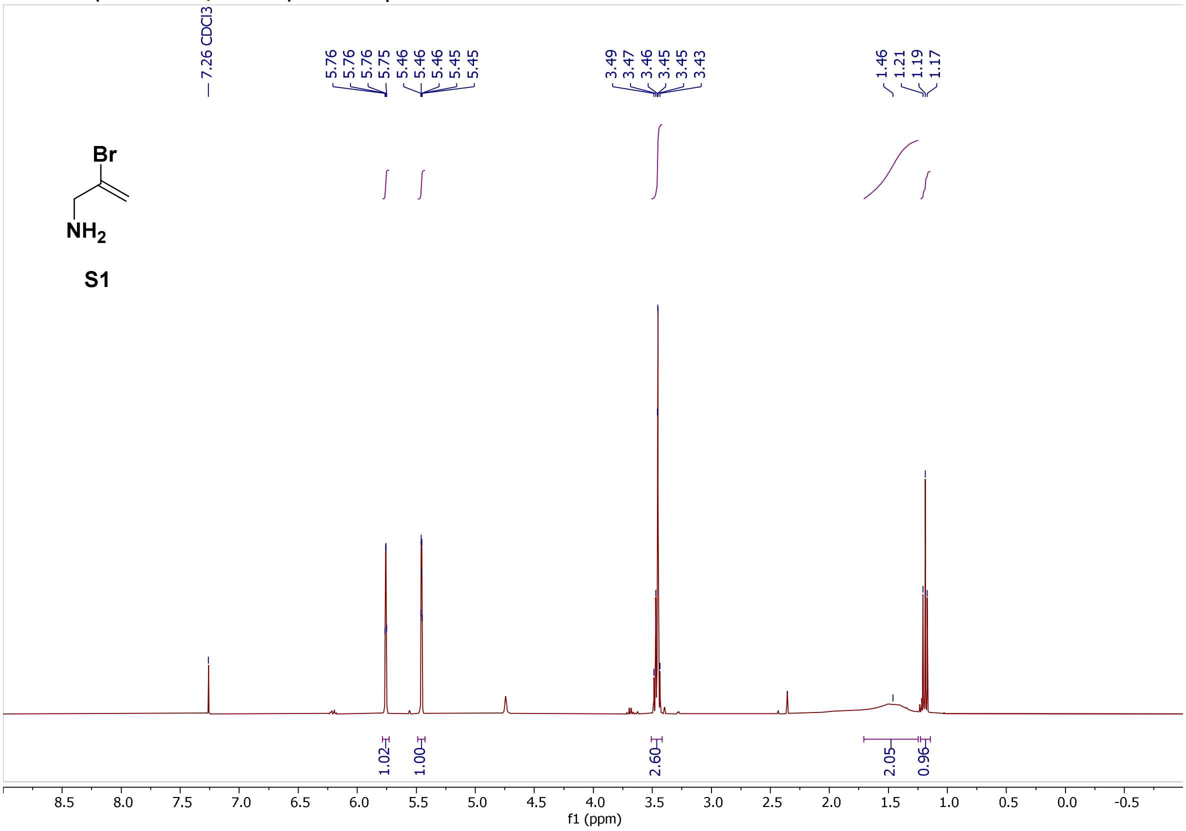

^{13}C NMR (101 MHz, $CDCl_3$) of compound S1**.**

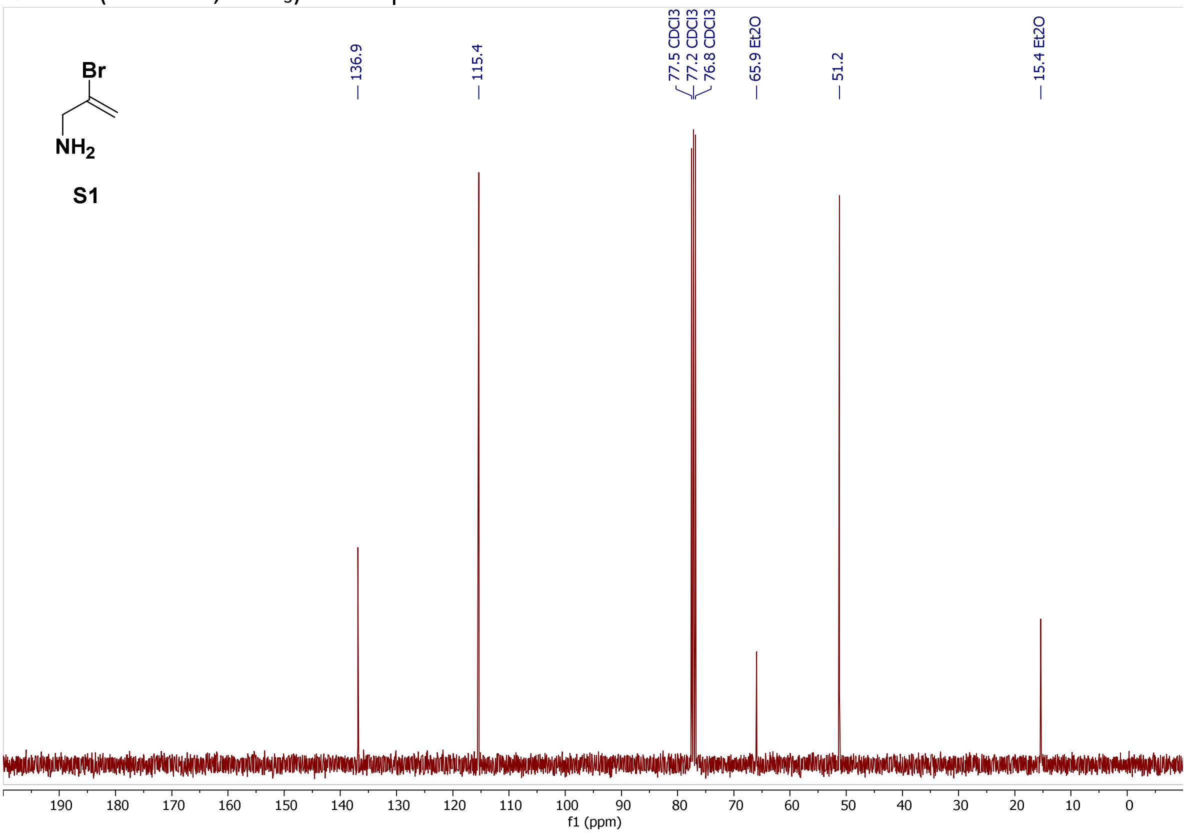

^{1}H NMR (400 MHz, $CDCl_3$) of compound **S2.**

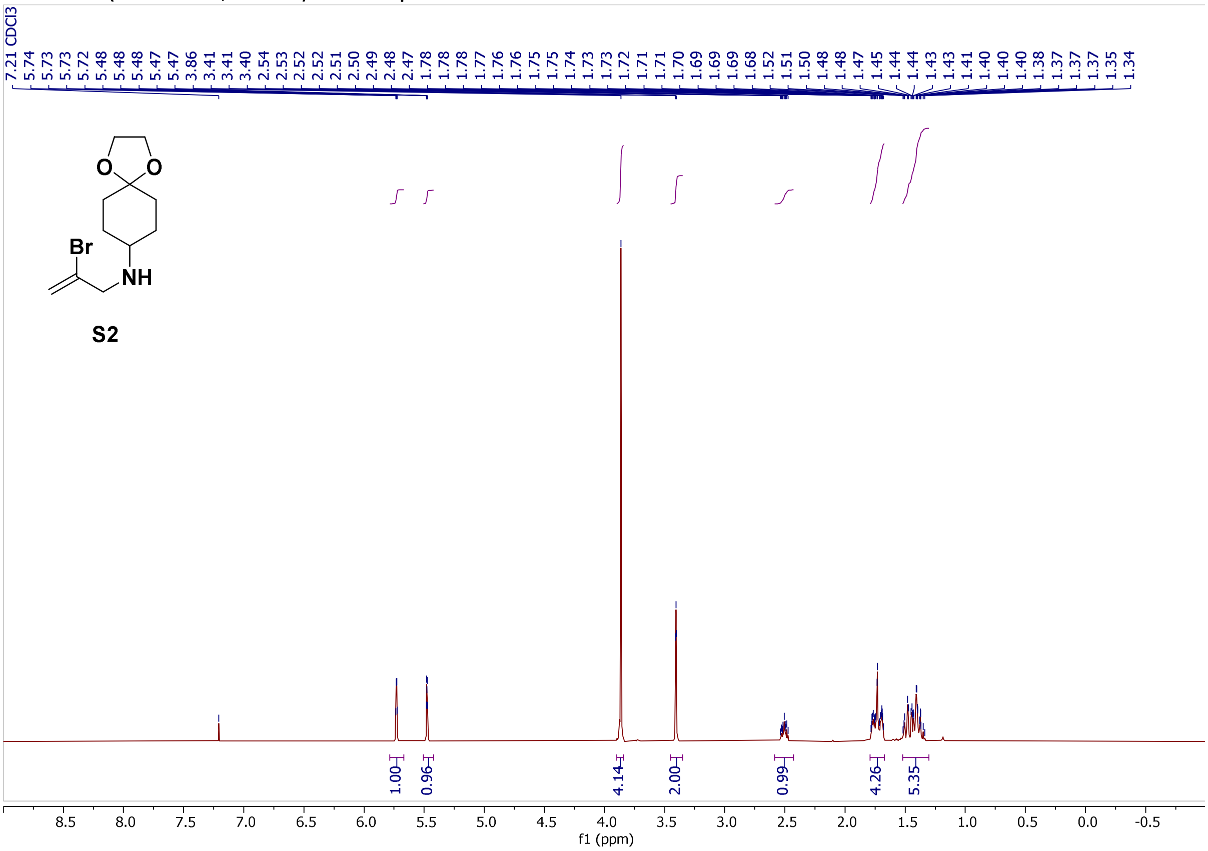

^{13}C NMR (101 MHz, $CDCl_3$) of compound **S2.**

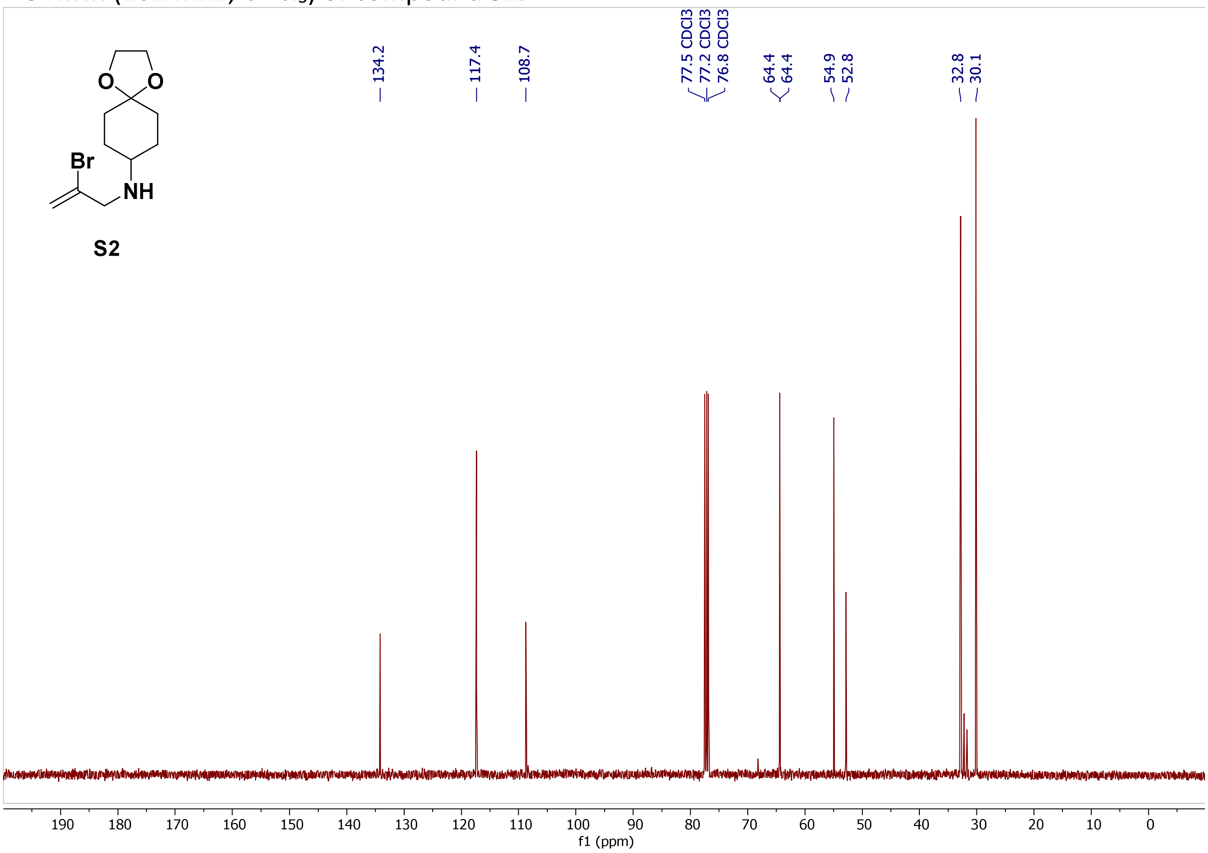

^{1}H NMR (400 MHz, $CDCl_3$) of compound **S3.**

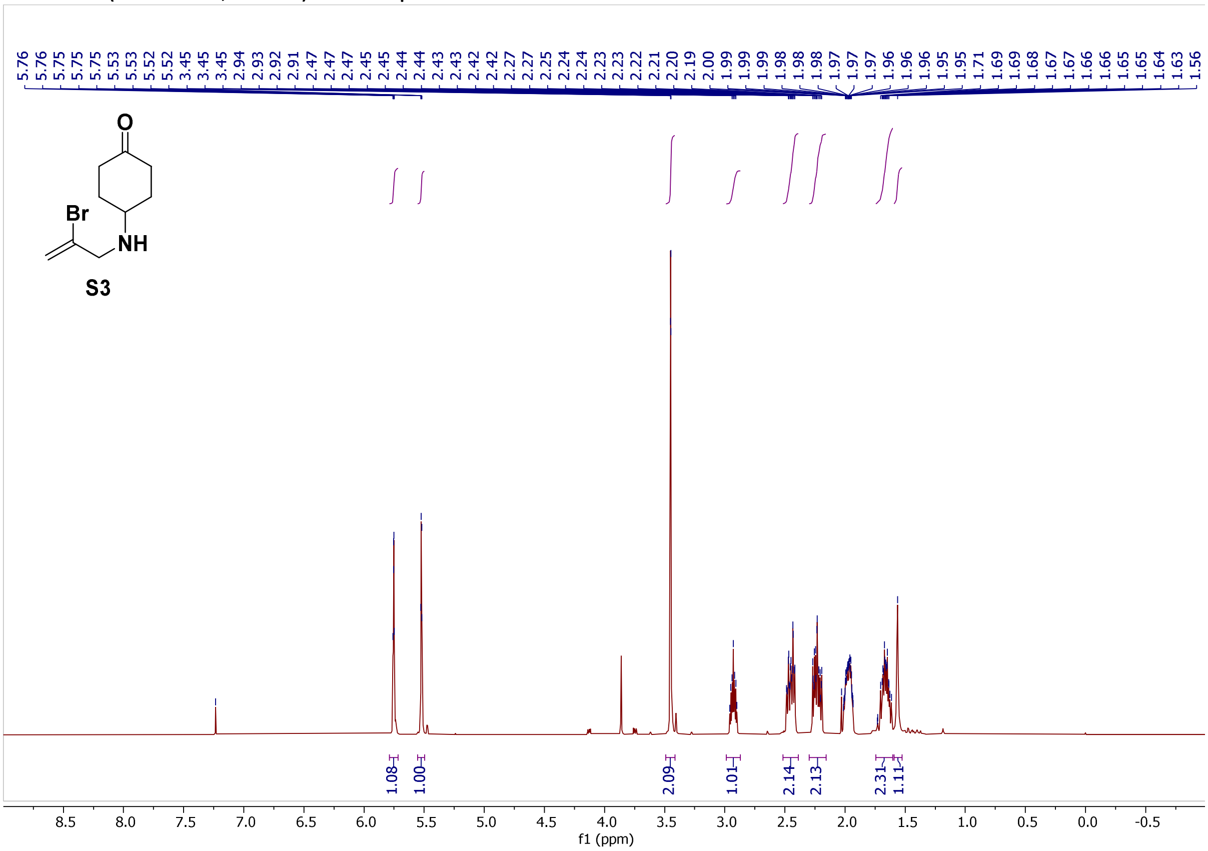

^{13}C NMR (101 MHz, $CDCl_3$) of compound **S3.**

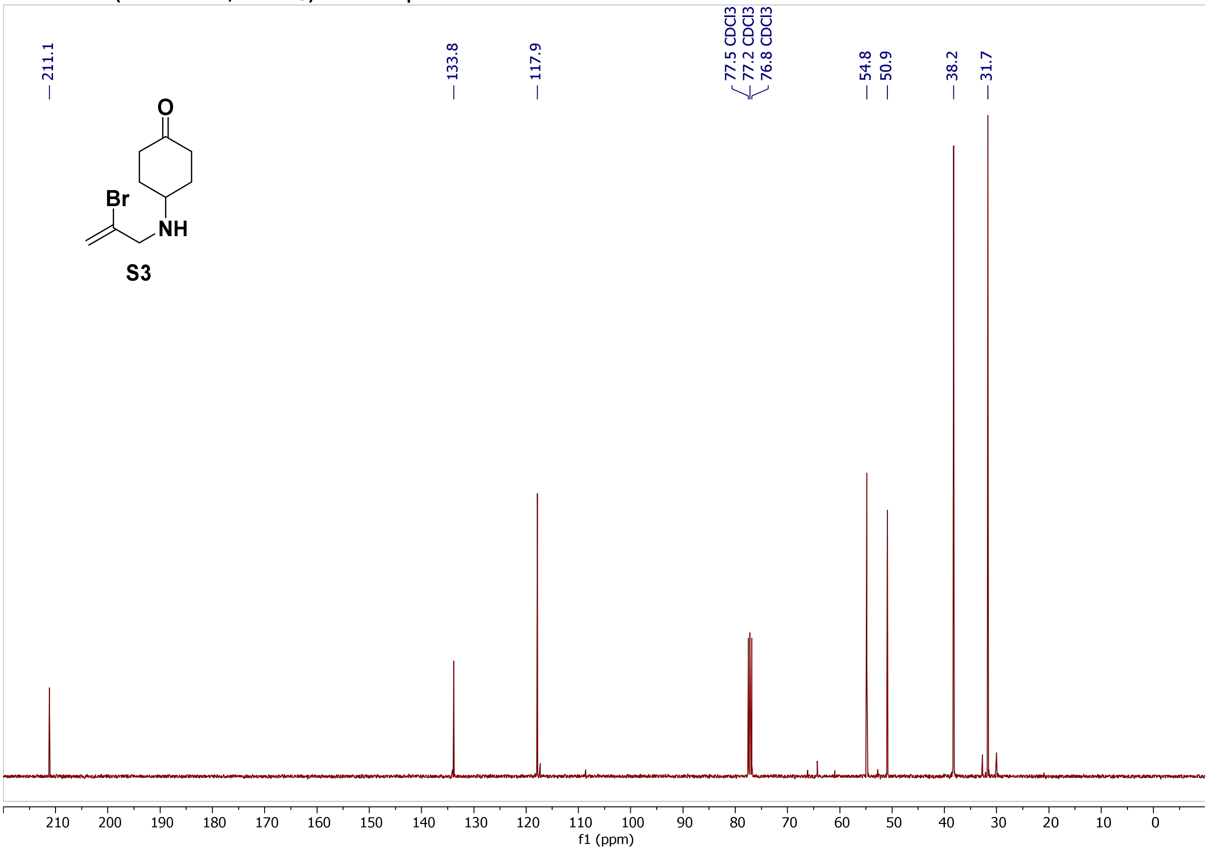

^{1}H NMR (400 MHz, $CDCl_3$) of compound **11a.**

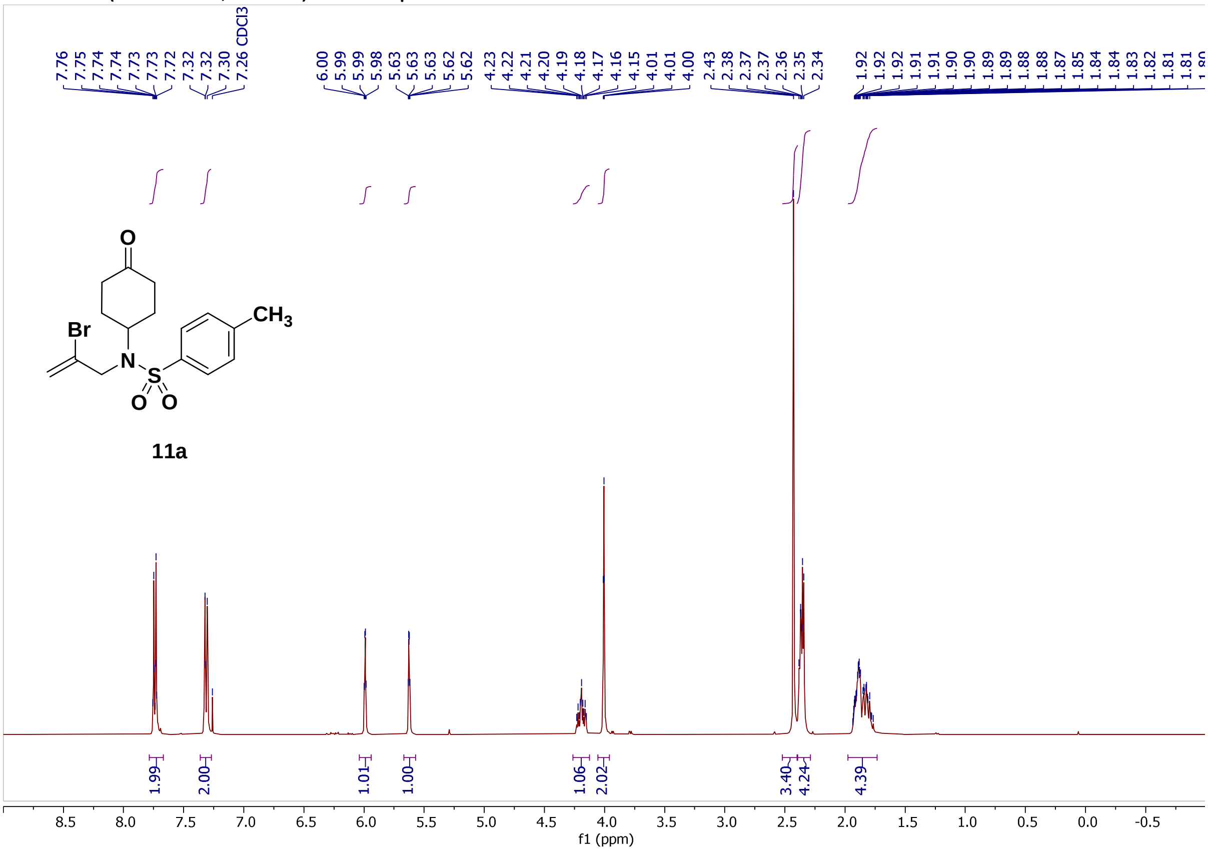

^{13}C NMR (101 MHz, $CDCl_3$) of compound **11a.**

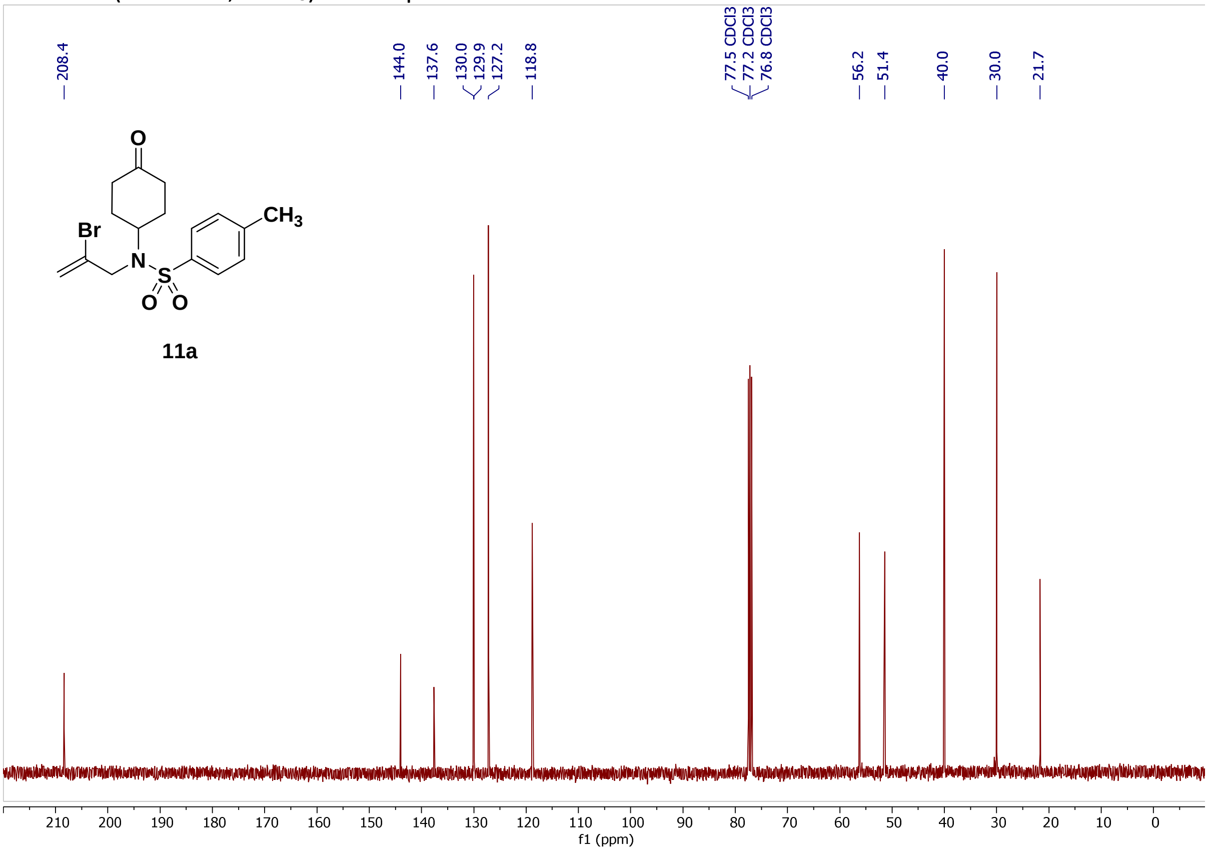

^{1}**H NMR** (400 MHz, $CDCl_3$) of compound **11b.**

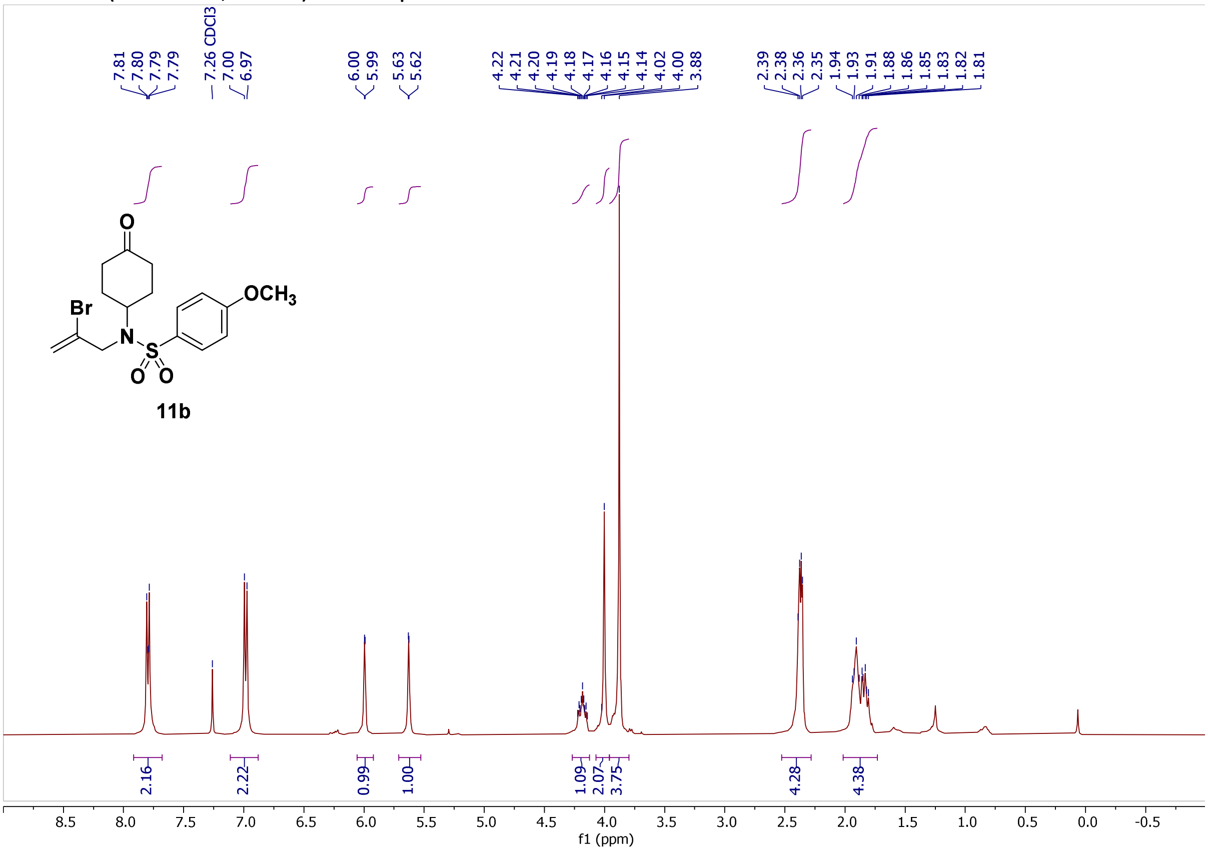

^{13}**C NMR** (101 MHz, $CDCl_3$) of compound **11b.**

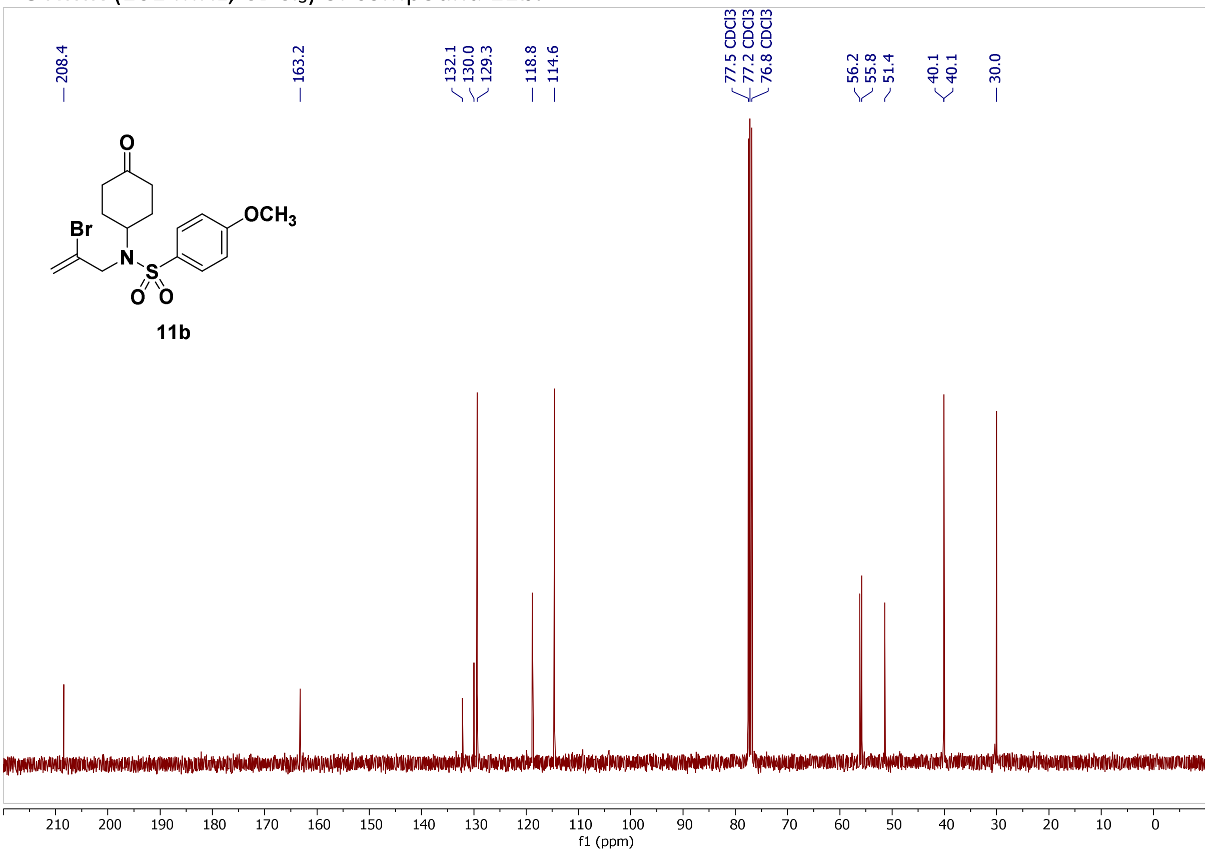

^{1}H NMR (400 MHz, $CDCl_3$) of compound **11c.**

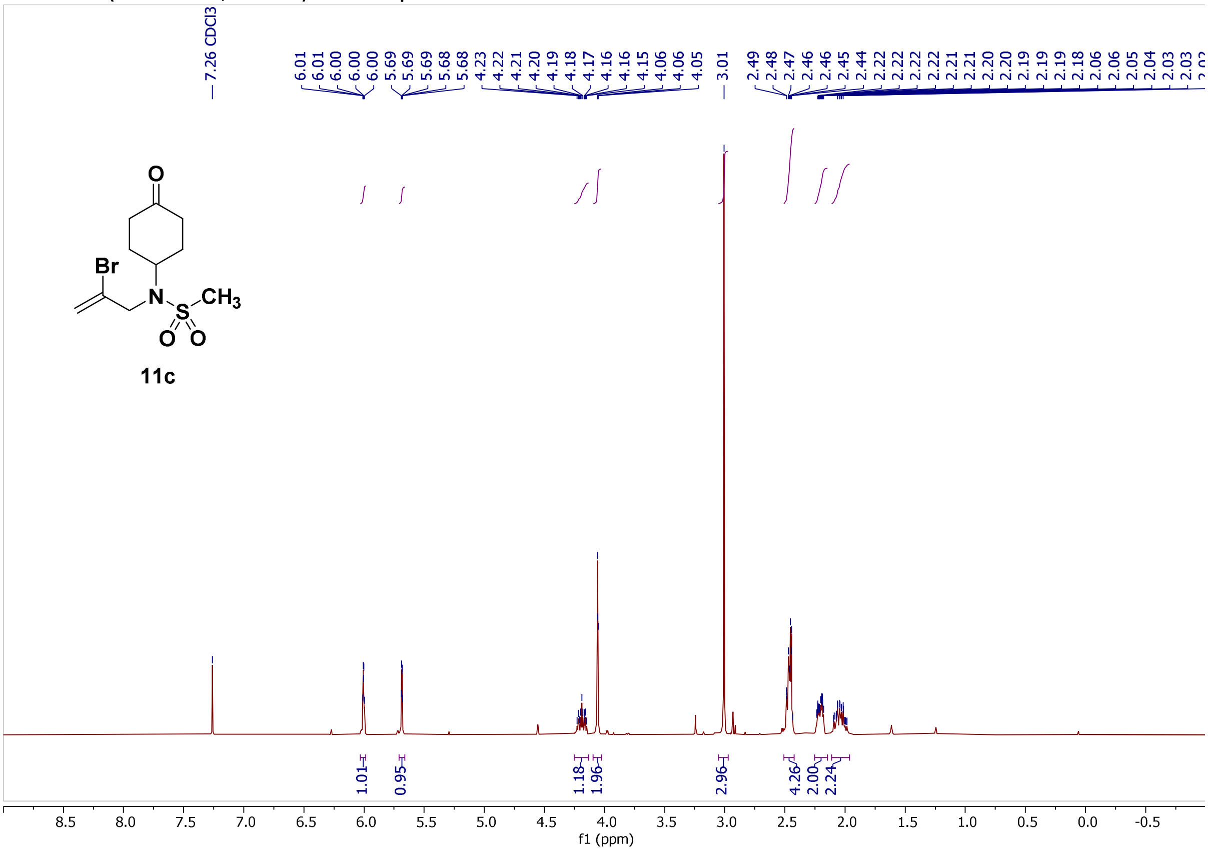

^{13}C NMR (101 MHz, $CDCl_3$) of compound **11c.**

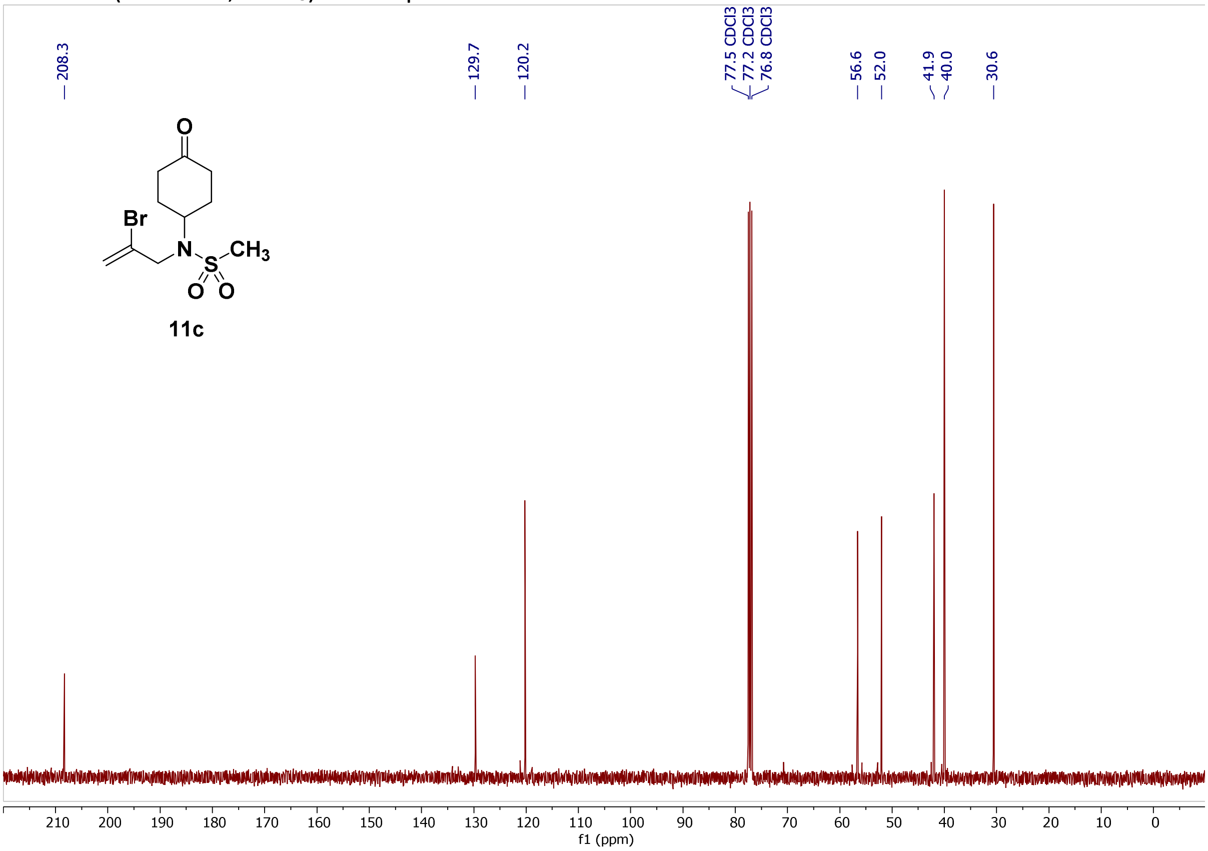

^{1}**H NMR** (400 MHz, $CDCl_3$) of compound **11d.**

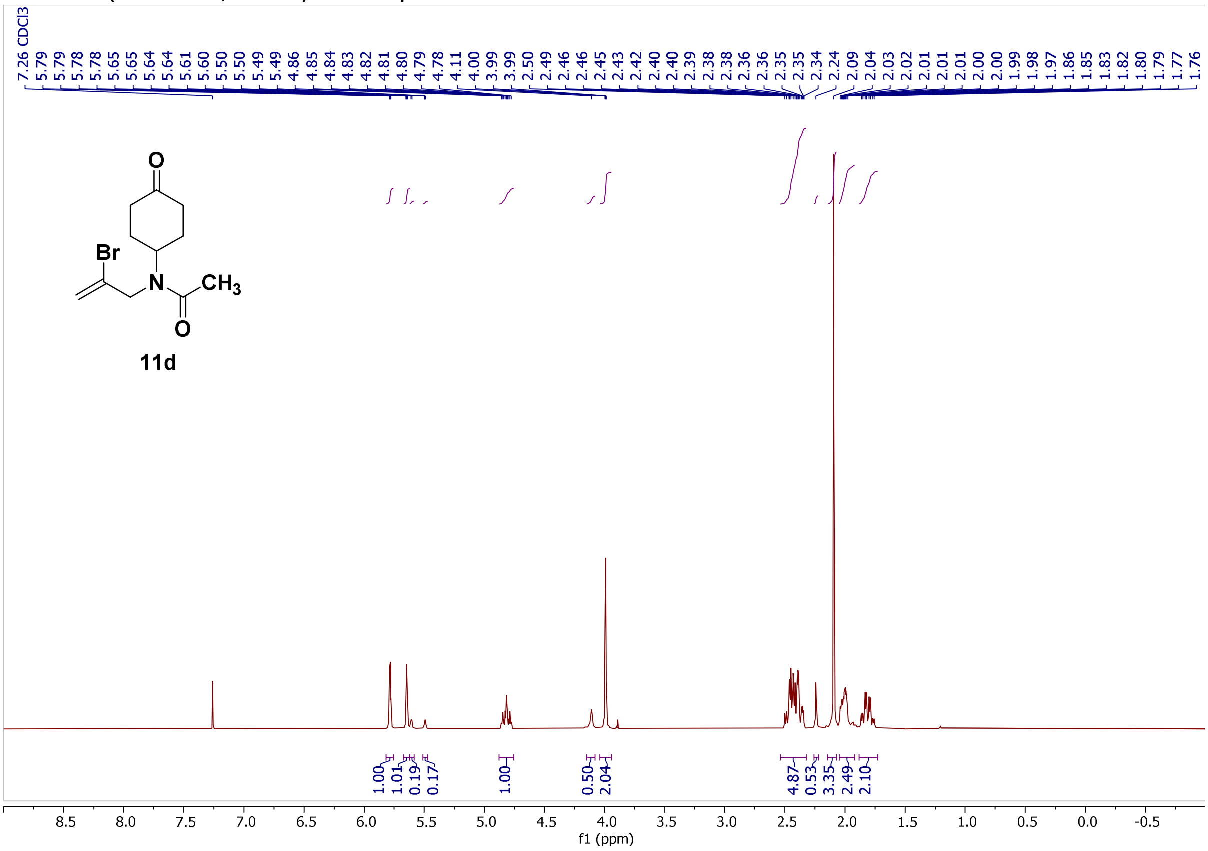

^{13}**C NMR** (101 MHz, $CDCl_3$) of compound **11d.**

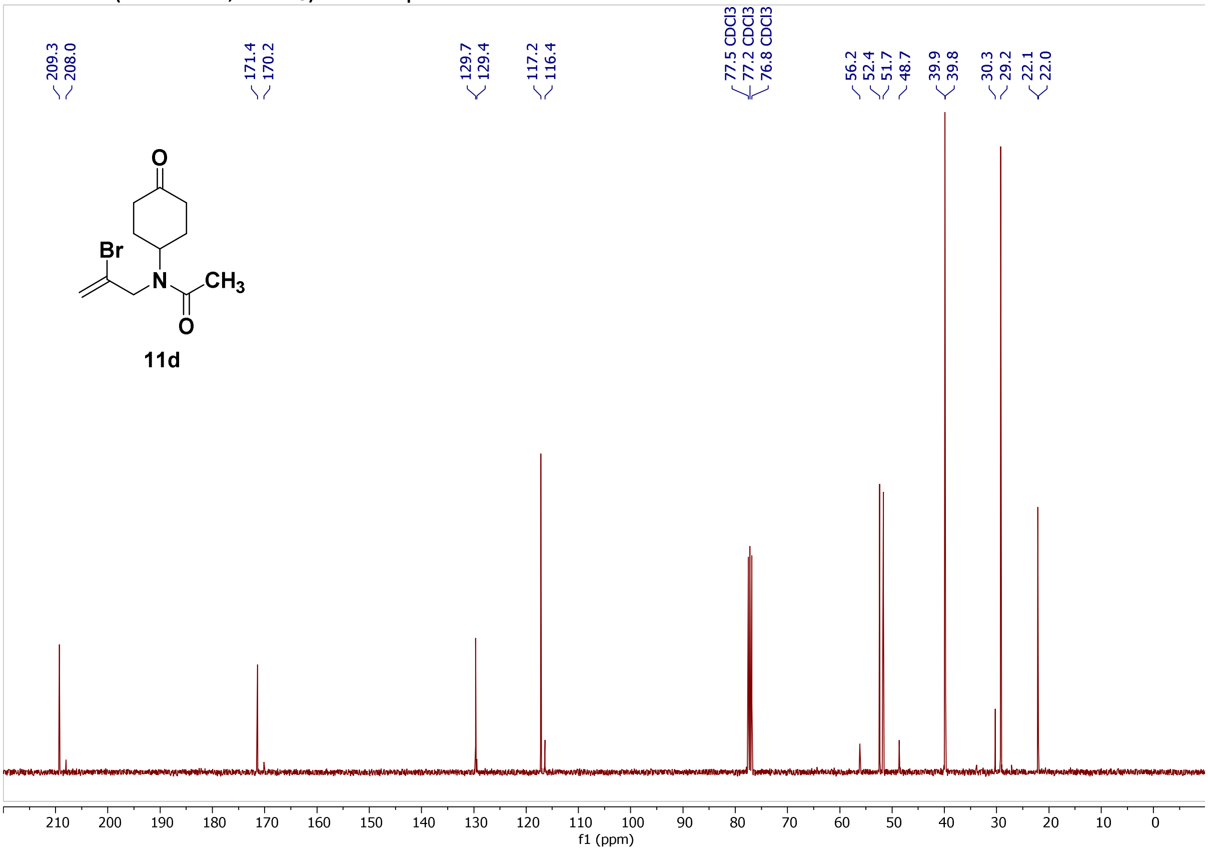

^{1}**H NMR** (400 MHz, $CDCl_3$) of compound **11e.**

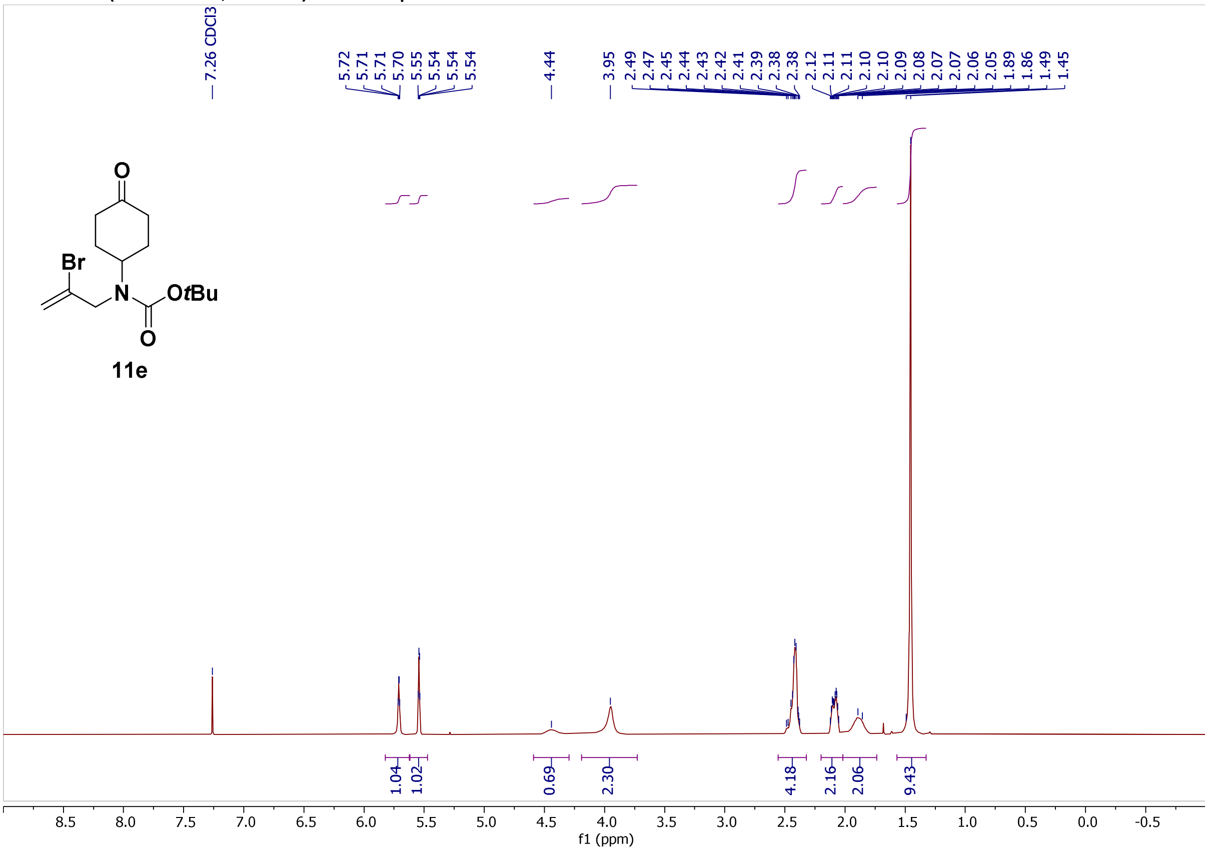

^{13}**C NMR** (101 MHz, $CDCl_3$) of compound **11e.**

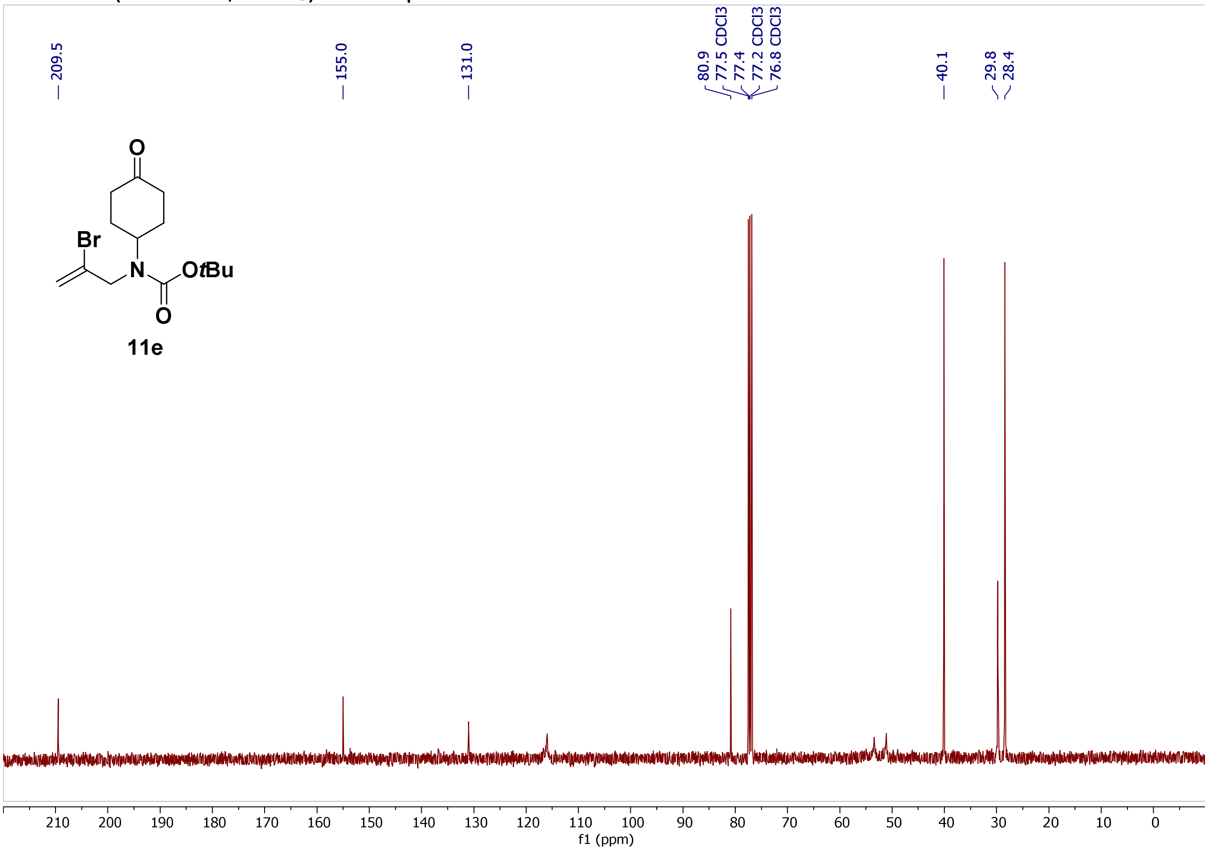

^{1}H NMR (400 MHz, $CDCl_3$) of compound **11f.**

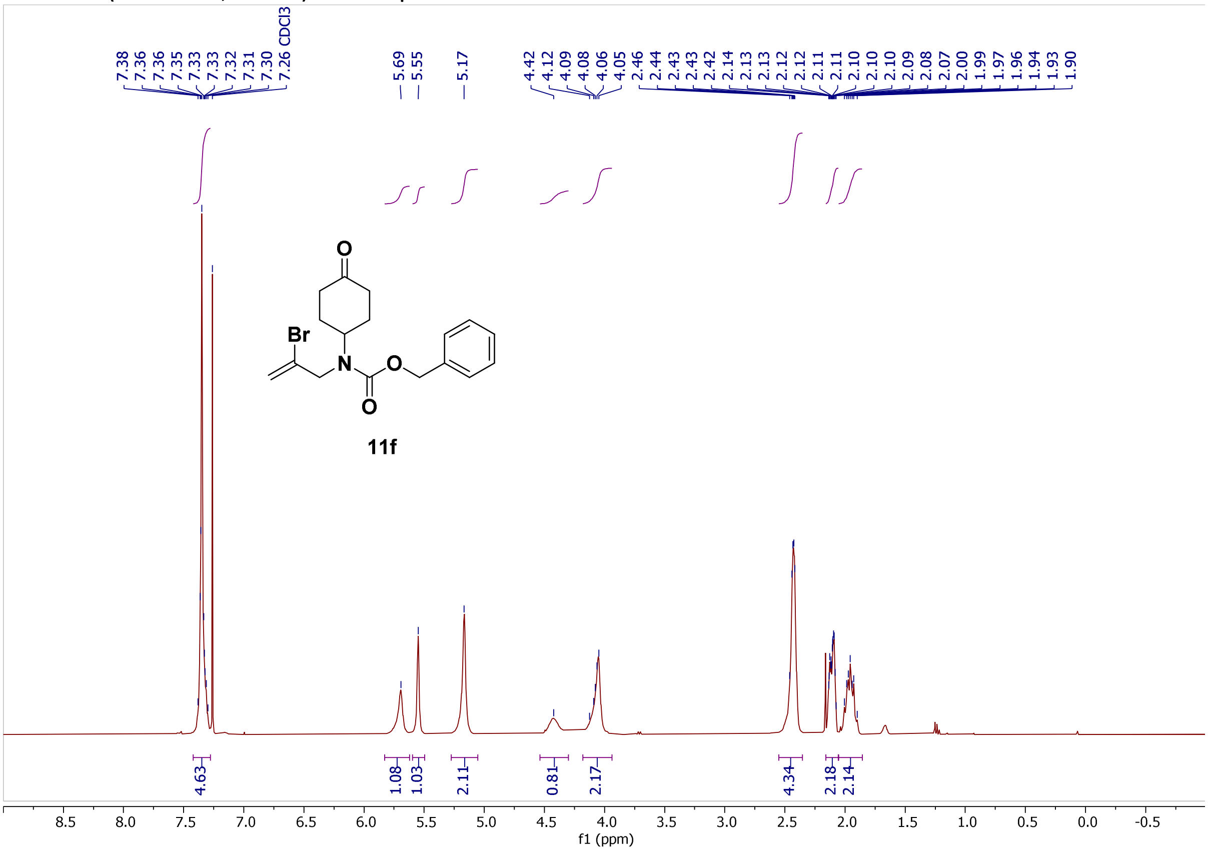

^{13}C NMR (101 MHz, $CDCl_3$) of compound **11f.**

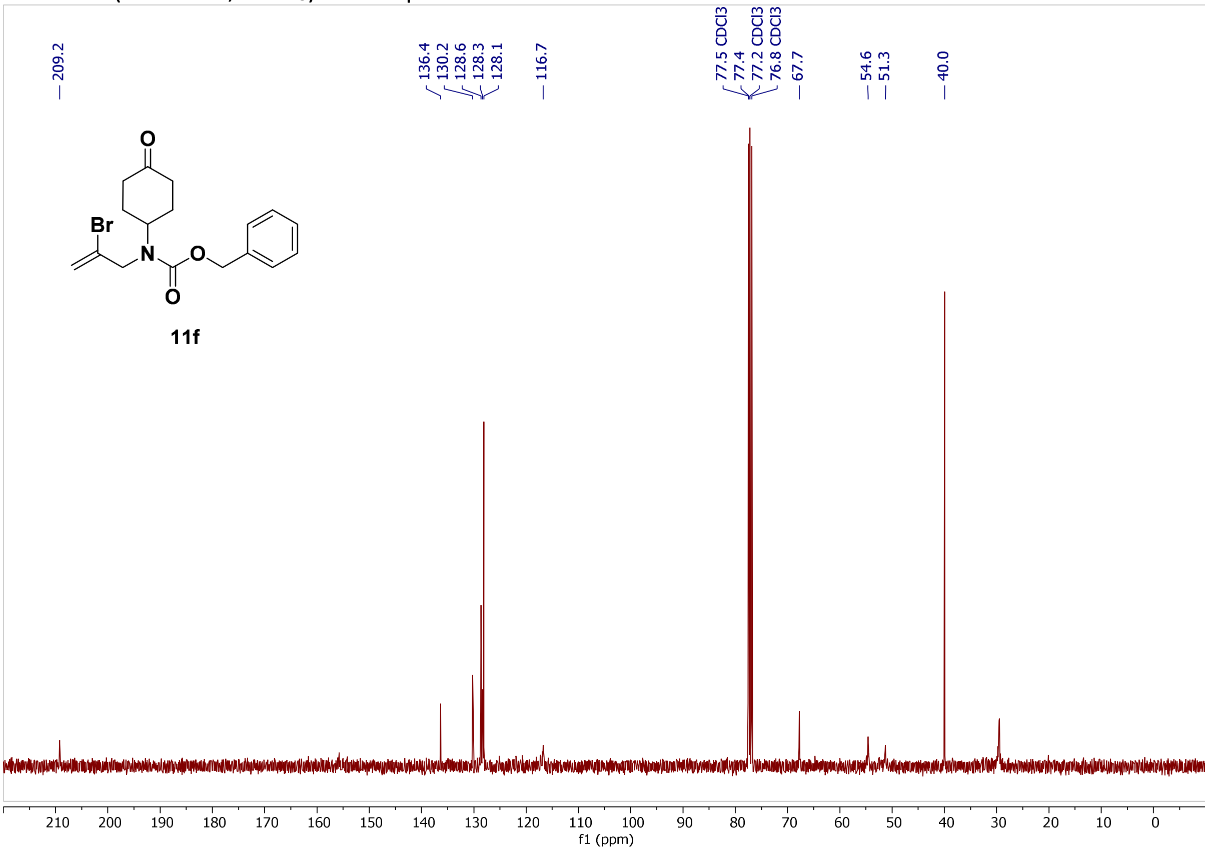

^{1}H NMR (400 MHz, $CDCl_3$) of compound **11g.**

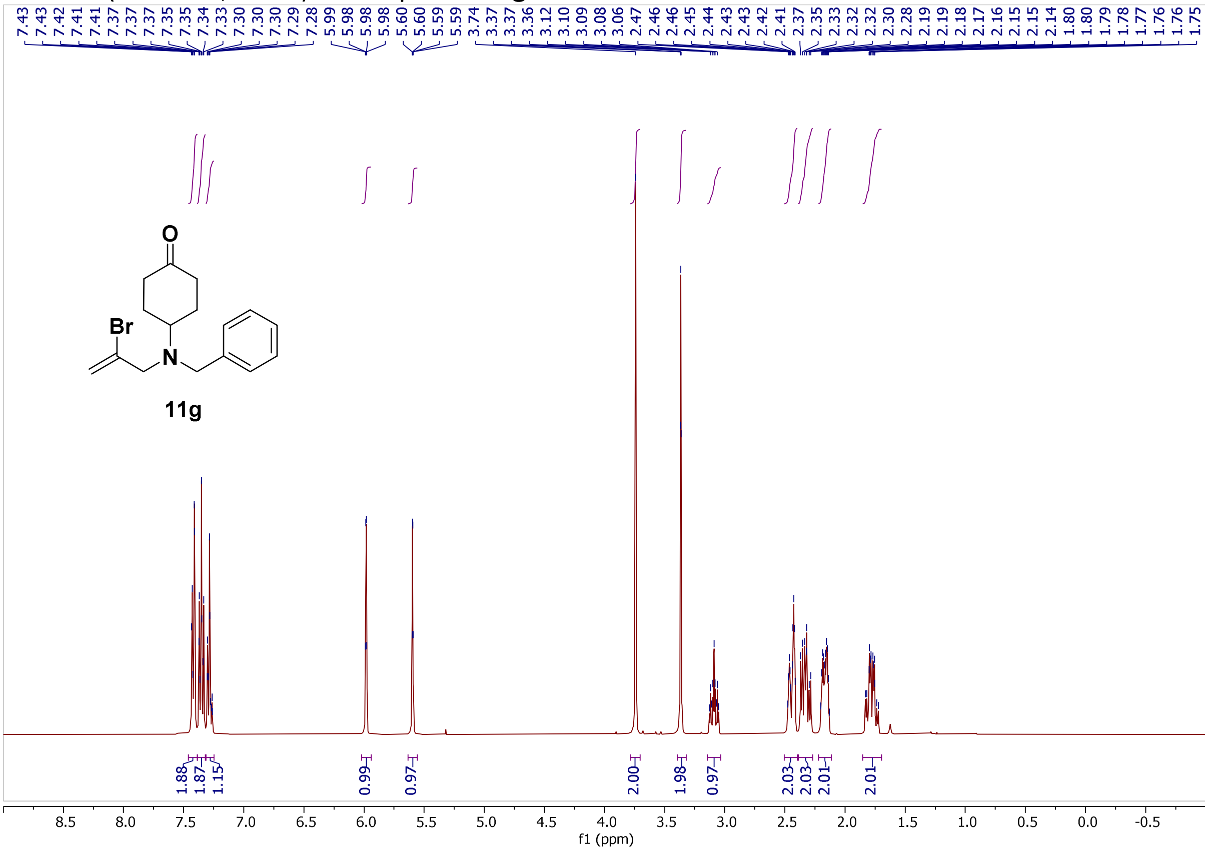

^{13}C NMR (101 MHz, $CDCl_3$) of compound **11g.**

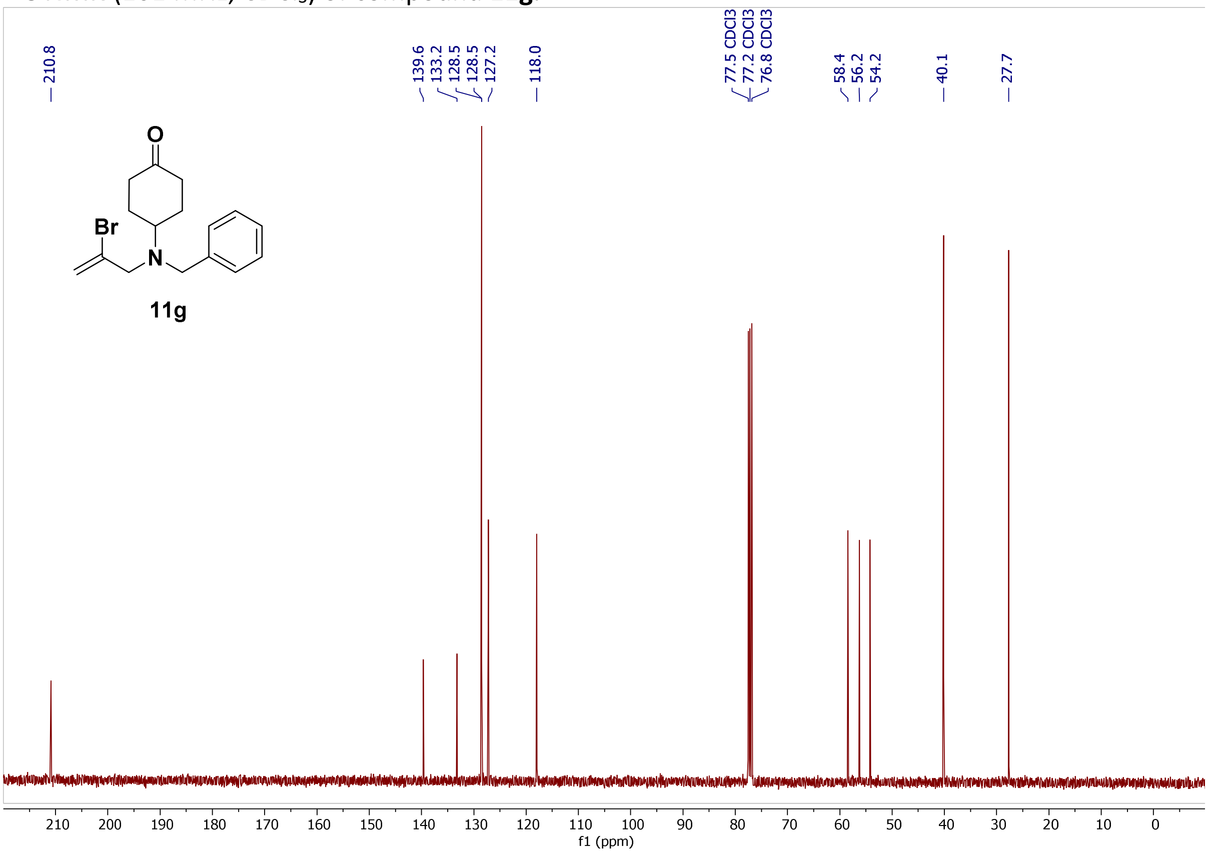

^{1}**H NMR** (400 MHz, $CDCl_3$) of compound **S5.**

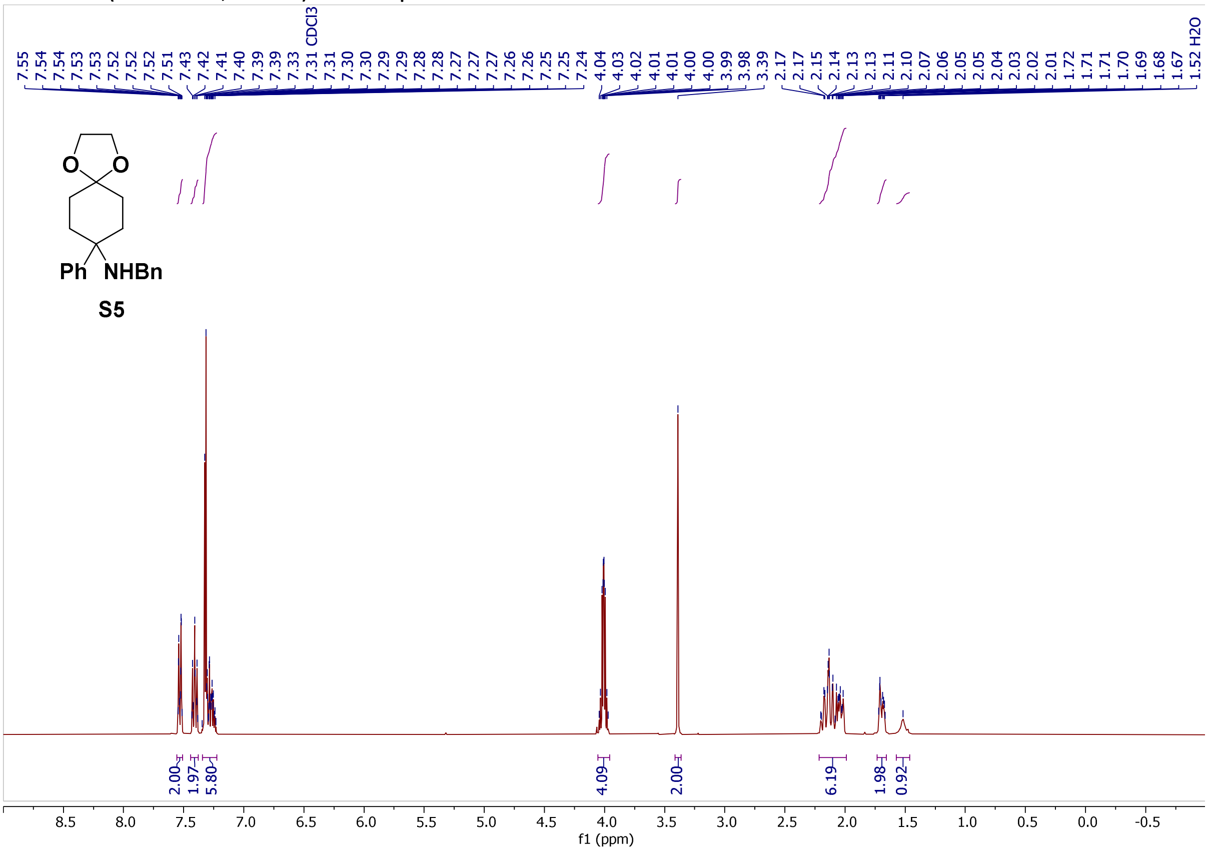

^{13}**C NMR** (101 MHz, $CDCl_3$) of compound **S5.**

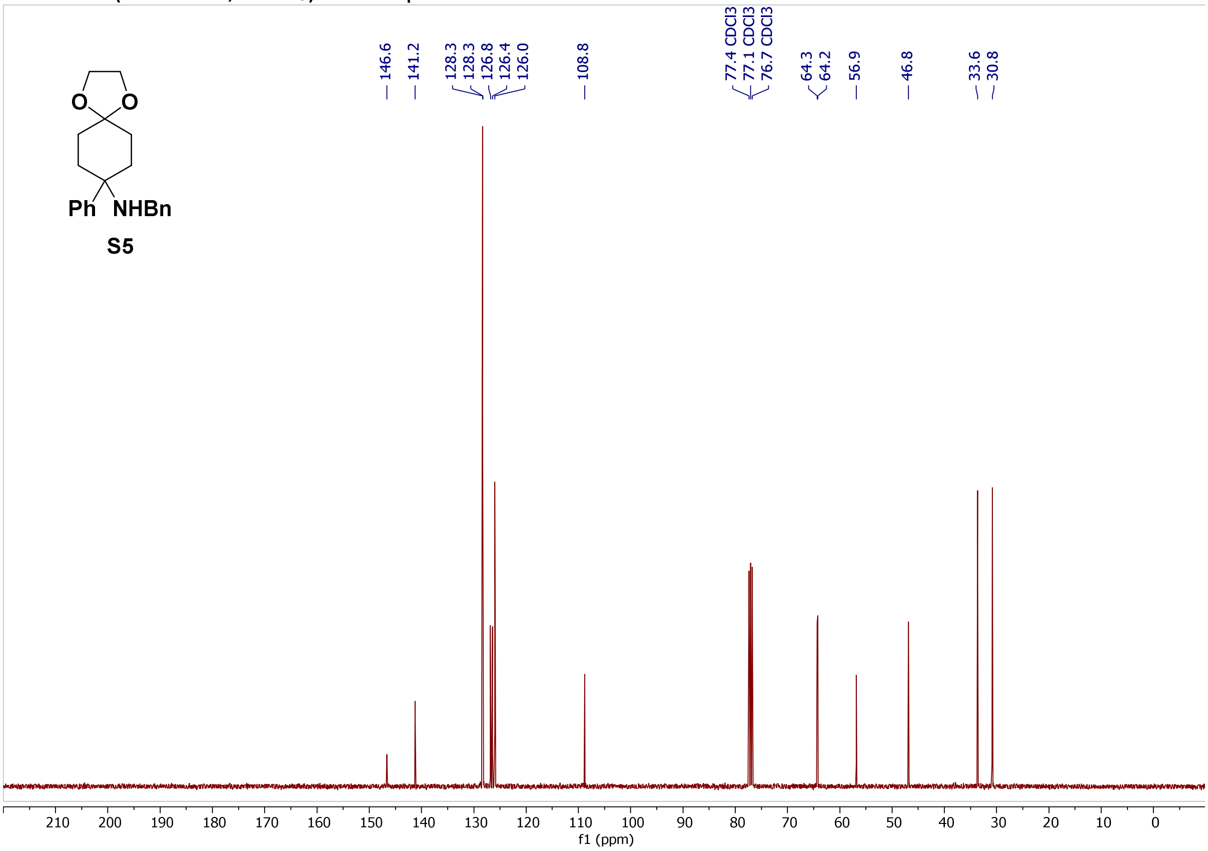

[1]**H NMR** (400 MHz, CDCl₃) of compound **S6.**

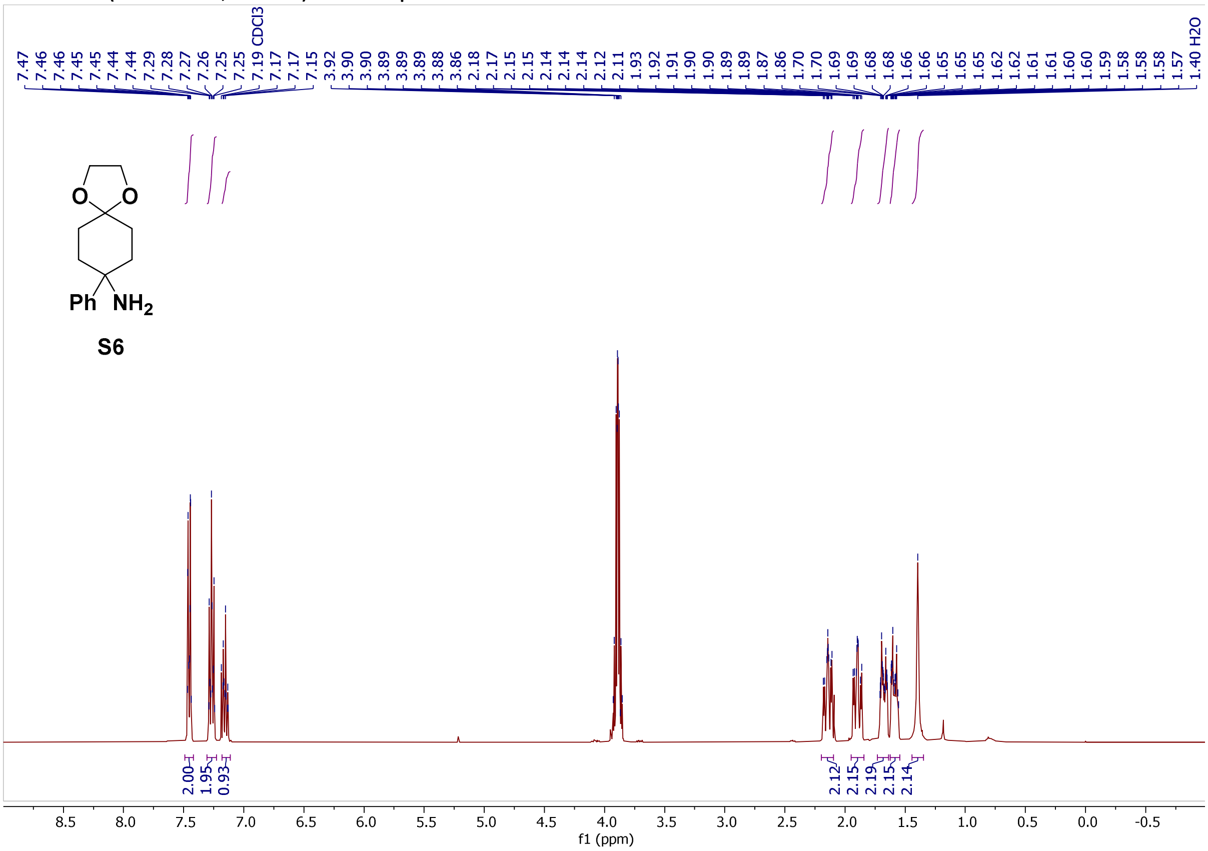

[13]**C NMR** (101 MHz, CDCl₃) of compound **S6.**

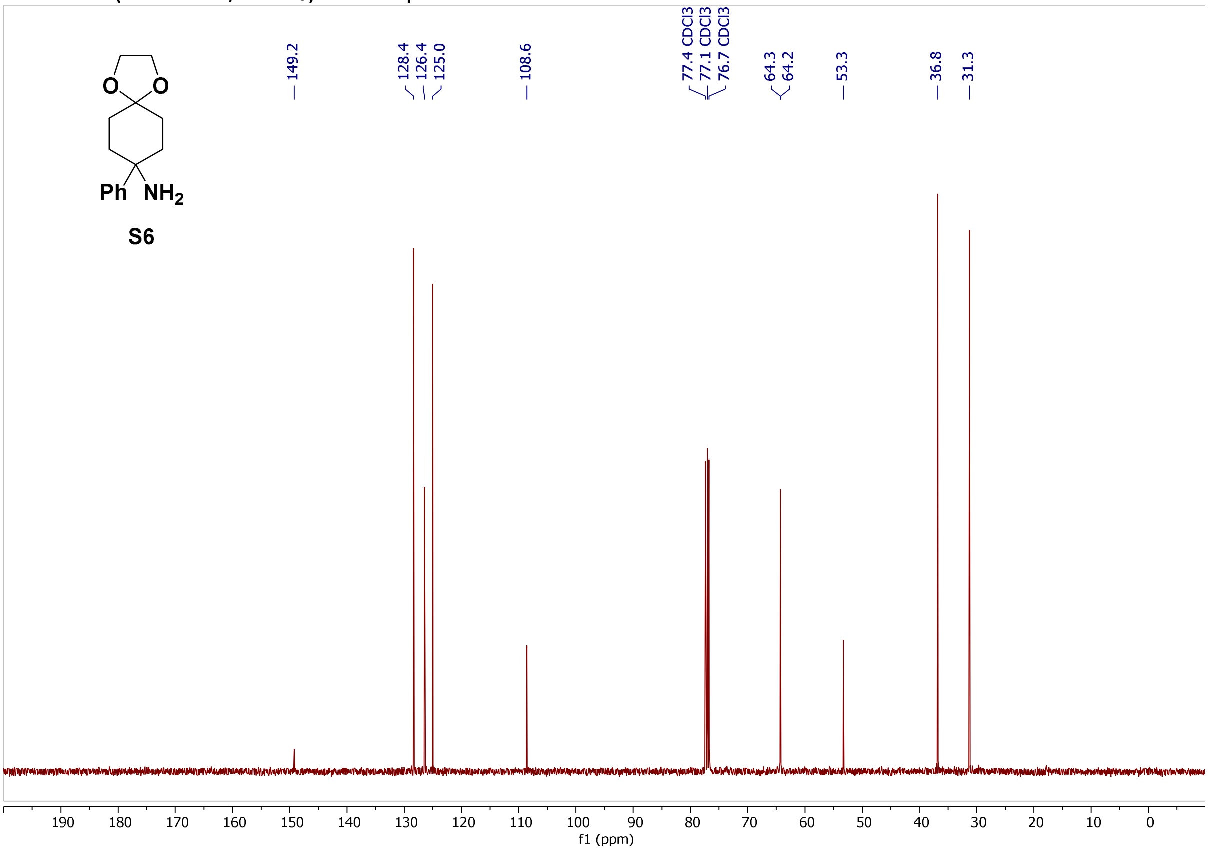

^{1}**H NMR** (400 MHz, $CDCl_3$) of compound **S7.**

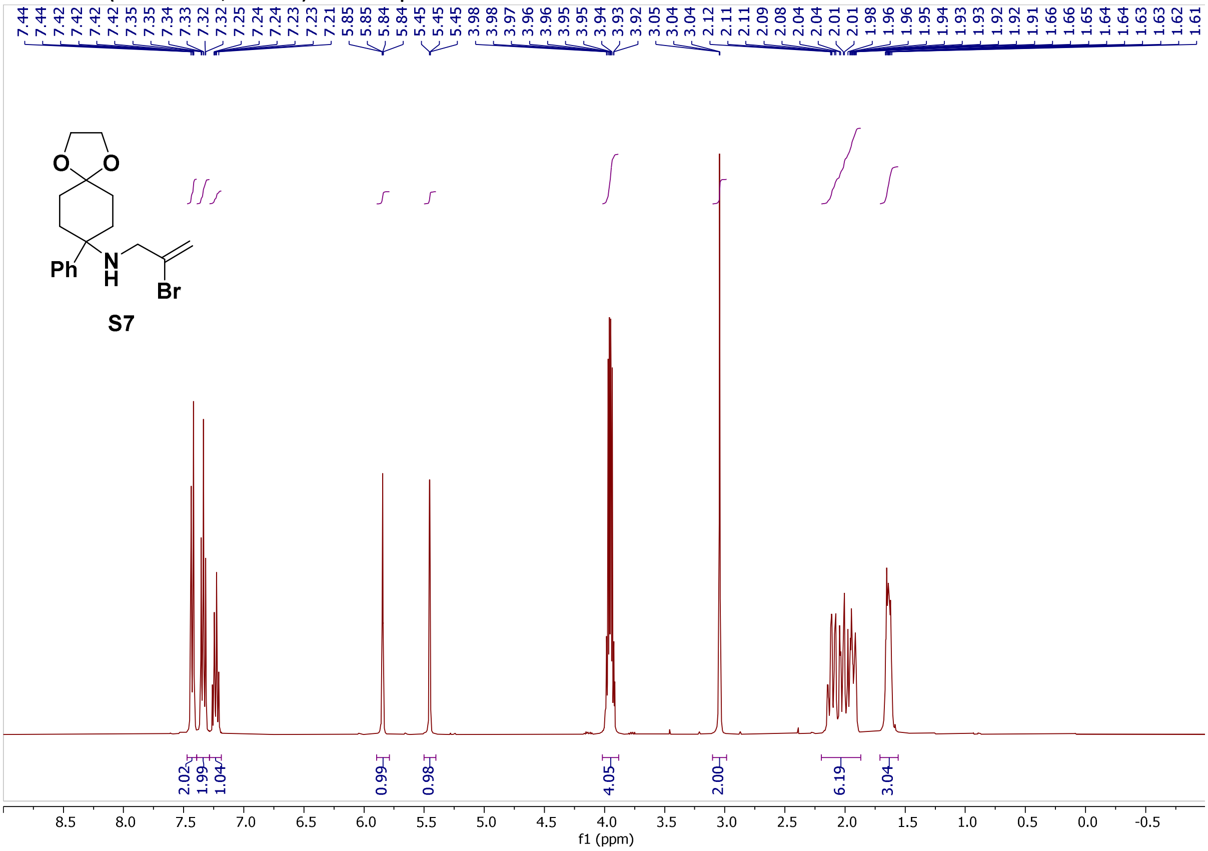

^{13}**C NMR** (101 MHz, $CDCl_3$) of compound **S7.**

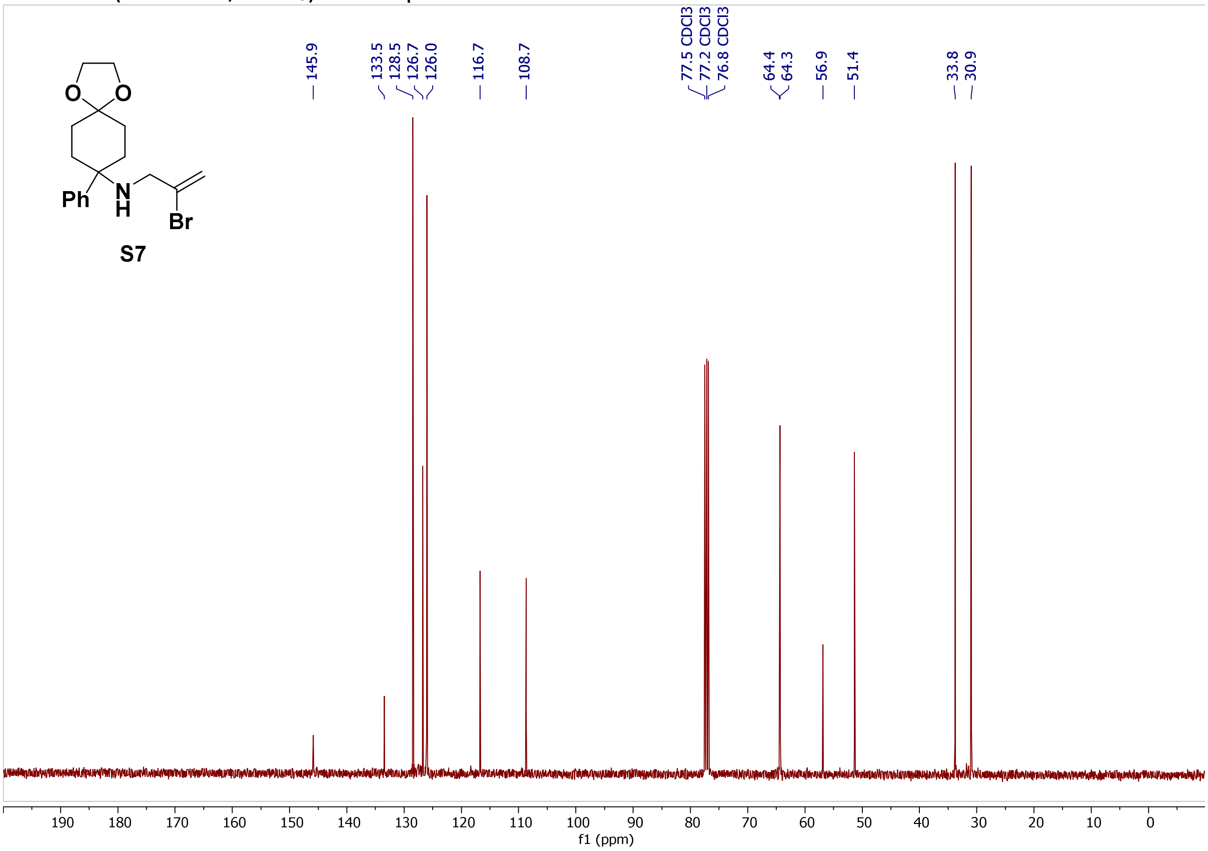

^{1}H NMR (400 MHz, $CDCl_3$) of compound **S8.**

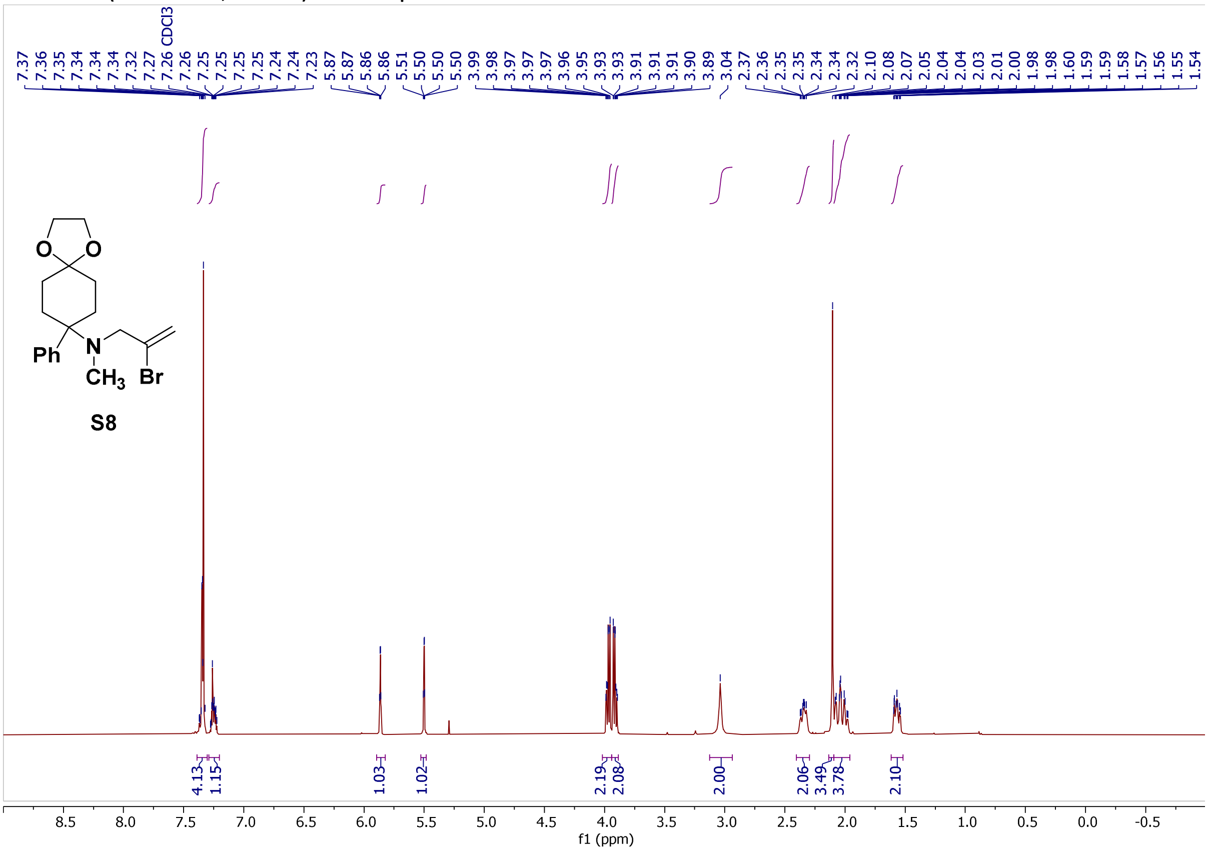

^{13}C NMR (101 MHz, $CDCl_3$) of compound **S8.**

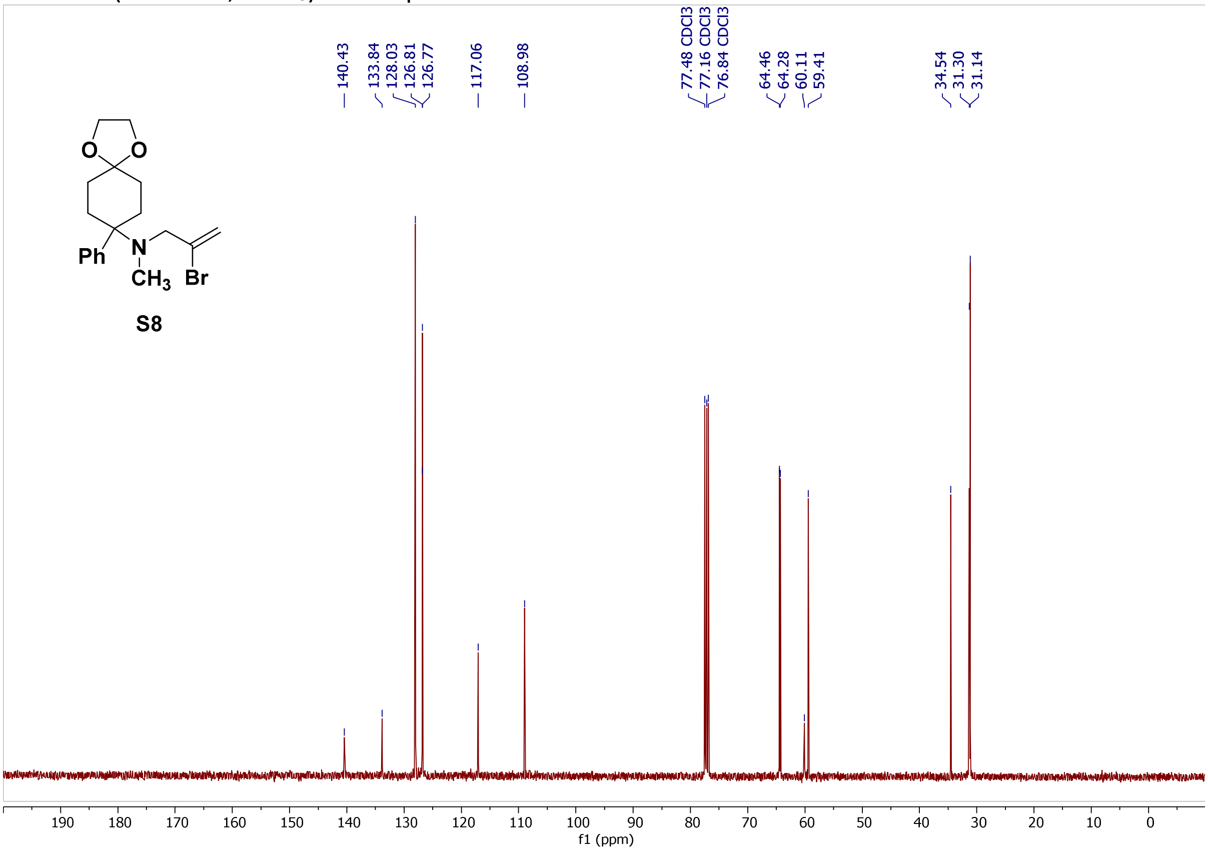

[1]**H NMR** (400 MHz, CDCl$_3$) of compound **11h.**

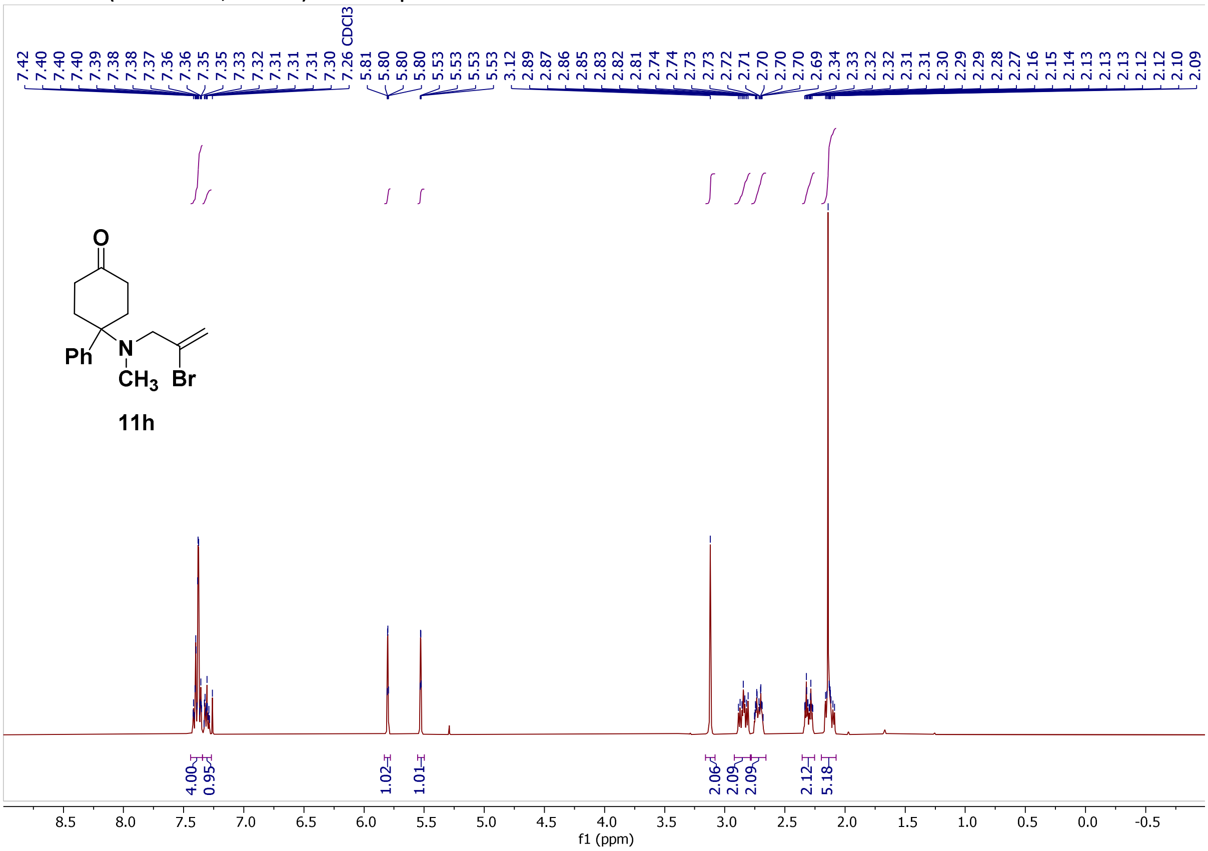

[13]**C NMR** (101 MHz, CDCl$_3$) of compound **11h.**

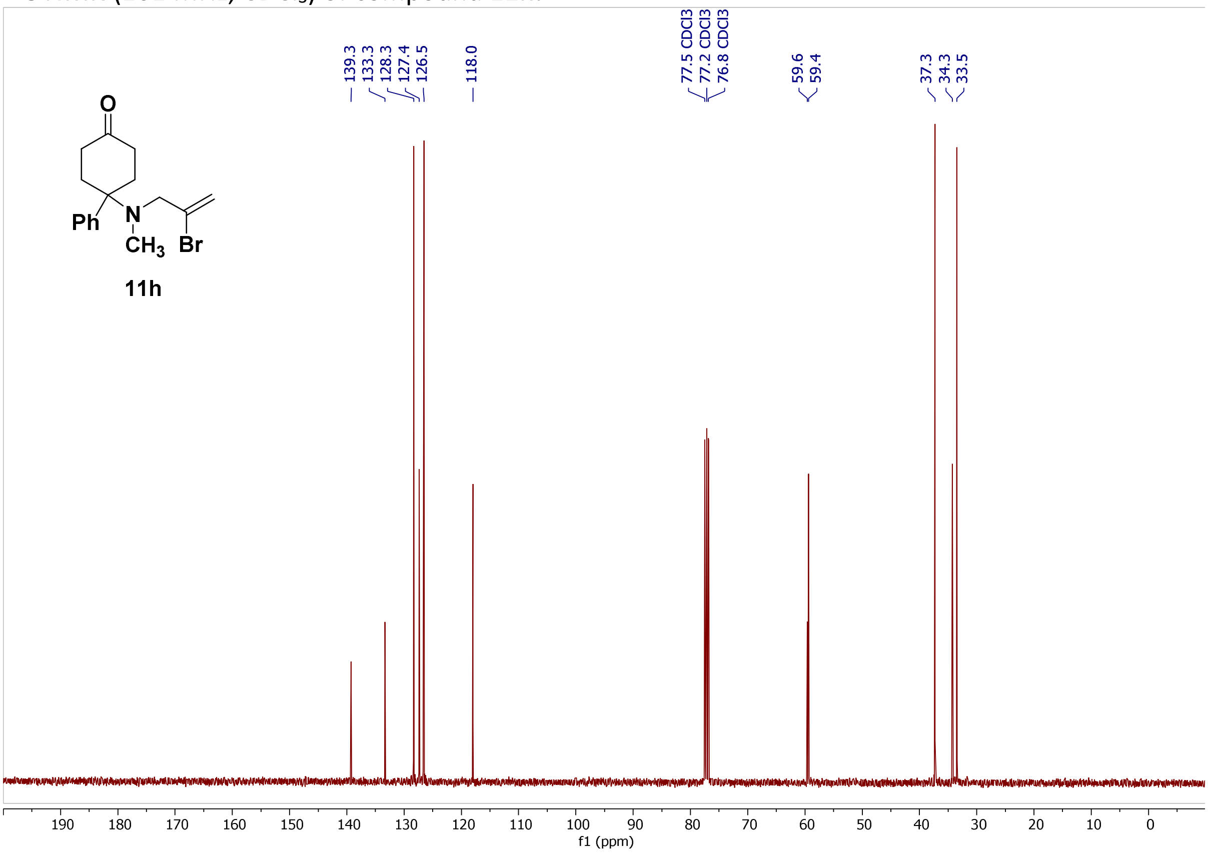

^{1}**H NMR** (400 MHz, $CDCl_3$) of compound **S9.**

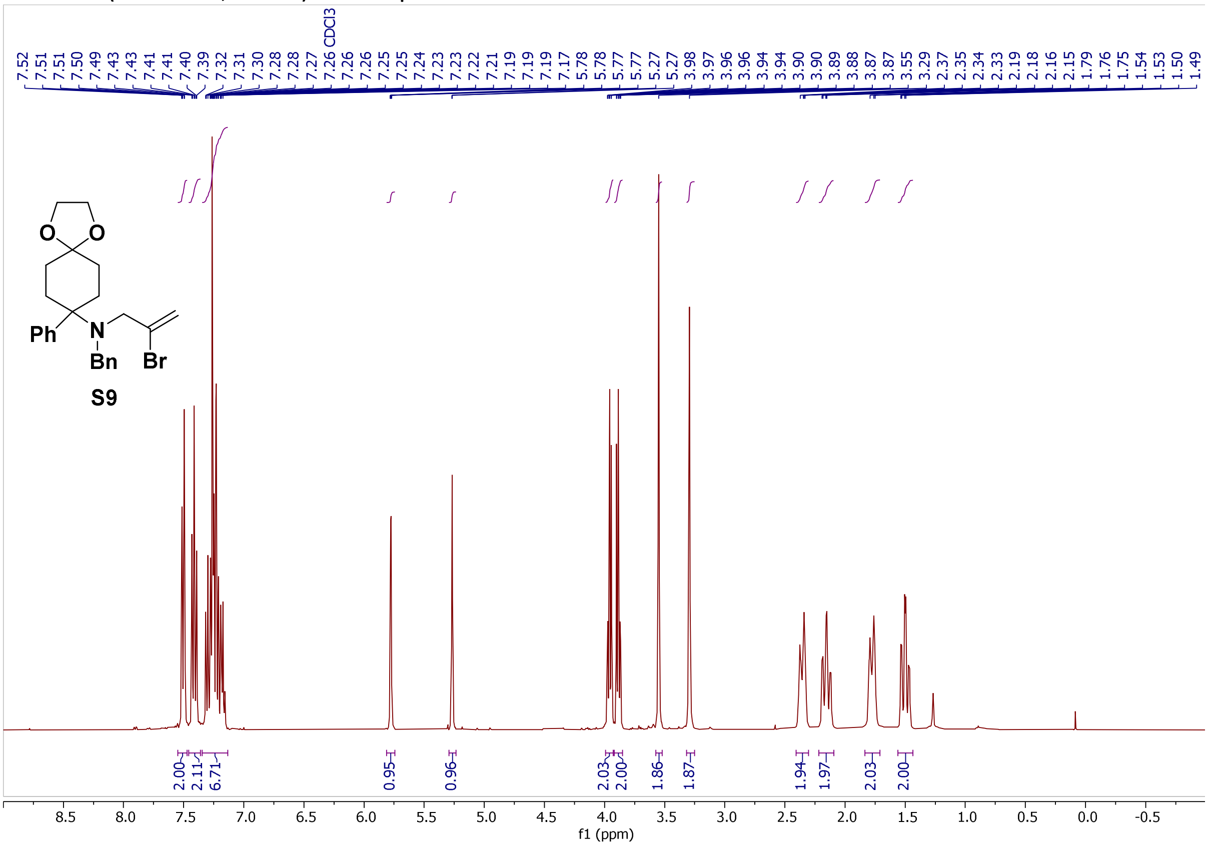

^{13}**C NMR** (101 MHz, $CDCl_3$) of compound **S9.**

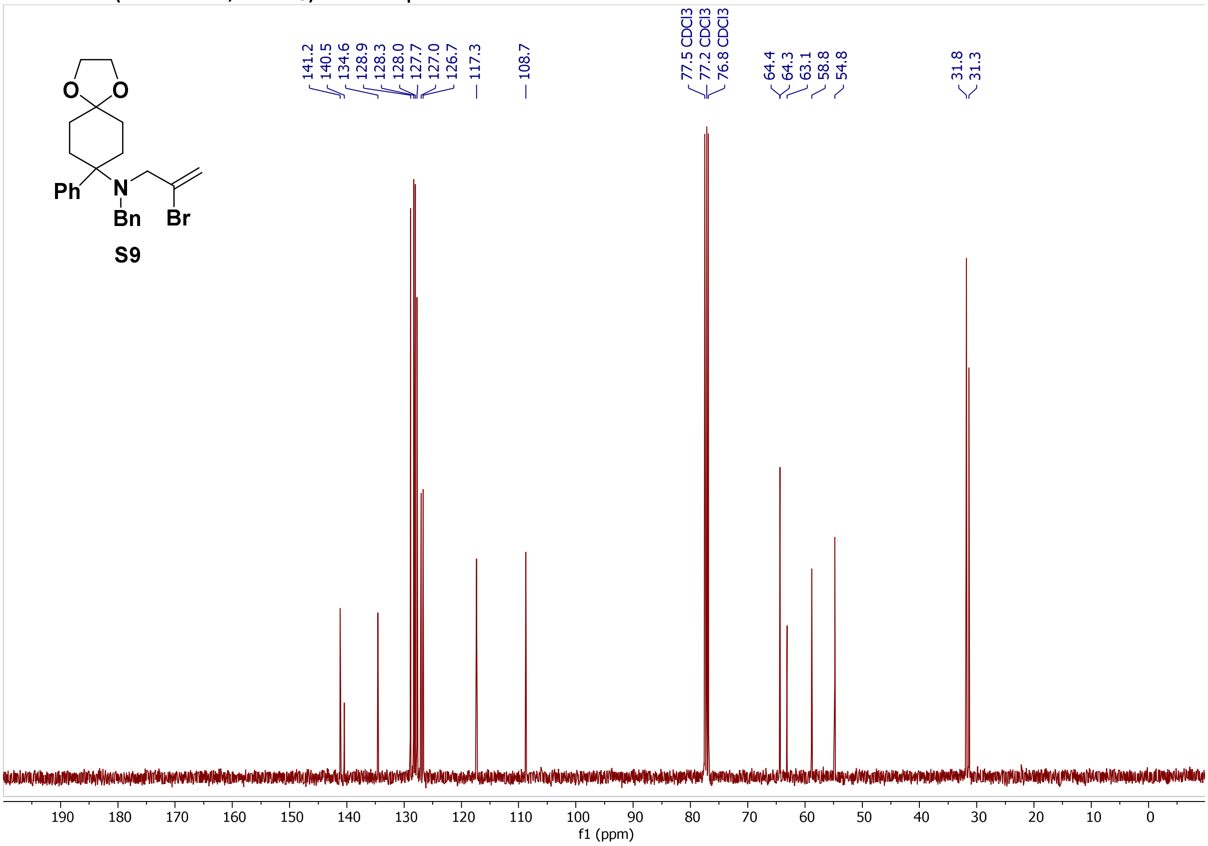

^{1}H NMR (400 MHz, $CDCl_3$) of compound **11i.**

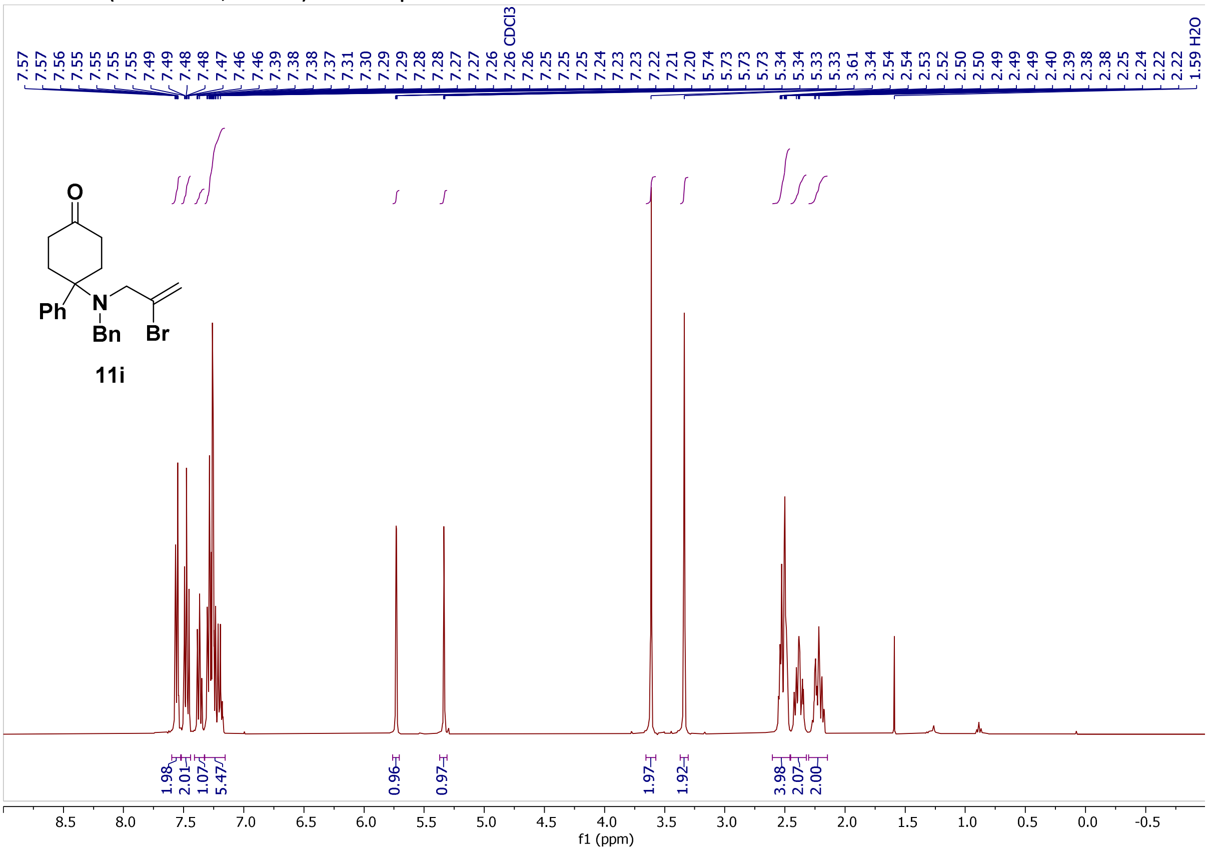

^{13}C NMR (101 MHz, $CDCl_3$) of compound **11i.**

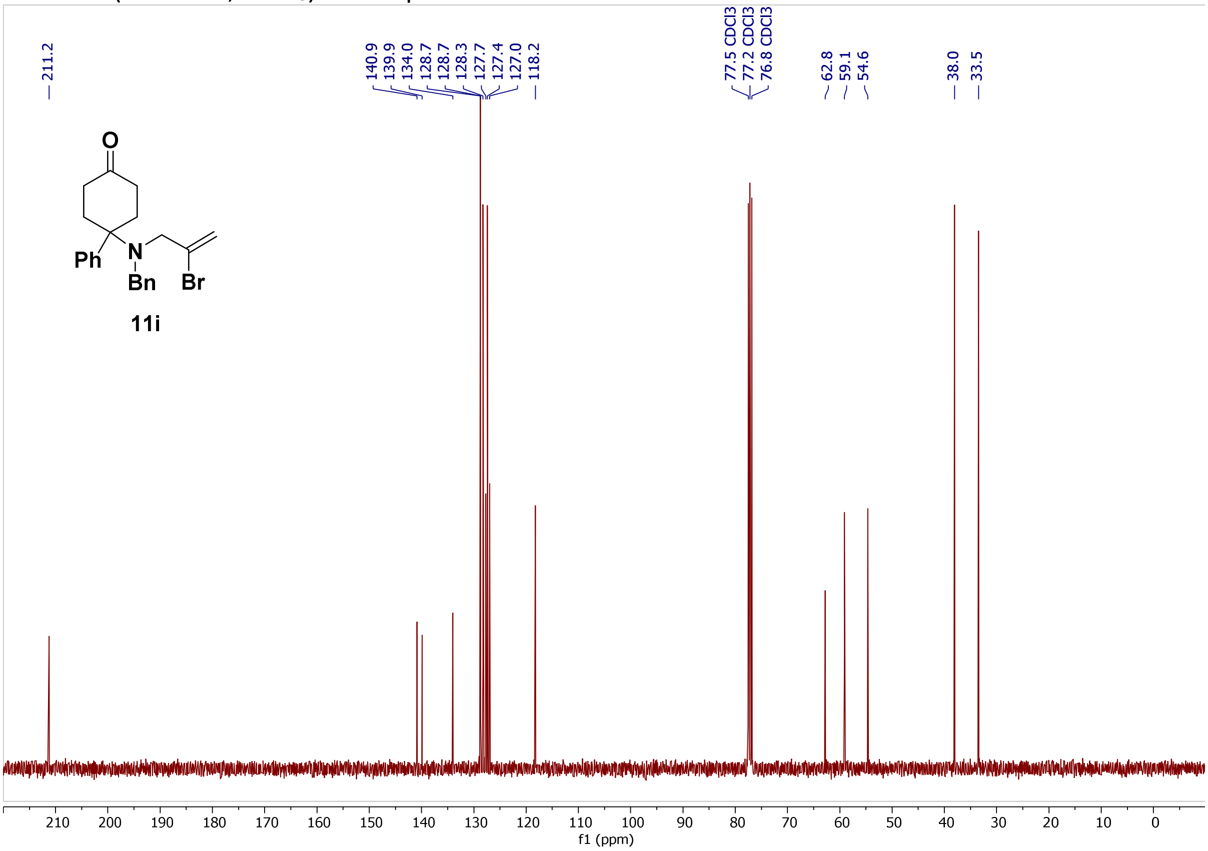

^{1}**H NMR** (400 MHz, $CDCl_3$) of compound **S10.**

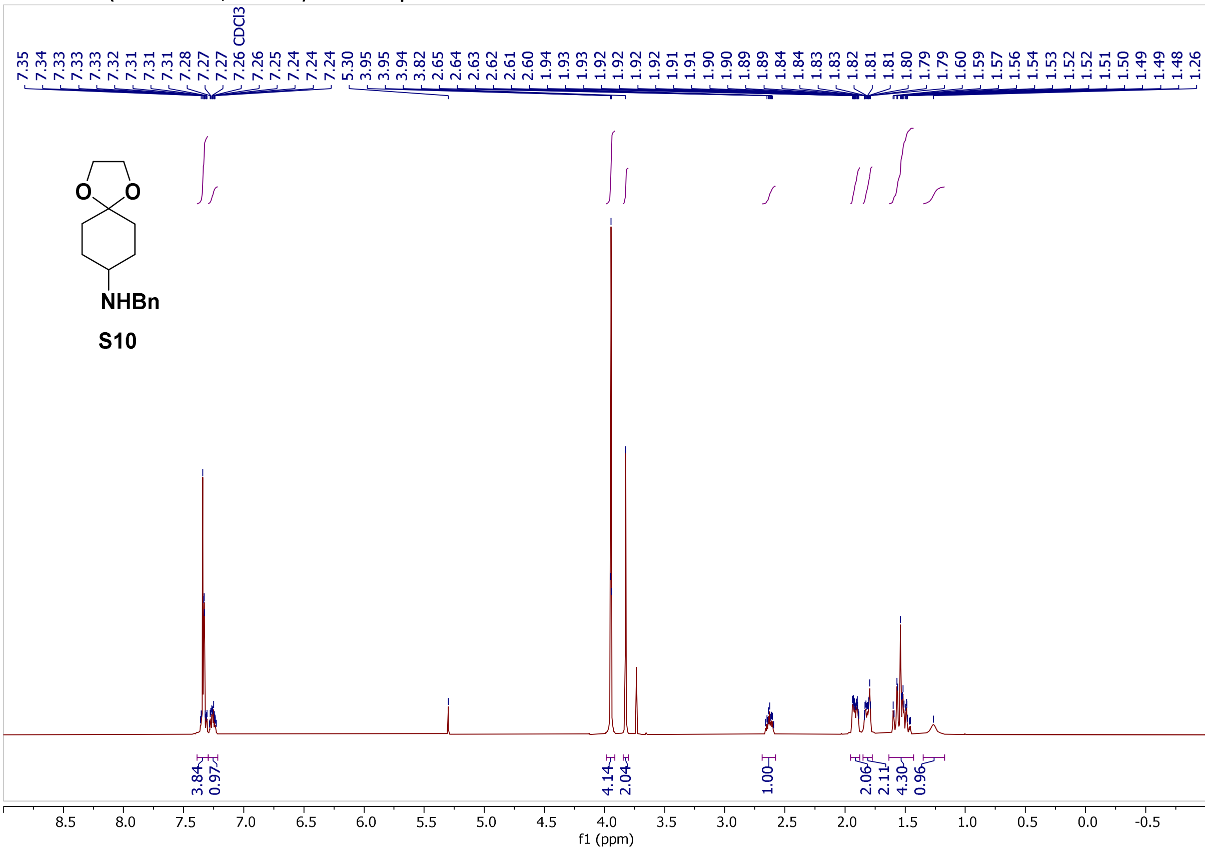

^{13}**C NMR** (101 MHz, $CDCl_3$) of compound **S10.**

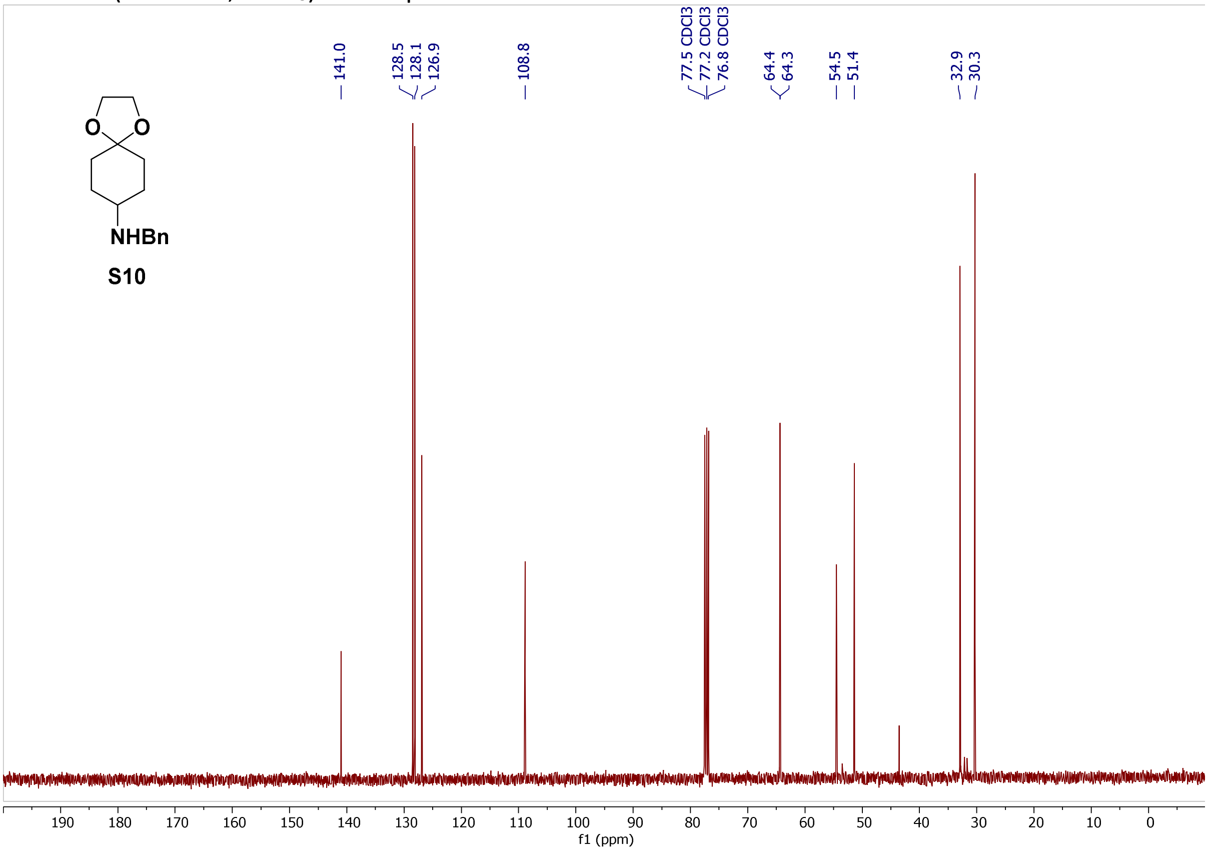

^{1}H NMR (400 MHz, $CDCl_3$) of compound **S11.**

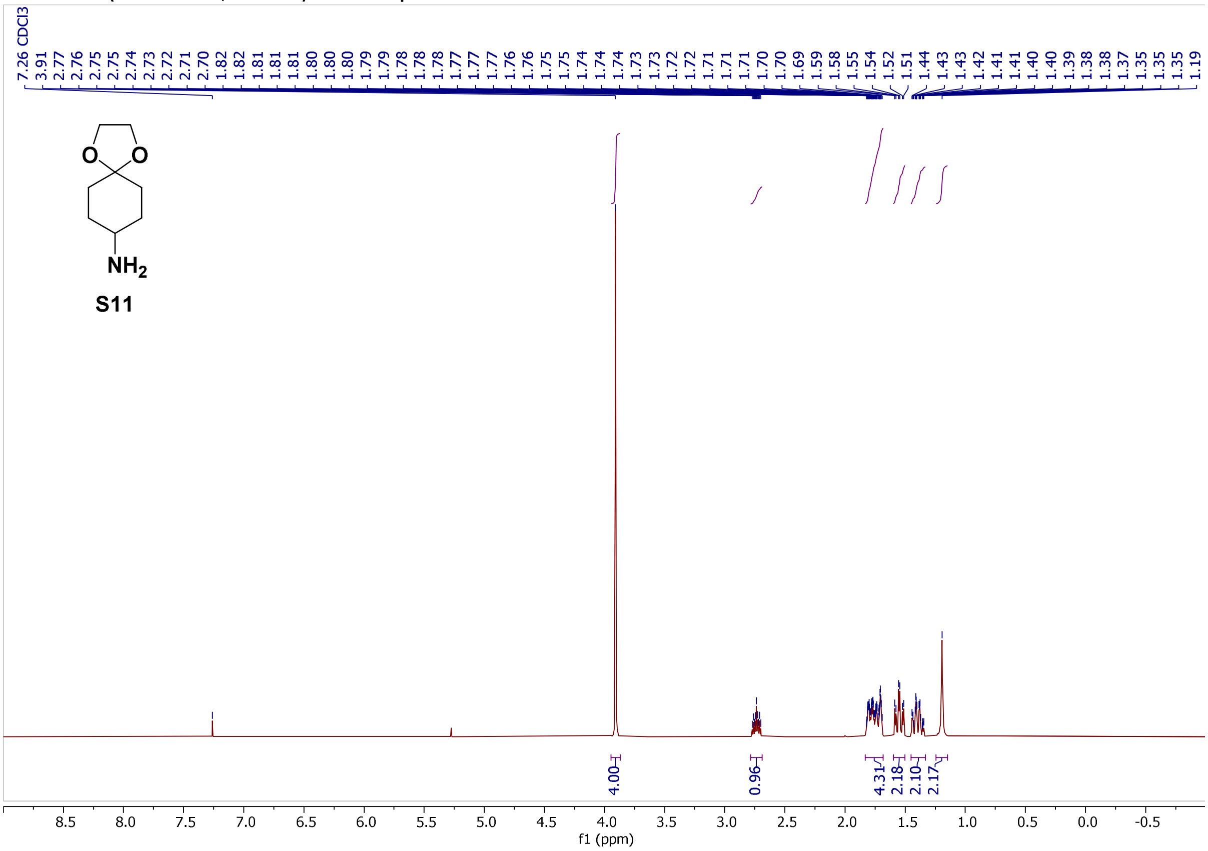

^{13}C NMR (101 MHz, $CDCl_3$) of compound **S11.**

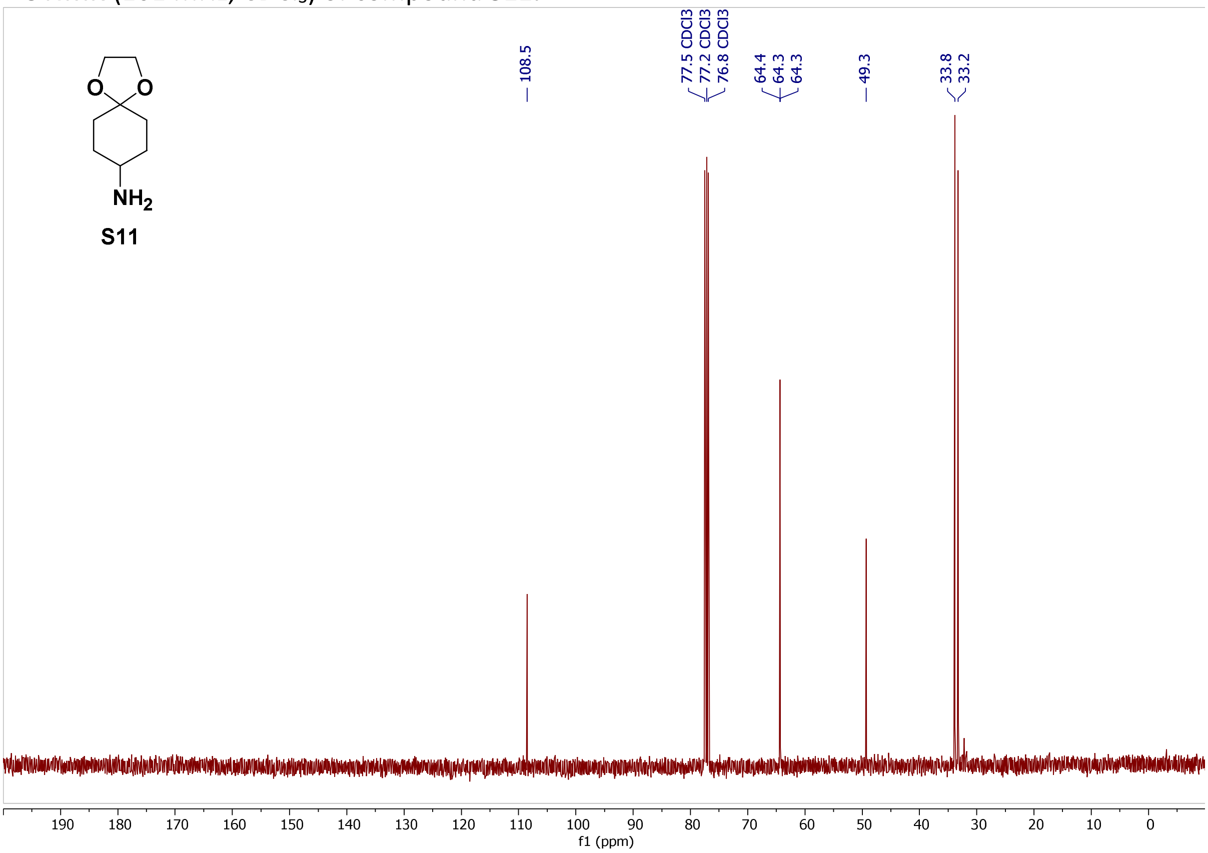

^{1}H NMR (400 MHz, $CDCl_3$) of compound **S12.**

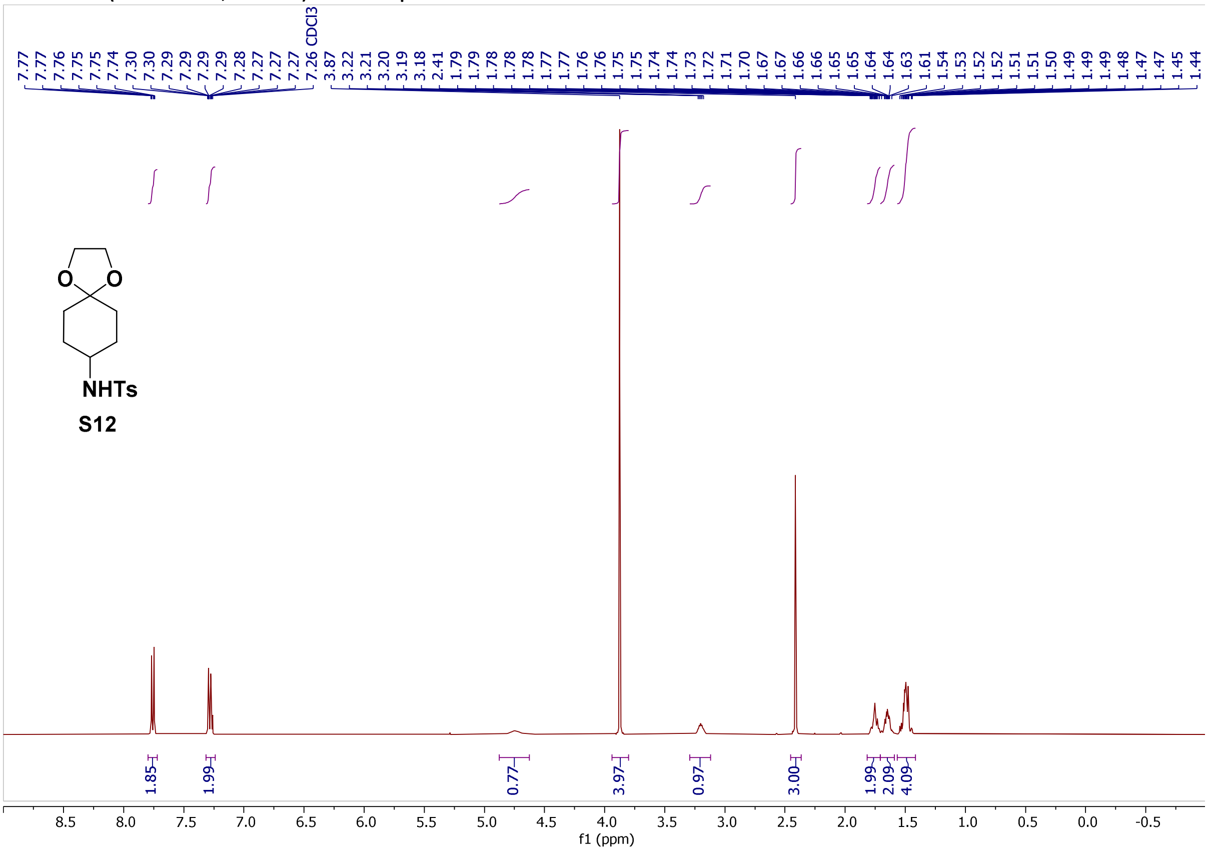

^{13}C NMR (101 MHz, $CDCl_3$) of compound **S12.**

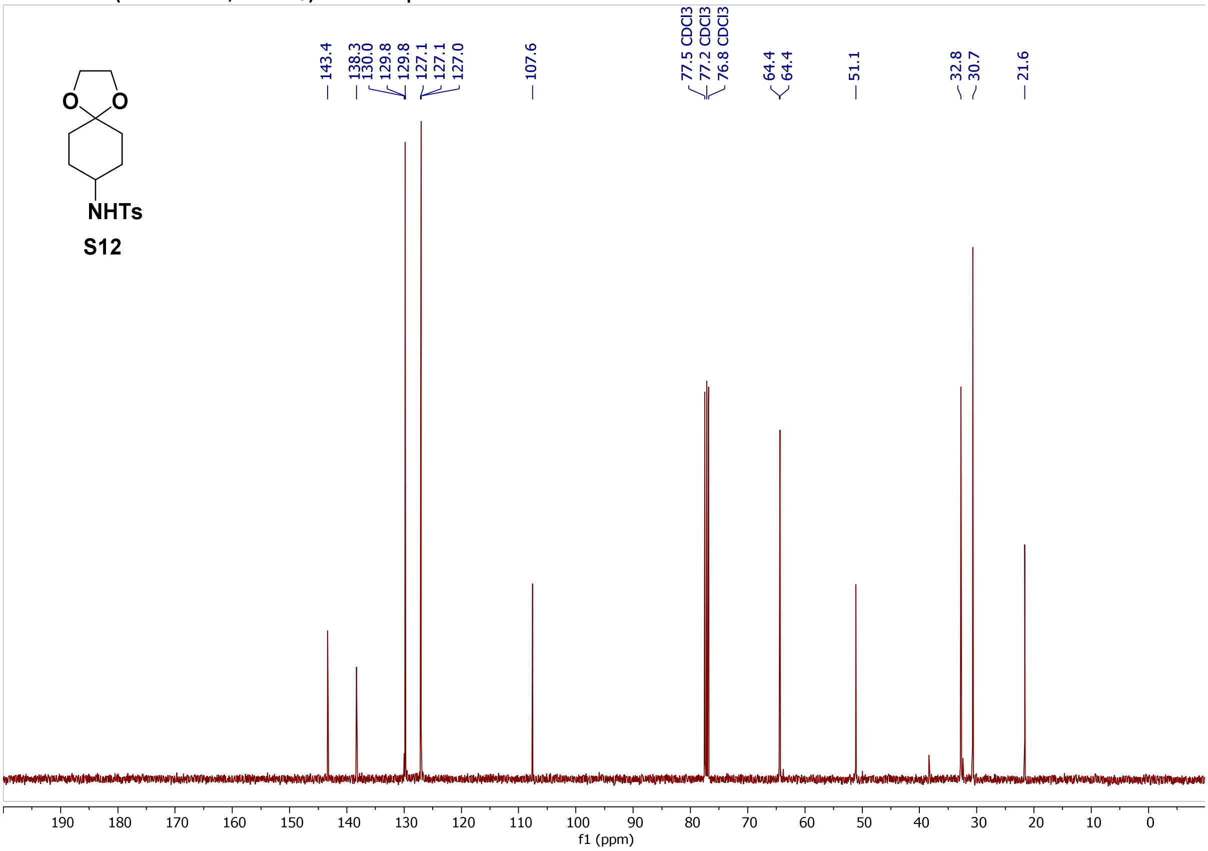

^{1}H NMR (400 MHz, $CDCl_3$) of compound **S19.**

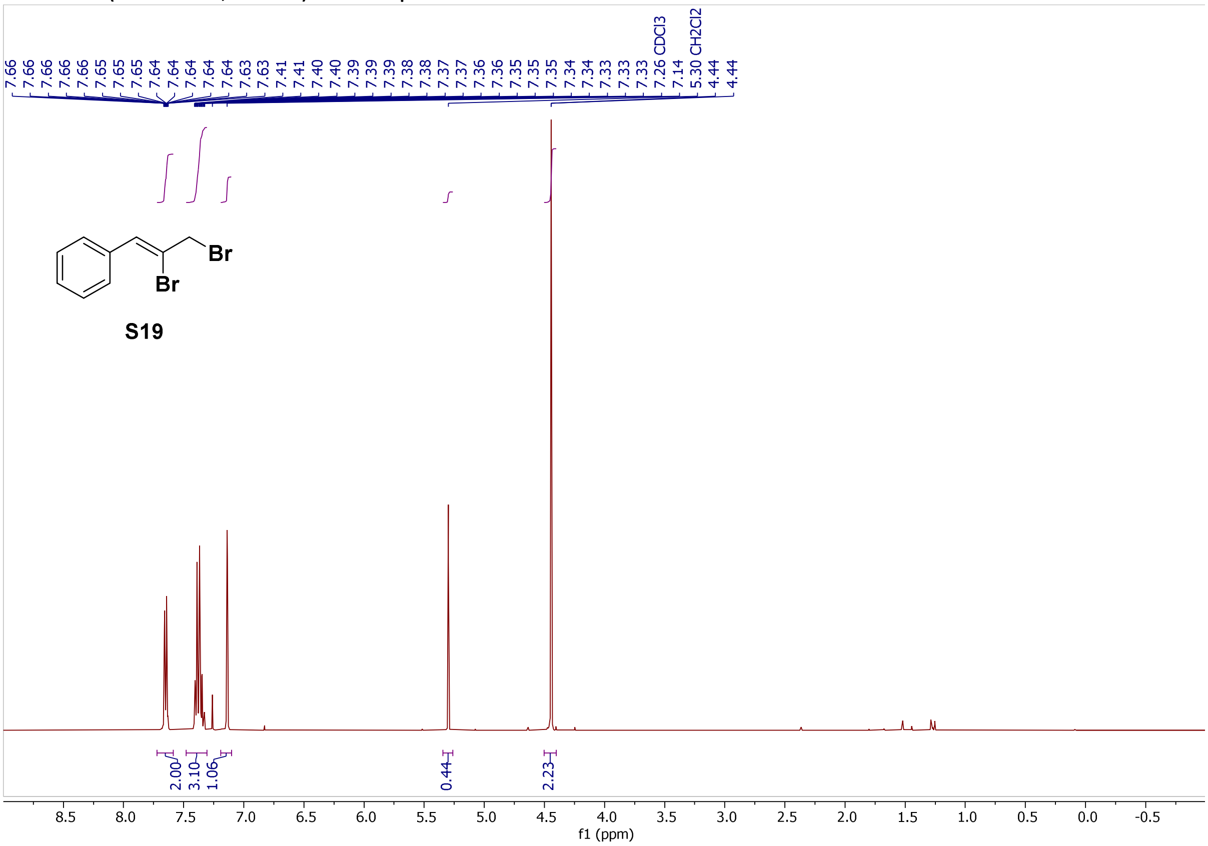

^{13}C NMR (101 MHz, $CDCl_3$) of compound **S19.**

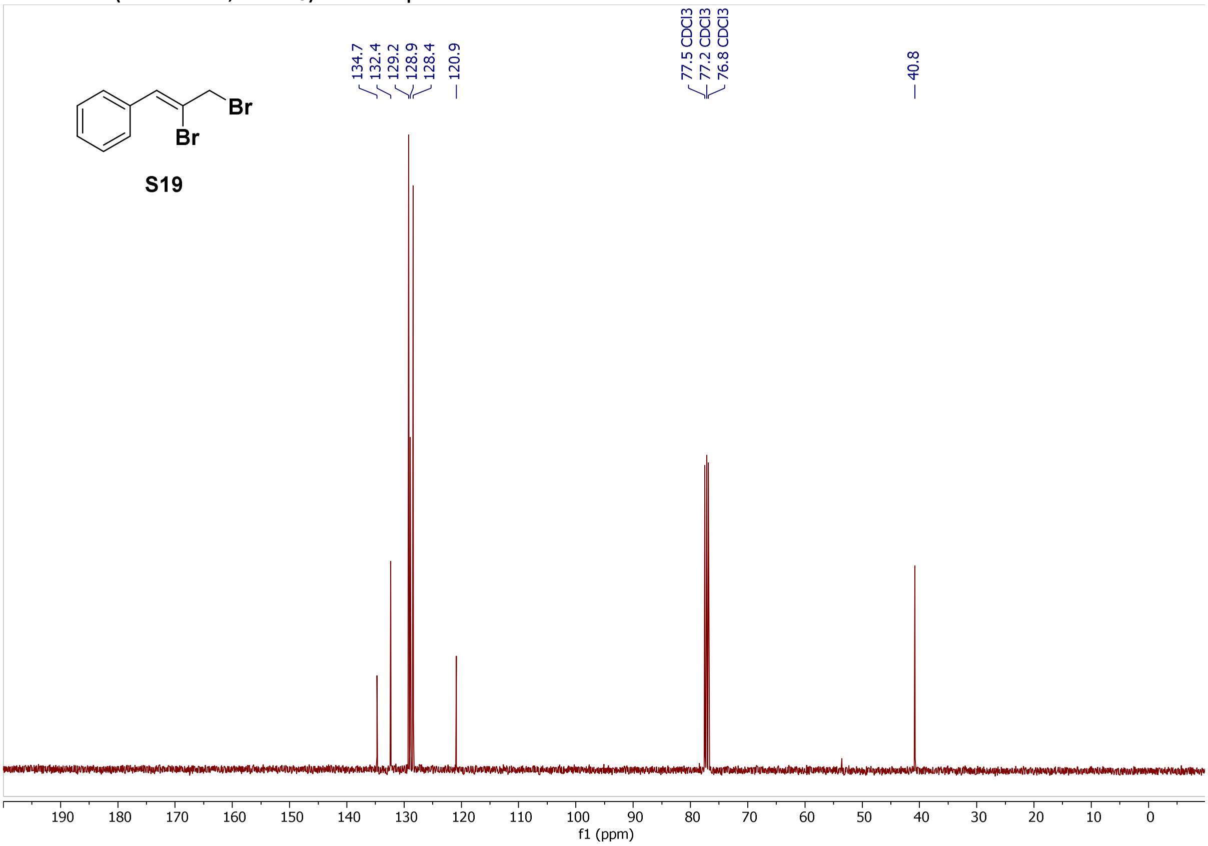

^{1}**H NMR** (400 MHz, $CDCl_3$) of compound **S20.**

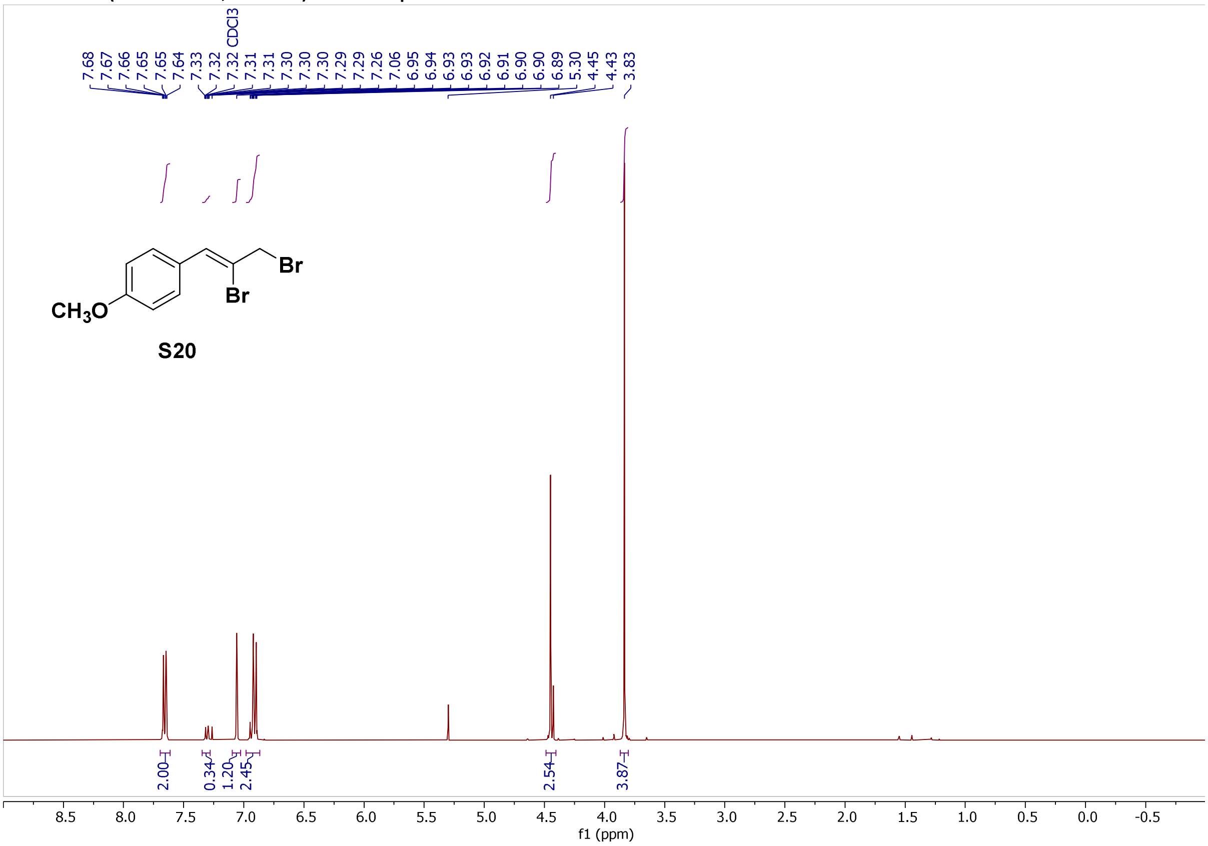

^{13}**C NMR** (101 MHz, $CDCl_3$) of compound **S20.**

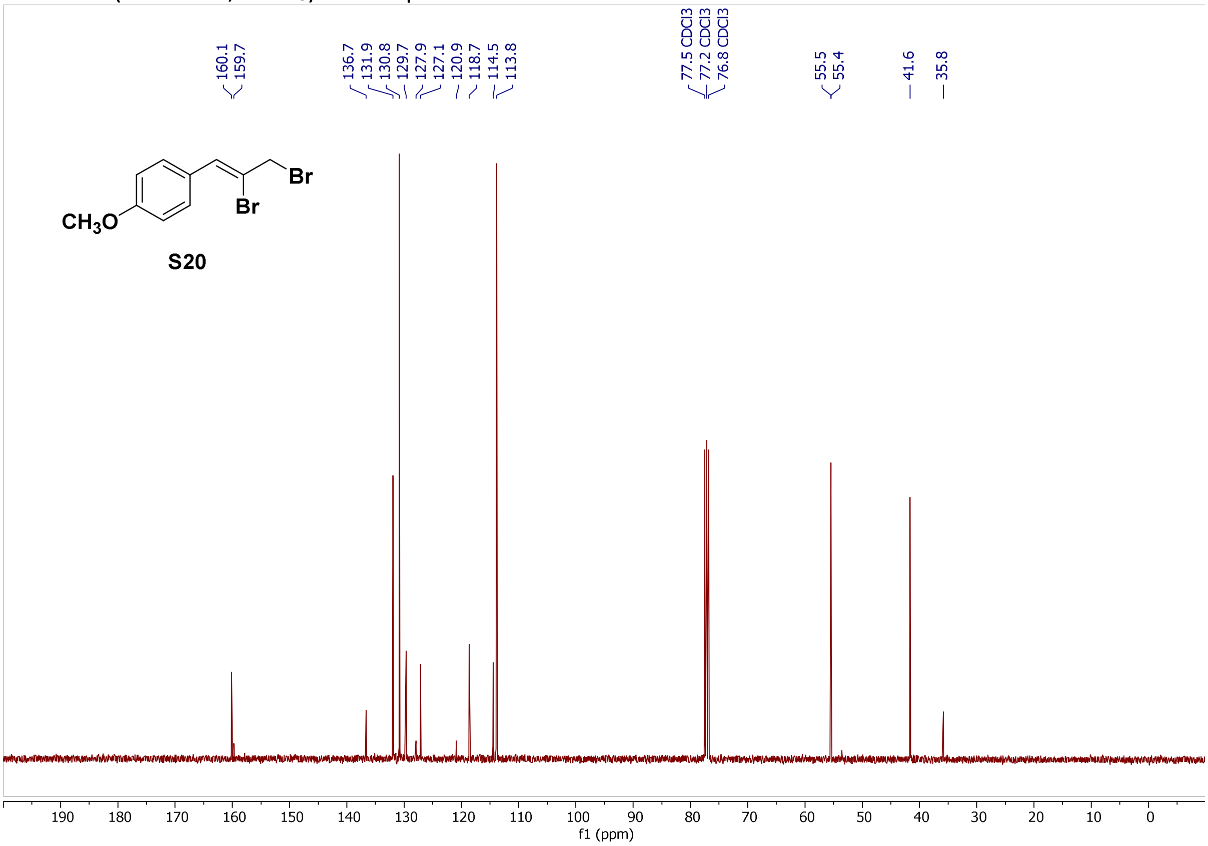

^{1}**H NMR** (400 MHz, $CDCl_3$) of compound **S22.**

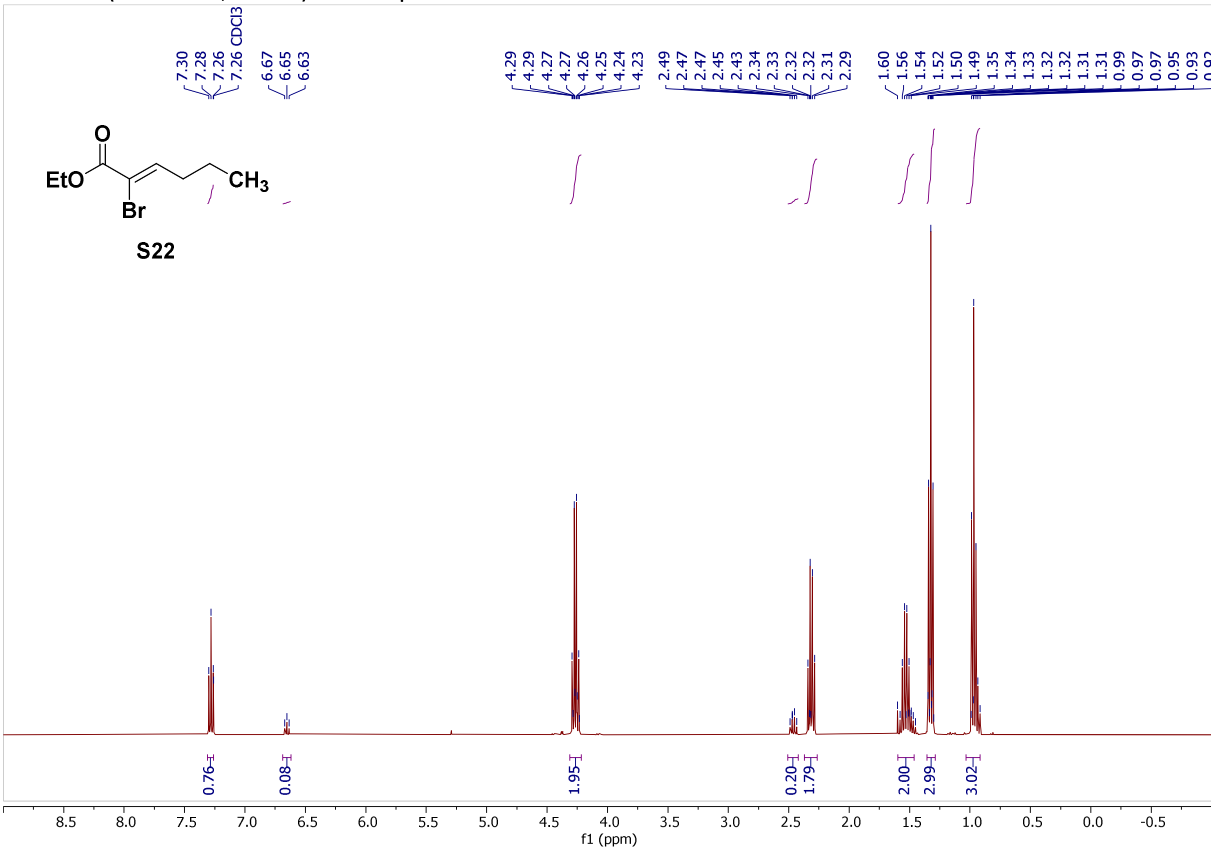

^{13}**C NMR** (101 MHz, $CDCl_3$) of compound **S22.**

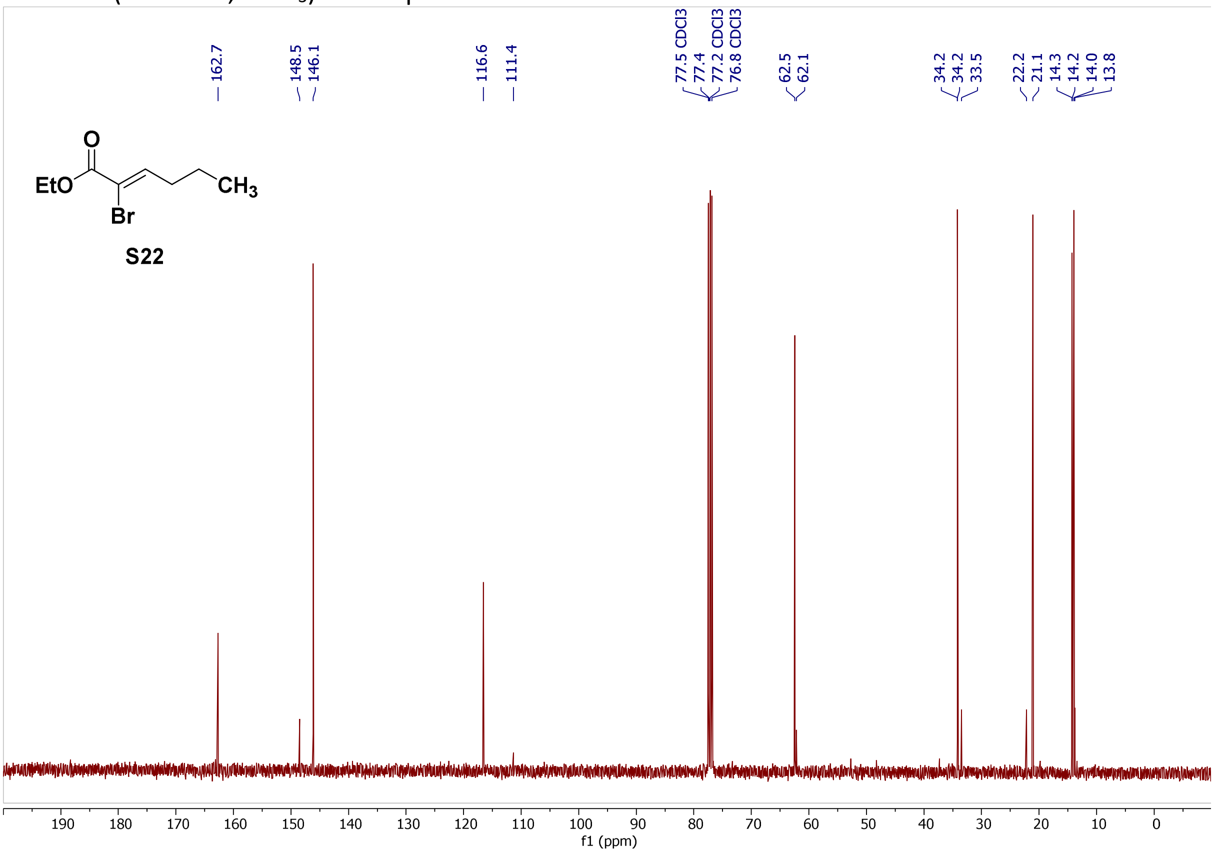

[1]**H NMR** (400 MHz, $CDCl_3$) of compound **S24.**

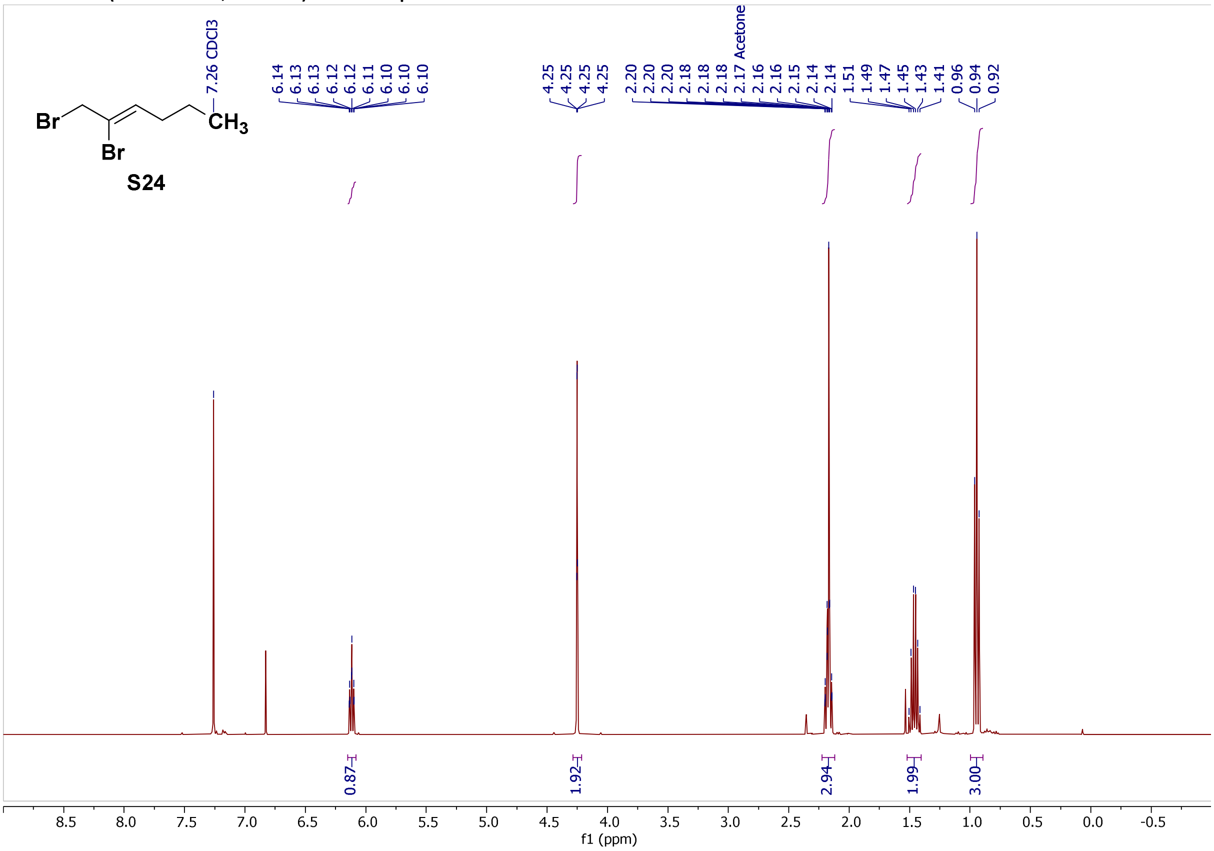

[13]**C NMR** (101 MHz, $CDCl_3$) of compound **S24.**

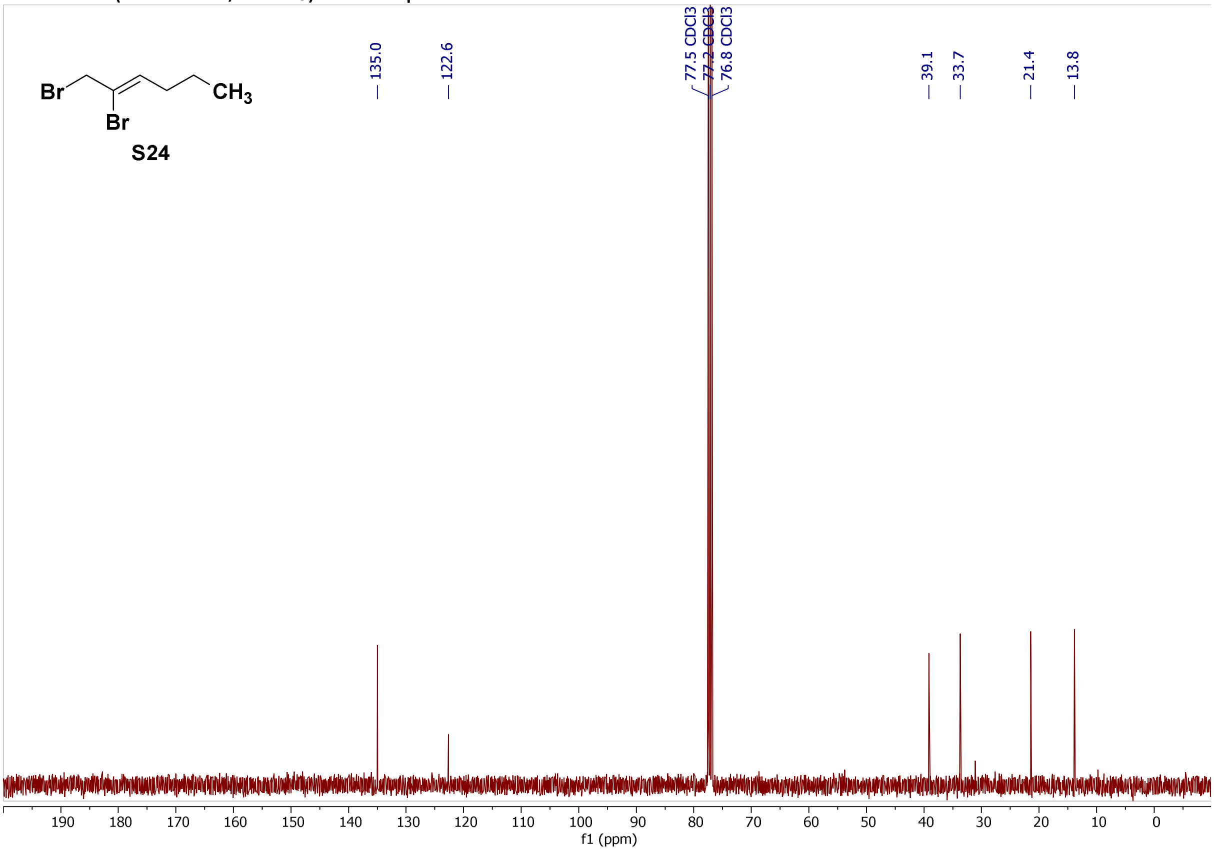

^{1}H NMR (400 MHz, $CDCl_3$) of compound **S25.**

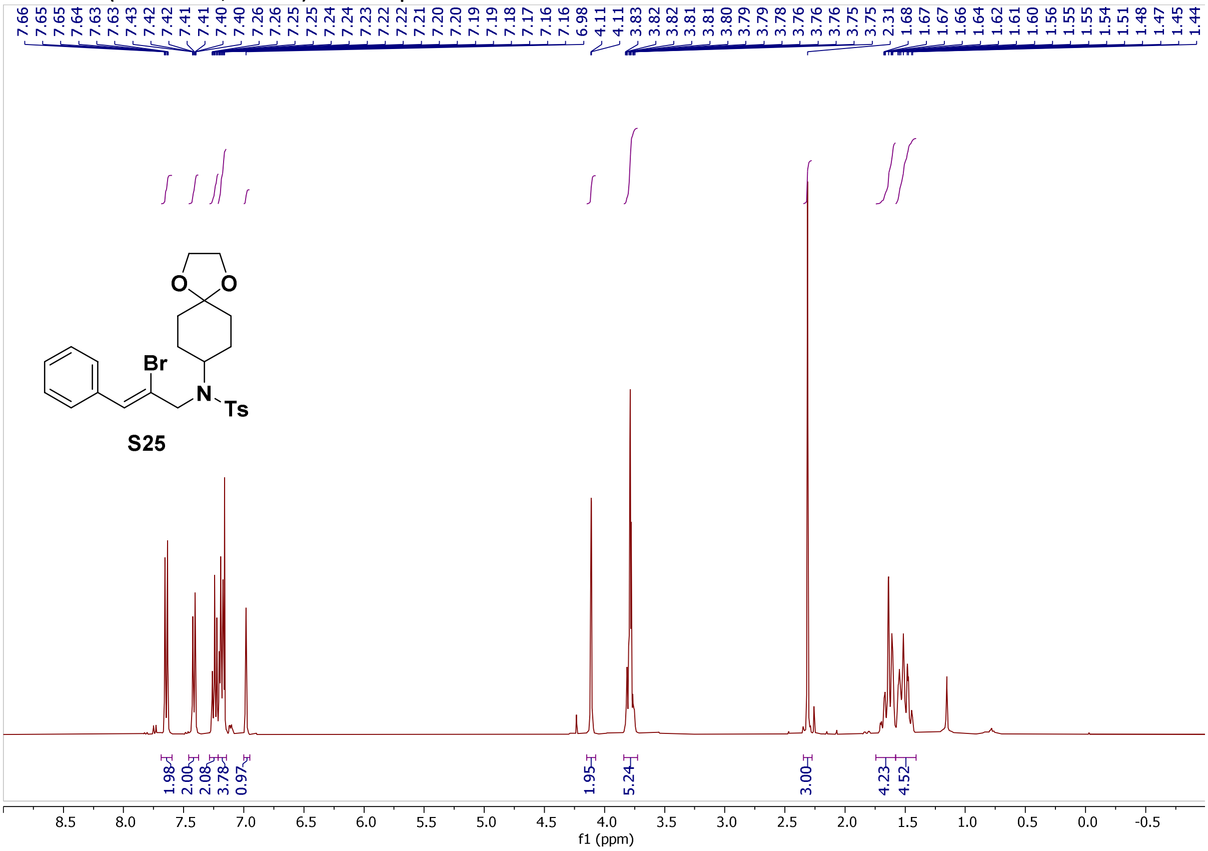

^{13}C NMR (101 MHz, $CDCl_3$) of compound **S25.**

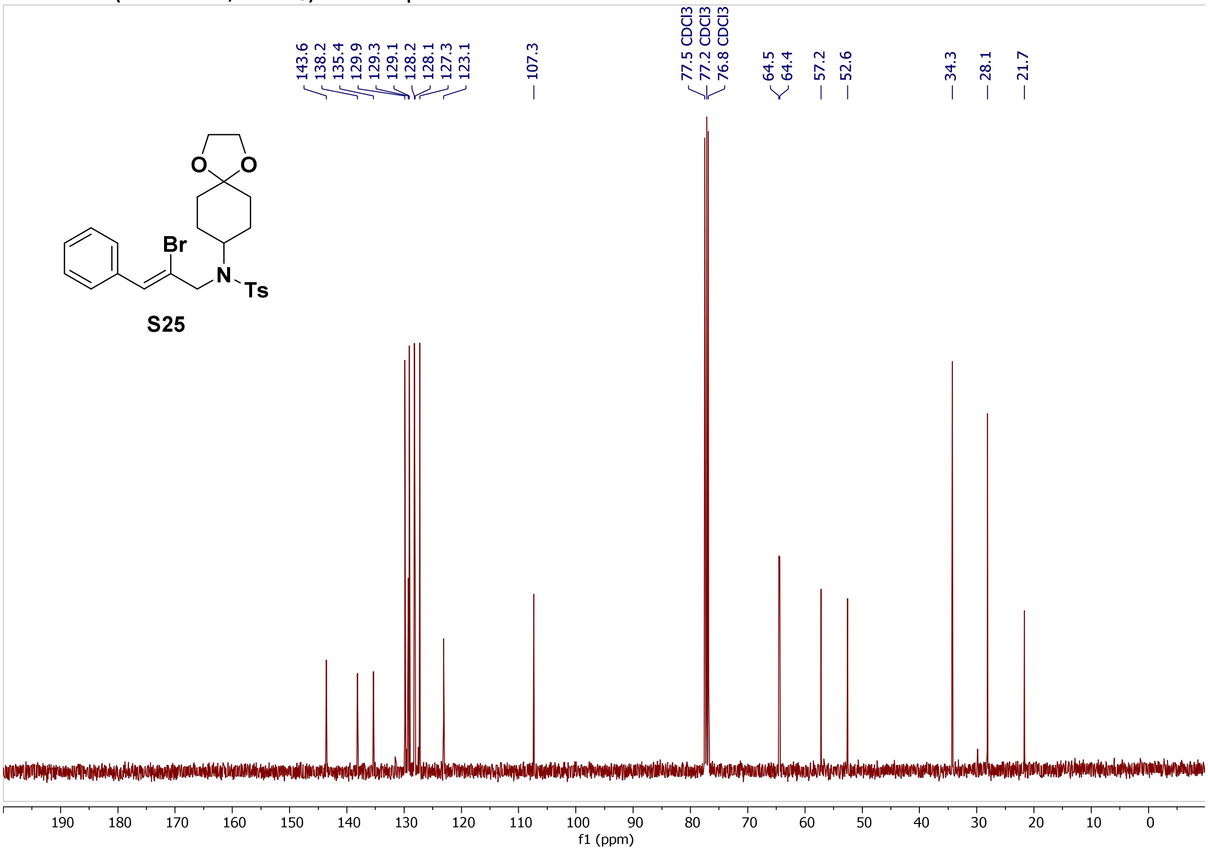

^{1}H NMR (400 MHz, $CDCl_3$) of compound **11j.**

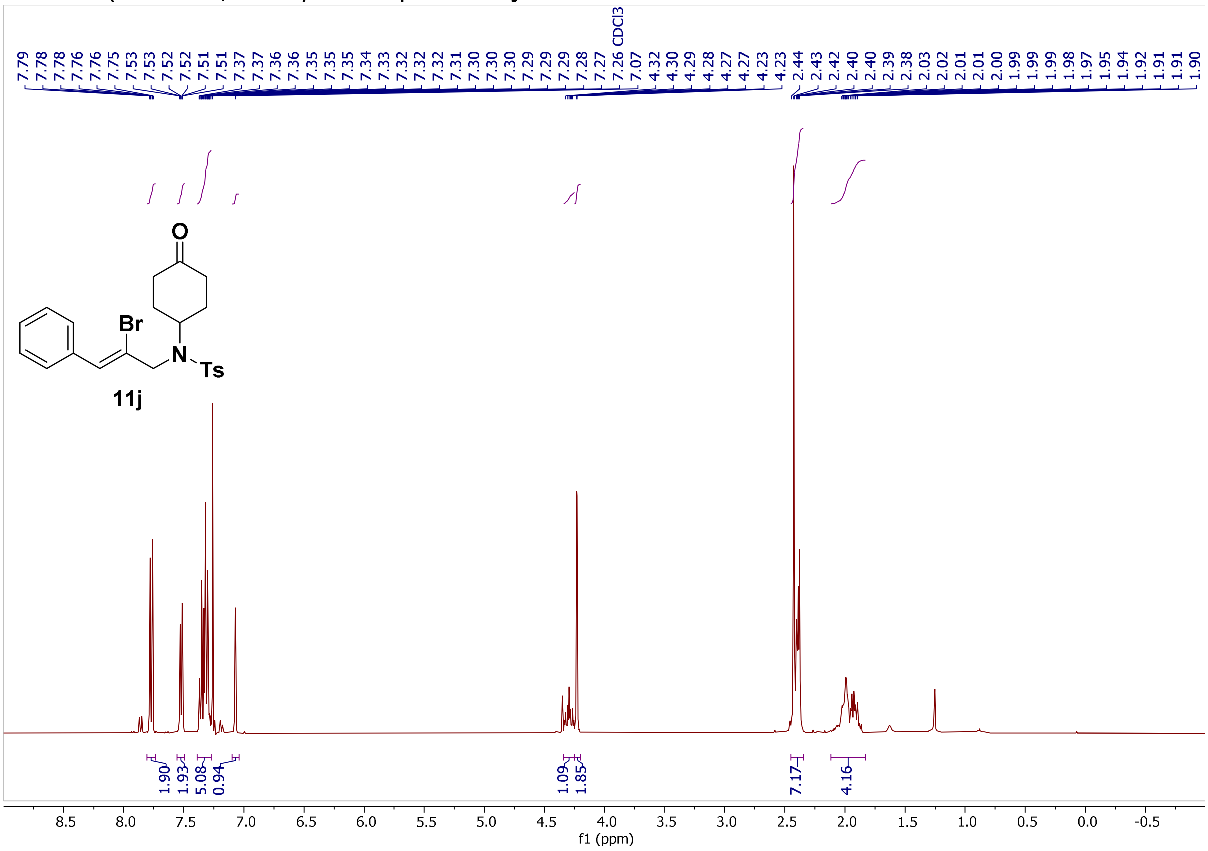

^{13}C NMR (101 MHz, $CDCl_3$) of compound **11j.**

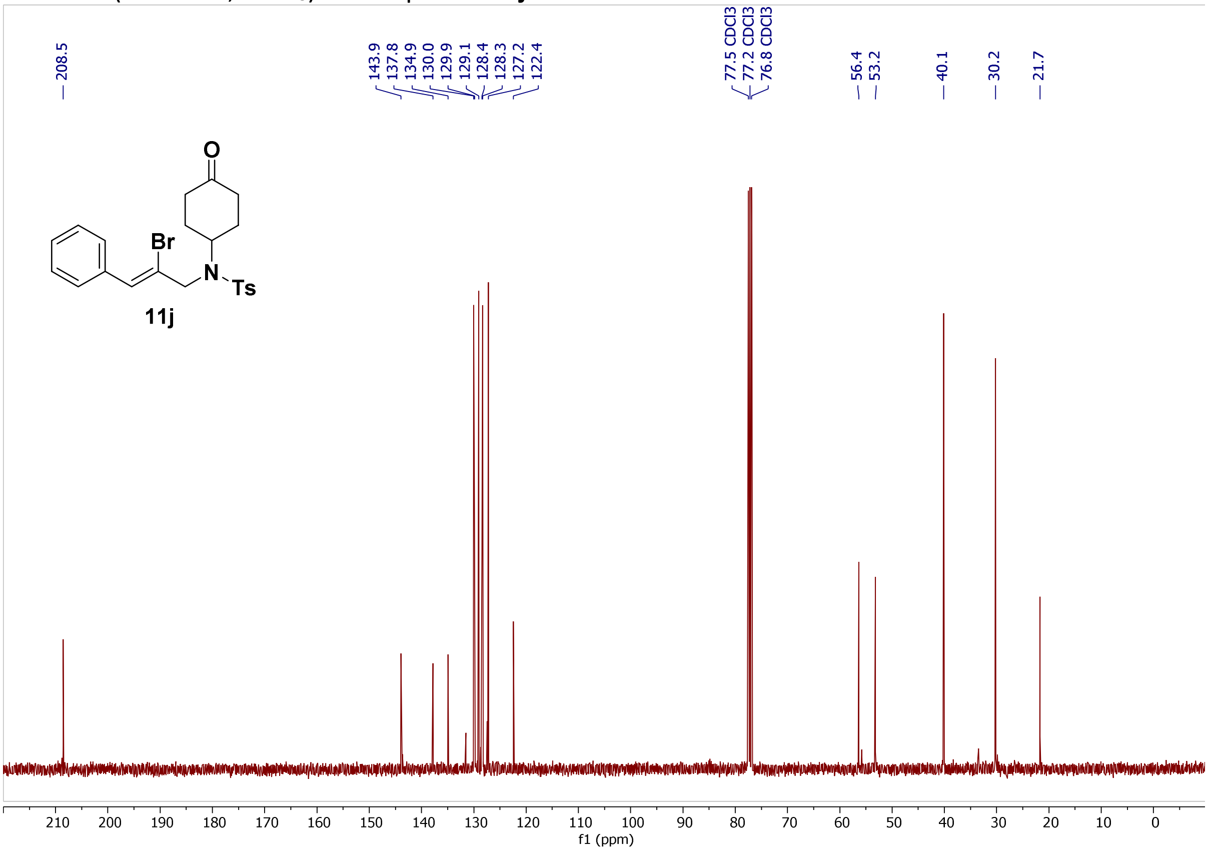

^{1}H NMR (400 MHz, $CDCl_3$) of compound **S26.**

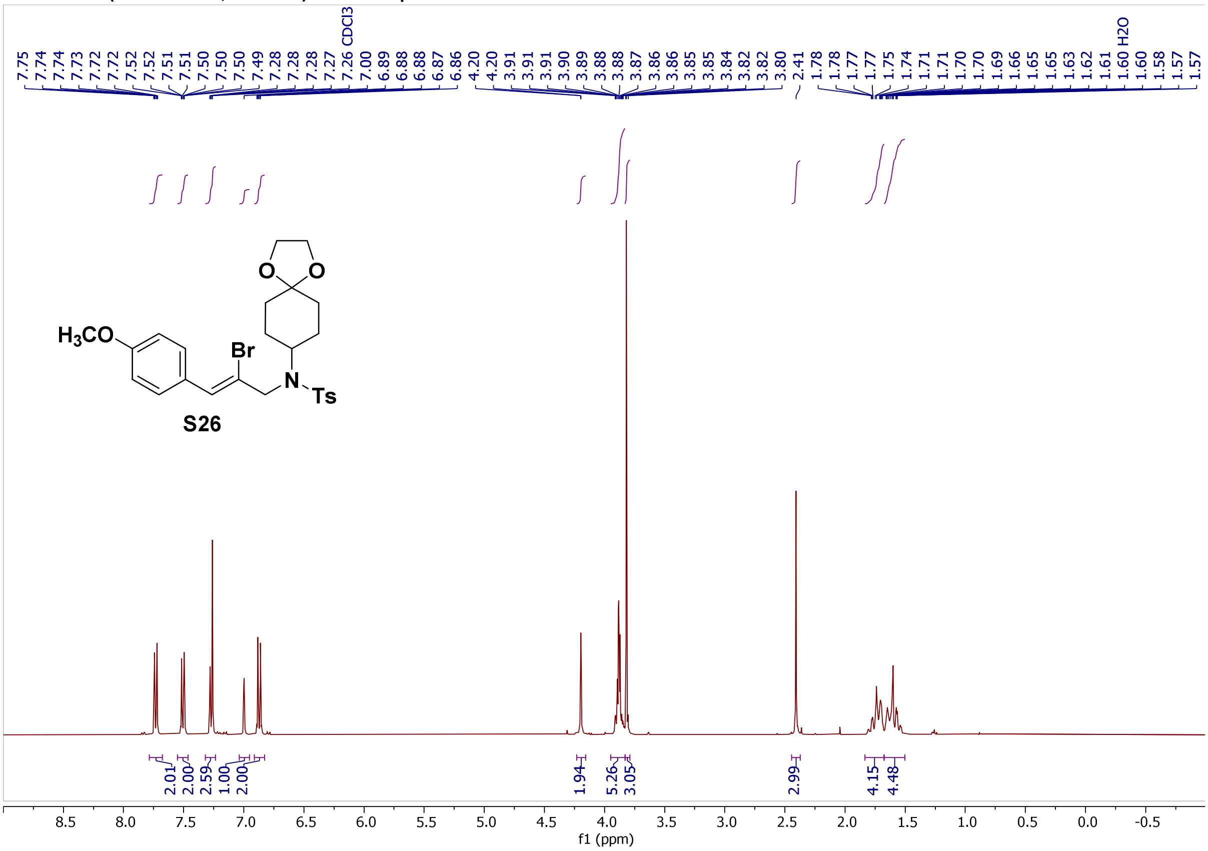

^{13}C NMR (101 MHz, $CDCl_3$) of compound **S26.**

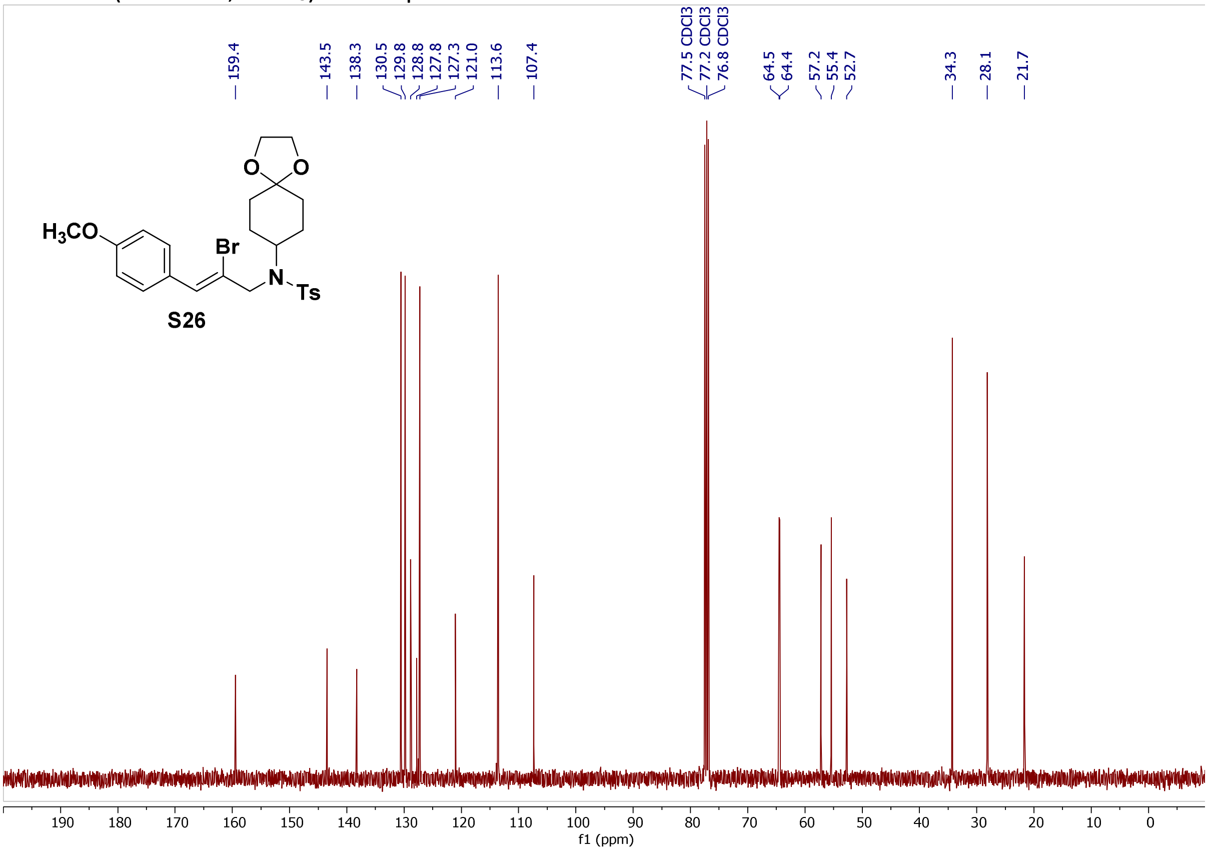

^{1}**H NMR** (400 MHz, $CDCl_3$) of compound **11k.**

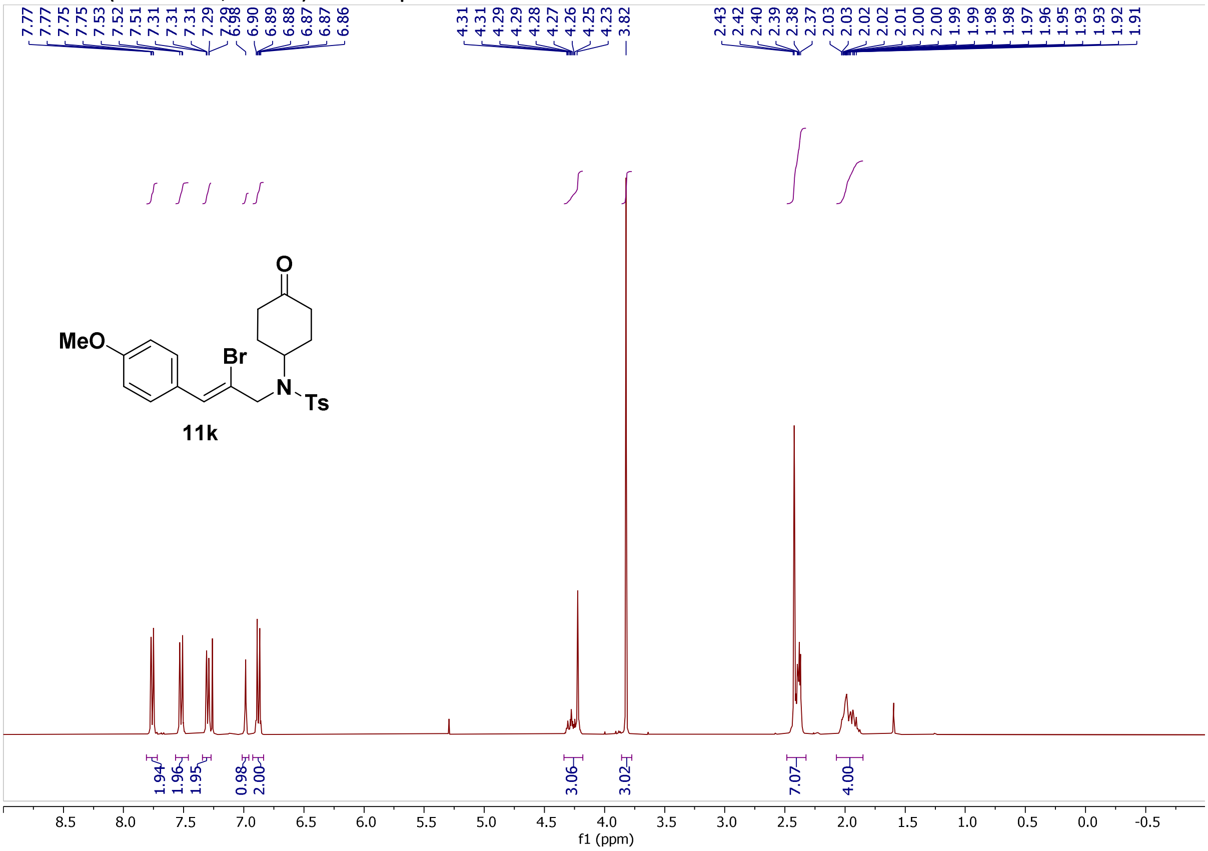

^{13}**C NMR** (101 MHz, $CDCl_3$) of compound **11k.**

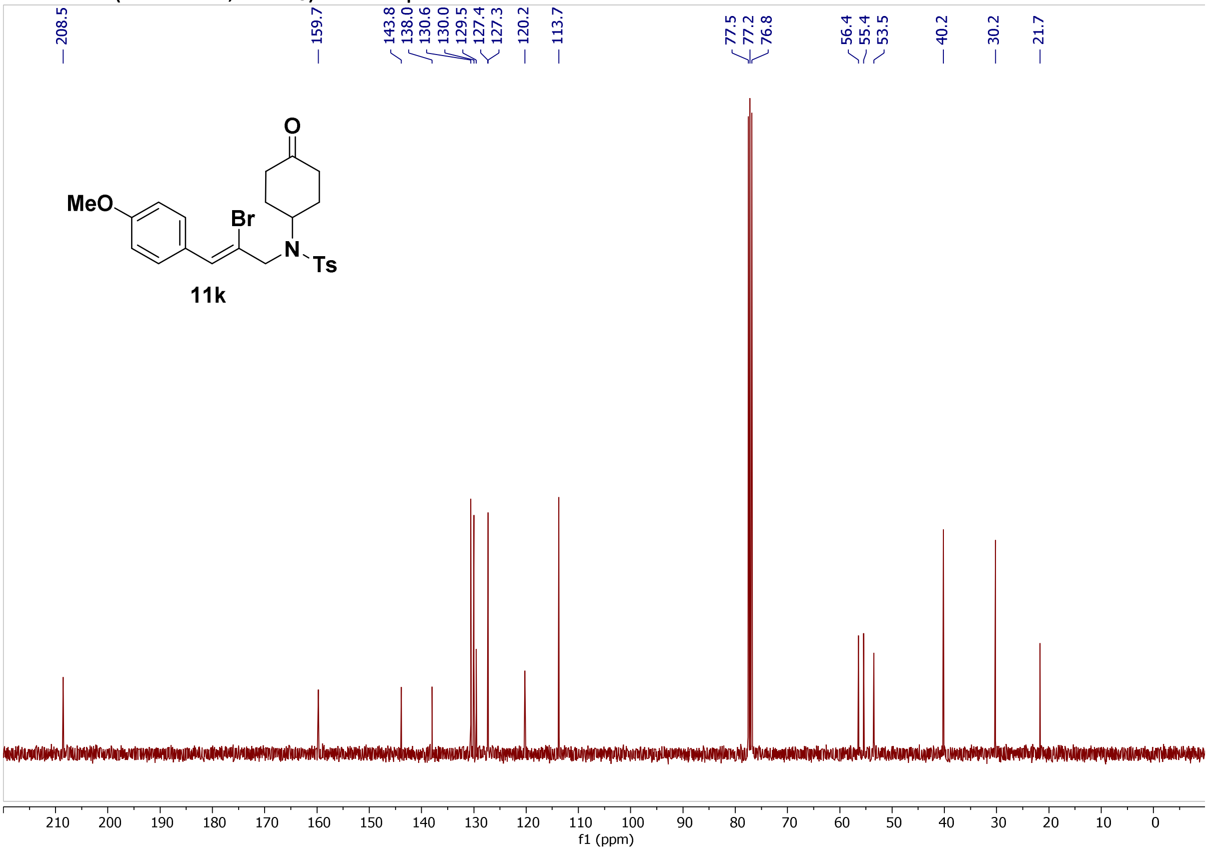

^{1}**H NMR** (400 MHz, $CDCl_3$) of compound **S27.**

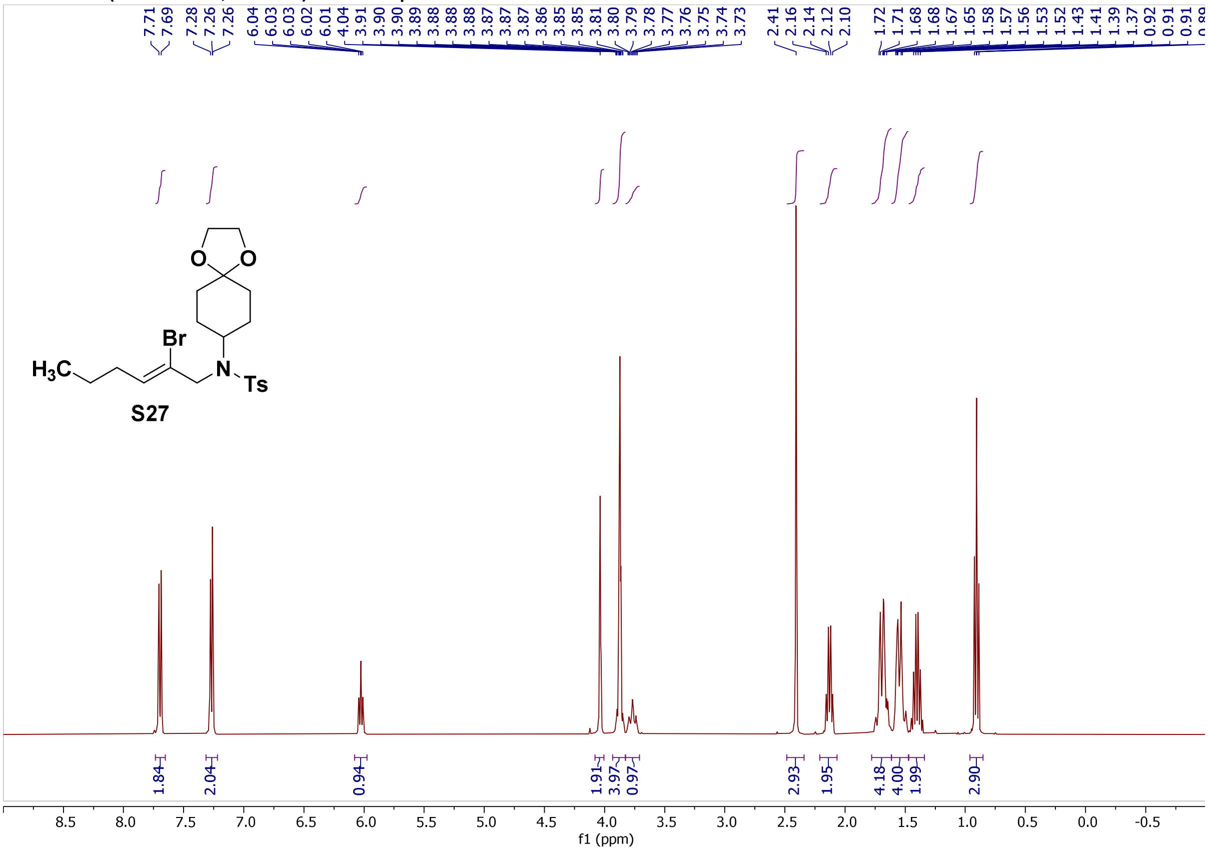

^{13}**C NMR** (101 MHz, $CDCl_3$) of compound **S27.**

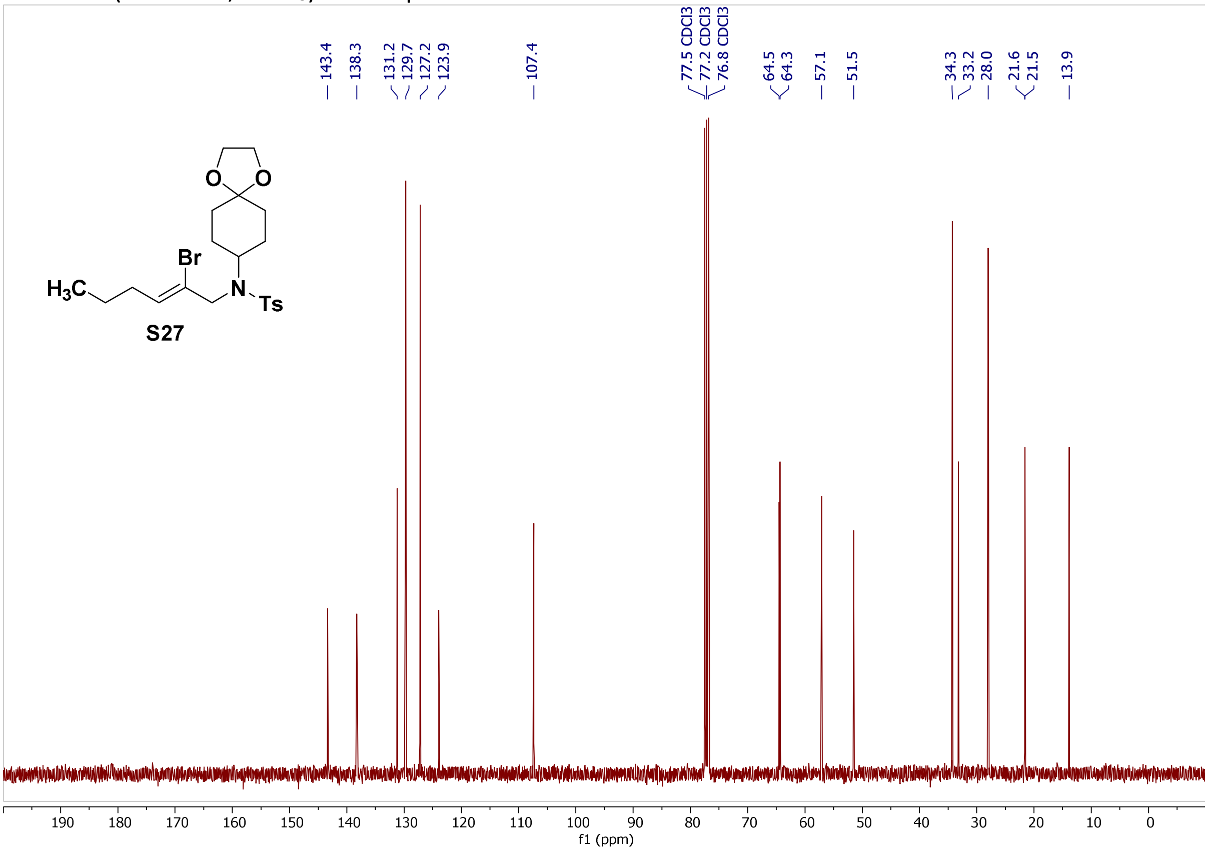

^{1}H NMR (400 MHz, $CDCl_3$) of compound **11l.**

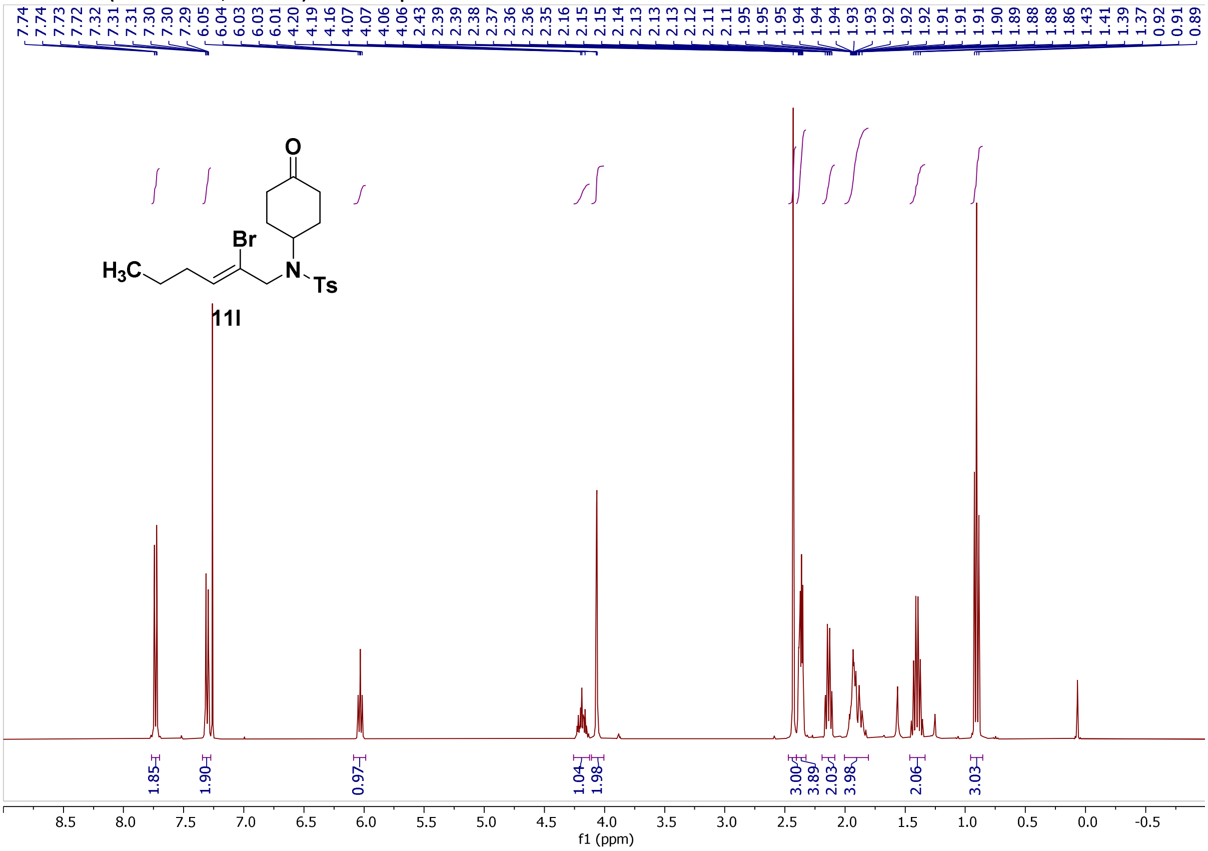

^{13}C NMR (101 MHz, $CDCl_3$) of compound **11l.**

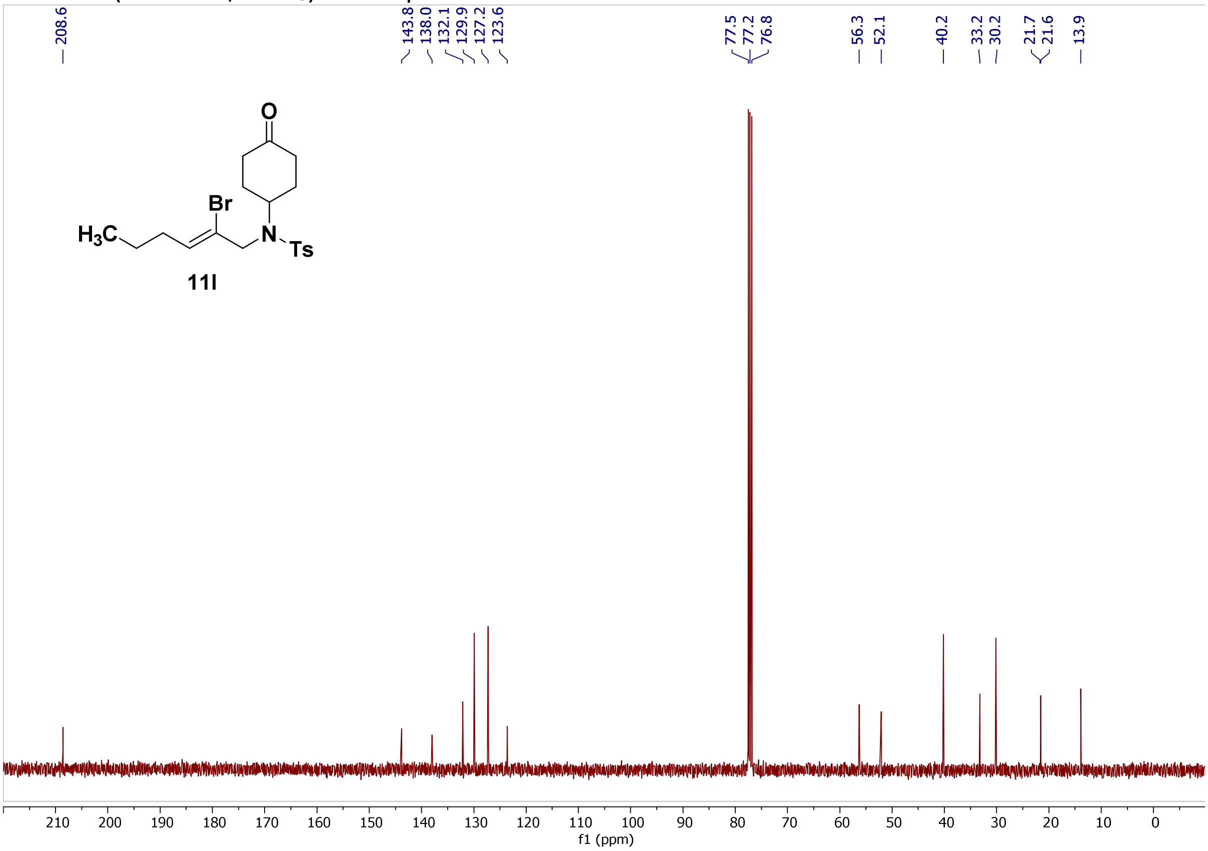

^{1}H NMR (400 MHz, $CDCl_3$) of compound **5a.**

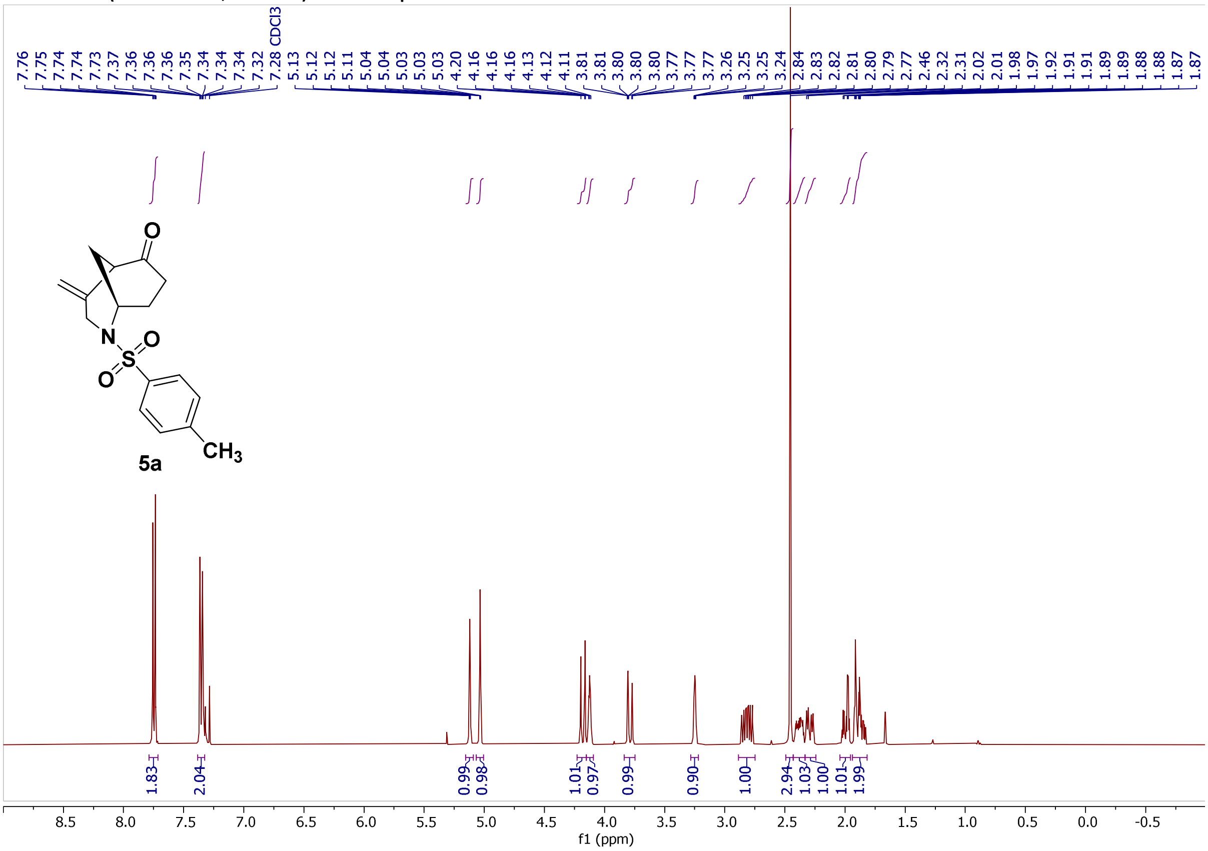

^{13}C NMR (101 MHz, $CDCl_3$) of compound **5a.**

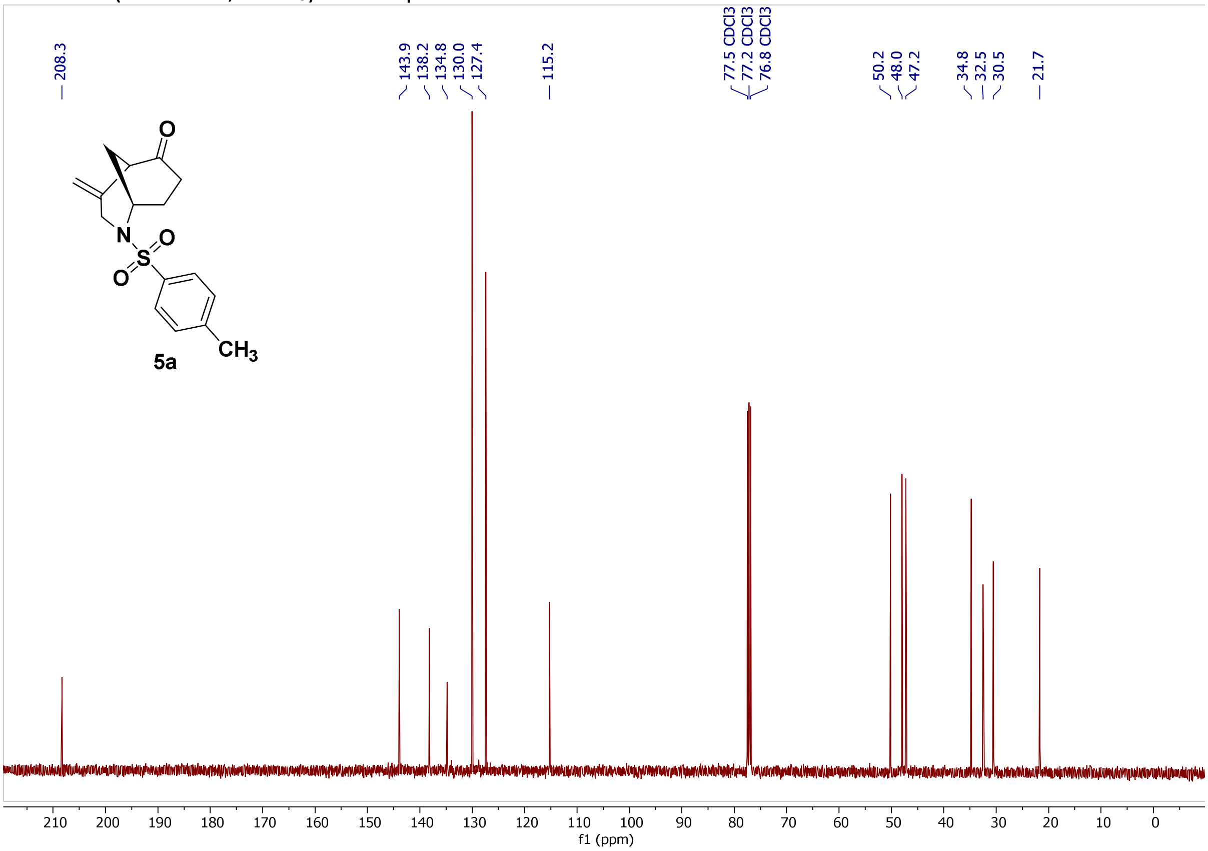

^{1}H NMR (400 MHz, $CDCl_3$) of compound **5b.**

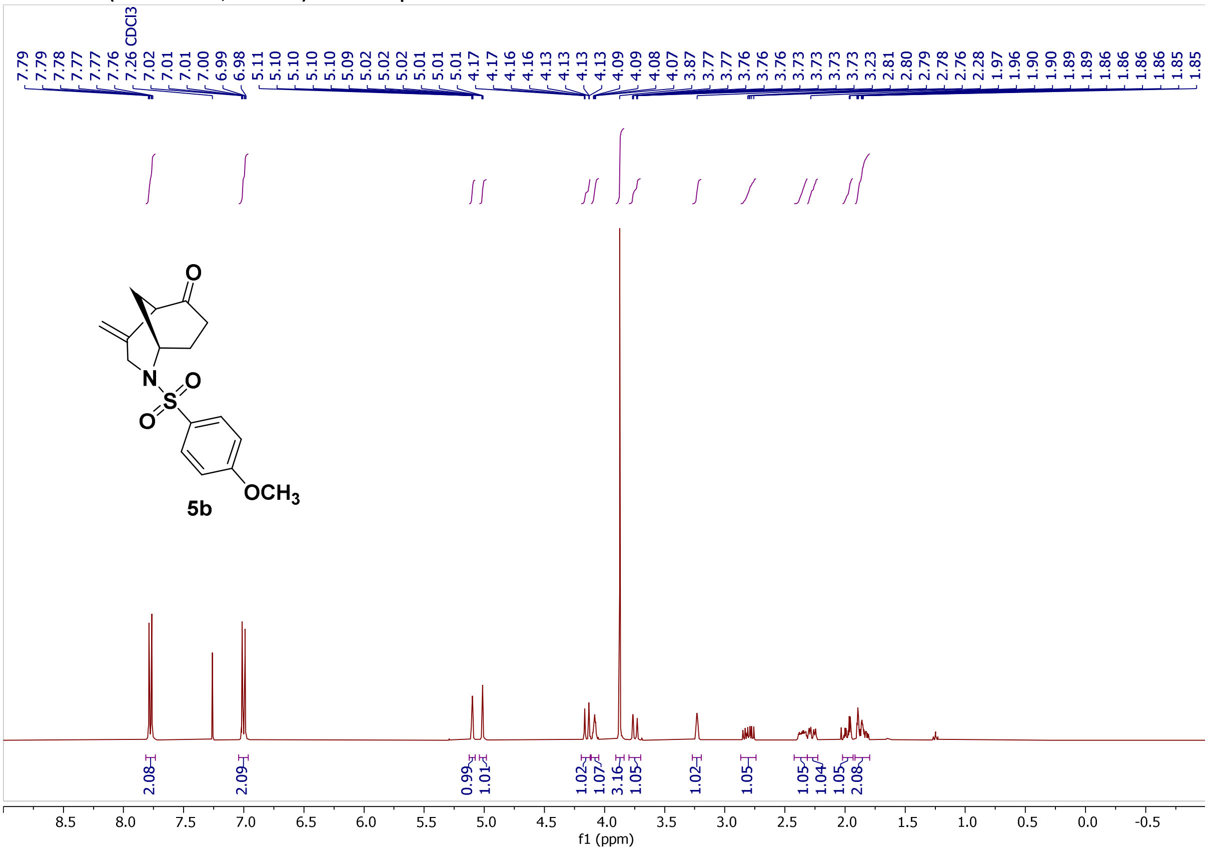

^{13}C NMR (101 MHz, $CDCl_3$) of compound **5b.**

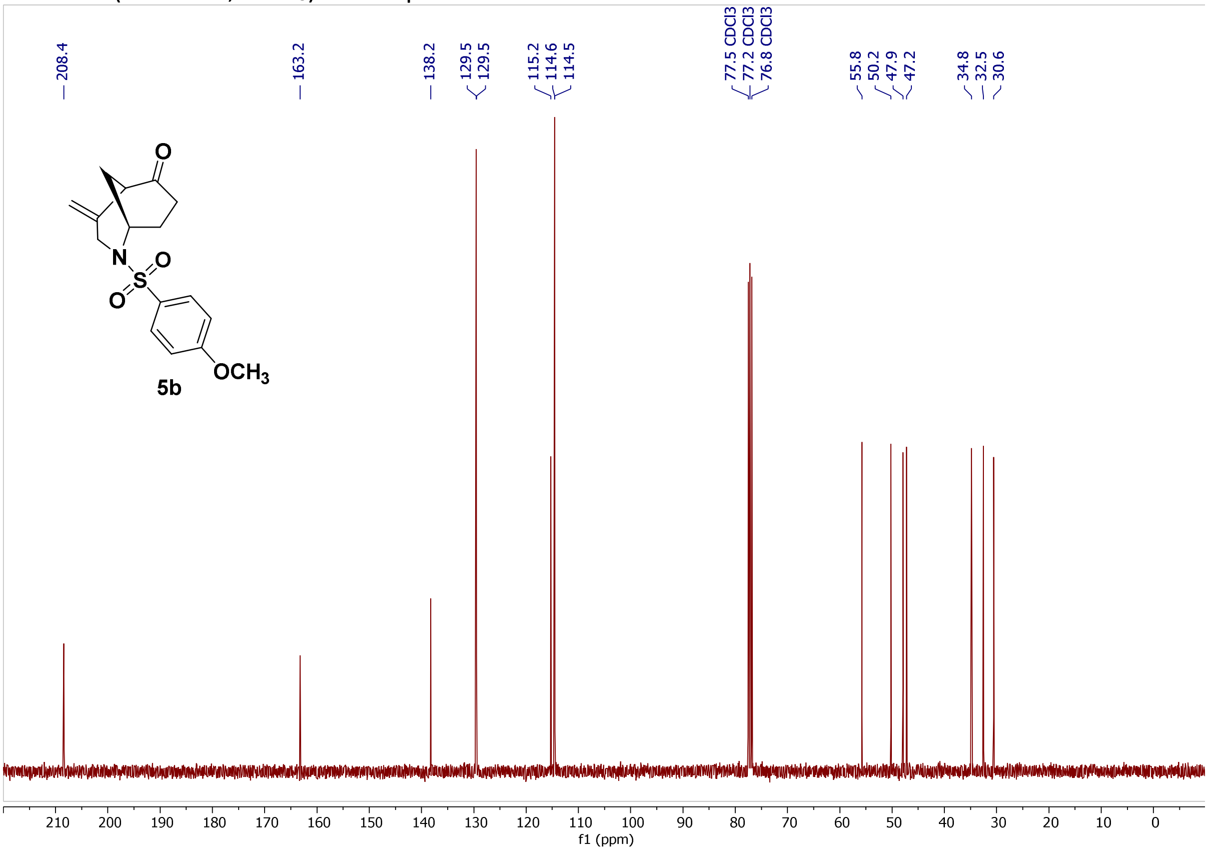

^{1}H NMR (400 MHz, $CDCl_3$) of compound **5c.**

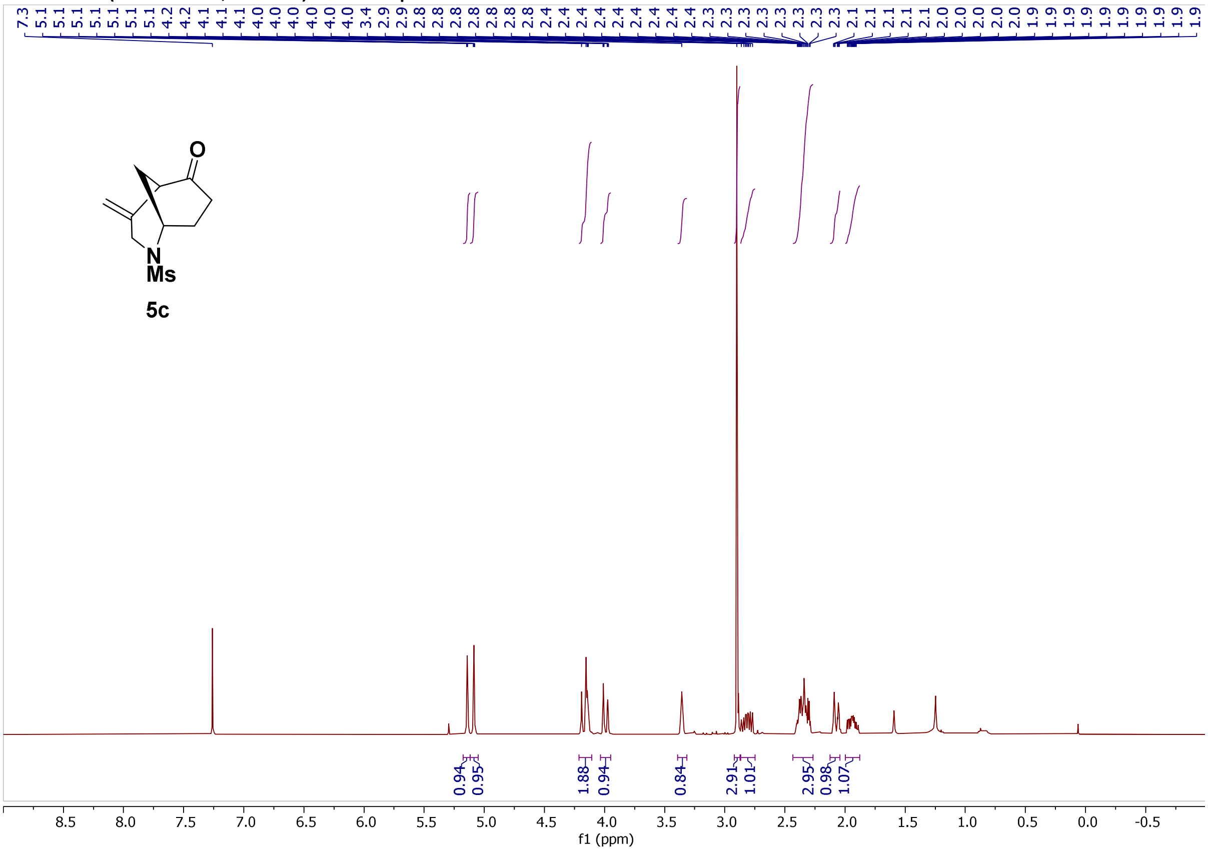

^{13}C NMR (101 MHz, $CDCl_3$) of compound **5c.**

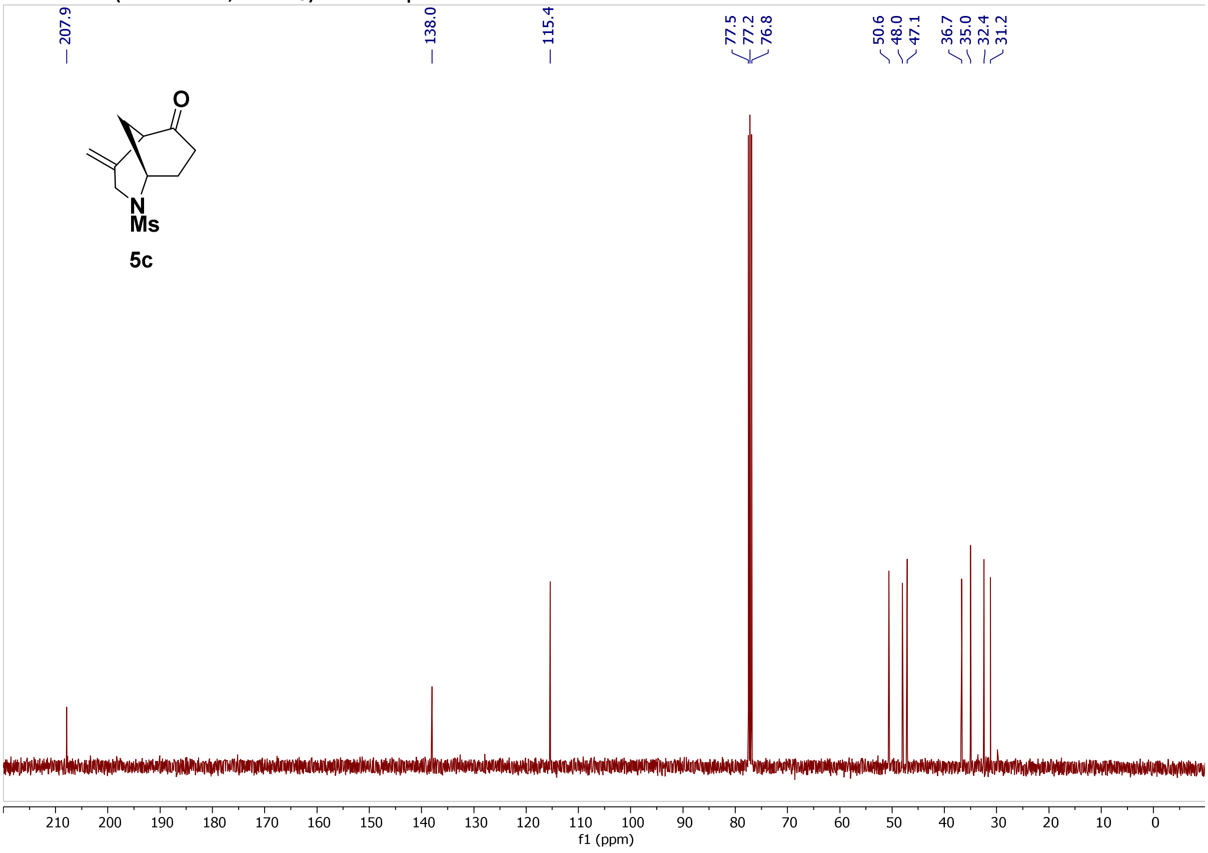

^{1}**H NMR** (400 MHz, $CDCl_3$) of compound **5d.**

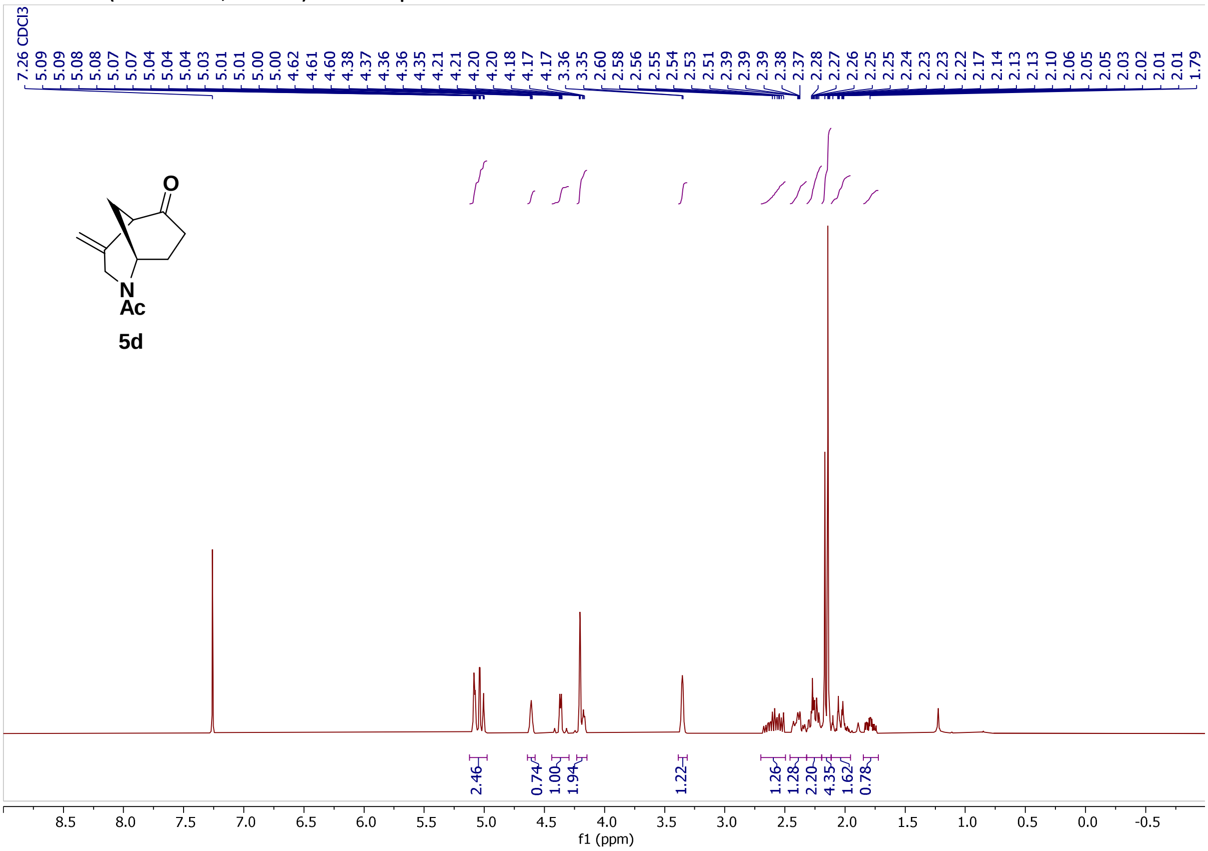

^{13}**C NMR** (101 MHz, $CDCl_3$) of compound **5d.**

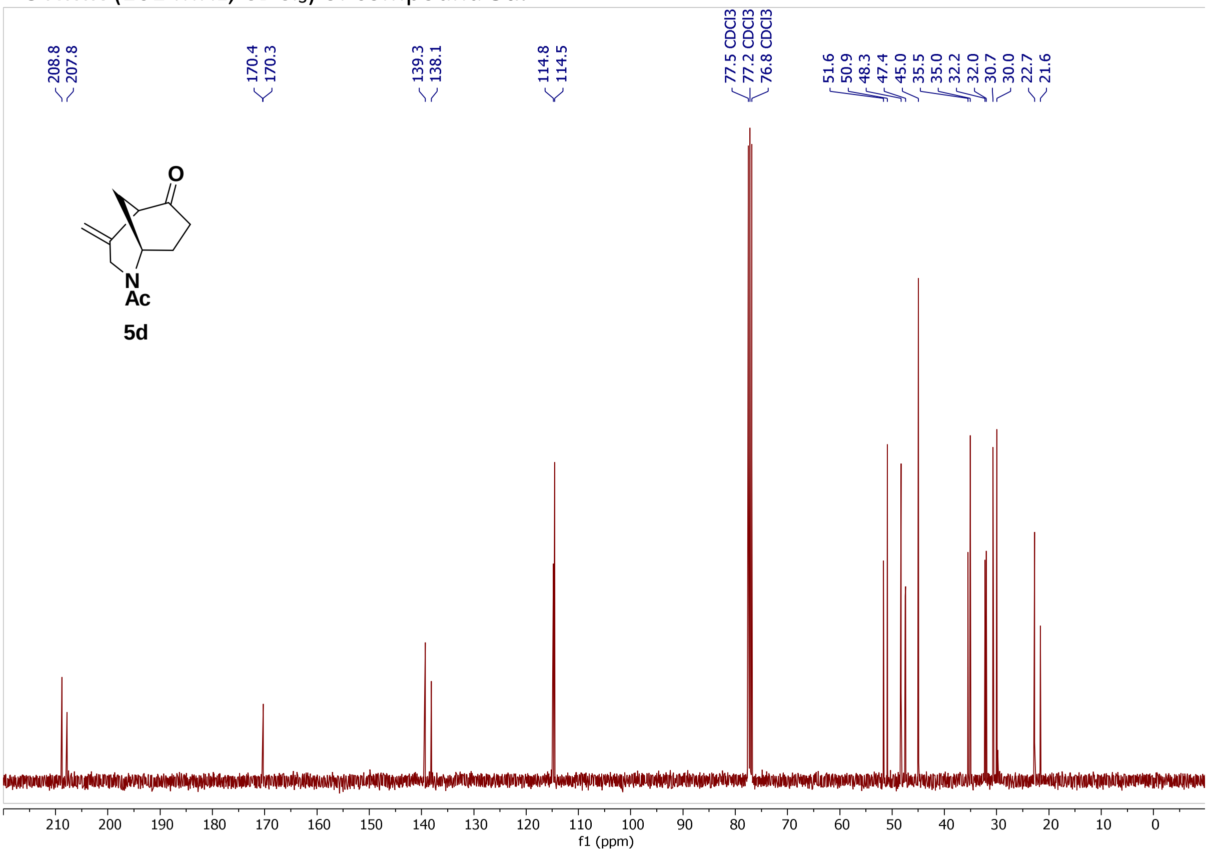

^{1}H NMR (400 MHz, $CDCl_3$) of compound **5e.**

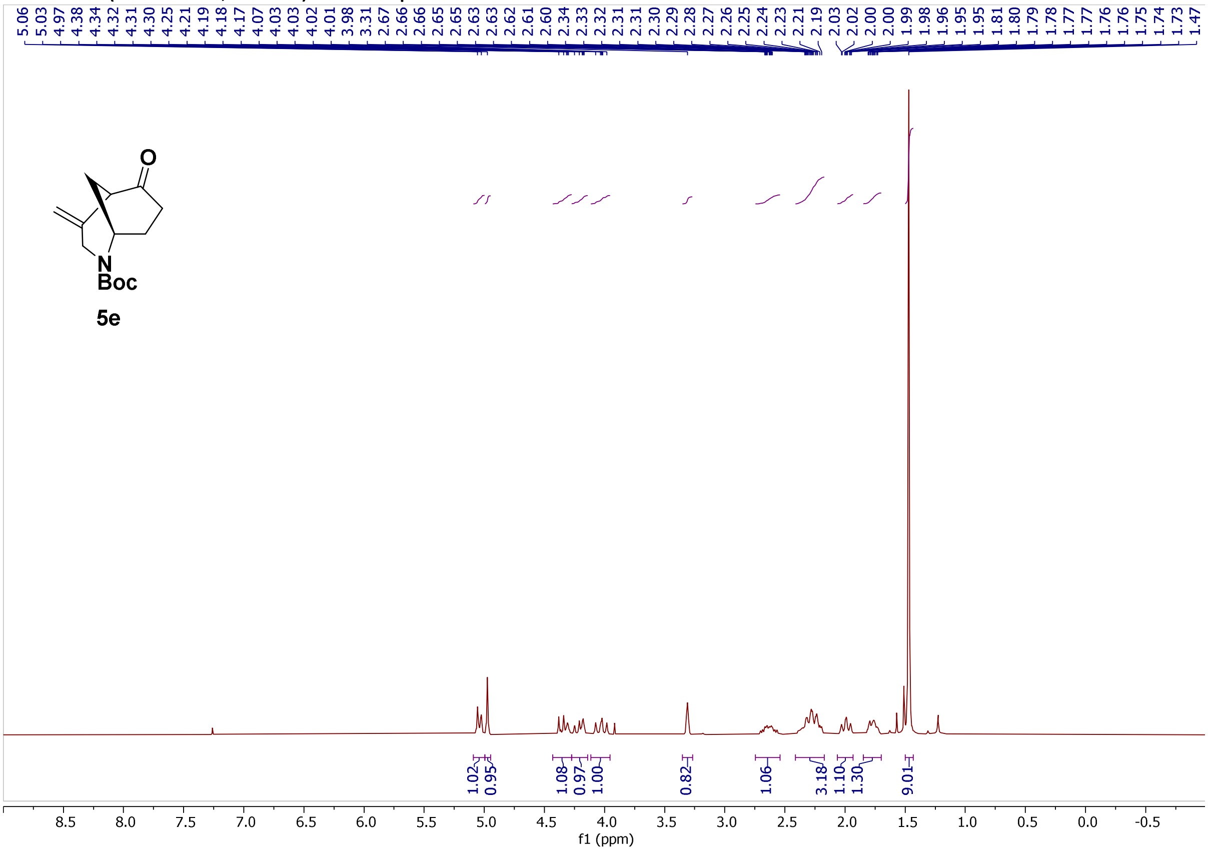

^{13}C NMR (101 MHz, $CDCl_3$) of compound **5e.**

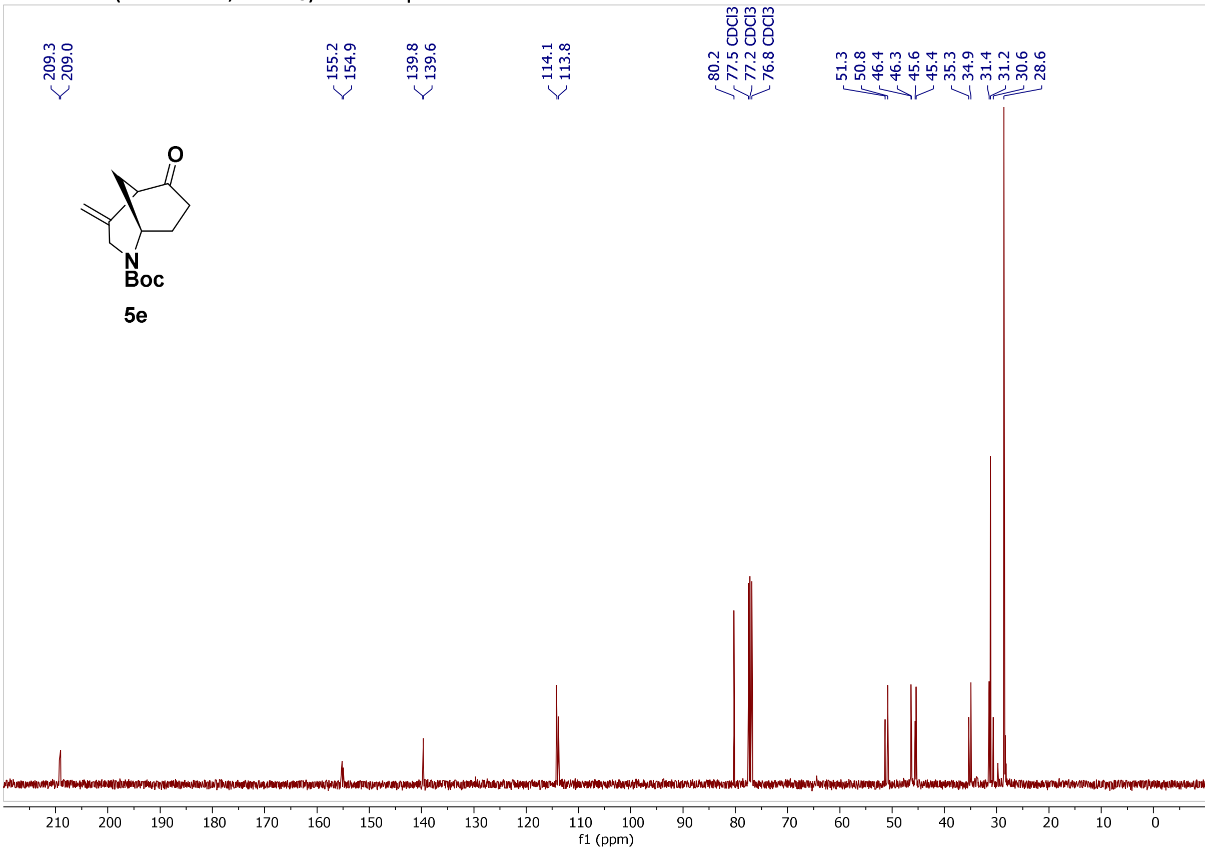

^{1}H NMR (400 MHz, $CDCl_3$) of compound **5f.**

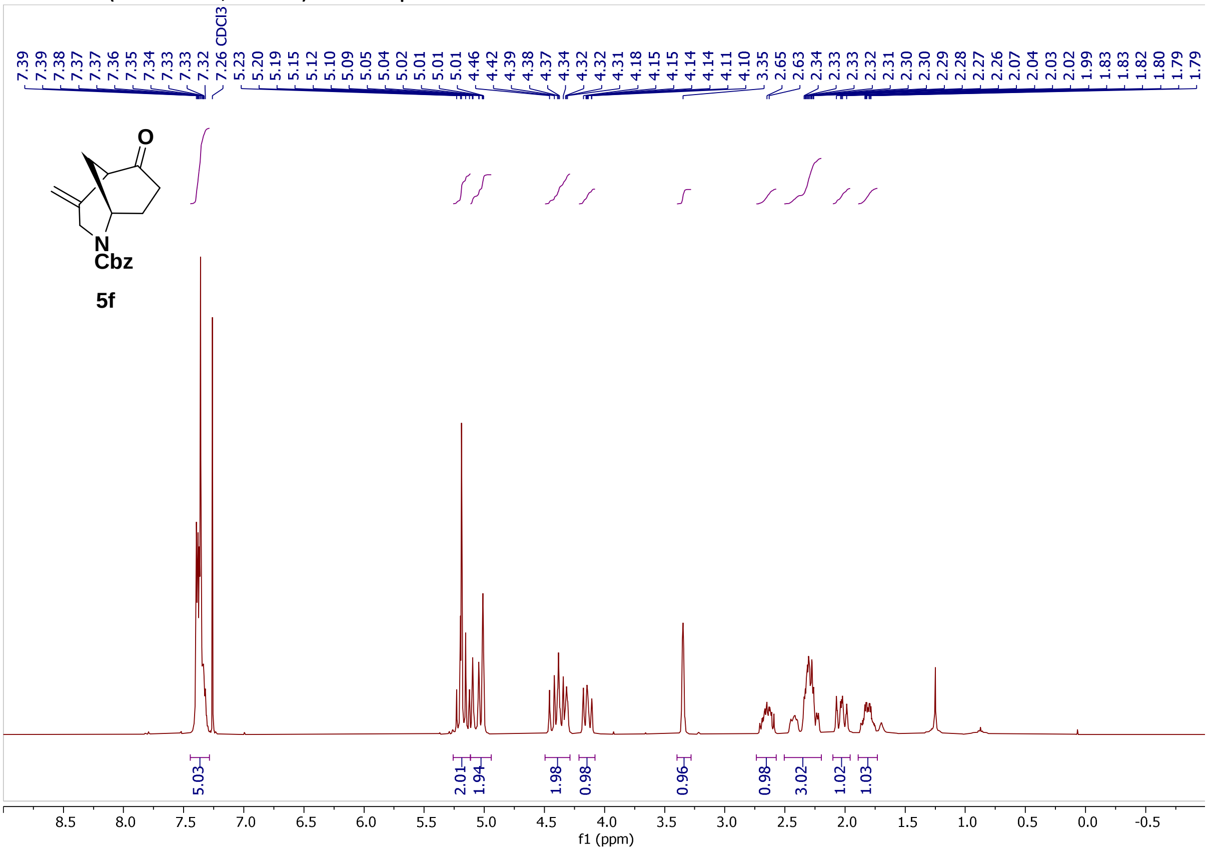

^{13}C NMR (101 MHz, $CDCl_3$) of compound **5f.**

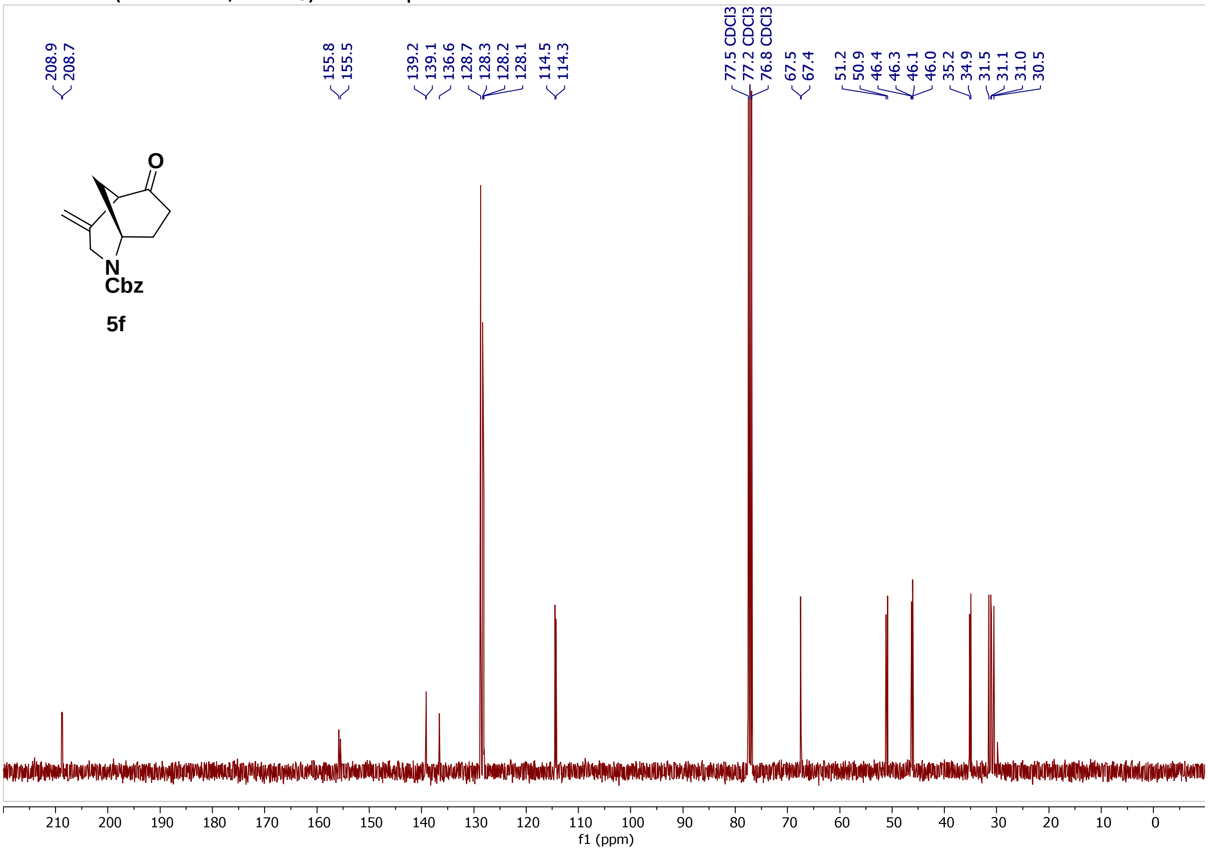

[1]**H NMR** (400 MHz, $CDCl_3$) of compound **5g.**

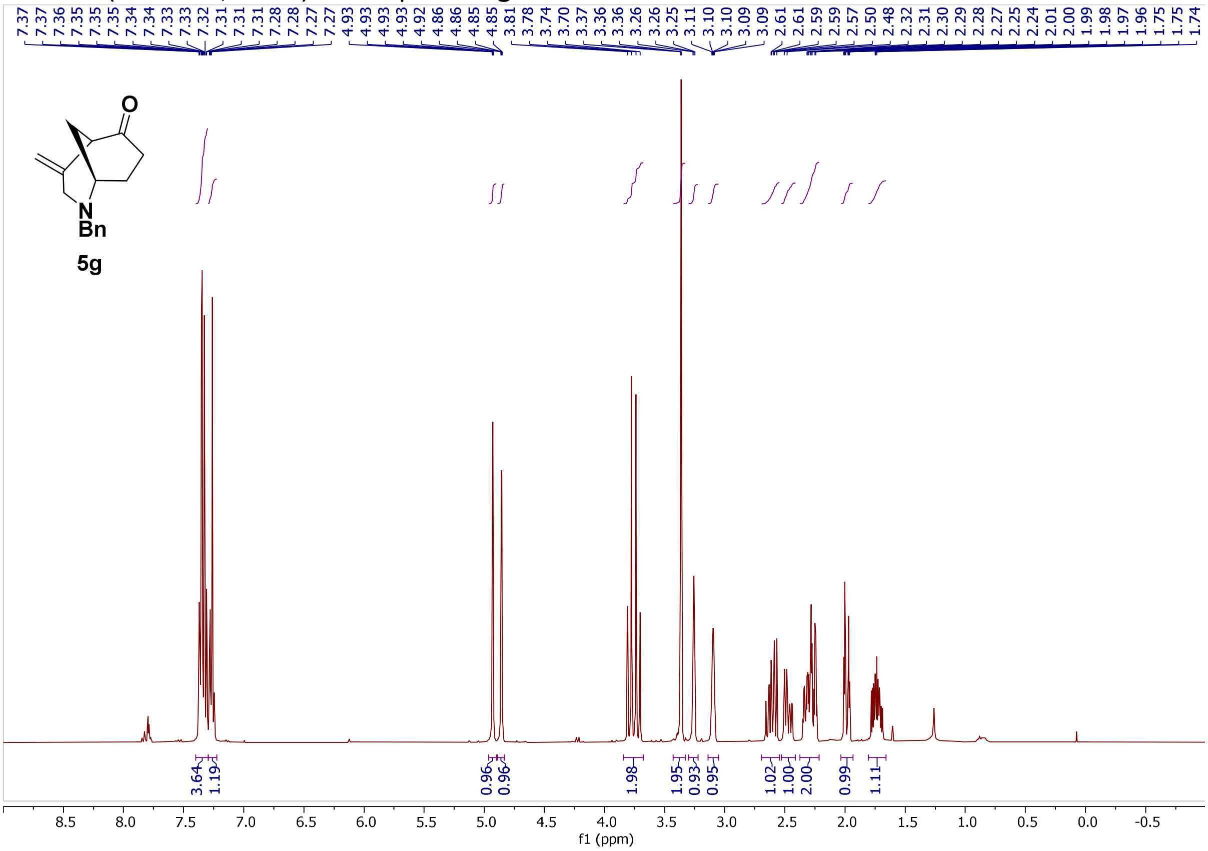

[13]**C NMR** (101 MHz, $CDCl_3$) of compound **5g.**

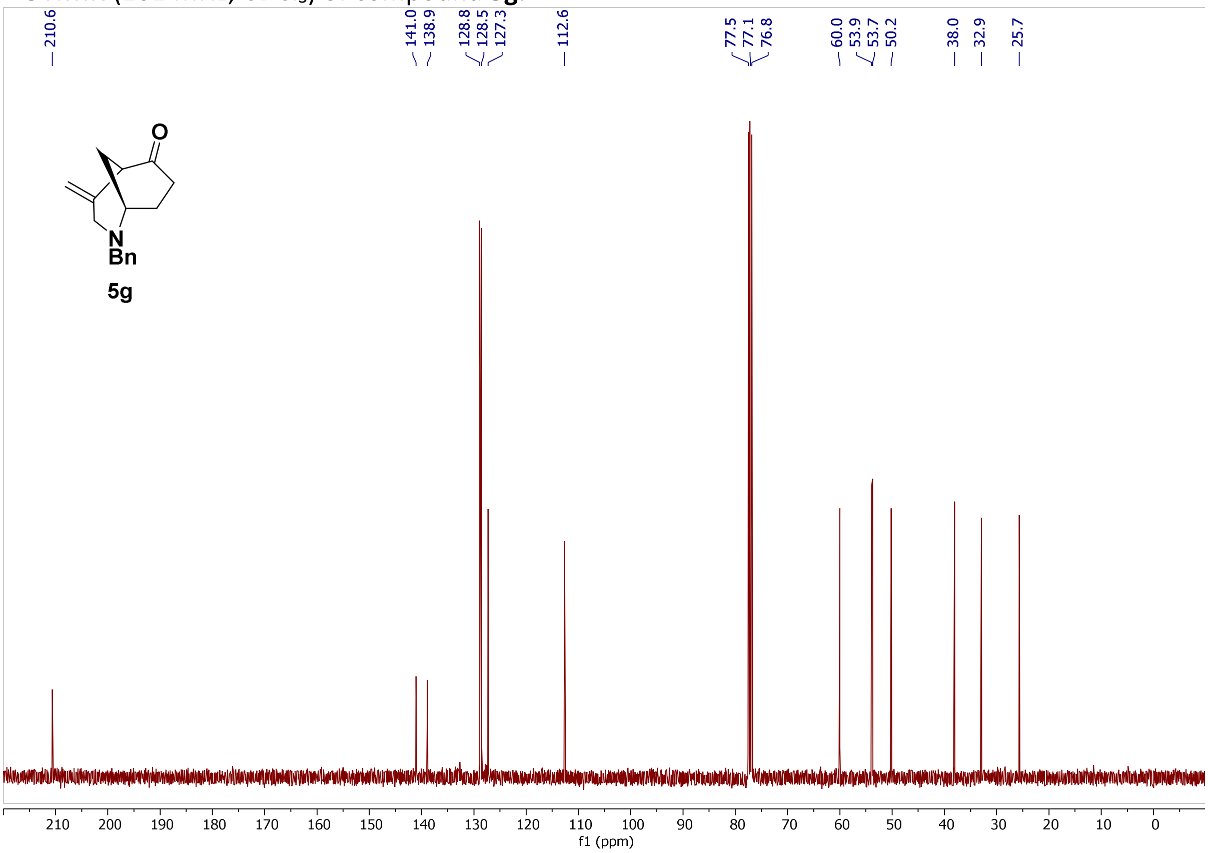

^{1}**H NMR** (400 MHz, $CDCl_3$) of compound **5h.**

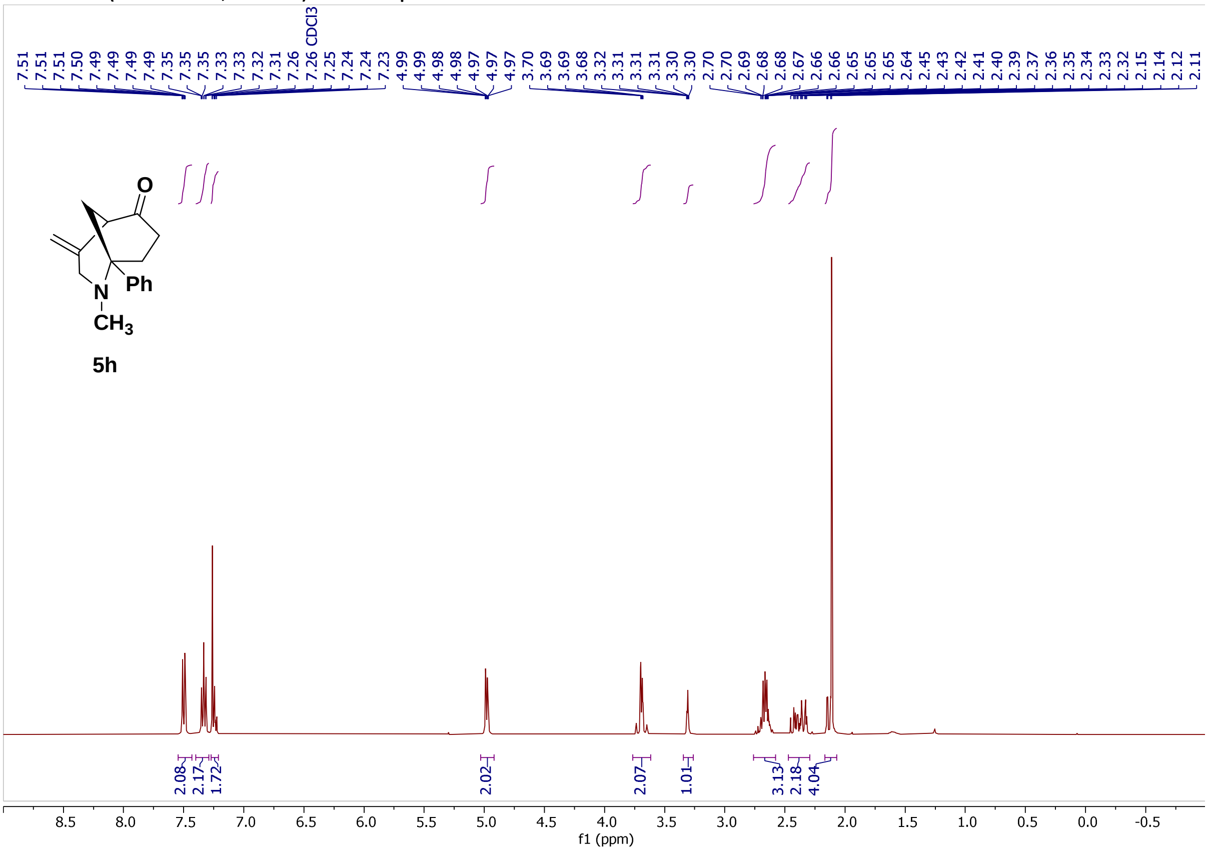

^{13}**C NMR** (101 MHz, $CDCl_3$) of compound **5h.**

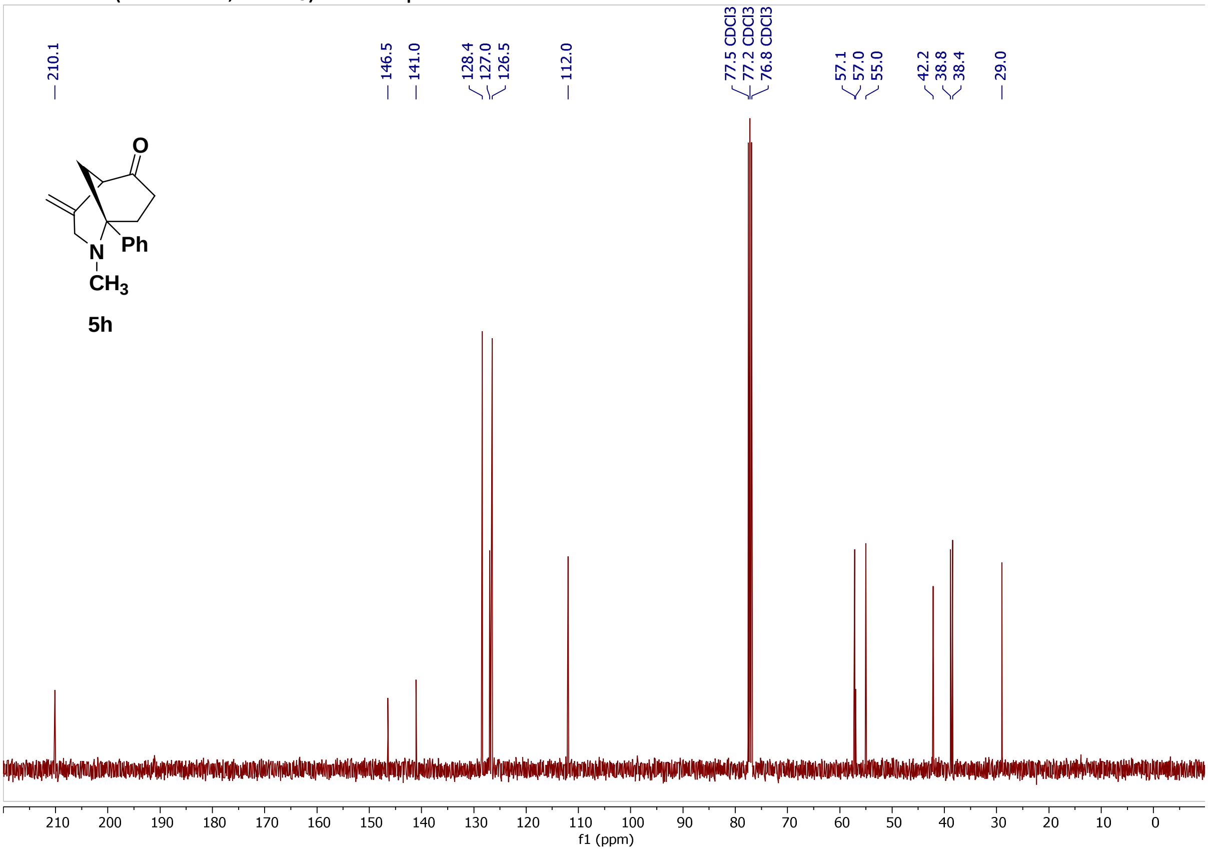

^{1}H NMR (400 MHz, $CDCl_3$) of compound **5i.**

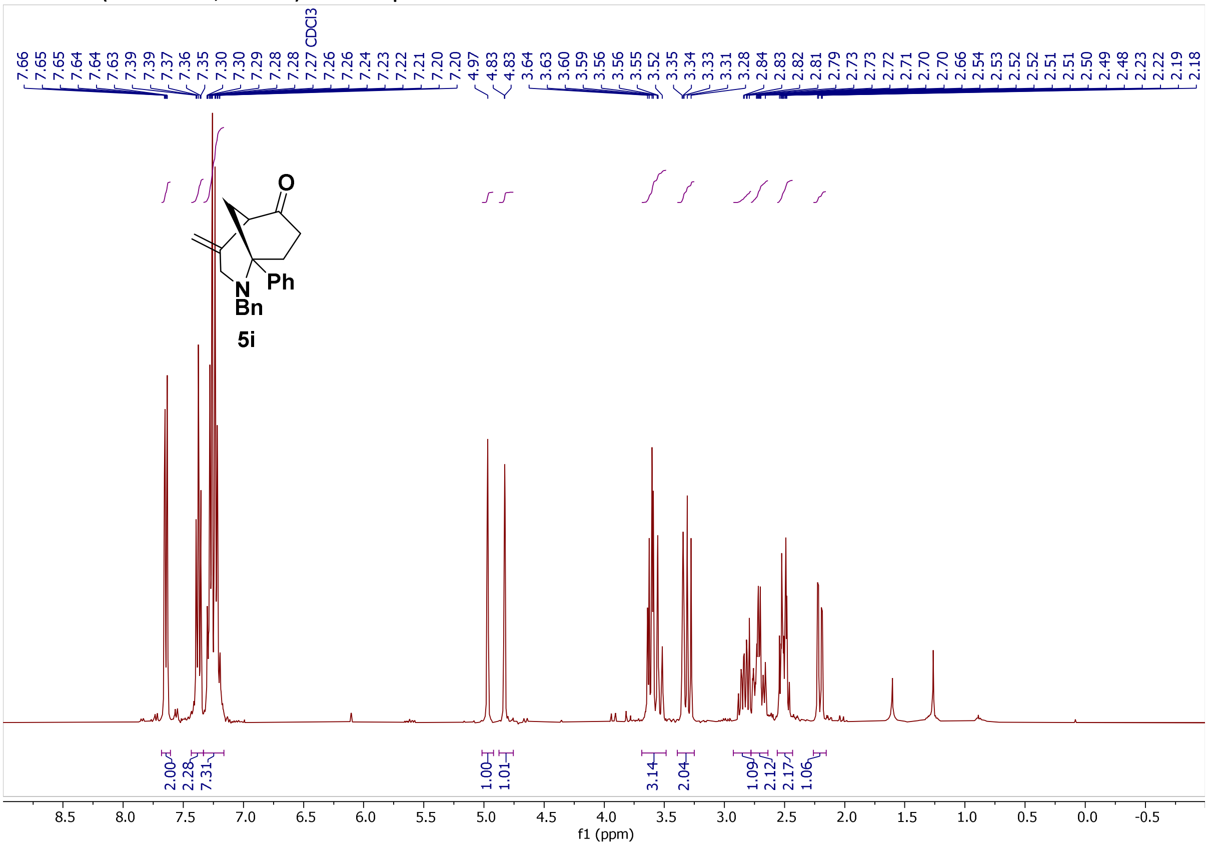

^{13}C NMR (101 MHz, $CDCl_3$) of compound **5i.**

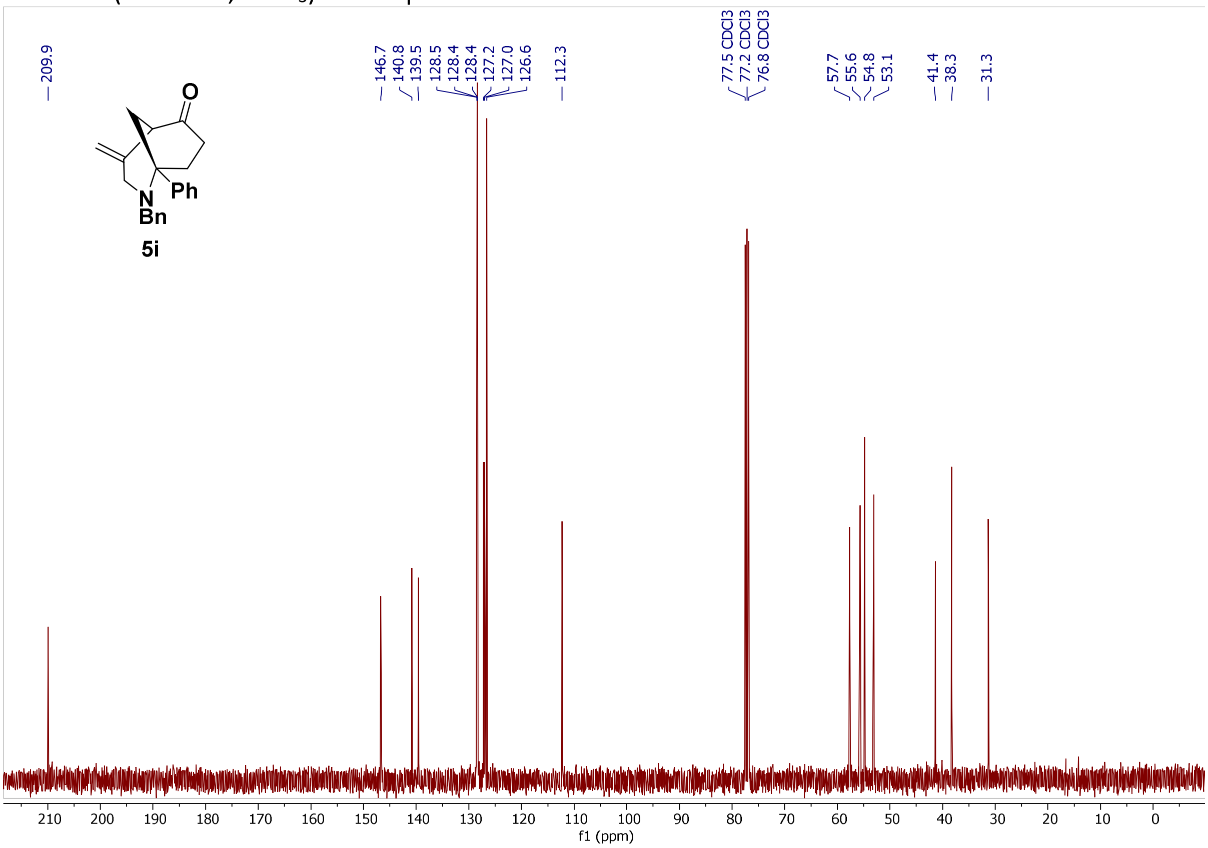

^{1}H NMR (400 MHz, $CDCl_3$) of compound **5j.**

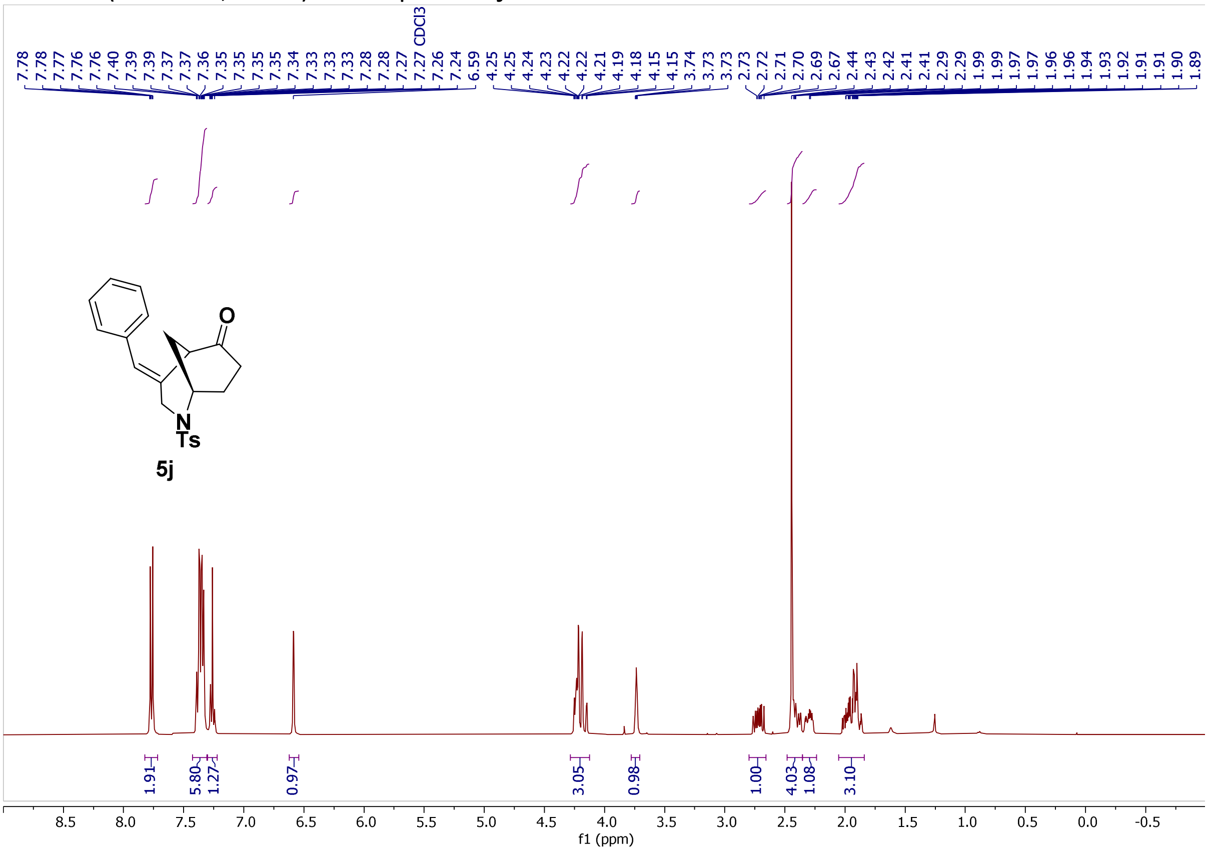

^{13}C NMR (101 MHz, $CDCl_3$) of compound **5j.**

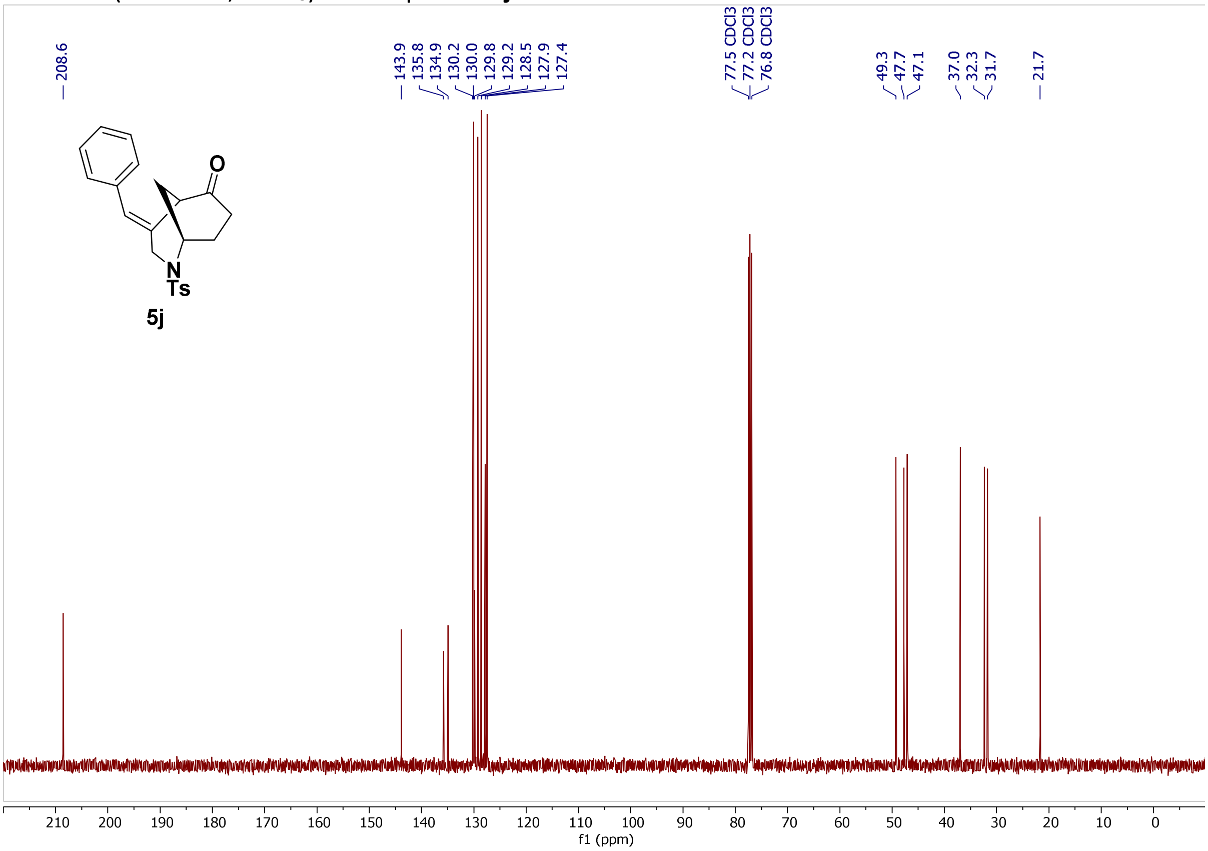

^{1}**H NMR** (400 MHz, $CDCl_3$) of compound **5k.**

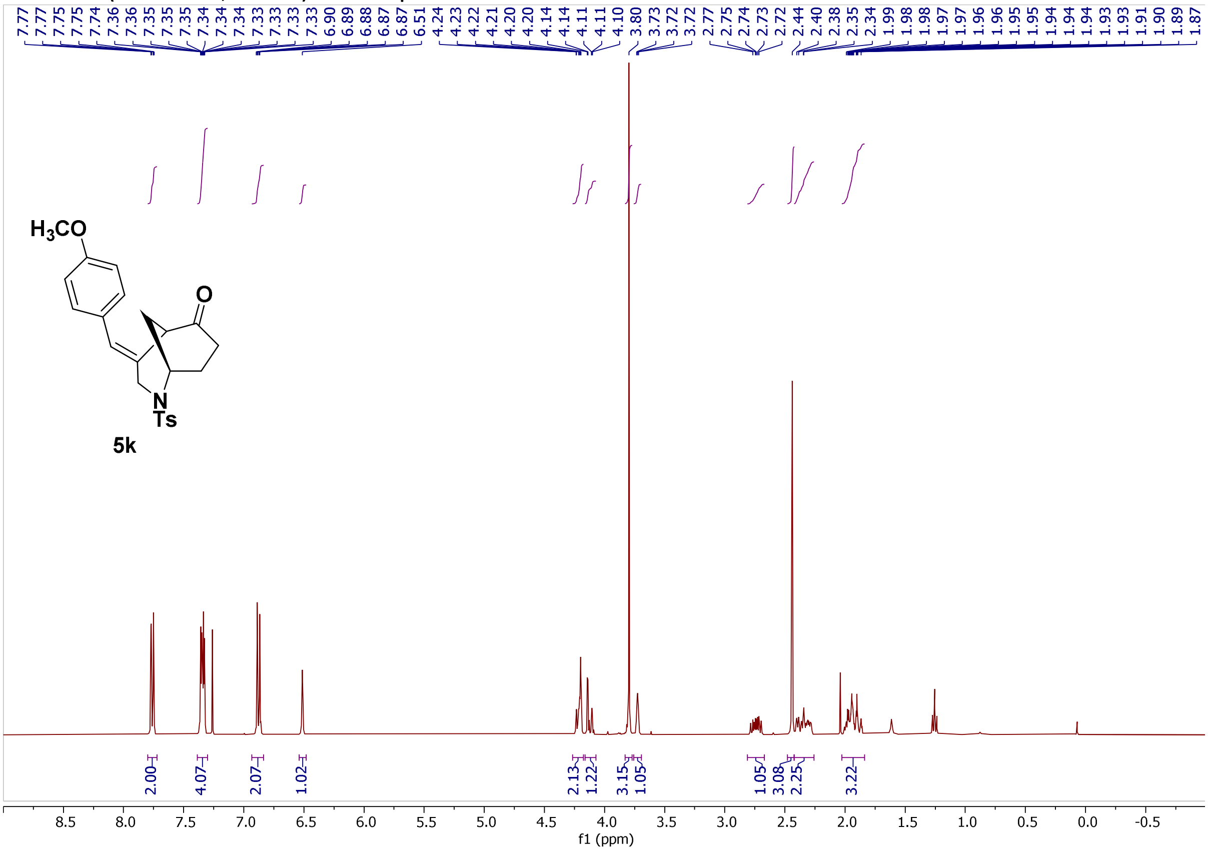

^{13}**C NMR** (101 MHz, $CDCl_3$) of compound **5k.**

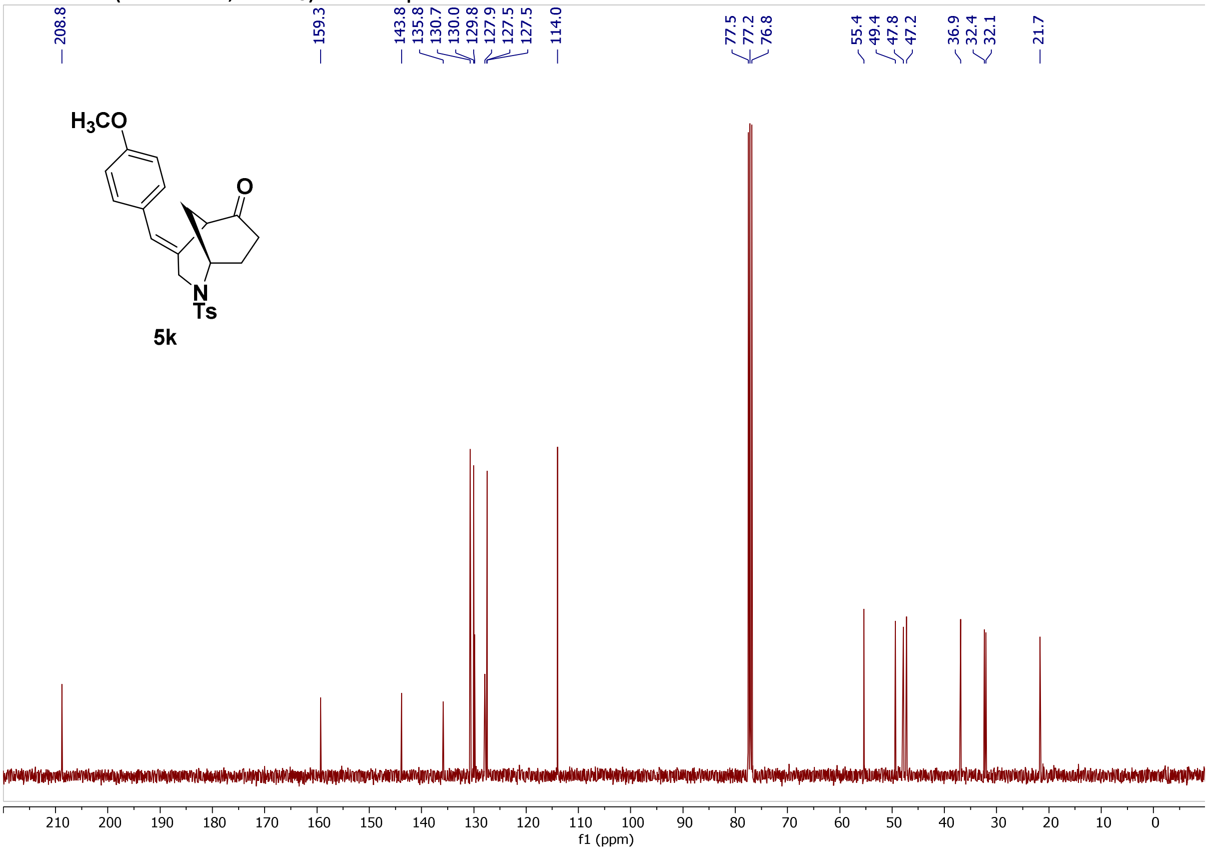

^{1}H NMR (400 MHz, $CDCl_3$) of compound **5l.**

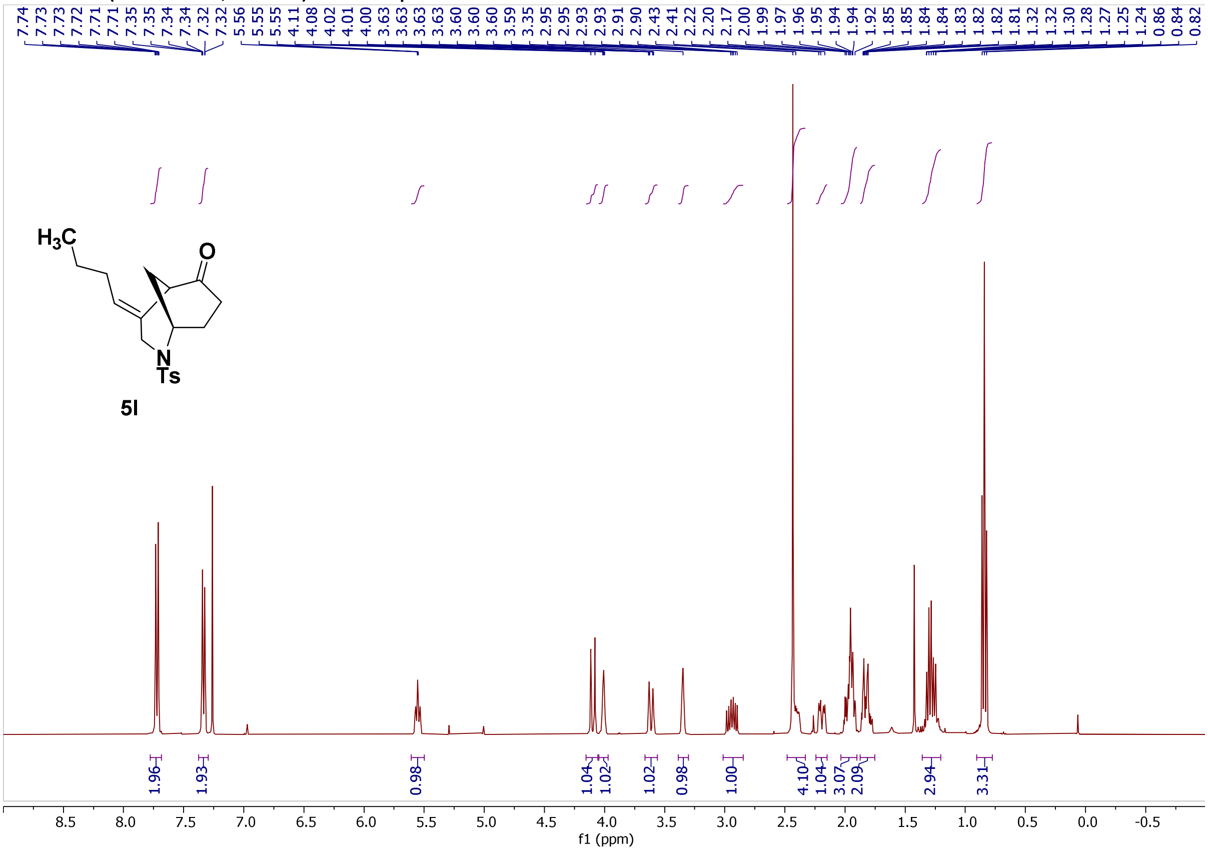

^{13}C NMR (101 MHz, $CDCl_3$) of compound **5l.**

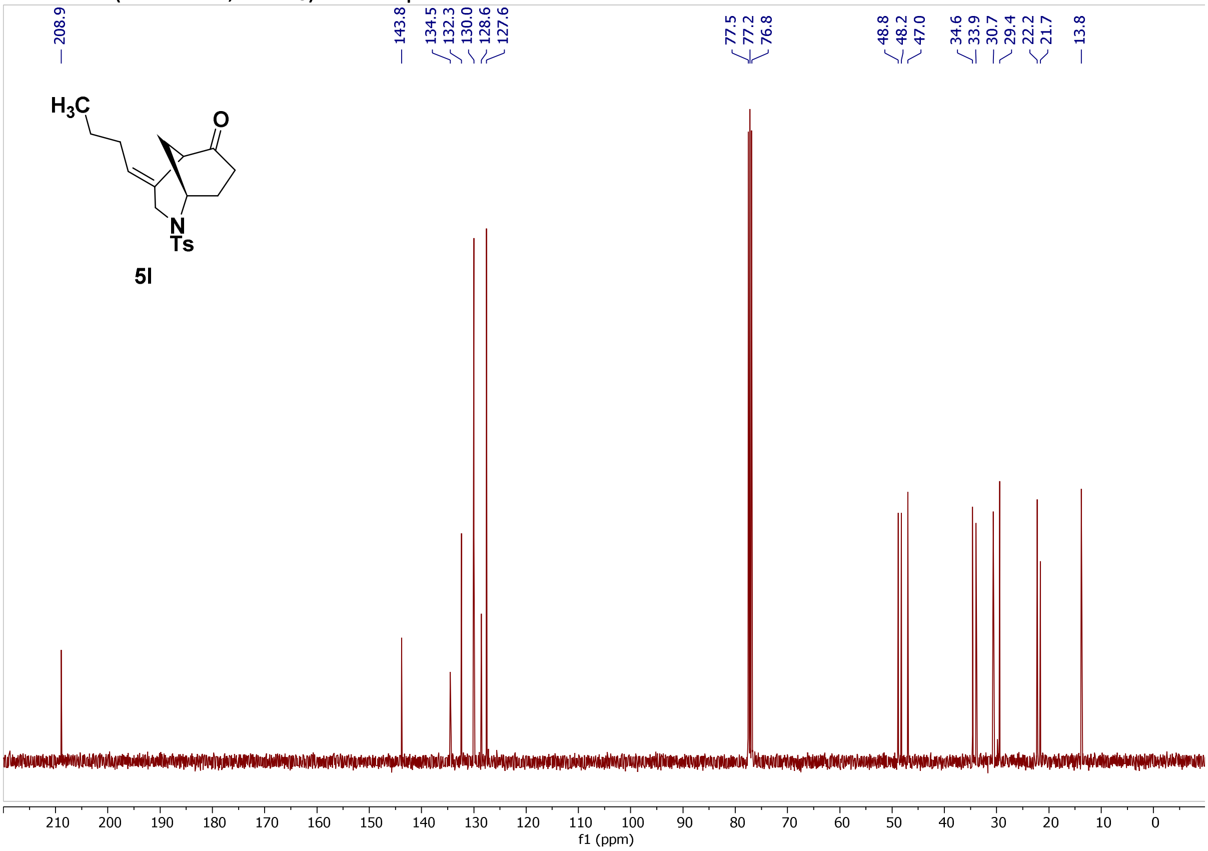

^{1}H NMR (400 MHz, $CDCl_3$) of compound **S30.**

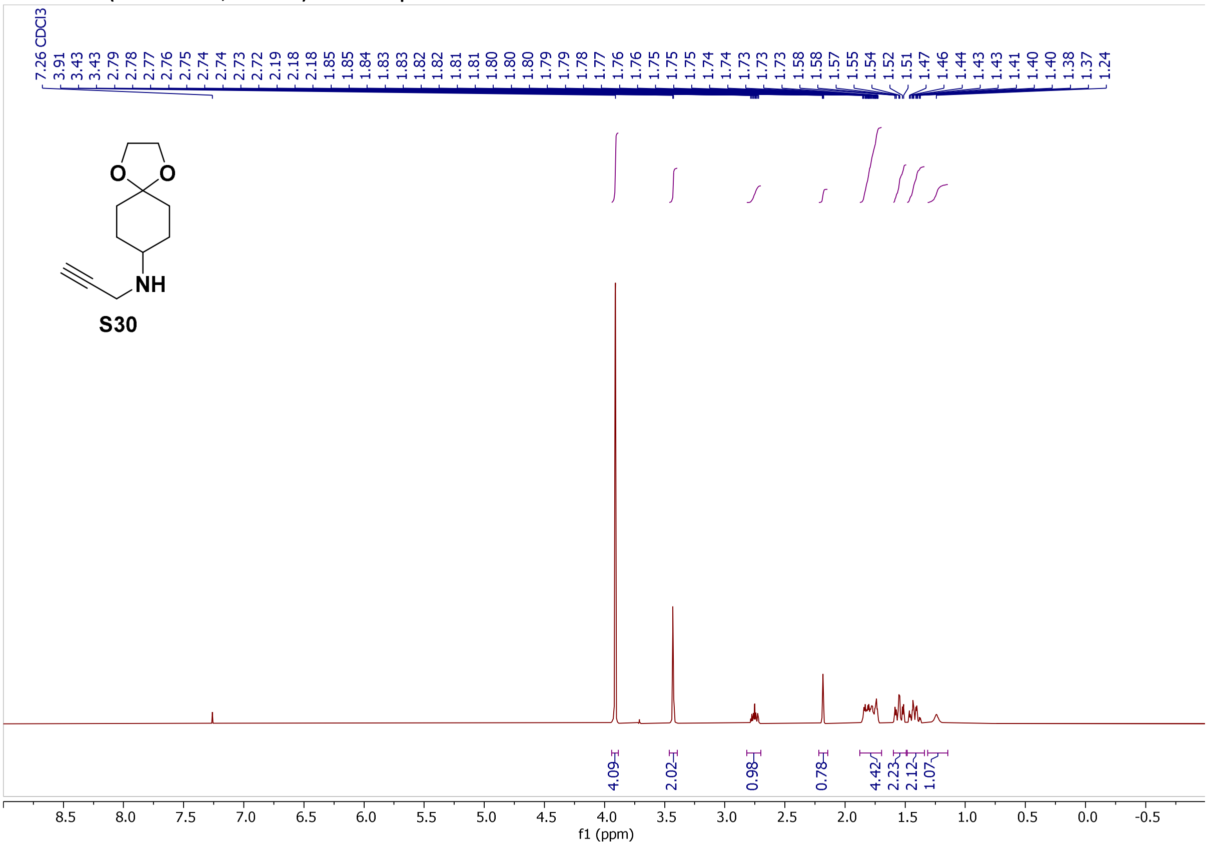

^{13}C NMR (101 MHz, $CDCl_3$) of compound **S30.**

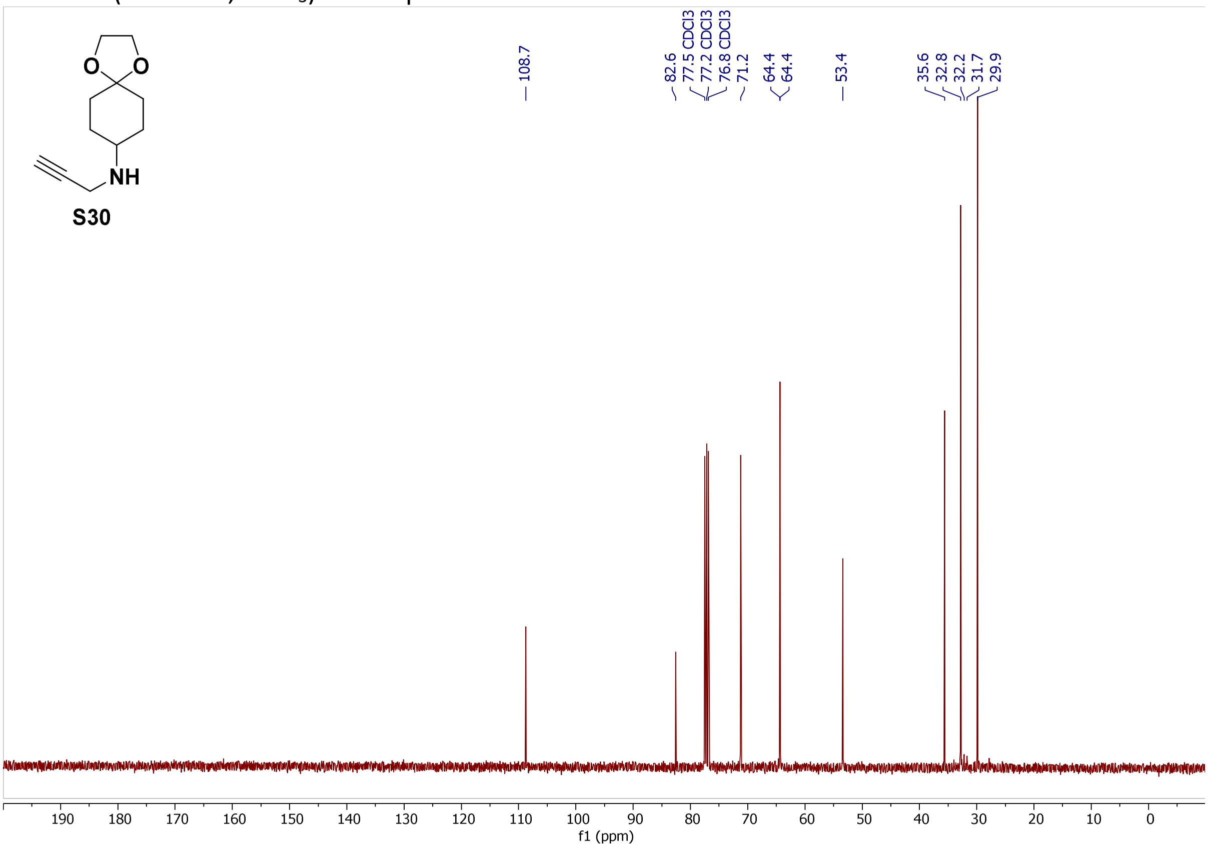

^{1}H NMR (400 MHz, $CDCl_3$) of crude product **S31.**

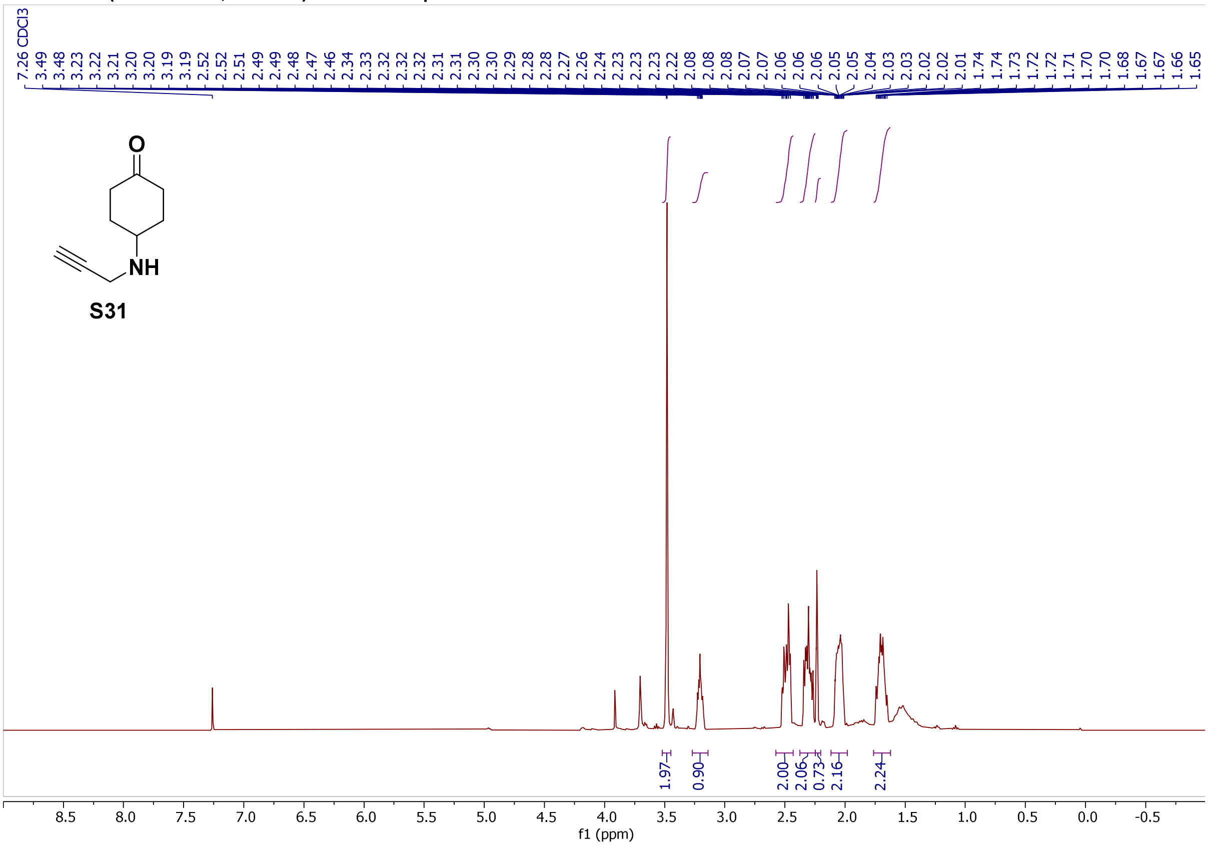

^{13}C NMR (101 MHz, $CDCl_3$) of crude product **S31.**

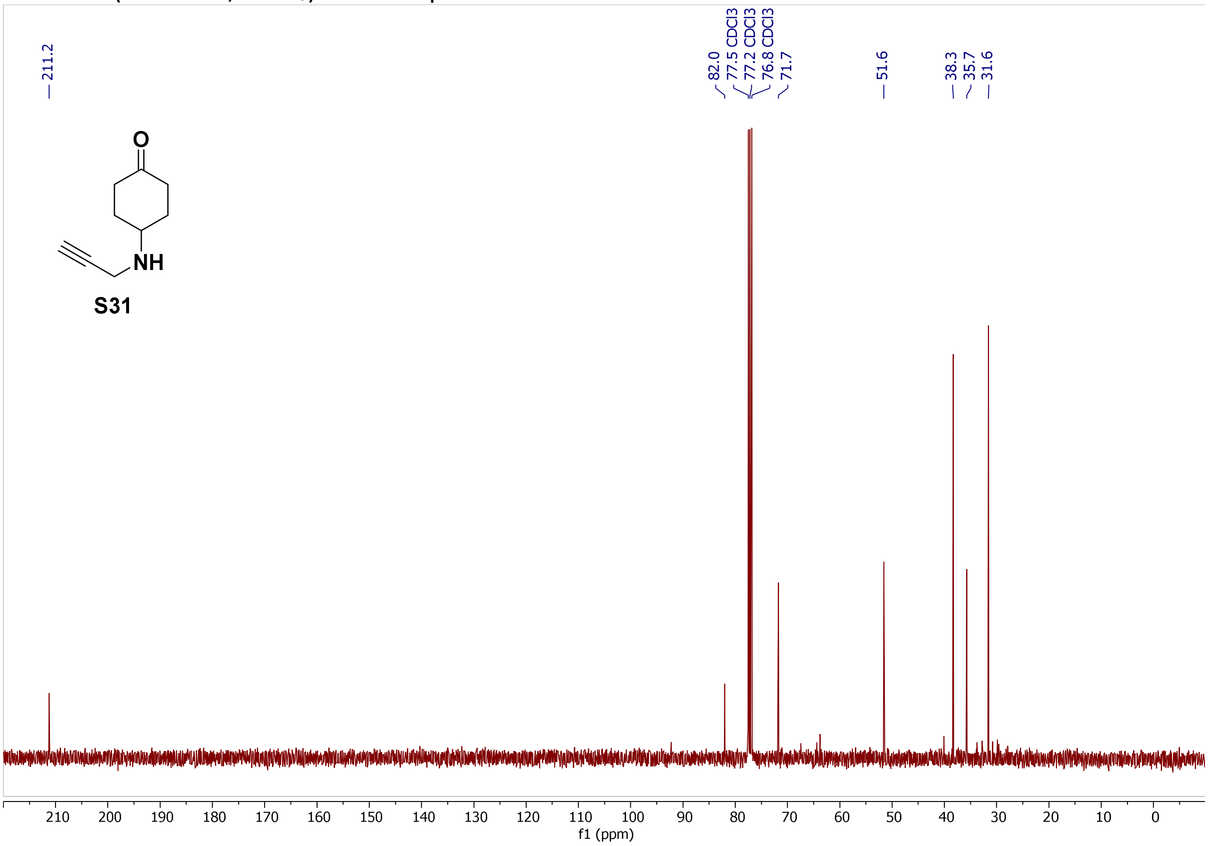

^{1}**H NMR** (400 MHz, $CDCl_3$) of compound **S32.**

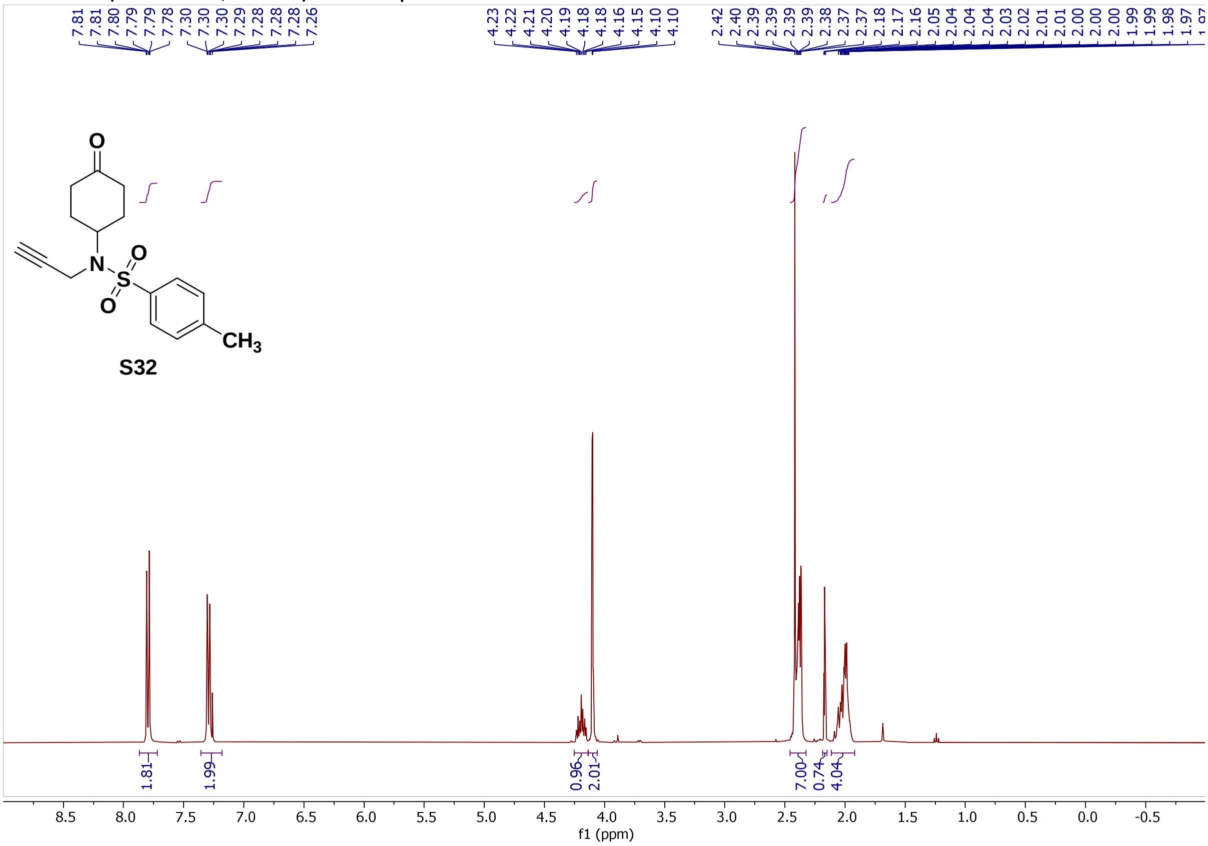

^{13}**C NMR** (101 MHz, $CDCl_3$) of compound **S32.**

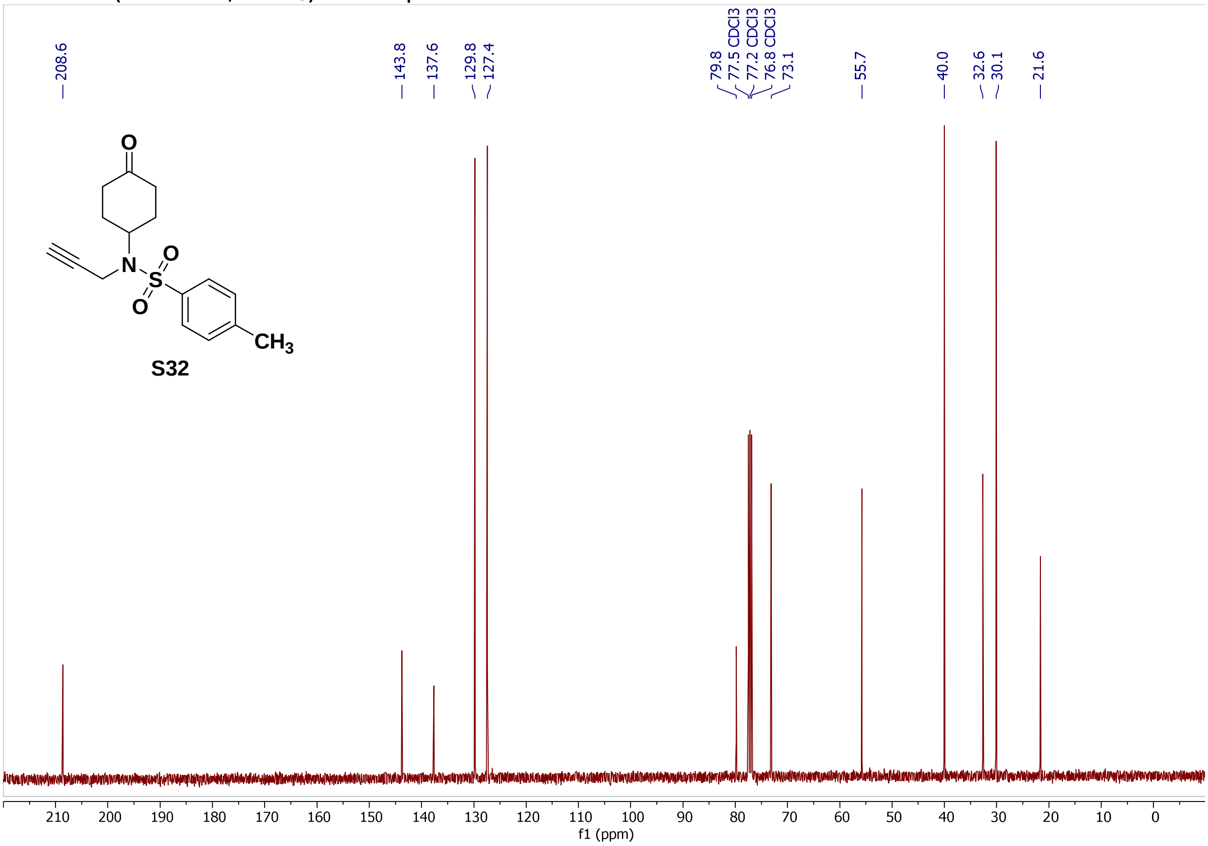

^{1}**H NMR** (400 MHz, $CDCl_3$) of compound **S28.**

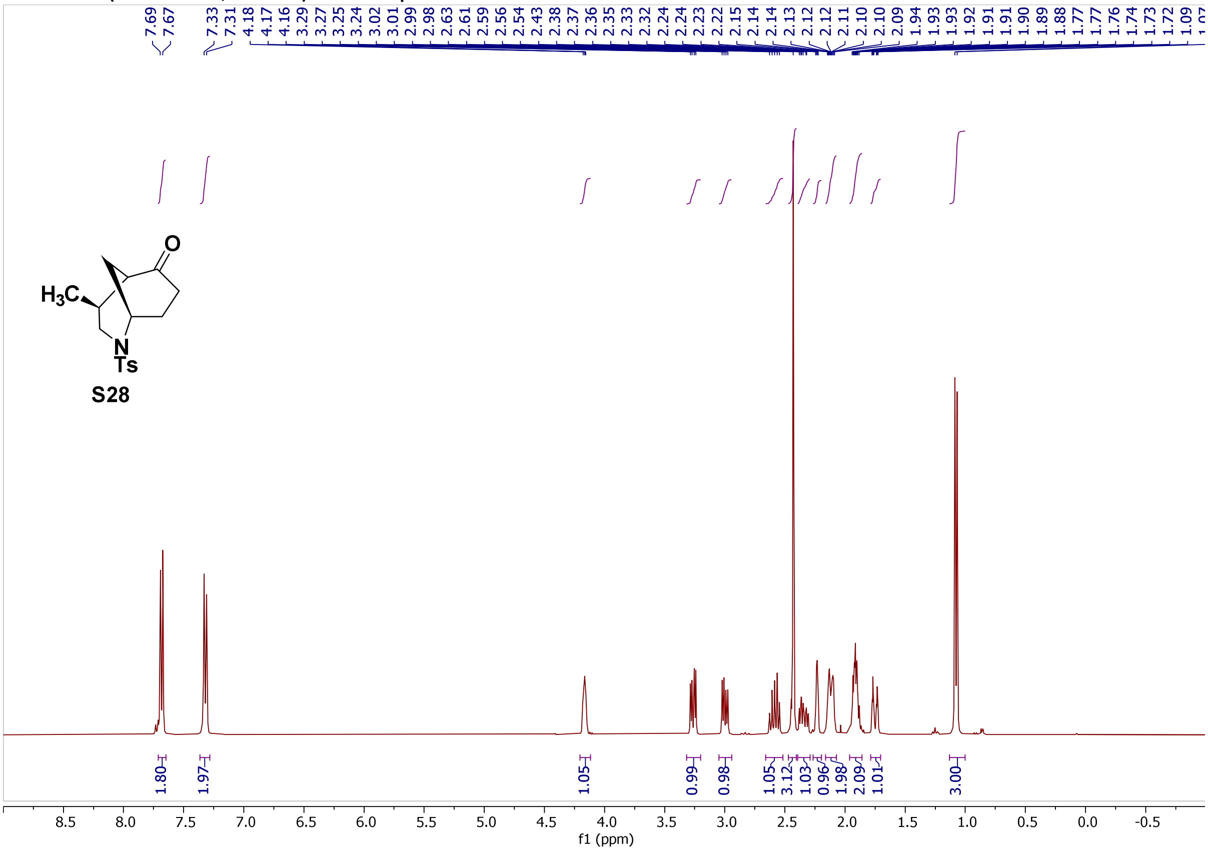

^{13}**C NMR** (101 MHz, $CDCl_3$) of compound **S28.**

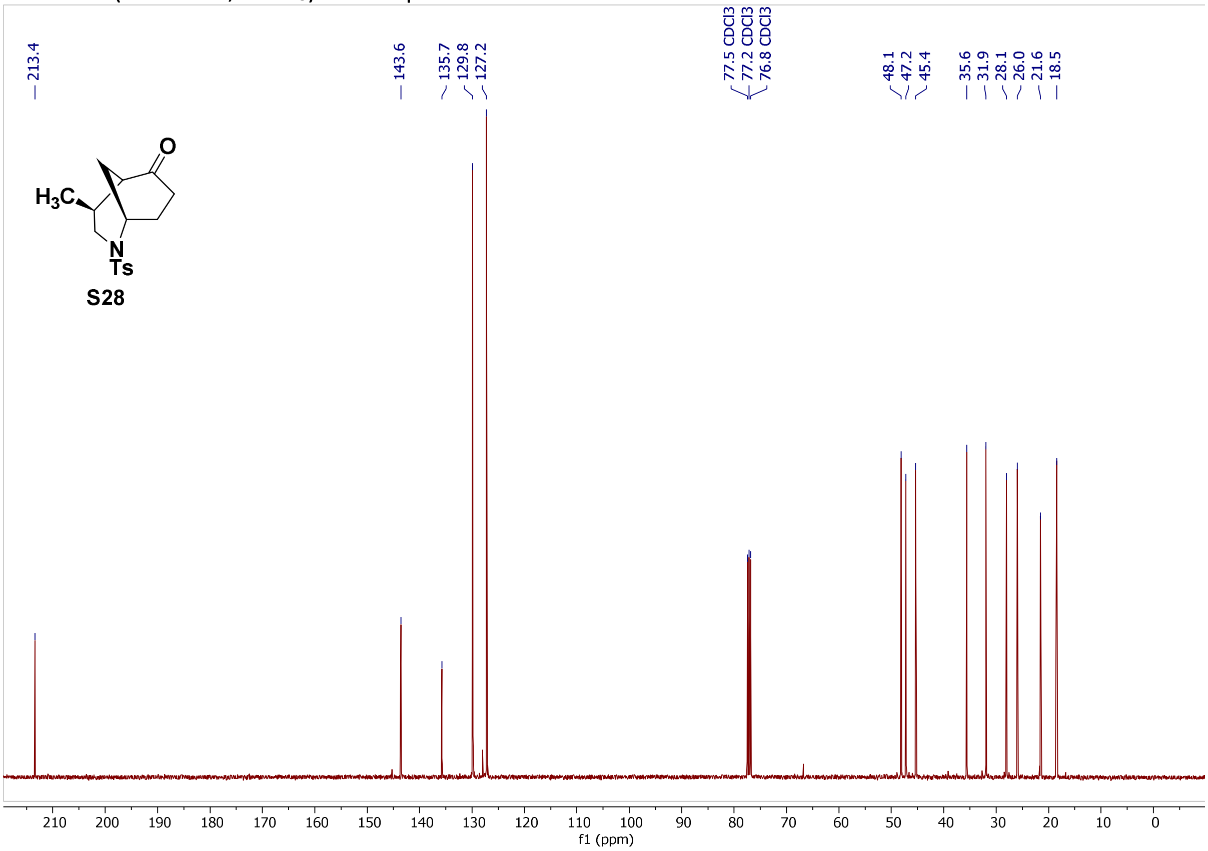

^{1}H NMR (400 MHz, $CDCl_3$) of compound **15.**

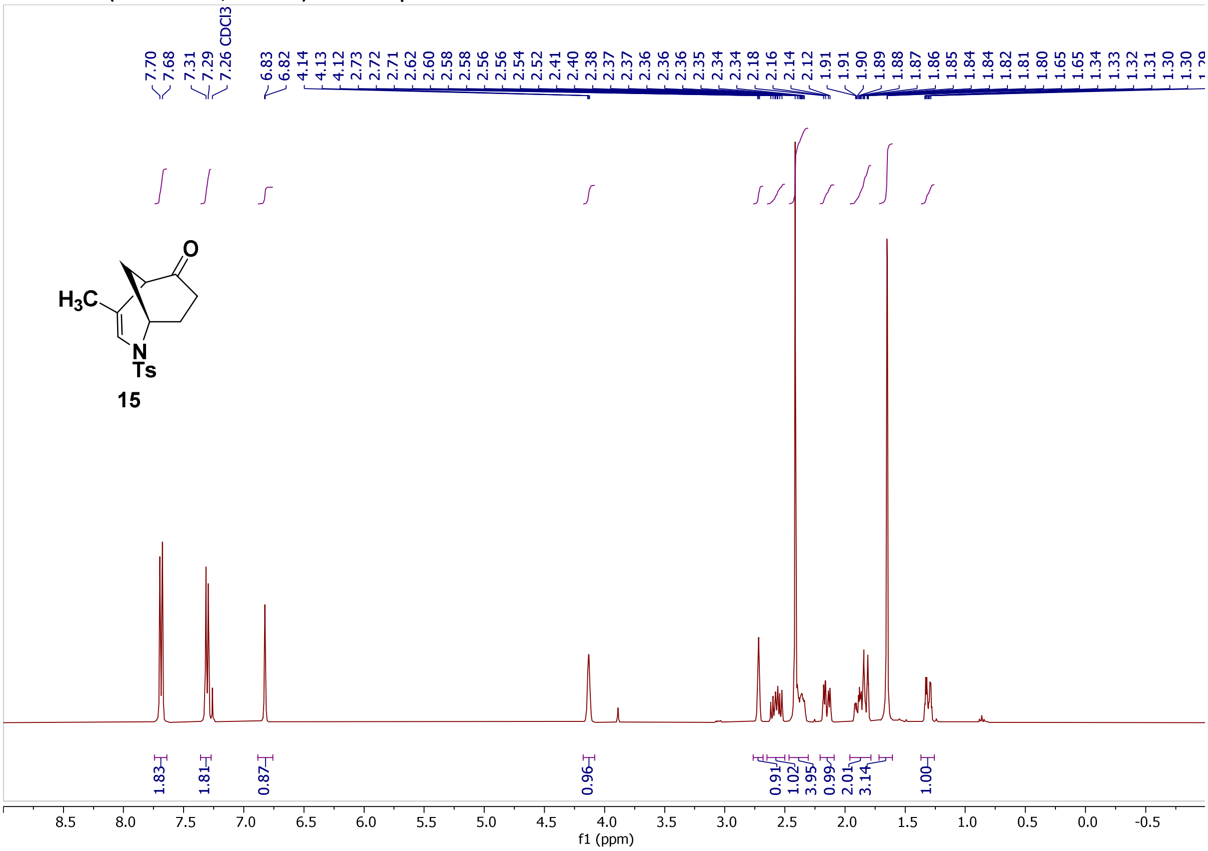

^{13}C NMR (101 MHz, $CDCl_3$) of compound **15.**

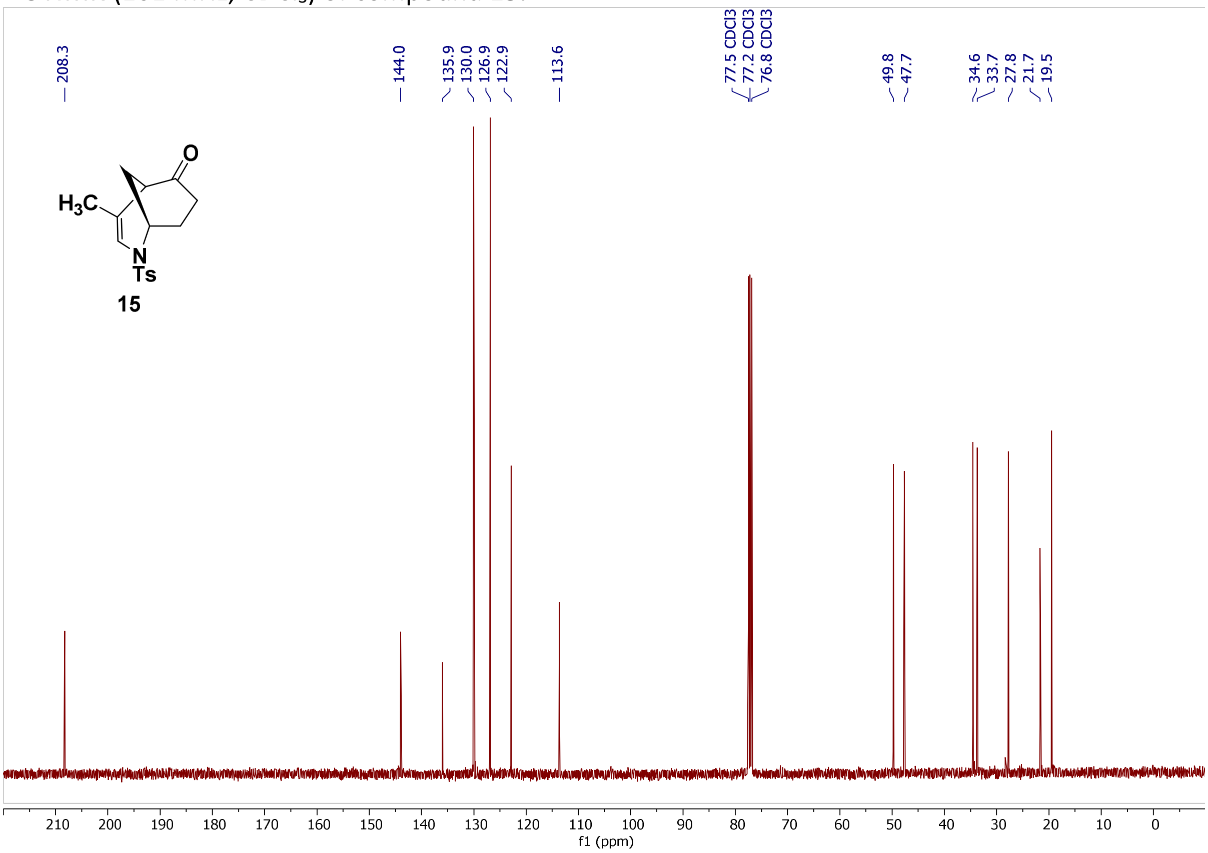

^{1}H NMR (400 MHz, $CDCl_3$) of compound **S33.**

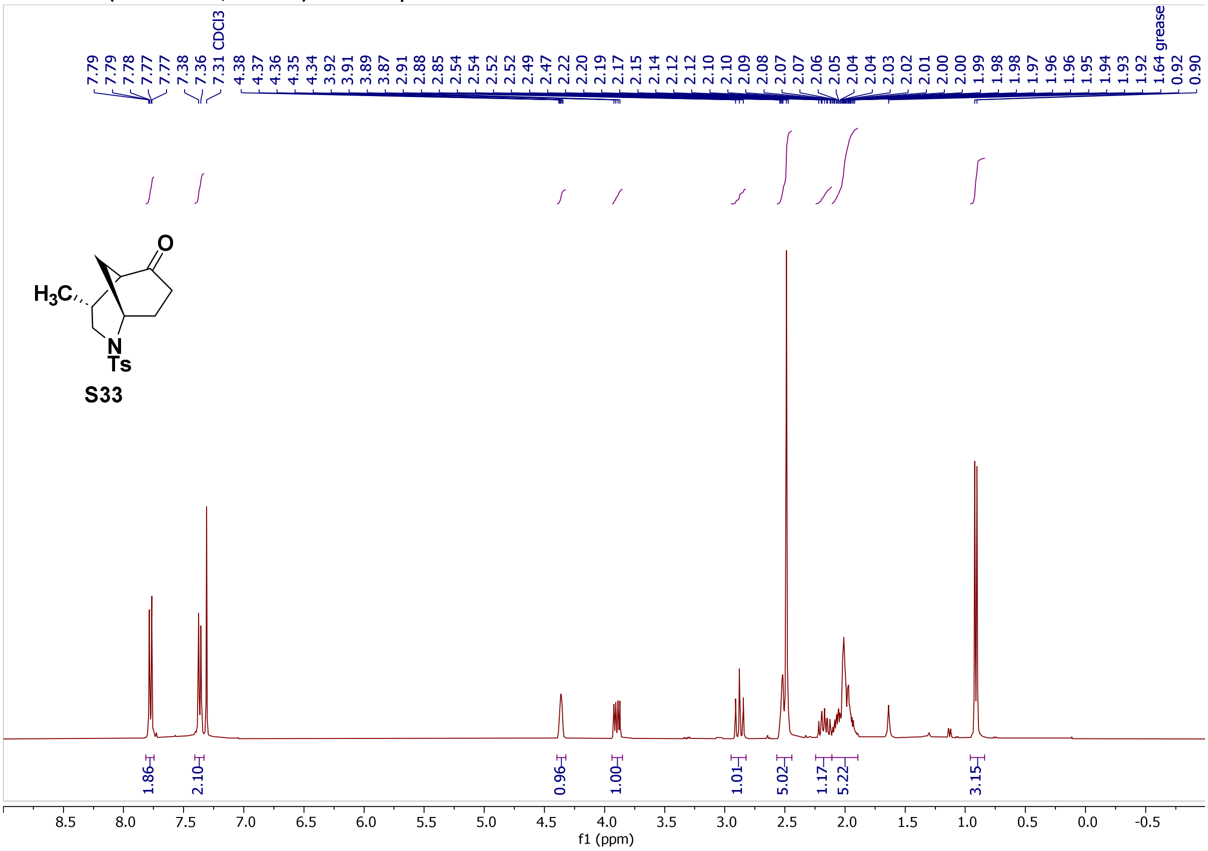

^{13}C NMR (101 MHz, $CDCl_3$) of compound **S33.**

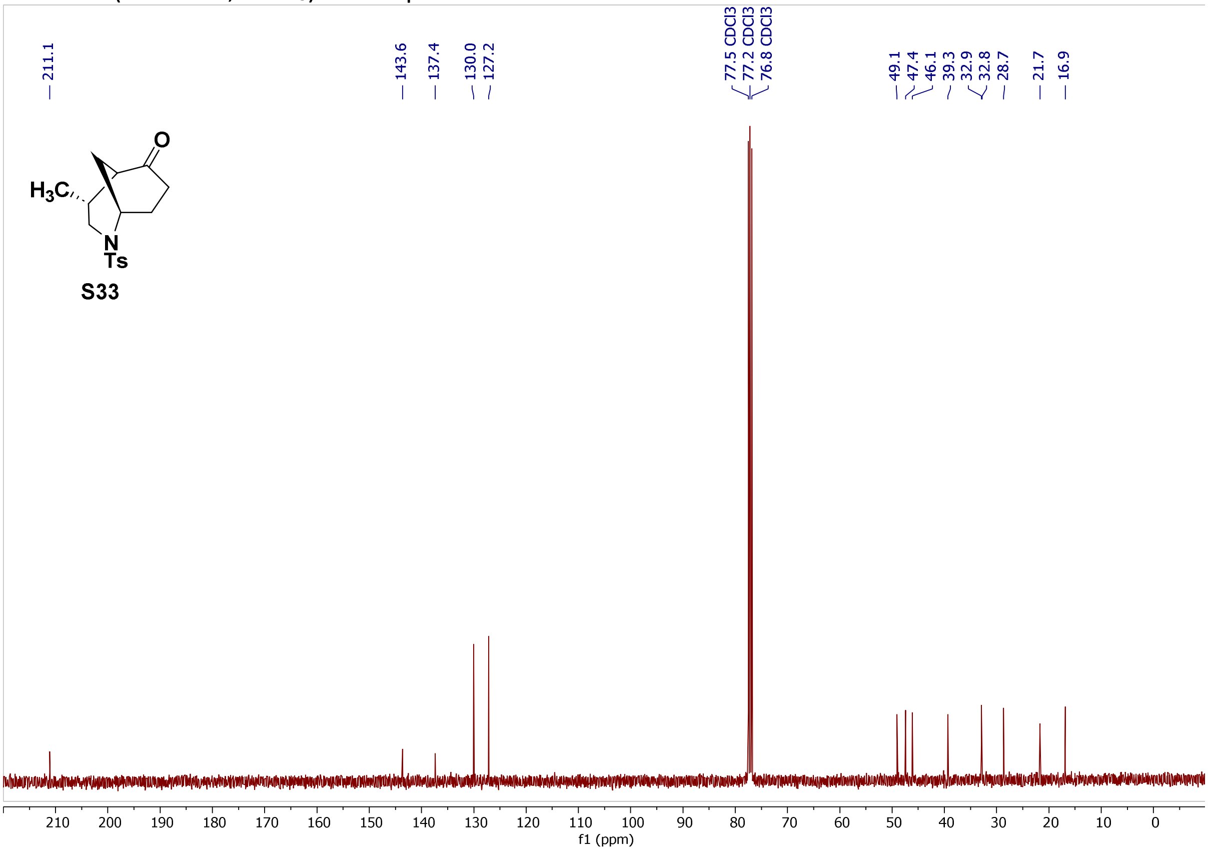

^{1}H NMR (400 MHz, $CDCl_3$) of compound **16.**

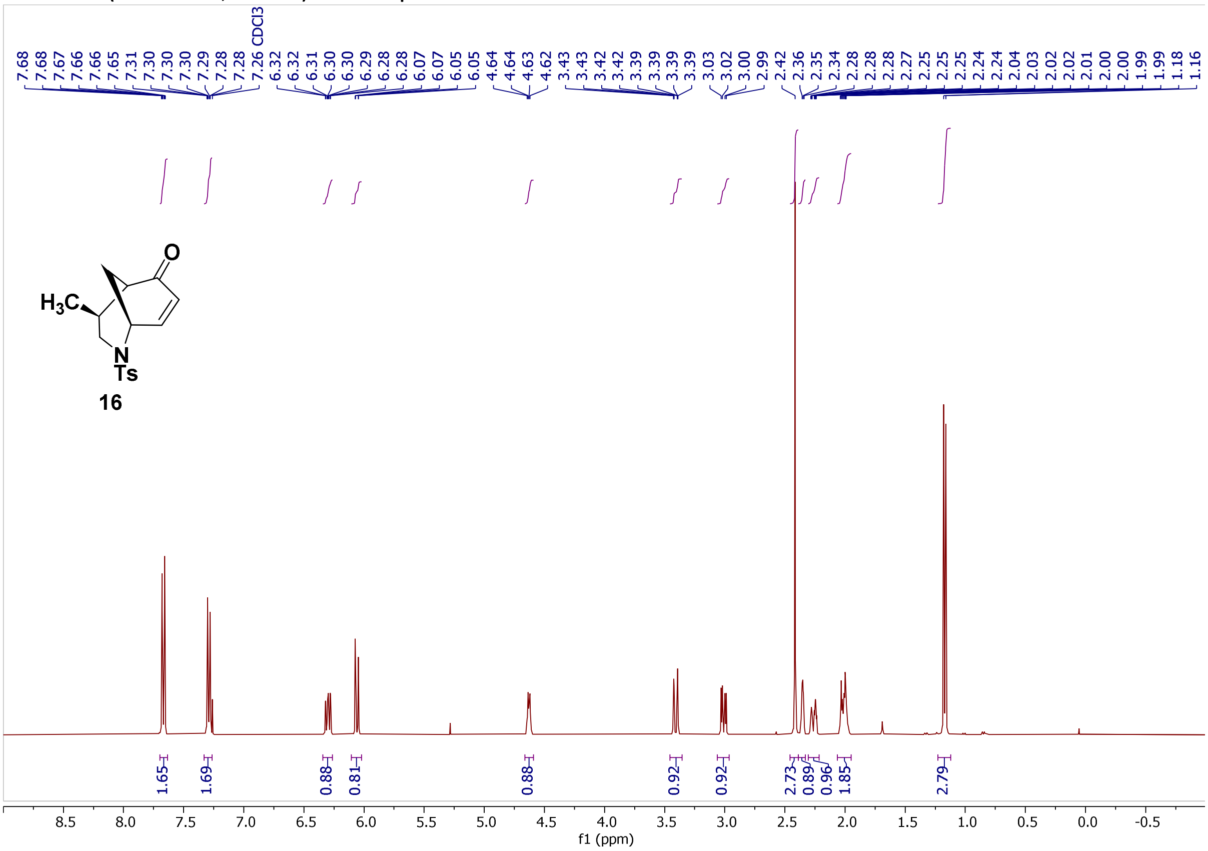

^{13}C NMR (101 MHz, $CDCl_3$) of compound **16.**

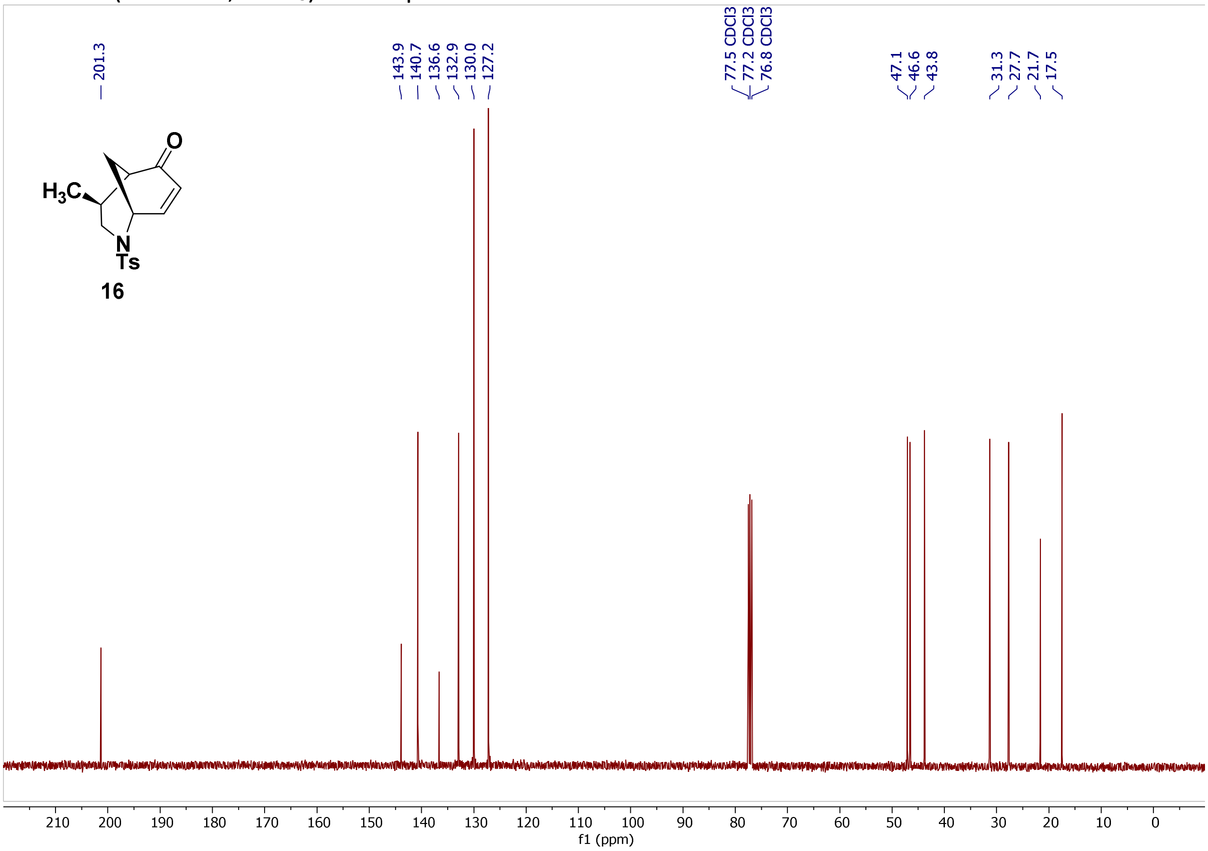

^{1}**H NMR** (400 MHz, C_6D_6) of compound **S29.**

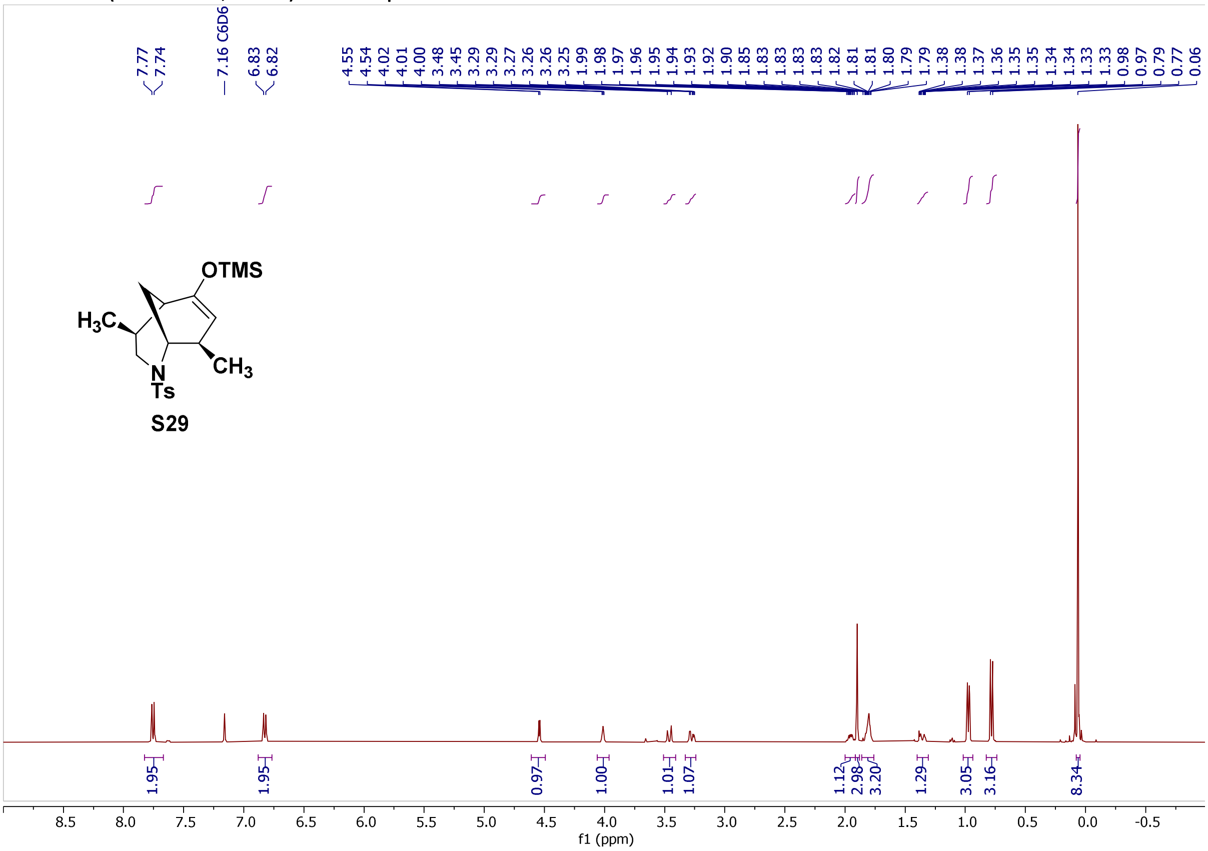

^{13}**C NMR** (101 MHz, C_6D_6) of compound **S29.**

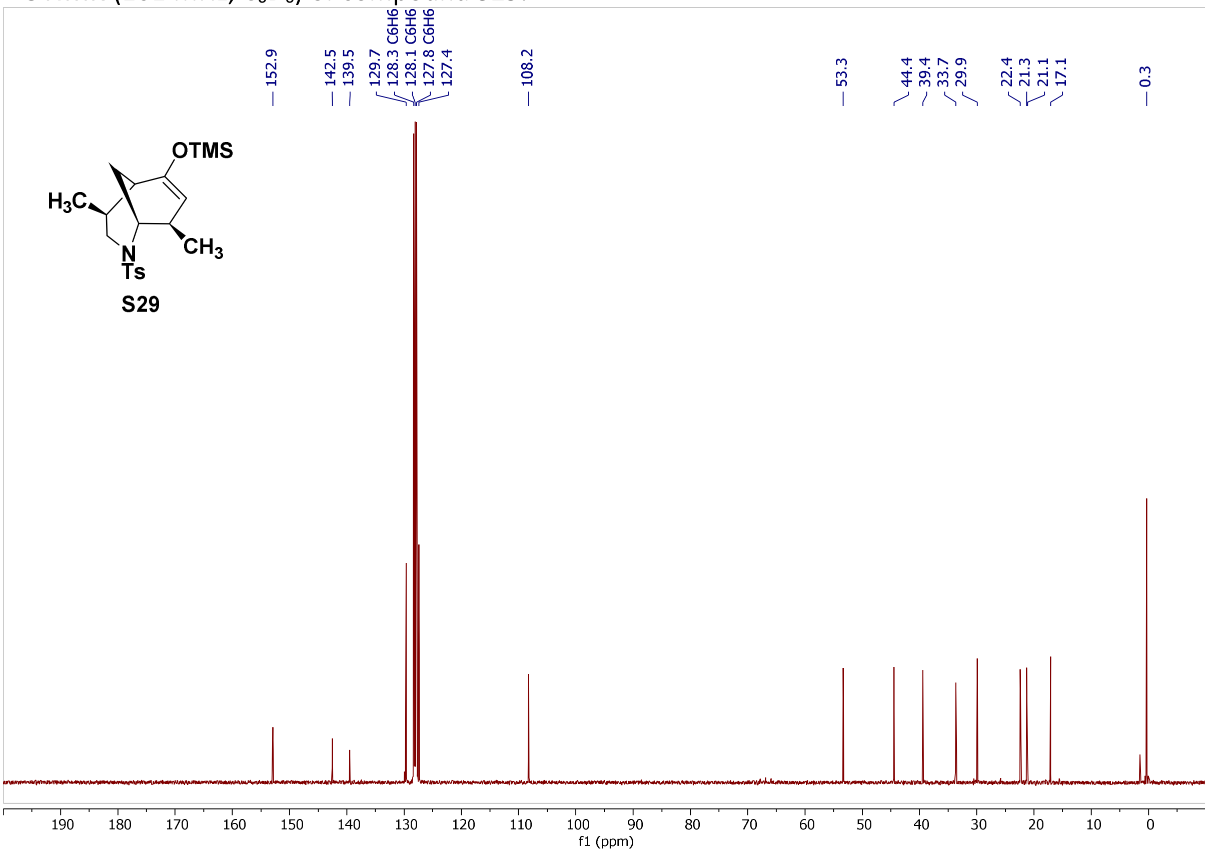

^{1}H NMR (400 MHz, $CDCl_3$) of crude product **S29.**

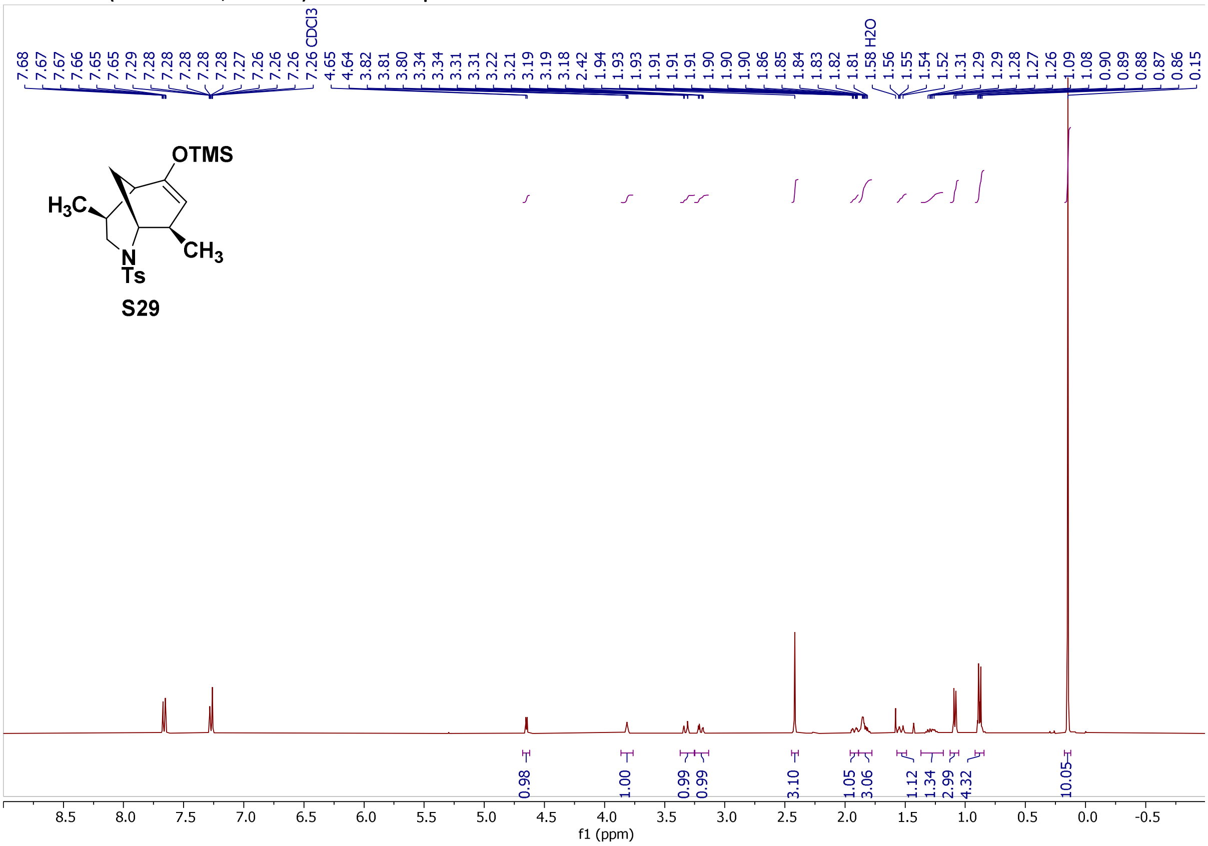

^{13}C NMR (101 MHz, $CDCl_3$) of crude product **S29.**

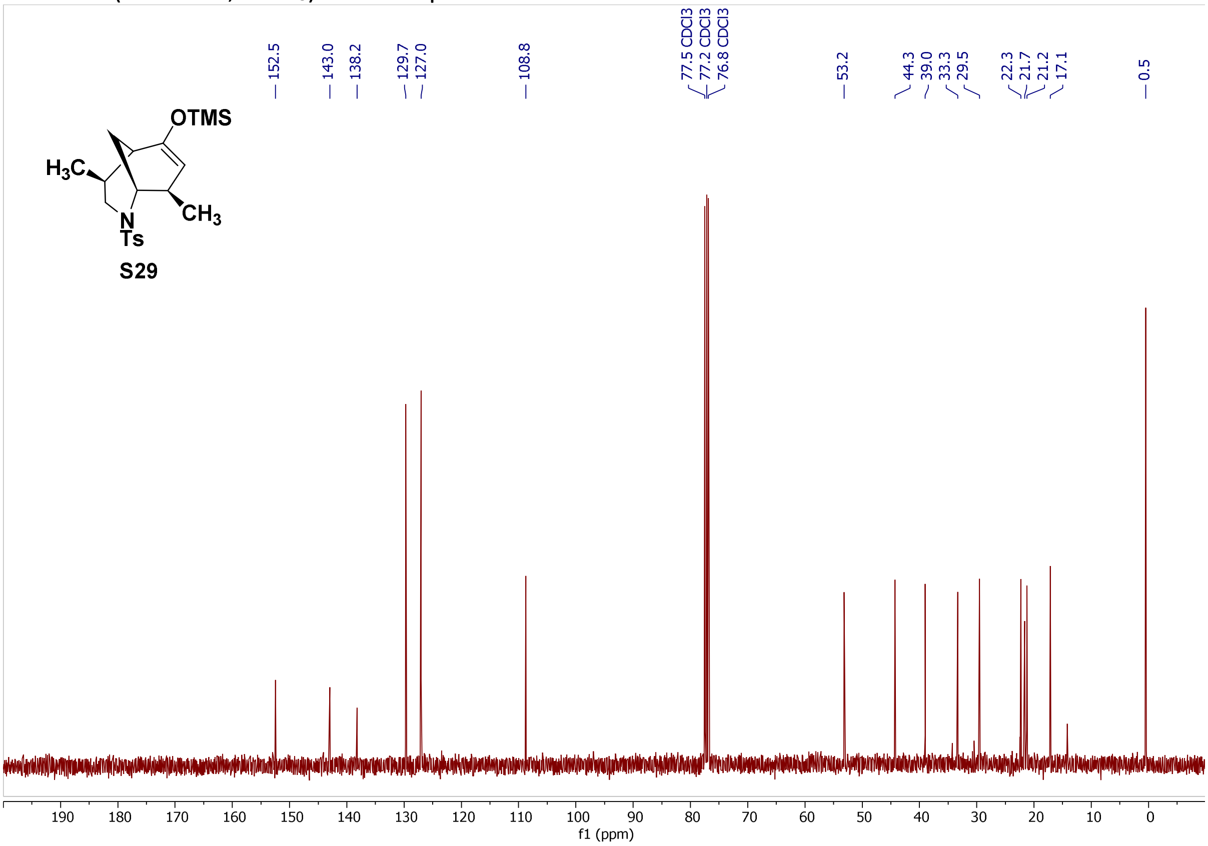

^{1}H NMR (400 MHz, C_6D_6) of compound **S34.**

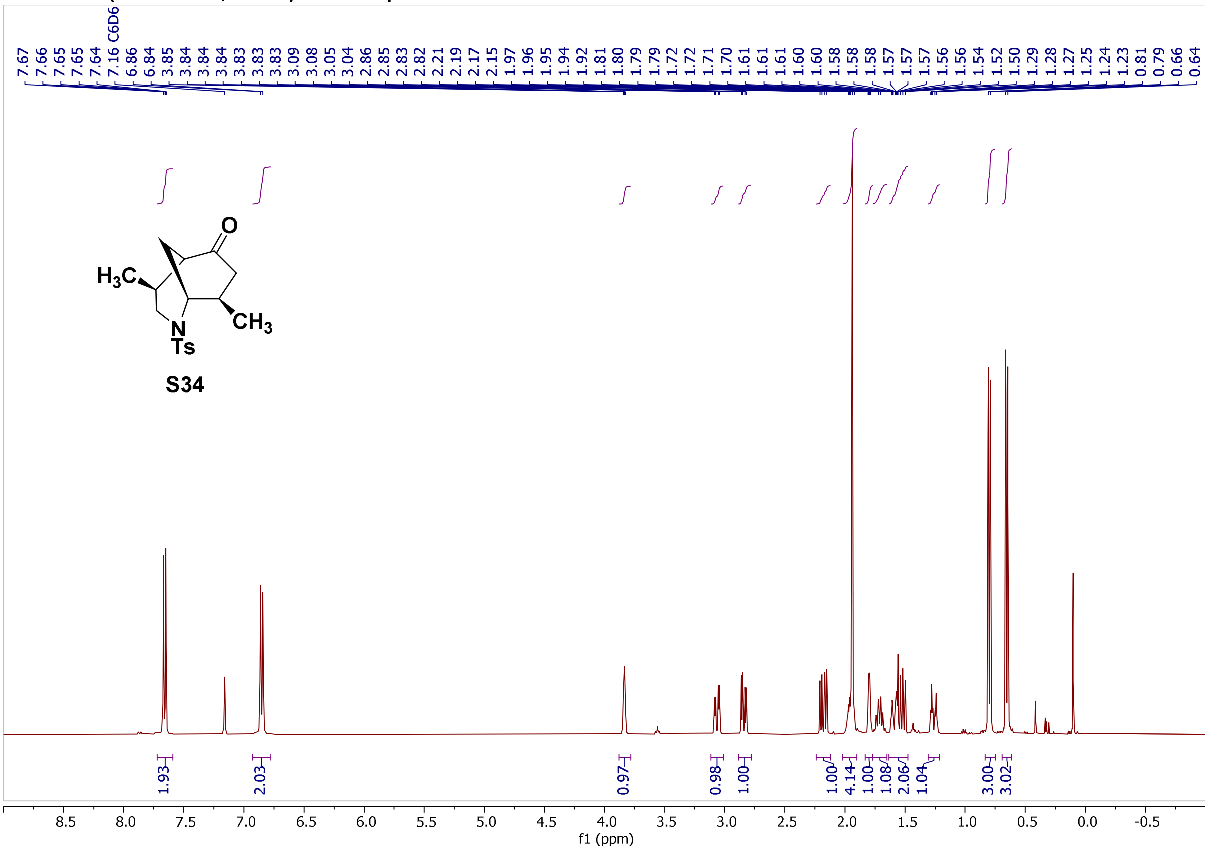

^{13}C NMR (101 MHz, C_6D_6) of compound **S34.**

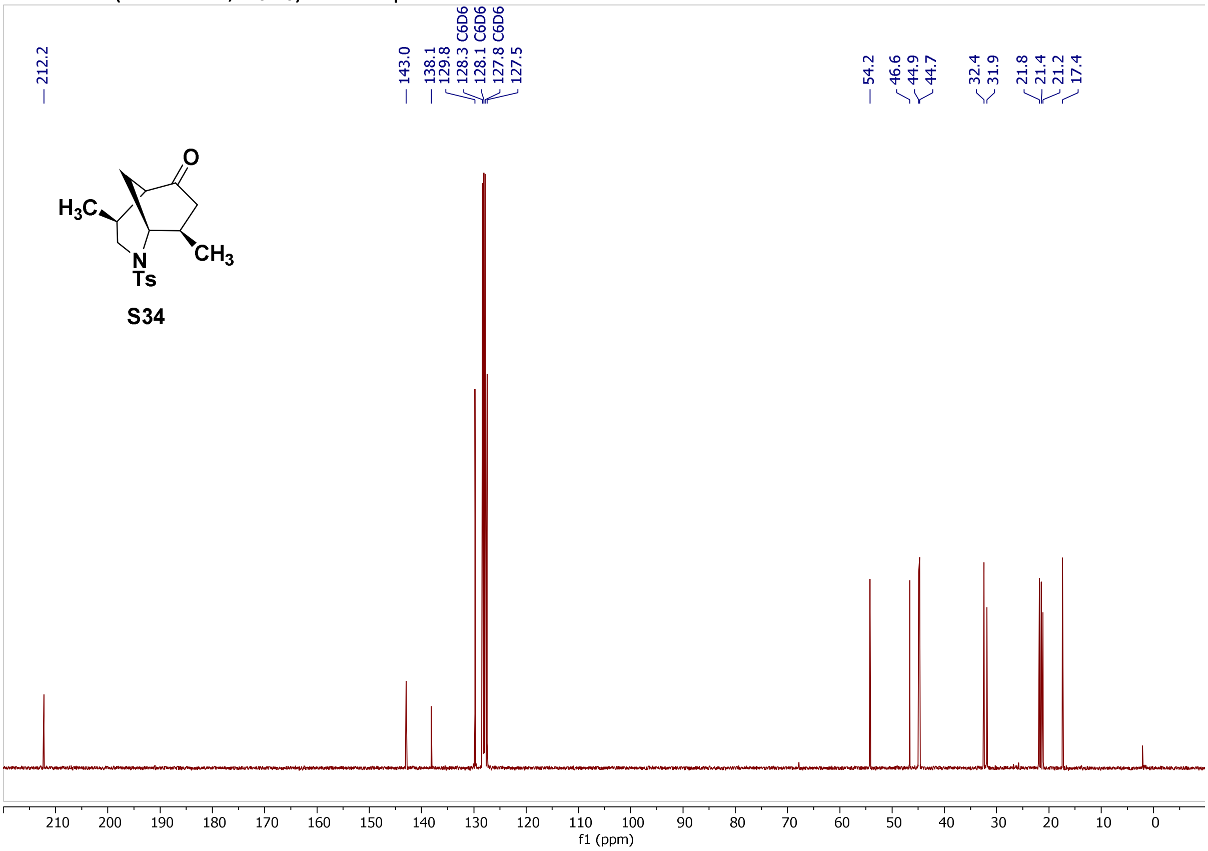

^{1}H NMR (400 MHz, $CDCl_3$) of compound **17.**

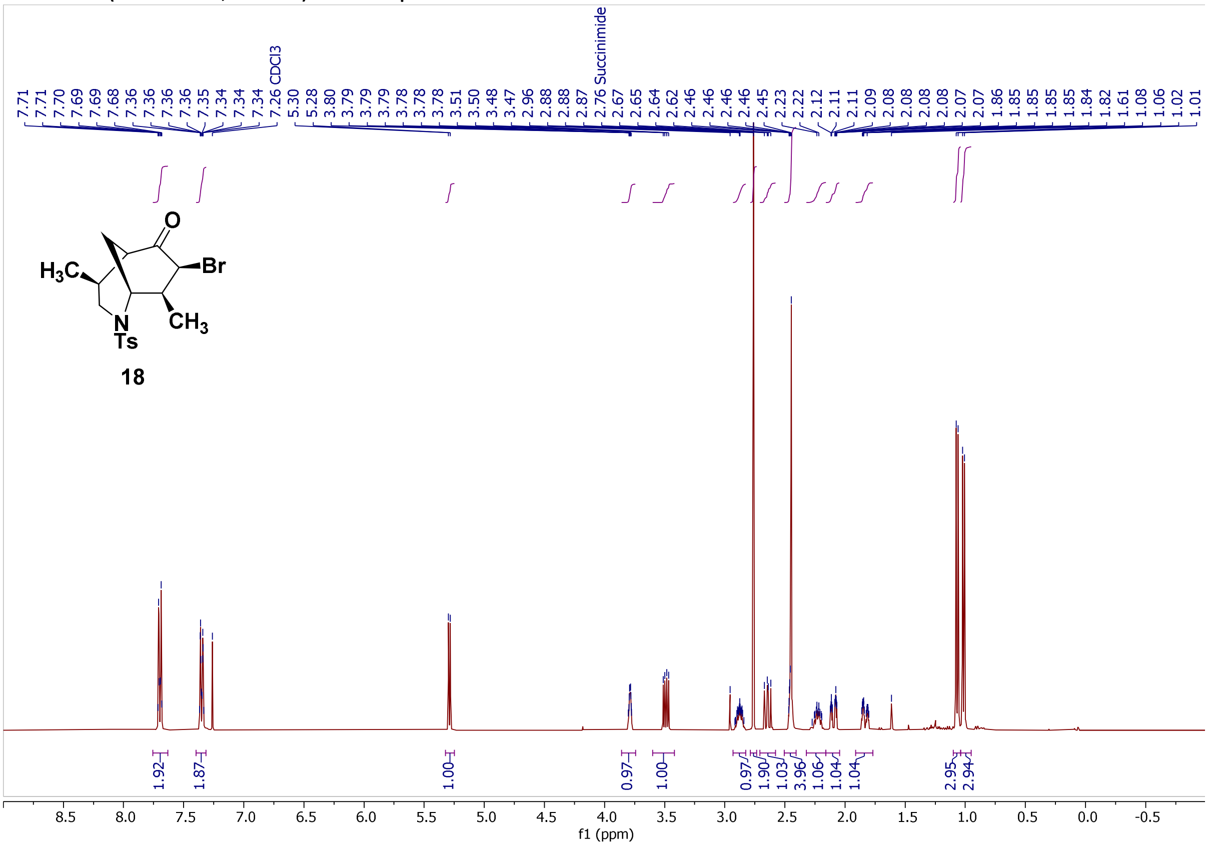

^{13}C NMR (101 MHz, $CDCl_3$) of compound **17.**

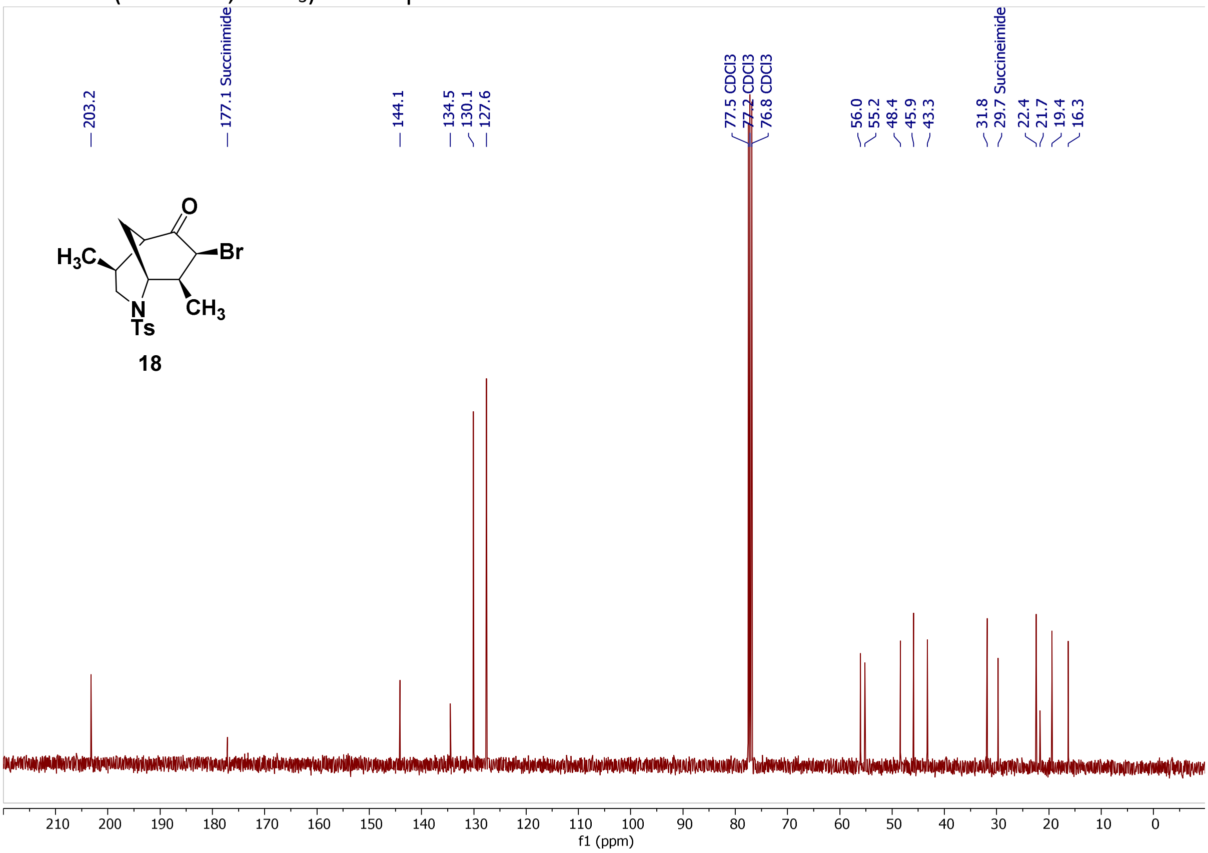

^{1}H NMR (400 MHz, $CDCl_3$) of compound **18.**

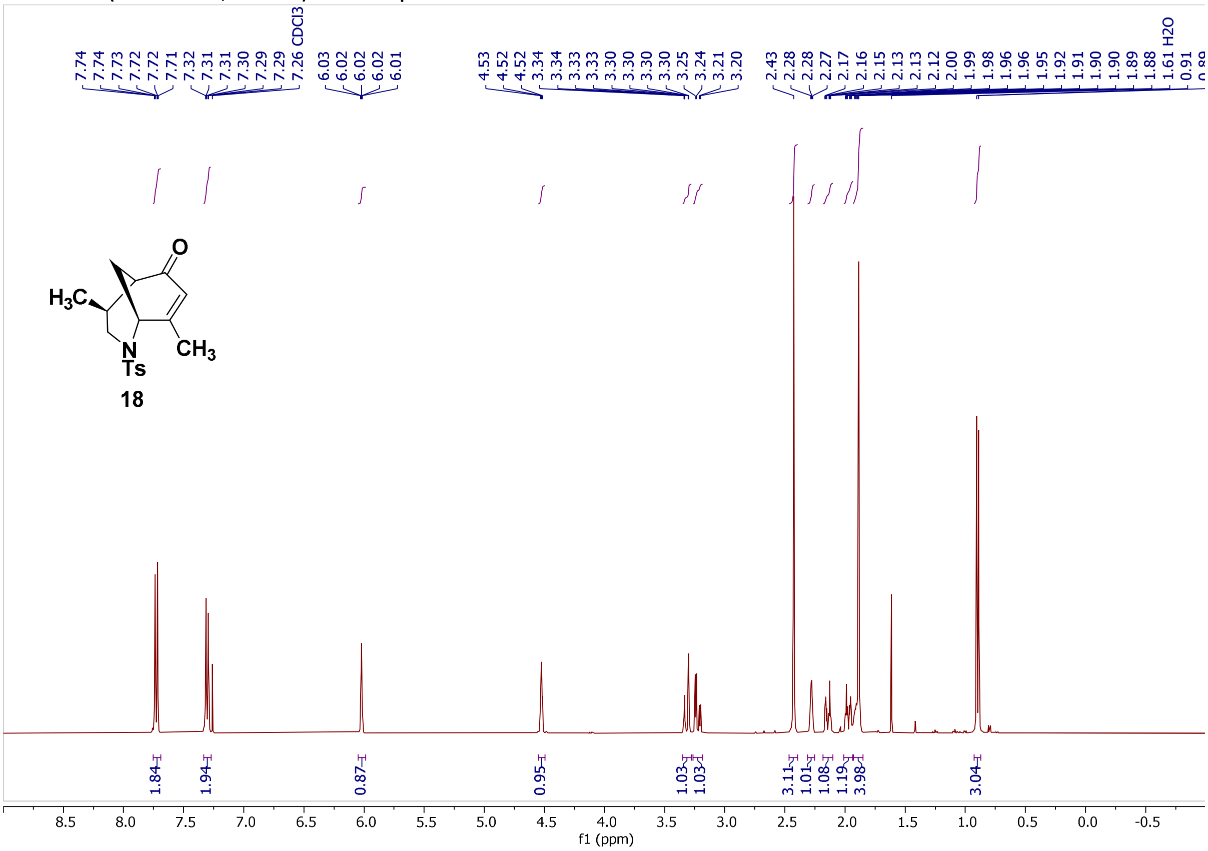

^{13}C NMR (101 MHz, $CDCl_3$) of compound **18.**

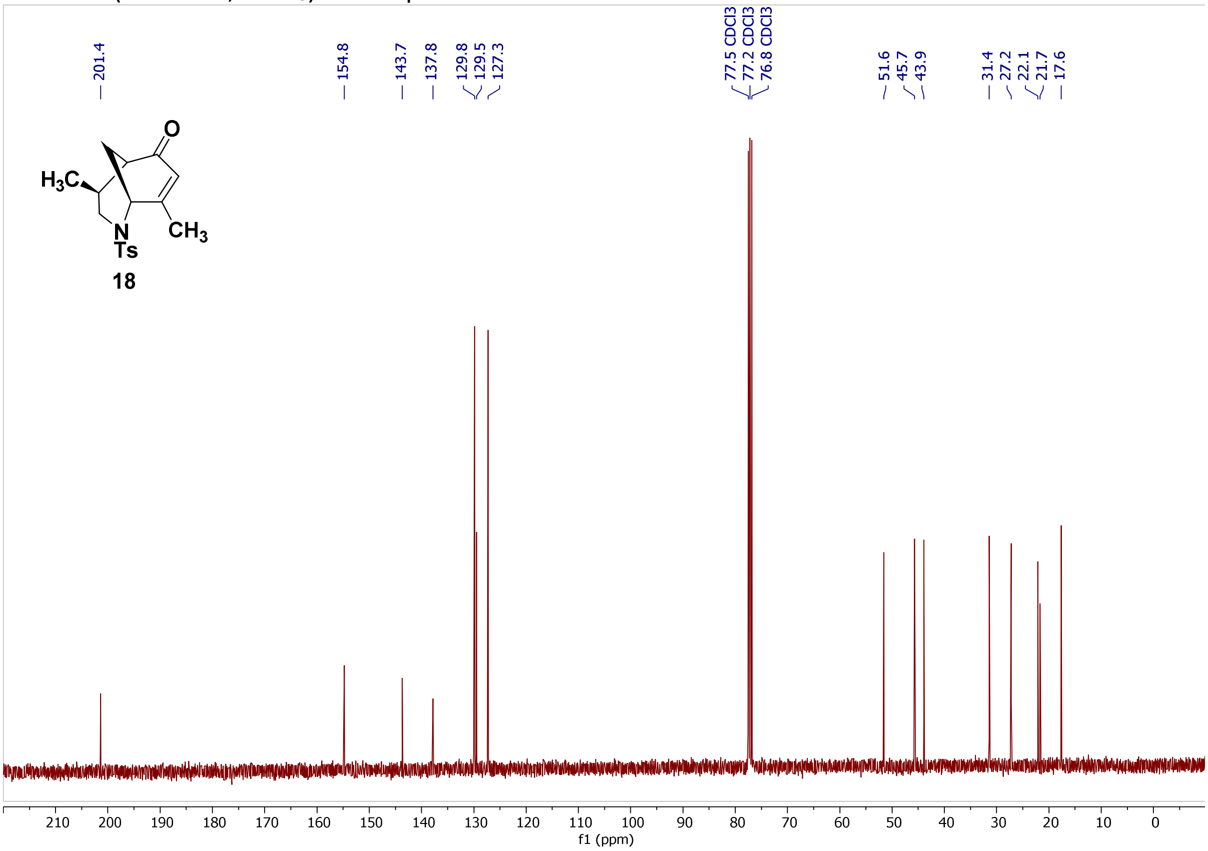

^{1}H NMR (400 MHz, $CDCl_3$) of compound **4.**

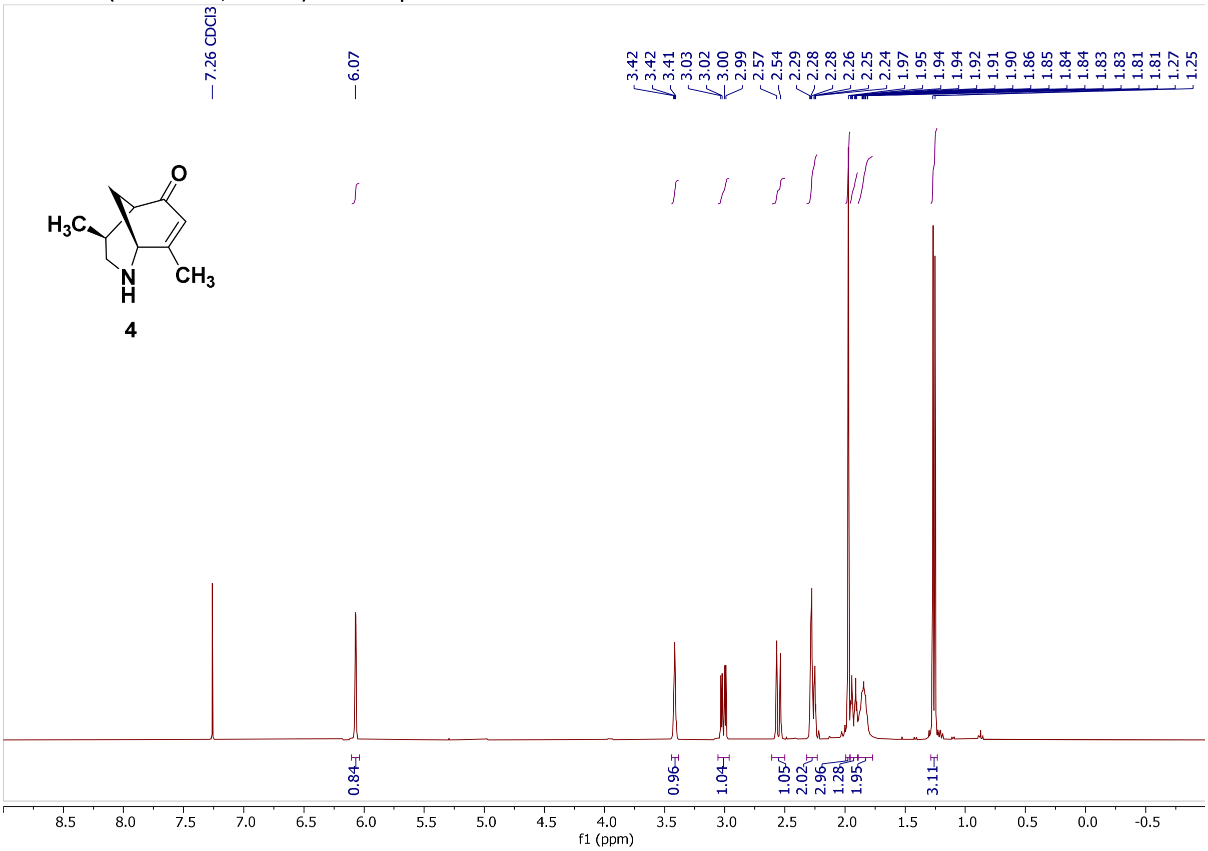

^{13}C NMR (101 MHz, $CDCl_3$) of compound **4.**

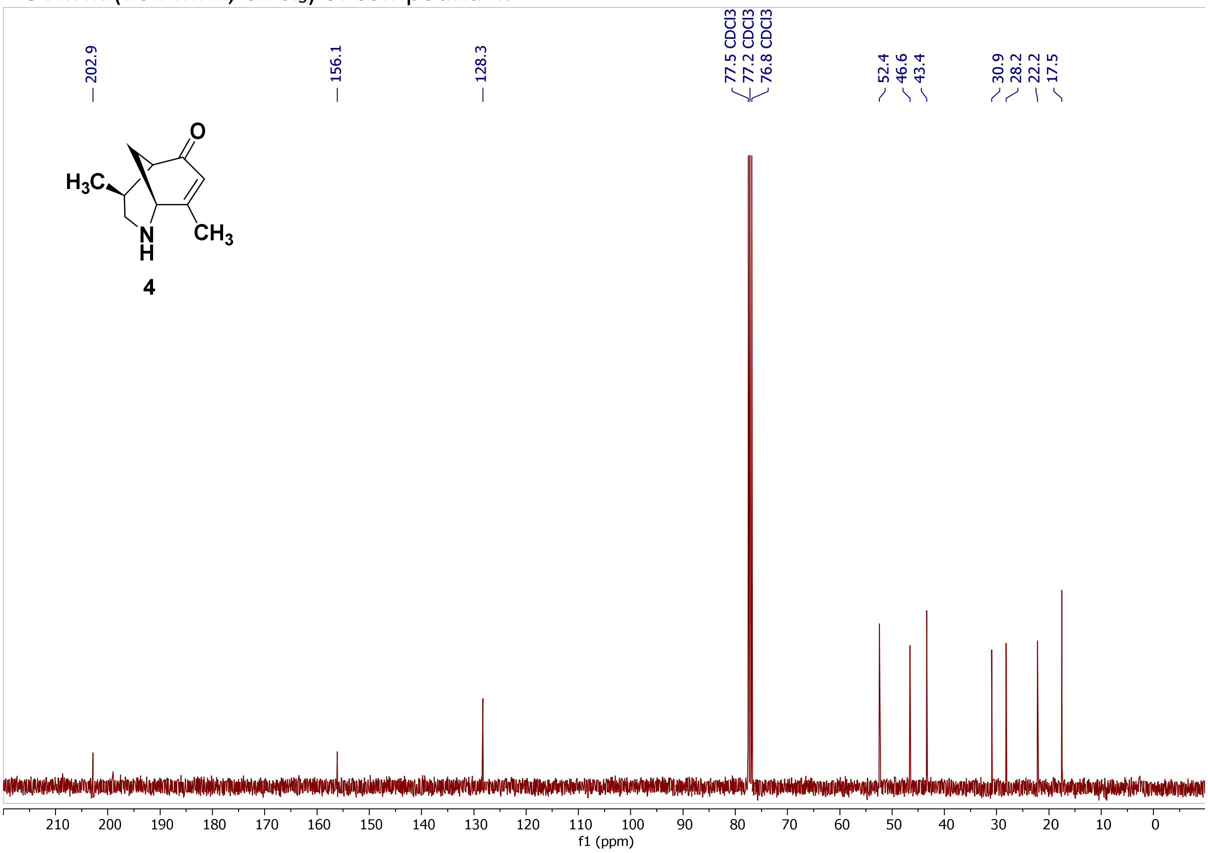

^{1}H NMR (500 MHz, $CDCl_3$) of compound **S35.**

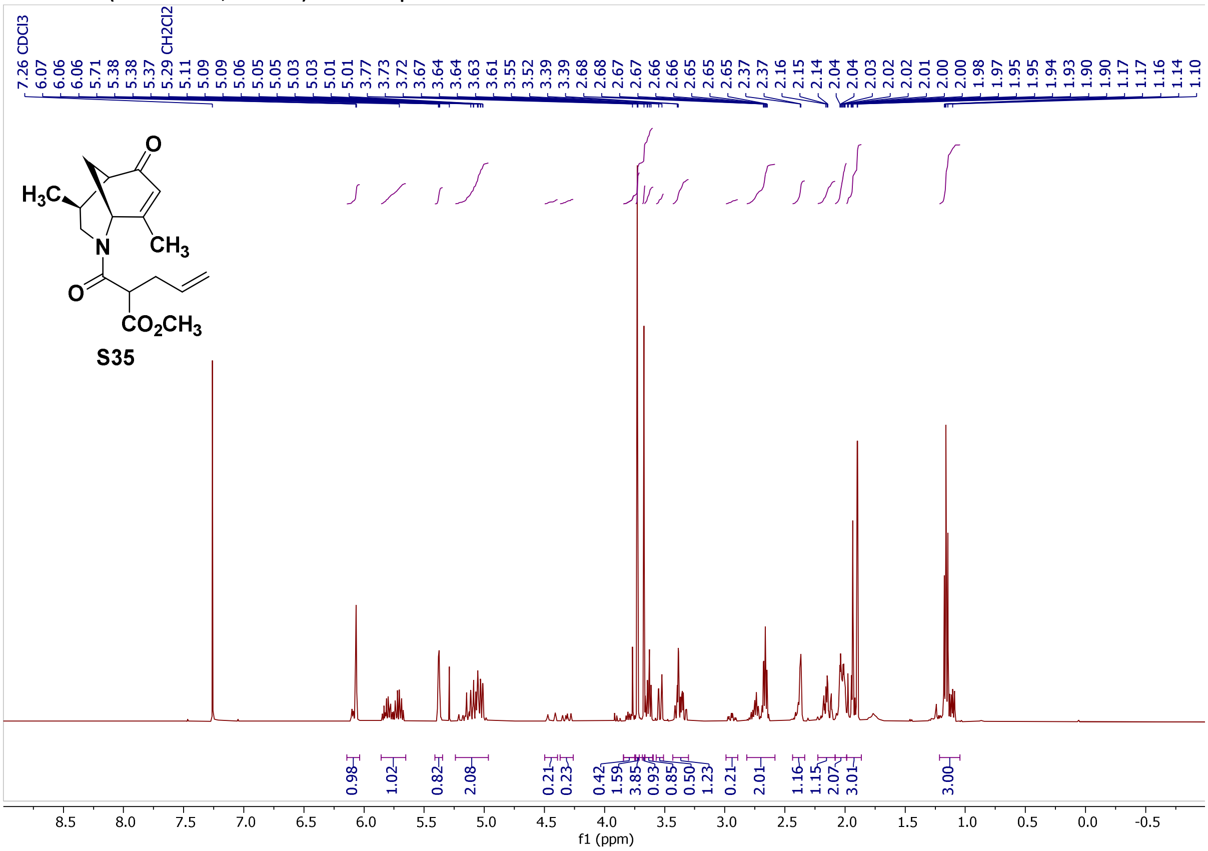

^{13}C NMR (126 MHz, $CDCl_3$) of compound **S35.**

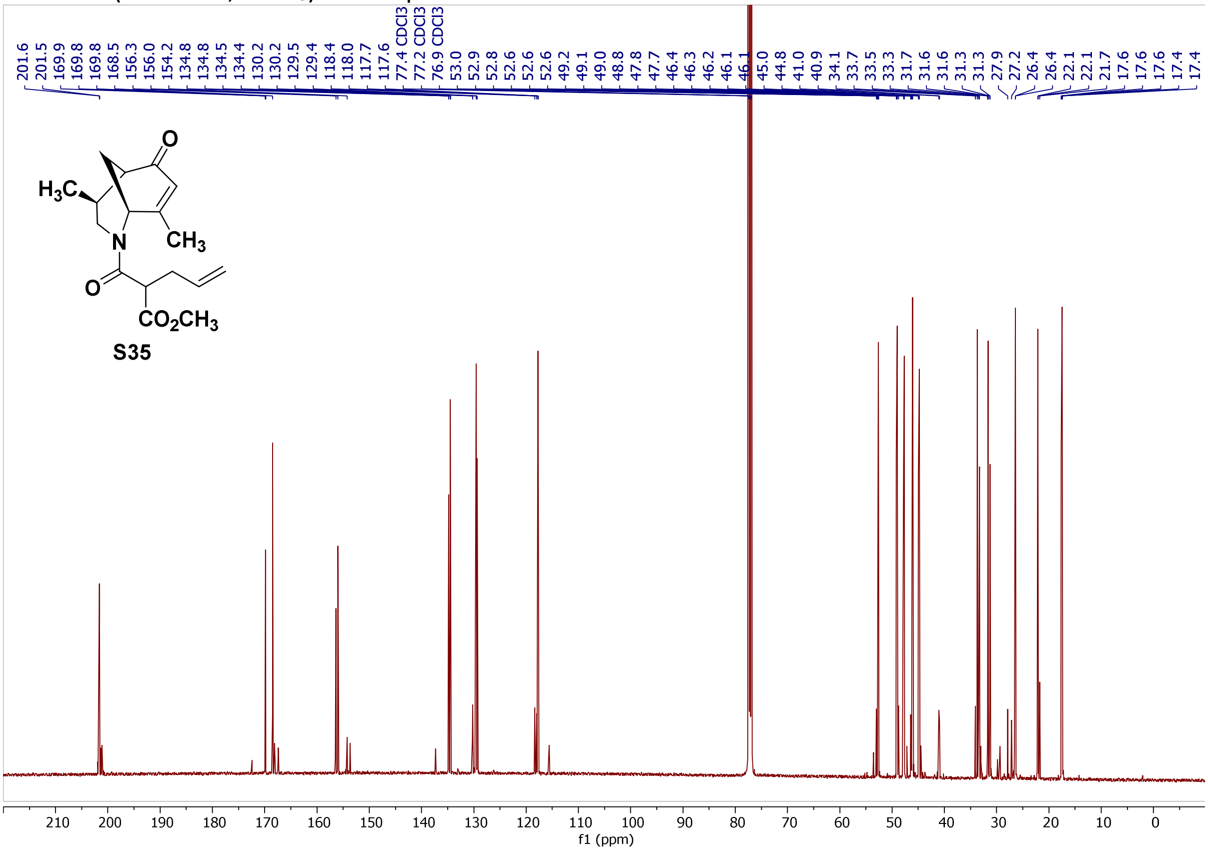

^{1}H **NMR** (400 MHz, $CDCl_3$) of compound **3.**

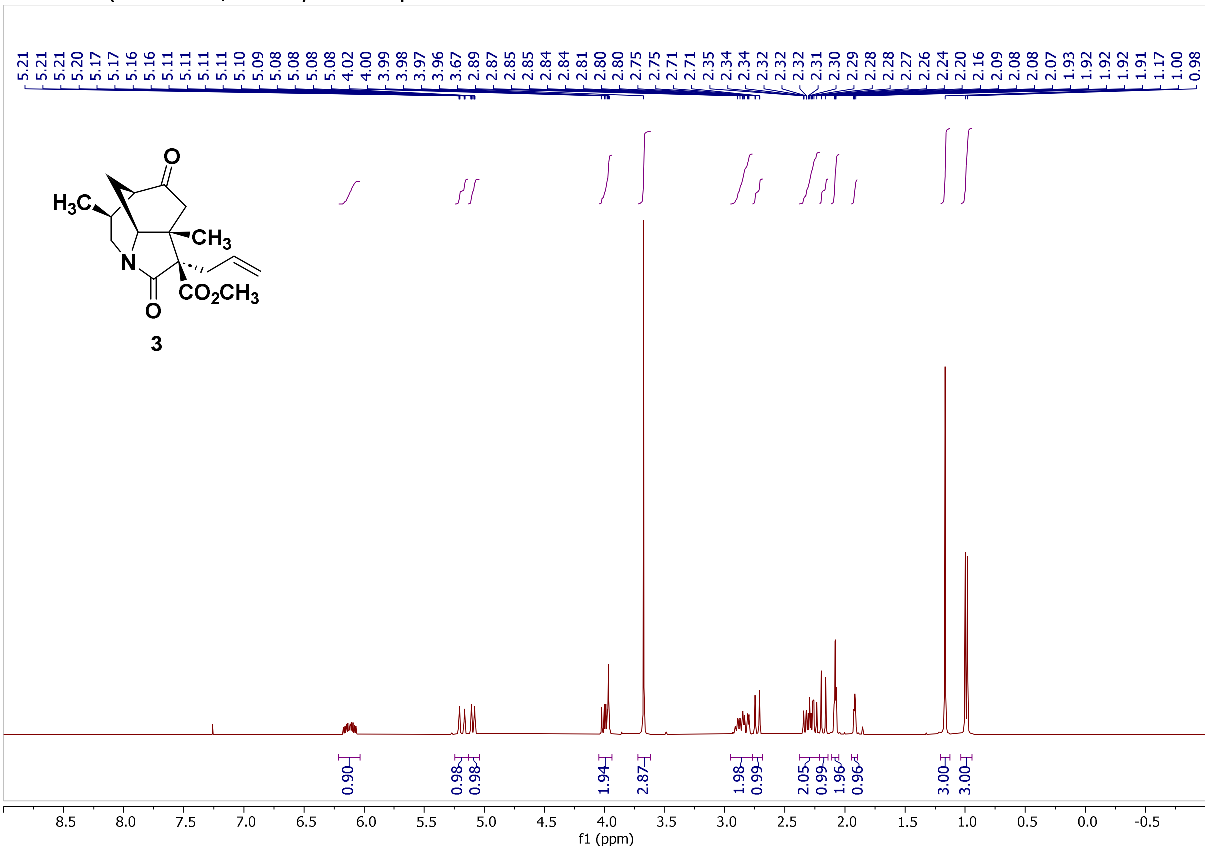

^{13}C **NMR** (101 MHz, $CDCl_3$) of compound **3.**

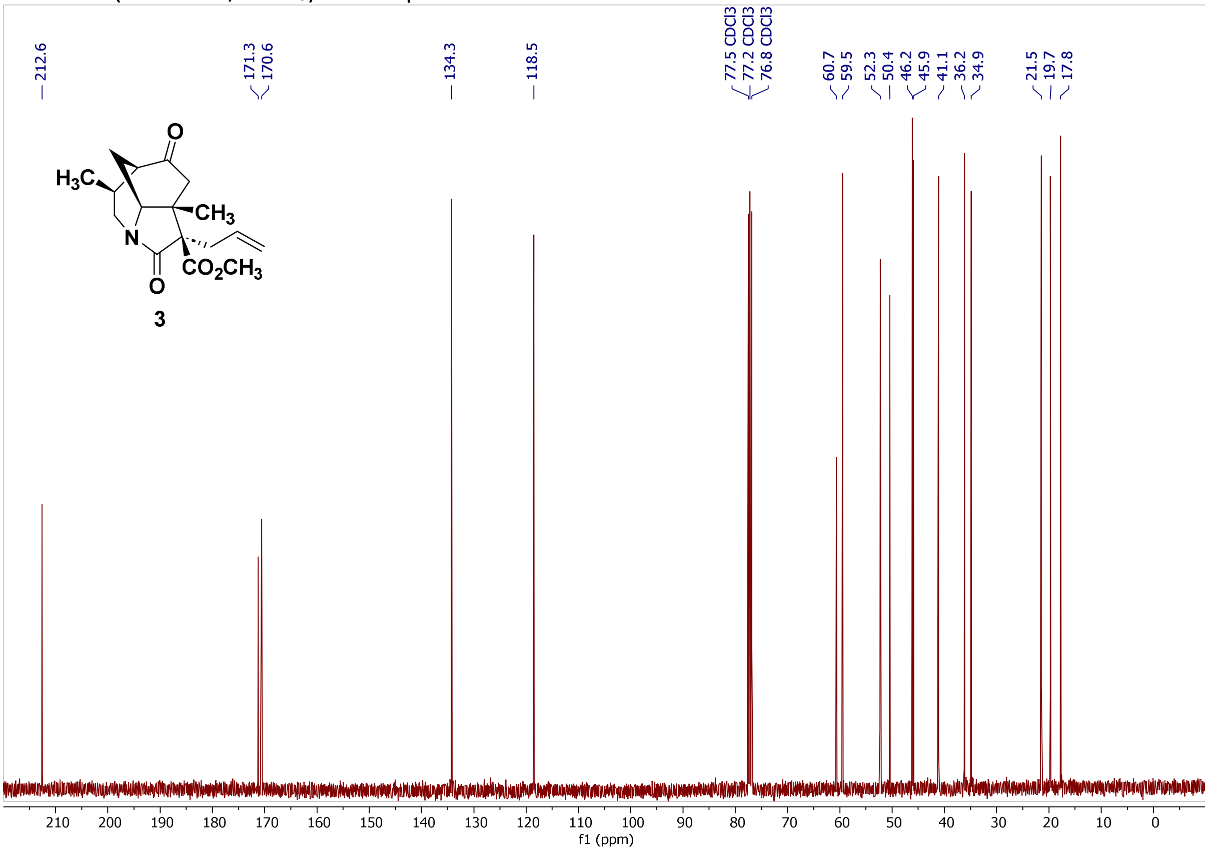

^{1}H NMR (400 MHz, $CDCl_3$) of compound **S36.**

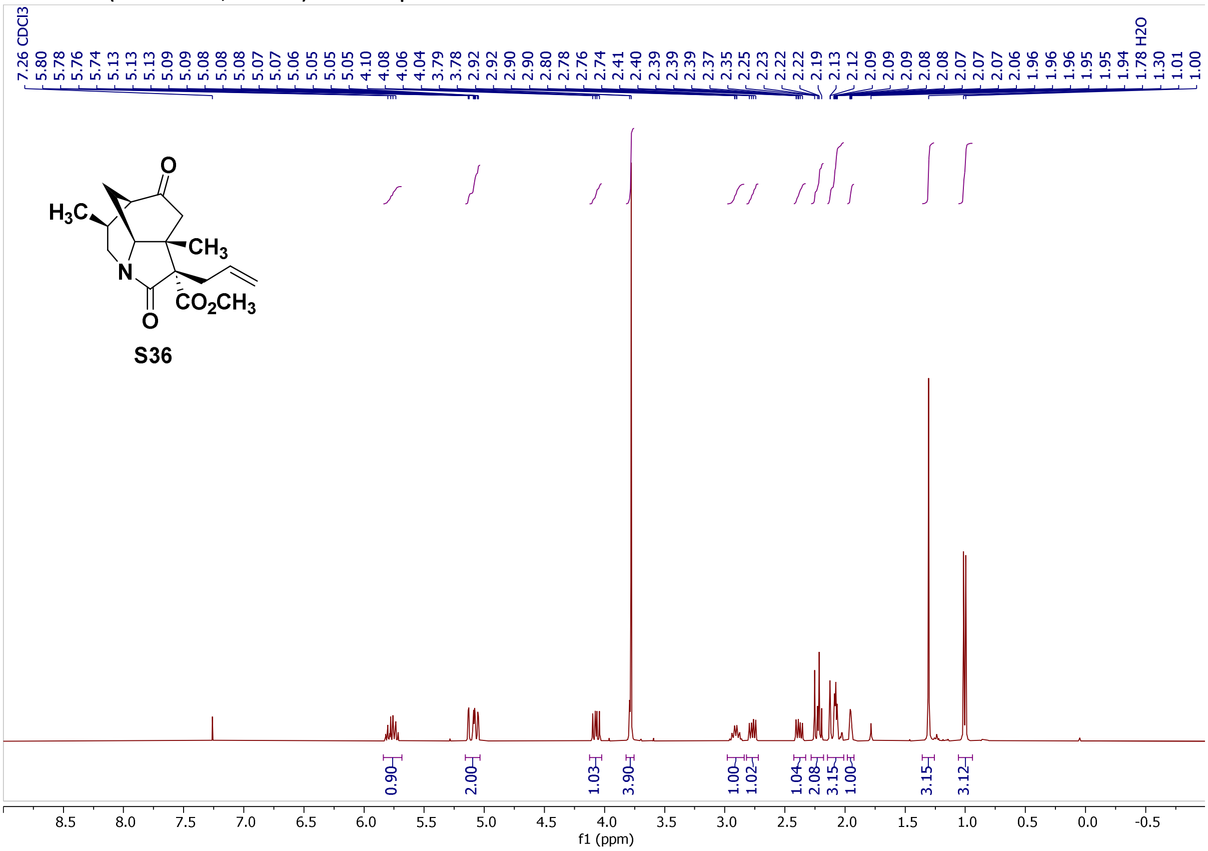

^{13}C NMR (101 MHz, $CDCl_3$) of compound **S36.**

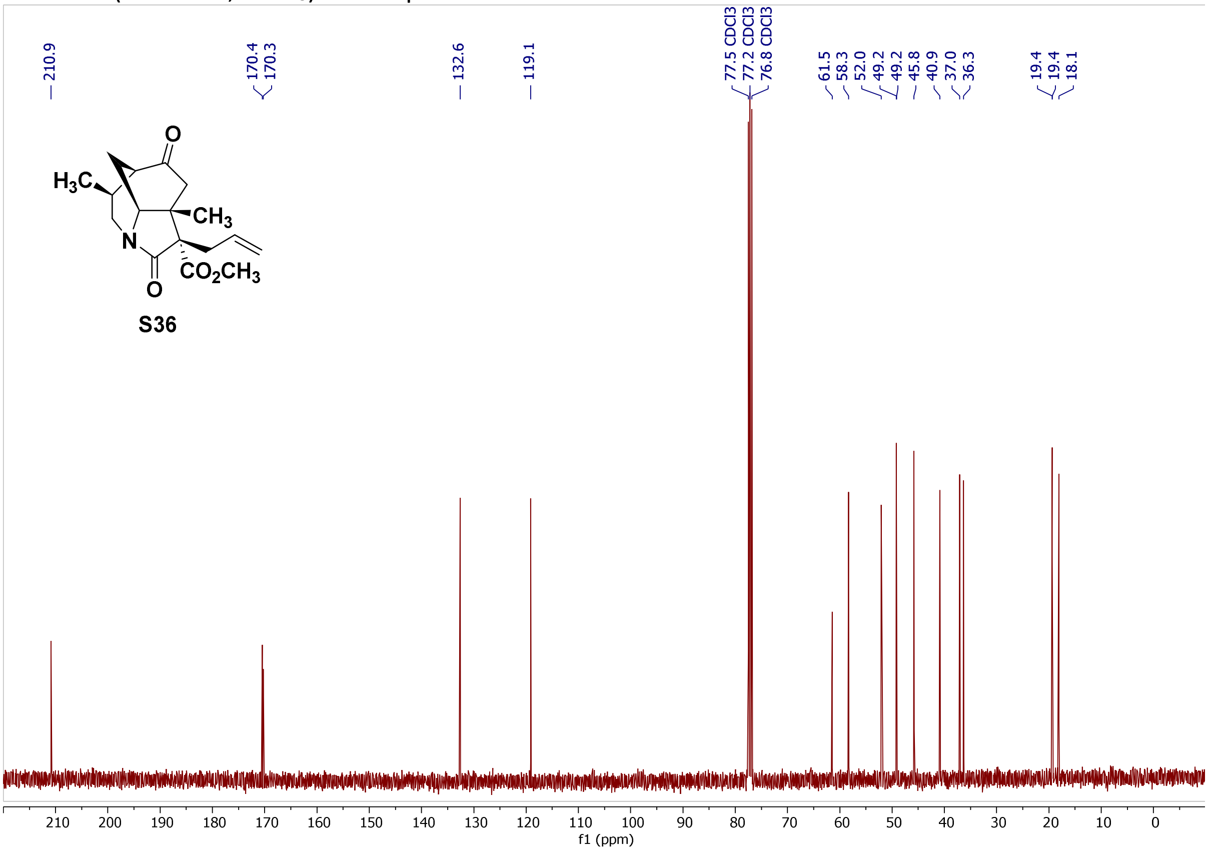

[1]**H NMR** (500 MHz, CDCl$_3$) of compound **S38.**

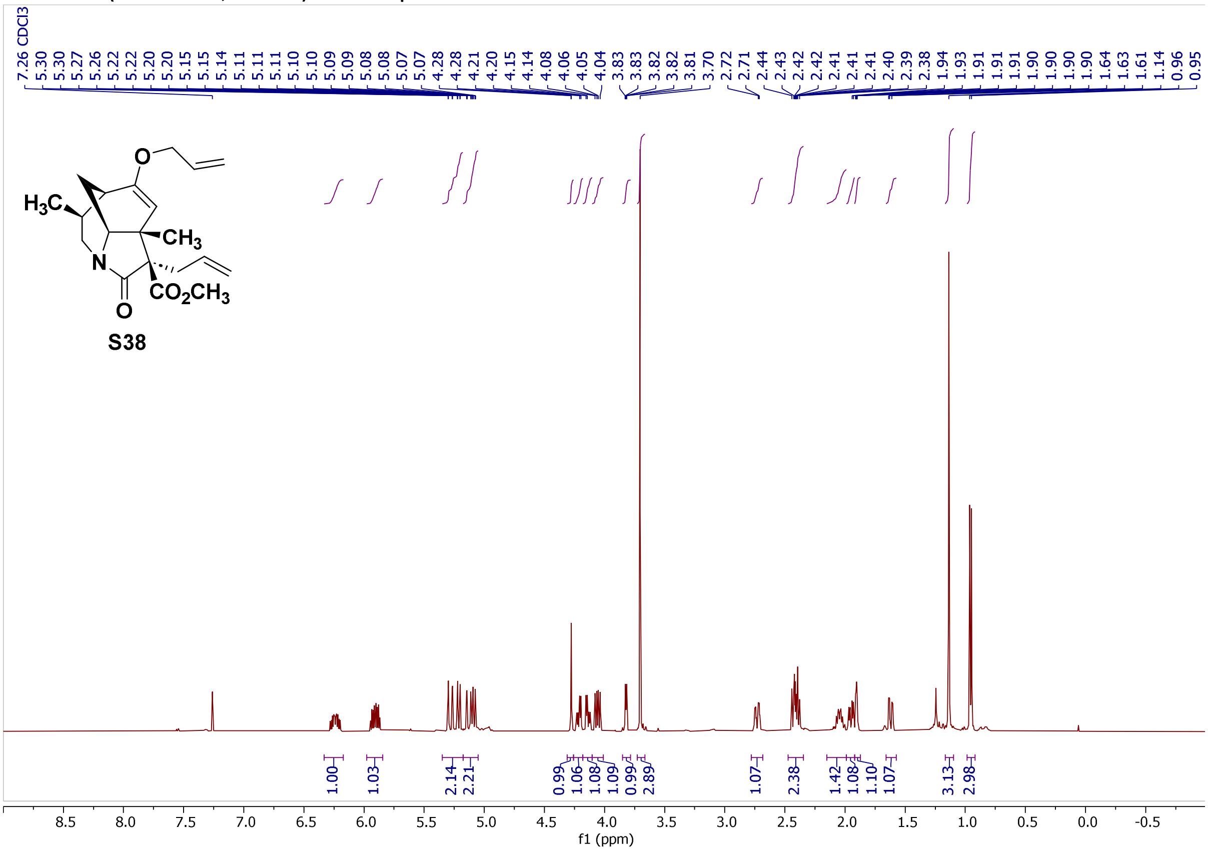

[13]**C NMR** (126 MHz, CDCl$_3$) of compound **S38.**

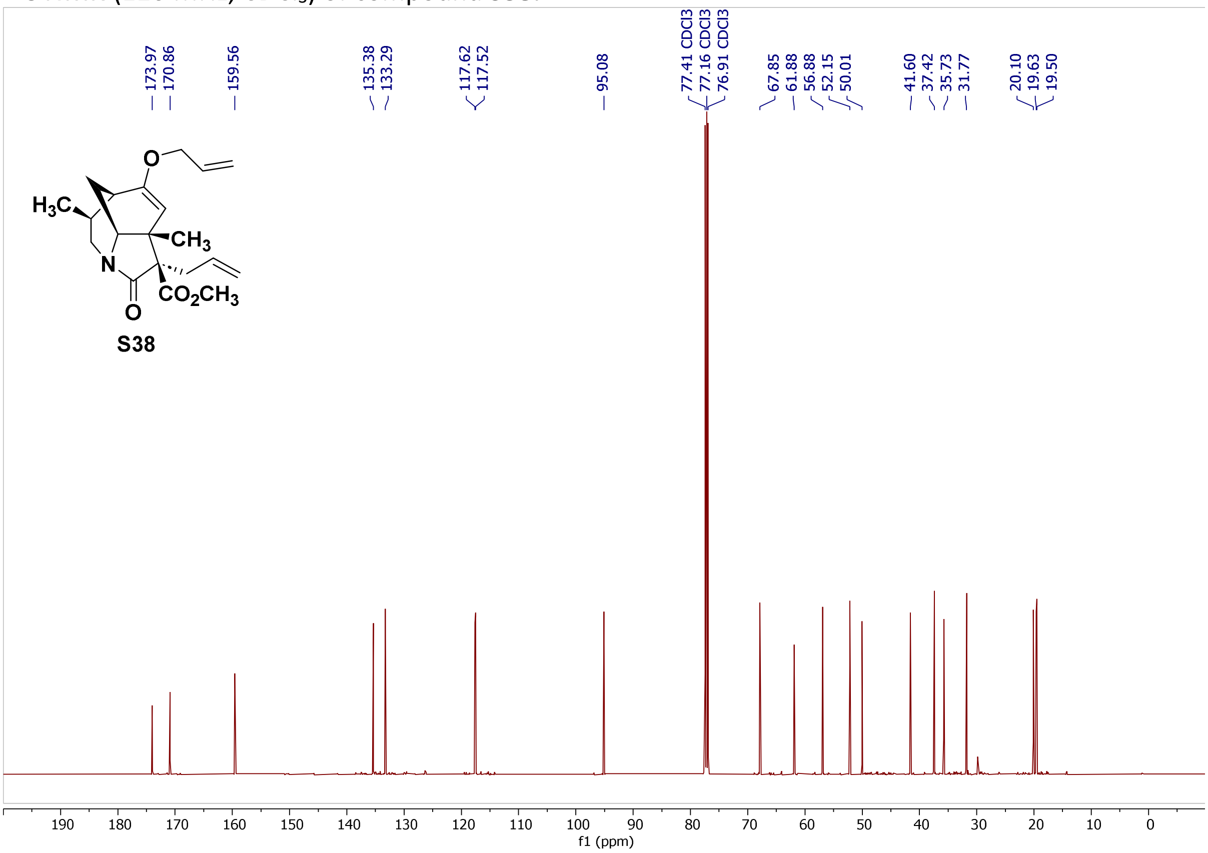

^{1}H NMR (400 MHz, $CDCl_3$) of compound **S39.**

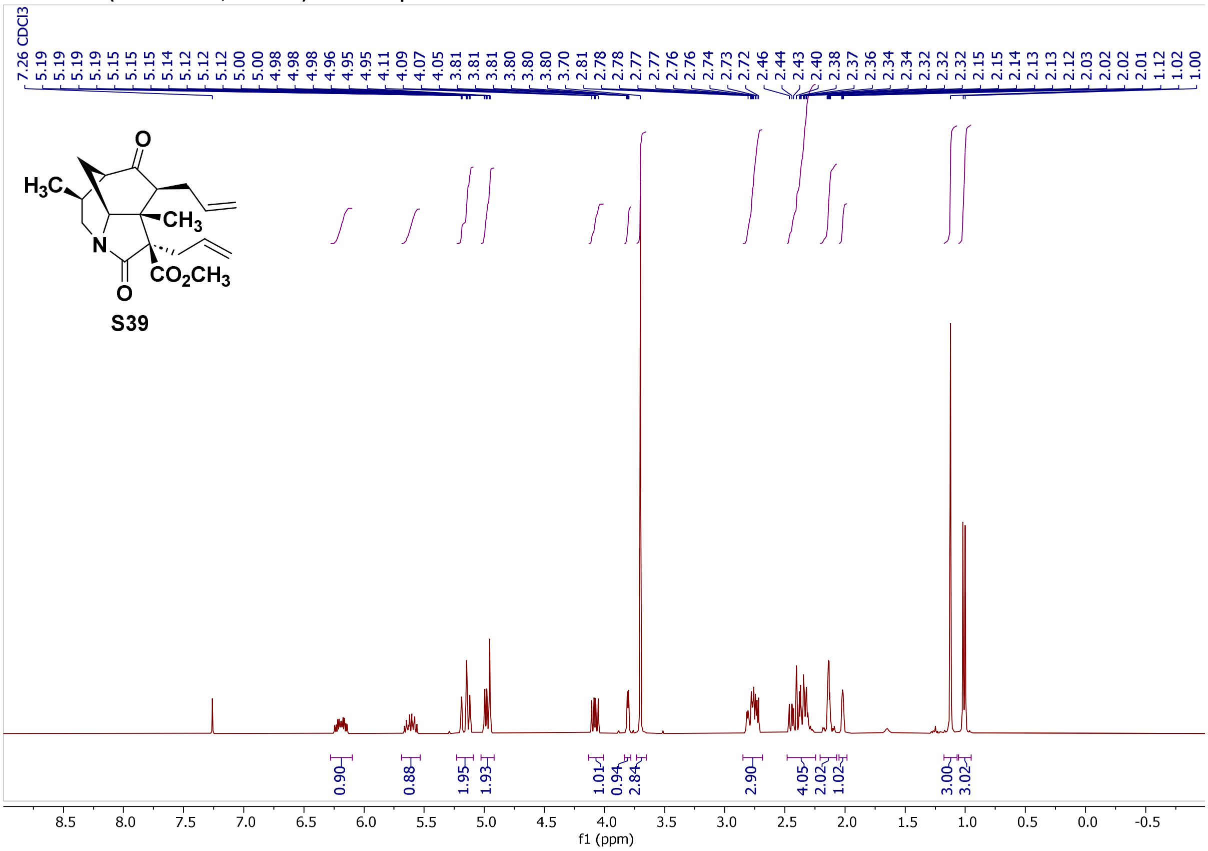

^{13}C NMR (101 MHz, $CDCl_3$) of compound **S39.**

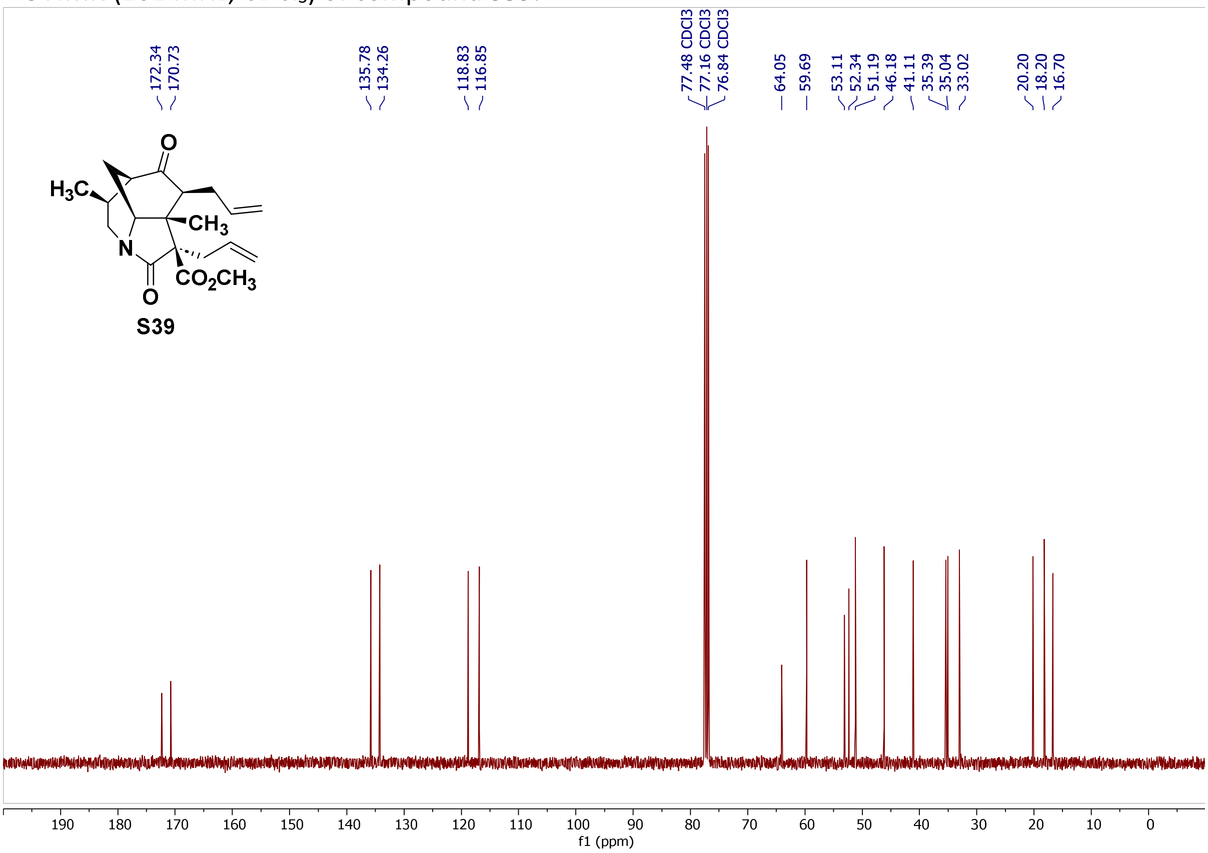

^{1}H NMR (500 MHz, $CDCl_3$) of compound **19.**

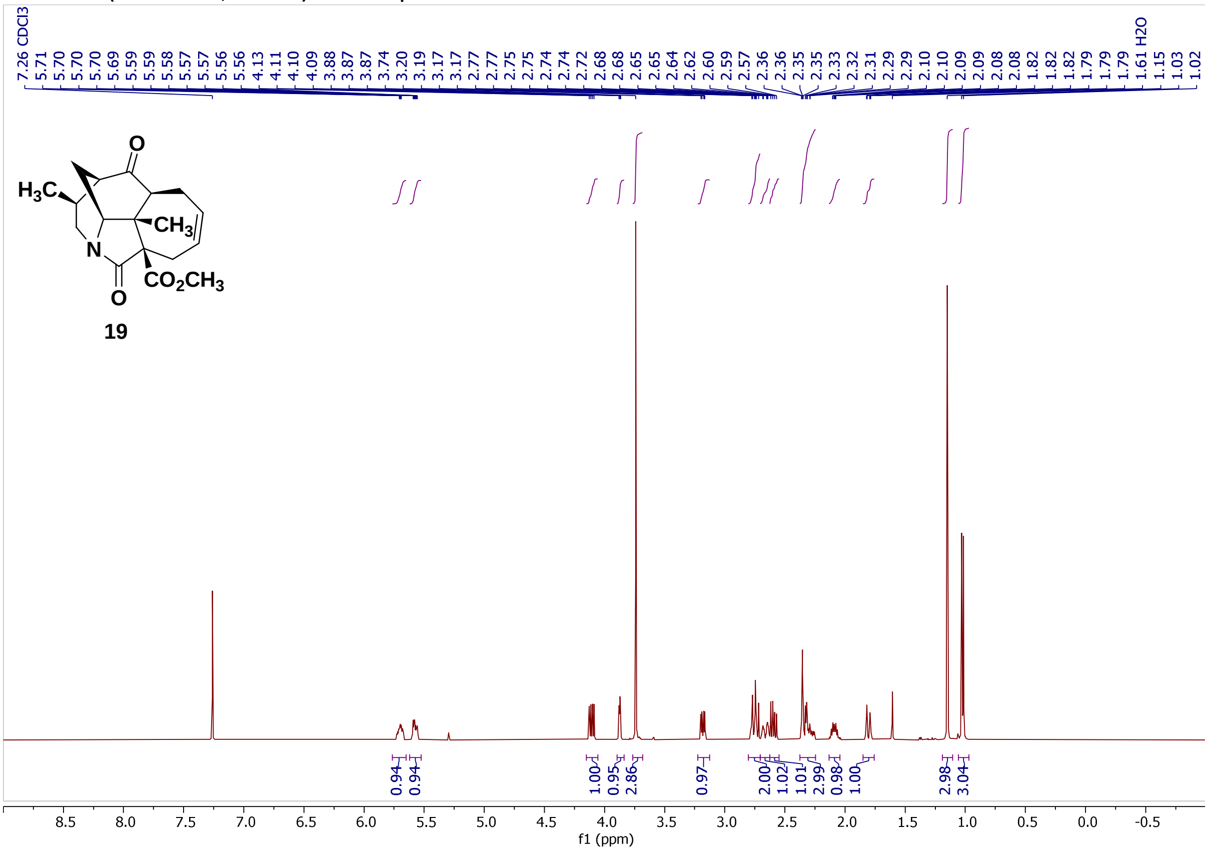

^{13}C NMR (126 MHz, $CDCl_3$) of compound **19.**

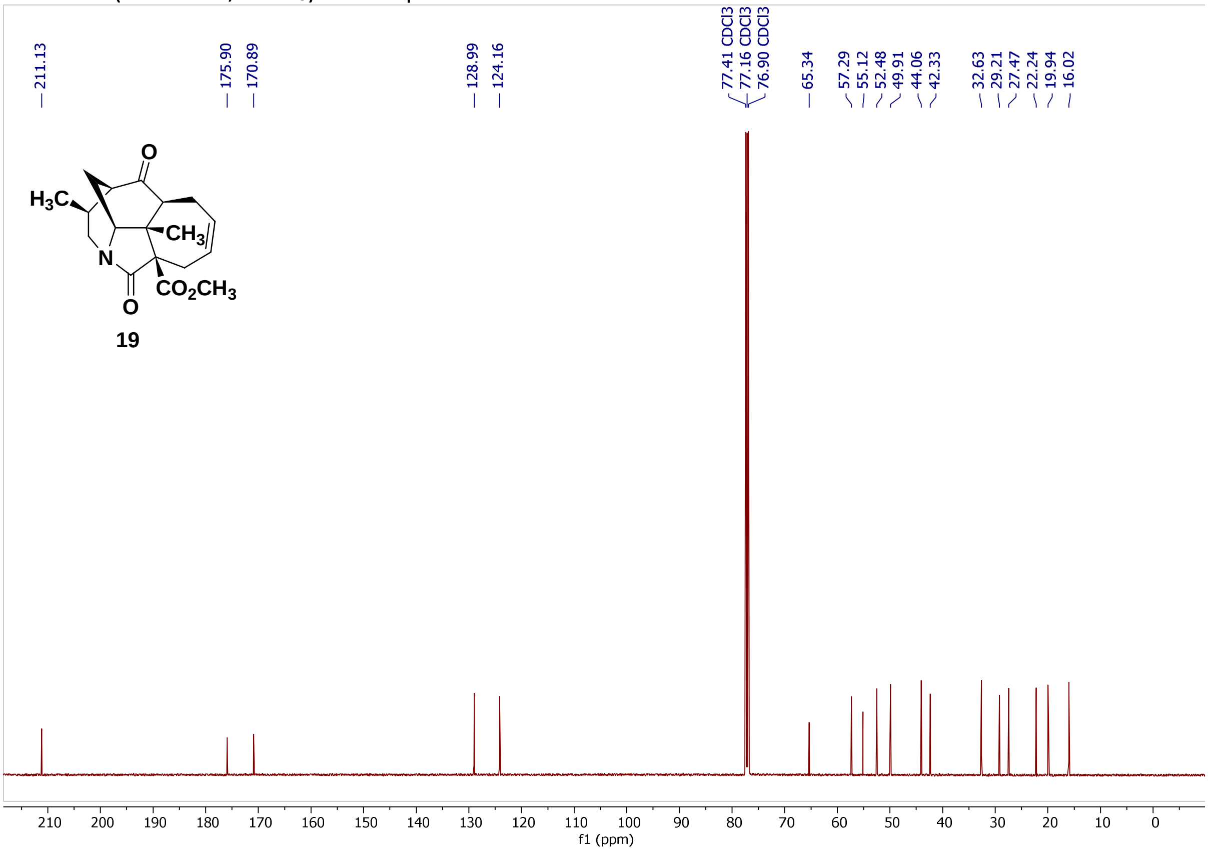

^{1}**H NMR** (500 MHz, $CDCl_3$) of compound **S40.**

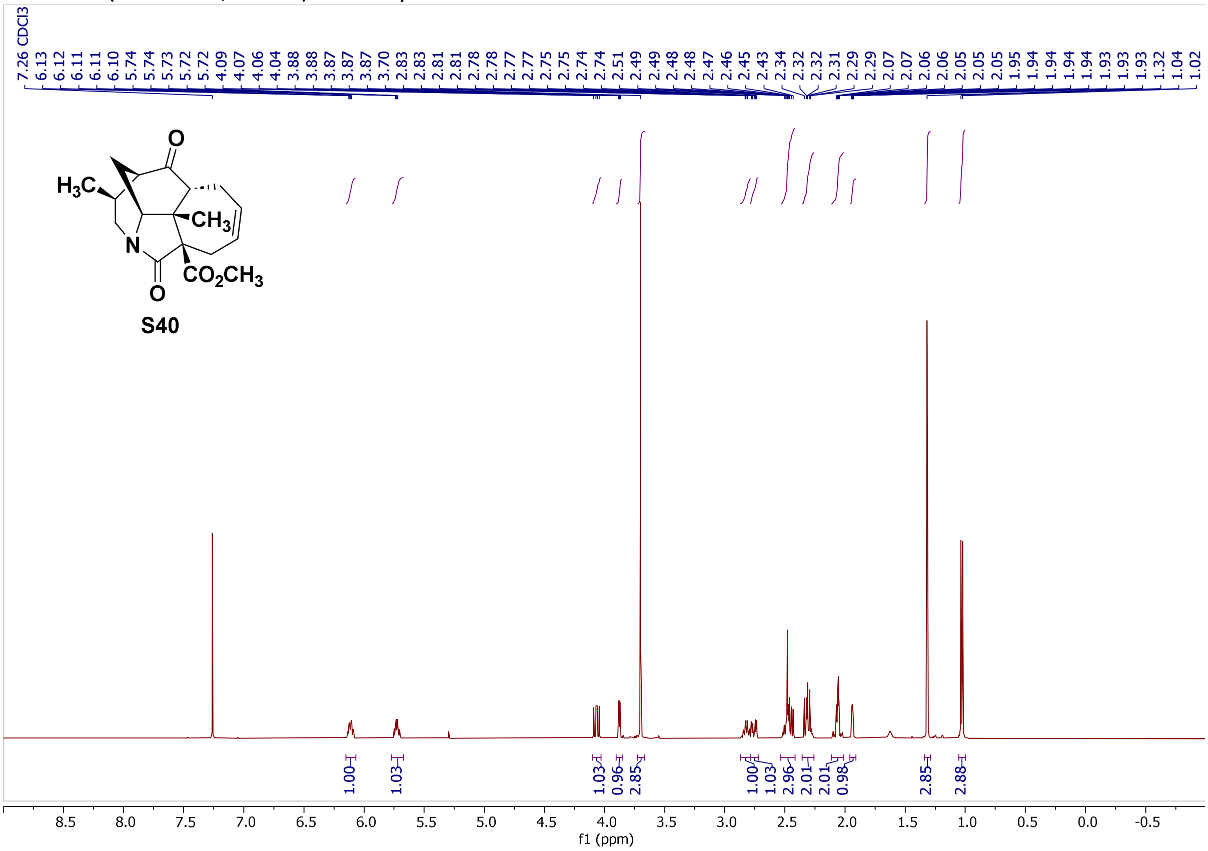

^{13}**C NMR** (101 MHz, $CDCl_3$) of compound **S40.**

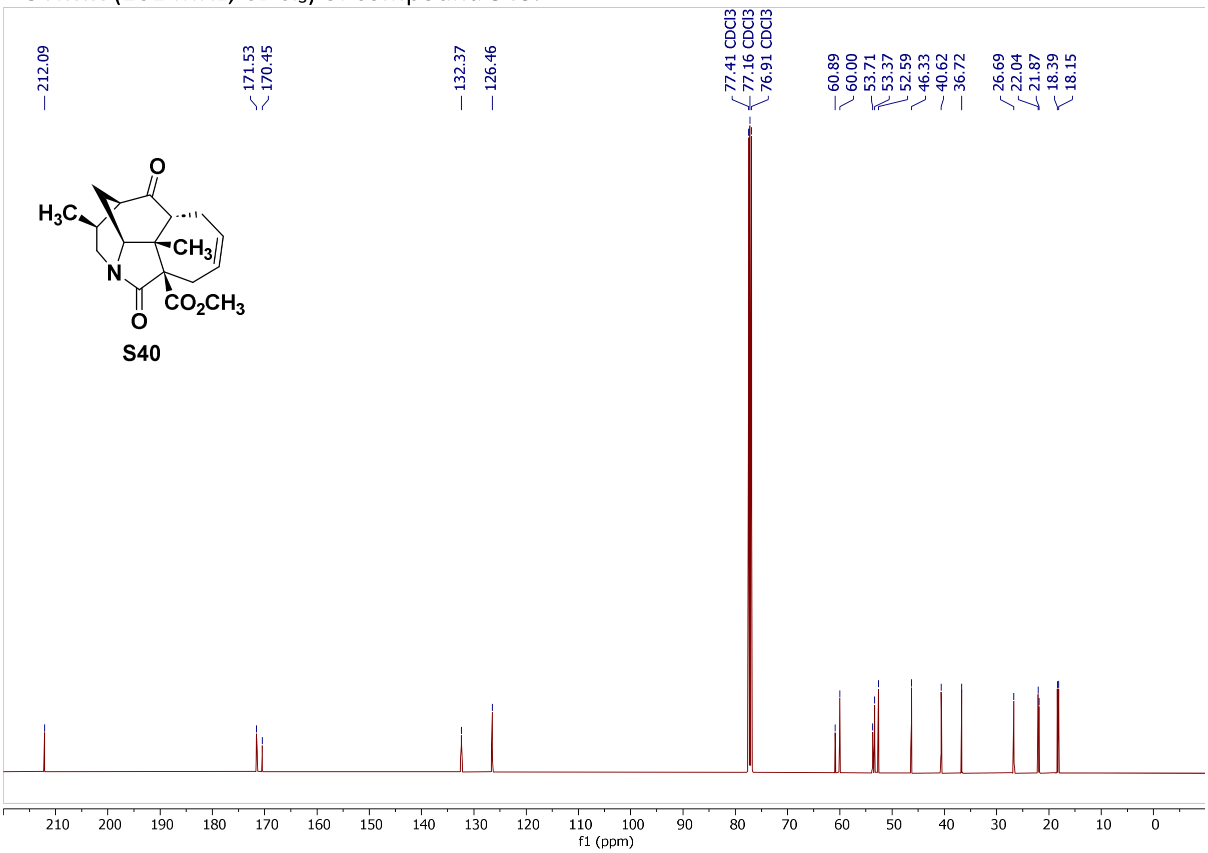

^{1}H NMR (400 MHz, $CDCl_3$) of compound **9.**

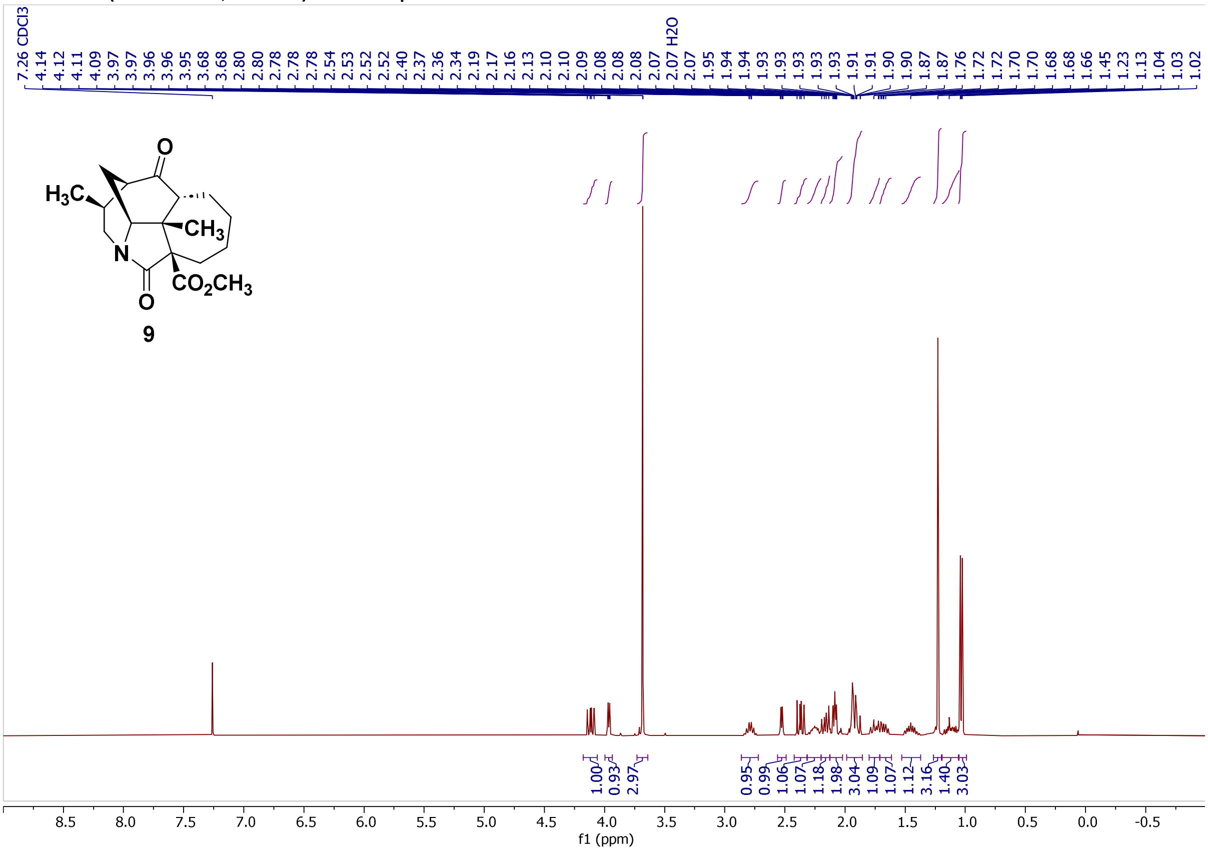

^{13}C NMR (101 MHz, $CDCl_3$) of compound **9.**

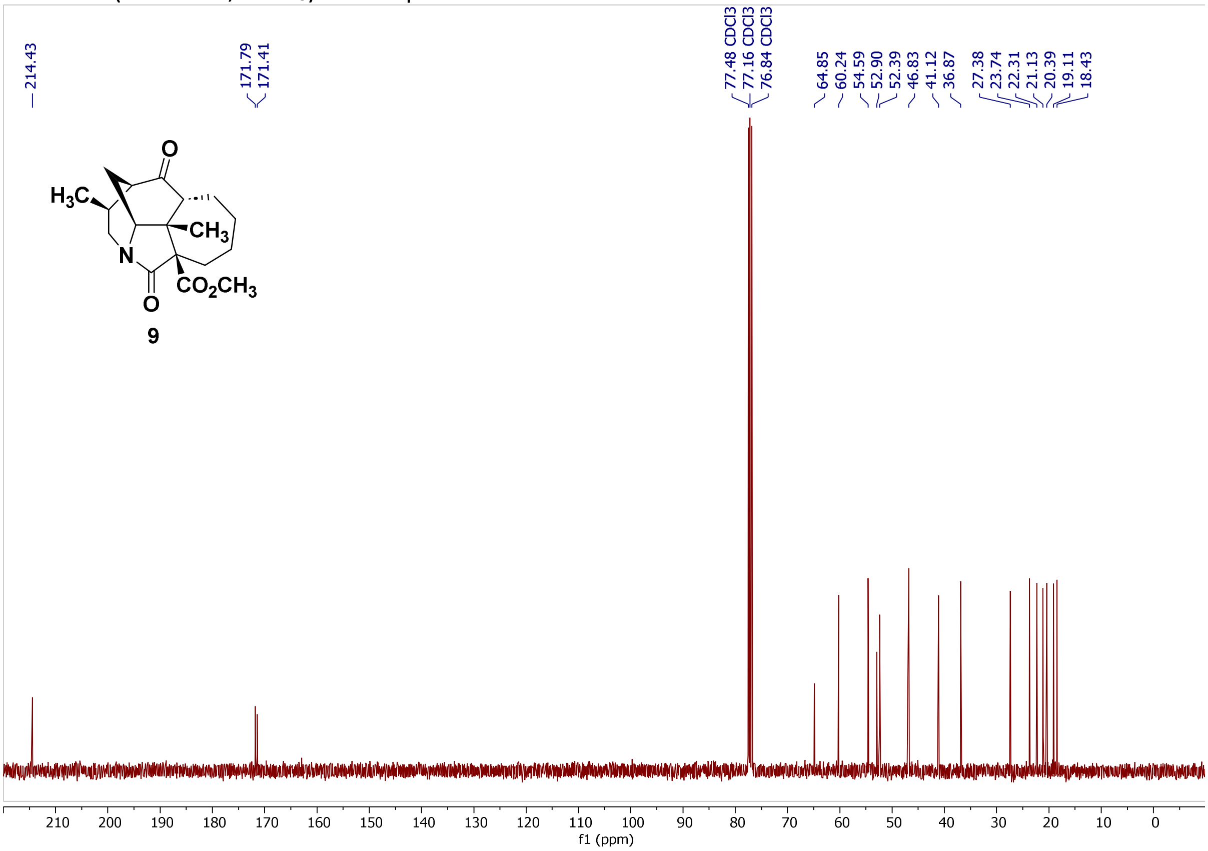

^{1}H NMR (400 MHz, $CDCl_3$) of compound **20.**

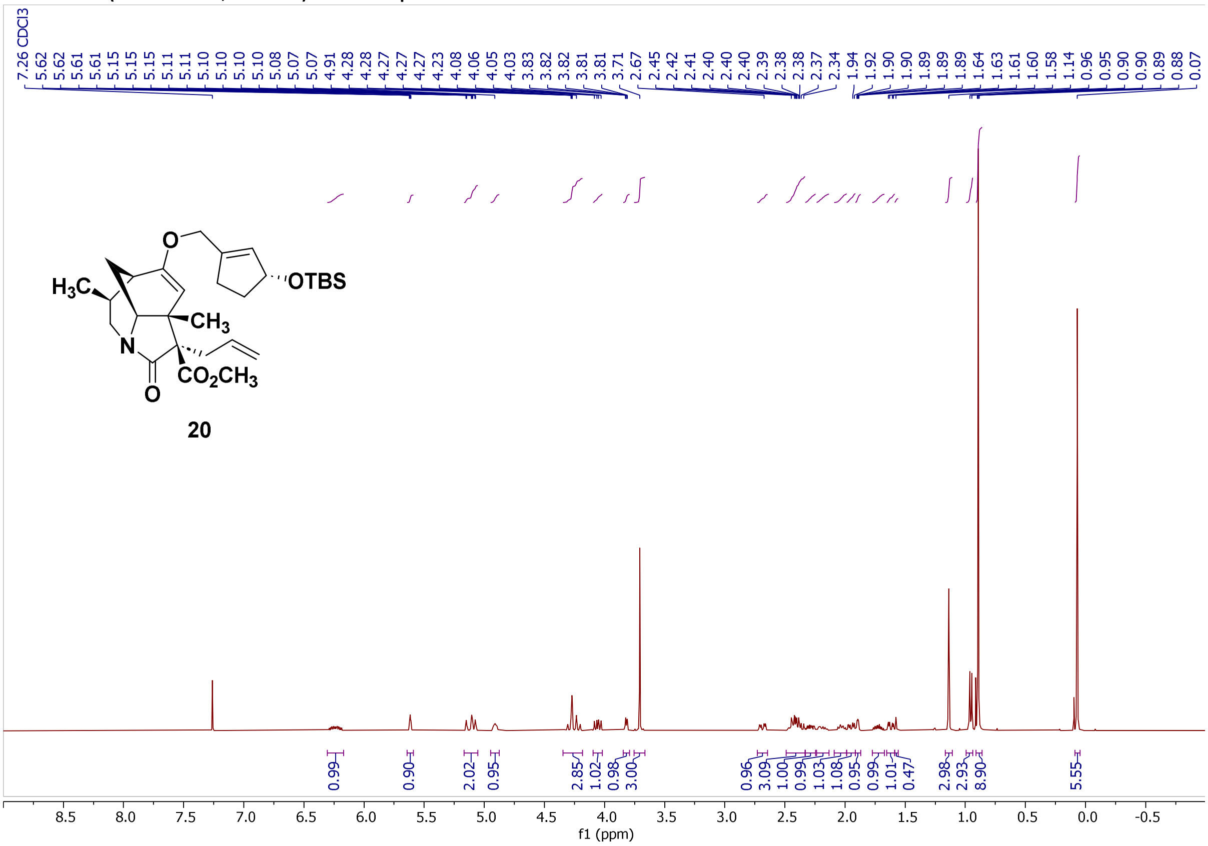

^{13}C NMR (126 MHz, $CDCl_3$) of compound **20.**

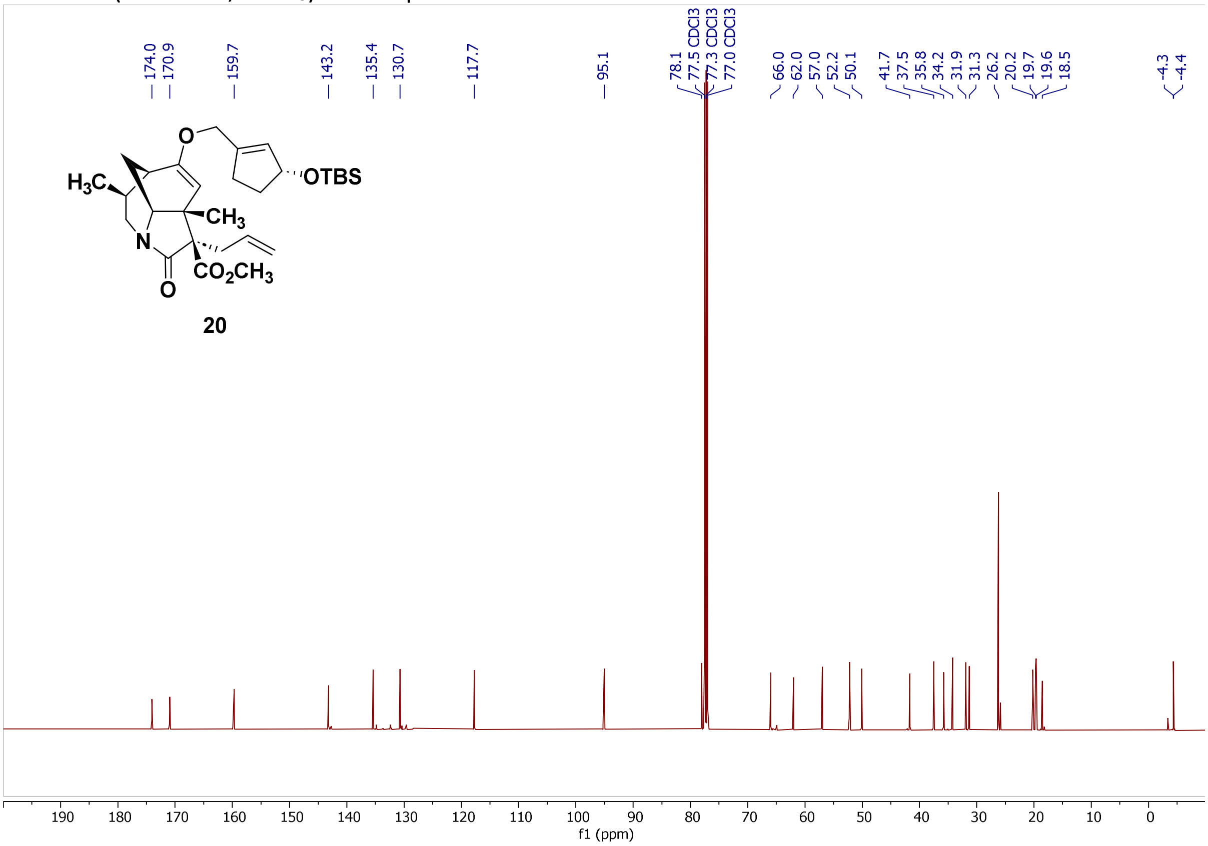

[1]**H NMR** (400 MHz, CDCl$_3$) of compound **S41.**

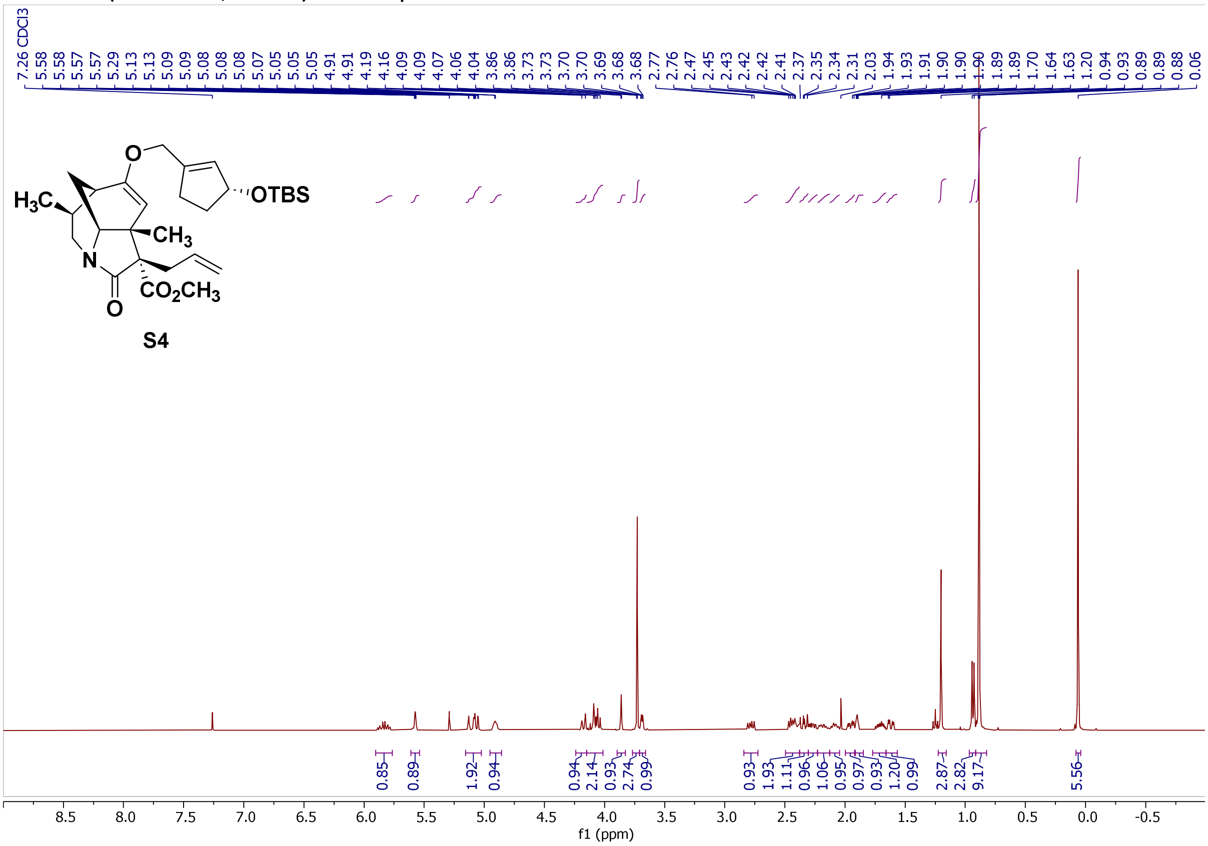

[13]**C NMR** (101 MHz, CDCl$_3$) of compound **S41.**

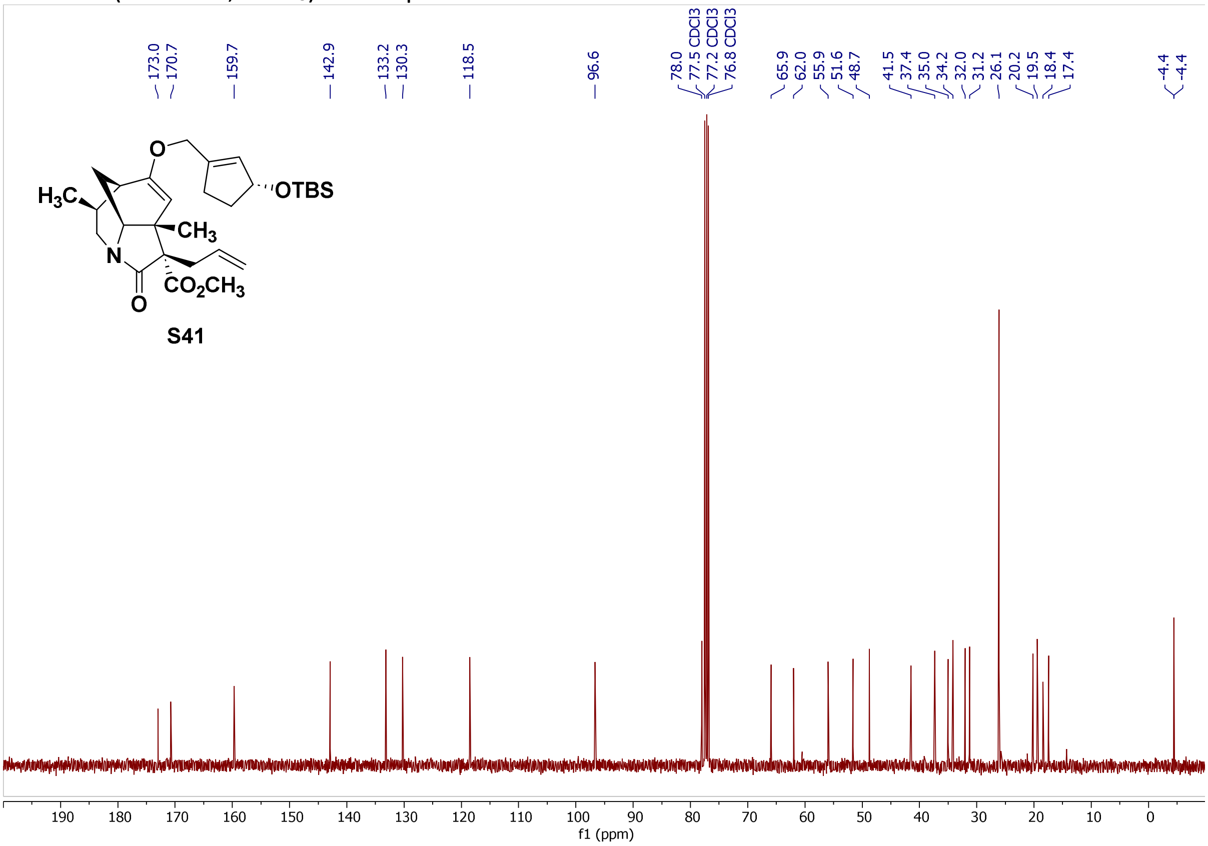

^{1}H NMR (400 MHz, $CDCl_3$) of compound **22.**

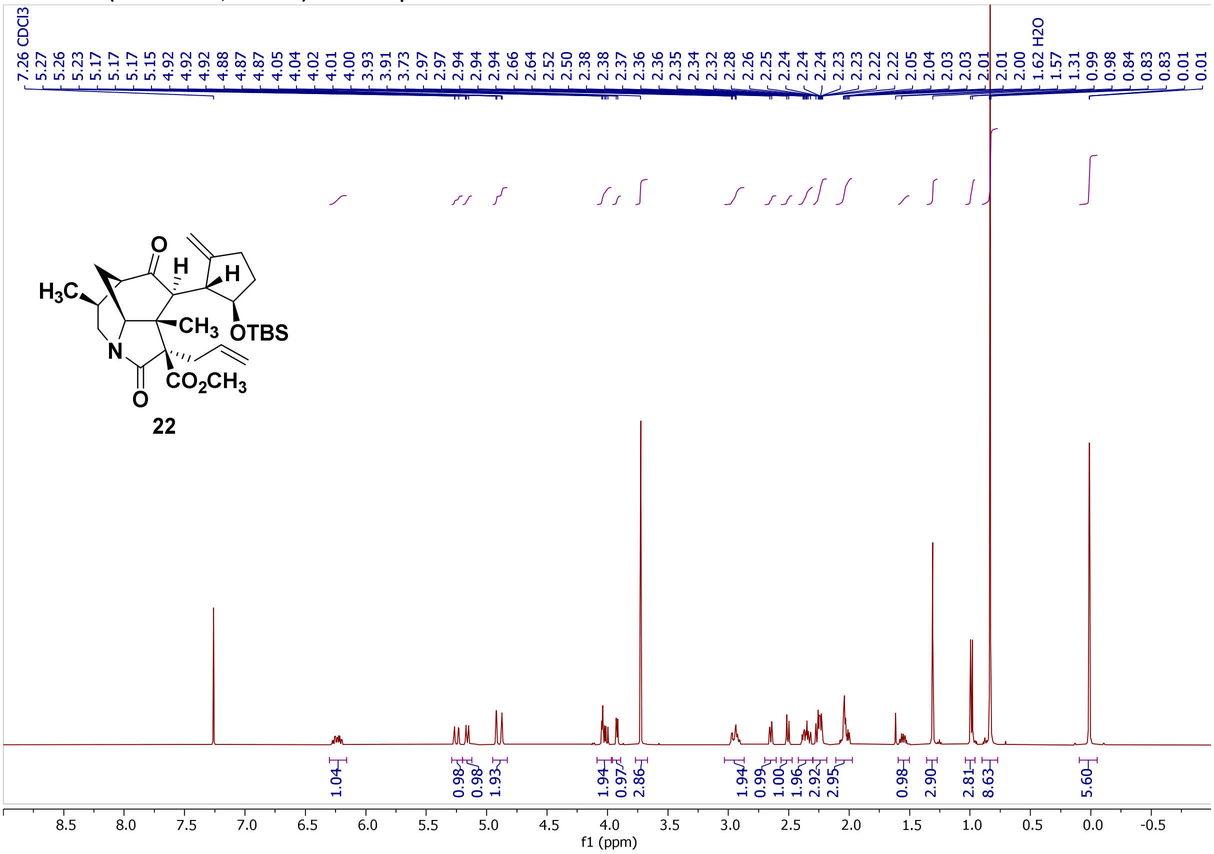

^{13}C NMR (101 MHz, $CDCl_3$) of compound **22.**

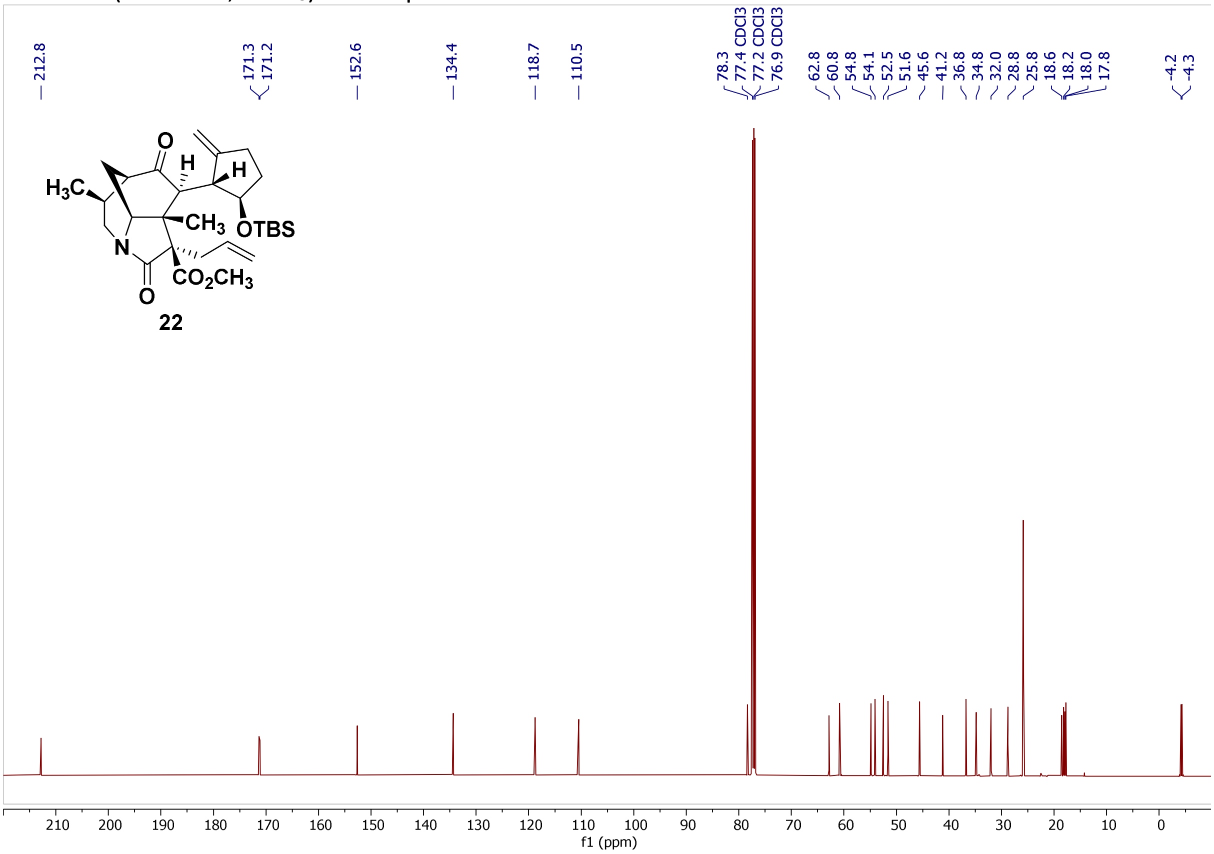

^{1}H NMR (500 MHz, $CDCl_3$) of compound **2.**

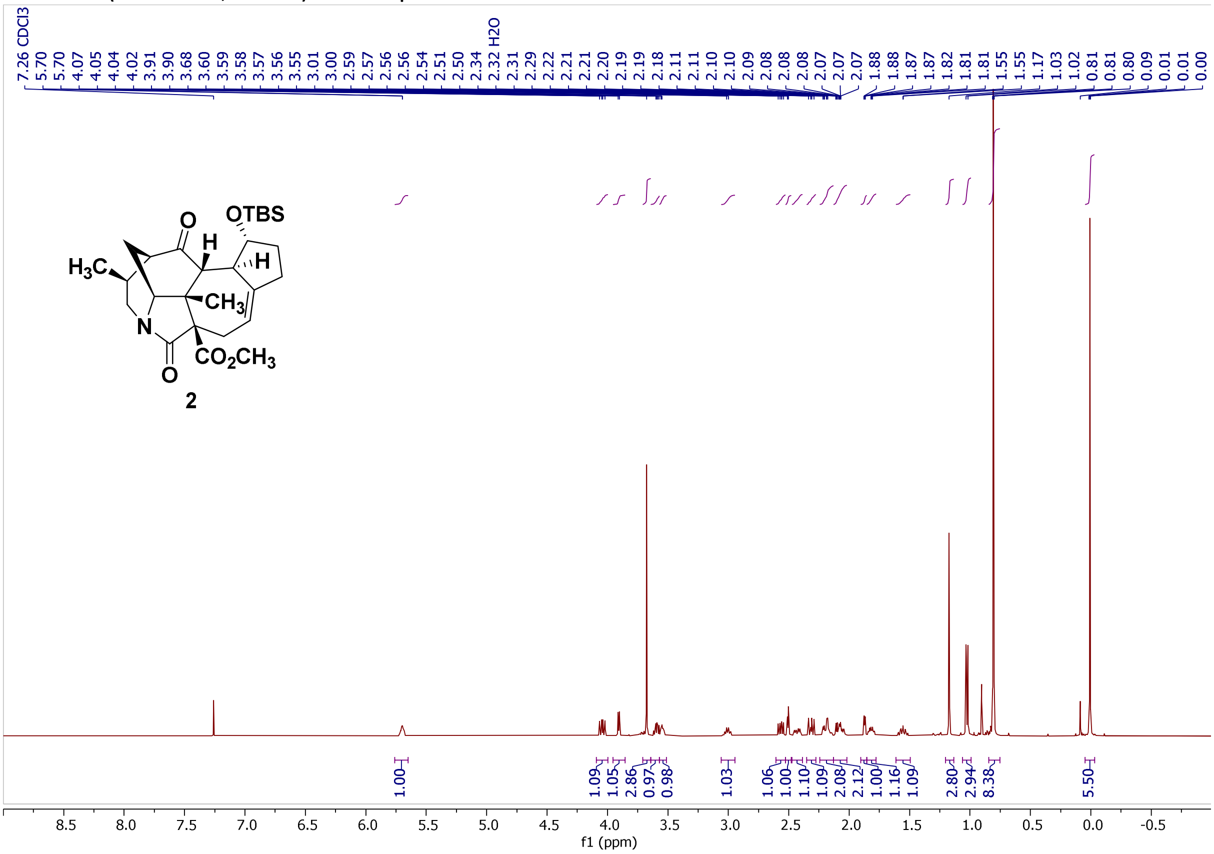

^{13}C NMR (126 MHz, $CDCl_3$) of compound **2.**

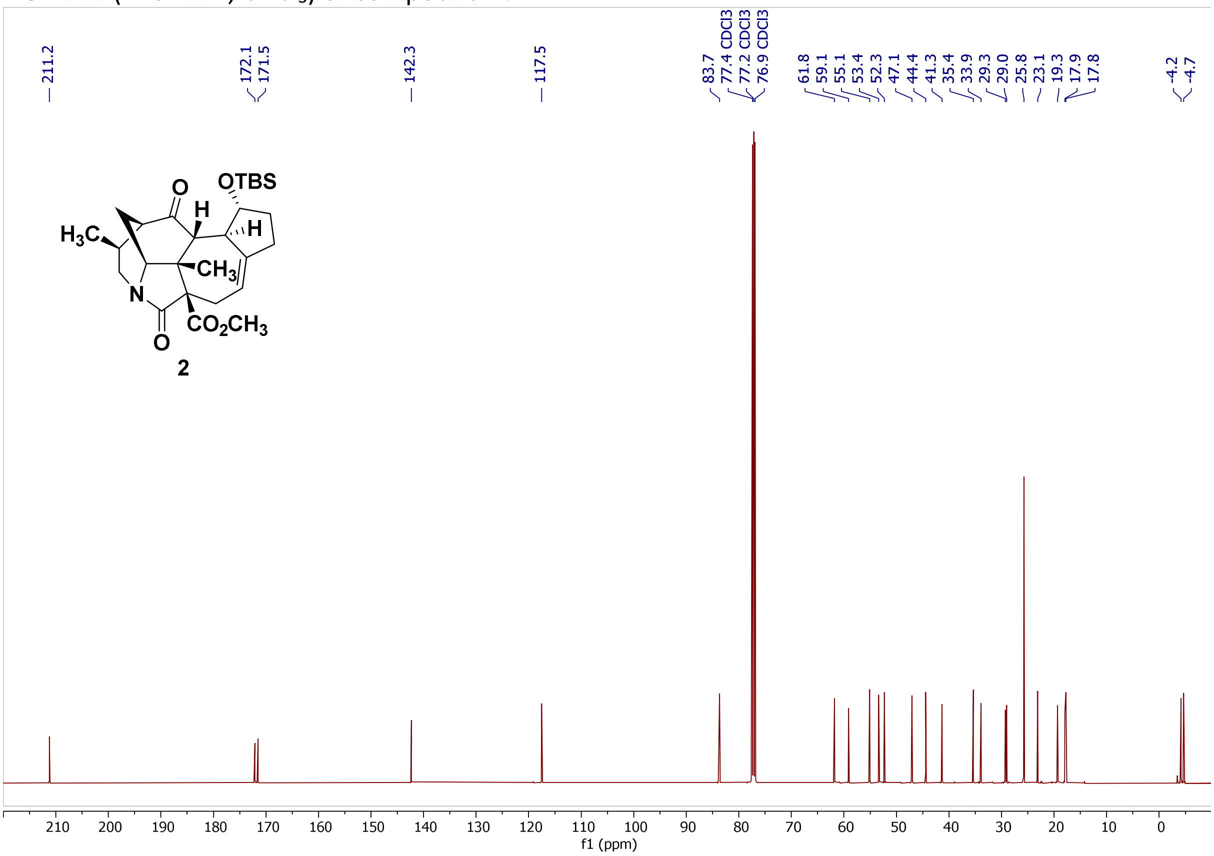

^{1}H NMR (500 MHz, $CDCl_3$) of compound **S42.**

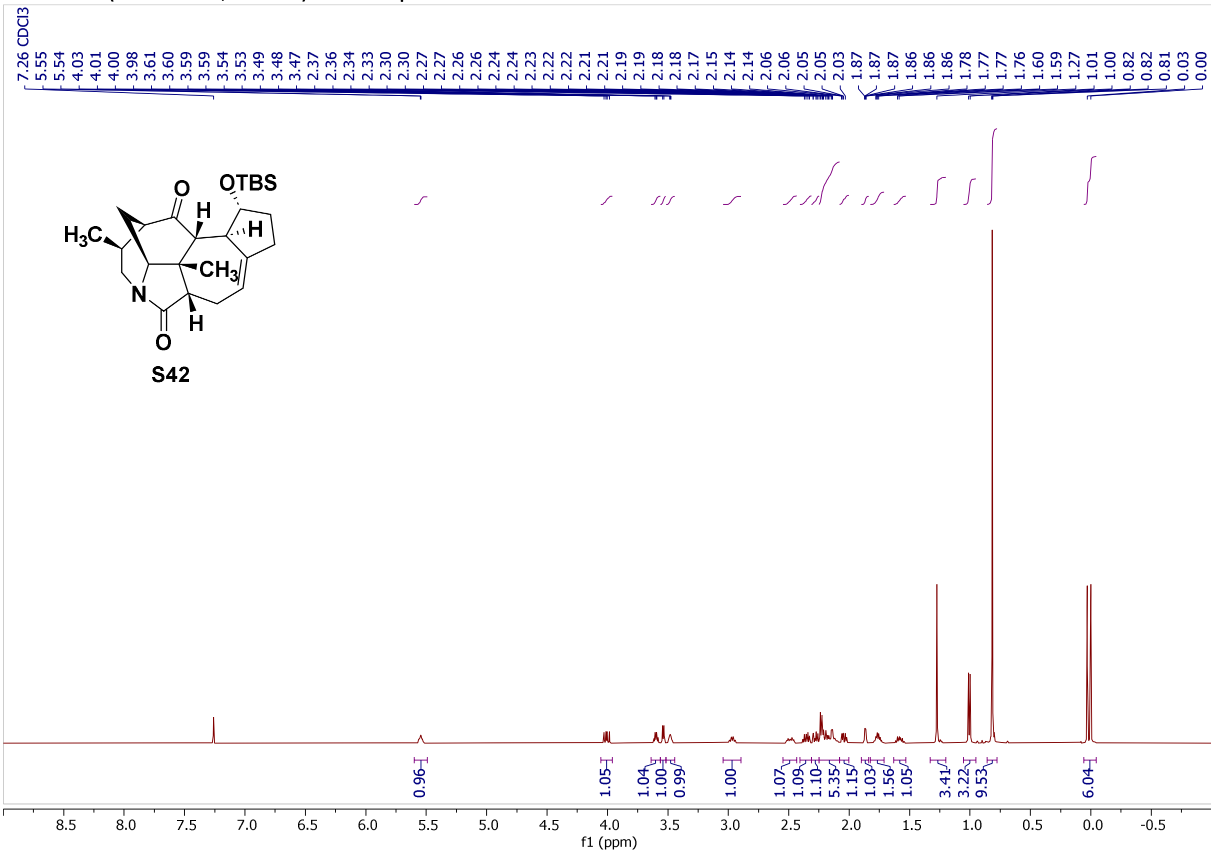

^{13}C NMR (126 MHz, $CDCl_3$) of compound **S42.**

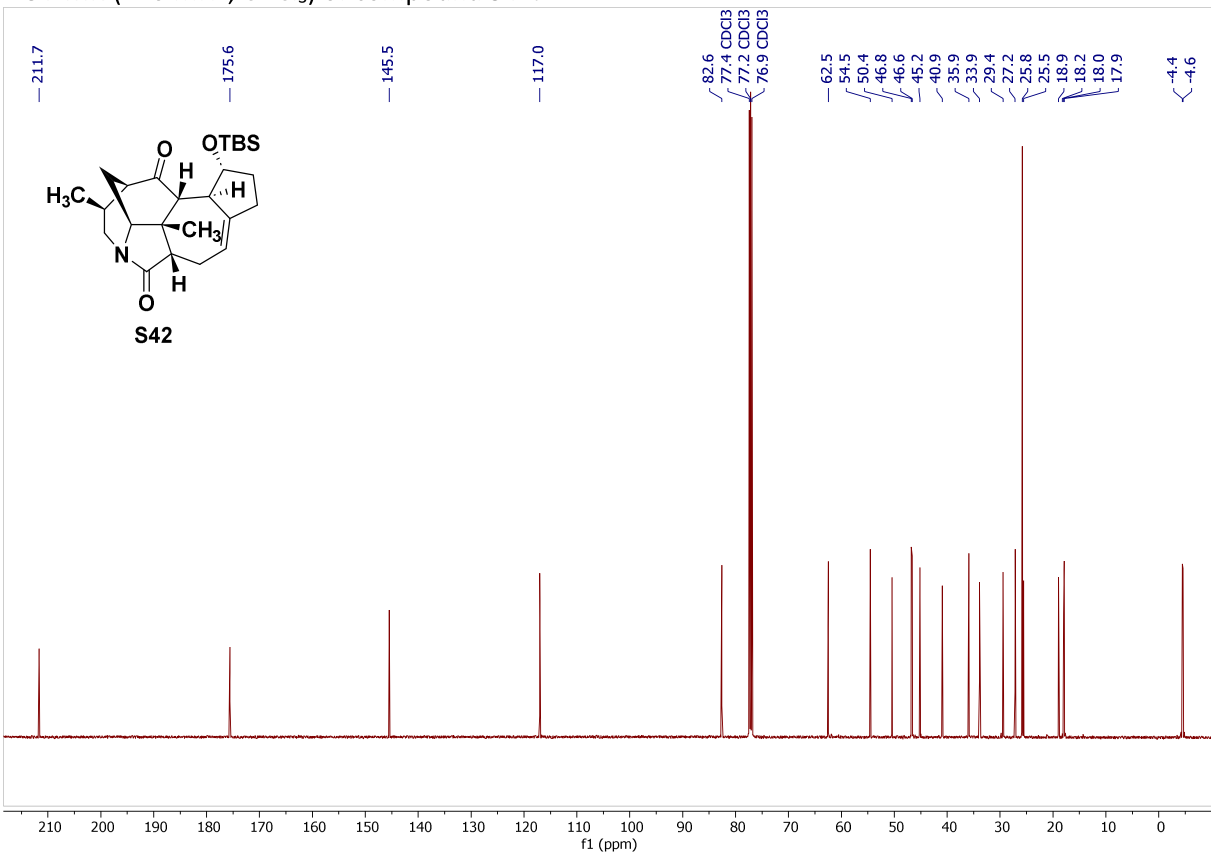

[1] **H NMR** (500 MHz, $CDCl_3$) of compound **24.**

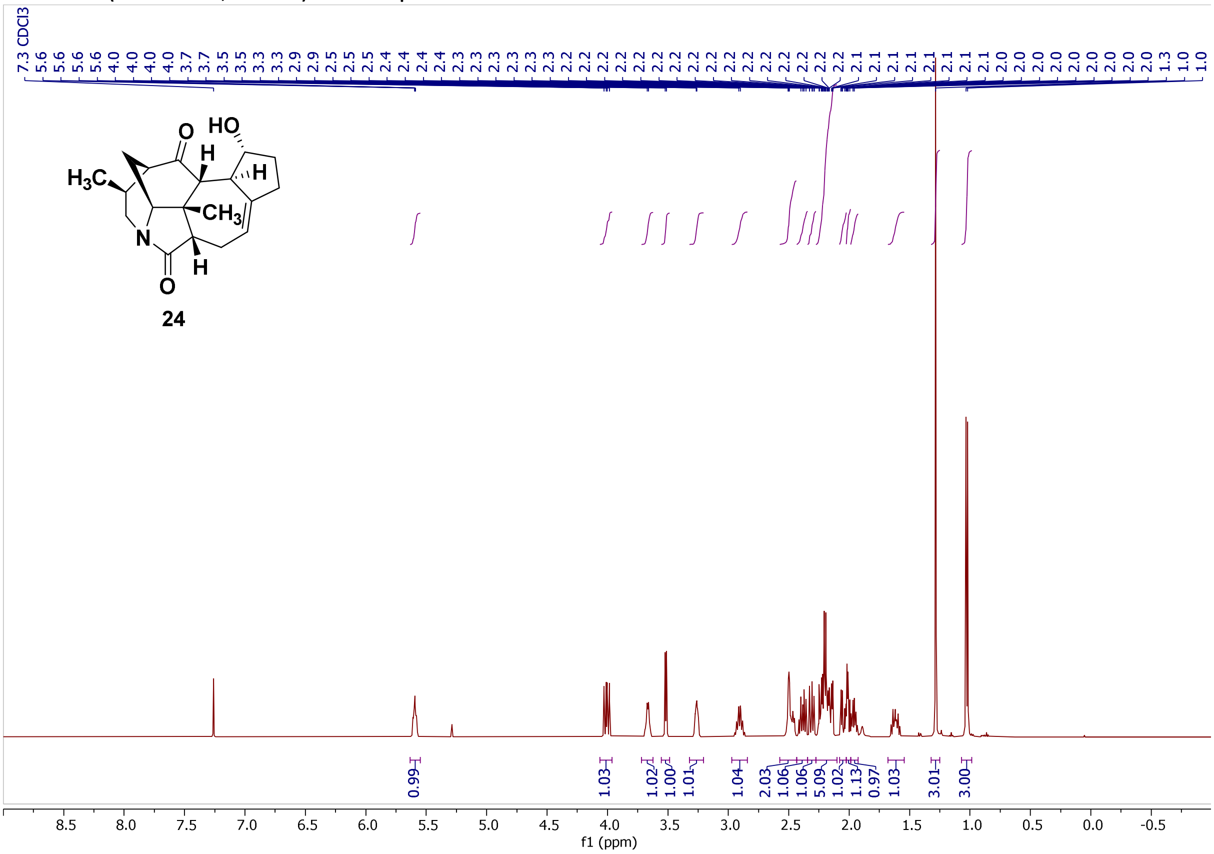

[13] **C NMR** (101 MHz, $CDCl_3$) of compound **24.**

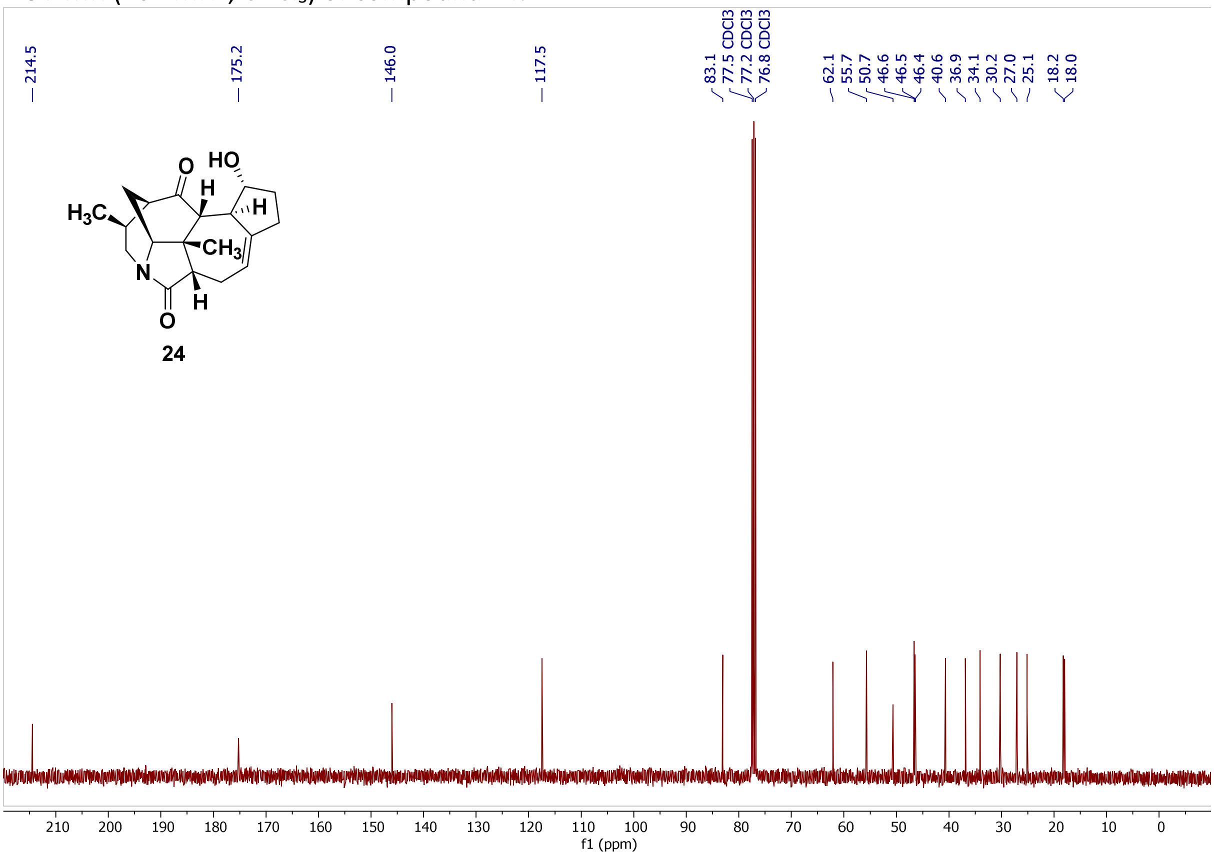

^{1}H NMR (400 MHz, $CDCl_3$) of compound **S43.**

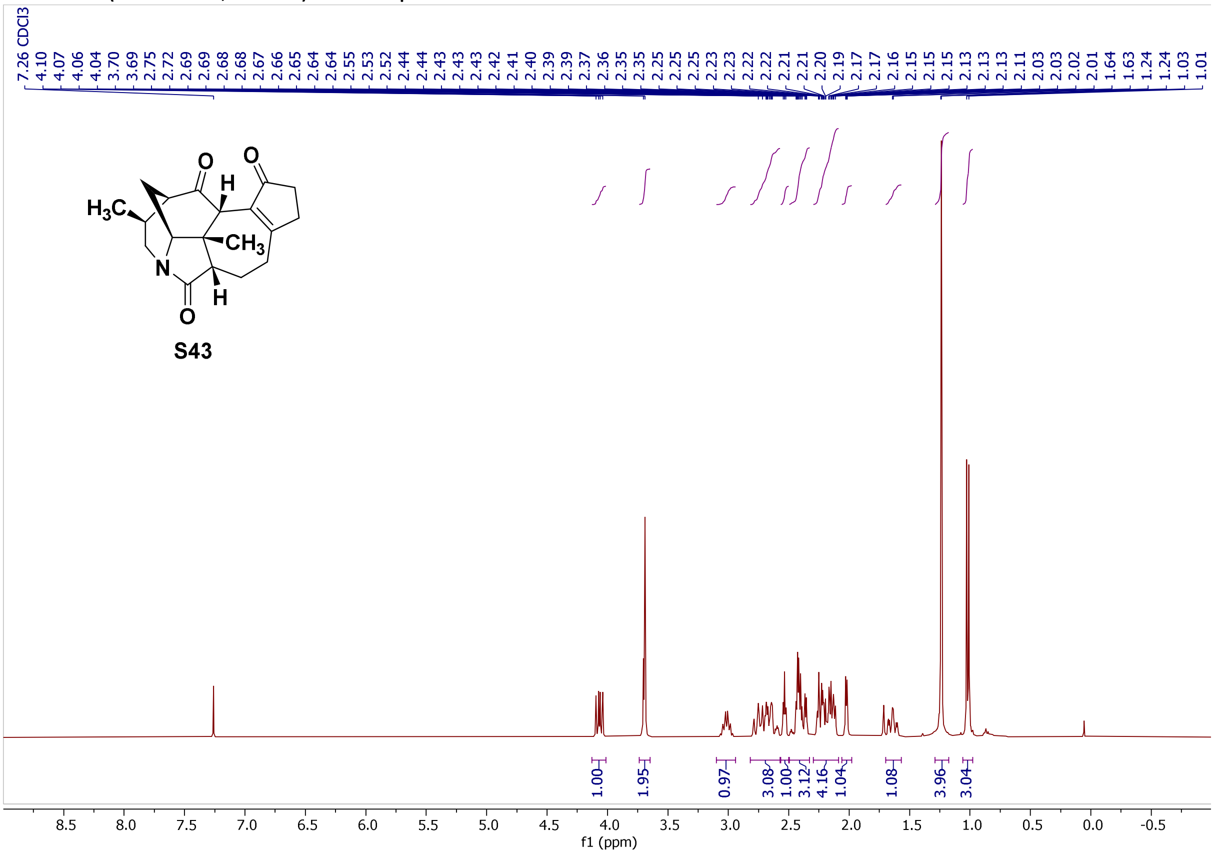

^{13}C NMR (101 MHz, $CDCl_3$) of compound **S43.**

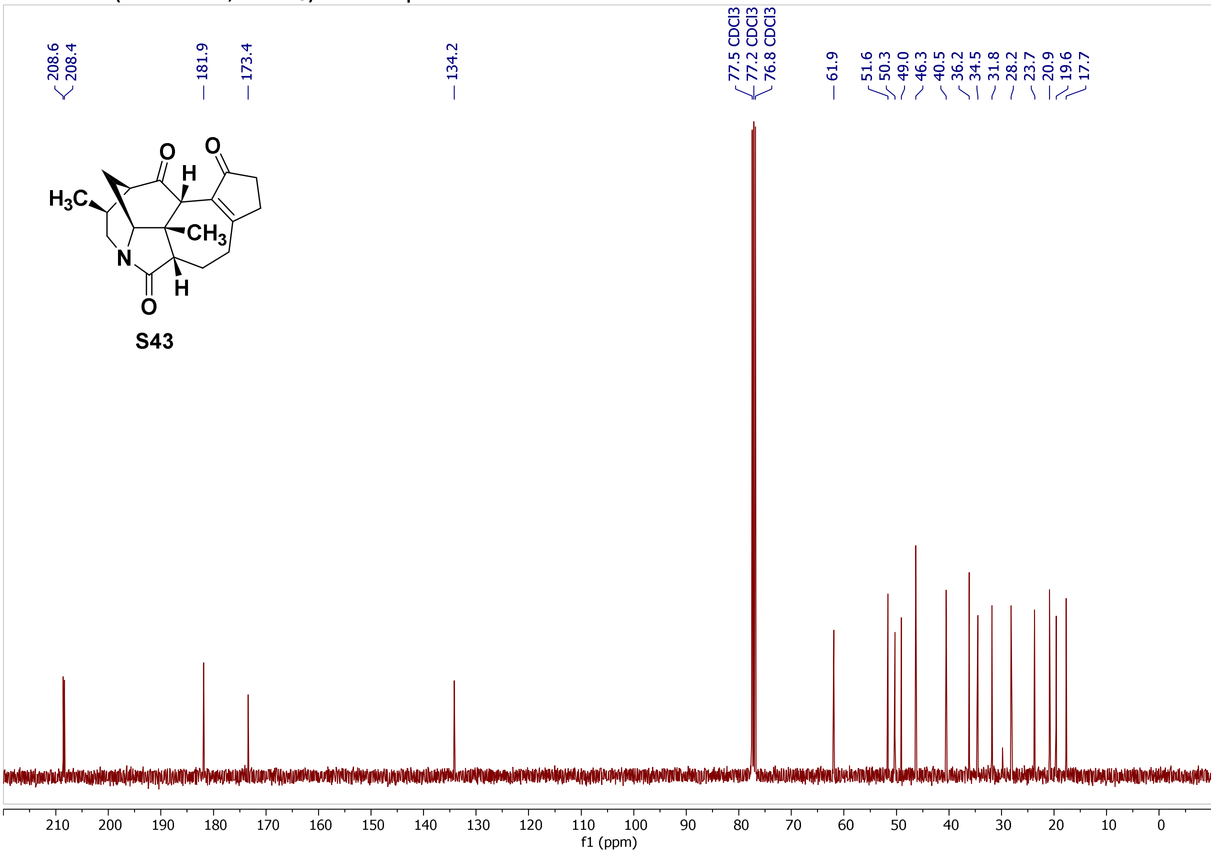

^{1}**H NMR** (600 MHz, $CDCl_3$) of himalensine A **(1).**

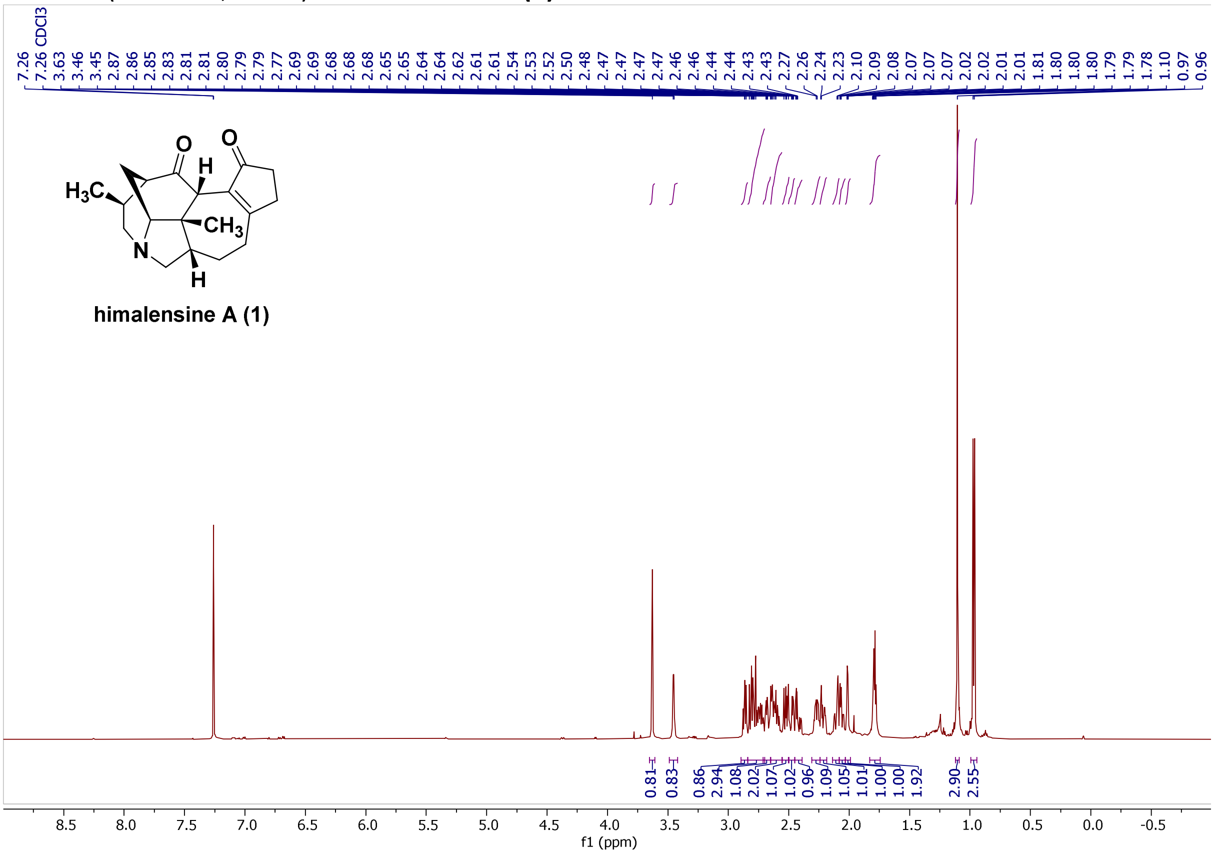

^{13}**C NMR** (151 MHz, $CDCl_3$) of himalensine A **(1).**

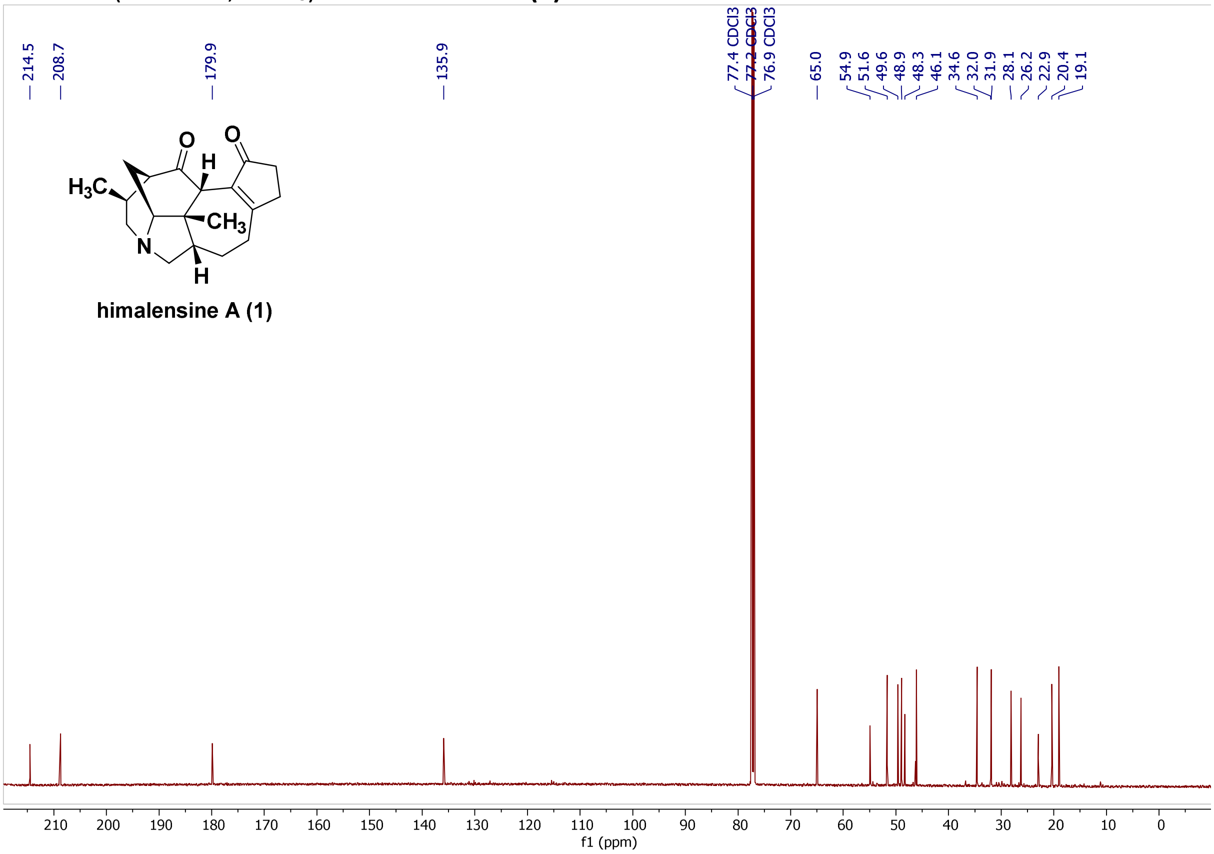

^{1}H NMR (400 MHz, $CDCl_3$) of compound **S44.**

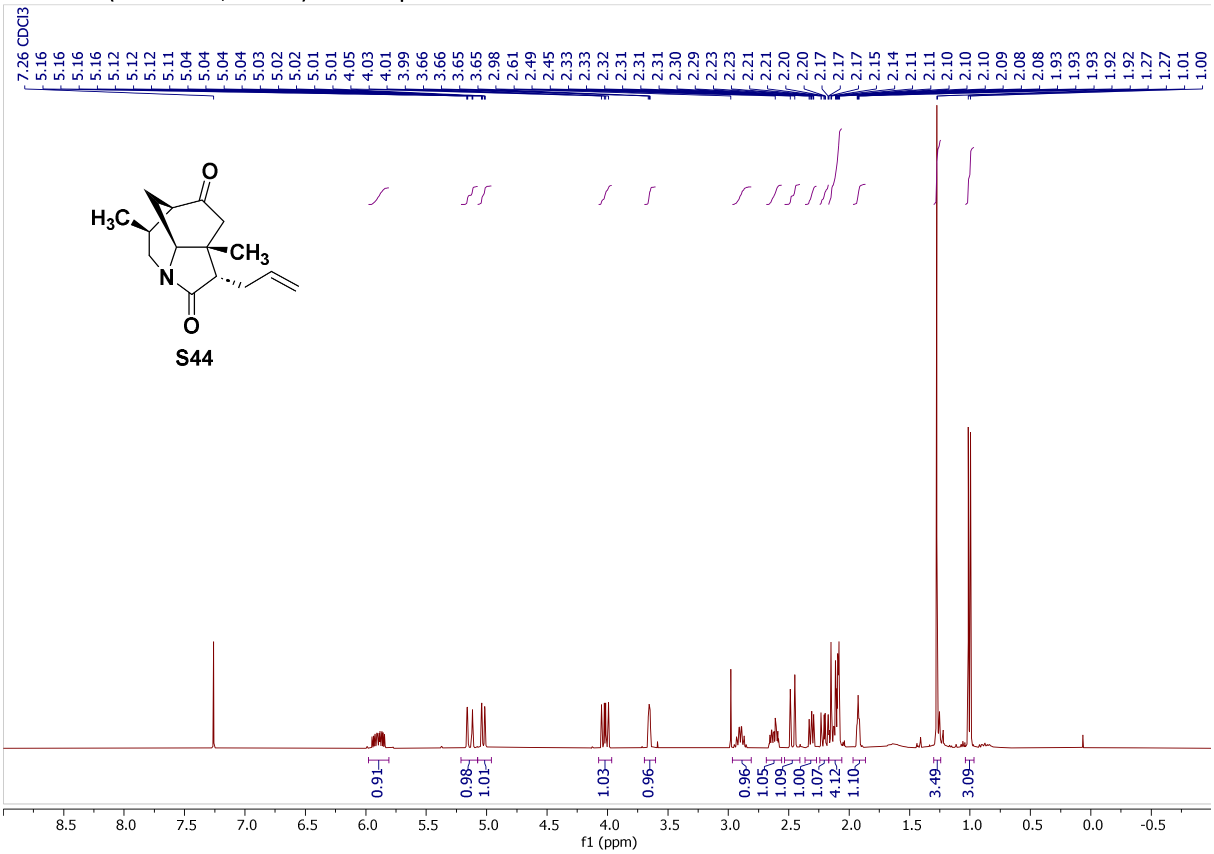

^{13}C NMR (101 MHz, $CDCl_3$) of compound **S44.**

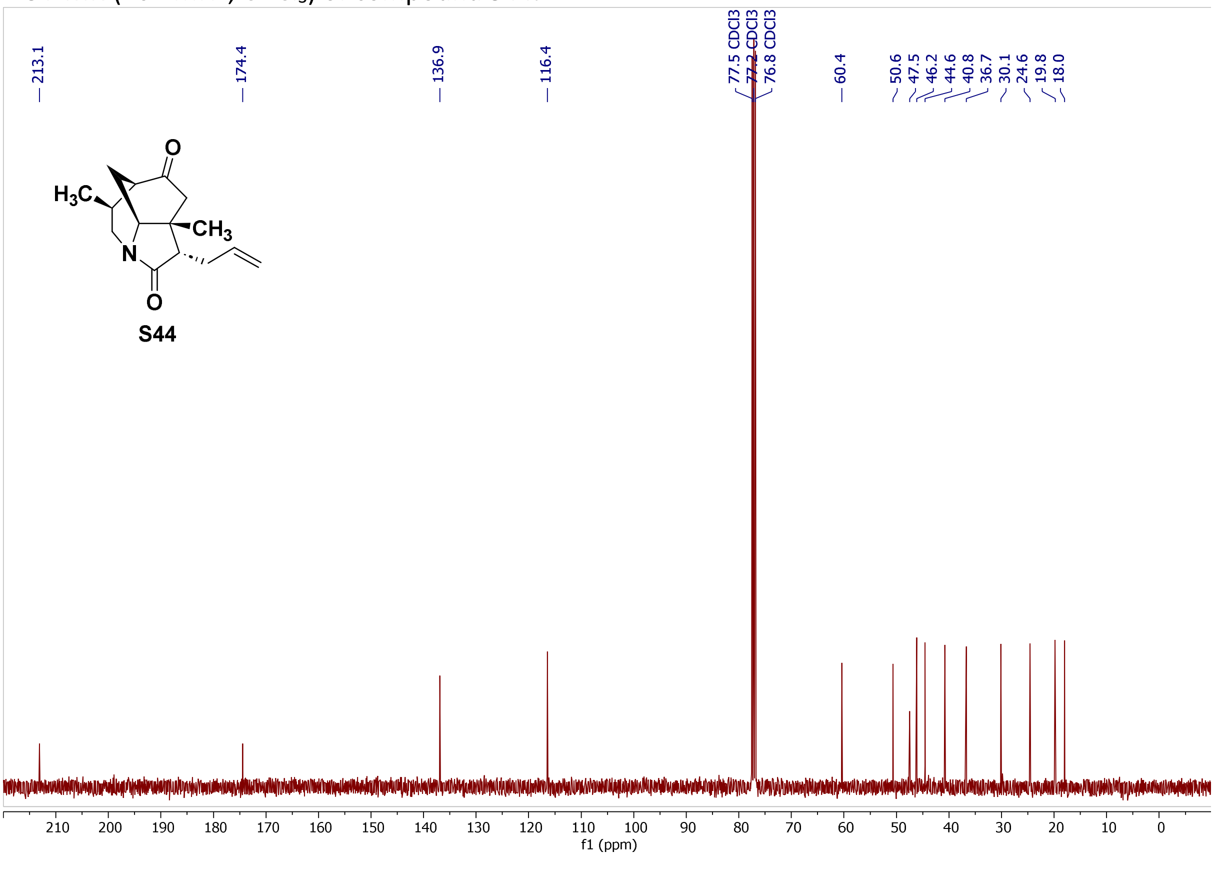

^{1}H **NMR** (400 MHz, $CDCl_3$) of 1:1 mixture of compound **S45a** and **S45b.**

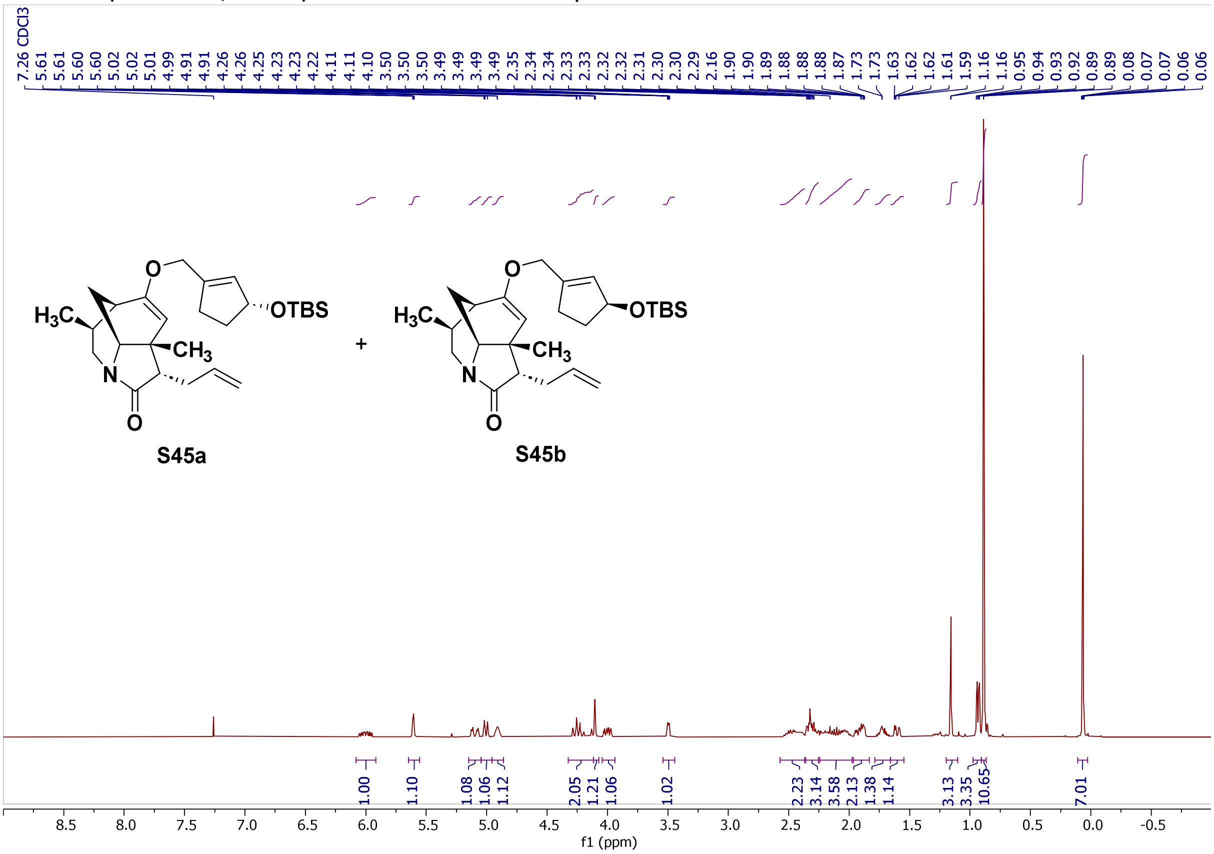

^{13}C **NMR** (101 MHz, $CDCl_3$) of compound **S45a** and **S45b.**

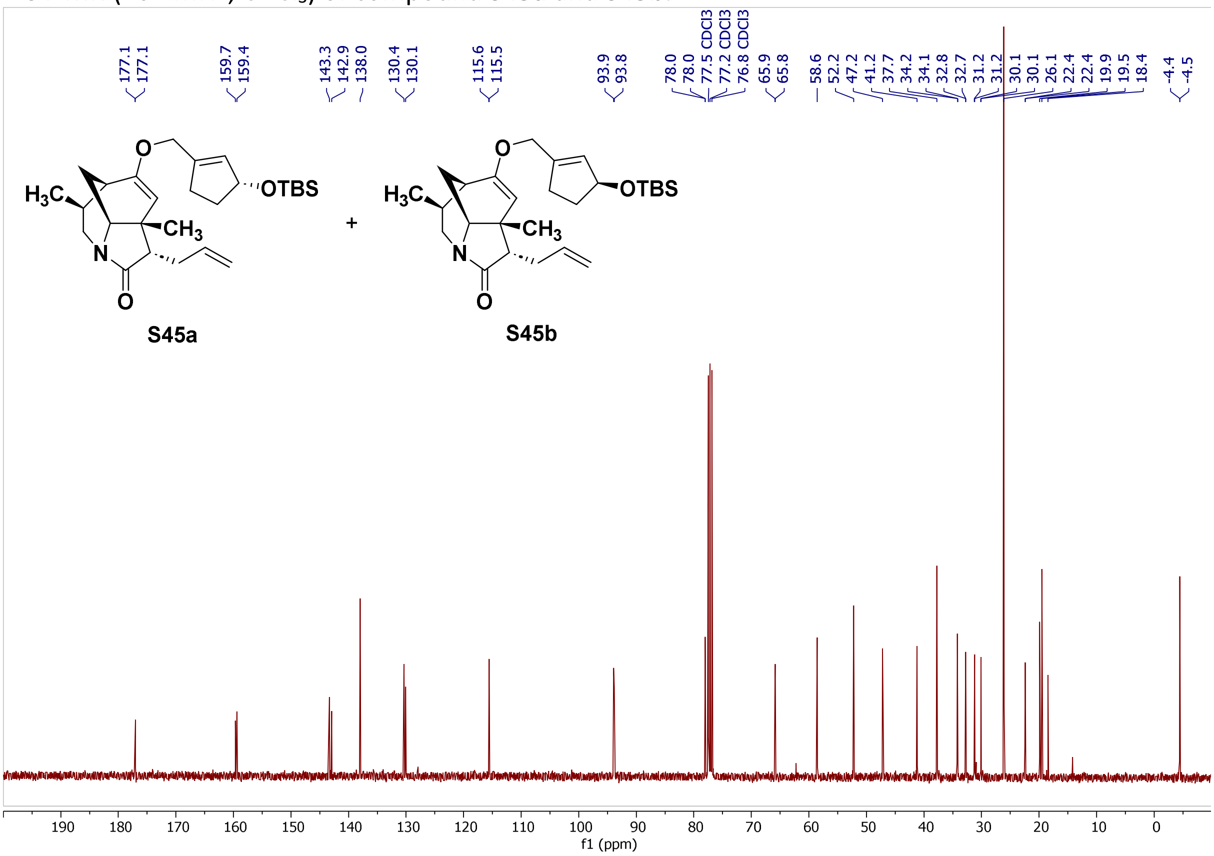

^{1}**H NMR** (500 MHz, $CDCl_3$) of compound **23.**

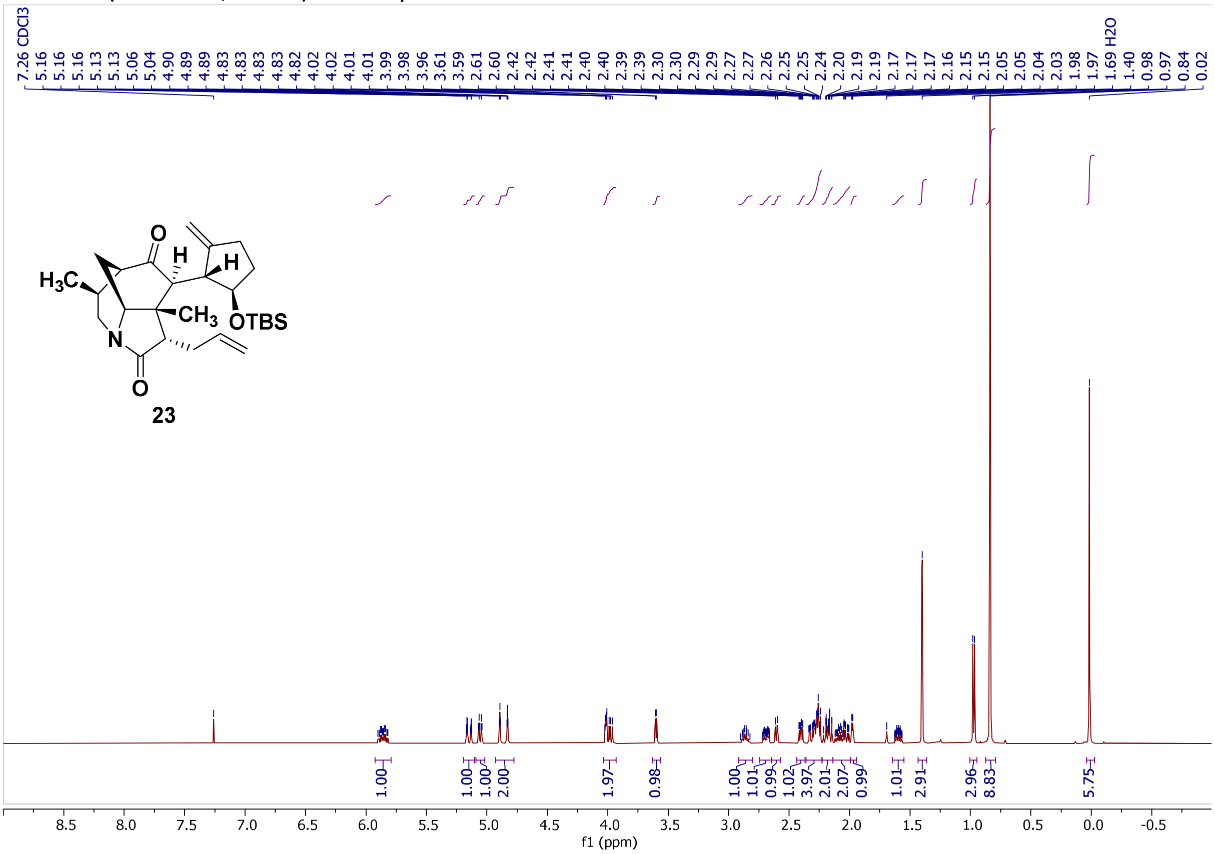

^{13}**C NMR** (1126 MHz, $CDCl_3$) of compound **23.**

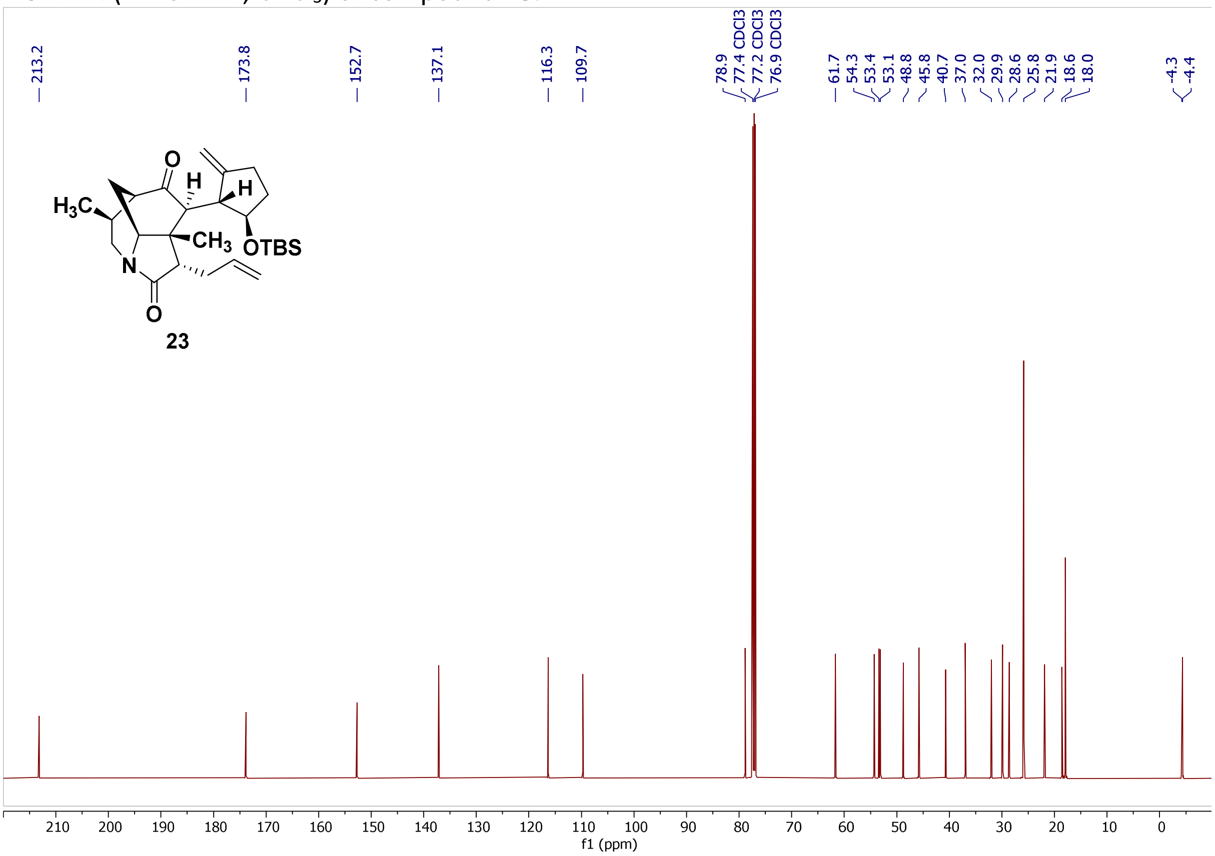

^{1}H NMR (400 MHz, $CDCl_3$) of compound **13d.**

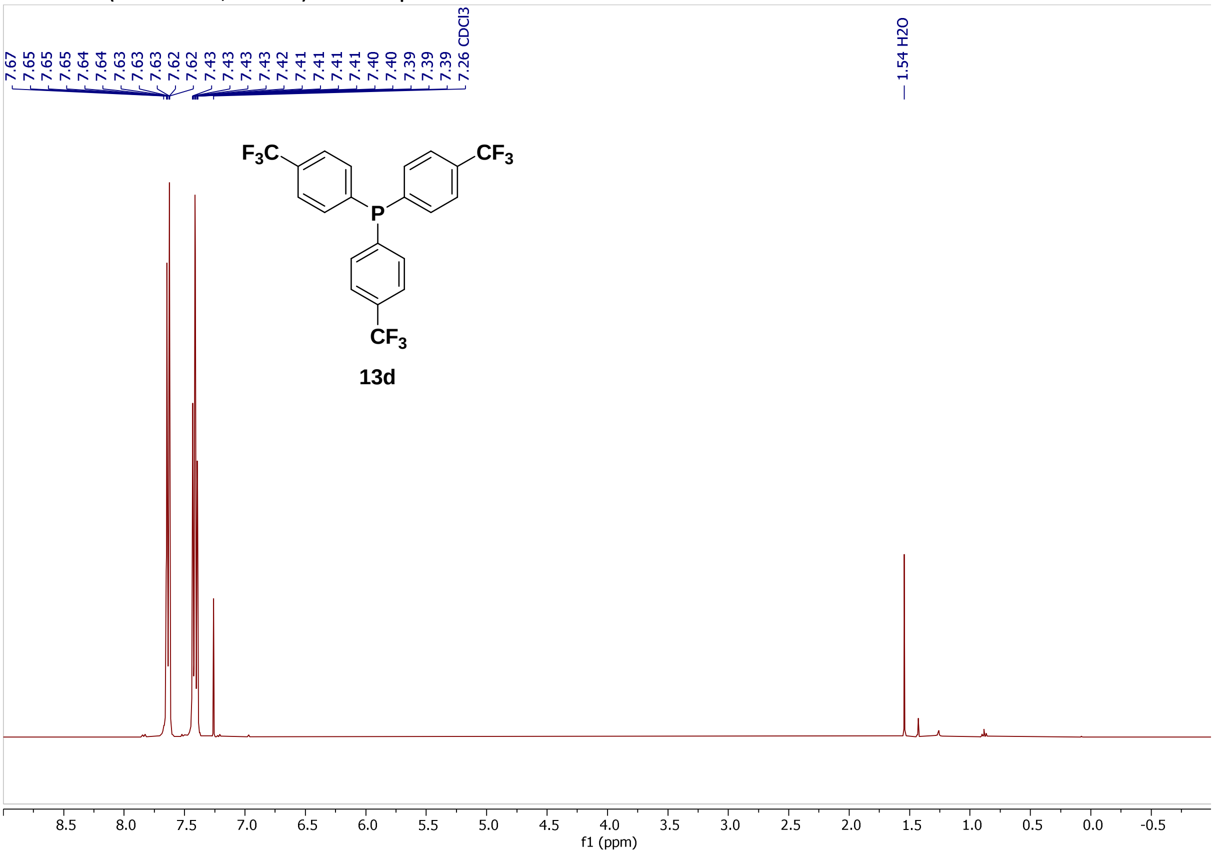

^{13}C NMR (101 MHz, $CDCl_3$) of compound **13d.**

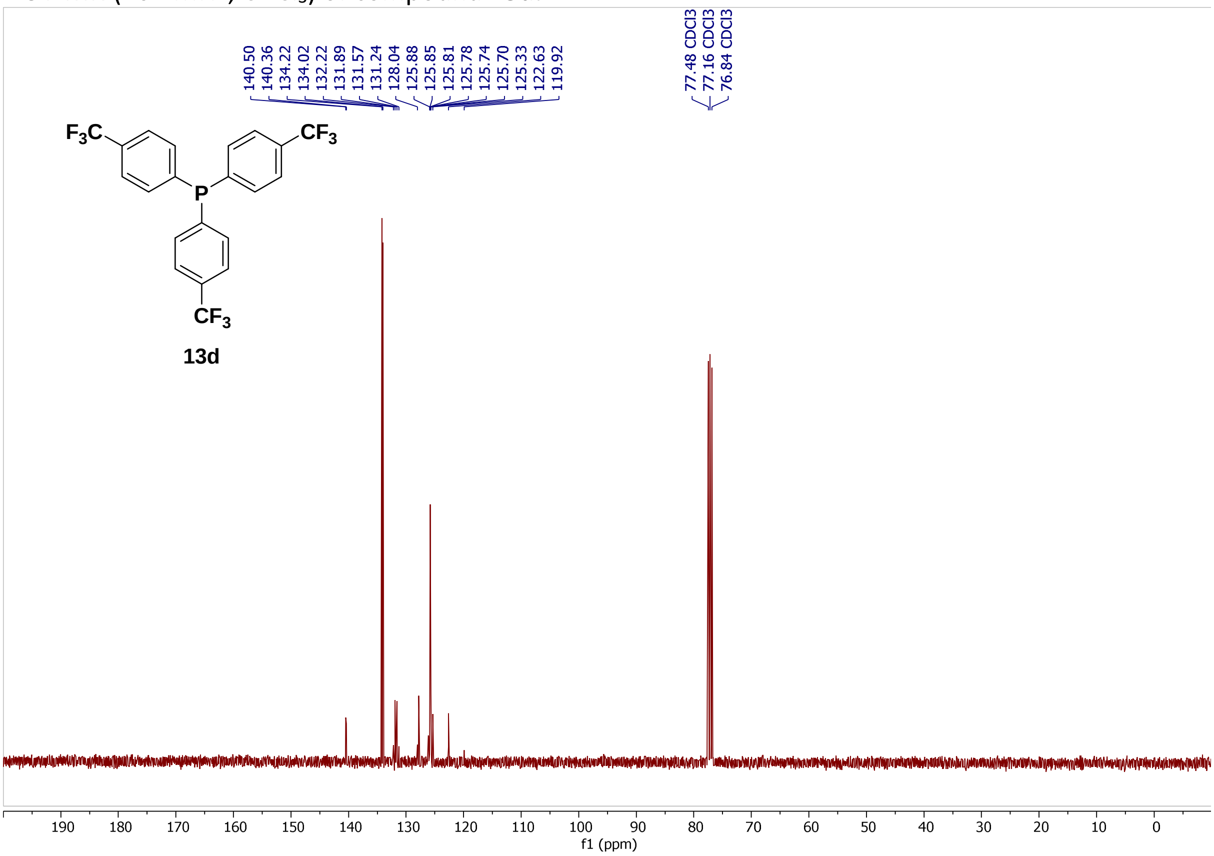

^{19}**F NMR** (376 MHz, $CDCl_3$) of compound **13d.**

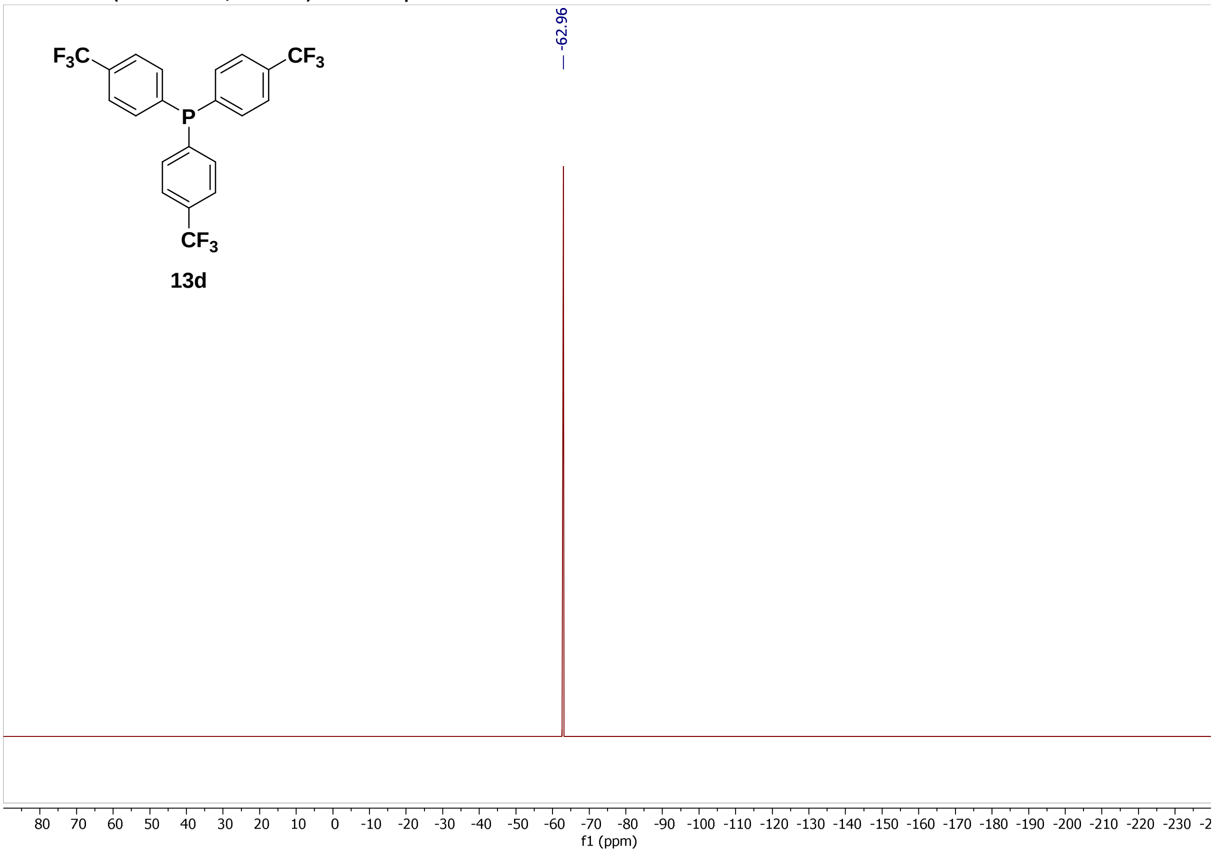

^{31}**P NMR** (162 MHz, $CDCl_3$) of compound **13d.**

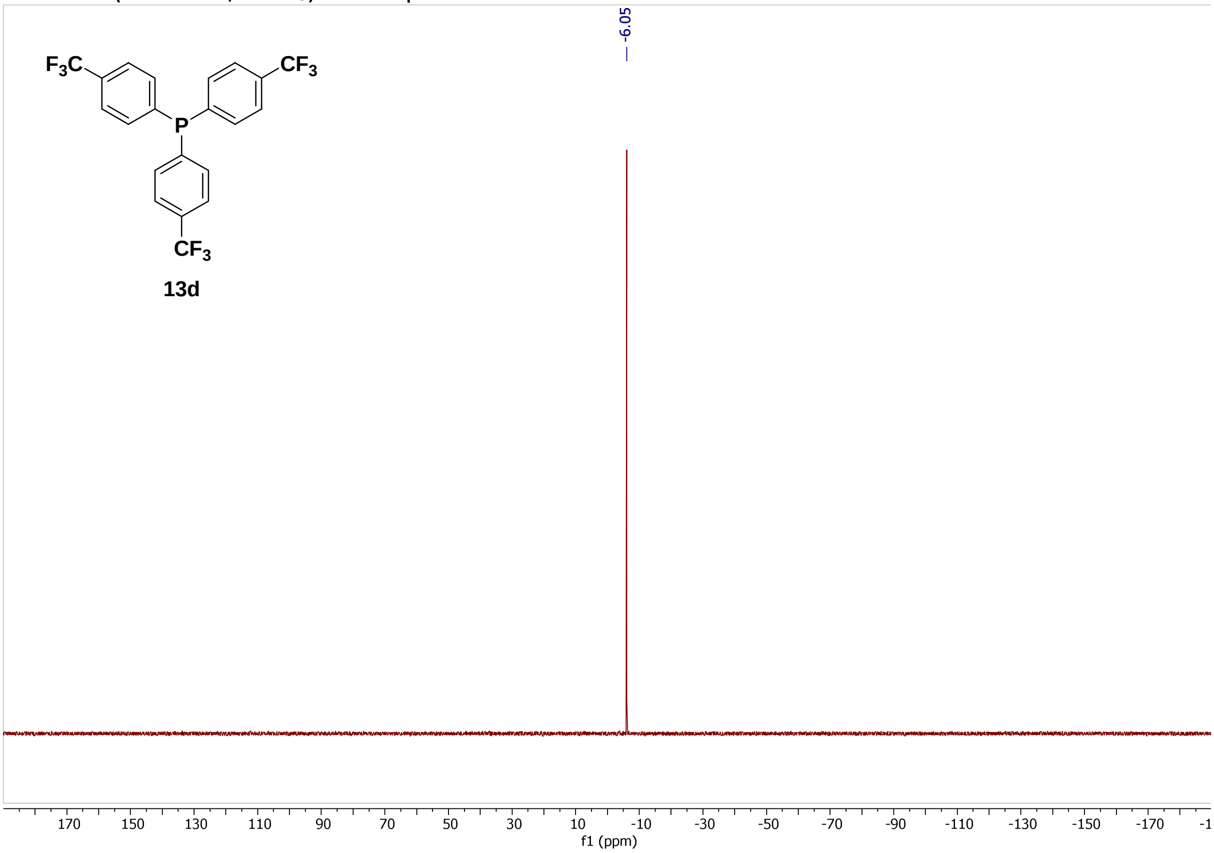

^{1}H NMR (400 MHz, $CDCl_3$) of compound **S48.**

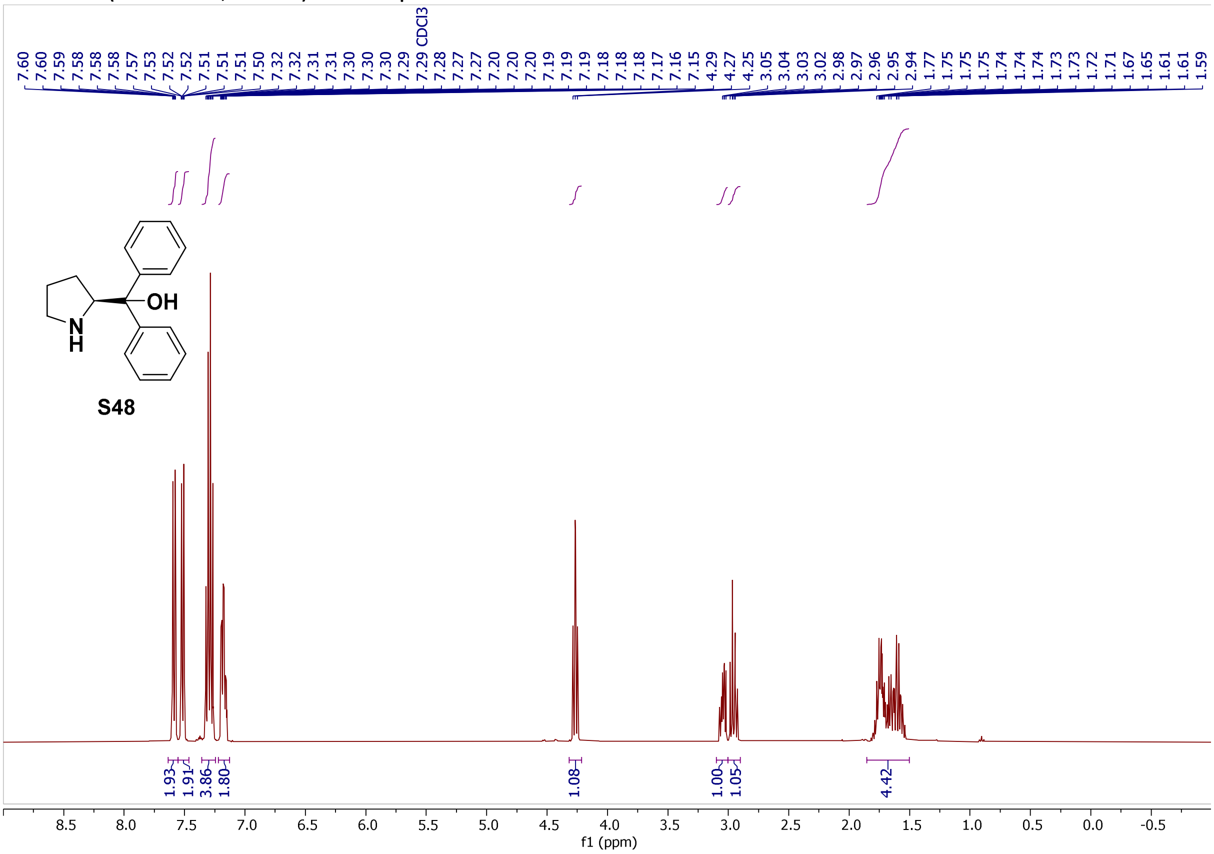

^{13}C NMR (101 MHz, $CDCl_3$) of compound **S48.**

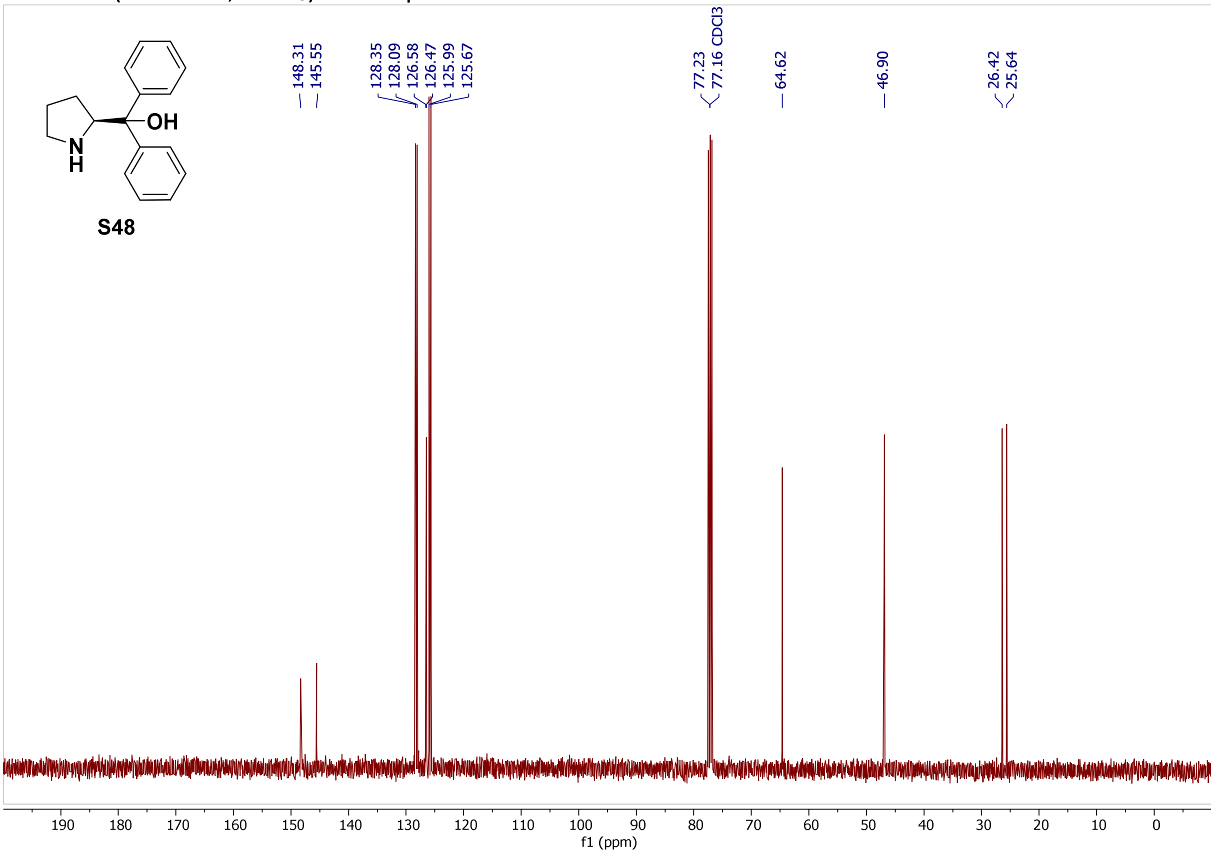

^{1}H NMR (400 MHz, $CDCl_3$) of compound **S49.**

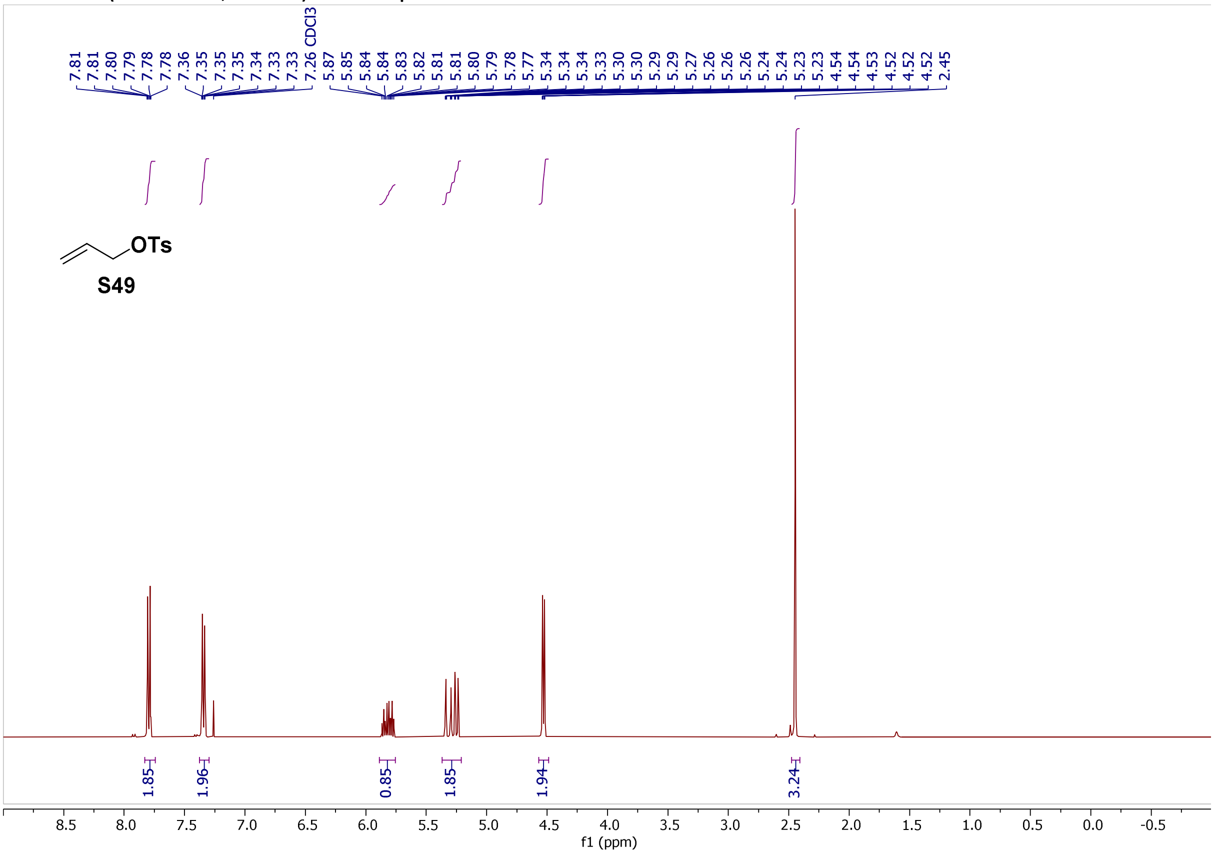

^{13}C NMR (101 MHz, $CDCl_3$) of compound **S49.**

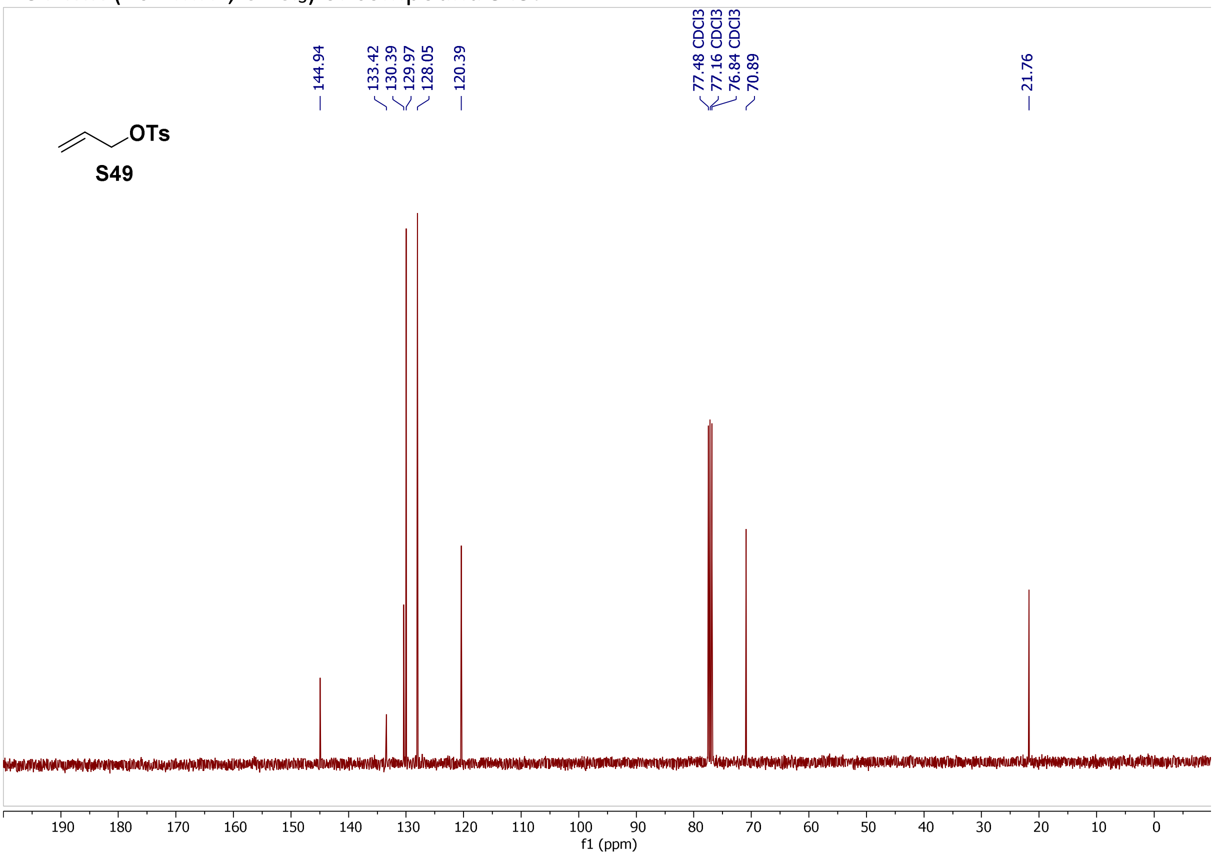

^{1}H NMR (400 MHz, $CDCl_3$) of compound **S52.**

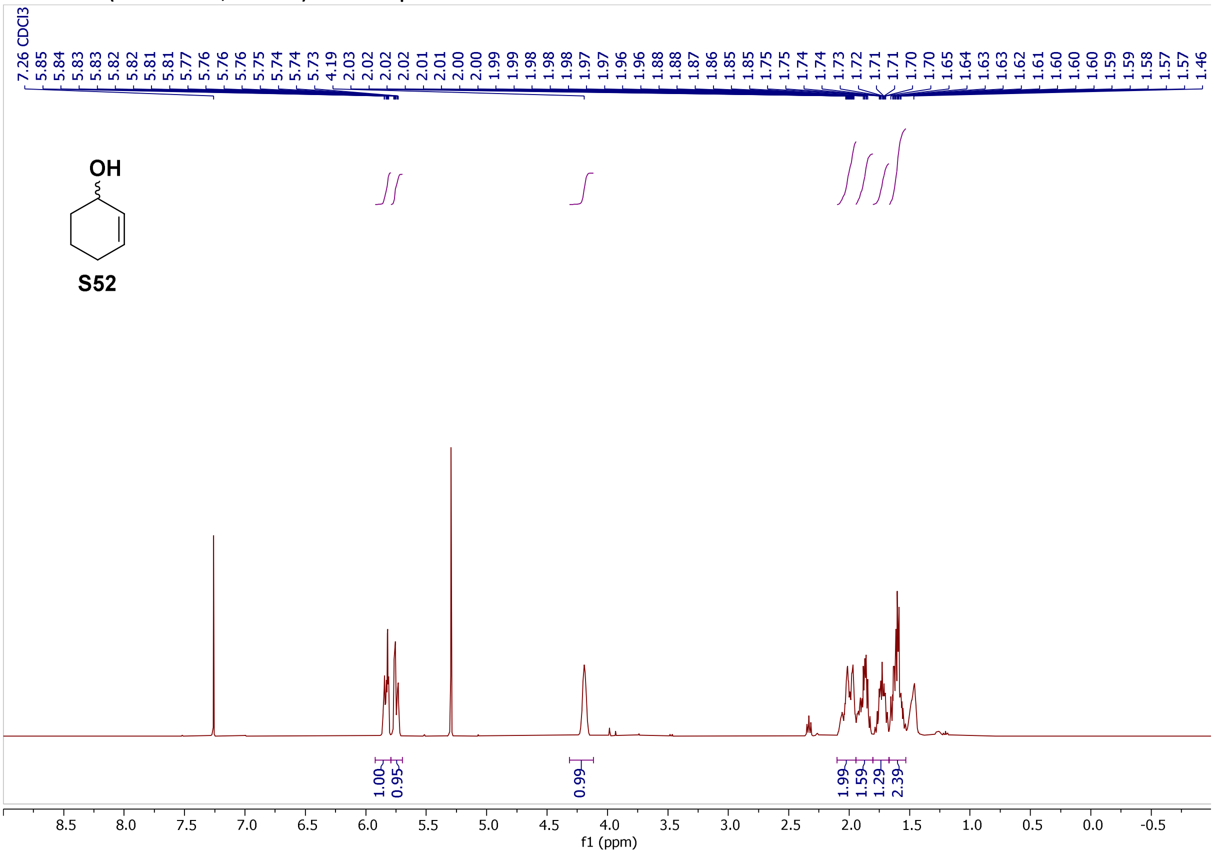

^{13}C NMR (101 MHz, $CDCl_3$) of compound **S52.**

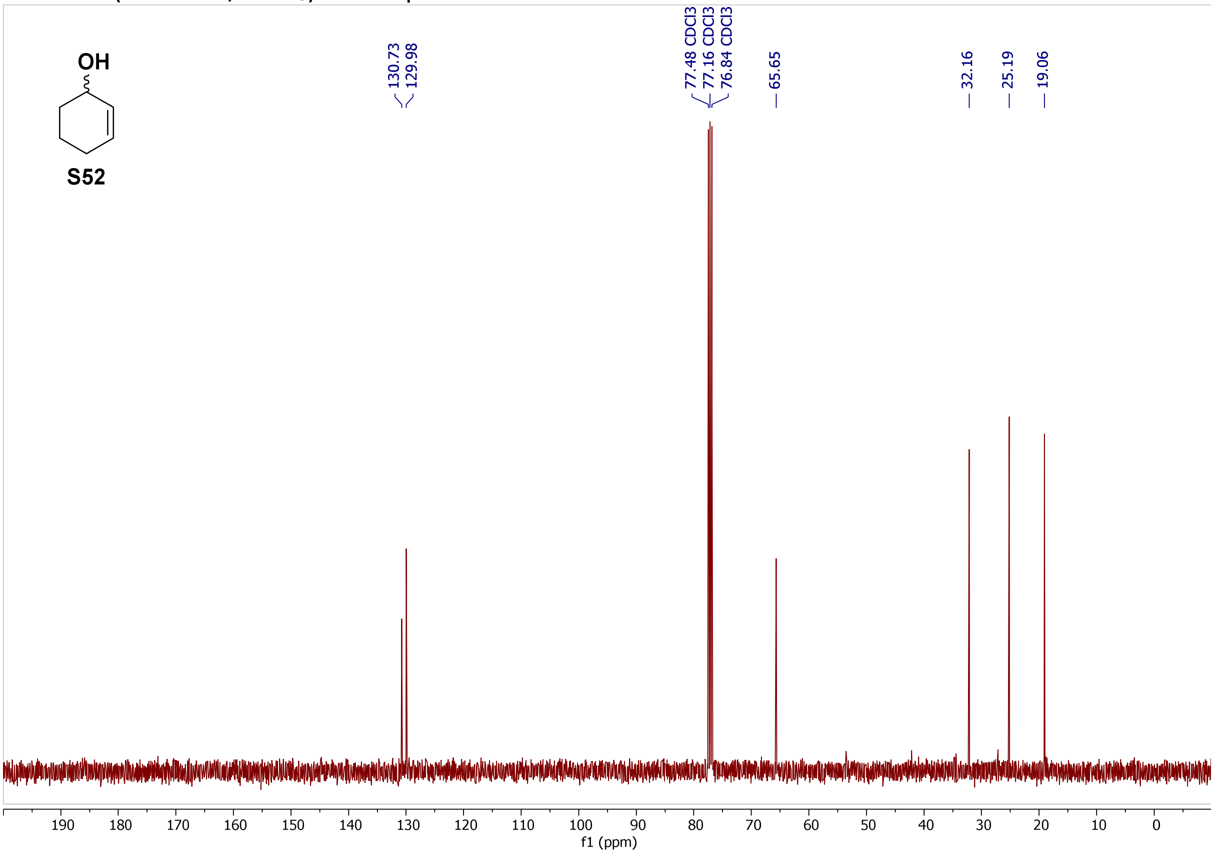

^{1}H NMR (400 MHz, $CDCl_3$) of compound S50.

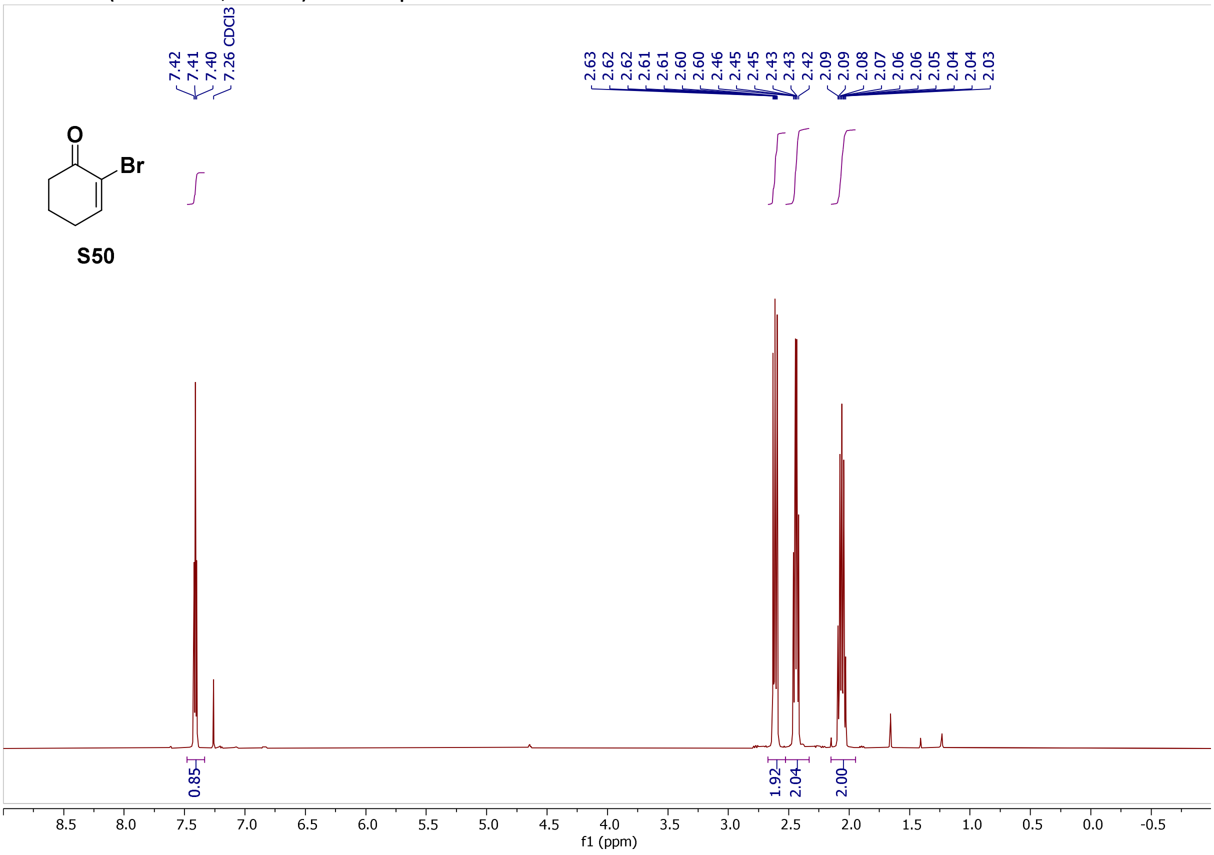

^{13}C NMR (101 MHz, $CDCl_3$) of compound S50.

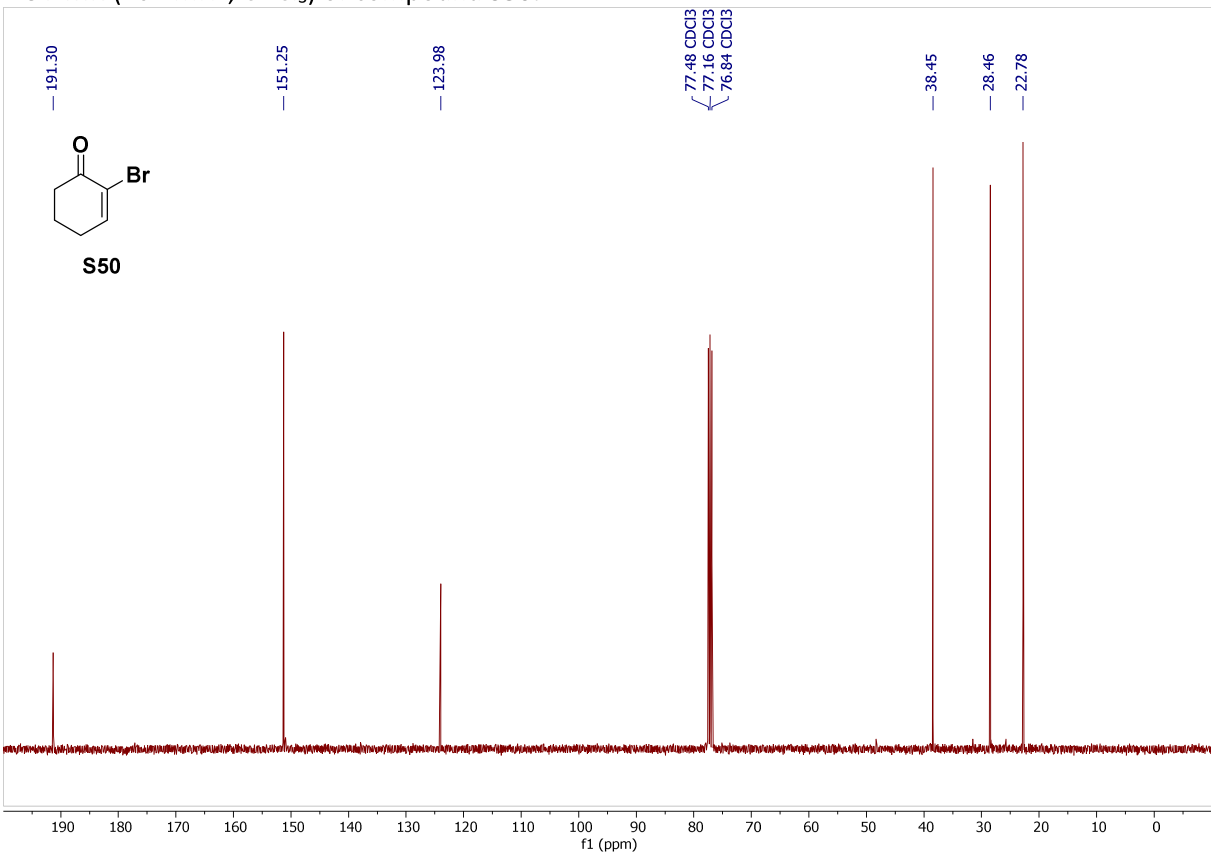

^{1}**H NMR** (400 MHz, $CDCl_3$) of compound **S51.**

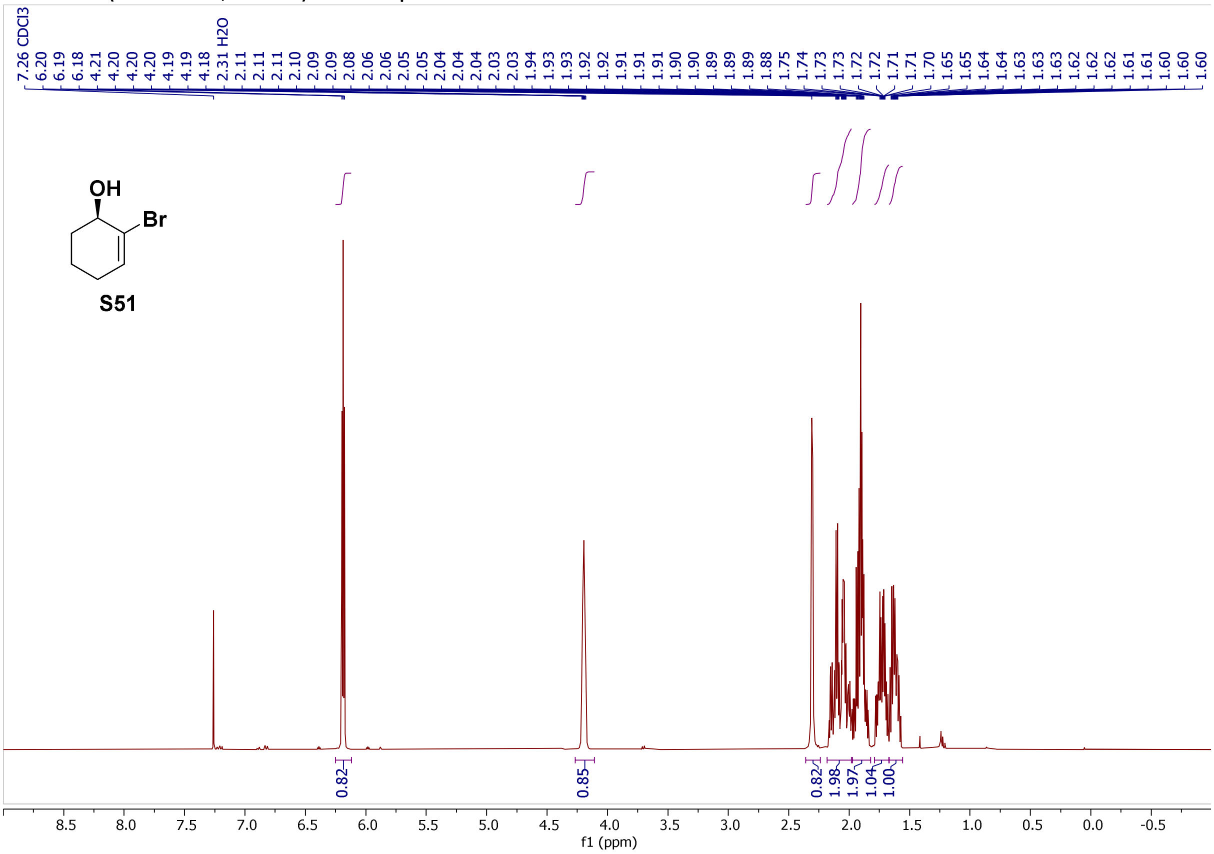

^{13}**C NMR** (101 MHz, $CDCl_3$) of compound **S51.**

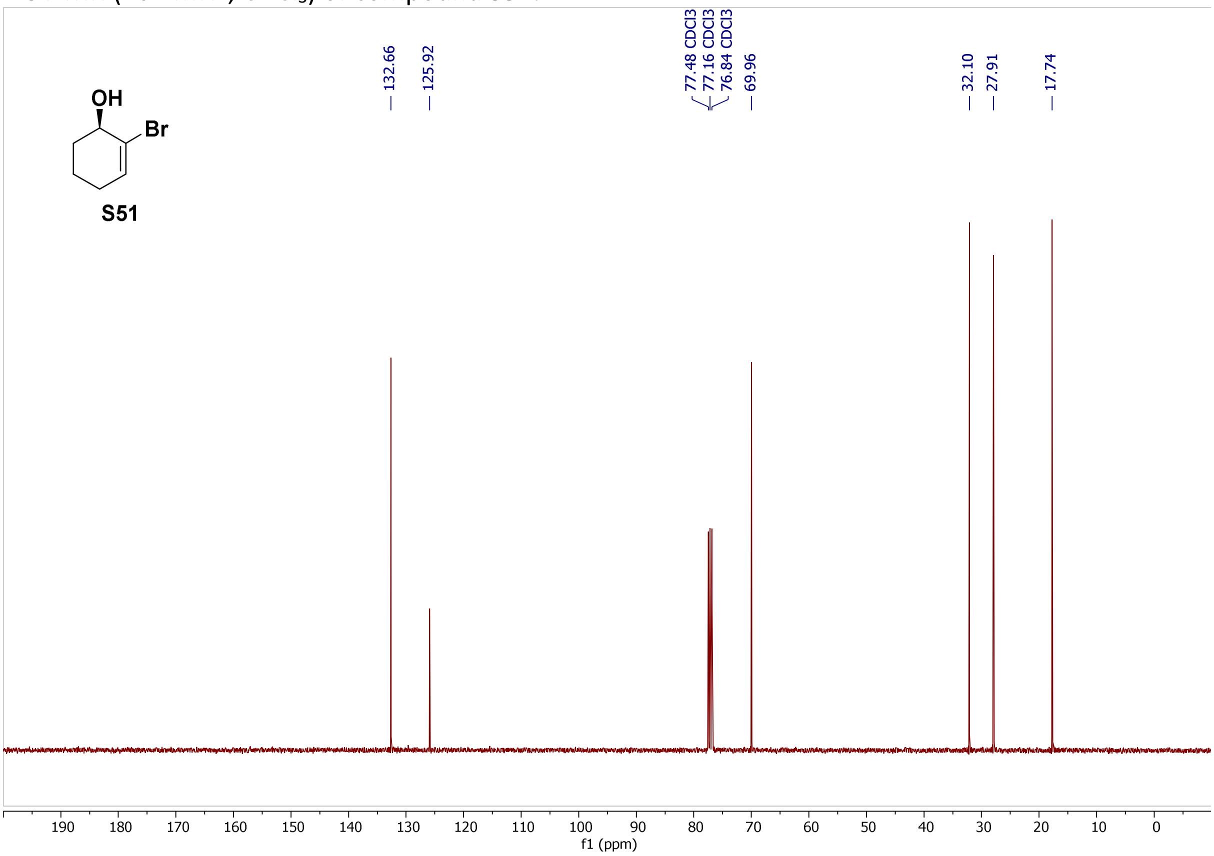

^{1}H NMR (400 MHz, $CDCl_3$) of compound **S53.**

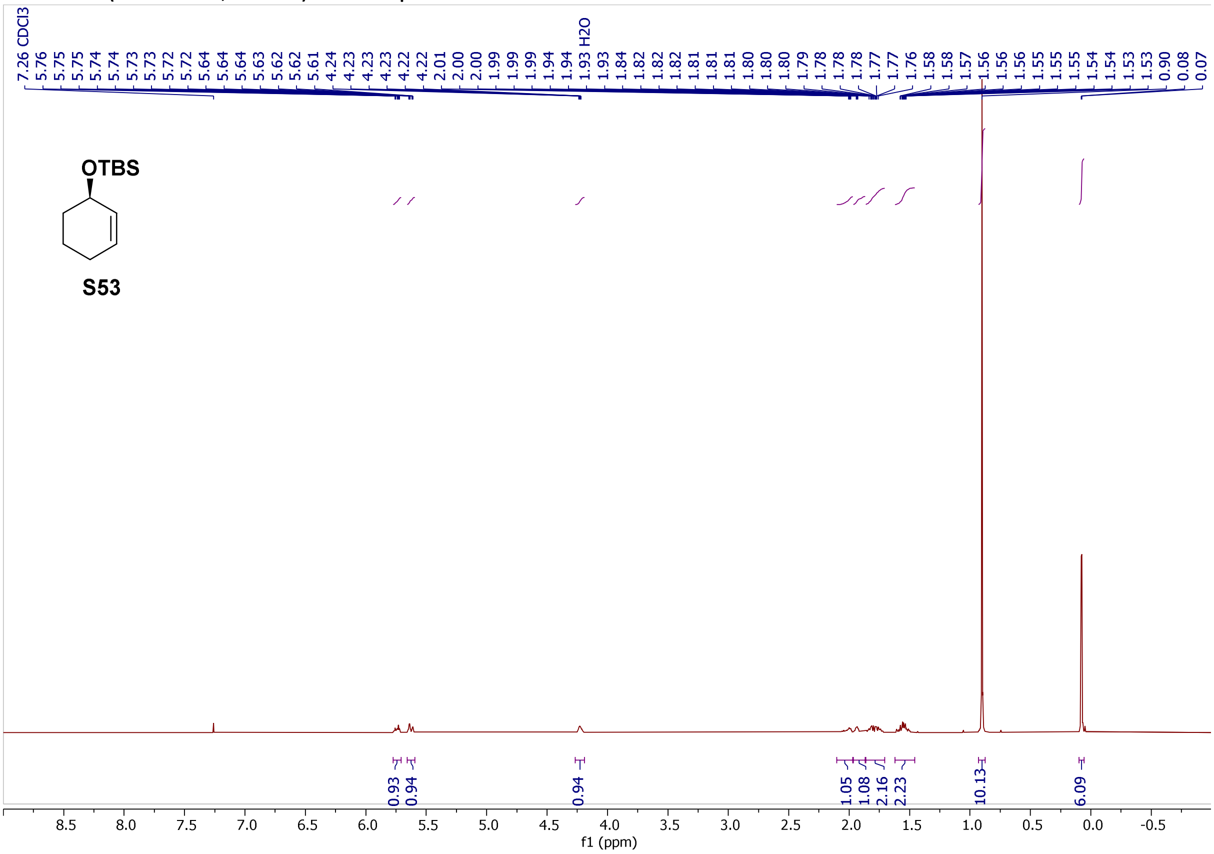

^{13}C NMR (101 MHz, $CDCl_3$) of compound **S53.**

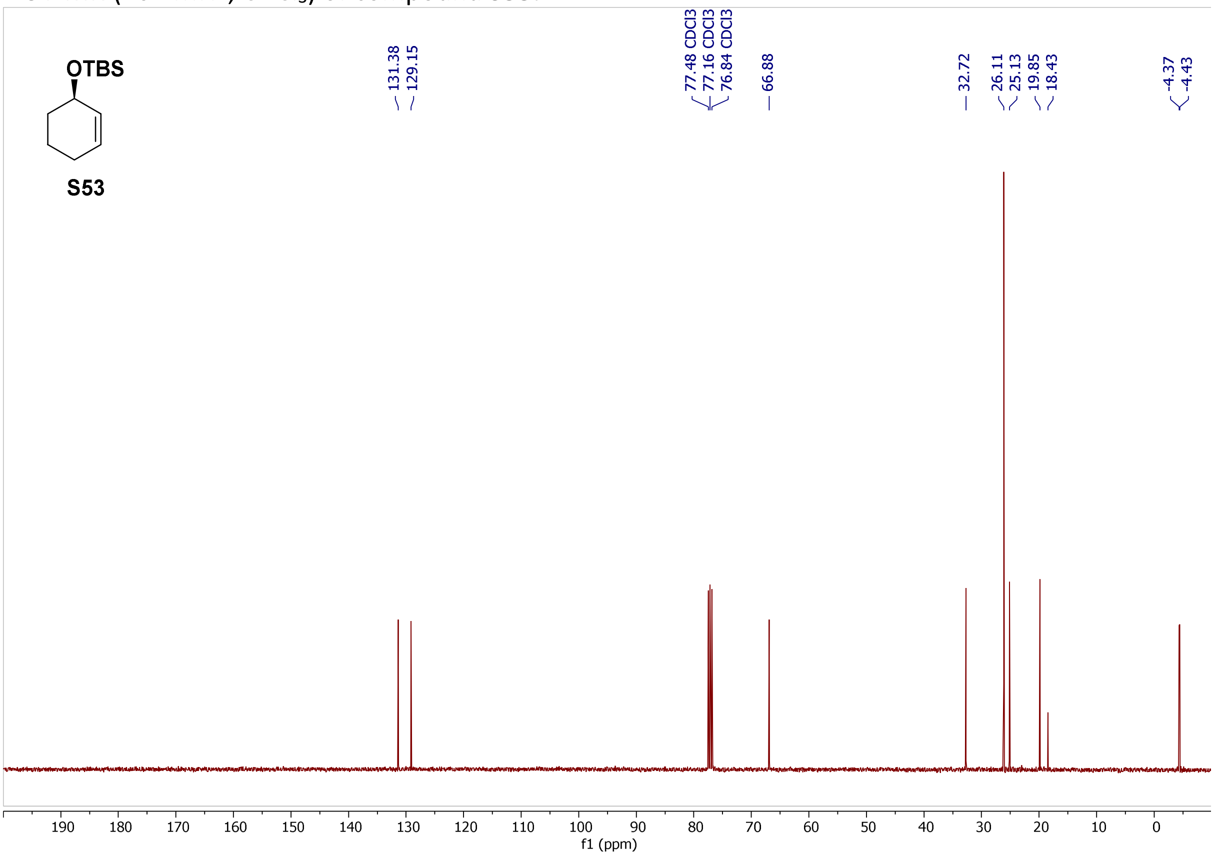

^{1}H NMR (400 MHz, $CDCl_3$) of compound **S56.**

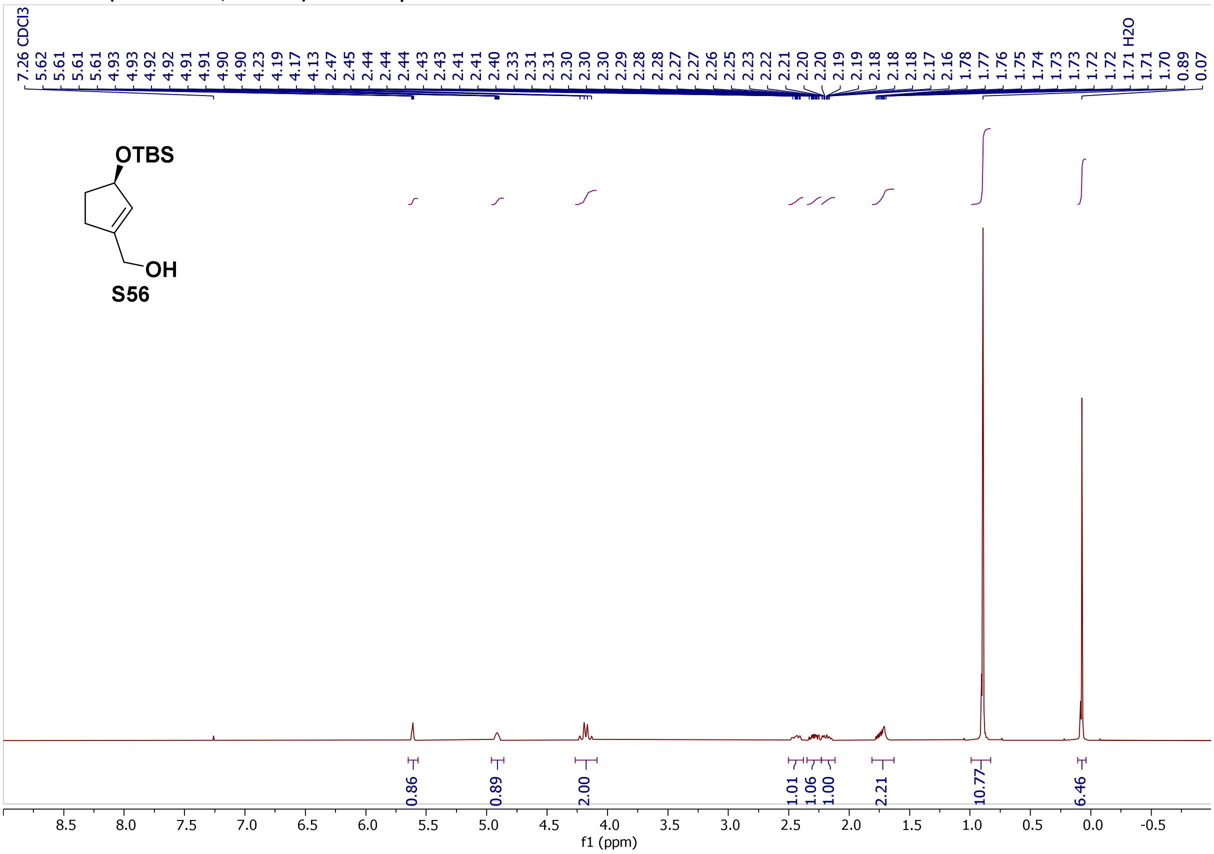

^{13}C NMR (101 MHz, $CDCl_3$) of compound **S56.**

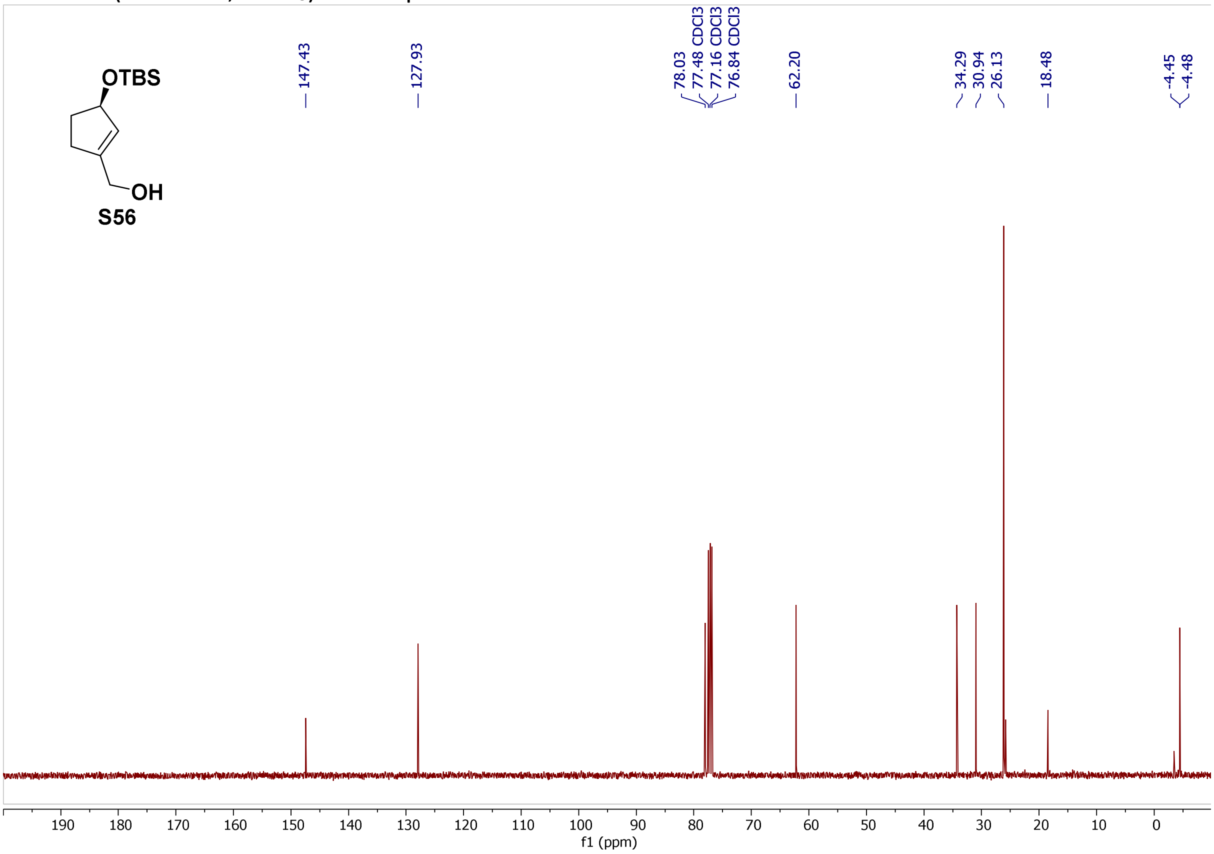

^{1}H NMR (400 MHz, C_6D_6) of compound **6.**

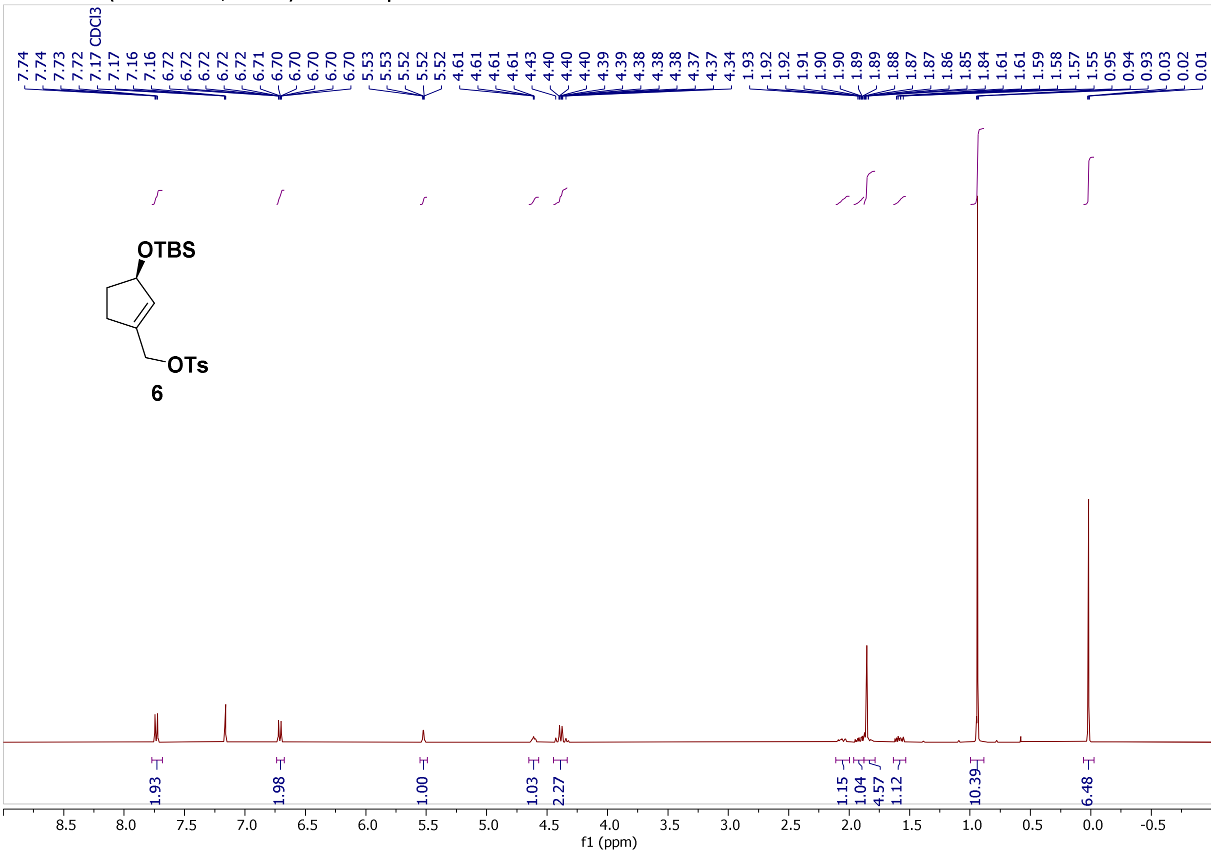

^{13}C NMR (101 MHz, C_6D_6) of compound **6.**

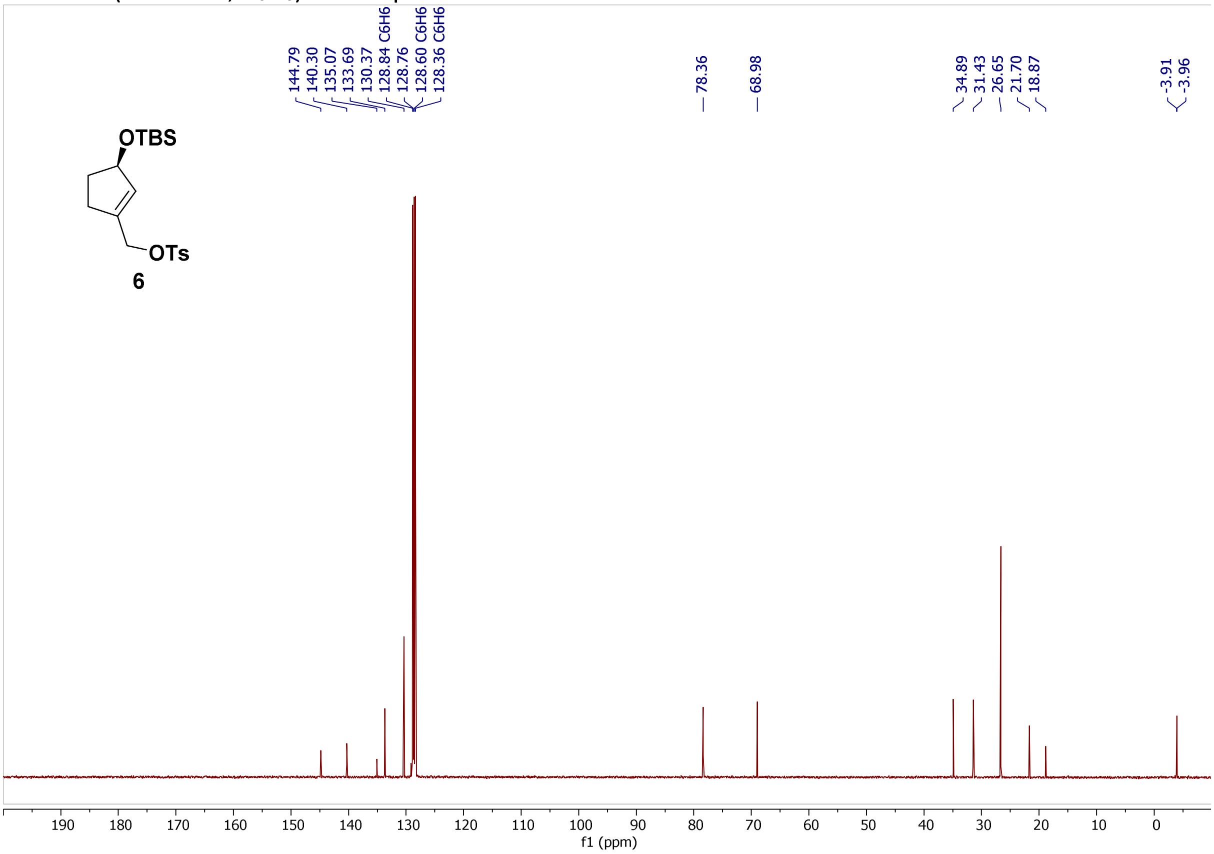

^{1}H NMR (400 MHz, $CDCl_3$) of compound **7.**

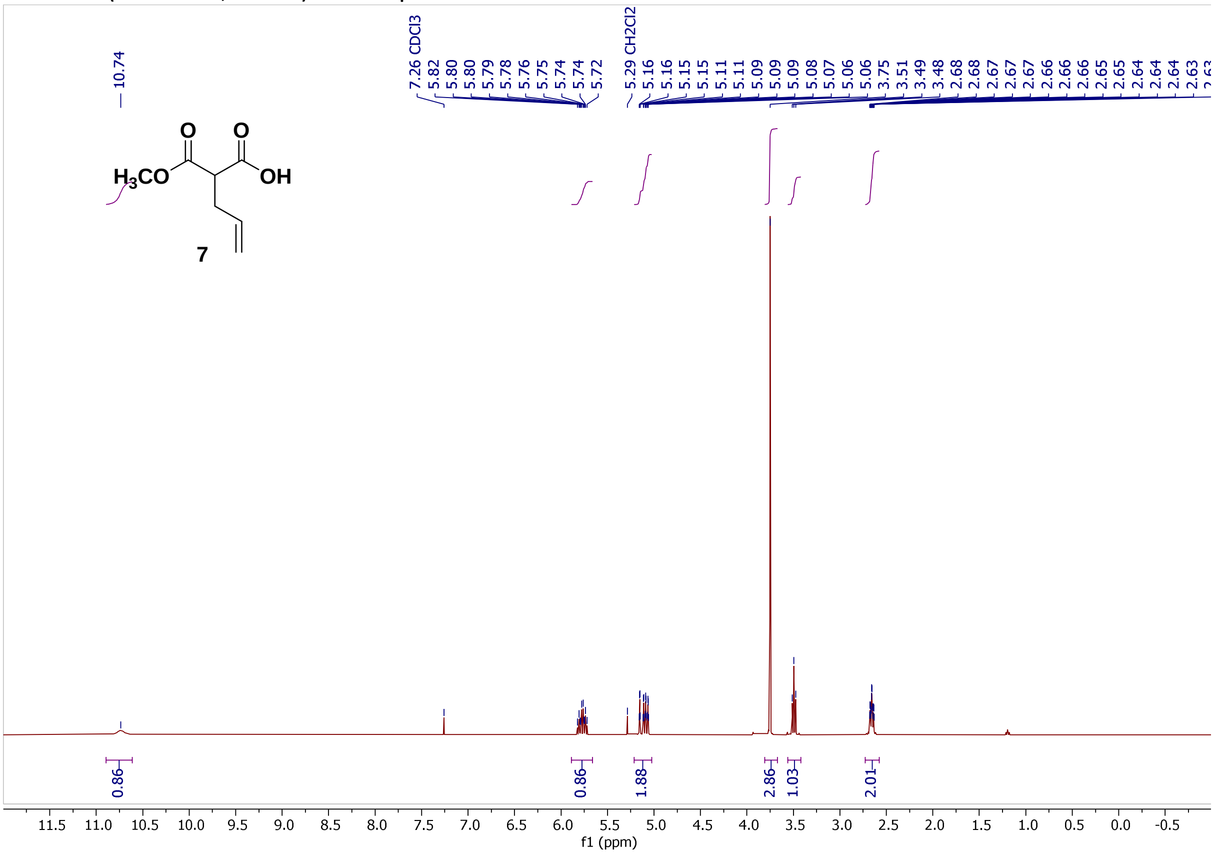

^{13}C NMR (101 MHz, $CDCl_3$) of compound **7.**

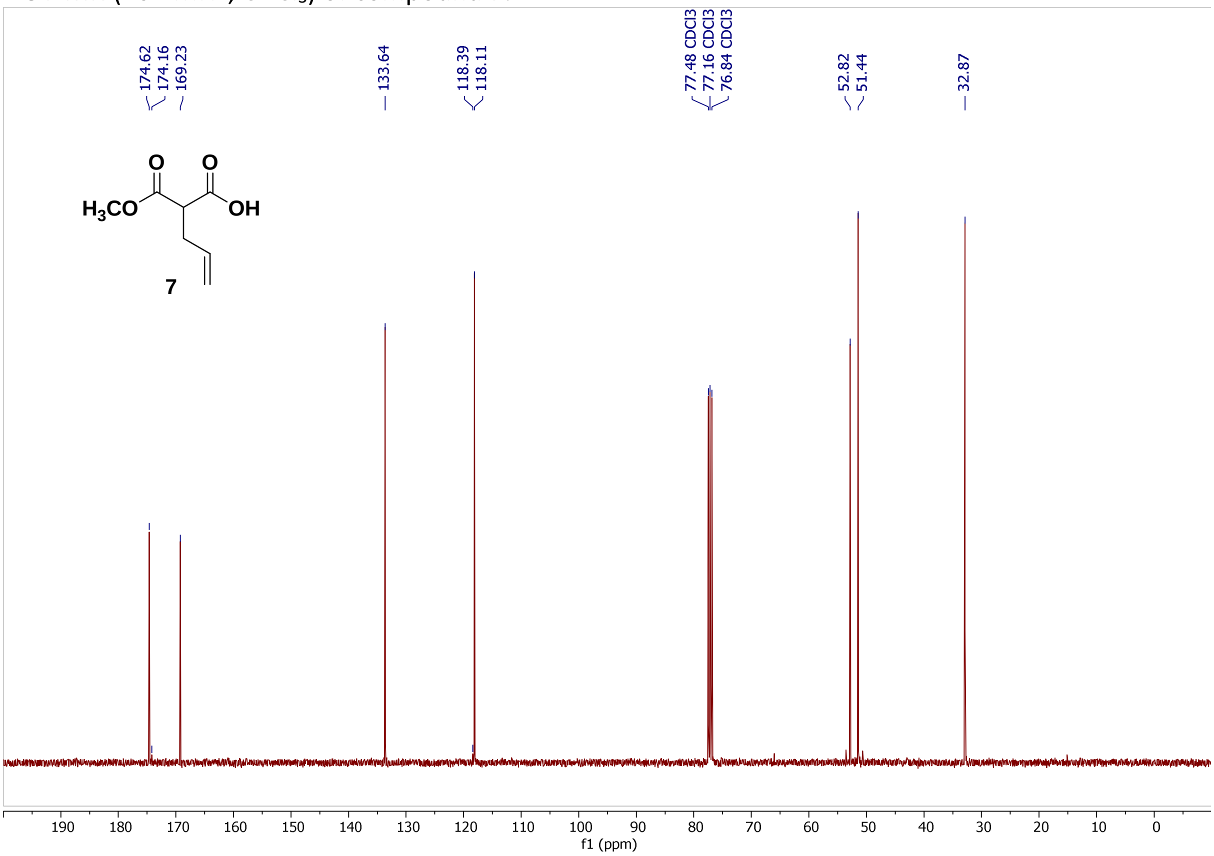

^{1}**H NMR** (400 MHz, $CDCl_3$) of compound **S58.**

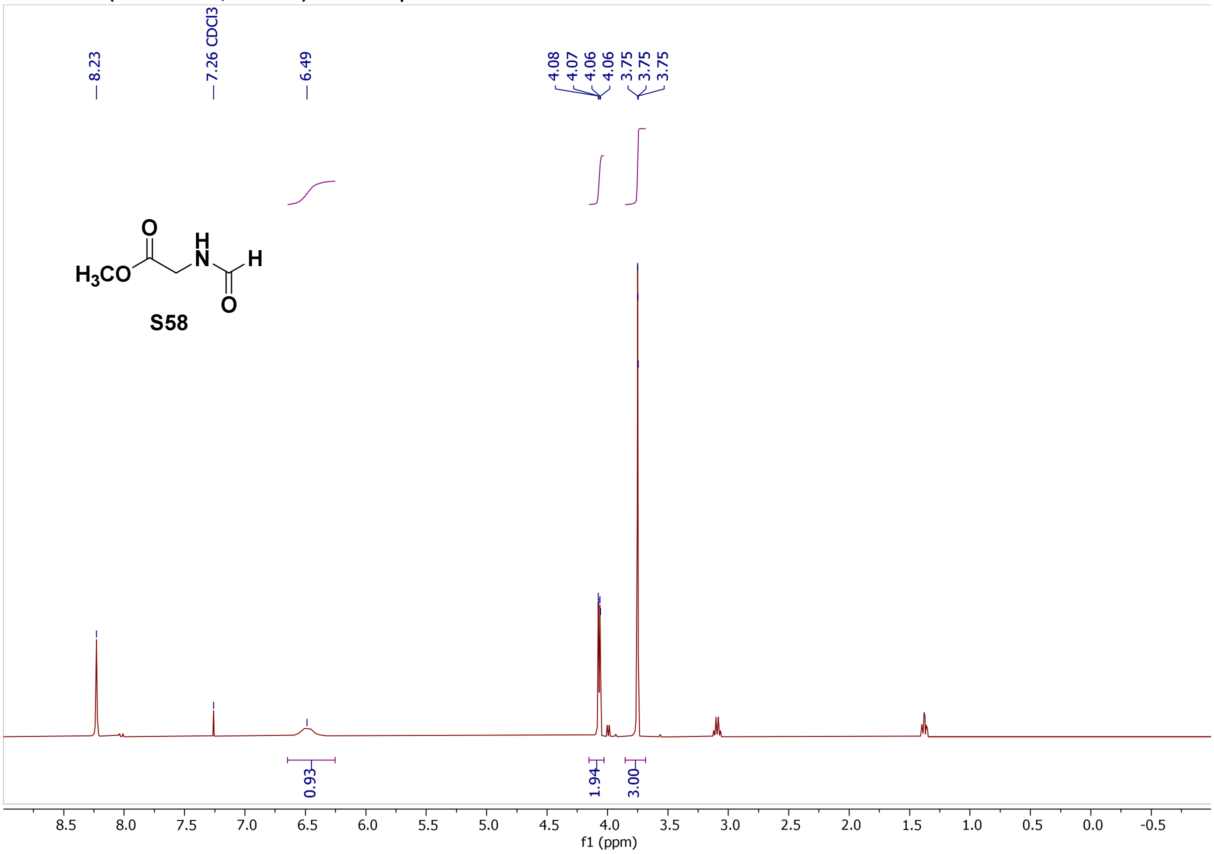

^{13}**C NMR** (101 MHz, $CDCl_3$) of compound **S58.**

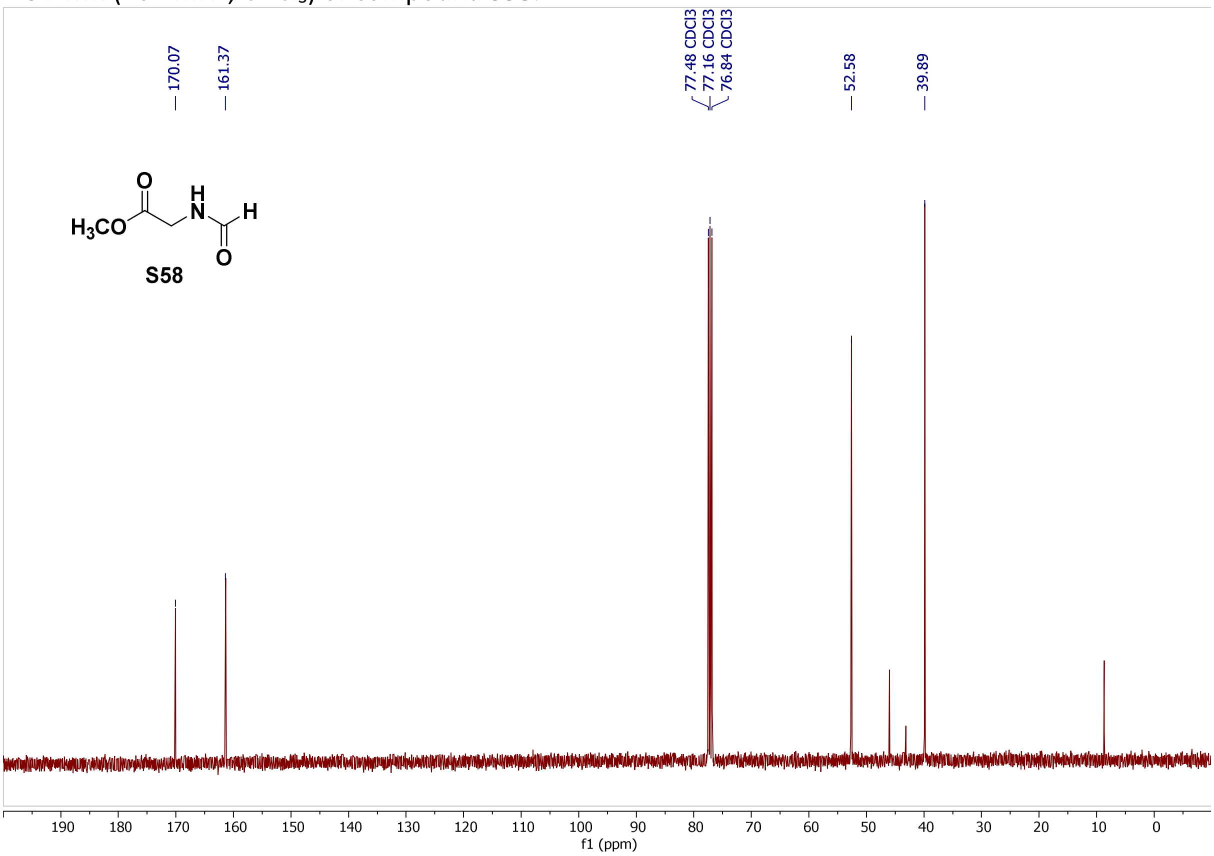

^{1}H NMR (400 MHz, $CDCl_3$) of compound **S59.**

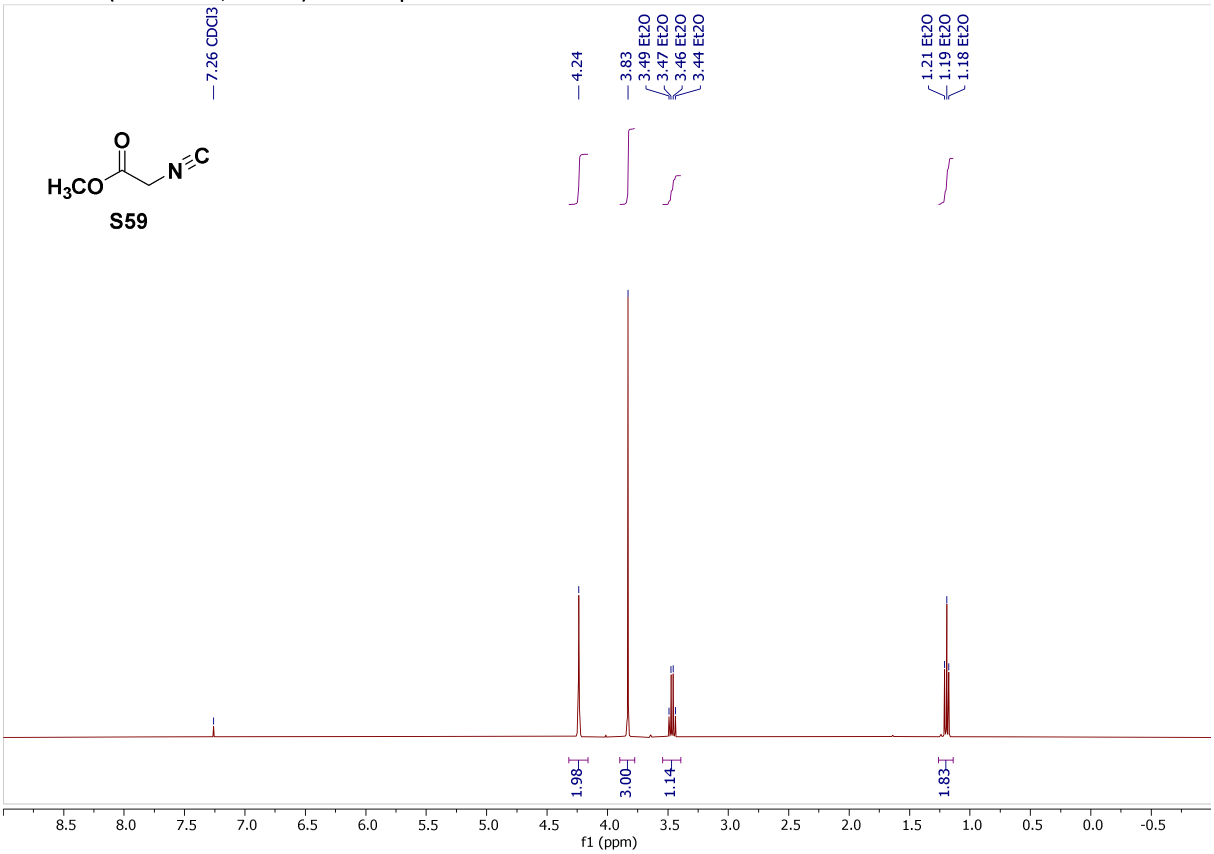

^{13}C NMR (101 MHz, $CDCl_3$) of compound **S59.**

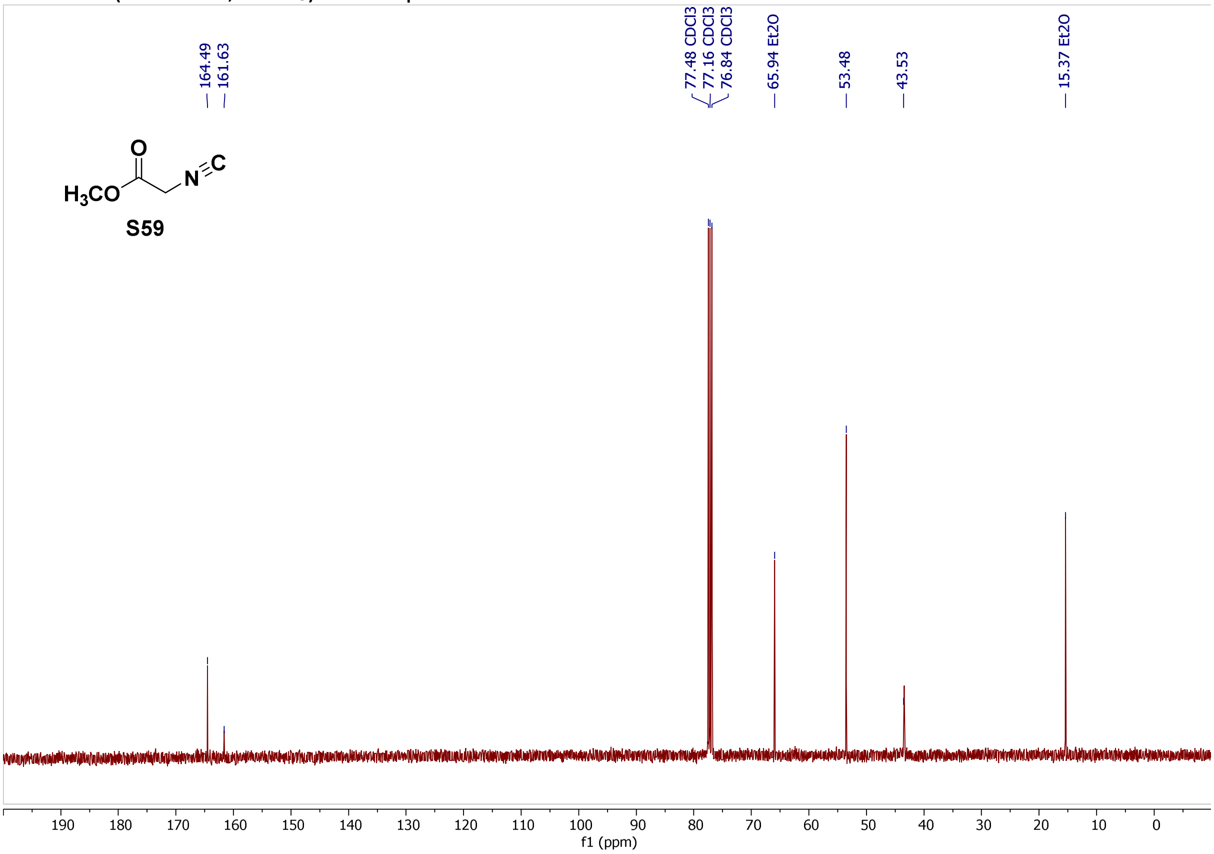

^{1}H NMR (400 MHz, DMSO) of compound **S60.**

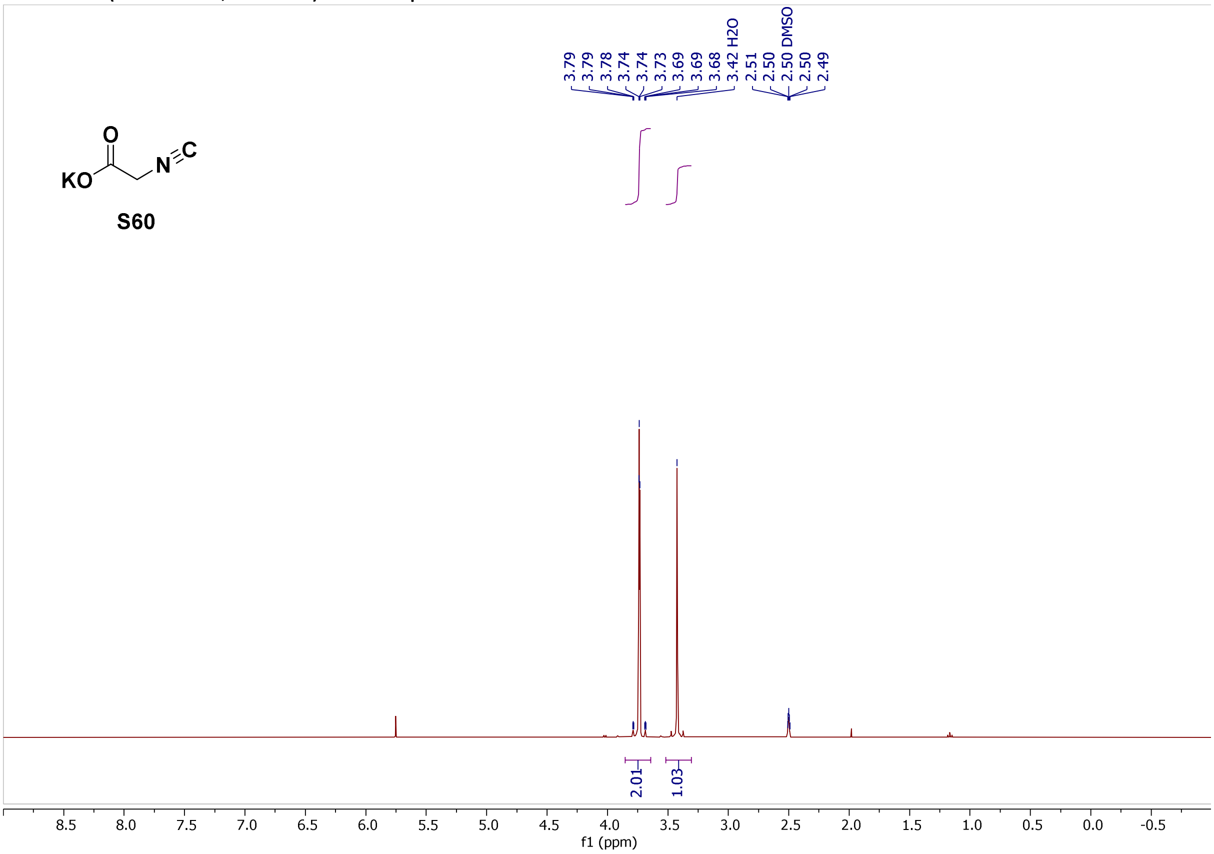

^{13}C NMR (101 MHz, DMSO) of compound **S60.**

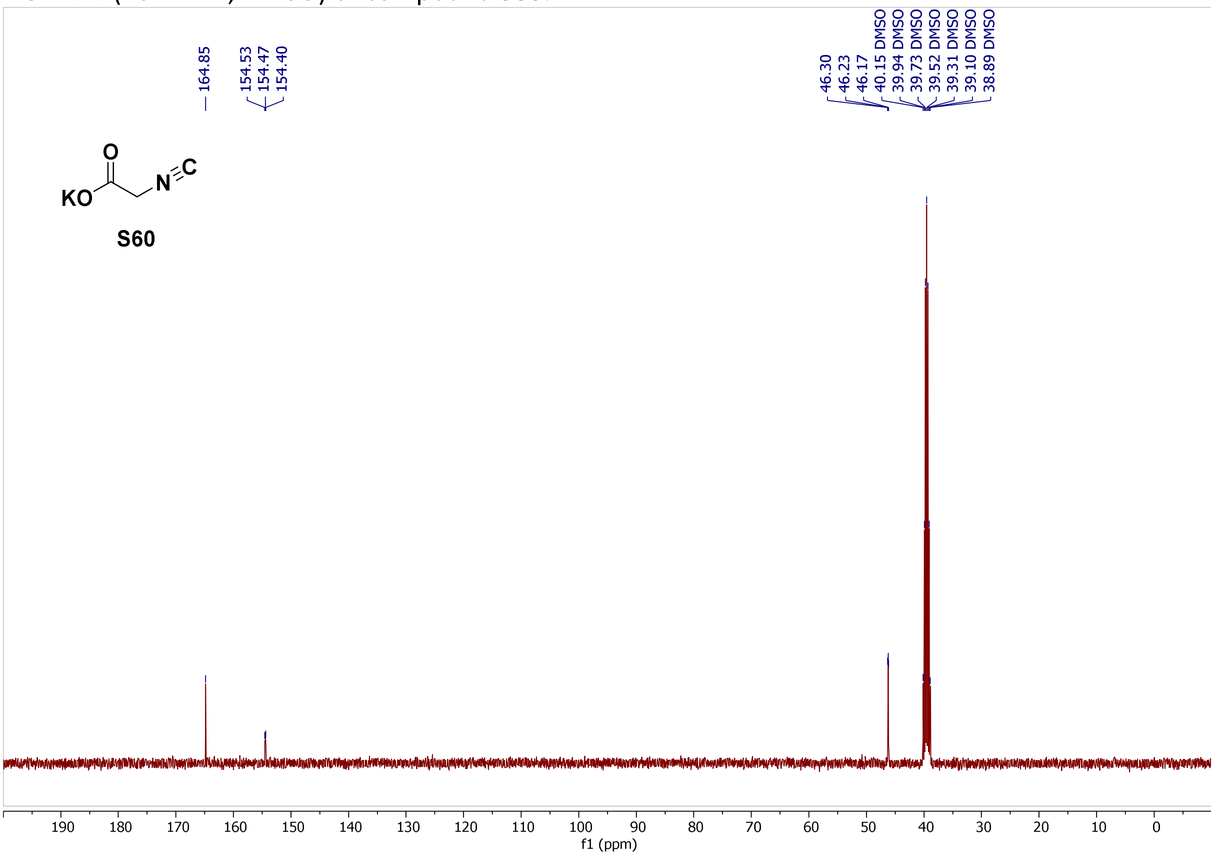

5 HPLC Traces

(*1R*)-4-methylene-2-tosyl-2-azabicyclo[3.3.1]nonan-6-one (5a)

HPLC (Chiralpak AD-H, hexane/isopropanol 90:10, 1.0 mL min^{-1}, λ =210 nm).

Racemic

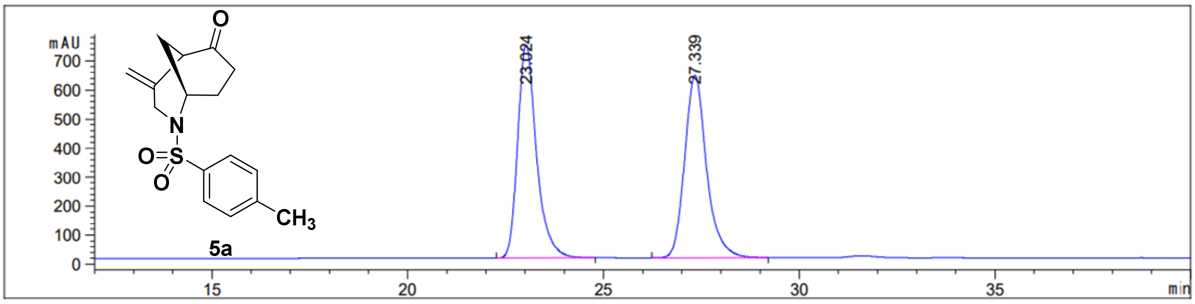

#	Time [min]	Width [min]	Area [mAU*s]	Height [mAU]	Area [%]
**1**	23.024	0.5076	2.45091e4	733.94055	49.9715
**2**	27.339	0.5962	2.45371e4	627.55475	50.0285

Enantiomerically enriched (*R,R* cat.) – 0.10 mmol scale

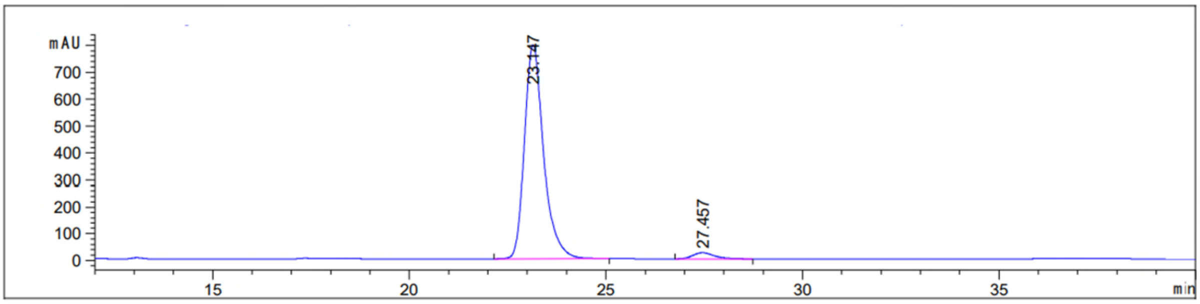

#	Time [min]	Width [min]	Area [mAU*s]	Height [mAU]	Area [%]
**1**	23.147	0.5167	2.70751e4	796.32513	96.7898
**2**	27.457	0.5861	897.98102	23.27439	3.2102

Enantiomerically enriched (*R,R* cat.) – 10.4 mmol scale

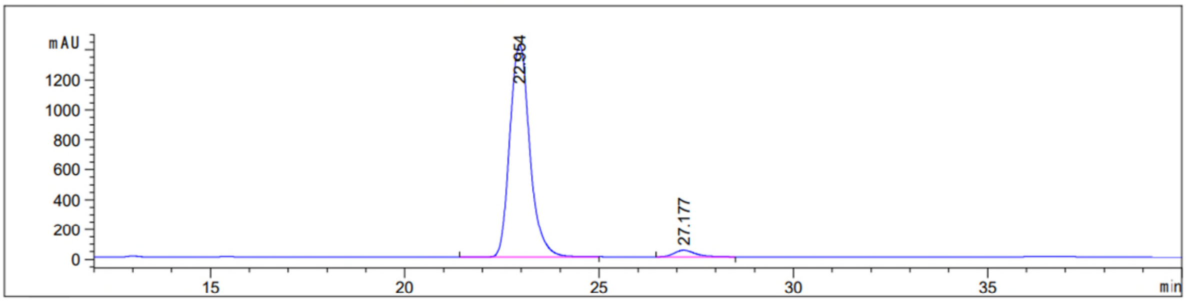

#	Time [min]	Width [min]	Area [mAU*s]	Height [mAU]	Area [%]
**1**	22.954	0.5588	5.09243e4	1418.53723	96.7869
**2**	27.177	0.5819	1690.55310	44.22779	3.2131

(*1R*)-2-((4-methoxyphenyl)sulfonyl)-4-methylene-2-azabicyclo[3.3.1]nonan-6-one (5b)

HPLC (Chiralpak AD-H, hexane/isopropanol 95:5, 1.0 mL min^{-1}, λ = 210 nm).

Racemic

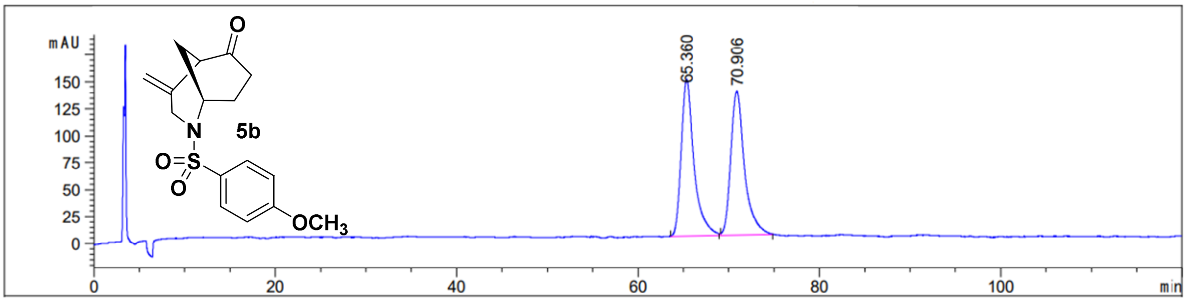

#	Time [min]	Width [min]	Area [mAU*s]	Height [mAU]	Area [%]
**1**	65.360	1.4423	1.42695e4	145.23088	50.2318
**2**	70.906	1.5308	1.41378e4	133.79330	49.7682

Enantiomerically enriched (*R,R* cat.)

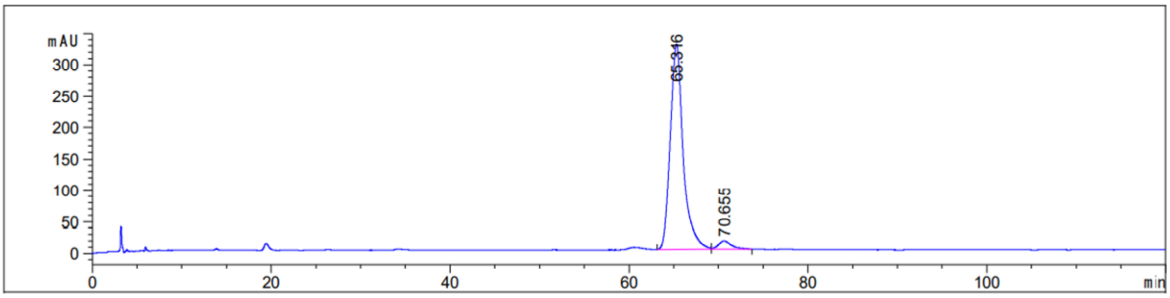

#	Time [min]	Width [min]	Area [mAU*s]	Height [mAU]	Area [%]
**1**	65.316	1.4927	3.28876e4	327.19464	95.8718
**2**	70.655	1.2397	1416.12415	13.63459	4.1282

(*1R*)-4-methylene-2-(methylsulfonyl)-2-azabicyclo[3.3.1]nonan-6-one (5c)

HPLC (Chiralpak AD-H, hexane/isopropanol 90:10, 1.0 mL min^{-1}, λ = 210 nm).

Racemate

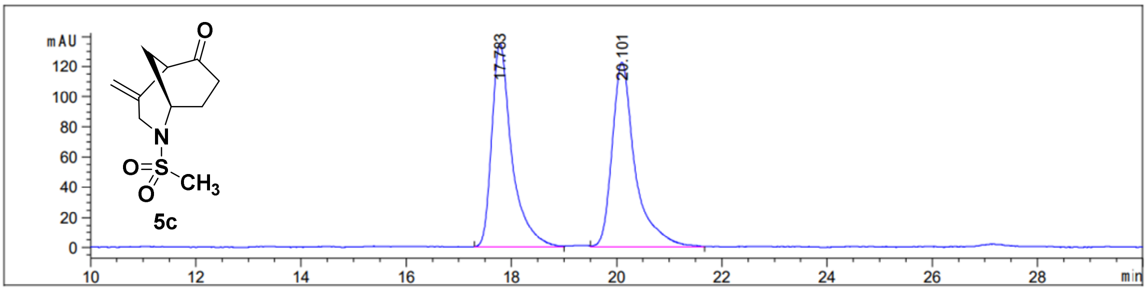

#	Time [min]	Width [min]	Area [mAU*s]	Height [mAU]	Area [%]
**1**	17.783	0.3931	3590.55322	135.51993	48.9939
**2**	20.101	0.4513	3738.02515	122.75068	51.0061

Enantiomerically enriched (*R,R* cat.)

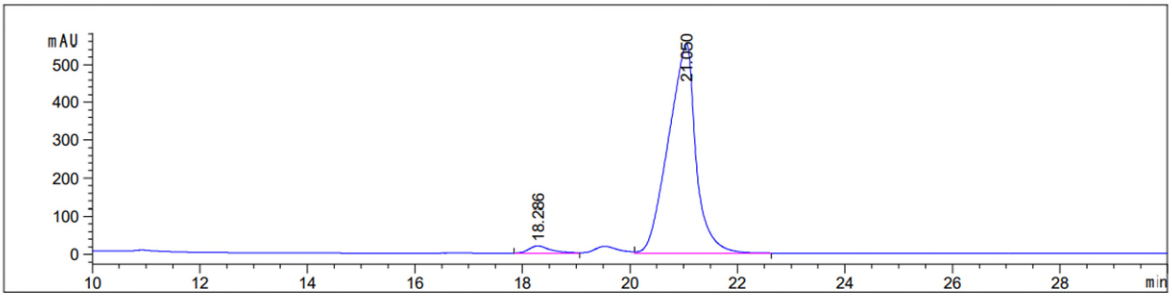

#	Time [min]	Width [min]	Area [mAU*s]	Height [mAU]	Area [%]
**1**	18.286	0.4216	553.76917	19.36860	2.7540
**2**	21.050	0.5253	1.95537e4	551.99231	97.2460

(*1R*)-2-acetyl-4-methylene-2-azabicyclo[3.3.1]nonan-6-one (5d)

HPLC (Chiralpak AS-H, hexane/isopropanol 70:30, 1.0 mL min^{-1}, λ = 210 nm).

Racemate

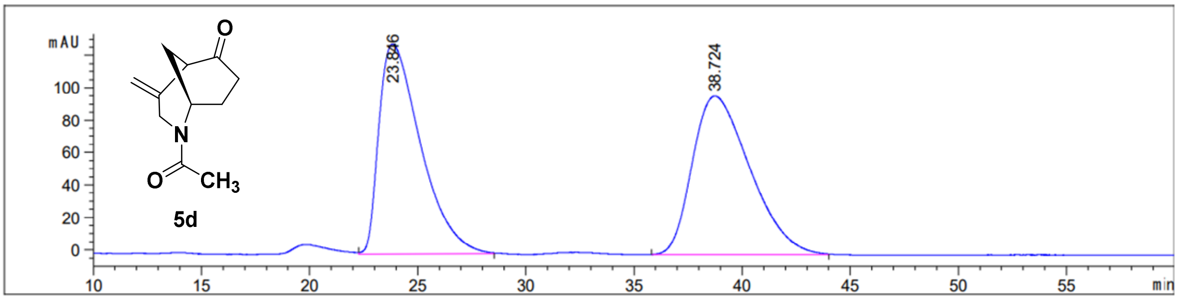

#	Time [min]	Width [min]	Area [mAU*s]	Height [mAU]	Area [%]
**1**	23.846	1.9333	1.73554e4	129.35760	48.9192
**2**	38.724	2.2011	1.81223e4	97.83450	51.0808

Enantiomerically enriched (*R,R* cat.)

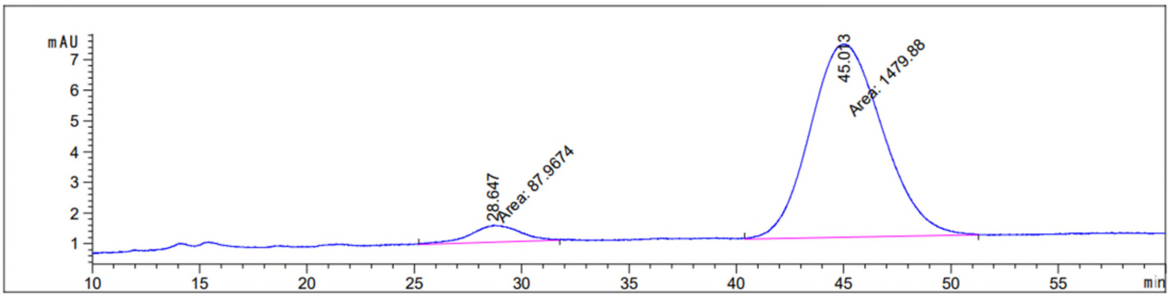

#	Time [min]	Width [min]	Area [mAU*s]	Height [mAU]	Area [%]
**1**	28.647	2.7007	87.96741	5.42875e-1	5.6107
**2**	45.013	3.9160	1479.88232	6.29847	94.3893

***tert*-butyl (*1R*)-4-methylene-6-oxo-2-azabicyclo[3.3.1]nonane-2-carboxylate (5e)**

HPLC (Chiralpak AD-H, hexane/isopropanol 98:2, 1.0 mL min^{-1}, λ = 210 nm).

Racemate

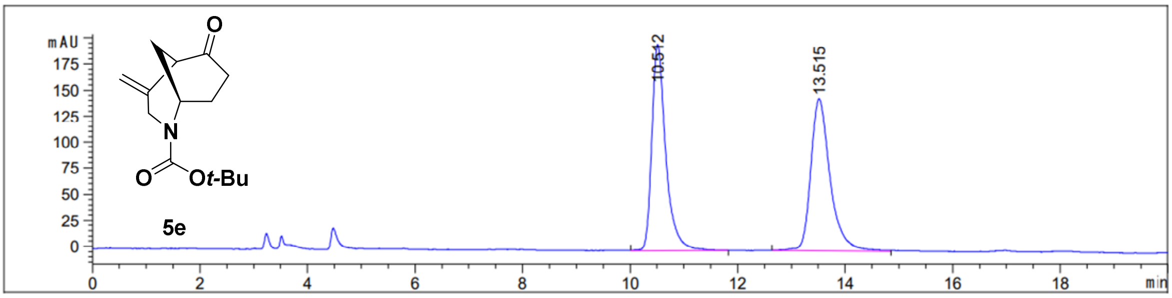

#	Time [min]	Width [min]	Area [mAU*s]	Height [mAU]	Area [%]
**1**	10.512	0.2702	3557.73755	197.50075	49.2704
**2**	13.515	0.3790	3663.11035	145.84015	50.7296

Enantiomerically enriched (*R,R* cat.)

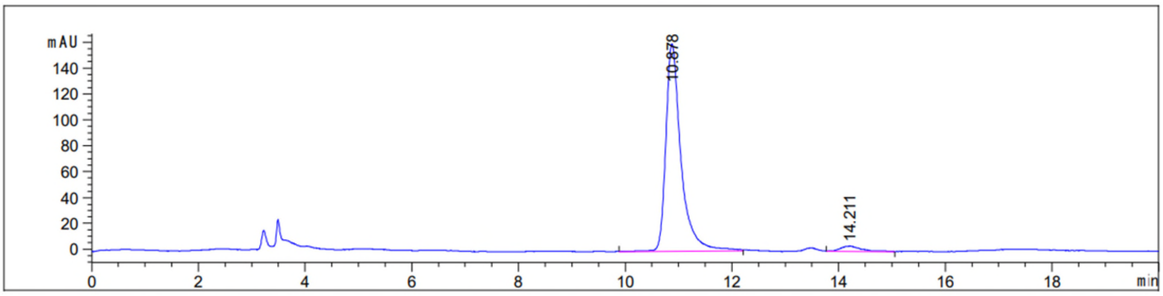

#	Time [min]	Width [min]	Area [mAU*s]	Height [mAU]	Area [%]
**1**	10.878	0.2924	3168.86548	160.42641	96.3355
**2**	14.211	0.3539	120.53999	4.21210	3.6645

benzyl (*1R*)-4-methylene-6-oxo-2-azabicyclo[3.3.1]nonane-2-carboxylate (5f)

HPLC (Chiralpak AD-H, hexane/isopropanol 98:2, 1.0 mL min^{-1}, λ = 210 nm).

Racemate

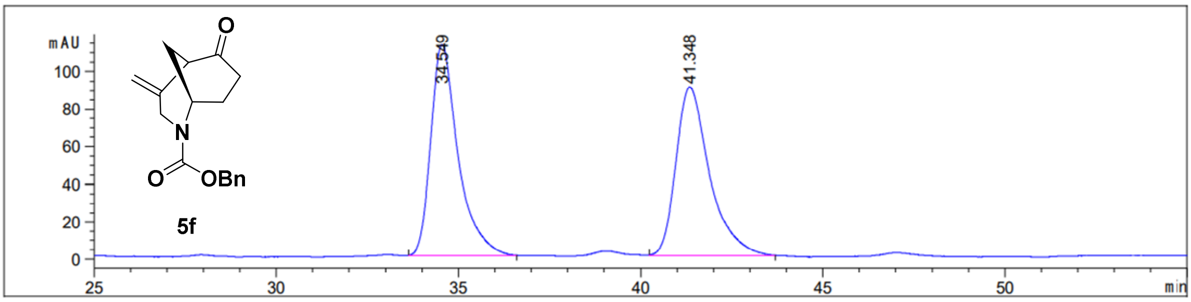

#	Time [min]	Width [min]	Area [mAU*s]	Height [mAU]	Area [%]
**1**	34.549	0.7782	5877.90576	112.38127	50.2942
**2**	41.348	0.9620	5809.13525	90.12437	49.7058

Enantiomerically enriched (*R,R* cat.)

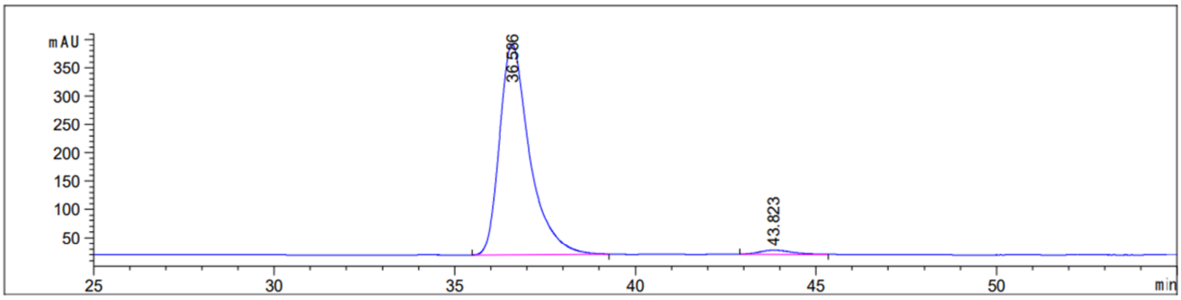

#	Time [min]	Width [min]	Area [mAU*s]	Height [mAU]	Area [%]
**1**	36.586	0.8672	2.15623e4	372.59464	98.0051
**2**	43.823	0.7355	438.89465	7.11695	1.9949

(*1R*)-2-benzyl-4-methylene-2-azabicyclo[3.3.1]nonan-6-one (5g)

HPLC (Chiralpak AD-H, hexane/isopropanol 98:2, 1.0 mL min^{-1}, λ = 210 nm).

Racemate

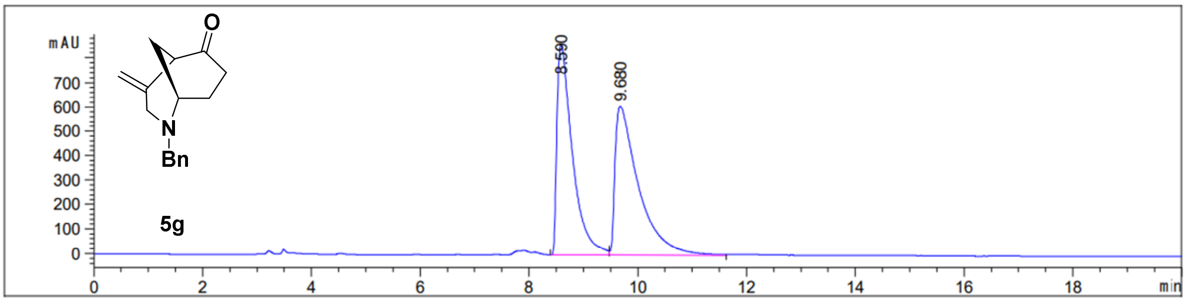

#	Time [min]	Width [min]	Area [mAU*s]	Height [mAU]	Area [%]
**1**	8.590	0.2980	1.71048e4	860.10712	48.2704
**2**	9.680	0.4304	1.83306e4	610.36389	51.7296

Enantiomerically enriched (*R,R* cat.)

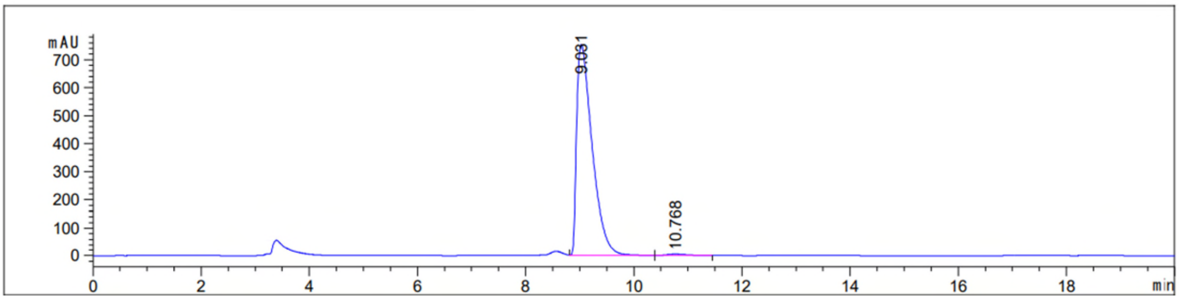

#	Time [min]	Width [min]	Area [mAU*s]	Height [mAU]	Area [%]
**1**	9.031	0.3034	1.50856e4	754.19440	99.0566
**2**	10.768	0.3970	143.66962	5.45986	0.9434

(*1R*)-2-methyl-4-methylene-1-phenyl-2-azabicyclo[3.3.1]nonan-6-one (5h)

HPLC (Chiralpak OD-H, hexane/isopropanol 99:1, 1.0 mL min^{-1}, λ = 220 nm).

Racemate

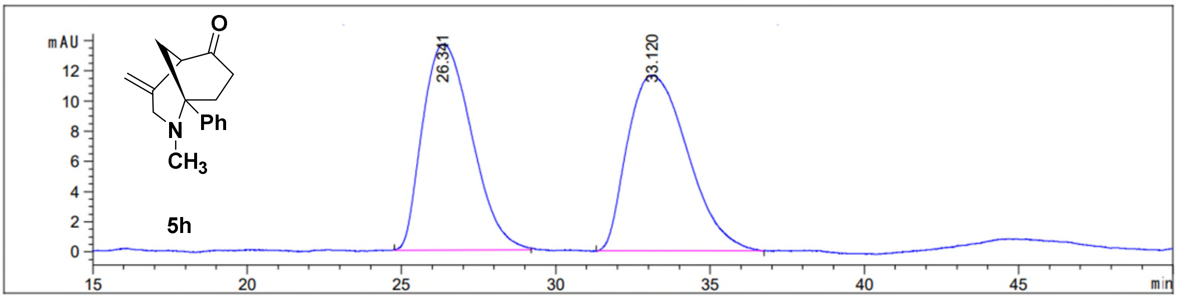

#	Time [min]	Width [min]	Area [mAU*s]	Height [mAU]	Area [%]
**1**	26.341	1.3392	1510.10034	13.64471	48.8935
**2**	33.120	1.5980	1578.44824	11.64054	51.1065

Enantiomerically enriched (*R,R* cat.)

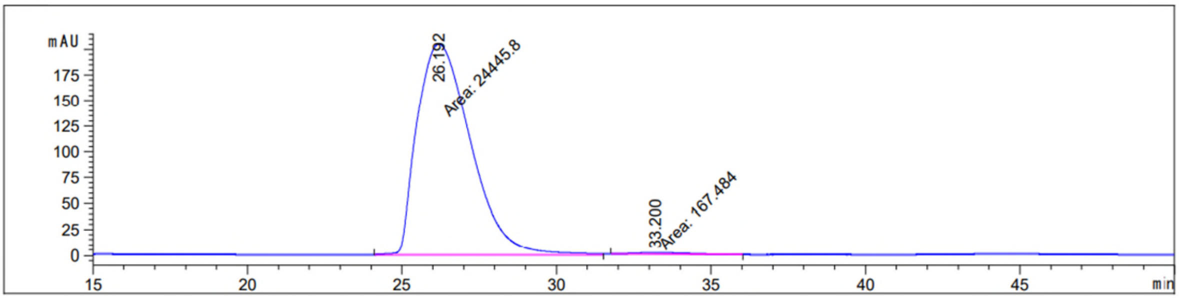

#	Time [min]	Width [min]	Area [mAU*s]	Height [mAU]	Area [%]
**1**	26.192	1.9890	2.44458e4	204.84531	99.3195
**2**	33.200	1.9868	167.48372	1.40498	0.6805

(*1R*)-2-benzyl-4-methylene-1-phenyl-2-azabicyclo[3.3.1]nonan-6-one (5i)

HPLC (Chiralpak AD-H, hexane/isopropanol 98:2, 1.0 mL min^{-1}, λ = 254 nm).

Racemate

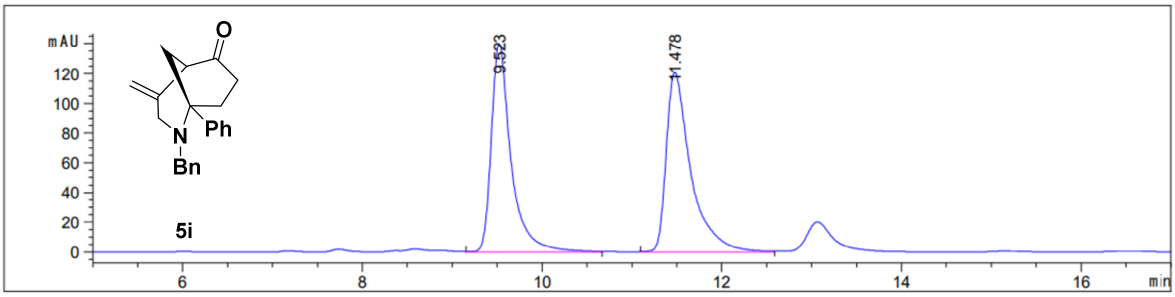

#	Time [min]	Width [min]	Area [mAU*s]	Height [mAU]	Area [%]
**1**	9.523	0.2269	2142.96729	139.77252	48.2781
**2**	11.478	0.2819	2295.83301	120.72227	51.7219

Enantiomerically enriched (*R,R* cat.)

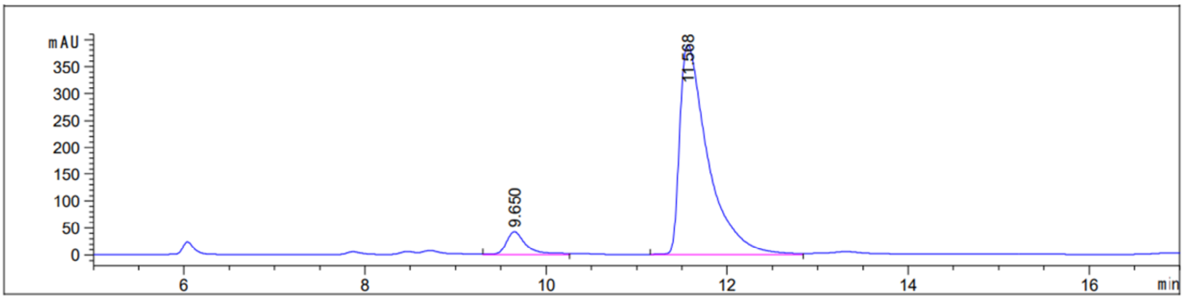

#	Time [min]	Width [min]	Area [mAU*s]	Height [mAU]	Area [%]
**1**	9.650	0.2285	656.35748	42.41661	7.2138
**2**	11.568	0.3206	8442.21680	389.86115	92.7862

(*1R*)-4-((*E*)-benzylidene)-2-tosyl-2-azabicyclo[3.3.1]nonan-6-one (5j)

HPLC (Chiralpak IA, hexane/isopropanol 80:20, 1.0 mL min^{-1}, λ = 220 nm).

Racemate

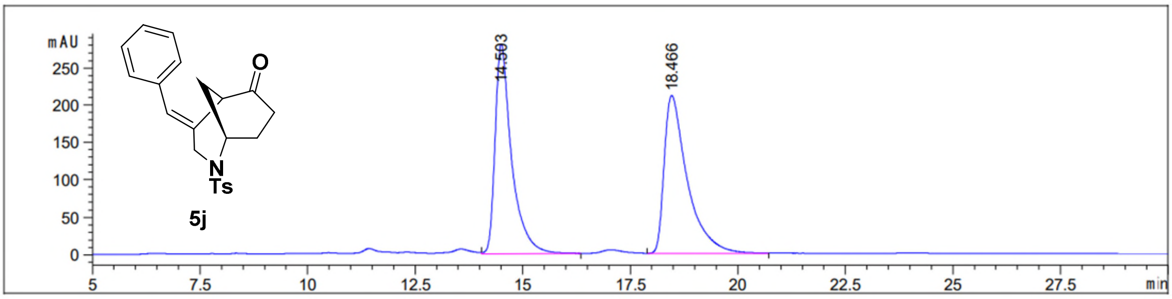

#	Time [min]	Width [min]	Area [mAU*s]	Height [mAU]	Area [%]
**1**	14.503	0.3953	7467.72754	279.85507	48.6853
**2**	18.466	0.5528	7871.03223	210.24707	51.3147

Enantiomerically enriched (*R,R* cat.)

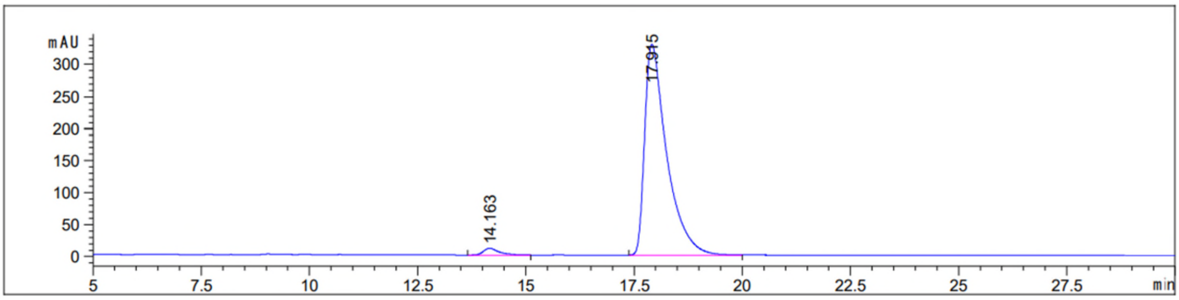

#	Time [min]	Width [min]	Area [mAU*s]	Height [mAU]	Area [%]
**1**	14.163	0.3741	274.07452	10.72707	2.2919
**2**	17.915	0.5261	1.16845e4	329.21753	97.7081

(*1R*)-4-((*E*)-4-methoxybenzylidene)-2-tosyl-2-azabicyclo[3.3.1]nonan-6-one (5k)

HPLC (Chiralpak IA, hexane/isopropanol 80:20, 1.0 mL min^{-1}, λ = 220 nm).

Racemate

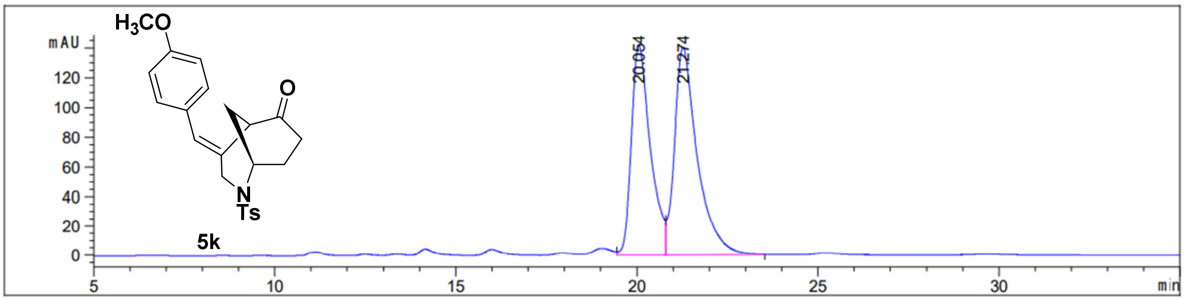

#	Time [min]	Width [min]	Area [mAU*s]	Height [mAU]	Area [%]
**1**	20.054	0.5455	5176.96338	141.96077	45.6886
**2**	21.274	0.6399	6154.01660	140.26508	54.3114

Enantiomerically enriched (*R,R* cat.)

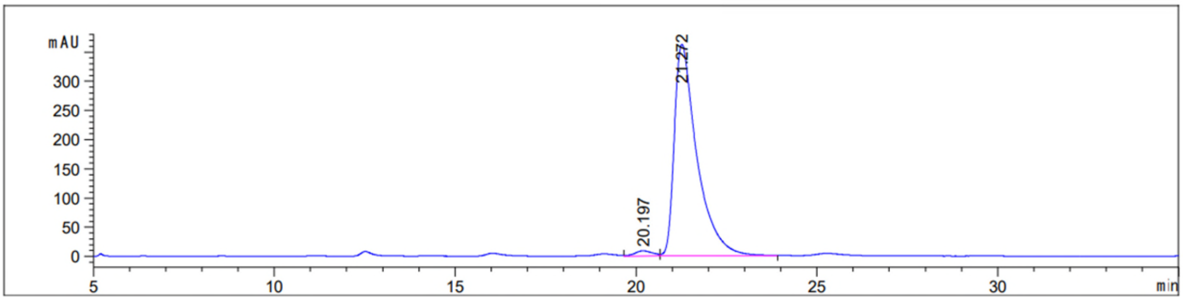

#	Time [min]	Width [min]	Area [mAU*s]	Height [mAU]	Area [%]
**1**	20.197	0.5127	307.29544	8.94699	1.8981
**2**	21.272	0.6424	1.58825e4	363.05569	98.1019

(*1R,E*)-4-butylidene-2-tosyl-2-azabicyclo[3.3.1]nonan-6-one (5l)

HPLC (Chiralcel OD, hexane/isopropanol 90:10, 1.0 mL min^{-1}, λ = 210 nm).

Racemate

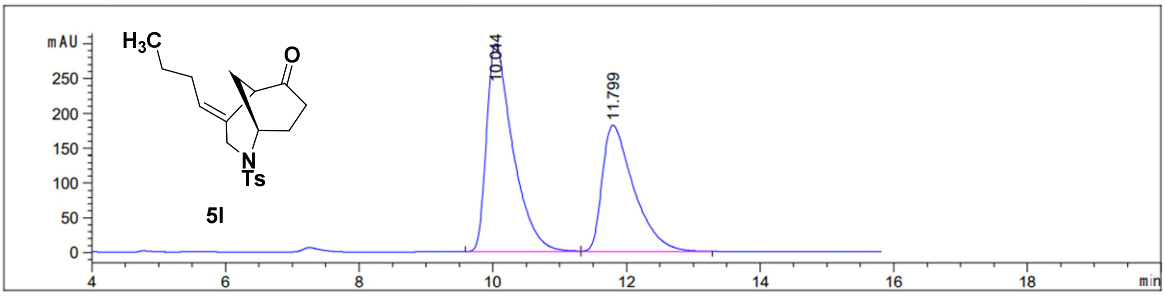

#	Time [min]	Width [min]	Area [mAU*s]	Height [mAU]	Area [%]
**1**	10.044	0.4014	8181.82813	298.82294	57.7883
**2**	11.799	0.4884	5976.44385	181.39310	42.2117

Enantiomerically enriched (*R,R* cat.)

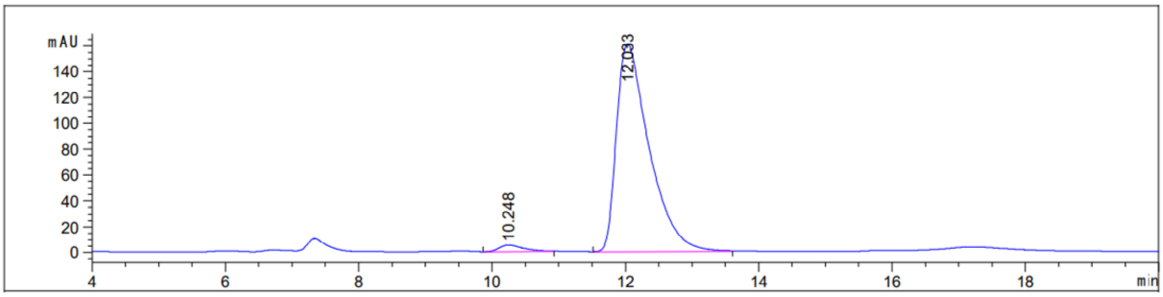

#	Time [min]	Width [min]	Area [mAU*s]	Height [mAU]	Area [%]
**1**	10.248	0.3766	135.53535	5.22473	2.4119
**2**	12.033	0.5051	5483.95215	161.11551	97.5881

Enantiomerically enriched (*S,S* cat.)

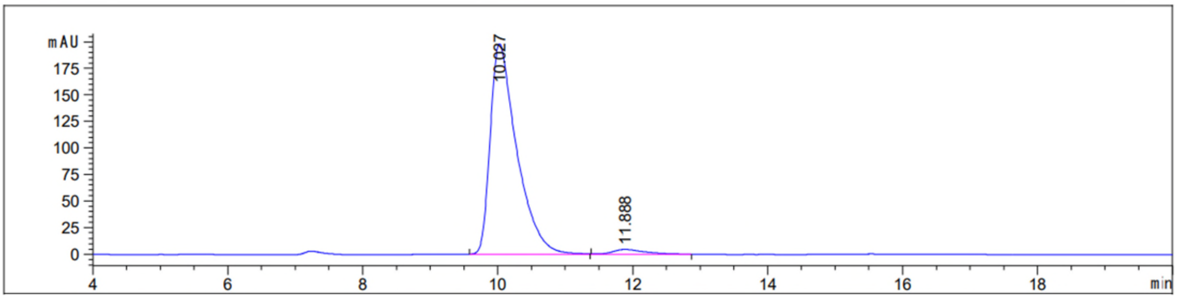

#	Time [min]	Width [min]	Area [mAU*s]	Height [mAU]	Area [%]
**1**	10.027	0.4005	5401.61963	197.84512	97.0230
**2**	11.888	0.5041	165.74120	4.69201	2.9770

***(R)*-2-bromocyclohex-2-en-1-ol (S51)**

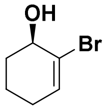

S51

HPLC (Chiralcel AD-H, hexane/isopropanol 95:5, 1.0 mL min^{-1}, λ = 220 nm).

Racemate

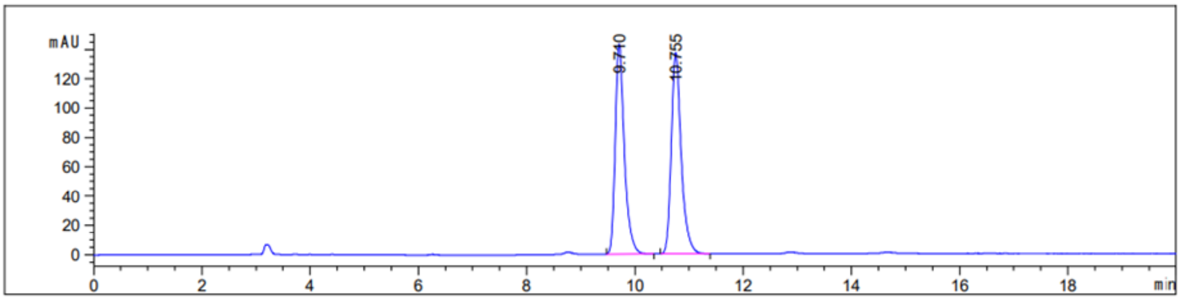

#	Time [min]	Width [min]	Area [mAU*s]	Height [mAU]	Area [%]
**1**	9.710	0.1757	1667.76904	143.38470	48.8135
**2**	10.755	0.1924	1748.84326	137.46294	51.1865

Enantiomerically enriched

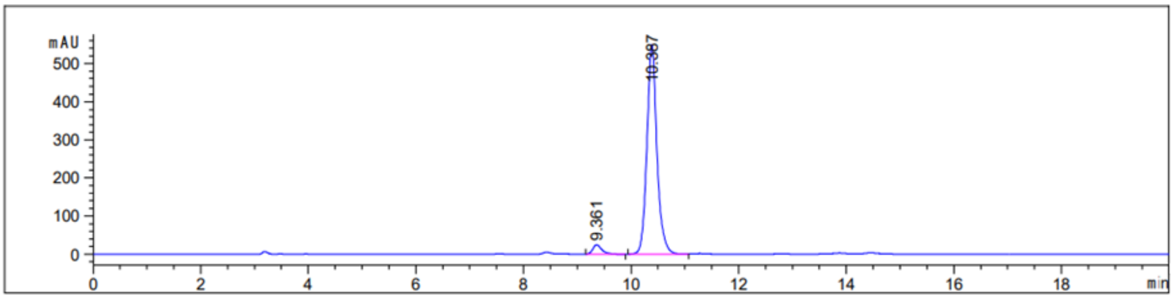

#	Time [min]	Width [min]	Area [mAU*s]	Height [mAU]	Area [%]
**1**	9.361	0.1727	284.00568	24.60742	3.8399
**2**	10.387	0.1950	7112.13379	549.53589	96.1601

(*1R,4S,5R*)-4,8-dimethyl-2-tosyl-2-azabicyclo[3.3.1]non-7-en-6-one (18)

SFC (Chiralpak® IA, 1500 psi, 30° C, flow: 1.5 mL min^{-1}, from 1% to 30% MeOH in 5 min).

Racemate

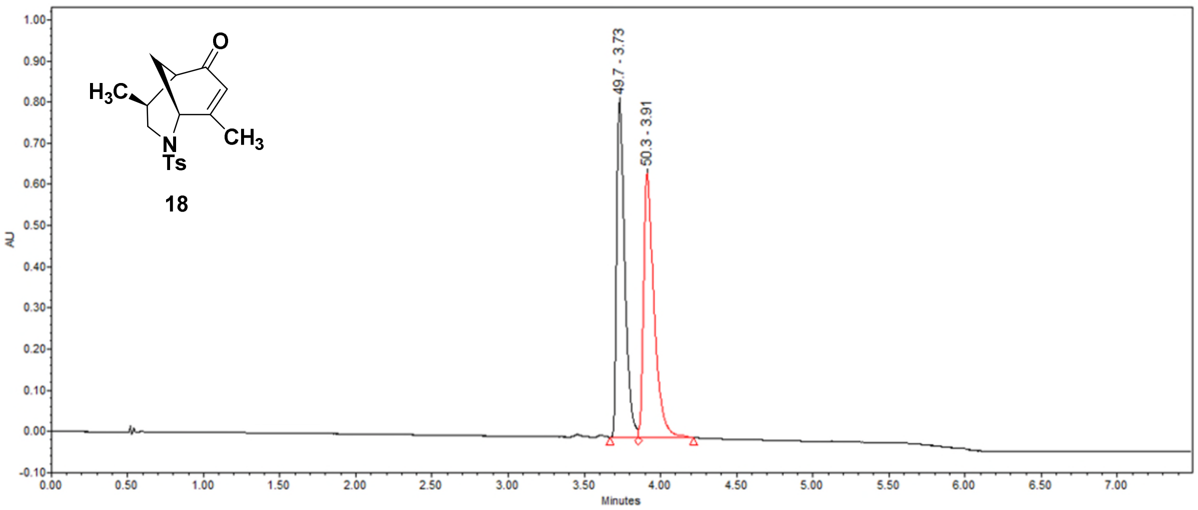

#	Time [min]	Area [µV.sec]	Height [µV]	Area [%]
**1**	3.731	3077690	814086	49.70
**2**	3.912	3114354	640877	50.30

Enantiomerically enriched

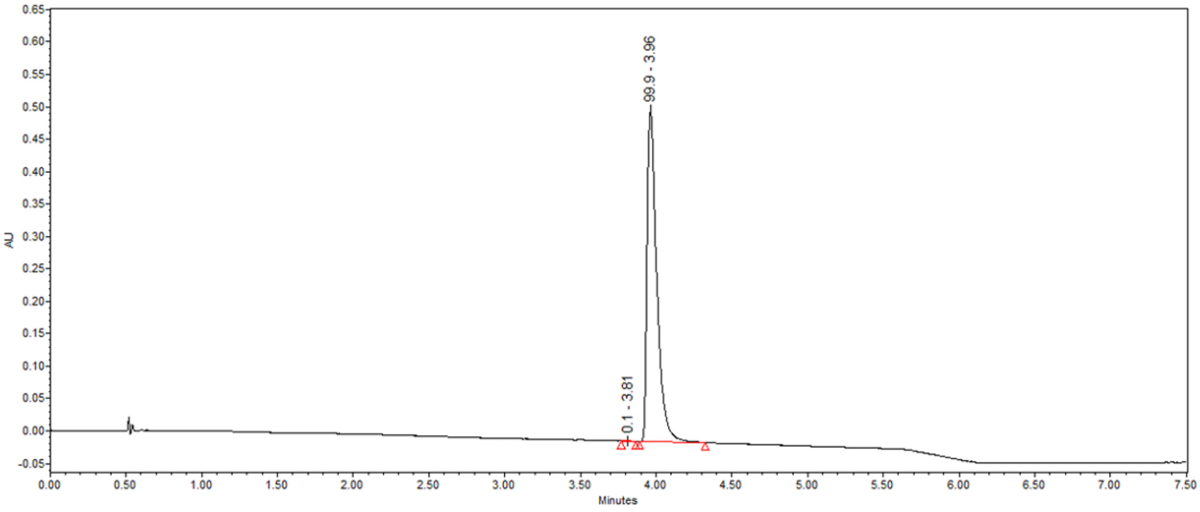

#	Time [min]	Area [µV.sec]	Height [µV]	Area [%]
**1**	3.813	2484	1018	0.11
**2**	3.963	2278815	511740	99.89

Methyl (2R,3S,6aR,11R,11aR,11bR)-3,11a-dimethyl-6,12-dioxodecahydro-2H-2,11-methanocyclohepta[a]indolizine-6a(6H)-carboxylate (9)

HPLC (Chiralpak AD-H, hexane/isopropanol 85:15, 1.0 mL min^{-1}, λ =210 nm).

Racemic

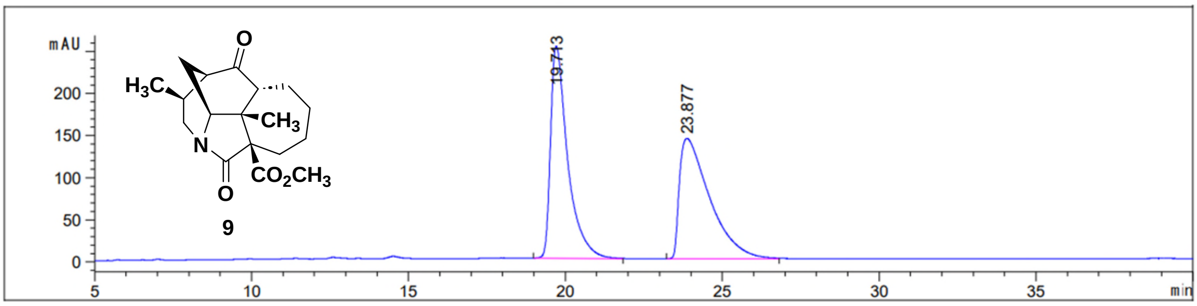

#	Time [min]	Width [min]	Area [mAU*s]	Height [mAU]	Area [%]
**1**	19.713	0.5617	9540.26563	251.97954	49.9658
**2**	23.877	1.0088	9553.32227	142.75111	50.0342

Enantiomerically enriched

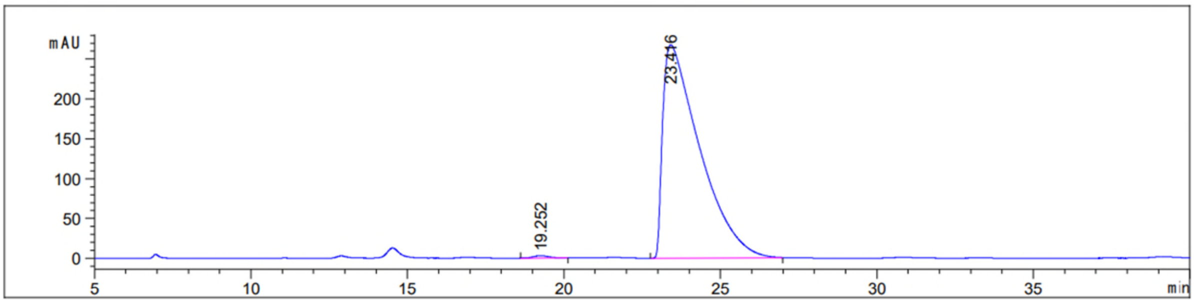

#	Time [min]	Width [min]	Area [mAU*s]	Height [mAU]	Area [%]
**1**	19.252	0.4162	101.83952	3.02966	0.4605
**2**	23.416	1.1798	2.20146e4	267.59479	99.5395

6 Crystallographic Data

Molecular structures (for compounds **5a**, **16**, **17** and **23**) determined through single-crystal X-ray diffraction studies are depicted below. For further details, see the full crystallographic data (in CIF format) which are available as associated content.

Data for compound 5a

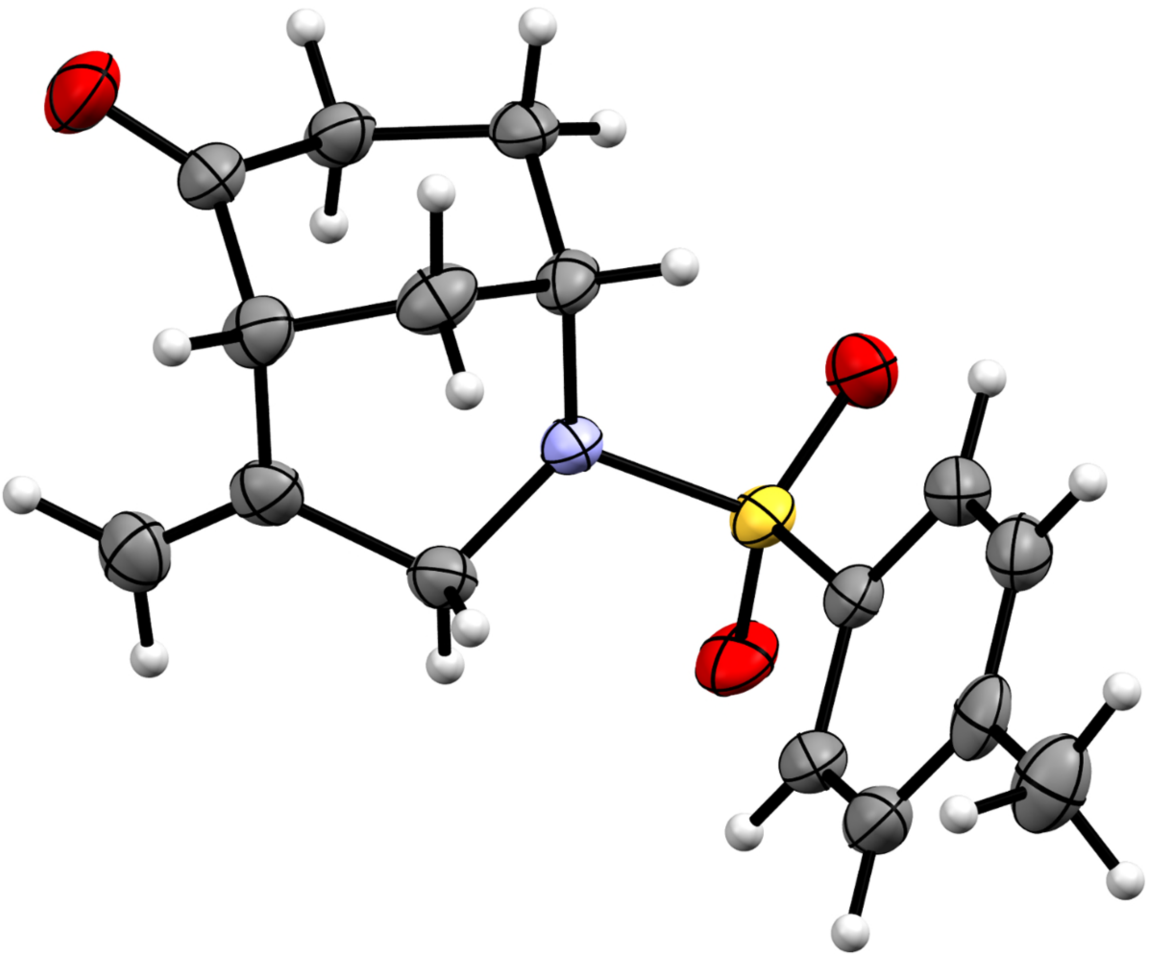

*Molecular structures of **5a** from single-crystal X-ray diffraction studies - displacement ellipsoids are drawn at 50% probability.*

Data for compound 16

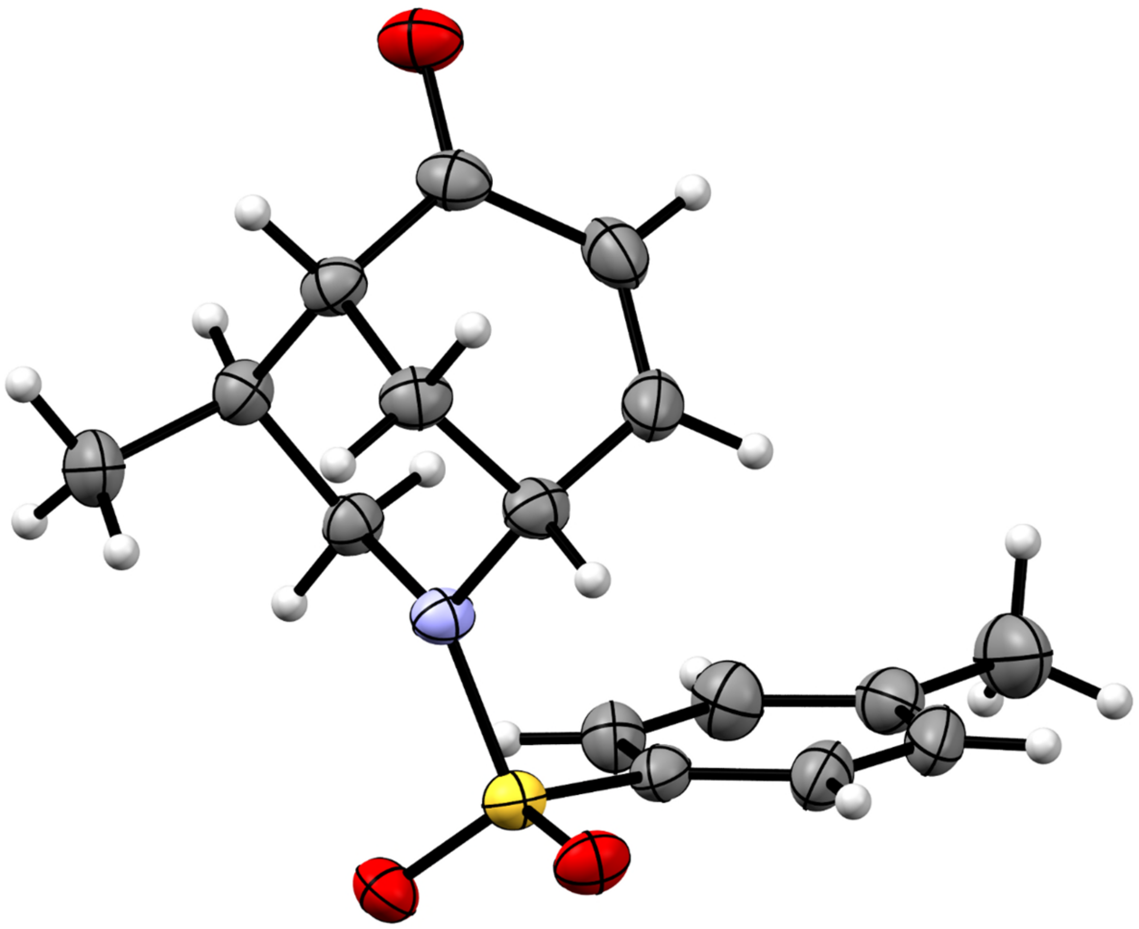

*Molecular structures of **16** from single-crystal X-ray diffraction studies - displacement ellipsoids are drawn at 50% probability.*

Data for compound 17

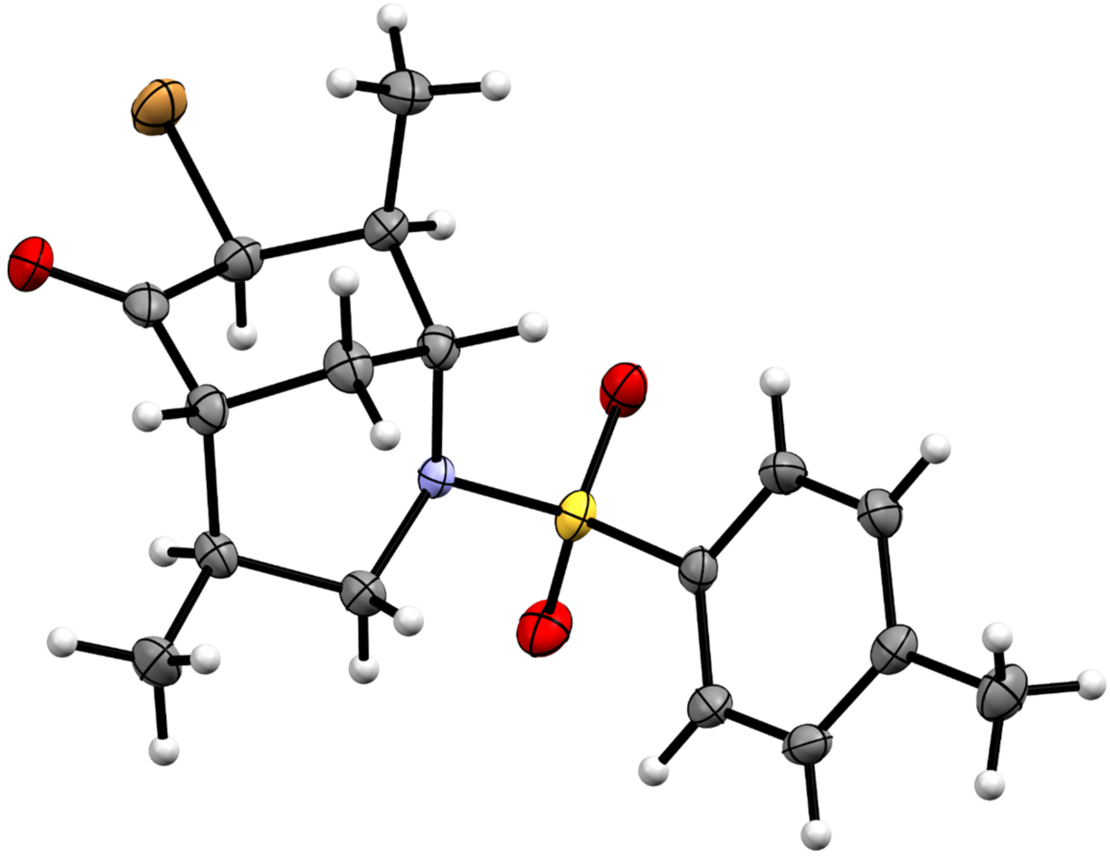

*Molecular structures of **17** from single-crystal X-ray diffraction studies - displacement ellipsoids are drawn at 50% probability.*

Data for compound 23

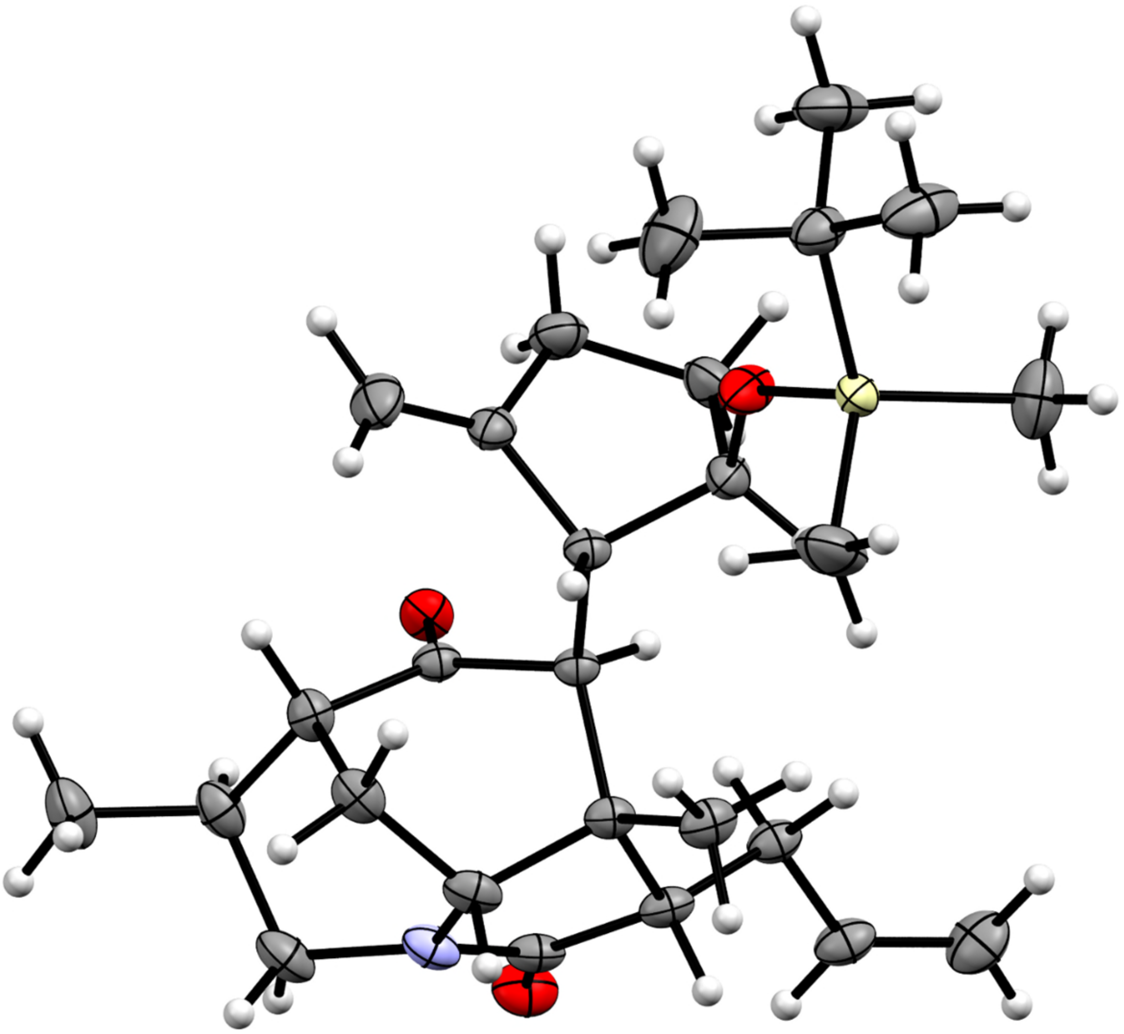

*Molecular structures of **23** from single-crystal X-ray diffraction studies - displacement ellipsoids are drawn at 50% probability.*

7 Computational Details

Table S8. Cartesian coordinates (in Å), energies (in kcal mol^{-1}), and the number of imaginary frequencies of all stationary points, computed at COSMO(MeOH)-ZORA-BLYP-D3(BJ)/TZ2P. Energies (in kcal mol^{-1}) at COSMO(MeOH)-ZORA-M06/TZ2P//COSMO(MeOH)-ZORA-BLYP-D3(BJ)/TZ2P are also provided.

Cyclohexanone substrate

COSMO(MeOH)-ZORA-M06/TZ2P//COSMO(MeOH)-ZORA-BLYP-D3(BJ)/TZ2P

E = -6475.16

G = -6318.52

COSMO(MeOH)-ZORA-BLYP-D3(BJ)/TZ2P

E = -5175.65

G = -5019.01

N_{imag} = 0

C	1.47666040	0.37465119	-0.04882294
C	0.78657378	-1.01262070	-0.10541361
H	0.88669803	1.07172137	0.56289326
H	1.52063469	-1.73782959	-0.47664104
C	-0.44385309	-1.11962846	-1.04146465
C	1.63853378	1.02417739	-1.41180283
C	0.42237575	0.97681364	-2.31793649
C	-0.17502407	-0.44877674	-2.40492022
H	-0.59214240	-2.18086382	-1.25146826
H	-1.09084955	-0.43430576	-3.00174573
H	0.54280771	-1.08770831	-2.93398332
H	-2.45283180	-0.99543849	2.90377917
H	2.45955203	0.29581271	0.42551197
H	0.51827345	-1.33044831	0.90588435
N	-1.74207665	-0.69553456	-0.42695172
C	-1.98365809	0.68042249	0.03026106
H	-2.49442460	0.67753895	0.99832691

H	-1.02760545	1.17793850	0.19812201
C	-2.80980926	1.48758721	-0.94629794
C	-3.43681328	1.08237681	-2.04147206
C	-1.85288550	-1.89242178	2.78815086
S	-2.72806338	-1.90405553	0.17383113
O	-3.94273436	-1.24453485	0.65979807
O	-2.82884271	-2.94943619	-0.84845030
H	-1.10981907	-4.29145231	0.46321088
H	-1.08066766	-1.83690537	4.79537285
H	0.28124910	-3.90201321	4.52513048
H	0.25767683	-5.13833066	2.36504594
O	2.69064780	1.56399034	-1.75514427
C	-1.09497661	-3.76276205	1.41040622
Br	-2.90342223	3.37658270	-0.39005995
C	-1.08225896	-2.37162059	3.84930017
H	-3.38050387	0.03535718	-2.32686837
C	-0.31851416	-3.53574200	3.69592986
H	-0.32141293	1.67715721	-1.91442403
H	0.70117992	1.34028489	-3.31131789
C	-1.84634543	-2.59468516	1.57540410
H	-4.01353126	1.75452991	-2.66678525
C	-0.32938206	-4.23136552	2.48180751

$Pd(PPh_3)_2$

COSMO(MeOH)-ZORA-M06/TZ2P//COSMO(MeOH)-ZORA-BLYP-D3(BJ)/TZ2P

E = -12037.45

G = -11746.64

COSMO(MeOH)-ZORA-BLYP-D3(BJ)/TZ2P

E = -9897.53

G = -9606.72

N_{imag} = 0

Pd	-0.00123873	0.11419815	-0.03829563
P	2.30589796	0.06101763	-0.01832795

P	-2.30801368	0.09329306	0.03314191
C	-3.16284259	-0.21670624	-1.57375437
C	-4.36087606	0.41182925	-1.94754582
C	-2.57298978	-1.14657876	-2.44864678
C	-4.96344942	0.10904663	-3.17438525
H	-4.82514266	1.13590284	-1.28413071
C	-3.18143343	-1.45729495	-3.66723879
H	-1.63172089	-1.61687176	-2.16922522
C	-4.37876409	-0.82792316	-4.03359726
H	-5.89042424	0.60363160	-3.45609499
H	-2.71855035	-2.18080507	-4.33469553
H	-4.84845934	-1.06230338	-4.98613014
C	-3.11312739	1.64064210	0.63696436
C	-4.25552187	1.65058801	1.45250460
C	-2.54182954	2.86300631	0.24011218
C	-4.82147781	2.86465474	1.85914018
H	-4.70431851	0.71438774	1.77164549
C	-3.11486093	4.07418964	0.63589919
H	-1.64194539	2.85588531	-0.37252982
C	-4.25585273	4.07724545	1.44943358
H	-5.70455993	2.86161086	2.49429743
H	-2.66667216	5.01372156	0.32045096
H	-4.69676629	5.01949236	1.76650919
C	-3.02379941	-1.20672499	1.13197275
C	-4.21303191	-1.89389759	0.84182266
C	-2.33092620	-1.50440874	2.31905729
C	-4.70331366	-2.86037600	1.72763342
H	-4.75581086	-1.67920864	-0.07421055
C	-2.82733080	-2.46078578	3.20849438
H	-1.39572332	-0.98889075	2.53069551
C	-4.01508613	-3.14303228	2.91300026
H	-5.62337166	-3.39045205	1.49156070
H	-2.28421359	-2.68193715	4.12457153

H	-4.39723333	-3.89498476	3.59950262
C	3.08761236	-1.20475063	-1.11125867
C	2.43902950	-2.44750841	-1.22594498
C	4.28751277	-0.99064428	-1.80714578
C	2.99150813	-3.46520549	-2.00773443
H	1.49625470	-2.60494579	-0.70479152
C	4.83384636	-2.00680133	-2.60010259
H	4.79641267	-0.03393679	-1.73241332
C	4.19082052	-3.24578981	-2.69855388
H	2.48364872	-4.42390850	-2.08629316
H	5.76271247	-1.83020181	-3.13779545
H	4.61712091	-4.03421776	-3.31462175
C	3.16231202	1.61894868	-0.51498324
C	4.32863632	2.09299228	0.10540422
C	2.60545504	2.34979020	-1.57989592
C	4.93159494	3.27657688	-0.33648539
H	4.76728537	1.54217119	0.93239262
C	3.21528906	3.52380570	-2.02879999
H	1.68792831	1.99537177	-2.04644945
C	4.38002222	3.99138603	-1.40553963
H	5.83319096	3.63750742	0.15357342
H	2.77805640	4.07883135	-2.85580889
H	4.85006300	4.91050223	-1.74779156
C	3.04400108	-0.31145301	1.63277695
C	4.20612959	-1.07956567	1.80465846
C	2.39933857	0.22103224	2.76370485
C	4.71876390	-1.30583286	3.08729560
H	4.71129232	-1.50261470	0.94107989
C	2.91865131	0.00488465	4.04257200
H	1.48403432	0.79535568	2.63164248
C	4.07982837	-0.76164179	4.20726555
H	5.61779563	-1.90572576	3.21020296
H	2.41345846	0.42402831	4.90993733

H	4.47976645	-0.93864441	5.20309088

A

COSMO(MeOH)-ZORA-M06/TZ2P//COSMO(MeOH)-ZORA-BLYP-D3(BJ)/TZ2P

E = -18526.95

G = -18060.96

COSMO(MeOH)-ZORA-BLYP-D3(BJ)/TZ2P

E = -15097.46

G = -14631.47

N_{imag} = 0

C	0.71919726	-1.64663127	-1.17189288
C	0.13601042	-1.22734348	-2.54100341
H	-0.07021292	-2.12339718	-0.57502635
H	0.93874366	-0.81759069	-3.16642018
C	-0.93995559	-0.12726227	-2.43727609
C	1.21066710	-0.45738185	-0.37443469
C	0.25285670	0.71722460	-0.30671634
C	-0.33302679	1.07726531	-1.69341608
H	-1.18999722	0.19730622	-3.45299833
H	-1.05030338	1.89536768	-1.59633184
H	0.47459673	1.45284429	-2.33310897
C	-5.57681014	1.11391968	1.55388412
H	1.53237979	-2.36864293	-1.29136209
H	-0.23859189	-2.10633453	-3.06682733
N	-2.20068792	-0.60843983	-1.76278180
C	-3.06257316	0.36522502	-1.05474388
H	-3.82993373	-0.20075051	-0.53078871
H	-2.45692451	0.84606251	-0.28564611
C	-3.70026615	1.43639902	-1.94493359
C	-3.66581945	1.46472824	-3.33966395
C	-2.32895787	-3.43937715	-0.23679704
S	-2.98357766	-1.93957859	-2.45210005
O	-4.37885462	-1.90521636	-2.00865882

O	-2.67662148	-2.02017452	-3.88106670
H	-1.55239348	-4.26025360	-3.46280451
H	-1.83733676	-4.61658835	1.49675514
H	-0.70118389	-6.40146871	0.18476464
H	-0.56038209	-6.23036743	-2.29325075
O	2.30533466	-0.44580821	0.19116749
H	-6.23547797	-1.88270005	0.06953344
Pd	-5.74220352	0.87330353	-2.68993593
H	-4.71252871	-2.66002230	1.84883873
H	-3.66773025	-1.01998497	3.41130429
H	-4.22458702	1.40127945	3.20583874
C	-7.77434758	2.42303493	-0.26474839
C	-8.66325782	2.56154583	0.81505241
C	-9.13733379	3.82386181	1.17956332
C	-8.73263890	4.96034023	0.46460936
C	-7.85212991	4.82845380	-0.61430291
C	-7.37550232	3.56329142	-0.97688447
H	-9.82382827	3.92337799	2.01762283
C	-1.77574710	-4.54229259	0.41418733
H	-3.25112056	0.62866451	-3.89567900
C	-1.13645001	-5.54660519	-0.32637182
H	-9.10574341	5.94208066	0.74760796
H	-7.53644846	5.70675260	-1.17302714
C	-2.23483546	-3.35493543	-1.63125589
H	-3.74490604	2.40398494	-3.87716037
C	-1.05511135	-5.45113597	-1.71934277
H	-2.82029288	-2.65184654	0.32603427
C	-1.60890800	-4.35006942	-2.38282193
H	-5.80498385	2.17163058	1.46848985
H	-8.98566917	1.68264054	1.36776526
Br	-3.63307206	3.24641015	-0.99921094
P	-7.12809657	0.77512385	-0.79216245
H	-8.40860458	-1.19022480	1.16552121

H	-6.68575825	3.45098434	-1.80993543
C	-8.68305360	-0.22313604	-0.74951140
C	-9.54597444	-0.08517719	-1.85051266
C	-10.75079371	-0.78493104	-1.90420834
C	-11.10719255	-1.64766617	-0.86025602
C	-10.25656328	-1.79030539	0.24017306
C	-9.05327002	-1.07686502	0.29980355
H	-9.26335908	0.56216896	-2.67552918
H	-11.39901849	-0.67712200	-2.76994390
H	-12.03730301	-2.20910139	-0.90902315
H	-10.52756752	-2.45581931	1.05721653
C	-6.16475229	0.19781590	0.66486940
C	-5.82644666	-1.16485242	0.77375304
C	-4.94721486	-1.60133430	1.76733713
C	-4.36307644	-0.68121337	2.64704622
C	-4.67840294	0.67752008	2.53232538
H	0.75989076	1.57625127	0.14237685
H	-0.55673287	0.41540326	0.37444549
C	-5.44851405	-2.01017942	-6.16334786
H	-4.75704234	1.32527057	-6.17423875
H	-3.01205287	0.56280232	-7.75697397
H	-2.79514161	-1.86055003	-8.30487343
H	-4.35894419	-3.50277959	-7.27408755
C	-7.86043946	-1.47084665	-4.28778398
C	-9.02518582	-1.74099999	-5.02064957
C	-9.82900649	-2.83620218	-4.68724349
C	-9.47254449	-3.67343558	-3.62468093
H	-10.73875622	-3.02889796	-5.25173788
H	-6.12530550	-2.73414101	-5.71971317
H	-9.31831627	-1.09096051	-5.83972072
P	-6.77194723	-0.01505820	-4.59885833
H	-7.58188937	0.16367313	-7.49924442
C	-7.82884509	1.13172755	-5.58204799

C	-8.43069031	2.20546978	-4.90358337
C	-9.26099093	3.10073929	-5.58465833
C	-9.48552588	2.93988161	-6.95741679
C	-8.87782360	1.88105287	-7.64404611
C	-8.05432827	0.98065392	-6.96053139
H	-9.72184468	3.92751571	-5.04884191
H	-10.12421344	3.63992847	-7.49112936
H	-9.04543342	1.75653811	-8.71167141
C	-5.57373262	-0.64994479	-5.84771520
C	-4.67983274	0.27019341	-6.42621412
C	-3.69284299	-0.16048650	-7.31292239
C	-3.57156734	-1.52281480	-7.62202738
C	-4.44910971	-2.44341533	-7.04272773
C	-8.30563893	-3.41396984	-2.89653488
C	-7.50725881	-2.31604387	-3.22128399
H	-10.10640575	-4.51595974	-3.35801498
H	-8.02948923	-4.05489790	-2.06226522
H	-6.61215026	-2.09790101	-2.64607624
H	-8.23067244	2.34221950	-3.84291991

TSA-B

COSMO(MeOH)-ZORA-M06/TZ2P//COSMO(MeOH)-ZORA-BLYP-D3(BJ)/TZ2P

E = -18508.39

G = -18041.72

COSMO(MeOH)-ZORA-BLYP-D3(BJ)/TZ2P

E = -15087.54

G = -14620.87

$\boldsymbol{N}_{\mathbf{imag}}$ = 1, 162*i* cm^{-1}

C	-0.54018028	-0.26122878	-1.79366754
C	-1.55905414	-1.37165400	-2.14301144
H	-3.51311225	8.31345919	-7.24578927
H	-1.09236853	-2.34122389	-1.93114004
C	-1.97245717	-1.43706638	-3.62513753

C	0.62516997	-0.20680548	-2.76500711
C	0.25750265	-0.29470072	-4.22911692
C	-0.72636490	-1.44468964	-4.52966877
H	-2.46102130	-2.40819983	-3.74280607
H	-1.02965630	-1.40773765	-5.57783682
H	-0.22188771	-2.40739845	-4.37185836
Br	-1.12541211	3.02187351	-3.64143364
H	-5.35632290	6.68055087	-6.85256763
H	-2.43103285	-1.31286421	-1.48587069
N	-2.99355695	-0.39032010	-4.06615705
C	-3.52101599	0.61400667	-3.09500744
H	-4.56181340	0.38421006	-2.83036293
H	-2.97253698	0.49930126	-2.16739335
C	-3.52306038	2.05812538	-3.48859919
C	-4.13011698	2.72929608	-4.50108357
C	-6.09011307	-2.01163398	-3.43782350
S	-4.13735965	-1.03538137	-5.13132636
O	-5.11023234	0.02021604	-5.41889107
O	-3.39524148	-1.65658712	-6.23106631
H	-3.72725289	-3.91285972	-5.02221669
H	-7.58428072	-2.77518874	-2.09079214
H	-6.78997332	-5.12569604	-2.24450438
H	-4.86152935	-5.69662721	-3.71203160
O	1.78676265	-0.06118898	-2.37927451
H	-4.47797006	8.38028339	-0.15288942
Pd	-3.92952224	3.67298477	-2.23126124
H	-2.80243157	9.27421210	1.43420832
H	-0.54225678	8.24198702	1.58236894
H	0.04391036	6.32329663	0.09987005
C	-4.07598660	6.82065867	-3.68862376
C	-3.04424744	7.74334262	-3.91347760
C	-2.84183166	8.27597002	-5.19313802
C	-3.67128278	7.89835344	-6.25316435

C	-4.70742966	6.97933749	-6.03245877
C	-4.90296132	6.43857777	-4.76132791
H	-2.39899750	8.04883011	-3.09552792
C	-6.73204688	-3.02144019	-2.71804662
H	-4.94722459	2.26429996	-5.04887295
C	-6.28618314	-4.34480193	-2.80823353
H	-2.03678338	8.98890000	-5.35687979
C	-1.88092508	6.28428535	-0.86339653
C	-4.99941066	-2.34474875	-4.25091985
H	-3.79258625	3.70388835	-4.83865632
C	-5.20111702	-4.66749739	-3.63132212
H	-6.44364921	-0.98769348	-3.38633253
C	-4.55244083	-3.66861487	-4.36169588
H	-1.63512501	5.42970094	-1.48912694
H	-1.00604784	0.73243222	-1.85138559
H	-0.16076268	-0.38556099	-0.77523363
P	-4.37406276	6.02524754	-2.05121305
H	-5.81017143	8.60775703	-2.55089923
H	-5.69868661	5.71474418	-4.59840471
C	-6.01424526	6.69199710	-1.56381292
C	-6.85159366	5.88100336	-0.78340563
C	-8.09735298	6.35235914	-0.36039859
C	-8.52046261	7.63515691	-0.72552183
C	-7.69425553	8.44564457	-1.51564205
C	-6.44578835	7.97774737	-1.93454745
H	-6.52444603	4.88516065	-0.50408252
H	-8.73362323	5.71840459	0.25192490
H	-9.49148538	8.00201633	-0.40099444
H	-8.02288524	9.44127603	-1.80489588
C	-3.16468008	6.85373517	-0.94020996
C	-3.48571958	7.94172433	-0.11593046
C	-2.54051726	8.44080397	0.78635749
C	-1.26898590	7.86144623	0.86861547

C	-0.93780463	6.78559699	0.03491368
H	1.16851210	-0.38145348	-4.82936187
H	-0.22698897	0.66176774	-4.47577554
H	-6.94488222	6.05775043	3.50508358
H	-4.71176231	6.87659301	2.74896107
H	1.49661055	2.96410695	2.49482962
H	-0.55658369	2.85931220	3.90152572
C	-4.71253940	3.83369911	1.20159046
C	-5.96227639	3.37105826	1.64493896
C	-6.75852747	4.16822159	2.47406089
C	-6.31933590	5.43532647	2.87008238
C	-5.06764998	5.89534209	2.44533519
C	-4.27017456	5.10198372	1.62071823
H	-6.32166614	2.39071183	1.35214210
C	-1.90090520	2.88863387	2.21892267
H	-2.79158057	2.84536132	2.83910275
P	-3.66343906	2.91115680	-0.00742797
H	-2.47405773	0.41883206	1.20398047
C	-4.22469606	1.16617236	0.17842560
C	-5.47131784	0.81438097	-0.37482947
C	-5.93215134	-0.49907836	-0.30474348
C	-5.13919031	-1.49264159	0.28476565
C	-3.89506043	-1.15476635	0.82332116
C	-3.44151175	0.17003487	0.77999683
H	-6.06847773	1.56895192	-0.88227487
H	-6.90135704	-0.75108969	-0.72487129
H	-5.48679865	-2.52187911	0.31272304
H	-3.27004055	-1.92058289	1.27642169
C	-2.01882088	2.94164168	0.81805198
C	-0.86079187	2.99990590	0.03207893
C	0.40182357	3.00240573	0.63291806
C	0.51551666	2.95419383	2.02556911
C	-0.63932642	2.89745712	2.81776630

H	-0.94450692	3.05339925	-1.04915584
H	1.29309487	3.04908405	0.01142522
H	-7.72447135	3.79560958	2.80705971
H	-3.29987288	5.46702586	1.30588485

B

COSMO(MeOH)-ZORA-M06/TZ2P//COSMO(MeOH)-ZORA-BLYP-D3(BJ)/TZ2P

E = -18536.74

G = -18067.71

COSMO(MeOH)-ZORA-BLYP-D3(BJ)/TZ2P

E = -15116.08

G = -14647.05

$\boldsymbol{N}_{\mathbf{imag}}$ = 0

C	3.22458601	2.23642266	-1.14130930
C	3.11988849	0.70393763	-0.97188295
H	2.39448373	2.71501937	-0.60504917
H	3.99300971	0.22790102	-1.43531114
C	1.87996977	0.09244733	-1.65501762
C	3.08940182	2.68868048	-2.57839133
C	1.96622091	2.04358611	-3.35230462
C	1.89825467	0.51732315	-3.13695382
H	1.97281764	-1.00107063	-1.62161450
H	1.04992925	0.11120319	-3.68973726
H	2.79205169	0.04702053	-3.56629815
C	-3.57648130	6.87706324	0.10242278
H	4.15929225	2.61927105	-0.72018279
H	3.16802461	0.45064908	0.08871238
N	0.58014554	0.46554954	-0.95935144
C	-0.67731659	0.14899178	-1.73195290
H	-0.41736872	-0.53245195	-2.54982681
H	-1.34300478	-0.40769193	-1.07719077
C	-1.40723029	1.35514229	-2.26675071
C	-1.53632402	1.56438573	-3.57585596

C	1.01619138	2.54431105	1.36267855
S	0.48504207	-0.07429087	0.65126656
O	-0.93764500	-0.07713242	1.02768304
O	1.23264769	-1.32922783	0.85043210
H	2.26653818	-0.23735311	2.90809349
H	1.36596222	4.57066243	1.98575831
H	2.77070425	3.92013601	3.93679389
H	3.21506759	1.51627188	4.40146422
O	3.81754090	3.55920666	-3.06478577
H	-0.60273657	5.77482441	1.33992355
Pd	-2.27639918	2.60977247	-0.84833106
H	0.01508356	8.12135307	0.89443252
H	-1.65783957	9.69336406	-0.07943582
H	-3.96080125	8.88192499	-0.58616350
C	-4.84019398	4.26756617	1.36011531
C	-5.30957401	4.18976397	2.68087772
C	-6.68174771	4.18590009	2.94286229
C	-7.60199658	4.26816417	1.89194375
C	-7.14176636	4.35611450	0.57428148
C	-5.76999245	4.34910787	0.31074212
H	-4.61040474	4.10931086	3.50602955
C	1.56336768	3.52269370	2.19361126
H	-1.07222327	0.89021143	-4.30079386
C	2.35196189	3.15571323	3.28733008
H	-7.03088625	4.11005175	3.97006039
H	-8.66970416	4.25775063	2.09795306
C	1.28465630	1.20154734	1.62988453
H	-2.09987244	2.39216369	-3.99563091
C	2.60268976	1.80318832	3.55064819
H	0.40081874	2.82725871	0.51608455
C	2.07123177	0.81319884	2.71964375
H	-4.58830618	6.54801400	-0.10937114
H	-7.84564094	4.41336466	-0.25134373

Br	-0.38693358	4.30259388	-1.48006883
P	-3.04393396	4.20626722	0.92836625
H	-2.50028024	5.80960661	3.40485724
H	-5.42637826	4.39277899	-0.71616758
C	-2.22211326	3.84427297	2.53762976
C	-1.72244739	2.55349093	2.76226505
C	-1.15629367	2.21341295	3.99351927
C	-1.06136320	3.16906878	5.00667727
C	-1.54124579	4.46733584	4.78645409
C	-2.12417178	4.80334529	3.56275880
H	-1.75038798	1.81273030	1.97093616
H	-0.76422017	1.21128576	4.14387090
H	-0.60655551	2.91183213	5.96027274
H	-1.46538771	5.21723636	5.57039526
C	-2.64056876	5.98769880	0.65255140
C	-1.34046483	6.45000921	0.92271509
C	-0.99212739	7.77796241	0.66970464
C	-1.93065739	8.65995153	0.12137459
C	-3.22206072	8.20440684	-0.16405300
H	2.06193602	2.28927034	-4.41486112
H	1.03723174	2.50051649	-2.97999443
H	-7.14082183	1.13178005	-1.26521291
P	-4.13166874	1.18869376	-0.98520968
H	-4.52649962	0.33751020	-3.81260837
H	-3.84917871	1.75186200	1.89071657
C	-3.93485517	-0.41251426	-1.87445998
C	-3.47252212	-1.53312068	-1.16283397
C	-3.25042765	-2.74440793	-1.82210114
C	-3.48844031	-2.84792507	-3.19762380
C	-3.95546154	-1.73601198	-3.90830532
C	-4.17400729	-0.52197295	-3.25162958
H	-3.28377628	-1.45986462	-0.09548382
H	-2.88981414	-3.60391635	-1.26212975

H	-3.31529643	-3.79048768	-3.71140238
H	-4.14721327	-1.81169594	-4.97606674
C	-5.03040080	0.60350964	0.51437525
C	-6.06199396	-0.35147986	0.40289809
C	-6.73220576	-0.79989930	1.54169522
C	-6.37039334	-0.32127422	2.80840171
C	-5.32909705	0.60163054	2.92751596
C	-4.66560820	1.05593667	1.78474227
H	-6.32494159	-0.76450736	-0.56545811
H	-7.52956657	-1.53237434	1.44161702
H	-6.89102008	-0.67745149	3.69409735
H	-5.02775105	0.97009035	3.90475505
C	-5.35799073	2.10009430	-2.02412400
C	-4.87621412	3.07970242	-2.90956126
C	-5.75671169	3.80171748	-3.71874291
C	-7.13259151	3.55778458	-3.64686575
C	-7.61893533	2.58623649	-2.76546202
C	-6.73858593	1.86009682	-1.95899351
H	-3.81242020	3.29530640	-2.94094074
H	-5.36854821	4.56074767	-4.39332600
H	-7.82130318	4.12602409	-4.26719248
H	-8.68768104	2.39893391	-2.69482322

Proline

COSMO(MeOH)-ZORA-M06/TZ2P//COSMO(MeOH)-ZORA-BLYP-D3(BJ)/TZ2P

E = -2802.96

G = -2735.76

COSMO(MeOH)-ZORA-BLYP-D3(BJ)/TZ2P

E = -2227.95

G = -2160.75

N_{imag} = 0

C	4.04855338	0.13226173	0.00228855
H	3.74488897	-0.29272610	0.96366001

H	4.27745325	-0.69001607	-0.68868900
C	5.09847479	1.96705297	-1.14855375
C	5.24292136	1.09243212	0.11366816
H	5.62121577	2.92455662	-1.08428600
H	5.47247773	1.43040871	-2.02709221
H	5.15273732	1.70648389	1.01838049
H	6.19814832	0.56202735	0.15537487
C	3.06701231	3.44734686	-0.60938787
O	2.26050416	3.21285459	0.43794559
H	3.24627401	2.18439192	-2.32673190
O	3.37290736	4.57500749	-0.98095974
N	2.95253394	0.99486461	-0.54589091
C	3.56289067	2.14697062	-1.28011356
H	2.23555647	2.17643245	0.42856452
H	2.31519726	0.46827734	-1.13983767

PPh_3

COSMO(MeOH)-ZORA-M06/TZ2P//COSMO(MeOH)-ZORA-BLYP-D3(BJ)/TZ2P

E = -5979.33

G = -5842.60

COSMO(MeOH)-ZORA-BLYP-D3(BJ)/TZ2P

E = -4904.05

G = -4767.32

$\boldsymbol{N}_{\mathbf{imag}}$ = 0

H	2.59925892	0.61851463	4.88522017
P	2.24920570	0.04639969	-0.01608954
H	4.52444575	-0.93806576	5.18105312
C	3.07785620	-1.20102770	-1.11191132
C	2.46350955	-2.46178161	-1.22499530
C	4.26783689	-0.95924488	-1.81794502
C	3.03647256	-3.46568884	-2.01173081
H	1.53439290	-2.65843474	-0.69271143
C	4.83562999	-1.96073170	-2.61424679

H	4.75515281	0.00861716	-1.74427271
C	4.22445241	-3.21614083	-2.71043538
H	2.55314818	-4.43740767	-2.08537859
H	5.75792371	-1.76047881	-3.15542338
H	4.66769279	-3.99352484	-3.32858289
C	3.13014871	1.60585715	-0.50156419
C	4.25641562	2.11535274	0.16501574
C	2.62024708	2.30765289	-1.60961513
C	4.86388770	3.29885857	-0.27147022
H	4.66327189	1.58822142	1.02305612
C	3.23440162	3.48242303	-2.05314323
H	1.74057311	1.93048093	-2.12862903
C	4.35773563	3.98287938	-1.38233184
H	5.73572848	3.68248774	0.25426441
H	2.83259100	4.01079058	-2.91495093
H	4.83185870	4.90118247	-1.72124091
C	3.03174093	-0.32330322	1.62571156
C	4.11566336	-1.19873150	1.80219511
C	2.48663634	0.31963866	2.75290101
C	4.64740826	-1.42102992	3.07815037
H	4.54930806	-1.70521698	0.94500527
C	3.02666840	0.10853928	4.02464618
H	1.63877968	0.99222181	2.63293356
C	4.10828011	-0.76620802	4.19099978
H	5.48694767	-2.10228767	3.20037590

K_2HPO_4

COSMO(MeOH)-ZORA-M06/TZ2P//COSMO(MeOH)-ZORA-BLYP-D3(BJ)/TZ2P

E = -1368.55

G = -1374.87

COSMO(MeOH)-ZORA-BLYP-D3(BJ)/TZ2P

E = -1009.46

G = -1015.78

***N*$_{imag}$** = 0

P	-0.78832194	1.37332753	1.84659948
O	-0.81482457	2.18250343	0.52376084
O	-1.85627504	1.85236013	2.85450321
O	-1.25025996	-0.17000687	1.33459566
O	0.63319214	1.24101005	2.43713979
K	1.82662339	2.59094488	0.43636155
K	-2.82841343	3.77893058	1.25537963
H	-1.21473938	-0.79203780	2.08487390

KH_2PO_4

COSMO(MeOH)-ZORA-M06/TZ2P//COSMO(MeOH)-ZORA-BLYP-D3(BJ)/TZ2P

E = -1392.87

G = -1391.79

COSMO(MeOH)-ZORA-BLYP-D3(BJ)/TZ2P

E = -1031.00

G = -1029.92

***N*$_{imag}$** = 0

P	-1.17018412	1.53914952	1.53940621
O	-0.29917623	1.79724296	0.16587955
O	-2.59377724	1.94051362	1.22083329
O	-1.17454468	-0.09902494	1.70485156
O	-0.48050225	2.18510716	2.72839478
H	0.65888338	1.83492009	0.34983295
K	-2.72111122	3.73132324	3.32021927
H	-0.38608559	-0.41231507	2.18776992

KBr

COSMO(MeOH)-ZORA-M06/TZ2P//COSMO(MeOH)-ZORA-BLYP-D3(BJ)/TZ2P

E = -160.37

G = -175.95

COSMO(MeOH)-ZORA-BLYP-D3(BJ)/TZ2P

E = -118.39

G = -133.97

***N*imag = 0**

Br	0.00000000	0.00000000	1.19226772
K	0.00000000	0.00000000	4.35236362

C

COSMO(MeOH)-ZORA-M06/TZ2P//COSMO(MeOH)-ZORA-BLYP-D3(BJ)/TZ2P

E = -15162.76

G = -14773.49

COSMO(MeOH)-ZORA-BLYP-D3(BJ)/TZ2P

E = -12278.21

G = -11888.94

***N*imag** = 0

C	-4.21491261	-0.07294687	1.95749754
C	-5.25380268	0.68479485	1.09447736
H	-3.49167425	0.63504521	2.38007354
H	-6.07672514	-0.00568788	0.87247074
C	-4.77157205	1.17509039	-0.29124733
C	-3.39740094	-1.07979587	1.17858822
C	-2.90714050	-0.59375303	-0.16723672
C	-4.06020114	0.00535791	-1.00426557
H	-5.67156246	1.43645958	-0.86439404
H	-3.67764765	0.29956884	-1.98511046
H	-4.81715559	-0.76638722	-1.19055875
C	-3.10117627	4.96173049	-7.18315241
H	-4.70827028	-0.57400304	2.79586009
H	-5.69208659	1.49614368	1.67619522
N	-3.86561351	2.39556758	-0.30831443
C	-3.63842565	2.93121090	-1.70109518
H	-4.01644065	2.16549572	-2.37677850
H	-4.27024093	3.80502214	-1.85680426
C	-2.18652588	3.23360961	-2.05516137
C	-1.21981819	2.32098343	-1.92448213

C	-2.00299362	2.69668946	2.12203486
S	-4.19799130	3.65605937	0.76974198
O	-3.52791665	4.84988194	0.23604715
O	-5.63040389	3.78098918	1.08456455
H	-4.96370568	3.63178982	3.56131379
H	-0.27290992	1.99413783	3.19120035
H	-1.34661053	2.20462071	5.42707072
H	-3.68854354	3.02003970	5.61649883
O	-3.13435501	-2.20143519	1.61615024
H	-1.34848841	7.07227750	-5.15245615
Pd	-1.74480652	5.11722794	-2.66775495
H	-0.51242569	7.85417781	-7.35665522
H	-1.32717160	6.78539240	-9.45404471
H	-2.98814256	4.93205756	-9.33414658
C	-4.13602347	3.46092056	-4.69999284
C	-5.52298265	3.30045582	-4.57407229
C	-6.09790550	2.03334875	-4.72183398
C	-5.29279806	0.92510401	-5.00300973
C	-3.90787556	1.08602039	-5.15001304
C	-3.33056605	2.34627688	-4.99549356
H	-6.15243247	4.15341682	-4.34160249
C	-1.29290685	2.35964657	3.27445392
H	-1.41958014	1.32741768	-1.52211130
C	-1.90013585	2.47729713	4.53235434
H	-7.17294822	1.91408905	-4.61093347
H	-5.73940040	-0.06058564	-5.10754243
C	-3.31978455	3.15571028	2.24846634
H	-0.18734986	2.51167250	-2.20743332
C	-3.21761891	2.93516603	4.64095413
H	-1.56236268	2.58353532	1.13748815
C	-3.93876200	3.28232480	3.49350943
H	-3.82591097	4.15504427	-7.14315731
H	-3.27884799	0.22710619	-5.37048597

O	0.24687527	8.85096207	-2.48083543
P	-3.29784339	5.05504189	-4.35607542
H	-5.19849816	6.57775606	-6.07158533
H	-2.25330216	2.46240133	-5.07488262
C	-4.65326730	6.24026522	-4.00404459
C	-4.94299131	6.55221448	-2.66487314
C	-6.00418508	7.40934068	-2.35950418
C	-6.77383209	7.96646135	-3.38738332
C	-6.47974753	7.66688362	-4.72338079
C	-5.42305873	6.80621090	-5.03350928
H	-4.33532035	6.13220199	-1.86670668
H	-6.22444433	7.64658889	-1.32143876
H	-7.59583456	8.63737813	-3.14920824
H	-7.07236411	8.10342885	-5.52376961
C	-2.64241095	5.56199535	-5.99817566
C	-1.70435483	6.60749647	-6.06772683
C	-1.23759722	7.04495597	-7.31001223
C	-1.69548846	6.44434051	-8.48933958
C	-2.62690640	5.40269780	-8.42275723
H	-2.41051989	-1.41033400	-0.69882343
H	-2.16439202	0.19250253	0.02982170
O	-1.22446976	7.23437941	-3.00634567
C	-0.20971737	7.68944951	-2.35156114
H	1.54465458	6.74428521	-1.57423850
C	0.47890979	6.77580170	-1.32207396
N	-0.04320625	5.35042115	-1.29293962
C	-0.21006434	4.94721820	0.15885478
C	0.53861976	6.01501402	0.96699154
C	0.31069009	7.28398505	0.12846213
H	0.66467863	4.74942428	-1.71443609
H	-0.71032447	7.65889994	0.27590780
H	0.17232718	3.93470770	0.29329280
H	-1.27382366	4.95599532	0.39184157

H	1.60978142	5.78407792	1.02325035
H	0.14969246	6.10116979	1.98656720
H	1.00962947	8.09353463	0.34953402

D1

COSMO(MeOH)-ZORA-M06/TZ2P//COSMO(MeOH)-ZORA-BLYP-D3(BJ)/TZ2P

E = -15151.80

G = -14758.60

COSMO(MeOH)-ZORA-BLYP-D3(BJ)/TZ2P

E = -12263.07

G = -11869.87

N_{imag} = 0

C	1.60009369	-0.72788715	-0.62848995
C	0.72963161	-1.66895932	-1.47589482
H	1.21400994	-0.65501019	0.38792010
H	1.29184507	-1.96970150	-2.36851860
C	-0.58521403	-1.05643119	-1.98525451
C	1.77230810	0.71078688	-1.17533468
C	0.53136057	1.29471753	-1.87945852
C	-0.29285696	0.28272148	-2.69153047
H	-0.98153277	-1.72105714	-2.75349639
H	-1.23534061	0.75022452	-2.99546741
H	0.25190269	0.02609939	-3.60957081
H	3.52120930	0.11059118	-1.91123116
H	2.59148694	-1.18569545	-0.53787138
H	0.53473159	-2.58589540	-0.90870766
N	-1.68705492	-0.97323510	-0.95918115
C	-1.39053769	-0.61457484	0.45805737
H	-2.21744439	-0.98259449	1.06747995
H	-0.49915334	-1.13706622	0.80091094
C	-1.31303596	0.88836895	0.66995206
C	-2.29956429	1.68615746	0.25041761
C	-2.45063644	-3.93029215	0.78498901

S	-2.96338739	-2.03476702	-1.15607690
O	-4.03290146	-1.59743048	-0.25352860
O	-3.23789729	-2.13508607	-2.59427601
H	-1.67378710	-4.23968447	-2.53440265
H	-1.93289615	-5.35610934	2.31173828
H	-0.76717413	-6.89337220	0.73860297
H	-0.64758523	-6.34049132	-1.68492339
N	2.10463412	1.59597006	0.02493229
C	2.45987202	3.02837284	-0.28735507
Pd	0.31516049	1.78105646	1.51789853
C	3.33056868	1.13598575	0.79049818
H	3.20603266	1.48500275	1.81574171
H	3.39540036	0.05158351	0.81197876
C	3.96665145	3.02468666	-0.66028928
C	4.55515026	1.82650907	0.13614261
H	4.42697439	3.97840161	-0.39442209
H	4.08160770	2.86926235	-1.73404291
H	5.25757774	2.15462703	0.90810097
H	5.09316185	1.14139829	-0.52613242
C	2.22524043	3.94827987	0.95552480
C	-1.87110989	-5.11138959	1.25451163
H	-3.13547828	1.27863041	-0.32189113
C	-1.21731339	-5.97590319	0.36824350
O	1.74660492	3.42682332	2.03527293
O	2.54689635	5.15398222	0.83445909
C	-2.36671063	-3.62772708	-0.58023416
H	-2.31082379	2.75722968	0.42997591
C	-1.14715333	-5.66516197	-0.99518430
H	-2.96315042	-3.25824395	1.46437416
C	-1.72048965	-4.48605678	-1.47869483
H	-3.45400723	2.38344128	4.93804901
H	1.74261152	0.84817768	3.81306975
H	1.82892383	3.40323386	-1.09441951

P	-0.95746830	1.82686760	3.38000282
H	-0.30351002	-0.70607803	4.69751076
C	-1.94233092	0.31616763	3.71827607
C	-3.25711638	0.18875608	3.23714489
C	-3.93625393	-1.02436373	3.36537577
C	-3.31000430	-2.12380223	3.96504809
C	-2.00120934	-2.00233936	4.44370335
C	-1.31972234	-0.78774481	4.32404420
H	-3.73928407	1.02364091	2.74111252
H	-4.94995131	-1.11388724	2.98400392
H	-3.83944938	-3.06895993	4.05695703
H	-1.50774807	-2.85125165	4.91012181
C	-2.09294384	3.25053784	3.49254036
C	-1.78733522	4.39229133	2.73202955
C	-2.59502909	5.52837469	2.81828168
C	-3.71191176	5.53140438	3.66255738
C	-4.01686059	4.39778307	4.42499187
C	-3.21079021	3.25892798	4.34387519
H	-0.91809188	4.38396461	2.07889816
H	-2.35550874	6.40711191	2.22474656
H	-4.34387159	6.41396320	3.72497767
H	-4.88188070	4.39842701	5.08354814
C	0.18755557	1.97107417	4.80899448
C	-0.17915229	2.64896917	5.98248481
C	0.70678874	2.70468032	7.06267229
C	1.95894350	2.08429991	6.98062820
C	2.32925901	1.41062812	5.81098521
C	1.44979259	1.35866744	4.72718743
H	-1.14560202	3.13771228	6.05500315
H	0.41852300	3.23520144	7.96694687
H	2.64574046	2.13162030	7.82222971
H	3.30300692	0.93249522	5.73908132
O	2.80788061	0.71291254	-2.19024739

H	0.87267555	2.08865536	-2.55204248
H	-0.11045837	1.75792542	-1.13378650

D2

COSMO(MeOH)-ZORA-M06/TZ2P//COSMO(MeOH)-ZORA-BLYP-D3(BJ)/TZ2P

E = -15158.42

G = -14765.05

COSMO(MeOH)-ZORA-BLYP-D3(BJ)/TZ2P

E = -12269.54

G = -11876.17

$\boldsymbol{N_{imag}}$ **=** 0

C	1.06092326	-1.31354520	0.18696102
C	0.25435118	-2.01866890	1.29828695
H	0.44999611	-0.53178357	-0.26980176
H	0.66782458	-3.02036849	1.46468512
C	0.28257856	-1.33251668	2.67923420
C	2.40149885	-0.66033624	0.61483371
C	2.45215439	-0.14291073	2.06504336
C	1.73539686	-1.04099565	3.07435080
H	-0.10936546	-2.05296282	3.39863491
H	1.76081639	-0.57069086	4.06286887
H	2.25043642	-2.00728844	3.15670613
H	-3.36745795	-1.10132705	-2.08774523
H	1.26050720	-2.03242386	-0.61765084
H	-0.77658888	-2.16610597	0.95666066
N	-0.59580390	-0.10530106	2.82738376
C	-0.90568819	0.75145394	1.64788988
H	-1.86546977	1.23349743	1.84628724
H	-1.06876777	0.12047320	0.77471924
C	0.09870251	1.86540320	1.36650356
C	0.47572873	2.74215074	2.29632803
C	-3.84245432	-1.03824187	2.12330029
S	-1.86490240	-0.29211401	3.90730460

O	-2.54906760	1.00241619	3.99982763
O	-1.31914367	-0.89970673	5.12517646
H	-2.20270772	-3.14786215	4.26196642
H	-5.29016216	-1.65075804	0.65795158
H	-5.11645463	-4.06801165	1.22208028
H	-3.57688545	-4.81690911	3.02937371
N	2.65173918	0.51751012	-0.33272205
C	3.01086048	0.06165411	-1.74872058
Pd	0.73932913	1.84167808	-0.55739927
C	3.78295452	1.41872935	0.12290042
H	4.55016972	0.81792649	0.62543907
H	3.40293224	2.16708353	0.81931965
C	4.36053363	0.75892048	-2.08015625
C	4.35331244	1.99481683	-1.16803764
H	5.19093843	0.09754410	-1.80883985
H	4.44304711	0.99331830	-3.14409667
H	5.34844733	2.42380823	-1.01819484
H	3.69397298	2.77245418	-1.57103101
C	1.98120320	0.42618093	-2.83892208
C	-4.61329310	-1.98062746	1.43999778
H	0.14843180	2.63961461	3.33371989
C	-4.51491683	-3.33998760	1.76022446
O	1.20851834	1.43878449	-2.64865652
O	2.01577891	-0.24416883	-3.90007580
C	-2.97540669	-1.47504328	3.13525449
H	1.12968990	3.58125528	2.07317380
C	-3.64888124	-3.76278318	2.77474771
H	-3.92319868	0.01568624	1.87838381
C	-2.87198315	-2.83114725	3.46917766
H	-2.43029384	3.29349786	1.60979300
H	3.13009382	-1.02116648	-1.77093166
H	1.22601896	4.21113363	-2.43148521
P	-1.07594098	3.13124751	-1.01980211

H	-1.41328894	0.41239520	-1.87114014
C	-1.50298169	4.52846184	0.08848042
C	-1.12695853	5.83811191	-0.25236329
C	-1.38137582	6.89646584	0.62622592
C	-2.01935788	6.65898740	1.84771302
C	-2.40034669	5.35597662	2.19089586
C	-2.13505383	4.29609311	1.32316776
H	-0.63985528	6.03798672	-1.20109164
H	-1.08424780	7.90535523	0.35065681
H	-2.22044626	7.48275755	2.52837703
H	-2.89715943	5.16246027	3.13836099
C	-0.89904358	3.90731722	-2.67161031
C	-2.00778544	4.08736872	-3.51297974
C	-1.85135263	4.73034674	-4.74479719
C	-0.59261963	5.19975023	-5.13823183
C	0.51425357	5.02112377	-4.29972626
C	0.36428062	4.37049612	-3.07312297
H	-2.98707301	3.72558040	-3.21412063
H	-2.71225926	4.86340658	-5.39554027
H	-0.47389341	5.69844050	-6.09722129
H	1.49495957	5.37806887	-4.60548193
C	-2.57325693	2.07805565	-1.12949232
C	-3.85736780	2.53300028	-0.79226896
C	-4.95722918	1.67638590	-0.91146468
C	-4.78441544	0.36920521	-1.37722687
C	-3.50703160	-0.08561769	-1.72685721
C	-2.40669698	0.76288248	-1.60173999
H	-4.00323295	3.54770516	-0.43672583
H	-5.94781498	2.03418386	-0.64228442
H	-5.64166570	-0.29285606	-1.46913814
H	2.01757593	0.85154628	2.10784662
H	3.50553758	-0.05263947	2.34649738
O	3.47628821	-1.62851244	0.54757976

H 3.29537925 -2.25797603 -0.17335055

H_2O

COSMO(MeOH)-ZORA-M06/TZ2P//COSMO(MeOH)-ZORA-BLYP-D3(BJ)/TZ2P

E = -423.00

G = -421.36

COSMO(MeOH)-ZORA-BLYP-D3(BJ)/TZ2P

E = -323.69

G = -322.05

N_{imag} = 0

H 0.76839599 0.00000000 0.29009758

O 0.00000000 0.00000000 0.89004694

H -0.76839599 0.00000000 0.29009758

E1

COSMO(MeOH)-ZORA-M06/TZ2P//COSMO(MeOH)-ZORA-BLYP-D3(BJ)/TZ2P

E = -14733.59

G = -14356.46

COSMO(MeOH)-ZORA-BLYP-D3(BJ)/TZ2P

E = -11948.61

G = -11571.48

N_{imag} = 0

C 1.53342269 -0.86831283 -0.78942849

C 0.47674789 -1.50151034 -1.71308341

H 1.23392111 -0.94177167 0.26235611

H 0.93198929 -1.64303647 -2.70194763

C -0.79899649 -0.66681994 -1.94042097

C 1.80607231 0.58616441 -1.12901258

C 0.76259445 1.40349530 -1.62623355

C -0.44029237 0.78496224 -2.33502956

H -1.31990227 -1.09945597 -2.79463745

H -1.32251285 1.41895774 -2.21868463

H -0.21324475 0.76110721 -3.41184824

H	1.05644747	2.35946169	-2.05729750
H	2.45689785	-1.44550948	-0.87829312
H	0.22544888	-2.49808961	-1.34170310
N	-1.82173336	-0.72675813	-0.84348764
C	-1.52727484	-0.17505496	0.51004220
H	-2.36659304	-0.44722877	1.15137572
H	-0.63347936	-0.64550180	0.93104050
C	-1.40919776	1.33373293	0.49929982
C	-2.48654169	2.11994616	0.44114317
C	-1.90282180	-3.43897188	1.28713089
S	-2.89864408	-2.00668249	-0.85863118
O	-4.01491182	-1.63666849	0.01963458
O	-3.17433266	-2.33518886	-2.26144822
H	-1.69174897	-4.38931239	-1.99205365
H	-1.03720823	-4.50659405	2.94161279
H	-0.07568464	-6.30671565	1.51578882
H	-0.41769712	-6.25859982	-0.94934072
N	3.06211620	1.03512609	-0.94485743
C	3.54632805	2.36011064	-1.38511147
Pd	0.44277452	2.10535628	0.54569197
C	4.11579640	0.28739560	-0.20008472
H	3.72989494	-0.06566543	0.76050508
H	4.43365363	-0.58431551	-0.78427249
C	5.08160913	2.19697578	-1.30317963
C	5.25839377	1.30413933	-0.06109612
H	5.58268011	3.16271199	-1.22561999
H	5.44477485	1.68208760	-2.20014771
H	5.12892928	1.89907275	0.84982726
H	6.23371331	0.81308156	-0.01638730
C	3.07295743	3.57616756	-0.51974080
C	-1.17721490	-4.48320120	1.86424659
H	-3.48805722	1.68722168	0.36780178
C	-0.63843244	-5.49560111	1.06100259

O	2.31485102	3.38520527	0.48985042
O	3.52241099	4.69504818	-0.88378236
C	-2.07328846	-3.41637463	-0.10367155
H	-2.42699604	3.20487437	0.45832279
C	-0.82782010	-5.46853941	-0.32572294
H	-2.33079326	-2.66271728	1.91136561
C	-1.54385560	-4.42428140	-0.91773750
H	-1.44863074	3.07841059	5.29191332
H	2.77613497	1.36765640	2.22519610
H	3.20915481	2.54426598	-2.41023191
P	0.14595522	2.38154823	2.83536334
H	1.23714349	-0.14688146	3.85236023
C	-0.63920305	0.90525234	3.60230099
C	-2.03741557	0.80053690	3.70219261
C	-2.62655338	-0.39465765	4.12276120
C	-1.82989046	-1.50172194	4.43835091
C	-0.43776371	-1.40418239	4.33963048
C	0.15535645	-0.20807657	3.92589264
H	-2.66601006	1.64227993	3.43290259
H	-3.70927610	-0.46250422	4.19594364
H	-2.29171908	-2.43236778	4.75893512
H	0.18956145	-2.25820500	4.58275392
C	-0.85413116	3.82321766	3.34696709
C	-0.93617834	4.90052503	2.44885203
C	-1.64920590	6.05177604	2.79354359
C	-2.29030416	6.13151890	4.03532197
C	-2.21469652	5.05920927	4.93276099
C	-1.49906470	3.90787562	4.59271440
H	-0.44236607	4.82904832	1.48222897
H	-1.70922237	6.88070037	2.09253675
H	-2.85061510	7.02443392	4.30197399
H	-2.71343167	5.11816786	5.89714378
C	1.75099012	2.53192420	3.72132159

C	1.88181406	3.23333739	4.93021469
C	3.11458622	3.26819277	5.58965347
C	4.22185453	2.60117796	5.05180185
C	4.09724702	1.90572623	3.84363317
C	2.86996795	1.87912591	3.17757741
H	1.03102689	3.75538963	5.35691129
H	3.20880181	3.81635894	6.52406601
H	5.17833223	2.62933534	5.56818666
H	4.95594791	1.39255049	3.41756166

E2

COSMO(MeOH)-ZORA-M06/TZ2P//COSMO(MeOH)-ZORA-BLYP-D3(BJ)/TZ2P

E = -14722.92

G = -14347.92

COSMO(MeOH)-ZORA-BLYP-D3(BJ)/TZ2P

E = -11939.09

G = -11564.09

N_{imag} = 0

C	1.36320120	-1.57266344	0.82760405
C	0.97788289	-1.74735807	2.30786282
H	0.52418261	-1.14561426	0.26661875
H	1.74933449	-2.35911660	2.79425682
C	0.88936554	-0.44395296	3.12721977
C	2.59889282	-0.69179524	0.68052713
C	2.79174878	0.38036607	1.56539875
C	2.18123930	0.38166387	2.95833873
H	0.83745952	-0.73501592	4.17659210
H	2.02533629	1.40052707	3.31655765
H	2.92399607	-0.07377559	3.63164213
H	3.73806473	0.91142173	1.50820725
H	1.55900845	-2.55780718	0.39576100
H	0.04718527	-2.31294106	2.37625311
N	-0.34713337	0.39760672	2.95043696

C	-0.60202563	1.27674890	1.76711677
H	-1.46966521	1.88568639	2.03552102
H	-0.88420746	0.66503789	0.90092156
C	0.52158757	2.21460176	1.38720287
C	0.70750582	3.39738524	1.96931386
C	-3.36981091	-0.30242407	1.35072094
S	-1.77872883	-0.18363319	3.61571728
O	-2.59846743	0.98320521	3.96585363
O	-1.42509897	-1.14580190	4.66404253
H	-1.98820570	-3.00448560	2.92755269
H	-4.47734375	-0.37938146	-0.49155287
H	-4.22764288	-2.84140449	-0.75036619
H	-3.01115815	-4.15776159	0.97672650
N	3.52019779	-1.02124695	-0.25655041
C	3.18426260	-1.59084184	-1.57412683
Pd	1.57024756	1.51231829	-0.16498274
C	4.84480331	-0.35566136	-0.33907301
H	5.38939225	-0.54159603	0.59183499
H	4.72108018	0.72754349	-0.46100072
C	4.55792187	-2.10314956	-2.04824868
C	5.52256742	-0.98335425	-1.58463206
H	4.78333092	-3.04759868	-1.54069371
H	4.57955321	-2.26917793	-3.12663240
H	6.51961361	-1.36655380	-1.35249129
H	5.62848393	-0.22738100	-2.36894664
C	2.62235299	-0.50180948	-2.55251233
C	-3.93434778	-0.95435834	0.25297419
H	0.06817701	3.71933006	2.79559679
C	-3.79398930	-2.33896227	0.11008876
O	2.47023562	0.69485106	-2.12765177
O	2.40781874	-0.88872100	-3.73028480
C	-2.64873646	-1.04833502	2.29421361
H	1.48100430	4.09234319	1.65364596

C	-3.10231217	-3.07924543	1.07544162
H	-3.49529515	0.76705875	1.47812064
C	-2.52147701	-2.43660758	2.17249275
H	-2.47975040	2.56011326	-0.22341077
H	2.44805786	-2.39257455	-1.48436592
H	2.69723840	3.78004613	-1.84438397
P	0.09723805	2.57719948	-1.63668245
H	0.05535386	-0.32538286	-1.72443385
C	-0.95215690	3.91167011	-0.94268701
C	-0.51187648	5.24347068	-0.96207248
C	-1.23453818	6.23306962	-0.28825075
C	-2.40103236	5.90274590	0.41016068
C	-2.84807117	4.57569930	0.42651500
C	-2.12709650	3.58687468	-0.24321802
H	0.39500696	5.51075597	-1.49667196
H	-0.88492800	7.26257351	-0.31042683
H	-2.96028654	6.67345677	0.93502595
H	-3.75664496	4.31143962	0.96245481
C	0.96092214	3.35368571	-3.05497369
C	0.38559229	3.45505718	-4.32987154
C	1.08228236	4.09147248	-5.36194527
C	2.35095935	4.63413073	-5.12602061
C	2.92899532	4.53078471	-3.85509172
C	2.24007858	3.88553526	-2.82532047
H	-0.59519301	3.03347804	-4.52636034
H	0.63335988	4.16244483	-6.34990673
H	2.88976048	5.12848128	-5.93090599
H	3.91820421	4.94224373	-3.66859981
C	-1.11845055	1.37797522	-2.31686992
C	-2.30952093	1.80183748	-2.93408865
C	-3.22134613	0.86222004	-3.42230708
C	-2.95729725	-0.50681186	-3.29716676
C	-1.77565736	-0.93492191	-2.68390914

C	-0.86233420	0.00280335	-2.19846670
H	-2.53372748	2.86092927	-3.01810626
H	-4.14080610	1.20044911	-3.89393924
H	-3.67415082	-1.23511617	-3.66818444
H	-1.56933025	-1.99643963	-2.57421868

TS1

COSMO(MeOH)-ZORA-M06/TZ2P//COSMO(MeOH)-ZORA-BLYP-D3(BJ)/TZ2P

E = -14705.00

G = -14331.25

COSMO(MeOH)-ZORA-BLYP-D3(BJ)/TZ2P

E = -11922.30

G = -11548.55

$\boldsymbol{N}_{\mathbf{imag}}$ = 1, 300*i* cm^{-1}

C	0.91284420	-0.84120697	-0.77183880
C	-0.03181860	-1.23145719	-1.93998041
H	0.34870893	-0.76577652	0.16202814
H	0.58182461	-1.61116931	-2.76582544
C	-0.88513827	-0.08149535	-2.52376902
C	1.62564410	0.47820640	-1.01989129
C	0.83646356	1.56747565	-1.60981342
C	-0.00139291	1.13471711	-2.82094021
H	-1.31589670	-0.42573122	-3.46655350
H	-0.60994373	1.97421874	-3.16414256
H	0.69393155	0.86398394	-3.62833478
H	1.39337323	2.47493597	-1.83471901
H	1.63551642	-1.64503281	-0.62803331
H	-0.66787406	-2.05788556	-1.61328766
N	-2.03990084	0.35274196	-1.68937922
C	-1.76131122	0.93587022	-0.34805071
H	-2.70395612	1.32171860	0.04432410
H	-1.40914558	0.16487418	0.34315397
C	-0.78141657	2.08828164	-0.45200282

C	-1.19649906	3.33898689	-0.73977309
C	-3.31031528	-2.42979378	-0.19064958
S	-3.51549460	-0.35398705	-1.98414588
O	-4.47440029	0.27338605	-1.06871309
O	-3.75210962	-0.28945765	-3.43050842
H	-3.23437605	-2.75322984	-3.59561271
H	-3.05503239	-4.04256335	1.20957219
H	-2.75228844	-5.77103868	-0.55296935
H	-2.85130133	-5.13224038	-2.95625052
N	2.95308199	0.53428949	-0.90360734
C	3.79964548	1.66501640	-1.36291005
Pd	0.93658261	2.00543980	0.74445907
C	3.76742091	-0.49831134	-0.18556900
H	3.32208946	-0.70777301	0.78976485
H	3.78068448	-1.41850031	-0.77880763
C	5.21695977	1.04708372	-1.33436830
C	5.16554874	0.12805694	-0.09963030
H	5.98660853	1.81839937	-1.27426035
H	5.38133750	0.45926949	-2.24454578
H	5.26039460	0.71964275	0.81670594
H	5.94708535	-0.63544926	-0.10091634
C	3.75479366	2.95716732	-0.46794579
C	-3.09211208	-3.76252846	0.16100203
H	-2.26141243	3.57243110	-0.77737059
C	-2.92356573	-4.73494745	-0.83302987
O	3.20963089	2.90180048	0.67940984
O	4.32249679	3.95822324	-0.98410981
C	-3.35926717	-2.08658113	-1.54692393
H	-0.50516179	4.15807069	-0.91874415
C	-2.97823638	-4.37724675	-2.18484745
H	-3.44195515	-1.67280372	0.57486601
C	-3.19503539	-3.04572737	-2.55180831
H	0.76308304	4.29661173	2.61113225

H	3.25256229	1.31187400	2.37259553
H	3.49322891	1.94979754	-2.37174238
P	0.43303781	1.45919924	2.94504025
H	0.23840956	-1.48727573	2.98501078
C	1.82268713	0.63268802	3.84041752
C	1.64574420	-0.07395788	5.04252443
C	2.74227878	-0.65447126	5.68472436
C	4.02603068	-0.53310739	5.13549659
C	4.20891084	0.17367911	3.94255283
C	3.11193750	0.75423000	3.29601585
H	0.65467791	-0.18265884	5.47327168
H	2.59580833	-1.20295708	6.61238735
H	4.87689464	-0.98899492	5.63625282
H	5.20348770	0.27144436	3.51317964
C	-0.97602439	0.29910297	3.14081273
C	-2.29167733	0.78978881	3.21606341
C	-3.37535317	-0.09191215	3.24422931
C	-3.16078978	-1.47439826	3.19609233
C	-1.85499495	-1.96926168	3.09849870
C	-0.77017804	-1.09021109	3.06078862
H	-2.46995396	1.86076586	3.25722685
H	-4.38717941	0.30132215	3.30773413
H	-4.00471110	-2.15907616	3.22720289
H	-1.67736491	-3.04068872	3.05317584
C	0.00736563	2.87549944	4.04627356
C	-0.56764307	2.70858682	5.31796694
C	-0.81757939	3.81891088	6.12774890
C	-0.49407203	5.10623081	5.67708317
C	0.07668797	5.27954970	4.41189781
C	0.32348511	4.16797667	3.59838071
H	-0.82714389	1.71601078	5.67467202
H	-1.26398676	3.68131084	7.10991929
H	-0.68992766	5.96880657	6.30972124

H	0.32556447	6.27646289	4.05566112

TS2

COSMO(MeOH)-ZORA-M06/TZ2P//COSMO(MeOH)-ZORA-BLYP-D3(BJ)/TZ2P

E = -14711.96

G = -14336.10

COSMO(MeOH)-ZORA-BLYP-D3(BJ)/TZ2P

E = -11928.42

G = -11552.56

$\boldsymbol{N}_{\mathbf{imag}}$ = 1, 411*i* cm^{-1}

C	0.82606848	-1.09223960	1.59493203
C	-0.22342428	-1.59934410	2.60622788
H	0.64237887	-1.55295019	0.61982023
H	0.15360342	-1.47802340	3.62857368
C	-1.55356850	-0.82917197	2.53333028
C	0.85595187	0.43126327	1.44834257
C	-0.32395320	1.22377397	1.77232256
C	-1.25005301	0.64259754	2.83504802
H	-2.24705688	-1.22257987	3.28383611
H	-2.15709184	1.24684746	2.91137286
H	-0.74893920	0.68144187	3.81064396
H	-0.12067133	2.27692489	1.91821949
H	1.81549606	-1.43596739	1.90766908
H	-0.37448917	-2.67053089	2.45609356
N	-2.18968168	-0.91783198	1.17656014
C	-2.73952368	0.30941988	0.55061310
H	-3.60127189	0.67696231	1.12638822
H	-3.10671203	0.03341072	-0.43809469
C	-1.69567590	1.40856789	0.39605576
C	-2.09994700	2.69174189	0.26820744
C	-0.87758717	-2.56115608	-1.36095878
S	-2.74266272	-2.39860151	0.66105785
O	-3.78664861	-2.15917912	-0.34287402

O	-3.04596202	-3.22711493	1.83552075
H	-1.13922425	-4.75149931	1.24960360
H	0.61358083	-2.63993755	-2.90118741
H	1.71978882	-4.67230604	-1.97779468
H	0.83935133	-5.73394370	0.09408937
N	2.06986745	1.03482423	1.32212461
C	3.26062116	0.35614192	0.75435072
Pd	-0.00920096	0.69298834	-0.68583907
C	2.29027947	2.49375137	1.47694492
H	1.83015754	2.84915397	2.40257516
H	1.84501048	3.04166914	0.63451562
C	4.24946863	1.52196588	0.48735142
C	3.81755825	2.61737309	1.47567554
H	5.28545899	1.20084270	0.61223306
H	4.12134311	1.87680354	-0.54280946
H	4.20904756	2.40802511	2.47799417
H	4.14656923	3.61636053	1.17631697
C	3.05602071	-0.46463961	-0.55829964
C	0.22925389	-3.11259400	-2.00443942
H	-3.15766263	2.94926921	0.23868977
C	0.85020051	-4.25197965	-1.47931646
O	2.03859797	-0.27358562	-1.29483108
O	4.01434432	-1.24323188	-0.82171595
C	-1.35381551	-3.15975060	-0.18852411
H	-1.39363346	3.51146002	0.17404019
C	0.35924536	-4.84782574	-0.31277469
H	-1.34777951	-1.66519872	-1.74942653
C	-0.75096911	-4.30309093	0.34138389
H	0.80608994	3.16559464	-1.49497172
H	1.55717867	-0.34108746	-3.37970310
H	3.67622341	-0.33098561	1.50089030
P	-0.82026565	1.18799950	-2.84091599
H	-2.88081473	-0.87324305	-3.16946144

C	-2.64971145	1.26981783	-2.93405197
C	-3.33347313	2.48043370	-2.73671000
C	-4.72891875	2.49643674	-2.63834755
C	-5.45615943	1.30581482	-2.73337439
C	-4.78185513	0.09394887	-2.93187308
C	-3.38976869	0.07547689	-3.02513333
H	-2.77858466	3.40942729	-2.65891856
H	-5.24535834	3.44084127	-2.48273950
H	-6.54052783	1.31984735	-2.65360194
H	-5.33760877	-0.83753788	-3.00214186
C	-0.26537400	2.87568825	-3.33681133
C	-0.61448038	3.43678873	-4.57673280
C	-0.17094853	4.71669203	-4.91817454
C	0.62782658	5.44613132	-4.02669692
C	0.98359591	4.89075951	-2.79320391
C	0.53751127	3.60989719	-2.45141031
H	-1.23148035	2.87718332	-5.27444373
H	-0.44620370	5.14533203	-5.87901504
H	0.97232713	6.44187518	-4.29559675
H	1.60527954	5.45185361	-2.09943312
C	-0.33881462	0.15830710	-4.29202950
C	-1.15725851	-0.00788412	-5.42289930
C	-0.70899531	-0.76343924	-6.51034103
C	0.56064749	-1.35344228	-6.48275816
C	1.38032007	-1.18660866	-5.36065814
C	0.93399491	-0.43920618	-4.26589165
H	-2.14370013	0.44422569	-5.45565606
H	-1.35142342	-0.89004349	-7.37886500
H	0.90592105	-1.94250950	-7.32928436
H	2.36427531	-1.64975217	-5.33077022

F1

COSMO(MeOH)-ZORA-M06/TZ2P//COSMO(MeOH)-ZORA-BLYP-D3(BJ)/TZ2P

E = -14748.35

G = -14370.77

COSMO(MeOH)-ZORA-BLYP-D3(BJ)/TZ2P

E = -11963.79

G = -11586.21

N_{imag} = 0

C	1.11915039	-0.64990211	-0.29975217
C	0.86482644	-1.42410548	-1.61651520
H	0.21843363	-0.67006624	0.31047816
H	1.81635580	-1.58010697	-2.14007276
C	-0.07031705	-0.67388688	-2.58516727
C	1.50906843	0.79602225	-0.58040916
C	0.53788969	1.53054845	-1.53316542
C	0.49048148	0.72514370	-2.86659968
H	-0.14356578	-1.23284023	-3.51893095
H	-0.13755097	1.25628686	-3.58874140
H	1.49502176	0.62455033	-3.28959437
H	0.91402158	2.53269976	-1.72995161
H	1.88659567	-1.15817958	0.28114451
H	0.46820577	-2.41354013	-1.37428114
N	-1.48199436	-0.49708029	-2.10640667
C	-1.74819183	0.44220888	-0.97710187
H	-2.80248338	0.72087580	-1.01400993
H	-1.57847903	-0.06170835	-0.01559154
C	-0.89098829	1.68478665	-1.05264214
C	-1.40485120	2.88407907	-0.76340213
C	-2.97367675	-2.37339233	0.44211828
S	-2.55034023	-1.77352214	-2.22753705
O	-3.89733026	-1.19455511	-2.17981372
O	-2.14145424	-2.56670294	-3.38932985
H	-1.15306043	-4.26777126	-1.74954294
H	-3.26454680	-2.83063617	2.52094953
H	-1.91349012	-4.91951182	2.44222968

H	-0.85815514	-5.63906860	0.30490815
N	2.83317057	1.01055270	-0.85965233
C	3.36733530	2.21236238	-1.52720079
Pd	1.43567363	1.95985947	1.33213645
C	3.92240472	0.20627799	-0.23160430
H	3.65672882	-0.03957437	0.79850164
H	4.05764511	-0.72487381	-0.79576878
C	4.88391493	1.92423495	-1.61190816
C	5.15491310	1.11199835	-0.33349697
H	5.45942312	2.84838460	-1.67878442
H	5.09241227	1.31870861	-2.50135507
H	5.20155689	1.77825470	0.53610051
H	6.08276369	0.53599292	-0.37766448
C	3.07477669	3.59067370	-0.81779422
C	-2.80126173	-3.14728907	1.59210535
H	-2.45156312	3.00770244	-0.49553100
C	-2.04033182	-4.32017004	1.54456277
O	2.35948405	3.62871005	0.24263677
O	3.56877593	4.59225911	-1.38939819
C	-2.36784374	-2.78716560	-0.75230721
H	-0.78501089	3.77705594	-0.77081427
C	-1.44333647	-4.72423627	0.34481245
H	-3.58499830	-1.47831099	0.47324229
C	-1.60142815	-3.95721305	-0.81164378
H	-0.95377784	-1.11157683	4.81937089
H	0.91823621	3.30783109	3.91002359
H	2.93399113	2.30903262	-2.52698070
P	0.68834226	0.58845600	2.96472848
H	3.41152082	-0.27112418	3.39045805
C	1.14683995	1.27812400	4.61630266
C	1.44380623	0.46717042	5.72384254
C	1.73264643	1.04828017	6.96236617
C	1.72303581	2.44095416	7.10742975

C	1.42643019	3.25437438	6.00723221
C	1.14297137	2.67515719	4.76713217
H	1.45919373	-0.61383331	5.62105529
H	1.96561011	0.41282340	7.81369174
H	1.94979871	2.88967901	8.07168455
H	1.42154497	4.33672202	6.11201012
C	-1.11914735	0.29222463	3.17443176
C	-2.00853514	1.00598076	2.35958822
C	-3.39075970	0.84920748	2.51052825
C	-3.89162976	-0.01470767	3.48870800
C	-3.00747469	-0.71523553	4.32122290
C	-1.62818185	-0.56380664	4.16736628
H	-1.60884020	1.69037860	1.61700860
H	-4.07198636	1.40265703	1.86853224
H	-4.96496497	-0.14139668	3.60743610
H	-3.39452351	-1.38173308	5.08827758
C	1.45095059	-1.08591799	2.96942972
C	0.73629630	-2.24749290	2.64333906
C	1.40272874	-3.46882485	2.49657456
C	2.78659244	-3.54359332	2.67845698
C	3.50661698	-2.38772796	3.01092824
C	2.84458554	-1.16739663	3.14954573
H	-0.33574190	-2.20415951	2.48591127
H	0.83826283	-4.35878862	2.23328754
H	3.30294349	-4.49295300	2.55987992
H	4.58349903	-2.43592220	3.15368216

F2

COSMO(MeOH)-ZORA-M06/TZ2P//COSMO(MeOH)-ZORA-BLYP-D3(BJ)/TZ2P

E = -14738.68

G = -14362.16

COSMO(MeOH)-ZORA-BLYP-D3(BJ)/TZ2P

E = -11953.53

G = -11577.01

N_{imag} = 0

C	0.93308307	-0.83604001	0.20518151
C	0.40120958	-1.31132430	1.58502266
H	0.24214676	-1.12224556	-0.58584327
H	1.20329901	-1.22850431	2.32836592
C	-0.78773895	-0.48586230	2.11590749
C	1.11651621	0.68394060	0.19172993
C	-0.16025348	1.44952721	0.62155082
C	-0.44401235	1.01233339	2.09842768
H	-1.00594671	-0.79533074	3.13902725
H	-1.27621828	1.59949097	2.49787208
H	0.43553499	1.18095175	2.72721858
H	0.03482620	2.52038375	0.58133790
H	1.86359523	-1.35689225	-0.01668340
H	0.14349988	-2.37087190	1.51875984
N	-2.06923164	-0.65898448	1.35646024
C	-2.10744000	-0.14910740	-0.04637341
H	-3.15217350	-0.07383073	-0.34960505
H	-1.62133769	-0.86486154	-0.71959129
C	-1.46446105	1.21836099	-0.12043331
C	-2.15185806	2.24028404	-0.63668121
C	-2.75561271	-3.39407282	-0.69942559
S	-3.03594540	-1.98617743	1.66376212
O	-4.37202732	-1.64948461	1.16075725
O	-2.85667261	-2.32864182	3.07695982
H	-1.32987468	-4.24422574	2.29268516
H	-2.46245086	-4.45402557	-2.55122232
H	-0.91565449	-6.13000587	-1.56127478
H	-0.36444195	-6.03953961	0.86417851
N	2.26608257	1.13050828	0.80507360
C	3.47903871	0.30404376	1.01907349
Pd	1.84907059	1.06006104	-1.87966502

C	2.51448365	2.54417374	1.19703088
H	1.62252615	2.99616124	1.62847605
H	2.80639962	3.12191148	0.30856353
C	4.51718852	1.29513177	1.61321277
C	3.66654073	2.43284675	2.19770485
H	5.15578925	0.80163766	2.34803035
H	5.15561101	1.68163230	0.81024569
H	3.27656806	2.15749305	3.18471280
H	4.21869788	3.37182391	2.29243845
C	4.09809595	-0.39828199	-0.22884868
C	-2.20591399	-4.40190294	-1.49657672
H	-3.14113836	2.10058892	-1.06513897
C	-1.33971611	-5.34799905	-0.93682524
O	3.70470134	-0.06109964	-1.39859116
O	4.99907085	-1.23518357	0.02337369
C	-2.41987906	-3.34413438	0.66055429
H	-1.74786310	3.24477824	-0.64875240
C	-1.02579616	-5.29652715	0.42650981
H	-3.44977994	-2.67440016	-1.12030862
C	-1.56303433	-4.29089482	1.23404627
H	-2.08121709	3.74542338	-3.91034349
H	1.80201075	0.46831445	-5.16526992
H	3.24699201	-0.48624561	1.74066472
P	0.40505727	2.11681944	-3.24298985
H	-0.76398474	-0.40786569	-2.54775144
C	1.41154611	2.55333292	-4.73256963
C	1.65761483	3.87664900	-5.12844620
C	2.45952429	4.13993207	-6.24584833
C	3.01903635	3.08692382	-6.97649479
C	2.77656692	1.76259476	-6.58533355
C	1.98271911	1.49743719	-5.46867676
H	1.22433399	4.70197006	-4.57244421
H	2.64167434	5.16949050	-6.54533264

H	3.64080257	3.29385836	-7.84414426
H	3.20912919	0.93888182	-7.14835326
C	-0.27205641	3.74470937	-2.72160395
C	0.48351031	4.46186647	-1.77823067
C	0.04472620	5.70360151	-1.31323925
C	-1.16207997	6.23751190	-1.78145322
C	-1.91732646	5.52922578	-2.72315729
C	-1.47379051	4.29023240	-3.19593762
H	1.40869850	4.03032671	-1.40250597
H	0.63579948	6.24681359	-0.57995315
H	-1.51396561	7.19699247	-1.41062317
H	-2.85716549	5.93768490	-3.08665788
C	-0.96840749	1.15572342	-4.00089748
C	-1.62795409	1.56924211	-5.17159966
C	-2.65338837	0.78831503	-5.71225362
C	-3.01852712	-0.41863716	-5.10071560
C	-2.34191667	-0.85128662	-3.95528118
C	-1.31902990	-0.06806064	-3.41520001
H	-1.33144226	2.48717216	-5.67139522
H	-3.16177366	1.11679651	-6.61558891
H	-3.81498680	-1.02469269	-5.52541290
H	-2.60148668	-1.79814838	-3.48914047

TS3

COSMO(MeOH)-ZORA-M06/TZ2P//COSMO(MeOH)-ZORA-BLYP-D3(BJ)/TZ2P

E = -14717.40

G = -14345.01

COSMO(MeOH)-ZORA-BLYP-D3(BJ)/TZ2P

E = -11932.13

G = -11559.74

N_{imag} = 1, 806*i* cm^{-1}

C	1.64586244	-0.94222877	-1.31362273
C	0.65647133	-1.81494491	-2.09786208

H	0.92792050	-1.01549556	0.06861213
H	1.25169363	-2.52412280	-2.68980901
C	-0.23943504	-1.06232637	-3.10517658
C	1.94843074	0.39146137	-1.75881103
C	1.06879335	1.05409165	-2.83771546
C	0.60711245	-0.02637260	-3.84712853
H	-0.62164395	-1.79463416	-3.82020176
H	0.02960630	0.45025523	-4.64513643
H	1.47233053	-0.52677980	-4.29524370
H	1.68427660	1.78348608	-3.36472468
H	2.49999661	-1.50956862	-0.95256191
H	0.04193665	-2.41034135	-1.42027086
N	-1.44815210	-0.36085948	-2.56243324
C	-1.23400255	0.86490402	-1.73122566
H	-2.18545239	1.39095318	-1.64922011
H	-0.91561743	0.57931211	-0.70979607
C	-0.18134621	1.75710243	-2.33579288
C	-0.35619167	3.07558139	-2.44809538
C	-2.85324362	-3.72883060	-3.36603933
S	-2.70735902	-1.32774341	-2.01042785
O	-2.31463647	-2.20622139	-0.89511695
O	-3.84362477	-0.42647172	-1.77394420
H	-3.60972668	-0.65049016	-4.66150189
H	-2.99756805	-5.58992393	-4.44096882
H	-3.79206408	-4.52434167	-6.54369810
H	-4.10085140	-2.05807839	-6.65757766
N	3.12550587	1.00324835	-1.45652241
C	3.31483972	2.47142943	-1.50526342
Pd	0.99188355	0.49925414	0.53570025
C	4.15189661	0.36167199	-0.58370354
H	3.67364949	-0.13948586	0.26488973
H	4.69502228	-0.38841784	-1.17134485
C	4.81639726	2.61843147	-1.18002171

C	5.05085046	1.52254888	-0.12316274
H	5.05353115	3.62301412	-0.82603931
H	5.40463579	2.41710961	-2.08282340
H	4.72287511	1.87465032	0.86107865
H	6.09835353	1.22001648	-0.04597563
C	2.42794572	3.30543143	-0.50915822
C	-3.13289479	-4.51281449	-4.49016992
H	-1.28129728	3.55373176	-2.13417402
C	-3.57970761	-3.91144784	-5.67141217
O	2.55430855	4.55464997	-0.64769500
O	1.68007194	2.74095231	0.35213888
C	-3.03283656	-2.34576986	-3.44922726
H	0.42877202	3.72073786	-2.83455895
C	-3.75406056	-2.52211157	-5.73809330
H	-2.50070256	-4.17660919	-2.44294563
C	-3.48251853	-1.72799317	-4.62312842
H	-2.35104946	-0.45163366	1.05482693
H	1.70658169	2.64434276	2.63586049
H	-4.57987548	0.53793445	0.69498136
P	-0.02608560	0.34137526	2.58434777
H	1.49413901	-2.10898426	2.34703045
C	0.76388689	1.23909283	3.98309337
C	0.58634153	0.86217204	5.32434375
C	1.18936563	1.60171770	6.34639050
C	1.97028049	2.72238807	6.03793474
C	2.15162751	3.09954921	4.70195491
C	1.55771751	2.35847436	3.67516472
H	-0.01681065	-0.00593935	5.57330325
H	1.04982857	1.30232062	7.38268638
H	2.43865040	3.29527078	6.83493636
H	2.76139949	3.96663528	4.45790857
C	-0.32206767	-1.35346711	3.23364250
C	-1.45078786	-1.67148634	4.00661672

C	-1.62240029	-2.96894989	4.49968833
C	-0.66605485	-3.95524746	4.23178778
C	0.46237802	-3.64258671	3.46351888
C	0.62972345	-2.34900733	2.96186177
H	-2.19879165	-0.91187475	4.21516217
H	-2.50264142	-3.20843990	5.09186686
H	-0.80256207	-4.96427224	4.61381240
H	1.20479679	-4.40716367	3.24669980
C	-1.71220743	1.06510248	2.46554930
C	-2.07826469	2.24233123	3.13473073
C	-3.34506262	2.80239634	2.92813824
C	-4.25196000	2.19129295	2.05610717
C	-3.88977867	1.01387313	1.38657563
C	-2.62763652	0.45556905	1.58666387
H	-1.37994044	2.72357291	3.81308734
H	-3.62019383	3.71608591	3.45033476
H	-5.23376006	2.63034578	1.89444761
H	3.09217013	2.85254072	-2.50434127

TS4

COSMO(MeOH)-ZORA-M06/TZ2P//COSMO(MeOH)-ZORA-BLYP-D3(BJ)/TZ2P

E = -14710.63

G = -14339.46

COSMO(MeOH)-ZORA-BLYP-D3(BJ)/TZ2P

E = -11925.60

G = -11554.43

$\boldsymbol{N}_{\mathbf{imag}}$ = 1, 823i cm^{-1}

C	0.81055257	-0.87107507	0.93059190
C	-0.24544528	-1.54992300	1.84251142
H	-0.15841633	-0.94476309	-0.33511371
H	0.31645182	-2.13431214	2.58379208
C	-1.16185352	-0.59988388	2.64894329
C	1.21955886	0.47513920	1.27108922

C	0.17500314	1.37734858	1.93281717
C	-0.37421374	0.60732876	3.15987637
H	-1.55606805	-1.16189214	3.49938428
H	-1.02318423	1.26292141	3.74835125
H	0.45402382	0.27586813	3.79574675
H	0.65078983	2.29171602	2.27744239
H	1.62764531	-1.55725594	0.71512115
H	-0.85497363	-2.26117590	1.28054219
N	-2.34404243	-0.05088231	1.92697171
C	-2.07084855	0.78607845	0.72834249
H	-2.99053809	1.30335849	0.45289902
H	-1.77936113	0.15491040	-0.11988189
C	-0.96802147	1.78818914	1.00346024
C	-0.99116660	2.99733506	0.43444500
C	-4.13915475	-2.42833526	4.22474259
S	-3.71523018	-1.00098051	1.88978332
O	-3.46767966	-2.34915759	1.35263195
O	-4.76281066	-0.19669761	1.24935760
H	-4.23314187	0.97891487	3.91515025
H	-4.50209737	-3.51277296	6.05055716
H	-4.91610543	-1.46433233	7.40035180
H	-4.78578454	0.78144386	6.33549862
N	2.48152462	0.90796312	1.16666180
C	3.59824940	0.07786244	0.64965673
Pd	0.71508721	-0.03102631	-1.27375600
C	2.98012086	2.29304179	1.36963767
H	2.34940759	2.86075688	2.04871045
H	2.99775235	2.79296126	0.39270134
C	4.88861173	0.80731253	1.15109254
C	4.39067672	2.05599797	1.91738631
H	5.48610946	0.14735333	1.78380212
H	5.50566118	1.09540383	0.29601295
H	4.33202000	1.85325861	2.99217415

H	5.03559683	2.92563380	1.76574787
C	3.64871132	-0.03081324	-0.91470202
C	-4.44811259	-2.53202035	5.58547938
H	-1.80932980	3.30382096	-0.21249763
C	-4.68030566	-1.37919587	6.34256544
O	4.46462265	-0.88684969	-1.34991037
O	2.95353269	0.78568439	-1.60731362
C	-4.06959375	-1.15969458	3.64418791
H	-0.18398383	3.71054951	0.58376524
C	-4.60702731	-0.11314753	5.74483205
H	-3.95032971	-3.31169046	3.62387041
C	-4.30061370	0.00413897	4.38854861
H	-2.46664615	-1.52402407	-2.37057029
H	0.63685957	2.79701598	-1.95394375
H	3.51707827	-0.92750068	1.06875061
P	-0.49721871	0.44514825	-3.16022325
H	1.66149885	-1.19414832	-4.10417833
C	-0.05275566	2.08620938	-3.87037486
C	-0.23892526	2.40769657	-5.22443615
C	0.09223830	3.68229222	-5.69521207
C	0.60608558	4.64611771	-4.81877359
C	0.79702314	4.32962121	-3.46843212
C	0.47634144	3.05314108	-2.99851174
H	-0.63924054	1.66728810	-5.91111131
H	-0.05242665	3.92317339	-6.74594006
H	0.86172623	5.63658235	-5.18791532
H	1.20183530	5.07261644	-2.78505646
C	-0.38658423	-0.70794939	-4.58620190
C	-1.45339516	-0.93619503	-5.47036332
C	-1.29215418	-1.80436484	-6.55522320
C	-0.06546045	-2.44457134	-6.76768741
C	1.00191443	-2.21947262	-5.88887200
C	0.84018597	-1.35999517	-4.79898294

H	-2.40762772	-0.44247336	-5.31015162
H	-2.12377875	-1.97925789	-7.23402203
H	0.05684190	-3.12046705	-7.61084020
H	1.95445895	-2.71972812	-6.04694805
C	-2.29988239	0.58548291	-2.82659379
C	-2.97366357	1.81440927	-2.84602639
C	-4.31761489	1.88675533	-2.45657164
C	-4.99673131	0.73404551	-2.05442383
C	-4.32900589	-0.49794230	-2.03969627
C	-2.98917416	-0.57090582	-2.41527743
H	-2.45378540	2.71612170	-3.15481396
H	-4.82853140	2.84697200	-2.46646806
H	-6.03568475	0.79310134	-1.74032186
H	-4.84625387	-1.39443361	-1.70885265

G1

COSMO(MeOH)-ZORA-M06/TZ2P//COSMO(MeOH)-ZORA-BLYP-D3(BJ)/TZ2P

E = -14735.62

G = -14360.89

COSMO(MeOH)-ZORA-BLYP-D3(BJ)/TZ2P

E = -11951.44

G = -11576.71

$\boldsymbol{N}_{\mathbf{imag}}$ = 0

C	1.61416238	-0.59618358	-0.35450428
C	0.95883272	-1.79167404	-1.03200356
H	0.35606863	-1.07900702	1.94076735
H	1.75481636	-2.45548256	-1.40337045
C	0.07774303	-1.45155437	-2.25305676
C	1.67076616	0.66080669	-0.96773901
C	0.83946730	0.93715170	-2.22965680
C	0.76805321	-0.35936315	-3.07668059
H	-0.04600385	-2.35040892	-2.85714785
H	0.21628600	-0.15992003	-4.00048812

H	1.77670993	-0.69729180	-3.33921274
H	1.35186010	1.71072646	-2.80571393
H	2.42853324	-0.86559714	0.31173094
H	0.39542146	-2.37921020	-0.30560129
N	-1.31530076	-0.97692989	-1.95577865
C	-1.47221486	0.36076357	-1.31051506
H	-2.51960800	0.65627671	-1.38307656
H	-1.20265863	0.28886098	-0.23965446
C	-0.59239546	1.38275624	-1.98099032
C	-1.06385467	2.56524708	-2.38058018
C	-3.13532259	-1.50023252	1.04946096
S	-2.49447277	-2.09621199	-1.57089568
O	-3.78302452	-1.45711416	-1.86307048
O	-2.13671653	-3.34459464	-2.25165948
H	-1.16308024	-4.10752893	0.04520475
H	-3.57447226	-1.00092139	3.09447743
H	-2.21315186	-2.85638806	4.03094704
H	-1.00599140	-4.40935872	2.51006799
N	2.49416661	1.64940431	-0.53312547
C	2.14075185	3.08065412	-0.64633976
Pd	0.41117908	0.37707865	1.44765770
C	3.41964686	1.44248583	0.61263906
H	2.89669535	0.91870209	1.42721302
H	4.25664842	0.81546765	0.28664265
C	3.40259353	3.77821954	-0.10703272
C	3.84325970	2.85974993	1.05264411
H	3.18991941	4.80105861	0.20951982
H	4.16550842	3.80601480	-0.89326495
H	3.31172049	3.13197665	1.96969212
H	4.91607573	2.92066959	1.25312519
C	0.85625822	3.46718014	0.16257196
C	-3.04840012	-1.68068577	2.43110436
H	-2.10911316	2.83420940	-2.24764919

C	-2.28214278	-2.72440591	2.95637386
O	0.22699507	2.58708789	0.83511579
O	0.51862129	4.67785351	0.07030006
C	-2.44428072	-2.37557025	0.20319697
H	-0.41519938	3.31083447	-2.83389282
C	-1.59876063	-3.59550644	2.10119414
H	-3.74346502	-0.70470049	0.63306163
C	-1.67645303	-3.42734624	0.71671883
H	-2.44013385	1.21721632	1.51812176
H	0.36047948	3.72252589	2.95827359
H	1.94873125	3.35005858	-1.68760801
P	-0.36287762	0.97281547	3.51328071
H	1.13597117	-1.43648472	4.09100513
C	0.86355202	2.09766735	4.29393472
C	1.71654266	1.67163942	5.32399509
C	2.69543215	2.53572209	5.82708091
C	2.82818372	3.82804637	5.30892088
C	1.97619287	4.25657091	4.28248658
C	1.00116780	3.39843372	3.77197311
H	1.61915987	0.67371896	5.73964563
H	3.34936751	2.19727764	6.62741561
H	3.58918868	4.49800774	5.70190654
H	2.07524517	5.25980426	3.87443787
C	-0.62563828	-0.36613077	4.74116741
C	-1.71983296	-0.37335901	5.61887870
C	-1.88107349	-1.42235363	6.53073609
C	-0.94509955	-2.46036412	6.58476091
C	0.15253194	-2.45309971	5.71504518
C	0.30466782	-1.41831729	4.79062287
H	-2.45524609	0.42343362	5.58544906
H	-2.74123274	-1.42763495	7.19576439
H	-1.07448209	-3.27528070	7.29271002
H	0.87943504	-3.26095105	5.74432182

C	-1.90776437	1.95287091	3.46763600
C	-2.27080406	2.80274092	4.52651247
C	-3.47126200	3.51568210	4.47025460
C	-4.31211160	3.39038574	3.35706169
C	-3.94566092	2.55745877	2.29369469
C	-2.74443062	1.84638396	2.34732360
H	-1.61720475	2.91068736	5.38763118
H	-3.74871019	4.16952247	5.29348986
H	-5.24480328	3.94765970	3.31552009
H	-4.58865915	2.47037017	1.42114636

G2

COSMO(MeOH)-ZORA-M06/TZ2P//COSMO(MeOH)-ZORA-BLYP-D3(BJ)/TZ2P

E = -14732.35

G = -14358.19

COSMO(MeOH)-ZORA-BLYP-D3(BJ)/TZ2P

E = -11947.39

G = -11573.23

$\boldsymbol{N}_{\mathbf{imag}}$ = 0

C	1.51340723	-0.91217200	0.90272213
C	0.74043857	-2.01337968	1.62919772
H	-0.59488037	-0.98867033	-1.00037022
H	1.47390100	-2.62856817	2.17089902
C	-0.29138203	-1.54560796	2.69091806
C	1.43250745	0.43097716	1.31969562
C	0.31289728	0.84479301	2.28718586
C	0.19455908	-0.26187863	3.36242755
H	-0.40048806	-2.33548664	3.43401899
H	-0.50756877	0.05249687	4.14123730
H	1.17159359	-0.43747644	3.82615078
H	0.61492010	1.76661736	2.78278687
H	2.47227300	-1.25232978	0.51522422
H	0.26446288	-2.68356135	0.91265479

N	-1.68157807	-1.26486304	2.20892925
C	-1.87634698	-0.12194898	1.27483603
H	-2.93389255	0.14306743	1.27347443
H	-1.59492090	-0.43031095	0.25745125
C	-1.04506883	1.08720022	1.64398942
C	-1.49413939	2.31802648	1.38143244
C	-3.07487613	-2.66734496	-0.73889273
S	-2.74205820	-2.53927395	2.00172137
O	-4.08948225	-1.95901577	1.98220669
O	-2.40101936	-3.54764006	3.00948004
H	-1.05800268	-4.72611005	1.10278049
H	-3.22425701	-2.68445810	-2.88856237
H	-1.56843671	-4.51063303	-3.17062558
H	-0.50310891	-5.55308908	-1.17701558
N	2.38070522	1.35168512	1.02669427
C	3.72807532	1.00520903	0.50837486
Pd	0.74086491	-0.24354111	-1.18245995
C	2.22316793	2.82235004	1.10288123
H	1.50289501	3.11523073	1.86388737
H	1.86350124	3.19643477	0.13285346
C	4.50999554	2.35671707	0.54101080
C	3.64638536	3.30144287	1.39656625
H	5.51903432	2.21693124	0.93374676
H	4.59831416	2.75189625	-0.47697556
H	3.86308198	3.17126024	2.46316131
H	3.79128135	4.35401014	1.13760692
C	3.82419418	0.41206545	-0.92356424
C	-2.75317810	-3.13026442	-2.01722090
H	-2.47035437	2.48345428	0.93347488
C	-1.82090751	-4.16061061	-2.17464060
O	4.96292777	-0.02098068	-1.24273286
O	2.81135445	0.45081755	-1.70217768
C	-2.44402333	-3.23847579	0.37300301

H	-0.89105735	3.19875854	1.58622711
C	-1.21587651	-4.74193043	-1.05444760
H	-3.81317280	-1.88556372	-0.59869646
C	-1.52170767	-4.28165164	0.22842452
H	-2.86748264	-0.28319461	-1.87857570
H	1.52744536	2.63952132	-2.23438579
H	4.16924666	0.27282592	1.19386256
P	-0.36408143	0.59405222	-3.00187170
H	0.70406019	-1.94572358	-3.81990670
C	0.59829545	1.79877128	-3.99366591
C	0.50618654	1.85781331	-5.39317546
C	1.23272726	2.82006788	-6.10190988
C	2.04881163	3.73010404	-5.41997605
C	2.14588235	3.67139062	-4.02413374
C	1.43061251	2.70543290	-3.31361410
H	-0.12703592	1.15752261	-5.92943643
H	1.15857182	2.85896357	-7.18608039
H	2.61144780	4.47795467	-5.97362500
H	2.78428705	4.37196096	-3.49120911
C	-0.98136106	-0.64957399	-4.19486555
C	-2.17693342	-0.48131555	-4.91093357
C	-2.59871253	-1.46681379	-5.80868331
C	-1.82327259	-2.61507615	-6.00724102
C	-0.62378950	-2.77914046	-5.30434003
C	-0.20900484	-1.80524935	-4.39362689
H	-2.78173409	0.40814106	-4.76232240
H	-3.53127023	-1.33646585	-6.35220329
H	-2.15489487	-3.38154325	-6.70338048
H	-0.02180222	-3.67232436	-5.45190970
C	-1.85674815	1.52474256	-2.46800210
C	-1.93321287	2.92421079	-2.51978736
C	-3.06387379	3.58328084	-2.02264989
C	-4.12587185	2.85405485	-1.47922282

C	-4.05654880	1.45518354	-1.43340759
C	-2.92745711	0.79836907	-1.91948011
H	-1.11874279	3.50260942	-2.94368211
H	-3.11238057	4.66873445	-2.06390839
H	-5.00162148	3.36954120	-1.09299770
H	-4.87921375	0.88042707	-1.01464987

H1

COSMO(MeOH)-ZORA-M06/TZ2P//COSMO(MeOH)-ZORA-BLYP-D3(BJ)/TZ2P

E = -14742.41

G = -14368.08

COSMO(MeOH)-ZORA-BLYP-D3(BJ)/TZ2P

E = -11955.01

G = -11580.68

$\boldsymbol{N}_{\mathbf{imag}}$ = 0

C	1.52922759	-0.47003890	-0.50593690
C	0.63064190	-1.46328712	-1.23602602
H	1.04510296	2.25613164	1.27799496
H	1.27181898	-2.17557076	-1.77762534
C	-0.32148740	-0.84610717	-2.28856157
C	1.64284238	0.85980462	-0.90113226
C	0.70197124	1.41874588	-1.97873633
C	0.39506585	0.30081939	-3.00723758
H	-0.60270423	-1.61829091	-3.00533074
H	-0.23008801	0.70324742	-3.81052199
H	1.32757259	-0.07426003	-3.44325626
H	1.20722510	2.23721064	-2.49452217
H	2.37612412	-0.92653259	0.00437992
H	0.06171717	-2.06564503	-0.52226402
N	-1.61613682	-0.29504812	-1.76473199
C	-1.52864657	0.88016892	-0.85016876
H	-2.53232407	1.27390834	-0.69592189
H	-1.11964388	0.56449732	0.13385817

C	-0.62791666	1.93132232	-1.44336515
C	-0.99873797	3.21127174	-1.52931256
C	-2.78468496	-1.58795519	1.24874455
S	-2.88863710	-1.34009294	-1.50368707
O	-4.07414514	-0.50267722	-1.28821498
O	-2.87776549	-2.30665302	-2.60646060
H	-1.92550820	-4.01610789	-1.00377794
H	-2.63923380	-1.78499273	3.38148086
H	-1.74806838	-4.09966388	3.30254908
H	-1.39509806	-5.22627996	1.10850601
N	2.65674556	1.67848214	-0.46100762
C	2.67258720	3.14226169	-0.56818901
Pd	0.60524236	0.13029383	1.53962466
C	3.63672930	1.20291304	0.54284446
H	3.09512347	0.71516100	1.37450551
H	4.29565070	0.45503521	0.08598027
C	4.11537456	3.49049470	-0.12226167
C	4.38275297	2.46989637	1.00085900
H	4.20676411	4.53064487	0.19697681
H	4.78681364	3.32384161	-0.97089403
H	3.96097305	2.83099556	1.94564427
H	5.44851627	2.28565542	1.15653942
C	1.65744028	3.91045881	0.31048955
C	-2.48526653	-2.27412546	2.42665394
H	-1.97653547	3.54361858	-1.18833705
C	-1.98367651	-3.57849162	2.37871167
O	0.98196611	3.27297386	1.27680226
O	1.50741179	5.11796515	0.15850053
C	-2.57857522	-2.23371745	0.02228533
H	-0.33299813	3.97139579	-1.93080734
C	-1.78117543	-4.21111854	1.14725169
H	-3.17912557	-0.57851847	1.28838381
C	-2.07681676	-3.53894645	-0.04134097

H	-2.86783659	0.07570002	4.92492237
H	2.49932401	1.42439028	3.90717690
H	2.49172582	3.47176622	-1.59339498
P	-0.26704498	0.77130918	3.51363833
H	0.32320265	-2.05287715	3.59263447
C	0.53837493	2.23447736	4.30310982
C	-0.17630951	3.31325160	4.84217952
C	0.50538419	4.39669050	5.40976960
C	1.90357472	4.40627716	5.45022804
C	2.62253126	3.32662004	4.91888090
C	1.94324088	2.25173079	4.34360334
H	-1.26208059	3.31630787	4.81270745
H	-0.05784043	5.23226534	5.81970820
H	2.43138864	5.25029592	5.88822231
H	3.71000586	3.32995379	4.94364008
C	-0.26092763	-0.44617238	4.89990282
C	-0.56058377	-0.08031092	6.22353804
C	-0.54261190	-1.03899668	7.24002312
C	-0.21807593	-2.37009573	6.94542586
C	0.09467909	-2.73794025	5.63208231
C	0.07621766	-1.77811022	4.61515443
H	-0.80341250	0.95213673	6.46093375
H	-0.77825249	-0.74860389	8.26132848
H	-0.20208359	-3.11415314	7.73838878
H	0.35659673	-3.76778136	5.40039586
C	-2.03114434	1.29081949	3.34433652
C	-2.33349116	2.19362139	2.30599069
C	-3.65412215	2.55725803	2.04358871
C	-4.69819593	2.01273045	2.80481064
C	-4.40618779	1.12104527	3.84085958
C	-3.07880116	0.76743815	4.11539829
H	-1.53010211	2.60117794	1.69782491
H	-3.87288167	3.25512876	1.23850834

H	-5.72923351	2.28215959	2.58905311
H	-5.20953215	0.69534458	4.43781817

H2

COSMO(MeOH)-ZORA-M06/TZ2P//COSMO(MeOH)-ZORA-BLYP-D3(BJ)/TZ2P

E = -14734.57

G = -14361.17

COSMO(MeOH)-ZORA-BLYP-D3(BJ)/TZ2P

E = -11948.86

G = -11575.46

$\boldsymbol{N}_{\mathbf{imag}}$ = 0

C	1.40673622	-0.99313721	1.06823754
C	0.59975013	-1.82832729	2.04762054
H	2.11707102	-0.34863669	-1.57798004
H	1.30096966	-2.37496087	2.69751182
C	-0.32514220	-1.02265977	2.98813874
C	1.48724230	0.39202169	1.14214792
C	0.60350992	1.15673710	2.15982333
C	0.39483635	0.26195352	3.40971390
H	-0.54380208	-1.63227376	3.86544366
H	-0.18982218	0.80802210	4.15710381
H	1.36167346	-0.00402015	3.85088699
H	1.15637206	2.05064092	2.45772231
H	2.20217850	-1.54140079	0.56807657
H	0.03513805	-2.59828172	1.51639243
N	-1.66852743	-0.62564231	2.44114222
C	-1.72971461	0.44324486	1.40391002
H	-2.75546778	0.80762890	1.34991536
H	-1.46414479	0.01868183	0.41266399
C	-0.77735263	1.57265213	1.69153823
C	-1.15598973	2.84917210	1.57712250
C	-2.98630797	-2.04900132	-0.46453901
S	-2.85769297	-1.79332802	2.29240936

O	-4.13071113	-1.07258158	2.17645943
O	-2.66132495	-2.74373728	3.39102370
H	-1.68283349	-4.36483861	1.69003888
H	-3.00067361	-2.23104798	-2.60428925
H	-1.85935399	-4.43883290	-2.61512309
H	-1.20732473	-5.51458114	-0.46571555
N	2.49027644	1.13847212	0.54614207
C	3.82060204	0.63484226	0.17505327
Pd	0.22124527	-0.44254813	-0.90885242
C	2.26764226	2.50556062	0.03366078
H	1.57606579	3.05283638	0.67440356
H	1.82802707	2.46380414	-0.97463906
C	4.55468087	1.90003844	-0.39927856
C	3.67834398	3.09687144	0.01907068
H	5.57939775	1.95455117	-0.02691576
H	4.59468598	1.83485430	-1.49198485
H	3.94796477	3.44172061	1.02361968
H	3.77254930	3.93621211	-0.67589562
C	3.99489987	-0.47841168	-0.87594148
C	-2.71325798	-2.70021582	-1.66965891
H	-2.17574302	3.12068998	1.31524143
C	-2.07024264	-3.94141233	-1.67230163
O	5.03924690	-1.12489350	-0.90224596
O	3.06885847	-0.67239960	-1.83141443
C	-2.60132594	-2.65978391	0.73680479
H	-0.45665464	3.66581567	1.74039889
C	-1.69933491	-4.54559511	-0.46554407
H	-3.49693049	-1.09244543	-0.46458161
C	-1.96123405	-3.90511890	0.74767683
H	-3.09194404	-0.32594193	-4.40481361
H	2.12163636	0.87199289	-3.69559794
H	4.31850838	0.25754853	1.07590101
P	-0.58812683	0.36497041	-2.85316402

H	-0.08994406	-2.45040759	-3.22197699
C	0.25558463	1.92402320	-3.37744263
C	-0.36631612	3.18060270	-3.38439248
C	0.37133563	4.33302505	-3.68504795
C	1.73186911	4.24117498	-3.99232351
C	2.35527824	2.98573106	-4.00573598
C	1.62441662	1.83826392	-3.69707127
H	-1.42274979	3.26974228	-3.15283799
H	-0.12312963	5.30193149	-3.68067818
H	2.30328439	5.13753575	-4.22117905
H	3.41224801	2.90273107	-4.24825560
C	-0.49152174	-0.67327988	-4.36729880
C	-0.66948344	-0.13710396	-5.65401947
C	-0.61699591	-0.97034905	-6.77444426
C	-0.38637918	-2.34411279	-6.61894512
C	-0.19951300	-2.88156534	-5.34048284
C	-0.24652448	-2.04674266	-4.21924735
H	-0.84738634	0.92818202	-5.77814033
H	-0.75567903	-0.55026149	-7.76803988
H	-0.34558641	-2.99035312	-7.49265559
H	-0.01012555	-3.94541180	-5.21704125
C	-2.36974414	0.81879268	-2.71788834
C	-2.75152982	1.62652537	-1.62974024
C	-4.09592490	1.91136491	-1.39191567
C	-5.08505379	1.37624536	-2.22851700
C	-4.71470955	0.57289498	-3.31133717
C	-3.36383928	0.30054637	-3.56090553
H	-1.98928934	2.02129401	-0.96359141
H	-4.37548201	2.53896943	-0.54882000
H	-6.13474422	1.58249293	-2.03422590
H	-5.47613925	0.15287812	-3.96453338

I1

COSMO(MeOH)-ZORA-M06/TZ2P//COSMO(MeOH)-ZORA-BLYP-D3(BJ)/TZ2P

E = -8688.97

G = -8466.90

COSMO(MeOH)-ZORA-BLYP-D3(BJ)/TZ2P

E = -6958.91

G = -6736.84

N_{imag} = 0

C	1.58846642	-0.59848575	-0.50240697
C	0.71154577	-1.69013628	-1.07164786
C	-2.11788133	-3.60346790	0.20618797
H	1.33578325	-2.52403826	-1.43187018
C	-0.18061761	-1.24314256	-2.25365887
C	1.68829403	0.64719573	-1.02424896
C	0.85759893	1.03857302	-2.25025808
C	0.57773794	-0.21998431	-3.10918627
H	-0.45234434	-2.10957933	-2.85732856
H	-0.01299597	0.05345656	-3.98966344
H	1.52298966	-0.66226114	-3.44318200
H	1.41142887	1.76299069	-2.85456881
H	2.17878229	-0.87452370	0.36662979
H	0.08852629	-2.12083165	-0.27891304
N	-1.47938982	-0.60066917	-1.85587658
C	-1.37536667	0.67972658	-1.08817013
H	-2.37671229	1.09471356	-0.97689769
H	-0.95088068	0.48747032	-0.09220853
C	-0.48484888	1.63245422	-1.85183691
C	-0.88033762	2.86540526	-2.17740597
C	-2.81721038	-1.49198644	1.21597837
S	-2.79744314	-1.56885672	-1.54489800
O	-3.96340314	-0.67928527	-1.48416673
O	-2.77192739	-2.65661331	-2.52776885
H	-1.94743184	-4.20227420	-0.68229376
H	-2.74327779	-1.41416550	3.36556962

H	-1.90261511	-3.74439998	3.60475305
H	-1.51993746	-5.14563623	1.58358389
N	2.56567883	1.61276105	-0.52843047
C	2.35568819	3.05281847	-0.64303659
H	2.15302764	3.35420973	-1.67332531
C	3.44928294	1.30580773	0.61439212
H	2.87529073	0.89844037	1.45964841
H	4.17839188	0.54396767	0.30998503
C	3.71351726	3.62078116	-0.15546498
C	4.10538683	2.65837139	0.98833824
H	3.64182544	4.66463205	0.15927180
H	4.42278022	3.55548522	-0.98657004
H	3.70317320	3.01908733	1.94065164
H	5.18987729	2.57192465	1.09687035
C	1.20892360	3.64239757	0.20069503
C	-2.56602856	-2.02366126	2.48336701
H	-1.86478553	3.23915877	-1.90433637
C	-2.09527307	-3.33571247	2.61603234
O	0.71776824	2.77902118	1.12521698
O	0.80621148	4.78999868	0.07629770
C	-2.58498500	-2.28930027	0.08729775
H	-0.22553255	3.54696563	-2.71583882
C	-1.87765073	-4.12451711	1.48024891
H	-3.19848263	-0.48185536	1.10860223
H	0.01631226	3.24833083	1.62730412

I2

COSMO(MeOH)-ZORA-M06/TZ2P//COSMO(MeOH)-ZORA-BLYP-D3(BJ)/TZ2P

E = -8687.32

G = -8464.66

COSMO(MeOH)-ZORA-BLYP-D3(BJ)/TZ2P

E = -6958.41

G = -6735.75

N

N_{imag} = 0

C	1.56292950	-1.33012231	1.77176654
C	0.58166314	-2.26682503	2.43353504
H	3.80735463	-0.31844827	0.26234077
H	1.09151128	-2.95886368	3.12427604
C	-0.49337817	-1.53029734	3.25373836
C	1.64457757	-0.00470790	2.05060603
C	0.78113384	0.62454276	3.14838613
C	0.20673603	-0.45532632	4.09422744
H	-1.00899894	-2.23956644	3.90185939
H	-0.48810751	0.01008311	4.80073164
H	1.01033554	-0.93465287	4.66420636
H	1.36958387	1.33964793	3.73083815
H	2.17596990	-1.76416568	0.98713030
H	0.11573914	-2.90674047	1.67804710
N	-1.58392916	-0.85675381	2.45466722
C	-1.27881726	0.41287644	1.70949528
H	-2.22752065	0.89597568	1.47126570
H	-0.75285822	0.17755962	0.77497373
C	-0.40788168	1.33114951	2.52630460
C	-0.67977681	2.62957146	2.67920185
C	-2.28010705	-1.23545030	-0.95220275
S	-2.74321020	-1.80795798	1.71857398
O	-3.93676648	-0.96927105	1.55143552
O	-2.84155399	-3.05224696	2.48763319
H	-1.44499152	-4.16073475	0.60774556
H	-1.83154908	-0.75818875	-3.00384428
H	-0.62969284	-2.89696774	-3.43053789
H	-0.46442575	-4.61075296	-1.63216093
N	2.36338537	0.89028667	1.26750913
C	3.04475519	0.44916623	0.05478435
H	0.38708163	-0.05016981	-1.76328898
C	2.83928410	2.21893179	1.72132882

H 3.11876408 2.19832564 2.77949194

H 2.05285810 2.97445296 1.58944679

C 3.72170235 1.73933128 -0.48036664

C 4.05093869 2.50228631 0.81610506

H 4.59757426 1.51059569 -1.09186930

H 3.00762402 2.30697496 -1.08773520

H 4.96139093 2.09300652 1.26937379

H 4.20133245 3.57249800 0.64699028

C 2.12320577 -0.16516445 -1.00254381

C -1.72794876 -1.49493076 -2.21083235

H -1.57291076 3.08003863 2.25105821

C -1.06162004 -2.70245123 -2.45243691

O 2.47364182 -1.05567496 -1.76373536

O 0.89846596 0.41659731 -1.06521205

C -2.15235695 -2.19619254 0.05962104

H -0.00931082 3.28355018 3.23269821

C -0.96354810 -3.66471746 -1.44063150

H -2.80992853 -0.30773439 -0.76665180

C -1.50895491 -3.41627655 -0.17872458

J

COSMO(MeOH)-ZORA-M06/TZ2P//COSMO(MeOH)-ZORA-BLYP-D3(BJ)/TZ2P

E = -6314.25

G = -6159.61

COSMO(MeOH)-ZORA-BLYP-D3(BJ)/TZ2P

E = -5060.45

G = -4905.81

$\boldsymbol{N}_{\mathbf{imag}}$ = 0

C 2.12729925 0.35809781 0.52960907

C 1.56665950 -0.82625642 -0.30036139

H -2.59332909 0.29024574 1.58284282

H 2.41061455 -1.40756902 -0.69074040

C 0.72042162 -0.41977643 -1.52887031

C	2.53474085	1.57047841	-0.29021673
C	1.67368744	1.90448516	-1.51763252
C	1.47881952	0.61950597	-2.36464001
H	0.53598942	-1.30895447	-2.13320399
H	0.92234050	0.86254905	-3.27554208
H	2.45111113	0.20674227	-2.65588326
H	2.20777825	2.67116375	-2.08566765
C	-1.40733190	-2.40331306	3.30583185
H	1.00283671	-1.49454544	0.35442199
N	-0.63013025	0.15711408	-1.23180355
C	-0.67103345	1.45927209	-0.50618635
H	-1.68255750	1.85821259	-0.58152296
H	-0.45108610	1.30839748	0.56099165
C	0.31220587	2.44455280	-1.10632434
C	-0.01495303	3.73139378	-1.26498087
C	-2.14774823	-0.68161012	1.76729000
S	-1.91257294	-0.88146124	-0.97739838
O	-3.12748730	-0.06269188	-1.04602981
O	-1.74072213	-2.00407040	-1.90312451
H	-0.95753621	-3.40707434	0.07419237
H	-2.24177791	-0.50268159	3.91003398
H	-1.26171783	-2.75236593	4.32486235
H	-0.63711375	-4.21333059	2.40931622
O	3.50291179	2.26469325	0.01094222
C	-1.23161938	-2.78104320	0.91684459
C	-1.77564370	-1.50974317	0.69949014
H	0.69960437	4.45107572	-1.65759964
C	-1.05322997	-3.22555636	2.22953125
C	-1.95658105	-1.13665691	3.07471307
H	-1.00389805	4.10200895	-1.00418691
H	2.98485825	0.04443022	1.13286605
H	1.36619064	0.71428146	1.23813615

8 References

(1) (a) te Velde, G.; Bickelhaupt, F. M.; Baerends, E. J.; Fonseca Guerra, C.; van Gisbergen, S. J. A.; Snijders, J. G.; Ziegler, T. Chemistry with ADF. *Journal of Computational Chemistry* **2001**, *22* (9), 931-967. (b) Fonseca Guerra, C.; Snijders, J. G.; te Velde, G.; Baerends, E. J. Towards an order-N DFT method. *Theoretical Chemistry Accounts* **1998**, *99* (6), 391-403.

(2) (a) Slater, J. C. *Quantum Theory of Molecules and Solids*; McGraw-Hill, 1974. (b) Becke, A. D. Density functional calculations of molecular bond energies. *The Journal of Chemical Physics* **1986**, *84* (8), 4524-4529. (c) Becke, A. D. Density-functional exchange-energy approximation with correct asymptotic behavior. *Physical Review A* **1988**, *38* (6), 3098-3100.

(3) Lee, C.; Yang, W.; Parr, R. G. Development of the Colle-Salvetti correlation-energy formula into a functional of the electron density. *Physical Review B* **1988**, *37* (2), 785-789.

(4) Van Lenthe, E.; Baerends, E. J. Optimized Slater-type basis sets for the elements 1–118. *Journal of Computational Chemistry* **2003**, *24* (9), 1142-1156.

(5) (a) Jong, G. T. d.; Solà, M.; Visscher, L.; Bickelhaupt, F. M. Ab initio benchmark study for the oxidative addition of CH4 to Pd: Importance of basis-set flexibility and polarization. *The Journal of Chemical Physics* **2004**, *121* (20), 9982-9992. (b) de Jong, G. T.; Bickelhaupt, F. M. Oxidative Addition of the Fluoromethane C−F Bond to Pd. An ab Initio Benchmark and DFT Validation Study. *The Journal of Physical Chemistry A* **2005**, *109* (42), 9685-9699. (c) de Jong, G. T.; Bickelhaupt, F. M. Oxidative Addition of the Chloromethane C−Cl Bond to Pd, an ab Initio Benchmark and DFT Validation Study. *Journal of Chemical Theory and Computation* **2006**, *2* (2), 322-335.

(6) (a) Klamt, A.; Schüürmann, G. COSMO: a new approach to dielectric screening in solvents with explicit expressions for the screening energy and its gradient. *Journal of the Chemical Society, Perkin Transactions 2* **1993**, (5), 799-805, 10.1039/P29930000799. (b) Klamt, A. Conductor-like Screening Model for Real Solvents: A New Approach to the Quantitative Calculation of Solvation Phenomena. *The Journal of Physical Chemistry* **1995**, *99* (7), 2224-2235. (c) Klamt, A.; Jonas, V. Treatment of the outlying charge in continuum solvation models. *The Journal of Chemical Physics* **1996**, *105* (22), 9972-9981. (d) Pye, C. C.; Ziegler, T. An implementation of the conductor-like screening model of solvation within the Amsterdam density functional package. *Theoretical Chemistry Accounts* **1999**, *101* (6), 396-408.

(7) (a) Grimme, S.; Antony, J.; Ehrlich, S.; Krieg, H. A consistent and accurate ab initio parametrization of density functional dispersion correction (DFT-D) for the 94 elements H-Pu. *The Journal of Chemical Physics* **2010**, *132* (15), 154104. (b) Becke, A. D.; Johnson, E. R. A density-functional model of the dispersion interaction. *The Journal of Chemical Physics* **2005**, *123* (15), 154101.

(8) (a) Lenthe, E. v.; Baerends, E. J.; Snijders, J. G. Relativistic regular two ‐ component Hamiltonians. *The Journal of Chemical Physics* **1993**, *99* (6), 4597-4610. (b) Lenthe, E. v.; Baerends, E. J.; Snijders, J. G. Relativistic total energy using regular approximations. *The Journal of Chemical Physics* **1994**, *101* (11), 9783-9792.

(9) Legault, C. Y. *CYLview*. 2009. http://www.cylview.org.

(10) Zhao, Y.; Truhlar, D. G. The M06 suite of density functionals for main group thermochemistry, thermochemical kinetics, noncovalent interactions, excited states, and transition elements: two new functionals and systematic testing of four M06-class functionals and 12 other functionals. *Theoretical Chemistry Accounts* **2008**, *120* (1), 215-241.

(11) Bottini, A. T.; Dev, V. Amines Derived from Dihalopropenes. II. Synthesis of (±)- and (-)-1-(2-Methylene-1-aziridinyl)-3-buten-2-ol1. **1962**, *27*, 6.

(12) Fort, D. A.; Woltering, T. J.; Nettekoven, M.; Knust, H.; Bach, T. Conformationally restricted pyrrolidines by intramolecular [2+2] photocycloaddition reactions. *Chemical Communications* **2013**, *49* (29), 2989-2991.

(13) Diaba, F.; Ricou, E.; Bonjoch, J. New Insights into NIS-Promoted Aminocyclization. Synthesis of Decahydroquinolines from 2-Allylcyclohexylamines. *Organic Letters* **2007**, *9* (14), 2633-2636.

(14) Manzano, R.; Datta, S.; Paton, R. S.; Dixon, D. J. Enantioselective Silver and Amine Co-catalyzed Desymmetrizing Cycloisomerization of Alkyne-Linked Cyclohexanones. *Angewandte Chemie International Edition* **2017**, *56* (21), 5834-5838.
(15) Zhou, H.; Che, X.; Bao, G.; Wang, N.; Peng, L.; Barnash, K. D.; Frye, S. V.; James, L. I.; Bai, X. Design, synthesis, and protein methyltransferase activity of a unique set of constrained amine containing compounds. *Bioorganic & Medicinal Chemistry Letters* **2016**, *26* (18), 4436-4440.
(16) Abdel-Magid, A. F.; Carson, K. G.; Harris, B. D.; Maryanoff, C. A.; Shah, R. D. Reductive Amination of Aldehydes and Ketones with Sodium Triacetoxyborohydride. Studies on Direct and Indirect Reductive Amination Procedures1. *The Journal of Organic Chemistry* **1996**, *61* (11), 3849-3862.
(17) Polishchuk, I.; Sklyaruk, J.; Lebedev, Y.; Rueping, M. Air Stable Iridium Catalysts for Direct Reductive Amination of Ketones. *Chemistry – A European Journal* **2021**, *27* (19), 5919-5922.
(18) Yao, B.; Miao, T.; Li, P.; Wang, L. Direct Synthesis of Benzo[f]indazoles from Sulfonyl Hydrazines and 1,3-Enynes by Copper-Catalyzed Annulation. *Organic Letters* **2019**, *21* (1), 124-128.
(19) Okutani, M.; Mori, Y. Conversion of Bromoalkenes into Alkynes by Wet Tetra-n-butylammonium Fluoride. *The Journal of Organic Chemistry* **2009**, *74* (1), 442-444.
(20) Bowman, W. R.; Bridge, C. F.; Brookes, P.; Cloonan, M. O.; Leach, D. C. Cascade radical synthesis of heteroarenes via iminyl radicals. *Journal of the Chemical Society, Perkin Transactions 1* **2002**, (1), 58-68.
(21) Feutren, S.; McAlonan, H.; Montgomery, D.; Stevenson, P. J. Palladium catalysed formal 6-endo-trig approaches to pumiliotoxin alkaloids: interception of the elusive cyclopropyl intermediate. *Journal of the Chemical Society, Perkin Transactions 1* **2000**, (7), 1129-1137.
(22) Iwasaki, K.; Wan, K. K.; Oppedisano, A.; Crossley, S. W. M.; Shenvi, R. A. Simple, Chemoselective Hydrogenation with Thermodynamic Stereocontrol. *Journal of the American Chemical Society* **2014**, *136* (4), 1300-1303.
(23) Diao, T.; Stahl, S. S. Synthesis of Cyclic Enones via Direct Palladium-Catalyzed Aerobic Dehydrogenation of Ketones. *Journal of the American Chemical Society* **2011**, *133* (37), 14566-14569.
(24) Nicolaou, K. C.; Gray, D. L. F.; Montagnon, T.; Harrison, S. T. Oxidation of Silyl Enol Ethers by Using IBX and IBX·N-Oxide Complexes: A Mild and Selective Reaction for the Synthesis of Enones. *Angewandte Chemie International Edition* **2002**, *41* (6), 996-1000.
(25) Ito, Y.; Hirao, T.; Saegusa, T. Synthesis of .alpha.,.beta.-unsaturated carbonyl compounds by palladium(II)-catalyzed dehydrosilylation of silyl enol ethers. *The Journal of Organic Chemistry* **1978**, *43* (5), 1011-1013.
(26) Larock, R. C.; Hightower, T. R.; Kraus, G. A.; Hahn, P.; Zheng, D. A simple, effective, new, palladium-catalyzed conversion of enol silanes to enones and enals. *Tetrahedron Letters* **1995**, *36* (14), 2423-2426.
(27) Lu, Y.; Nguyen, P. L.; Lévaray, N.; Lebel, H. Palladium-Catalyzed Saegusa–Ito Oxidation: Synthesis of α,β-Unsaturated Carbonyl Compounds from Trimethylsilyl Enol Ethers. *The Journal of Organic Chemistry* **2013**, *78* (2), 776-779.
(28) Matsuo, J.-i.; Aizawa, Y. One-pot β-substitution of enones with alkyl groups to β-alkyl enones. *Chemical Communications* **2005**, (18), 2399-2401.
(29) Kerr, W. J.; Pearson, C. M.; Thurston, G. J. Highly efficient methods for the one-pot synthesis of β-substituted enones. *Organic & Biomolecular Chemistry* **2006**, *4* (1), 47-50.
(30) Lu, Z.; Li, Y.; Deng, J.; Li, A. Total synthesis of the Daphniphyllum alkaloid daphenylline. *Nature Chemistry* **2013**, *5* (8), 679-684.
(31) Weiss, M. E.; Carreira, E. M. Total Synthesis of (+)-Daphmanidin E. *Angewandte Chemie International Edition* **2011**, *50* (48), 11501-11505.
(32) Li, J.; Zhang, W.; Zhang, F.; Chen, Y.; Li, A. Total Synthesis of Longeracinphyllin A. *Journal of the American Chemical Society* **2017**, *139* (42), 14893-14896.
(33) Sladojevich, F.; Michaelides, I. N.; Darses, B.; Ward, J. W.; Dixon, D. J. Expedient Route to the Functionalized Calyciphylline A-Type Skeleton via a Michael Addition–RCM Strategy. *Organic Letters* **2011**, *13* (19), 5132-5135.

(34) Shi, H.; Michaelides, I. N.; Darses, B.; Jakubec, P.; Nguyen, Q. N. N.; Paton, R. S.; Dixon, D. J. Total Synthesis of (−)-Himalensine A. *Journal of the American Chemical Society* **2017**, *139* (49), 17755-17758.
(35) Chen, Y.; Hu, J.; Guo, L.-D.; Zhong, W.; Ning, C.; Xu, J. A Concise Total Synthesis of (−)-Himalensine A. *Angewandte Chemie International Edition* **2019**, *58* (22), 7390-7394.
(36) Wang, B.; Xu, B.; Xun, W.; Guo, Y.; Zhang, J.; Qiu, F. G. A General Strategy for the Construction of Calyciphylline A-Type Alkaloids: Divergent Total Syntheses of (−)-Daphenylline and (−)-Himalensine A. *Angewandte Chemie International Edition* **2021**, *60* (17), 9439-9443.
(37) Zhang, H.; Shyaula, S. L.; Li, J.-Y.; Li, J.; Yue, J.-M. Himalensines A and B, Alkaloids from Daphniphyllum himalense. *Organic Letters* **2016**, *18* (5), 1202-1205.
(38) Jeschke, J.; Korb, M.; Rüffer, T.; Gäbler, C.; Lang, H. Atom Economic Ruthenium-Catalyzed Synthesis of Bulky β-Oxo Esters. *Advanced Synthesis & Catalysis* **2015**, *357* (18), 4069-4081.
(39) Nishioka, K.; Goto, H.; Sugimoto, H. Dual Catalyst System for Asymmetric Alternating Copolymerization of Carbon Dioxide and Cyclohexene Oxide with Chiral Aluminum Complexes: Lewis Base as Catalyst Activator and Lewis Acid as Monomer Activator. *Macromolecules* **2012**, *45* (20), 8172-8192.
(40) Del Valle, D. J.; Krische, M. J. Total Synthesis of (+)-Trienomycins A and F via C–C Bond-Forming Hydrogenation and Transfer Hydrogenation. *Journal of the American Chemical Society* **2013**, *135* (30), 10986-10989.
(41) Tiecco, M.; Testaferri, L.; Santi, C.; Tomassini, C.; Bonini, R.; Marini, F.; Bagnoli, L.; Temperini, A. A Chiral Electrophilic Selenium Reagent To Promote the Kinetic Resolution of Racemic Allylic Alcohols. *Organic Letters* **2004**, *6* (25), 4751-4753.
(42) Nicolaou, K. C.; Ding, H.; Richard, J.-A.; Chen, D. Y.-K. Total Synthesis of Echinopines A and B. *Journal of the American Chemical Society* **2010**, *132* (11), 3815-3818.
(43) Kobayashi, Y.; Feng, C.; Ikoma, A.; Ogawa, N.; Hirotsu, T. Synthesis of trans-2,6-Disubstituted Cyclohexanones through Allylic Substitution. *Organic Letters* **2014**, *16* (3), 760-763.
(44) Holub, N.; Neidhöfer, J.; Blechert, S. Total Synthesis of (+)-trans-195A. *Organic Letters* **2005**, *7* (7), 1227-1229.
(45) Renaud, P.; Ollivier, C.; Weber, V. One-Pot Rhodium(I)-Catalyzed Hydroboration of Alkenes: Radical Conjugate Addition. *The Journal of Organic Chemistry* **2003**, *68* (14), 5769-5772.
(46) Klahn, P.; Erhardt, H.; Kotthaus, A.; Kirsch, S. F. The Synthesis of α-Azidoesters and Geminal Triazides. *Angewandte Chemie International Edition* **2014**, *53* (30), 7913-7917.
(47) Panella, L.; Aleixandre, A. M.; Kruidhof, G. J.; Robertus, J.; Feringa, B. L.; de Vries, J. G.; Minnaard, A. J. Enantioselective Rh-Catalyzed Hydrogenation of N-Formyl Dehydroamino Esters with Monodentate Phosphoramidite Ligands. *The Journal of Organic Chemistry* **2006**, *71* (5), 2026-2036.
(48) Park, W. K. C.; Auer, M.; Jaksche, H.; Wong, C.-H. Rapid Combinatorial Synthesis of Aminoglycoside Antibiotic Mimetics: Use of a Polyethylene Glycol-Linked Amine and a Neamine-Derived Aldehyde in Multiple Component Condensation as a Strategy for the Discovery of New Inhibitors of the HIV RNA Rev Responsive Element. *Journal of the American Chemical Society* **1996**, *118* (42), 10150-10155.
(49) Ricardo, M. G.; Marrrero, J. F.; Valdés, O.; Rivera, D. G.; Wessjohann, L. A. A Peptide Backbone Stapling Strategy Enabled by the Multicomponent Incorporation of Amide N-Substituents. *Chemistry – A European Journal* **2019**, *25* (3), 769-774.
(50) Fehlhammer, W. P.; Schrölkamp, S.; Hoyer, M.; Hartl, H.; Beck, W. Alkaliisocyanacetate. Synthese und Struktur von [K(18-Krone-6)](O2CCH2NC). *Zeitschrift für anorganische und allgemeine Chemie* **2005**, *631* (15), 3025-3029.
